# Supplementary material for: “If It Works in People, Why Not Animals?”: A Qualitative Investigation of Antibiotic Use in Smallholder Livestock Settings in Rural West Bengal, India
Source: Antibiotics (Basel). 2021 Nov 23;10(12):1433. doi: 10.3390/antibiotics10121433 (PMC8698124; doi:10.3390/antibiotics10121433)
Supplement: Supplementary file 1 [file antibiotics-10-01433-s001.zip › Supplementary S1_ Interview Transcripts/Site 1/Homeopath 1 (site 1).pdf]

**Code for Study -** ‘If it works in people, why not animals?’: A qualitative investigation of antibiotic use in smallholder livestock settings in rural West Bengal, India: Homeopath 1, Site 1

**Date:** 21/11/2019

**Location:** Site 1

**Interviewee**: Homeopath- Antibiotic Provider to livestock

**Interviewer:** Mathew Hennessey (MH)

**Transcription:** Soumen Samanta (SS)

MH: Mathew Hennessey

SS: Soumen Samanta

SH: Stakeholder

MH: I Think we would start by asking him to tell us what type of work he does? How long he has been doing this for?

SS: What type of work do you do and how long you are doing this?

SH: *Redacted life history.* I am going through. And I will try to maintain that in my upcoming days. I have no degree, certificates, or institutional knowledge.

SS: Okay and what type of work do you do? Means treatments.

SH: In animal treatments there are different diseases in different aspects; it’s not possible for me to know the diseases name as I had not gone through to any training centre. I can’t tell those but traditional knowledge (I got) like in fever occurs, here few anthrax cases found..

MH: Does he treat different types of animals?

SS: What types of animals do you treat?

SH: In veterinary the livestock that means cows, with that poultry and ducks like animal.

SS: Cows, goats, poultry, duck all?

SH: Yes.

SS: birds?

SH: the birds' treatment has come recently, was not in training so it's not in my capacity, recently one thing came is Insemination (AI), it comes later that was not during us. But this Insemination which are done by pranibandhus nowadays, I had no training so I will not get those seeds, but any way, I can't control my work so I didn't give importance on that (AI).

SS: So you see only these parts; treatments?

SH: Yes, I am also getting older, (inaudible)

SS: He treats cattle, poultry, ducks, sheep and goats.

MH: Do you treat fish?

SH: No, no.

MH: Do you treat people as well as animal?

SH: in human (I use) homeopathy, in livestock we use homeopathy; in some condition allopathic medicine also.

MH: Do you use allopathic medicine for human?

SH: No.

MH: What type of allopathic medicine do you use for animals?

SH: Mainly in veterinary medicine those are available especially first of all Oxytetracycline. It is used heavily. Then comes Penicillin 10lakh, 20lakh; later in allopathy Ampicon(?) is used. Then Binocin® (ampicillin+cloxacillin) and these types of medicines; then neurobion forte,

SS: That is nerve tonic.

SH: Then conciplex,

SS: Then, Do you use these (indicating Ofloxacin medicine packet)?

SH: yes, yes.

(Shows Ofloxacin+tinidazole)

MH: What is the common type of medicines do you use?

SH: In ampicillin groups, as ampicillin is not available in market, **Amoxicillin** (Mox®), these type medicines are mostly used.

MH: Does he use these antibiotics as common as homeopathy?

SS: Do you use this antibiotic regularly?

SH: Yes.

MH: How many times in each week do you use these medicines?

SH: this veterinary, business depends on season totally. Like, this winter is off season; it is liked, in some case it cures with 1, 2 or 3 injections but according to disease we have to change the mode.

SS: How may case do you see in each week?

SH: it is not accurate; every day 3-5 cases come.

SS: In how many cases do you use antibiotics?

SH: According to conditions.

SS: Average?

SH: 2-3 (cases).

MH: What do you use in other 2 cases?

SH: It's according to case. Sometimes it seen that I left it with Homeopathy, I need not to go for allopathy.

MH: Out of 4 cases that you see, do you see certain type of animals more commonly? What are the most common types of animals do you see each day?

SH: Cows.

MH: How many cows do you see each day?

SH: 4, 5 or 6 and sometimes it also high up to 15-20.  It depends on season. Season wise disease, like human viral disease.

MH: What type of conditions, problems do you treat for?

SH: Different types of problems like mild fever; gas problem in any disease condition because they take the feed directly but we take our food after processing. So we don't have that problem, but in any diseases of them gas tendency occurs. It is situation wise.

SS: So, gas, fever and?

SH: injury, fracture, weakness due to short of vitamins.

SS: less eating?

SH: yes, yes.

MH: Do you go to peoples' houses or do they bring the animal to you here?

SH: They report me or call me and meet me, then I go to their houses.

MH: The antibiotics that you have here, where do you get them from?

SH: A shop in [village name redacted], [human drug shop name redacted]. And the veterinary medicines I take from [veterinary drug shop name redacted] shop in [nearest town name redacted]. The owner of that shop was a ex- veterinary surgeon. From there I use the medicine. And if urgently any medicine is needed I took it from local shop.

SS: Means human shop.

SH: yes, human shop.

SS: those two both are veterinary shop?

SH: no, no, both human and veterinary. In [nearest town name redacted] it is only veterinary.

MH: If you buy from local human pharmacy do you buy human antibiotics or do they sell veterinary antibiotics?

SS: Do you buy antibiotic from local human shops?

SH: suppose Neurobion forte injection or tablets are taken. D plus, gas medicine like omez (omeprazole) are taken, sometimes antibiotic **Sporidex (Cephalexin**) , different medicine according to disease, or paracetamol group etc.

MH: Is that cephalexin a human antibiotic?

SS: Yes.

MH: What do you use that (cephalexin) for?

SH: Sporidex is used in deeply wound which is not curing,

MH: What type of animal?

SS: In cows or which animal?

SH: in cows and Goats also.

MH: How do you decide which shop you will go to?

SH: It's area specific, when in which area I remain I used that area's medicine then.

SS: So you buy medicines from these 3 places?

SH: Yes.

MH: Are there any other places?

SH: area specific that I told, nearby market also according to need.

SS: From human shop?

SH: yes.

SS: So from different other local human shops also.

MH: Which is the most common place to buy antibiotics?

SH: for human, mostly [village name redacted] or local.

MH: Why it is more common than [nearest town name redacted]?

SH: It is nearby so. Like from [three village names in site 1 redacted] area that shop is nearby to them and easy to go

MH: Do you keep a stock of antibiotics like these?

SH: according to need less amount, for 3, 4 or 5 days medicine.

MH: When you see an animal do you write a prescription for people to go to buy antibiotics from somewhere else?

SH: Yes, I writes and they take from their easy places.

MH: Which is more common to write a prescription or to give them medication?

SH: there are many reasons, like people are of different types, and this village area is of poor people's area. If I give antibiotics and it worked, so then they will not come. So what benefit I hope from him it goes into loss account then as he not comes after that. And when he will come next I couldn't remember then.

And other thing is that this Sporidex 500mg capsule's price is 14-18rupees. So if 4 capsules go instead of 1 capsule it hurts them. So I write and I have no contract with the shops that you have to give me the percentages. Where ever they get they use that.

And another thing is also of invest; what I want is they live with food.

SS: So you prescribe and they do at their choice?

SH: they do themselves.

MH: So it's more common to write prescriptions?

SS: Yes.

MH: Do you have any concern about the quality of different antibiotic that you use?

SS: Do you think any antibiotic is better than other?

SH: Suppose what we used previously Penicillin, now what is available in veterinary that is of 40lakhs, it becoming problematic as because it is having a dose; and when I am going from lower power to higher power I can use the lower power easily like 10lakhs or 20lakhs. And it is according to case. So when I have to use 10lakhs from 40laks (vial) it is very problematic.

SS: So which antibiotic you use more? Penicillin?

SH: penicillin, oxytetracycline.

SS: it is better than other antibiotic?

SH: better. Use mostly.

MH: Why do you think that?

SH: Depends on its work.

SS: Animal gets cured?

SH: Yes.

MH: What happens with the other antibiotics? Why do you think they not work?

SS: Does the other antibiotic not work?

SH: one thing is that mainly in our business it is pulling each other's leg business. Mainly the others who practise with us, they want; or say about any training centre, seminar there is matter of discussion where many doctors meet together but I have no such scope. I am sitting in the same place, so I am going with my old theory. And by gods shake, the work is going well. And peoples call me.

SS: So you get results by using these two medicines?

SH: Yes getting results, and sometimes in some case I ask [name of ex-surgeon running the veterinary drug shop redacted], he tells 'give these medicines or send the case to me'. I don't want to take the risk when the case is critical as we can guess what fate of the case may be. Then I told them to go to other doctors. I want my goodwill stay in position. (Reputation).

MH: Which of the doctors you send them to?

SH: the name I said [name of ex-veterinary surgeon running the veterinary drug shop redacted].

SS: Who is he? Veterinarian?

SH: he is ex surgeon.

MH: the retired vet of [nearest town name redacted]?

SH: He was in [nearest town name redacted]; and [the block name is redacted]

MH: Do you work with any other local pranibondhu or animal health workers?

SH: No.

MH: Are there other people like you who are homeopathic?

SH: Homeopathy in veterinary, there is one in my village, [name redacted]. He practise veterinary and use homeopathy sometimes. And the [name of ex-veterinary surgeon running the veterinary drug shop redacted], he also use sometimes homeopathy. Otherwise these pranibondhus don’t know the things. They don’t know the application of homeopathy.

MH: Do you work in similar way to him? Or are there some differences in the way you work?

SS: Do you work with that homeopathic vet?

SH: No.

SS: No contact with him, different way of practise.

MH: What do you do when an antibiotic becomes expired?

SH: throw away in dust bin.

MH: So one of the ways of our project is to improving the access to antibiotics and how they would be used. What do you think about the ways it could be done?

SS: Can we cure the animal with antibiotics or it can be done without that?

SH: Couldn’t get the question?

SS: What we want is good animal productions, so in that case how important antibiotic is?

SH: without antibiotic disease can’t be cured.

MH: Do you think anything need to be changed the way antibiotics are used?

SH: Of course as new things are coming nowadays, new research is going on, new medicines are coming. Side effects of old medicines are coming that I can’t see but heard in the market that these things are coming. Govt is looking towards it, research is going on. Ever body hopes new things, ‘materia medica’, which medicine in which condition to be used or that are being used

now how effective they are, or how more effective it could be; these we all hope. All want the progress of science.

MH: Do you know why old antibiotics would not be working any more?

SH : no, I don't say that it not works. If the new things come like 'materia medica'which tells about the disease, I am using the old one, the new that are coming ..(not understandable). Our aim of this establishment is that people not get any suffering.

SS: So you want the new one besides those old one also?

SH: Yes, yes.

MH: Do you have any contact with the medical representative from drug companies?

SS: Do have contact with those medicos who supply drugs?

SH: No, no.

MH: Do you use the animal antibiotic to the people?

SH: No, no. only for veterinary treatments. Or only for human treatments.

MH: Why do you that people will not use animal antibiotic for them?

SS: Why do you think that animal antibiotic can't be used for human?

SH: the company instructions, there is written on medicine that 'only for veterinary use'. But the human medicines are used easily for animal. I have no medicine of human, I use homeopathy for them, but in veterinary case the human medicine that can be used are used heavily.

MH: Thank you.
