# Supplementary material for: “If It Works in People, Why Not Animals?”: A Qualitative Investigation of Antibiotic Use in Smallholder Livestock Settings in Rural West Bengal, India
Source: Antibiotics (Basel). 2021 Nov 23;10(12):1433. doi: 10.3390/antibiotics10121433 (PMC8698124; doi:10.3390/antibiotics10121433)
Supplement: Supplementary file 1 [file antibiotics-10-01433-s001.zip › Supplementary S1_ Interview Transcripts/Site 1/Human Drug Shop 1 (site 1).pdf]

Code for Study - ‘If it works in people, why not animals?’: A qualitative investigation of antibiotic use in smallholder livestock settings in rural West Bengal, India: Human drug shop 1, Site 1
Date: 11/07/2019
Location: Site 1
Interviewee: Human Drug Shop 1- Antibiotic Provider
Interviewer: Jean-Christophe Arnold (J-CA)
Transcription: Debanjan Debnath (DD)

I: Interviewer (JCA)
P: Participant (Human drug shop 1, Site 1)

START OF INTERVIEW

I: What's your role to the people of [village name redacted]?

P: They come to my pharmacy to see doctors, then they sometimes get medicines from the store. People of [village name redacted] are mostly poor, they don't have a lot of money. So, sometime even if the doctor has given medicine for seven days, they would take medicines for two days, we do that.

I: For whom do you give medicines?

P: Whoever comes to the shop to get the medicines, since we can't really go to them.

I: Do you also serve people from outside of [village name redacted]?

P: Yes, people come neighboring villages. (Names that are mentioned are indistinct because of the surrounding noise)

I: How is your career until now?

P: After finishing education you need to choose a career. So, I started my career like this. In order to open a pharmacy, you need a drug license, so all that was done! my career is going good.

I: What did you study before becoming a pharmacist?

P: We are not pharmacists. They are different.

I: Oh, I thought you mentioned drug license...

P: No, we got a drug license. We just have to keep a license. We are not actually pharmacists.

I: What did you study before this?

P: I passed Higher Secondary Exams.

I: So, what does the drug license do?

P: It has a registration number for the shop.

I: Is it different to a pharmacist certificate?

P: Yes, It's different.

I: Did you receive any training?

P: I have a brother who's a doctor, I have worked with him for 5 years. I gained some experience there, for the license you needed to have experience.

I: Like an experience certificate?

P: Yes.

I: How long are you doing this for?

P: 15 years.

I: Is it private or public?

P: It's private.

I: Why do you do this?

P: After finishing my studies I needed to get into a career, then start a family. Everyone needs to do the same thing. I had to do the same thing, if I weren't doing this, I would have done something else. I would have worked at a company if I didn't start my drug business, I needed to something or other.

I: Did you do anything else before this?

P: No!

I: Do you sell antibiotics?

P: Yes, all pharmacies have antibiotics.

I: What antibiotics do you stock?

P: There are many kinds.

I: Would you name a few of them that are very common?

P: Amoxicillin, Ampicillin, Cloxacillin, Potassium Carbonite.

I: For which reasons people come to him for antibiotics?

P: For fever, cold, cough.

I: Have you given antibiotics without a prescription?

P: Yes, you are supposed to give prescription. But sometimes the patient would just come and say that they have fever, or cough then we give it to them.

I: Has there been any situation where you haven't given any antibiotics to the clients?

P: If someone comes and says that they have fever, we would just give something for the fever.

I: You don't give antibiotics then?

P: No.

I: When you're giving antibiotics to someone how do you decide which antibiotics to give?

P: See, the doctors that see the patients, they prescribe antibiotics based on the symptoms. So, we have the understanding.

I: Do you stock any antibiotics for use in animals?

P: No, we don't keep it. But a lot of people raise chickens. They come and say that the chickens (and such) are having irregular stool. They know what medicine they want; they would ask for O2 (tablets). If I ask them why they would tell me it's for their chicken.

I: Is O2 an antibiotic?

P: Yes.

I: In which other cases would you give antibiotics?

P: Sometimes even human antibiotics are used in animals. For example, cephalexin doxycycline are medicines that work well in animals as well as humans.

I: For which problems in animals would people come to you?

P: There's a veterinarian [LDA] at the GP here, we could perhaps prescribe ciprofloxacin 500, we give it, or Calpol 650, we give that.

I: Without prescription how would you know how much medicine to give for the animals?

P: We don't want to take that risk. Maybe the cow was going to die anyway, but If it dies after taking medicines from my store if could create a problem. We still give medicine if there's an emergency. Otherwise it's preferred that you have a prescription from a veterinarian, now there's one in the GP office, you can see him there. In case he isn't there, we try and give medicines understanding the symptoms, we suggest the dose.

I: The medicines that you give the animals, how are they given to the animals?

P: We give the medicines. If they ask, we say you have to dust the tablet and mix it with water and give it to the animals. It gets absorbed soon. If it's a dispersible tablet it gets mixed fast. Other ones don't mix so fast, so you have to break it and put it in the water.

I: Which is the most common animal that you provide medicine for?

P: Chickens.

I: How do you know which antibiotics to give?

P: A lot of people raise chickens here, for the eggs. They only come to us for medicines.

I: Apart from treating diseases do you give medicines to animals for any other reason?

P: No, there's no question. If you don't have a disease, why would I give you medicine?

I: Sometimes medicines can also be given for prevention of the diseases. Or to help with growth.

P: There are certain vaccines for pox, hepatitis etc. Those aren't given without doctor's advice.

I: When you've given antibiotics to people do you explain to the clients how antibiotics work?

P: Sometimes people come asking for antibiotics. what prevents a disease, isn't that what antibiotics are? To kill the bacteria. The clients don't ask that. They would perhaps ask for antibiotics saying that they had been taking antibiotics from different sources which didn't work, so they would ask for something else.

I: Why do you think people come here for treatment of their animals?

P: Because I have a drug store. If I were a general person they would not have come!

I: Why do people come to you without going to veterinary drug store or doctors?

P: I'll tell you something, there's no veterinary medicine shop in this area. The nearest shop is 6KMs away at [nearest town name redacted], where there's a proper veterinary medicine shop. Some stores might keep one or two drugs.

I: That's why?

P: (nods)

I: When you're giving medicine to for the animals, what is usually the duration that you give the medicine for?

P: That, they would just ask for two tablets. or one.

I: The medicines that you give, they are generally for human use which is also used in chickens?

P: Yes. But it actually works. A lot of people in this area keep birds now a days. When they have diarrhea, they get O2 syrup, Metrogyl syrup (Metronidazole), vitamins, etc.

I: What is the difference between human antibiotics and animal antibiotics?

P: We don't exactly know what animal antibiotics are. But we have the same antibiotics in both cases. For example, cephalexin, ofloxacin, etc. I think the power is different.

I: Are you aware of guidelines for antibiotic use?

P: Not really. We don't really know what good and what bad these antibiotics do.

I: Do you have any idea as to how to regulate antibiotic use?

P: We have to maintain the dose depending on the body weight.

I: Did you have any other training apart from the training you mentioned?

P: Well I worked with a doctor... as I mentioned.

I: Anything else?

P: No! .

I: Do you have other opportunities to receive more training in the future?

P: No!

I: Why not?
P: We don't have the time. If we are to go somewhere to receive some sort of training, we won't have time for it. I have to run the shop as well.

I: If he had the time and the opportunities would you be interested in doing it?

P: If I had, I would be interested.

I: Are you part of any organization within your profession?

P: No!

I: We are finished, you have been very helpful, thank you!

END OF INTERVIEW
