# Supplementary material for: “If It Works in People, Why Not Animals?”: A Qualitative Investigation of Antibiotic Use in Smallholder Livestock Settings in Rural West Bengal, India
Source: Antibiotics (Basel). 2021 Nov 23;10(12):1433. doi: 10.3390/antibiotics10121433 (PMC8698124; doi:10.3390/antibiotics10121433)
Supplement: Supplementary file 1 [file antibiotics-10-01433-s001.zip › Supplementary S1_ Interview Transcripts/Site 1/Informal Provider 1 (site 1).pdf]

Code for Study - ‘If it works in people, why not animals?’: A qualitative investigation of antibiotic use in smallholder livestock settings in rural West Bengal, India: IP 1, Site 1
Date: 08/07/2019
Location: Site 1
Interviewee: Informal Provider of Human Health (IP)- Antibiotic Provider
Interviewer: Jean-Christophe Arnold (J-CA)
Transcription: Debanjan Debnath (DD)

I: Interviewer (JCA)
P: Participant (IP1))

START OF INTERVIEW

I: What role do you play in this village?

P: I am a physician, villagers come to me with their problem. When they are sick, they come! Not just this village, people from neighboring villages also come.

I: Whom do you treat?

P: Humans.

I: Do you serve people outside of the village?

P: Yes.

I: How do you treat people from outside of the village, if you could describe that.

P: Outside the village I have a clinic. I sit there. People come there with their problems.

I: How do people in [village name redacted] receive treatment from you?

P: Whatever problem people have; they come and tell me. I give treatment basing on that. Antibiotics and others.

I: Do you give treatments to same people many times (clients come back to you many times) or is it normally new people?

P: Not a lot of new people, it's usually the same people that come at different time. And also, people from other villages, neighbors, etc.

I: How many years have you been practicing?

P: 40 years.

I: Is your practice public or private?

P: Private.

I: Why did you want to be a doctor?

P: There were no other doctors in the village. We had to travel long distances to get treatment. So, my ambition has always been to be become a doctor.

I: What did you do before that?

P: I was studying.

I: What were you studying?

P: I was a student of science. Chemistry, Physics and Biology.

I: At what level did you study?

P: I dropped out at graduation. I failed in Chemistry.

I: Do you stock antibiotic drugs?

P: Yes, most chambers have antibiotics. if you want me to tell you about antibiotics I can. ciprofloxacin, (unclear drug name) etc.

I: If you could say which antibiotics do you have and why you have them?

P: When we see that someone is suffering from pneumonia, bronchitis, tuberculosis then we give antibiotics from amoxicillin group, to reduce the cold. If someone has trouble with the suspension that we give them (indistinct) suspension injection and Asthalin tablets and we give antibiotic injections to dissolve it.

I: Apart from these, what are the other reasons you keep antibiotics?

P: if someone has a boil, to cut it and take the pus out, and someone has an injury, after stitching, to heal the wound, we use antibiotics. Also, for infections, we use antibiotics.

I: Do people in the village understand what antibiotics are?

P: No, people here don't know about antibiotics. Very little percentage of people is literate here. So, they don't have an idea about antibiotics. Only a 5% of the villagers are educated.

I: Apart from antibiotics what sort of medicines do you keep?

P: If there's an infection, it's swollen and it hurts then we use different medications (term indistinct), or medicines for high temperature, or medicines for high pressure. There are different medicines for different problems.

I: Do you keep any medicine for animal treatment?

P: No! I don't. Only for humans.

I: Why?

P: There are other doctors for animals.

I: Are there other reasons?

P: After they see the doctor, if they are to give injections, since the doctor won't come every day. Then sometimes I go and do it

I: Which medicines do you give to the animals, as you said, when the doctor isn't here?

P: I follow what the doctor had advised. Or Clean the wound and apply the medicine, as per his instructions.

I: Where do you get the medicines from?

P: Mostly [nearby town name redacted].

I: That's where you get the medicines from?

P: (Nods)

I: The antibiotics that are given, are they human or animal antibiotics?

P: Animal antibiotics. Meant just for animals.

I: How did you learn how to give antibiotics or other drugs to animals?

P: [nearby town name redacted] has an animal hospital. We received a 15day training from the Surgeon there.

I: Do people in the village come to you for treatment of their animals?

P: Yes. I give some advice and prescribe medicine. They get it.

I: What sort of medicine do you prescribe?

P: Mostly medicines ((Indistinct drug), Gentamicin, Terramycin group) for swelling of the throat, cough.

I: The antibiotics that you have to the animals, do you have it here?

P: No. The client gets it after I prescribe them. I stock some human medicines.

I: The antibiotics you give to the animals, are they animal antibiotics?

P: Animal antibiotics. Animal antibiotics are different. It's in the same group but it's different. It's written on the medicine. It's completely different thing. In certain cases, there are some similarities, for example Terramycin, Gentamicin, Amoxicillin. They have the same name.

I: Where do people go after you've prescribed a medicine?

P: [nearby town name redacted]

I: What sort of shops are there?

P: Veterinary medicine shops. Veterinary medicine shops are different.

I: What happens next?

P: Then I give the injection. Ones that are meant to fed, they need to be fed.

I: which are the medicines that are given?

P: If they are antibiotic injections, I give it. and the tablets are fed according to instructions.

I: When medicines are brought for you to give the animals, how do you understand that the medicines are meant for animals?

P: It is mentioned. Only for animal use.

I: Has there been any situation where you have given human antibiotics for animals?

P: No.

I: When he treats human and animals do you explain how the drugs work to the client?

P: No!

I: Why not?

P: No one wants to know the composition of the medicine.

I: When you are giving treatment, what do you say to them about the treatment?

P: Imagine a patient come with 104 fever, and cough, I would do a checkup and probably give a cough syrup, an antibiotic for three days and a paracetamol for the temperature.

I: I am asking what you say to them...

P: I'd say have it for seven days, your dose will be completed. If the antibiotic is given for five days, the dose is completed.

I: Do you say anything else?

P: We tell them you have to finish a seven days dose! You have to complete the antibiotic dose, otherwise it might relapse. Sometimes we give medicines for two days for the money they can afford, and we tell them to get medicines for five more days. You have the dose of seven days. Sometimes they come, sometimes they don't.

I: Does it happen often that people don't complete their antibiotic dose?

P: In most cases it happens. Only the aware and conscious clients complete their courses.

I: I am just repeating the question. Do people here complete their antibiotic course?

P: Very few people do. People are not very aware, they don't know. People don't know about antibiotics, only the educated villagers would complete the courses.

I: Why do they do that?

P: They are poor.

I: How do you understand whether the treatment has worked?

P: We usually give medicine for three days; we know in three days the animals will get better. one third of the patients will come and tell us that they are better now.

I: Has there been any situation where the antibiotics didn't work?

P: No, they work! There are some antibiotics that we trust, some good company, good medicine, they work. We prescribe them!

I: How did you know which antibiotics to give?

P: See, we know which medicines to use for different kinds of infections.

I: When you prescribe antibiotics for animals, how do you ask the clients how to give the antibiotics to the animals?

P: We tell them to give the tablets thrice or twice a day.

I: Why do you think people come to you for treatment of their animals?

P: They believe that the medicines I am going to prescribe, works!

I: Could there be any other reason that the people come to you instead of going to an animal doctor?

P: I have an experience, I use that. That's what people know.

I: When you write a prescription for animals, what do you write there?

P: We write only for cows, or animals. We write the name of the medicine.

I: How do you understand what dose to use?

P: There are some medicines that are given thrice a day, some are given twice a day, and some are only given when required.

I: How do you know what amount to use?

P: The amount is mentioned on the case of the medicine. When we see it's a serious infection, antibiotics are given thrice a day. The amount is also written on the injection. The advised amount is given on the medicine itself.

I: Is it the same for all animals?

P: (... continues) In case of injection whether to give 5ml or 3ml is written on the case. For the liquid medicine it's written how many spoons are to the given, for tablets the physician decides how much to give depending on the situation.

I: Is it the same for all animals?

P: Yes, whatever the physician advices that is done.

I: How is the amount mentioned on the case?

P: It says "two spoon full" for syrup. then "as advised by physician".

I: What about injections?

P: In case of injections, as we know, there's a general dose. In some cases, you give 2ml, in some cases we give 5ml. We learn it from the books.

I: What do you mean by "general dose that are to be given"?

P: It depends on the infection. For example, you have a boil, in that case the tablet is not going to work, an injection would work better. It also depends on the age. Different dose for people from different age.

I: When you treat animals how do you decide which amount to give them?

P: there's a system at place. A minimum of three doses is important.

I: What difference do you understand between human antibiotics and animal antibiotics?

P: There is a difference. Because, see, when it comes to the animals, we use a 1000-unit injection, but we can't do that in case of humans. There's a difference in power.

I: Do you know of any other differences?

P: for animals the dose is twice powerful compared to humans, we know this much.

I: Do people also come here just for your advice?

P: No, they only come when they have a problem.

I: When people come to you with their problems in which situations or how often would you give them medicines?

P: Depending on their condition I decide which dose to give. Imagine some has high fever, after the primary medication we ask for a blood test, after seeing the repost I get confirmed which antibiotics to give them.

I: Do you examine the animals?

P: When you touch an animal, you understand the temperature. They might have already seen a doctor and they just want me to give the injection. I would just give whatever antibiotics the patients have bought.

I: Have you ever given antibiotics to animals without seeing them?

P: No, can't give medicines without seeing them.

I: Have you given treatment to humans without seeing them?

P: No, I usually don't give treatment without seeing them. In some rare cases if some comes and asks for medicines for fever for someone in the family, I'd give two tablets to reduce the temperature.

I: Has this ever happened for animals?

P: For diarrhea, we sometimes give medicines if they come and ask.

I: Which medicines are given in this case?

P: "Sulfagoanite" composition. There are many tablets. There are tablets like O2.

I: Are there antibiotics?

P: Ciprofloxacin, Ofloxacin are all antibiotics.

I: Are there any other cases you'd give medicine without seeing the patients?

P: Just diarrhea, or fever, that's it.

I: Are there any situations where you haven't given antibiotics to cows/animals?

P: Yes.

I: Both human and animals?

P: yes.

I: In which cases would you not give antibiotics?

P: In primary stages, I don't. We select the antibiotics depending on the situation. (unclear answer)

I: What criteria do you look for when you're giving antibiotics to humans?

P: When narrow spectrum and broad spectrum is badly affected by a bacteria when we have to use antibiotics. But when it's just a simple fever, we don't use antibiotics and use paracetamol for the temperature instead.

I: What about for animals?

P: It's the same.

I: How do you diagnose humans? Which diseases they have and which medicines to use?

P: It depends on the condition of the patient. How long he's been suffering.

I: How do you know which disease he has?

P: By his symptoms.

I: Why?

P: It depends on the symptom. Also, how long they have been suffering.

I: Have you ever tried to find out which bacteria is causing the disease?

P: Yeah, with blood test. We are not supposed to give medicines without knowing.

I: In which situations would you ask for a blood test?

P: If a patient is not recovering after three days of medication. Then we ask for a blood test. Then we get confirmed what's causing the problem then we can give medications accordingly.

I: How often does it happen that you would ask for a patient to get a blood test?

P: After three days of primary medication, we ask for a blood test, then we ask them to complete the dose.

I: Where do you get the tests done from?

P: There's a pathology in [town name redacted outside of Site 1]

I: Have you ever asked for tests in animals?

P: No.

I: Why not?

P: You don't get tests for animals here; we treat basing on the symptoms.

I: Why do you think that happens? Why do you think tests for animals aren't available?

P: We know that they are herbivores, they are likely to have stomach issues. They consume the grass and eventually fall sick because of Virus or Bacteria. There are medicines for Diarrhea what is given in those cases.

I: When you give antibiotics for human or animals, do you mention whether we should use human antibiotics in animals or vice versa?

P: We give the medicines accordingly, Human medications for humans, Animal medications for animals.

I: Do you explain it to your clients?

P: Yes.

I: What training did you have before you started practicing?

P: There have been veterinary camps, boys from different areas went there to receive training. For human treatment as I told you Studies, training then exam. I am a pharmacist; we know how medicines are manufactured.

I: Are you a qualified pharmacist? Do you have a certificate?

P: Yes, I have a certificate.

I: Did you do any additional training for this job?

P: I was working with the surgeon at the hospital for three years.

I: Do you know about the guidelines about antibiotic use?

P: Antibiotics are usually used for five to seven days.

I: What do you know about regulations of antibiotic use?

P: Antibiotics can be given as capsules, injections.

I: Do you know how to control the use of antibiotics?

P: Well, as for control, we know when antibiotics have to be used and we know which dose to give them.

I: Do you know of any laws for antibiotic use?

P: No!

I: Are you a member of any organization in your profession?

P: No.

I: The interview is finished.

END OF INTERVIEW
