# Supplementary material for: “If It Works in People, Why Not Animals?”: A Qualitative Investigation of Antibiotic Use in Smallholder Livestock Settings in Rural West Bengal, India
Source: Antibiotics (Basel). 2021 Nov 23;10(12):1433. doi: 10.3390/antibiotics10121433 (PMC8698124; doi:10.3390/antibiotics10121433)
Supplement: Supplementary file 1 [file antibiotics-10-01433-s001.zip › Supplementary S1_ Interview Transcripts/Site 1/Informal Provider 2 (site 1).pdf]

Code for Study - ‘If it works in people, why not animals?’: A qualitative investigation of antibiotic use in smallholder livestock settings in rural West Bengal, India: IP2, Site 1

Date: 27/07/2019

Location: Site 1

Interviewee: Informal Provider of Human Health (IP)- antibiotic provider

Interviewer: Dominic Day (DD)

Translation: Somraj Das (SD)

Transcription: Sayak Manna (SM)

D: Interviewer (DD)

B: Translator (SD)

J: Interviewee (IP2)

START OF INTERVIEW

D: So could you explain what your role is in the community?
B: What's your role here in this area?
J: I work as a health worker, in our village we don't have qualified doctors. If they are around then may be in a week for 1-1.5 hours or so, so they don't stick around everyday. The poor villagers can't travel 20kms to the hospital so we give them primary allopathy treatment to them. We don't try to treat the serious cases. Whatever little idea we have on the basis of that if we can figure out someone is serious, we try to get them to the hospitals. We even go along with them.
B: He actually works here as a social worker in this community, as a medical social worker. Just because there is a lack of qualified doctors in area, so sometimes you can get them or meet them for 2-3 days for an hour in a week. So in this case, the local villagers cannot make it to 20km, faraway for qualified doctors. In this case he works as a health (..?) or something. And there's one thing, if he sees there's a serious condition a patient is having then he tries to a better hospital and in that case sometimes they go with them. To the hospital to get them admitted!
D: Okay, great thank you. Erm, and who is he normally serve?
B: Whom do you serve?
J: Actually new born baby to adults (70-80 years).
B: From infant to 70/80 aged people.
D: Ok, and why's it those people?
B: Why do you serve them?
J: I can't understand.

B: Why do you serve them, from infant to 70/80years old people, why not other people of other age?
J: People who are above 80/90 they seldom come. As they are old and serious we don't usually treat them.
B: Generally from infant to 70/80, they are more. More than 80/90 years old, in that case they generally do not take the risk to care of them.
D: Umm, do you wanna..(..?)
(Unnecessary talks)
D: So how long have you been doing this from?
B: How long you are doing this?
J: about 12 years.
B: 12 years.
D: And what made you decide, you wanted to do this?
B: What made you think to do this?
J: *Life history redacted.* Suppose someone got cut or burnt and he needs immediate first-aid but we can't give him, may be a qualified doctor could have dressed the wound or given some injections but it's pretty far and can't get the treatment. They have to go to the far away hospitals. also we have problems with transport facilities. At night, we don't get vehicles, recently the transport improved a bit but 10 years back, there wasn't any facility. So we decided since we experience these at home also in the neighbourhood, why not we get some minimalistic training and coverup these issues momentarily. If we can give the first aid atleast and spend the night. or meanwhile arrange some cars to take them to hospital then it would be good! Hence I prefered to come to this line!
B: Actually he witnessed in his personal life that there's a situation at mignight that got worse, emergency case, *Life history redacted*, that is a one thing and before 10 years back there was less availability of transport. Suppose there is a bruise, minor treatment and first aid is required and people don't get them, so these kind of experiences that made him choose the career.
D: Ok and why is it in his opinion that people come to him.
B: Why people come to you?
J: As I said, there is no qualified doctors around, if there were doctors 24x7 then we need not be here. They are available 20 kms away. There are many qualified doctors who charge 300 rupees/visit, there's travelling too, the people are ignorant and poor, can't travel all by themselves, they have to get a company too. So to treat a patient one has to spend 700/800 rupees that would cover travelling, doctors fees and the expense of the helper. Plus you have the medicine, so they can't bear this much! Hence they first come to us. If we see something serious then we don't touch the person, just give the first aid and send the person away. If the case isn't that serious and is within our capability, we do what is necessary. And as the cost would be less so they would prefer to come to us.
B: Just because there's a lack of qualified doctor, they do it around here. It takes 20 km from here to get to a qualified doctor, since there's no 24x7 doctors here, so they give the first aid and all. The cost of going to a doctor 20km away is high. Going to an experienced doctor/vet is around Rs 300 and also, there's transportation. The back and forth would be around 700 rupees that the poor people can't afford.
D: Ok, What did he do before this job?

B: What did you do before this job?
J: I used to study.
B: He was in studies.
D: studies! Ok, What did he study?
B: What did you study?
J: HS (Higher secondary)
B: HS. In local board or Government system in education as 12 grade.
D: Ok and what subjects?
B: Which subjects?
J: Commerce!
B:Kho-morse!
D: So what..how did you get from commerce into this role?
B: How suddenly from commerce to this field?
J: I always wanted to be in this line. Since childhood, we are not very stable. So studying science was never in our cards. But in my mind I wanted to be someone like this as I have seen these problems. I tried to study science but couldn't get the stream also *Life history redacted*. So since childhood we were pretty much separated! We were very poor. When I was in class 6 since then I used to give tuitions and continued my studies!
B: *Life history redacted* and in his 12 grades he wanted to take up science or at least in his bachelors, unfortunately he couldn't. But there was an aspiration of him, he wanted to do something like that but he couldn't go for the science stream, one. *Life history redacted.* They usually got treated separately, that made him financially weaker and couldn't get the education. But there's an aspiration in him to be in this stream or career.
D: Okay, thank you. I like to talk to you about antibiotics.
B: I will talk about antibiotics.
J: Ok, I have 10 years of experience in nursing. In (..?) medical college I took some classes and 10 years nursing training.
B: He has taken a 10 years nursing training addition with 2 years medical training.
D: Ok, and where did you do this training?
B: From where you did the training?
J: *Life history redacted* nursing home.
D: Provides these training?
B: Who gave these trainings?
J: Actually in nursing home what happens is many doctors come from reputed Government hospitals like PG, NRS and medical college, they are from Kolkata, they set up their chambers here, I used to stay and work with them. Plus at OT I used to work as an assistant! So I learnt all of these by being with them. With the doctors I discussed about many diseases and medicines, have taken notes, I have a lot of notes on medicine and surgery. The RMO sir used to give me personal classes at night, (*Name redacted. Medical doctor)*. So I learned a lot from him, the signs and symptoms of diseases and the complication, medications and side effects, what medicines can't be given at which diseases etc. Which are the ones can be avoided especially some of them. So we got these trainings from him. So there are certain medicines that we never use because of the side effects!
B: There are renowned hospitals in Kolkata, from there doctors come to Diamond harbour

hospitals for general consultation and OPDs as well. At their supervision he was there. He was an operating technical assistant. And that is the reason he was there. Afterwards the doctors, he usually consult (Talks*) with him about the medicines and diseases, sometimes till the midnight. They helped him a lot about acquiring those medical knowledges. And it's more than 10 years he is doing that. They usually provided them the training. By doing long discussions and practical practices about the medicines. And side affects of the medicines and which medicines is supposed to be given at what kind of diseases. Which medicines are supposed to be avoided in which particular cases, these are the things. They actually gave them notes and he has many notes on these.
D: Okay, And did you acquire a qualification from this?
B: Have you got any degree from there?
J: No. From nursing home you can't get a degree, no one can give you a degree. If courses are taken from Govt. Hospitals then you can get a degree but not from this one!
B: generally nursing home do not provide any degree or qualifying certificate, if you are doing the same thing from a Govt college they do provide, degrees and certification and all.
D: Could you explain which antibiotics you stalk in here?
B: Which types of antibiotics you keep?
J: Actually amoxicillin, (..?) potassium, cephlixillin, cephodoxin, cephofloxicin, o-floxicin, clarithromycin, lymphomycin, metronizodol, ornidozol etc, Amphicilin, gentamycin, amigacin
D: Thank you. And what are the common reasons you prescribe these?
B:What the reasons you presribe these antibiotics?
J: Actually, when we can undertsand that there's a viral infection then we don't give any antibiotics. We observe for 2/3 days, we give paracetamol or antiallergic, antihistamin we observe for 2/3 days. If after the observation period we see it's cure then it's fine, if not then we give antibiotic. In other cases like pneumonia, bronchitis, laryngitis, pharyngitis, tonsillitis we give antibotics. Fever with running nose, nasal congestion, when these patients come, we think there must be a viral infection, we give paracetamol and antihistamin for 2 days, if not cured then we apply antibiotics!
B: This case, he just named some of the antibiotics. First of all if they see, someone is coming with a viral fever, they directly at the first moment do not prescribe antibiotics. They give paracetamol which are not antibiotic and observe for 2/3 days. If he sees the disease is not getting reduced and the intensity of symptoms are not getting reduced, then he prescribes medicine which are called antibiotics.
D: Ok, And do you have the same clients coming back again and again or lots of different people?
B: Do same people come to you or different people come to you?
J: No, both same and different people come to me.
B: Both. Old people come regularly, sometimes new people arrive here!
D: Ok, so when you prescribe antibiotics, do you usually administer them, could you explain what instructions you give when you prescribe antibiotics?
B: when you give antibiotics what instructions you give?
J: Antibiotics have different courses. If I give amoxicillin (..?) potassium powder, adult dose, so we tell them 650 3 times per day for 7 days. If cephodoxin then 200 then BD 2 times daily 5 days. Cephaxazin same dose. So we basically work with Cephaxazin, cephodoxin and

amoxicillin.
B: (I am not repeating it, because he is talking SHIT)
D: Could you explain why you give this doses?
B: Why these doses?
J: This is what I learnt from the beginning. Learnt from the doctors, read (..?) like Drug today, there the doses are mentioned in that way. Some are BDS, some are BD, some are TDS etc. We give accordingly. Actually we give on per kg body weight, so we give on the basis of that. We give cephodoxin per kg body weight, suppose 10 mg, we calculate how much per kg and then divide by 2 and recommend it for 5 days. This is the way.
B: He got the training to provide it. They have to give antibiotic, so they calculate on the basis of body weight and multiply by 2.
D: On what situation sir?
B: On what situation you give by multiplying it with 2 per Kg?
J: What?
B: You just said you multiply by 2 so, how do you do the calculation? In what situation?
J: Suppose cephodoxin 10mg per Kg body weight. Now we measure the body weight of the person, for an instance his body weight is 50kg, then it is 50x10, which is 500. Now cephodoxin is not more than 200, in that case if we divide 500/2 it is 250 each. Now in the market you can't get cephodoxin 250, you only get 200. So we give 200 only.
D: Okay and where do you get your informations on antibiotics?
B: where do you get your informations on antibiotics?
J: Medical Journals.
B: He's talking about medical journals. There're some Sims and Medical Today (Drug* Today), where he gets the information on antibiotics from
D: And are you part of a professional body?
B: Are associated with any professional organisation?
J: Right now I am not but I long to work with some organisation, but I never got a chance.
B: Well he wants to be a part of any professional organization but right now he's not!
D: Okay, And there professional bodies who carry out your role?
B: Come again?
D: Are there professional bodies or people that carry out the same role?
B: Do you think the professional bodies have the similar role like yours?
J: What is professional body? I can't understand.
B: Professional organisation!
J: You mean the qualified doctors?
B: What's everybody's role? He's not getting your point
D: Ok, do other medical providers, informal medical providers have organisation (..?)
B: Are the medical providers part of the professional organisations sometimes?
J: No. No. The one who gives medicines? No they are not.
B: Medical providers are not the part of professional organisations.
D: No, ok Thank you
J: Actually we are doing these thing illegally, we are from villages we can't help it. I know it is illegal and if we don't do this then the people of our village will be in trouble! If we had qualified doctors here for 24 hours then we wouldn't have come in this line! Just to help our

neighbours and family we have to do this despite knowing our job is very illegal.
B: Well he's very aware of that, what they are doing is not kind of legal, for their villagers sake they do. If they do not do this, they don't persue this career, the family, friends and neighbourhood and most of the villagers will be in much more trouble, because there is no qualified doctor here for 24x7. That's why they know that!
D: Yeah! That's right, it's understandable. So when you prescribe antibiotics at a given dose are you aware if people complete the course?
B: When..
J: well when we see someone is having cough, pharyngitis, laryngitis or bronchitis and prescribe medicines, we tell them to have the medicines for 5 to 7 days. Now there are many people who after having for 3 days feel better and never continue, in that case we don't have any role to play, after 1 week the patient comes and confirms, he is fine. And when I ask, "did you have the medicine for 7 days?" they reply, no I had it for 3 days, I feel fit and so I didn't continue. Now every time I make them understand that if these antibotics would develope resistance and the consequences are severe, if this would pass on to your child the medicine will not work on him. When a bacteria gets the resistance then there will be a huge problem. This will affect the next generation, which is why completion of the course is necessary! Now , they don't have money, they don't have money to have it for 7 days, if after having medicine for 3 days they feel better then why will they go for the 4th day? This is the problem.
B: Suppose a patient comes with problem like meningitis, laryngitis or bronchitis and sir prescribes him an antibiotic and instruct them to continue for 7 days. The patient just stop consuming after 3 days. Because they feel good. And when they come he asks whether you completed the course or not, so they answer no, after 3 days I feel good so why will I consume it? So he has to every time make them understand that if you don't complete it then the bacteria will grow resistance against the antibiotic and it will harm your next generation or the people around you. So specially he/they need to make them understand each and every time about completing the course properly. Stopping after 3 days is dangerous and second is people come with a capacity to buy an antibiotic for 3 days and can't buy it for 7 days. So they discontinue after feeling better.
D: That's pretty interesting.
J: There's obviously a lack of awareness. They say if I am cured after having a medicine on the 3rd day then why will I continue for 7 days, it's actually the doctors who want to extort money from us and asking us to continue! We are trying to spread awareness, because for this we may also get suffered! The children of my house will also suffer!
B: The matter is if someone's coming with the mentality...after stopping the med after 3 days, they are coming and saying we won't spend any more money on this, he things there's a lack of awareness, about consumption and completing the course of AB, first and second is if people are coming around and says I am feeling healthy why will I extend to next 4 days, there must be a doctor who wants more money out of me! So they try to understand them that, that is not the thing you're generally talking about. The patient thinks about the money that the doctor wants to get from them. That's why they ask them to continue.
D: Do you explain this when you first give the antibiotics?
B: Do you say all these when you first give the antibiotics?
J: Yes, I tell them on the first day. This particular med you have to have for 5 days or 7 days. You

must have it, you will feel better after 3days, If you feel better don't discontinue, complete your 7 days course. They just say yes. We also say, if you don't continue then this med will not work for you later! If the bacteria gets transferred to another person from you and that particular person comes to me, the med will not work in his case too. Even it comes to me, then it won't work for me too. I won't know that the resistant bacteria has gone from you to him. I give him the same AB, it didn't work for him and things would get serious! I explain these on first day only, else why will they have for 7 days? I have to explain them the consequences!
B: (He explained the same thing but with a lot of wrong information)
D: And have you noticed that the AB don't worked on people?
B: Have you ever seen cases where AB didn't work?
J: Yes I have, Many times I have seen the AB is not responding. So if it's not working with the meds we gave then we send them somewhere else, where they get latest AB and it worked!
B: He says when AB doesn't work he just refers them to better places where because of better facility, they try to prescribe latest or new AB and that actually works.
D: And how do you assess whether or not treatments failed.
B: What?
D: How do you assess that whether or not the ABs are not working.
B: How do you understand that ABs are not working?
J: After giving an AB, the patient is not responding after 3/4 days, his condition is becoming worse, then we figure out that the AB is not working properly. Because in the past we have given the same med and it fairly worked on the 2/3rd day but this time even after 4/5th day it's not working then we can conclude it. May be resistance! Then we refer the patient.
B: (Explained the same thing, but with complicated sentences)
D: So now I will ask few questions that I asked you yesterday. So sorry for repeating myself.
B: We will repeat the same questions, the last day questions!
D: Do people come to you regarding the health of the animals?
B: Do people come to you regarding the health of the animals?
J: No. Yes! they do come, but as I don't have much knowledge on this, and have experience in nursing. I don't have experience in animal health, I ask them to go to the Panchayat, there are doctors, they can treat you, we can't!
B: (Explained the same with NO mistakes)
D: Is there any situation where you prescribed an AB for animal use?
B: Has this ever happened that you prescribed ABs for animals?
J: No! Since I don't have any idea about animals, their diseases and symptoms also no idea about their dose, so I never do these things!
B: (Explained the same)
D: So what do you see the difference in human and animal AB?
B: What do you think the ABs for humans and cows and goats have any differences?
J: Now, I don't have idea about animals, if I had any idea I would have understood the differences. I have idea about humans, as I spend 10 years of my life at nursing homes with doctors! I can't say the difference!
B: Just because he has no idea about livestock he can't say the difference in human and animal AB. Just because he is a practitioner of human health, that's why he can't tell the difference!
D: Okay, is there any situation where you refused to prescribe AB?

B: Was there any situation where you refused to give AB?
J: Yes, it happened! It happened many times. Once a patient came fever with convulsion, so not only I refused but escorted him to the hospital and admitted him. I thought he might have meningitis! He actually had that, so in such cases I never gave AB.
B: Well he faced such situations where a patient arrived here with fever and convulsions. He just refused to treat him and took him to the hospital. He suspected the patient is having meningitis afterwards the senior doctors said, it was meningitis! In that case he didn't treat and refused to give AB.
J: In case of tuberculosis, I don't give AB. If a TB patient comes with fever, we see the x-ray reports, we do the pathological test and found him to have TB, in that case we refer to the hospital we don't give AB.
D: Okay, and why not?
B: Why not?
J: I have less idea about the antibiotics for Tuberculosis. I didn't see treatment of TB patients in the nursing home I got my training from. And these have long term treatments, the govt has specific measures to treat these. If we get to see such disease we simply refer! We only treat for fever, cough and cold, diarrhoea. We do work with few rural diseases! No more than these, we just refer those cases!
B: 1st of all he doesn't have any idea of TB patient, because where he got the training, he hasn't seen such TB patients. And secondly TB treatment is a long term treatment, so there is a facility our Govt provides for these kind of treatments. So they are sent to hospitals where they can be treated properly!
D: Ok.
J: If there's any awareness program regarding AB in villages I will love to get associated with such programs. I love to do such social work. Also on hygeine, awareness on this subject is very important in villages! These awareness is something we can't do from here, we can tell the patients but it's not always effective. A door-to-door program is necessary and will be beneficial to the villages. I want to get associated to these events, I will be very happy!
B: (Explained the same)
D: Alright, that's great, tell him I will convey this to my supervisors! 1 question that I missed, you said that people come to you for the advices on animals, why do you think this is?
B: Why do you think people come to you to take advice on animals?
J: They think, a doctor means he is doctor to both humans and animals. At least he will give some medicines. They think the meds for both the humans and animals are same. They think the medicines they have when they are ill are the same ones when their cows and goats will be sick. The med I take when I have diarrhoea the same med will work when my cow will have diarrhoea! They have such conception. But we don't understand when they come to us with animal problems!
B: (he explained the same)
D: Okay thank you very much!
