# Supplementary material for: “If It Works in People, Why Not Animals?”: A Qualitative Investigation of Antibiotic Use in Smallholder Livestock Settings in Rural West Bengal, India
Source: Antibiotics (Basel). 2021 Nov 23;10(12):1433. doi: 10.3390/antibiotics10121433 (PMC8698124; doi:10.3390/antibiotics10121433)
Supplement: Supplementary file 1 [file antibiotics-10-01433-s001.zip › Supplementary S1_ Interview Transcripts/Site 1/Informal Provider 3 (site 1).pdf]

Code for Study - 'If it works in people, why not animals?': A qualitative investigation of antibiotic use in smallholder livestock settings in rural West Bengal, India: IP3, Site 1

Date: 19/07/2019

Location: Site 1

Interviewee: Informal Provider of Human Health (IP)- antibiotic provider

Interviewer: Dominic Day (DD)

Translation: Somraj Das (SD)

Transcription: Sayak Manna (SM)

D: Interviewer (DD)

B: Translator (SD)

DM: Interviewee (IP3)

START OF INTERVIEW

D: Thank you very much for answering our question.
B: Thank you for answering and giving us time!
D: Could he explain what his role is?
B: What's your role here?
DM: I am rural practitioner, I serve the villagers. Primary treatments only. Like First Aid treatment. Then we refer to the hospitals. If some people come with fever or diarrhoea I give the primary treatment, give metrozil or ORS, ask them to have loads of water. If the person is having severe vomitting, I prescribe some meds to stop the vomit and diarrhoea, if the running stool doesn't receed then I refer to hospital. We have to give service 24x7! I offer day and night service, if I am called over phone I go to the house. My practice extends all the way to [names of villages outside of site 1 redacted], at least 3/4 km away.
B: He's rural practitioner, he generally treats the first stage disease. First Aid things.
D: First what?
B: First Aid things. If someone is coming with a disease and if it's controllable then he can deal with that. But if the situation is critical then he refers to GP- Health Dept. And he does it 24x7. And he does it far away, [names of villages outside of site 1 redacted]. He even does it at the middle of the night
D: And who does he normally serve?
B: Whom you treat?
DM: I treat the villagers, the poor villagers, the rich goes to the hospital, I treat the needy people who come with little money, like a10/20 rupees and can't afford to go to big doctors!

We even treat them on credits! We can't help, we have to serve day and night if people are sick. They ask us to treat on credits and like this from the people I am supposed to get arounf 1-1.5 Lakhs. They sometimes can't afford also people are not having the humane nature anymore. They don't want to give the money. For years they get the treatment and never pay. We even have to make credit books to track the people. On the basis of the cards which are given out, we estimated that out of 600 only 200 people paid and the rest didn't. The ones who come they barely pay. Of course we get annoyed towards the behaviour of the people around but again I console myself by saying, "They are poor and we need to serve them", I am serving for 35 years, so love and compassion is making me move ahead in life. People say "you are God", I tell them don't say like that, this is my duty. You call us doctors, though we don't have the certificates but we need to serve you for life. Who can give will give others will not. We just strike out the names from the credit book who never gives for more than 2/3 years. Sometimes people can pay but they usually don't. I do it for a better afterlife, I feel good when they see me helping them. I give med for stomach pain or gas and when they are cured they are so glad, they give so much of love and this is such a blessing. That's why I whole heartedly work, other doctors in this area is not much bothered but I religiously help people whenever they need me. If you ask people around and enquire you will see how much people appreciates my selfless dedication. Sometimes whole night I stay awake. This makes me happy!
B: (He explained in his way). People have money but they don't have the tendency to pay him because of which this man is at a loss of 100000 rupees per year. (explained)
D: Is this the reason why people come to him?
B: Yes!
D: Is he certain?
B: Are these the reasons why people come to you?
DM: Yes.
D: Is it normally the same people come back to him again and again or is it different people.
B: Do same patients visit you or new pwople also come?
DM: New people come too. And old patients come too. But people within 2/3 kms come to me not a lot of people. This village and the adjacent ones. (He took names of some villages as mentioned before). I usually go when I am called. There was another doctor here like me but he expired. His name was *Name redacted*, now I have replaced him. I'm 35 years working now and my age is 59.
B: (explained right)
D: But he doesn't practice any more?
B: He expired. That's why he is doing the same thing. He's in the profession for last 30 years and his age is 59.
D: Ok and how come..why did he choose to do this job?
B: Role or career?
D: career.
B: Ok, sir why did you select this profession to treat 30 years back?
DM: *Life history redacted*
D: *smirks*
B: (he explained in brief)
D: Great, thank you, can you tell him I would like to talk to him about his medicines now?

B:Now he wants to ask you some technical questions regarding your medicines.
D: Could you describe what AB you keep in stocks?
B: Which types of AB you keep?
DM: I seldom stock AB, almost never in use. I only give a priliminary treatment. Fever, diarrhoea, first aid etc. Though Amoxicillin is used sometimes but AB is not much in use. So with little treatments things get subsided. Also patients don't stick around for long, you understand what I mean? If I se little bit of problem or the situation is crtical, I immediately refer the patient! Hence people love me a lot, because I don't ask them to come for a long time. I just give meds for 2-3 days, they come with very less money so I can only give meds for few days only. If it doesn't work then I refer them to hospital under the supervision of experienced doctors. Patients are refered to Medicine specialist, child specialist and others. So not much AB is used.
B: What he's saying right now that he barely stock AB, he seldom use them because he practices with First-Aids. If he sees that the situation is getting out of his hands, he refers to a bigger qualified doctor. So generally he doesn't store the AB you are talking about.
D: Sure...ummm...can he explain why he does stock the ones he does?
B: The things that you have, what are those? If you have AB what are the types?
DM: Hmm.. Amoxicillin. For chickens and ducks Bactrin DS etc are used. (very apprehensively answered)
B: He talked about Amoxicillin and (..?) for chicken and livestock.
D: He use for chicken and livestock? So does hekeep both livestock and human Antibiotics?
B: Do you keep both human and livestock AB?
DM: Kind of yes! Sometimes it is needed to be kept. When the meds for cows and goats are not available around, I stock them here.
B: Well he does stock for both
D: Can he tell me what the common problem is that he prescribes AB for people?
B: For which problems you give AB?
DM: When children..umm..some grown up children (seems like he was hiding something, hence used grown up) have cold and cough or fever then.. most of the times it's cold and cough. And for chickens, when they have runny stools, I give metrozils and bactrin.
B: He's talking about (..?)
D: And how does he choose which AB to give?
B: How do you select which AB to give?
DM: I don't give AB much.
B: Whatever you give, give a general answer?
DM: If 1st time after giving the meds it's seen nothing is working then I give a little. If there's too much of cold and cough AB is given and if the sickness doesn't cure then i refer to specialist and hospital.
B: He's saying he doesn't prescribe AB but in certain cases when there is exces cough and cold, he prescribes Ab and after that (..?)
D: Can he tell me which species he most commonly give AB to?
B: Livestock?
D: Yeah.
B: Which type of AB you give to livestock?

DM: The same one AB only.
B: Name?
DM: Bactrin DS. The veterinary doctor of animal hospital often writes this name, I have seen it. He writes that and so..
B: Sir is saying there's a stuff called Bactrin as an AB, he saw a genuine health practitioner in the GP health Department has written in prescription, he prescribed it, that's all.
D: Ok, And what species of animals he most commonly give AB to?
B: To which animals do you prescribe AB mostly?
DM: We don't get animals much, not much at all. Here we have animal doctors, they come to us a lot. They go to animal doctors, sometimes they come to us, they rarely come when they can't get hold of meds. Maybe there is diarrhoea, then we give metrozil anti-diarrhoea meds. Otherwise not much. The animal doctor sits at [name of hospital redacted] hospital. Goat, cow, chicken doctors are there, they give free of cost med.
B: (Explained moderately)
D: So why do people come to you sometimes?
B: When they come to you, what do you think, why do they come to you?
DM: When they don't get other doctors, then they approach me. They come mostly at night. Also here money is less, the experienced ones will charge much more. The villagers are poor and in less cost they will get the First Aid.
B: Sir is saying about he availability, as I told you before that one thing. And at night, they call him, Also experienced practitioners are expensive. he charges less than other practitioners who are holding a degree.
D: Ok and does he normally administer the AB himself ?
B: Do you tell them how to give the Ab/med?
DM: Yes I tell them to which doctor to go, depending on the patients I refer them to different specialists.
B: He said, he generally asked the patient to go to that doctor to get the medicine. Basically he doesn't administer.
D: Ok, does he instruct them how much and how long to go?
B: Do you tell them what meds to take and how to have?
DM: The patients after visiting the doctors come to me with the prescriptions, so I tell them about the meds as per the prescription, most are poor uneducated people, so I have to tell them in bengali as per the prescription reads. How to have the meds etc.
B: (Explained right)
D: Ok, so do people come to him for information about their livestock generally?
B: Do the livestock owners come to you for advice?
DM: No, they don't come to me but to the animal doctors.
B: No, they go to GP.
D: Do they prescribe drugs for both humans and animals?
B: Do you prescribe any med that works both in case of humans and animals?
DM: Yes, the same meds work. The meds for humans work in animals too. By decreasing the dose it works.
B: Sir is saying that yes sometimes, meds for humans quite effectively work in the livestocks as well, it's the doses that needs to be taken care of, otherwise human meds generally work in

livestock.
D: Okay, could he tell me which AB he uses for both?
B: Which type of meds works for both?
DM: White stool, when they have white stool..Doctrin, metrozil, phoraxonanil work both for humans and animals. And also I have seen maxozol..
B: (xplained right)
DM: Digestive vitamins also work.
D: Does he give livestock meds to people?
B: Have you ever done this thing where you prescribed a med for livestock but it is meant for humans?
DM: Yes those metrozil and phoraxonanil, digestive vitamins work both in case of humans and animals. It was given and it was seen they worked.
B: (Explained right)
D: And what does he see to be the difference between human and animal AB?
B: What difference do you see between the meds of humans and animals?
DM: There's no such difference I've seen, I don't give a lot. Sometimes when they don't get meds then approach me. Doctors said they don't have so we came to you for the meds. And they worked. The ones who keep birds, they said it worked. Oh I forgot to mention there's someone here who farms birds, beautiful, expensive birds, I will take you to them. He comes and tell that the meds worked! When there's watery stool he comes and takes the meds.
B: (explained right)
D: How does he calculate the different dose for..
B: How do you calculate the dosages in case of humans and animals?
DM: The owners do it, you get me? The ones who owns, they know everything, they calculate the dose, they take the meds from me and give them. They say the doctors give us, we know it how to give.
B: sir is talking about bird keepers and they have the experience and experties. They usually decide the doses. So they are expert, they consult with him once and then they decide the dose.
D: So they seek advice from him everytime he prescribes meds?
B: So whenever they come to you for meds with prescription do they always take advice from you?
DM: No, they don't take advice, they are much more experienced than us. They are keeping animals for many years, they discuss with the doctors and give. They know about animals much better than us.
B: (Explained same)
D: Is there any situations where he wouldn't give AB?
B: Has it ever happened that you didn't have to give AB or have to give AB? (Question changed!)
DM: I don't have to give much AB. We barely give AB.
B: He didn't ever prescribed AB, or barely given.
D: But has he ever refused?
B: Did you ever refuse or not prescribed? AB?
DM: Yes
D: Could you describe the situation?

B: What happened exactly? Why did you refuse?
DM: AB.. not exactly refused, actually small doctors like us never prescribe AB, we don't, we just give the normal meds. We had a training in [name of NGO redacted], and from there we were asked not to give AB much.We were trained to avoid. We don't give, we try as much as not the give AB.
B: They do not like to prescribe B, even [name of NGO redacted] adviced them to ignore AB when you are practicing. Generally better for him according to [name of NGO redacted].
D: And why does he think this is?
B: Why do you think they tell it this way?
DM: AB has many reactions, we don't have much experiences and so we don't take that risk. So we avoid AB, if AB is needed to be given, we refer to the experienced doctors. *Name redacted* explained us and trained us. I took a 1 year training in LF.
B: (Explained it in right way but added unnecessary spices like AB can be fatal!))
D: Ok, Does he tell people when he gives them AB whether or not it should be only used in humans or in animals?
B: Do you tell them that use it on humans or use it on animals?
DM: No..we don't give for animals, we don't treat animals. Humans have cold cough fever and not getting cured then we refer.
B: Sir is saying he doesn't practice in livestock, but he is talking about if the situation about human arise, that is too much of cold cough then he prescribes some AB, otherwise he doesn't practice AB specially in livestock.
D: Ok, Final question about AB, does he ever prescribe them for a (..?)
B: When you gave AB, other than cold and fever, did you prescribe for any other disease?
DM: No.
D: Could he explain his medical training?
B: How was your Medical training? Where you got it from, how it was done?
DM: Since childhood, I did many courses on paramedical, all the short courses, alternative medicine like CMS, EDP, PET, FWT etc and short course. After coming to the medical field I did many small courses from private places. The experienced doctors gave us the medical training. Got many certificates.
B: (Explained moderately)
D: Has he done any vetenary training?
B: Any training on livestock?
DM: Once I did take, near [town name outside of site 1 redacted], they gave a certificate too, but never did any treatment.
B: He got a training many years ago but never did vetenary practice.
D: And does he get most of his information from now?
B: What kind of information?
D: Professional information.
B: Where do you get these medical informations from?
DM: From many places, at [institute name redacted]. I used to get my training from there, so they gave us informations about other trainings. Then from [name of rural practitioners committee redacted] too. I was the president of [town name outside site 1 redacted] subdivision committee and now I'm the president of [town name outside site 1 redacted] Rural

Practitioners committee.Through these I got many trainings too, different doctors gave us trainings.
B: (explained)
D: and do these organisations have guidelines on AB usage?
B: Do these organisations have guidelines on how to use AB?
DM: Yes, in the training only they tell us not to use AB, we will refer patients when AB will be required. Treatments are to be done without AB.
B: (explained)
D: Can he show us some of the meds he use for both?
B: Sir questions are over can you show us the meds?
Dm: Yes.
D: Thank you.
