# Supplementary material for: “If It Works in People, Why Not Animals?”: A Qualitative Investigation of Antibiotic Use in Smallholder Livestock Settings in Rural West Bengal, India
Source: Antibiotics (Basel). 2021 Nov 23;10(12):1433. doi: 10.3390/antibiotics10121433 (PMC8698124; doi:10.3390/antibiotics10121433)
Supplement: Supplementary file 1 [file antibiotics-10-01433-s001.zip › Supplementary S1_ Interview Transcripts/Site 1/Informal Provider 4 (site 1).pdf]

Code for Study - ‘If it works in people, why not animals?’: A qualitative investigation of antibiotic use in smallholder livestock settings in rural West Bengal, India: IP4, Site 1

Date: 22/07/2019

Location: Site 1

Interviewee: Informal Provider of Human Health (IP)

Interviewer: Dominic Day (DD)

Translation: Somraj Das (SD)

Transcription: Sayak Manna (SM)

D: Interviewer (DD)

B: Translator (SD)

R: Interviewee (IP4)

START OF INTERVIEW

D: Thank you very much for answering our questions.
B: Thank you very much for agreeing to answer us!
D: Umm.. could you describe what’s your role is and (..?)
B: Can you specify what your role in this area is?
R: Rural doctor. As a rural doctor. RNP doctor. Doctor…eh..mm..as a practitioner.
B: He’s basically a rural treater..rural practitioner. That’s it.
D: Okay! And who does he normally serve?
B: Whom do you serve?
R: Poor village people. Who have no money…have…very much no money. Poor People. We are treated as we can. We treat as much as we can.
B: Practically the poor people, they generally serve in this community.
D: Who so?
B: Poor people, financially weaker.
D: OK and why..why does he think it is these people that come to him? Why does he think that these people come to him?
B: What do you think, why do they come to you to get treated?
R: To get Better treatment in less fees.
B: Well it’s about money, they charge less and provide better service.
D: Okay, is there any other reason?
B: Any other reason?
R: No, this is the reason.
B: This is the reason.
D: This is the reason, okay and does he normally serve people.. does he serve people outside the village?
B: Do you treat or serve outside the village?

R: Actually I'm physiotherapist, who has physical problem, I need to help..as..family call me, home service as..is home service. (He meant he's a physiotherapist and when people have some physical problems, he gets calls from the patient's family and he goes to treat them at home, more like home service)
B: He's basically a physiotherapist, if someone calling him for home service..he goes there and treat him.
R: Who's patient has paralysis, spondylosis, osteoarthritis, he don't need to walk (he meant can't walk), so I have to go.
B: Just because..if the patient has paralysis, arthritis and some kind of disease, they cannot come here, so that's why they call him and he goes there. (..?)
D: Ummh..and how long he's been doing this role?
B: How long you are doing this work?
R: 4 years.
R's father: Since 1986 till present day
R: He's my dad.
B: Interviewee is saying it's about 4 years.
D: 4 years. Great. And do they do before? Do before they did this?
B: So you didn't do this before 4 years?
R: I'm in this practice for 4 years. I'm being a physiotherapist for 4 years.
B: He is in physiotherapy for last 4 years. Before that he was in this practice as well. As physiotherapist he is been more than 4 years.
D: Ok and what was his role before he was a physiotherapist?
B: What you used to do before you became a physiotherapist?
R: When I was a student, from 2011, I was in the practice with (as) my dad's assistant. Then I completed physiotherapy course, 4 years and (..?) practice.
B: Basically he is saying that, before he was a professional physiotherapist he was a helping hand of his dad, as an assistant compounder something. That was his role before becoming a physiotherapist.
D: Ok, and what made him decide to take his career path?
B: Why did you think of going to physiotherapy?
R: Actually, the people of villages are poor. They had to travel to Kolkata to get the physiotherapy done. The visit would cost them 300 or 350 or 400 rupees. Now in the village if I can give them the same treatment in 100 or 150 rupees then the poor people get some help. To help the poor people.
B: Actually he is saying that, around physiotherapy..around the year patient..the patient around the year go to Calcutta for the service..in the physiotherapy treatment which costed more than 400..350, so he chose it as a career because he wanted to serve poor people here, by charging less, around 100/150. That's why he took this path.
D: Ok, that's great! Umm..can you ask him what he understands by the term, antibiotic?
B: what do you understand by antibiotic?
R: There are antibiotic like Neosporin, cephalosporin, azithromycin which are known to us. These are the ones that we normally give.
Someone: (..?) higher antibiotic for not all (..?) lower antibiotic. Higher antibiotic like (couldn't get the names), because of the cost they can't buy these higher antibiotics. We have antibiotics which are less expensive, which is why in less costs, like 100 rupees we can give the treatment.
B: He gave the main interview. He named some antibiotics name. And he actually practiced..prescribed in his current carrier, that's why he knows about antibiotics.
D: Ok..umm..and what does he tend to use them for? Why he commonly use antibiotics for?
B: What are the reasons you commonly use antibiotics for?

Someone: Amoxicillin, potassium carbonate, (..?), potassium carbonate, combined medicine..(..?) and typhoid, influenza we use for these diseases.
R: Acute disease, acute disease recover we throw antibiotics (He meant to recover people from acute diseases we give antibiotics).
Someone: Amoxicillin 500 strength.
D: Acute
B: Acquid!
R: If the patient suffers acquid disease, he says..if the patient suffers acquid disease that's why we throw antibiotics.
B: In case of acute diseases he commonly use antibiotics.
D: Ok..umm.. Could you just remind them that it got to be just [interviewee name redacted] and not..
B: Sir your answer is important to get recorded. He may say, we will listen too, but your one is important and needs to be recorded because you are an interviewee. I will add his words separately in the context but your one is important.
D: Could you explain what he means by acute diseases?
B: What do you mean by acute disease?
R: Like diarrhoea, fever, influenza, typhoid, bronchitis, asthma.
B: He's talking about some disease thyroid, pneumonia, diarrhoea, bronchitis, he thinks these are acute diseases.
D: And could he explain what antibiotics does he stalks here?
B: What are the antibiotics you stock here?
R: Azithromycin, amoxicillin, (..?), Potassium carbonate, (..?) these normally.. ornidazole.
B: Amoxicillin and some kind of..
D: Yeah I got it, thanks. And does he provide antibiotics to livestocks?
B: Do you provide antibiotics for the animals?
R: NO, not for animals.
B: Sir, he is saying no, they do not!
D: So do people come to them for medical advice?
B: So people do come to you for medical advice? (He should have asked, whether people come to you for medical advice)
R: Yes
B: Yes
D: What sort of advice?
B: What kind of advice?
R: We treat which is within our abilities, but there are things which are not in our hands like operation, in that case we refer them to the hospitals.
B: Sir, he's saying, disease which they can treat or they can confine they usually treat. But if there's a case arise where disease that can't be cured by them, they refer to the hospital (..?)
D: Umm.ok..And where they normally, where they get their drugs from?
B: where do you get these drugs from?
R: Our representative come to give us. He shows a catalogue that we have these antibiotics in our stocks and take what we need.
B: Sir, he's saying that there are kind of medical representatives, they come with some catalogues showing them and they select what kind of drugs or antibiotics they store or buy (..?)
D: And how do select what drugs they are going to stock?
B: What kind of thing, they made decision for?
D: Yeah, how do they decide which antibiotics they like to stock?

B: How do you particularly decide that these are the antibiotics you need to stock?
R: These antibiotics are the basic ones and needs primarily. If there is fever or cold, we usually give azithromycin amoxicillin, because they are cheap. For high antibiotics the poor villagers can afford much. It's out of their ability or reach, they can't bear the cost! So we keep these.
B: So he is saying that, there are some amoxicillin (..?) which are antibiotics they usually store as antibiotics, and these are less cheaper (he meant more cheap) and more effective for poor people around this village. That's why they try to decide to keep these types of drugs in store every time.
D: Ok..umm..and do they normally administer the antibiotics themselves?
B: Do you people yourselves administer and manage these antibiotics? (I think he got the question wrong), I mean administer.
R: First we give simple medicine and if the things don't subside we give antibiotics. We don't give antibiotics in the first attempt. If there's fever we give simple paracetamol for two days, if the temperature persists then we give antibiotics.
B: Sir, he is saying that the very first moment they do not prescribe antibiotic, they usually give paracetamols or calpols type of things, afterwards they see and look around the symptoms and afterwards they decide whether they will prescribe antibiotic or not.
D: And at what point do they decide that a patient needs antibiotics?
B: Exactly at point do you understand that a patient needs antibiotic?
R: Suppose someone has fever and it is not subsiding, he's having medicine but the high temperature is increasing after intervals. If they report us after 2 days that, "Doctor the temperature is fluctuating but the fever is not getting cured completely" or someone is having cold, I gave theophylline (..?) for coughs and after taking the medicine if they complain that the coughing is still persisting, then we prescribe antibiotics.
B: Sir, he's saying suppose a patient is coming with fever, they prescribe calpol or paracetamol and the temperature fluctuates and the patient complains we have a fluctuation in fever everyday, then at that point, they decide to prescribe antibiotics.
D: Ok, could he explain what advice he gives at the time of prescribing antibiotics?
B: When you prescribe antibiotics then what advice do you give to the patients?
R: Have the medicine for 2/3 days and if you see it's still not getting cured then either go for blood test or go to a better experienced doctor or hospital.
B: Sir, he is saying that..
R: If they have to go to a nursing home then it becomes very expensive for the poor patients of the village, you know how poor they are, they bring only 100/150 for the checkup, so getting a service at nursing home is difficult. Public Hospitals are better, they get better treatment at free of cost!
B: Sir he's saying that after prescribing 2/3 times of antibiotics, if it's not working with the patient, then they advice the patient to have pathological test and afterwards go to a bigger facility. It is the main thing he's talking about.
D: Ok, and does he explain how much and how long to take the drugs for?
B: Generally how much and for how long do you ask them to have?
R: 3 days. Azithromycin for 3 days, per day OD dose. 3 medicine for 3 days. Or Amoxicillin TDS dose, 3 medicines for 3 days. If the thing is not getting cured we ask them to go to a better place.
B: He's saying about the amoxicillin. Suppose amoxicillin is prescribed for OD dose, which means 3 days each. Another kind (..?) dose which they prescribe for 3 days each, and after that if they sya there's no recovery at all, at that point they try to refer to patient to a bigger facility.
D: Ok..and does he explain how antibiotics work to the patient?
B: Can you tell how exactly antibiotics work on the patients? (He changed the question completely)
R: To some extent I can… umm.. antibiotics strengthen the antibodies in our body. The bacteria in our body is destroyed by the antibiotics. That's how it works.

B: Sir, he's saying moderately, it depends on the dosage and all, if he's prescribing something, it depends on the dosage. It works moderately according to his knowledge. ([interviewee name redacted] didn't say this)
D: Ok great..umm.. so for what reasons he thinks that sometimes it doesn't work?
B: Antibiotics?
B: Sir what do you think, why antibiotics sometimes doesn't work? What can be the reasons?
R: May be the disease has aggravated, for a long time they haven't taken the medicine and that increased the disease in them, for them antibiotics might not work.
Someone: When antibiotics are taken randomly sometimes they don't work. May be someone has bought antibiotics and had them when it wasn't required, which isn't right.
R: Yes, they might have had antibiotics, or they might have visited some other doctors who gave them antibiotics.
Someone: If someone is having viral problems and had antibiotics then the antibiotics will not work in that case. They need to understand antibiotics will not work on virus. By randomly buying and having antibiotics, he body might lost the ability to defend and now antibiotics are not working. In that case we have to give high dose antibiotics which are recommended and advised by World Health Organization. 3rd generation of Antibiotic!
D: what did he say?
B: Sir, he is saying that there are many things. Supposedly there is a chronic disease and he is using antibiotic for that chronic disease for a long time and plenty of the day (he meant many times in a day) there's a immunity system that has made antibody against the antibiotics, then again you have to go for higher doses of antibiotics that can usually be affective inside the body for the disease, especially about the viral flue, the main interviewee said, at this case antibiotics might not be working.
D: Ok, I want to ask him, is there any situation in which people come to him for advice regarding the livestock?
B: Umm..is there any cases where people visited you to get some advice for their livestock?
R: Yes, they do come, 1 or 2 of them do come, very rarely. "Doctor my goat is having loose motion for 3 days and stuff", so there we have our panchayat's or Government's animal hospital, we ask them to go there. If someone asks for loose motion medicine in that case I give norfloxin ornidazol, they have twice in a day and after half an hour the loose motion subsides.
B: Sir, he's saying yeah but rarely. Suppose, for an instance, say if someone is coming with the problem about the livestock which is..suppose a goat is having dysentery then he come here looking for an advice and he ([interviewee name redacted]) prescribes an anti-dysentery medicine with a mixture of water, it usually works, but rarely people come here for livestock advices.
D: Ok, other than amoxicillin is there any other drug that he might, prescribed to livestock?
B: Other than amoxicillin do you prescribe any other medication to livestock?
R: No, they come very rarely.
B: No, rarely they arrive here for the advice for their livestocks but except from amoxicillin they don't use or prescribe or practice any other medication on livestock.
D: And is the amoxicillin they give meant for humans?
B: Amoxicillin that you give is it for human?
R: It's for human but I may give 1 or 2 to the goats, actually the problem is nobody goes for full treatment. These are usually for humans
B: Sir he's saying amoxicillin that he prescribes only for humans, people here don't get the total course completed about the medication but when they come here they usually use amoxicillin as an antibiotic for humans.
D: Okay and what does he think people do with antibiotics which they don't use when they don't

complete the course?
B: So when human don’t complete their course or don’t continue, in that case what do you do or what do they do?
R: They have it for 3 or 4 days, when they think the disease got subsided then they don’t come here, that’s why we give azithromycin, a three days course because it gets completed in 3 days time. The expense is less. 3 tablets will cost 60-65 rupees. So we prescribe azithromycin to those who actually need it.
B: Sir he’s saying if people don’t continue the medicinal course, they usually come here and they give 3 days course of amoxicillin as an antibiotic. It’s cheaper and effective and 3 days course. So people come here and they practice amoxicillin for 3 days course.
D: Okay. And do people normally complete that course?
B: Do they complete this course?
R: Yes they mostly do, they bring 100 rupees and azithromycin is within that amount. They come with 100-150 rupees and requests to give medication within the amount.
B: Yes, they usually complete this course actually! If you have 100 rupees in your hand you can complete this course (..?)
D: Okay! So what does he see the difference between human and animal antibiotics?
B: What difference can you see between a human and an animal antibiotics?
R: We don’t treat animals, now when someone comes saying, “oh my animal has caught cold” then I give 1 or 2 of these to them. We don’t treat animals as such. We do have animal doctors visiting at the panchayat’s hospital.
B: sir, he is saying, they barely practice or barely prescribe for livestock…generally . barely…if someone is coming to them for some advice for the livestock, they prescribe general medicine, otherwise there is a general practitioner who is a vet, they barely come here. They go to them.
D: Ok, And what does he meant by general medicines?
B: What do you mean by general medicine?
R: General medicine means Zintac, Rantac etc. Medicines for pain relief, paracetamol, for stomach pain spasmodon, metrodinazol.. some of these and more.
B: He is saying the general medicine he meant are rantac, paracetamols, and some sorts of medicines like that, I do’t know the names actually.
D: Ok, and what are the names of the vets that other people normally go to?
B: What’s the name of the doctor that people go to at the animal hospital?
R: Name is not known to me. Do you know the name?
Someone: Whose?
R: Do you know the name of the vet? (to someone) (..?)
R: We don’t know the name.
B: They do not know the name.
D: Could they tell us where he is?
B: *Name redacted (CAHW1 Noorpur GP)* is his name. Where does he generally stay?
Someone: At his home.
(Some confusion which I couldn’t follow in the audio)
B: Where does he stay?
R: I can’t say.
B: Is there any phone number?
Someone: Of *Name redacted (CAHW1 Noorpur GP)*? He stays at homeand in the morning he visits villages to treat animals. He has his home at [village name redacted] Bhawanipore.
B: In this village?
R: Yes in this village

B: How far?
R: You have to go a little inside
(Some irrelevant conversations)
D: Could you tell me a situation where you might refuse to give antibiotics?
B: Has this ever happened where you refused to give antibiotics?
R: Yes, it has happened.
B: He says yes, there was a situation where he refused it.
D: Could he describe the situation?
B: Can you describe the situation?
R: What happened was, for 3 or 4 days that person might or might not had any medication, can't be sure about. He got a very bad cold that literally turned out to be like pneumonia, he had congestion in his chest and something like that. In that case anti (..?) 650 was given, it never worked. Then he might have taken to the hospital which I can't confirm. He never came back. He complained to me what medications I prescribed that it didn't work well, so I referred to the hospital. So there wasn't any news after that.
B: Sir, he is saying, there was a situation where a guy/patient with a high level of pneumonia symptoms, he applied one and that didn't work and afterwards sir referred him to a better hospital or better facility..afterwards.
D: Ok and why does he think that it didn't work?
B: Why do you think the medication didn't work?
R: May be he had some antibiotics that developed a resistance. He might had 1 or 2. You have to complete the antibiotic course right? If he didn't finish his course then there are chances that he might develop a resistance!
B: Sir he said that he isn't sure but he thinks that the patient might have consumed an antibiotic before so the entire body became resistant to that antibiotic, that can be a possibility, that's why he didn't prescribe antibiotic and send him to a better facility.
D: ok..ummm..and going back to the drugs he gets, they described a catalogue. Does that catalogue contain human and animal drugs?
B: You talked about the catalogue, that the MRs bring to you, do they have both animals and human medicines in them?
R: No, no. Nothing is written like that just composition is there and name of the medicines are there. Only human.
B: Sir he is saying nothing like that in the catalogue, only the compositions and medicine names.
D: Ok, that's great, I think that's everything then. Thank you very much.
B: Thank you sir, for giving us your time.
D: We appreciate!
