# Supplementary material for: “If It Works in People, Why Not Animals?”: A Qualitative Investigation of Antibiotic Use in Smallholder Livestock Settings in Rural West Bengal, India
Source: Antibiotics (Basel). 2021 Nov 23;10(12):1433. doi: 10.3390/antibiotics10121433 (PMC8698124; doi:10.3390/antibiotics10121433)
Supplement: Supplementary file 1 [file antibiotics-10-01433-s001.zip › Supplementary S1_ Interview Transcripts/Site 1/Informal Provider 5 (site 1).pdf]

Code for Study - 'If it works in people, why not animals?': A qualitative investigation of antibiotic use in smallholder livestock settings in rural West Bengal, India: IP 5, Site 1

Date: 27/07/2019

Location: Site 1

Interviewee: Informal Provider of Human Health (IP)- Antibiotic Provider

Interviewer: Dominic Day (DD)

Translation: Somraj Das (SD)

Transcription: Sayak Manna (SM)

D: Interviewer (DD)

B: Translator (SD)

P: Interviewee (IP5)

START OF INTERVIEW

D: Thank you for agreeing to answer our questions.
B: Thank you for answering.
D: First thing I would like to ask is what's your role in the community?
B: Your exact role in the community? In this society, what do you do?
P: Means?
B: Your profession here.
P: My profession is this one only, medical treatment.
B: This is what you do?
P: Yes I don't do anything else.
B: Medical treatment is his role in the community.
D:And who do you normally treat?
B: Who do you normally treat?
P: I treat the children and adults. We are rural practitioners, we are not specialized or qualified ones. More or less we try to do every type of treatment. Whether infant or adult. By every type of treatment I meant gynaecological problems, cough, cold, fever, stomach pain, initially I used to do deliveries too, now I have stopped the work. These types, more or less I know all types of treatments.
B: He generally treats each and every kind of patient from infant to aged. Few years back he used to play a role in pregnancy and labour pain situations but he doesn't do that anymore. Generally he treats from infants to aged.
D: And what area do you cover?

B: (Repeated)
P: Total [name of site 1 GP redacted], almost covering most of the place. Initially I used to sit in other places, so people know me since then, now they even come to me from very far distances. Else the locals come.
B: Generally around [name of site 1 GP redacted] and since he had chambers in past days so there are few familiar patients they usually come here but mostly the people of [village name redacted].
D: And normally you have people come back many times or you treat lots of new patients?
B: Do you meet new patients or there is a repetations of the old ones?
P: By same patients..umm..the villagers come with same cases, ok? We are not some specialists, like gynae or child specialis, nothing like that. We practiced on general problems so we treat the general problems, we treat villagers who come with fever, cold, cough, headache, stomach ache, normal delivery. If we see chronic issues we transfer to the hospitals. Normally I do most of the things like stitching and all.
B: Generally he practice everything like stitch, First Aids etc, because he is not a specialised person like gynaecologist, bronchologist or neurologist, that's why he does each and every kind of general treatments. From stitching, first aid, labour pain and these kind of situations.
D: But does he normally have the same patients coming back many times or does he treat a lot of new patients?
B: Normally same patients come or you do get new patients too?
P: Yes, new patients come too. Not every one is old, may be some people of this area used to go to other doctors but now they come to me. Alternate things happen, my patients will go to someone, others patient will come to me.
B: Sometimes patients who go to another doctor come to him for advices or his own patients go to new doctors, this kind of alternative situation is what he is talking about.
D: Ok, could you describe your career uptil now?
B: How long you are in this profession, what's your history and present senario?
P: It's been 22 years I am here. *Life history redacted.* I am 45 years now, you understand? Now from my age minus 15 years, till my 10th board. So tentatively I am in this line for 30 years. This chamber is for 22 years, initially I used to have chambers in other places. I was in Radhanagar, Diamond, Burul etc. I left those places now, I realised the distance was more, travelling was taking the time and money. Fooding was a problem, I often fell sick. Things were not suiting me. Expenditure was more than the income. So I left those places! I was bachelor then, now I am married. I realised it's better to stay at one place. People here are more, of late the population has increased too. When I started here there were barely any doctors, 5/6 of them maximum, now if you count you will get at least 40 of them. There were no medical stores too, people used to come to us only, we used to provide medicines to them. We were the drug suppliers and we were the doctors, now the scenario has changed. I am talking about 22 years back. Initially we used to treat hence we had medicines, of course we never had permission to keep all sorts of meds and keeping them was difficult too. In those days there were some problems, we had to send them to buy meds from different drug stores or medical representatives used to come and give us some meds. It was the same case for everyone. 22 years back it was the same. Also the rural people are more into bird business, very clearly speaking. So giving out meds for only Rs 200 is nothing. Poor people would come to us, the better offs with go to a

better place. If the charge is 200, the poor villagers will pay either 100 or 50. they will take on credits. Sometimes they never pay their debts. At the end of the year when audit was done, we see that we lost around 30.000 rupees or more! We had to make credit books. It's a matter of shame, doctors shouldn't have credit books. We invited them and feed them sweets once in a year so that they can pay off their debts but alas they just pay 5000 instead of 30,000. So we stopped the credit thing too. The problem with villagers is what you know? Suppose when a prescription is written, you do whatever you can to purchase the meds from a drug store but when I am giving the same facility to the villagers they are paying me full, if it's 500 they pay 200 etc. Some people with conscience will pay you properly but again you do get to see some rogues who won't pay. They go to other doctors. The thing is in village the business is much tough than town. Doing business is difficult here! There are many rogues around, I've seen in my case a patient never paid my due money but went to someone else. After a year or two when they will visit you and you will ask to clear the debt, they will on face deny! Like this I have lost a huge amount of money. Business is tough. This is a muslim area, so people are very bad! So now I've stopped the credit thing. People know me, they know how much I charge, after years of doing business I know, who can pay how much. So if people come with less money I give 1-2 meds and rest I write them in the prescription and ask them to buy from outside, I won't give it on credit! How much can you give on credit? After whole year you see there's so much of deficit in income, with that comes family problems etc.I have to buy meds from my savings, so wife is questioning. When my father was alive he used to ask me questions! You took 10,000 to buy meds so where did the money go after treating the patients? I was cheated many times in the past, so my new resolution is, I won't care if you have money or not, if you come to me, pay get your meds. If I see I can make 100 rupees from 1 patient I will treat 5 more and get 500/day. This is how I work now. In the past I used to sell meds costing 10-12,000 but I had great losses. The funny part is when I am writing the meds in the prescription they actually go to the drug store and purchase the meds but never buy from us. They won't pay the full cash. The percentage of people who will pay cash and get the meds is very low! This is what the business in villages is all about, what to do? Educated men like us are actually not earning much.

B: (he explained it rightly)

D: Are you part of the Govt or private system?

B: Do you work with Govt or privately?

P: Private. We did some training with Govt at [town name outside site 1 redacted] in the year 2015 with the doctors of [hospital name redacted], kolkata. I was i that training.

B: In 2015, one of the biggest hospitals in Kolkata called [hospital name redacted] they gave some training and he did it. The training camp was at [town name outside of site 1 redacted] but basically he practices in private!

D: Could he explain his training to me?

B: Can you tell us about your training?

P: Training was on human, about the treatment and the symptoms. Just the basic medical treatments. That's how we used to get the training.

B: Training was on medical disease system that usually get information from the doctors. And on an average he's saying that the training which is required for treating patients and some specific terms. He's saying that was the training. Getting information about medicine from the

doctors.
D: Ok and did he do any training before he started practising?
B: Did you take any training before you started practicing?
P: Yes, before I started practicing, I used to work under a qualified doctor. I learned from him, both the theory and the practical and I also did a DMS-BMS course.
B: Yeah he had some trainings, because before starting his practice, he was under a medical practitioner for few years or months and afterwards,he is talking about BDSM-BDS course he's talking about (LOL XD) , he took that kind of training as well, he basically had his training from senior doctor who he was an assistant.
D: Did he say how many years or months he trained there?
B: How many days did you take the training?
P: Under the doctor I got trained for 7-8 years. Both practical and theory. I mean the doctor I was working under was very known to my dad, so he gave me an opportunity to work with him. His aim was, he will make me a self-sufficient man, so that in my later half of life I can earn my own living. So patients used to come to his chamber, he used to teach me and I learned some basic things too. He later asked me to do the stitching, give injections, measure blood pressure, charge saline etc or at the time of complex operations he used to ask me to stand next to him and observe the whole procedure. He used to explain to me. If not right at the time of operation but may be later on. Basically I got good training.
B: He had training, the senior doctor gave him training for 7-8 years. The doctor was close to his father so he gave him the chance to work with him. He used to take him to the surgery/operating room to show him the exact way he did it. If not then maybe after the operation was over he used to explain it to him personally. That's how he got the training.
D: Okay, umm..so which AB you have in stock?
B: Which AB do you keep?
P: If I have to talk about keeping AB then I would say..I will tell you the facts.. There's a seasonal change happening? Time is also changing, right? Now there are some ABs which I used to use 10-20 years back, now I have to change on the basis of the position (He meant the change in position of disease and composition of meds). The reason I have to change is because the AB which I gave like DS- Otrimaxazol DS are now not in use because they don't work. Yes, maybe they work a little but we don't see the desired effect. Initially I used to give cotrimoxazole DS for fever. But now if I give that temperature won't go down. Which is why now I have to give Amoxicillin-Dicloxacillin, Ampicillin-Dicloxicillin or a high power Sophixin, Cephodoxin, Cephron means Ciplofloxazin, I have to give these, I can't help. And more than this the major problem is if I give DS and the temperature won't come down then the patient will go to someone else, I can't afford to lose patients, at the end I have to earn. Now as per the prescriptions of experienced doctors that I got trainings from or from the doctors at [name of hospital redacted] hospital I've seen that they use high AB, To maintain the goodwill they use high AB, though we don't have the permission to give such high AB but we have to give, else the patients will go to them and will not continue with me. Do you now understand the problem? If you have a fever and I give you low power AB because an unqualified doctor or an unregistered doctor doesn't have the permission to give high dose AB, I will see your temperature won't go down and you won't come to me from next time. I remember at the time of our training we were told if there's diarrhoea and dysentery use metronidazole. Now you think, if there's diarrhoea and you

are given metronidazole will it get cured? No it won't be cured! So if a patient comes to me and I give metronidazole, he won't be cured, his condition would deteriorate. Despite knowing the condition, how can I not give Cephron or higher AB? The scientific community is evolving, the meds are changing, we know that through the training or talking to qualified doctors. Now if on the basis of our limitations we won't give latest or better evolved meds then our business will shut down! If I can help you or treat you proper will you come to me tomorrow again? No you won't! You will go to someone else and conclude I know nothing, so despite knowing how you can be cured, in which ever way we get to know, how can I not treat you right? In spite of the knowledge and experience if I don't treat you then will my business not go down? So I have to use high AB to patients. I have just nothing to hide from you, I have to use both the lowe power and high power AB. Someone actually asked me, do you even have the permission to use this? I answered, "NO, by law I don't. In West Bengal none of the laws work as it should be. And if I have to follow the laws then we can't survive!". Do you think I can survive? If a patient comes with a diarrhoea and I give metrozil, then I can guarantee you he will never get cured of it. It would rather deteriorate! So we have to do this, if patients would stop coming, can you say at this 45 years old age, what new work will I do? So survive, to keep my goodwill and to cure the patient I have to give them high AB. Of course there are many AB which are very powerful and are not allowed to use, in those cases we just restrict ourselves from giving them to patients. This is the fact.

B: (Explained brilliantly)

D: What does he mean by high?

B: What do you exactly mean high antibiotics?

P: High means not the very powerful ones, the moderate ones. For an example with amoxicillin cephaxin is used, ciprofloxazin was always in use.

B: By high dosage he means using amoxicillin instead of another he named, He is not talking about too high dose AB but moderate ones. (Not clear with the answer)

D: Does he mean high dosage or

B: High dosage means high performing

D: So not dosage but the actual type of AB

B: Yes actual type, it's not high, it’s moderate kind, it's medium shot!

D: Okay and why do you think that the old meds are not working anymore?

B: Why do you think the old meds are not working?

P: It's not that I think they aren't working, practically I've seen that myself. I've applied and seen it. There was no response hence I had to give the high ones.

B: He does not think, he knows that old meds don't work, practically he prescribed those medicines and he hasn't seen any improvement in the patient.

D: For what reason he thinks that they don't work anymore?

B: Exactly what are the reasons you think that they don't work anymore?

P: Reasons be like..umm..there's a resistance in the body. If you use one med for too long automatically you will see that med has no effect on you. So we have to change in that case. I always try to keep this under observation. I try a lot to use the old meds, I might give it for a day and I barely see any result. Now if I continue with that med then the patient will go to someone else. So I am forced to think and come up with a new med to cure the patient. Suppose you have a cold and cough, there's congestion in the chest, if you give DS, there won't be any

improvement. Ok? Now in place of that if I give Amoxicillin-dicloxacillin, there will be some result at least! So if I am getting some results with those meds now I have to use a little more to get better results? Now the patient might need 3-4 days to cure completely but he is not giving that much of the time. And the bigger problem is, the patient literally demands, :Doctor, I don't have time in my hand at all, cure me in two days``. So I am just given 2 days because after that he might have to join work. So there's a constant pressure, in that case I have to give the meds. In that case I have to give Cephron and all, because the patient won't give time anymore! It is the patients who say, " Give us better AB, better meds so that we can be fit in 2 days time". So we see such tendencies in our patients too, now I try to make them understand about these as much as I can, there might be some grave diseases like jaundice and all, we can't cure them in 2 days time. If they demand a week's time then, I am sorry, I can't, it's impossible! No doctors can do it. I may explain to him, "Listen, you can't be cured before 15-20 days, you have to give me this much of time". You are asking me to cure you in 2 days, can I give you an overdose? No! If in the case of 500 if I give you 1000 can your body endure so much of the power? Then they understand and respond well to the counselling. We have to know about the body's health, its power, the heart's condition, there will be a change in pressure etc. We certainly know which med will have effect on heart and kidney. We give high AB doesn't mean we are giving the very strong ones or we are giving an overdose. Suppose someone is having cold and cough and I charge a very strong AB, it is not the case. I do that within the expected limit! The modern meds are to be given within the limits.
B: (Explained right)
D: Ok and how does he decide how long and how much AB to give?
B: How do you decide how much and how long to give the AB?
P: That depends on the patient's situation and symptoms. Depending on the congestion I provide meds, I estimate the dose on the degree of problem. So I make an idea if I will give this for 2 or 4 days then he may get cured. May be I gave it for 5 days and after that if there are some remnants then I prescribe for 2 more days.
B: Depend on the situation of the patient. Suppose someone coming with fever and cold, he usually checks the coughs condition inside lungs and decides whether the patient will have more than 3 days or less than 3 days.
D: Okay and is he aware of any rules regarding AB usage?
B: Do you know the rules before giving AB?
P: Yes I more or less know it.
B: Not all but he knows, but not that specifically.
D: Does he try follow these rules?
B: Do you maintain the rules?
P: Yes, I try to maintain these rules, I observe the patient first and try to know what the patient wants to say. If the patient gives me time..I mean if a disease will take 7 days to cure, like viral fever, a viral fever will subside within a week or less than 5 days when I give a low AB. Other doctors might charge high AB but when a patient comes to me in the first stage with viral fever, I always give low AB and see whether it works or not. I give Amoxicillin-dicloxacillin for 5 days and if it never subsides then I think of something else. Now, when the patient comes to me on the 3rd day and informs me he is better then I figure out that this particular medicine is now working on the present day Viral fever! So I start giving the same to my other patients. If I see

out of 5 patients 4 got cured with Amoxicillin and 1 is not then I have to give him a higher AB. This is how I work. I always try. I try to observe the patient's body condition and treat accordingly. If I see the patient has some patience then I will start treating him with low AB, may be I will take 7 days instead of 5 days to cure him but I always start with low, now if the patient is in hurry and wants and immediate remedy then I have to go for high AB!

B: (Explained right)

D: Okay, could you describe the instructions that you give when you prescribe AB?

B: When you give AB to patients what instructions you give?

P: I do give instructions. I give other meds other than the AB. We know which AB has what side effects, some AB cause acid refluxes or acidity so along with it I give some antacids. Also when AB is given Vitamin is common. This is what I have learnt. Whether it's a capsule or syrup, Vitamin with AB is must! Patient's financial condition is also something that needs to be considered. Suppose I give a patient a high AB but he can't afford it, then what? I also have to look to that side too, right? It's not that I will check a patient and write him expensive AB. I know on seeing that he won't buy them and will certainly go to someone else, so why shall I do that? I have to see whether the patient can afford expensive meds or not. I do study the background of the patient. And depending on the work profile the patient is involved in you can make an idea. So I give meds keeping two things in mind, 1 if he can afford to buy it and 2 whether it will have any side effects or not. I always try so that the patients health don't detoriate also whether they can buy the meds. I have to consider a lot of things before writing a prescription. If I think I am some big shot who cures people and I can write any expensive meds to treat patients then I will be a fool, I have to consider a patient's financial condition and my business too. A Cephodoxin file costs Rs300, I have to see if he can afford it or not, I also have to give other meds along with it too. The total cost will be 500, I will see if he can give that huge amount of money or not. Again I have to see the AB I will give whether it will cure him or not. So I have to consider two things. SO I will only touch my pen after considering all the factors! So giving AB arbitarily without antacid or vitamin will have an effect on his heart and kidneys. It may not happen immediately but someday it will. So how can I take the risk? I have to think!

B: (Explained rightly)

D: Okay great thank you. And what are the common reasons that people come to see you?

B: Reasons people visit you?

P: What diseases?

B: common ones.

P: Fever, cold, stomach pain, cuts and wounds, boils, stitch, lack of appetite, weakness, low pressure etc. If pressure is low or body is weak then I give vitamins and recommend having good food. Here we don't have good gynae doctors, so pregnant cases come to me, abortion cases come to me, urine test this and that. More or less every type.

B: (Explained)

D: Why do they come to him?

B: Why do they come to you?

P: They come to me because..how can I say, may be they get better results from me! They may think I treat well compared to other doctors.

B: (Explained)

D: Okay is there any reason other than to treat diseases that people come to?

D: Is there any other reason that people come to you?
B: Is there any other reason why people come to you?
P: They love me. I behave nicely with patients. Whatever little I can do. suppose treatment isn't that good but they get a lot of love from me.
B: (Explained)
D: Could he elaborate what he means by the way he behaves with the patients?
B: Can you explain "good behaviour" or "love people"?
P: By love I mean behave good! There are some doctors who treat really well but their behaviour towards the patients isn't well. They might be very rough or rude. I talk very politely, so the patient also gets satisfied. I am quite jolly with my patients. These are my personal views. Why patients come to me is something I can't answer! May be my way of explaining to take the meds is very easy to understand? May be my direction to better health is appealing. These might be the reasons.
B: (Explained)
D: Ok, and do people come to you for advice regarding animals?
B: Do people come to you to take advice for their cows and goats?
P: Yes they do ask me.. there's an animal doctor who sits right opposite to my chamber, *Name redacted (Public private VPP1 [name of site 1 GP redacted])* he is not there, and a person came to me and asked, "Doctor my goat is having loose motion, what can we do?" If I see the condition of the goat is really bad and it might die without a treatment, I may ask him to have a human medicine of a low dose. Maybe metronidazol-folazoritol meds I recommended, which are low meds, as I am not aware of the treatments so I just gave a low one. So I saw, you may try. Just try! If you trust me then you may. But if your animal dies then I may not be responsible. If you know your goat will any which way die and if you trust me then why not try the med that I say. It's a rare thing. I usually never say. If people come with an urgent request then I sometimes say. The reason why I say... though I don't know how to treat them but I still gave, I could very much be blamed if the animal suffers..but still I gave is because I have discussed with *Name redacted (Public private VPP1 [name of site 1 GP redacted])* in the past. If a goat with loose motion came to him and he prescribed a med which he discussed I try to remember that and recommend it to the person, if it's an emergency.So I have the idea that when there's a loose motion of a goat *Name redacted (Public private VPP1 [name of site 1 GP redacted])* gave this particular med, so keeping in mind I prescribed! Of course the minor cases not the serious ones!
B: (Explained in his own way)
D: Umm, okay, is there any other product other than metronidazole that do you sometimes use in animals?
B: Other than metronidazole do you use any other?
P: Oh Noo!! I don't know how to treat it. What if the animal dies? There will be an unnecessary ruckus!
B: (Repeated)
P: Just because it is not in my syllabus, not my patient, it's about a goat, so I avoid it! Why get into problems? If I don't have experience and because of my med the patient dies then I will be questioned! They of course will. I am not a doctor of goats so why will I give advice and put myself in trouble? Sometimes out of sympathy, if I can see the animal is about to die I just say,

"It's understandable that your animal will die so you may try this for one last time and see if it works".
B: (Repeated)
D: So what symptoms does he prescribe metronidazole for?
B: When you give metronidazole what symptoms do you look for?
P: I give on the basis of loose motion. By symptoms I saw runny stool. So I gave on the basis of that. Since your doctor is not there and the animal will die, so just try this and see if it works. Give it a last try.
B: (Repeated)
D: Ok sure..umm.. And what do you perceive the difference between human and animal antibiotics?
B: What do you think is the difference between human and animal AB?
P: I don't know the actual difference between the two AB. But if you ask me why I advise you to give metronidazole-furazol then I will say, when I discussed meds with the other doctor, for small animals like goats, hens and calves, human ABs can be used. In case of big animals it's almost 3 or 4 times of human. I will certainly not get into an argument on that. Whatever little I got to know about animals is all because of *Name redacted (Public private VPP1 [name of site 1 GP redacted])*. He sits on that bench and I sit here, if there's a patient (animal) that came to him and I can see the condition I may ask about the problem then he tells me about it! That's all, that's how I get to know about it. Suppose a goat came with stomach inflammation. Inflammation means digestive problem, now I saw him giving carmozyme. When I asked whether human meds work on animals or not he said, yes for minor problems they do work. For adult animals there's veterinary AB but for small animals human meds also work. Now if I am too curious and ask about the dose, then he would say for an adult goat the dose is same as that of a human. Only on these basis I prescribe meds sometimes. It's not my area so why to bring trouble unnecessarily. I don't even remember when I prescribed a med for an animal last time. In 500 cases I might have said once or twice.
B: (Repeated in his own way)
D: Ok, great thank you. And is there any situation where you refused to prescribe AB?
B: Has any situation arise where you refused to give AB?
P: Yes, many cases. May be out of 10 I won't prescribe to even 1 of them. Because their problems and my problems are different (He is answering on the basis of animals and humans). I always keep this in mind, I am a human doctor not of goat's so why to take risk? I don't know may be the owner would come and create a mayhem. I have seen one thing, if it does good to you then it's all fine, he will be speak nice about me but if the animal dies then I will be foul mouthed! Since it is not within my hold why will I give a chance to create a ruckus! So I never say. Also the customers here are maximum muslims. if anything goes wrong then chances of getting attacked for being hindu is very high. So I always say, your goat will anyway die so try this, if it survives come to your doctor and get the following advices.
B: (repeated)
P: If I see someone brought an animal to *Name redacted (Public private VPP1 [name of site 1 GP redacted])* or to the Panchayat and the doctor is not there, and the case is critical I immediately refer him to [name redacted] (another animal doctor), I will CERTAINLY not treat, that's anyway out of question but out of sympathy I would ask him to go to someone who is also capable! If

he goes to that doctor then it's fine! This is the advice I can give. I give directions. I do have *Name redacted (Public private VPP1 [name of site 1 GP redacted])* phone number, if I feel really bad, out of sympathy I will call *Name redacted (Public private VPP1 [name of site 1 GP redacted])* and inquire about his whereabouts and if I see he won't come so asking the patient to sit here unnecessarily is useless, in that case I simply direct the person to another animal doctor!
B: (Repeated)
D: Ok and do you carry out any continuing training at the moment?
B: At the moment are you into any kind of training?
P: No presently I am not into any training. Last I did was in the year 2015.
B: (Repeated)
D: Okay and why is that (..?)
B: Why did you do any training after that?
P: I didn't do because I didn't get any opportunity. Neither Govermental or private. I didn't get any chances or connections.
B: (Repeated)
D: And would he appreciate training in AB usage?
B: When you use AB, the training that is need for that did you...
P: Yes yes, our rural healthcare committee has a union. From that Union we demand the Government hospital doctors of Diamond harbour area to train us about the modern medicines. Since we are not registered ones so we need some trainings. Now after demanding for 5 times they will arrange one session for us. This is how it works. This is how in a year we do get some trainings for few hours. Suppose I am treating a patient but can't properly cure him, so when we are at such trainings we discuss the problems with the doctors and get some solutions, or else we simply refer to the hospitals.
B: (Repeated)
D: And does this union has rules about AB usage?
B: Does your union follow rules regarding AB usage?
P: Union means the doctors who are associated with the union?
B: Yes the union.
P: Yes the ones who are associated including myself we do follow the rules. If we follow the rules then it's better for the patients as well as we would be safer. If I apply something for 5 times, at least for once there can be some reaction, which will create some pressure. So we maintain as much as we can. In seminars we discuss with the doctors and then use them. Also if we use something and got some undesired faulty results in that case we either discuss at the seminars or I go to the doctor I practiced under and discuss the matter. I often continue these in my profession. Suppose there's a patient with some problem, I call him and ask shall I give this med, he immediately warn me of its side effects and would recommend me to give something along with that med. So I treat the patient after discussing with my doctor too!
B: (Repeated)
D: Okay thank you, is it alright if we see the metronitrozol that you use?
