# Supplementary material for: “If It Works in People, Why Not Animals?”: A Qualitative Investigation of Antibiotic Use in Smallholder Livestock Settings in Rural West Bengal, India
Source: Antibiotics (Basel). 2021 Nov 23;10(12):1433. doi: 10.3390/antibiotics10121433 (PMC8698124; doi:10.3390/antibiotics10121433)
Supplement: Supplementary file 1 [file antibiotics-10-01433-s001.zip › Supplementary S1_ Interview Transcripts/Site 1/Key Informant 1 (site 1).pdf]

Code for Study - ‘If it works in people, why not animals?’: A qualitative investigation of antibiotic use in smallholder livestock settings in rural West Bengal, India: Key Informant 1, Site 1
Date: 26/06/2019
Location: Site 1
Interviewee: Key Informant (a senior high school teacher- KI1)
Interviewer: Jean-Christophe Arnold (J-CA)
Transcription: Debanjan Debnath (DD)

I: Interviewer (J-CA)
P: Participant (KI1)

N.B: the terms veterinarian and doctor are used by the participant to describe any provider of animal or human health and do not describe fully qualified and licensed veterinary and medical practitioners.

START OF INTERVIEW

I: How many households are in *[village name redacted]*?

P: So, there are almost 900 voters, how many households would that make? Around 300-350.

I: What kind of jobs do people have in the village?

P: Oh job, mostly they work for the company, *[company name redacted]*. Both men and women work for the *[company name redacted]*. For that there has been an economic development. And one more thing I'd like to add is that most of the population is less educated.

I: What about the economy of the village?

P: The modes of communication have improved, like the roads have been made into concrete roads. And as I said most of the population work for the company, so there's an improvement in the economy of the village.

JCA removes a sheet of A4 paper with images of different livestock species listed and prepares to begin taking notes

I: We are now going to the next section of the interview. About the livestock in the village...

P: (Interrupting)... As for the livestock in the village there are goats, ducks, chickens, cattle (...) People raise cows here and there.

I: Let me just confirm that we are talking about the village *[village name redacted]*?

P: Yes, yes, what I am saying is absolutely true! Because I have been to the village, my relatives stay there.

I: What sort of livestock are in the village?

P: As I said, cows, goats, chicken, and the way people keep ducks. Chicken, cows, ducks are there.

I: Apart from these are there any other kinds of farming in the village?

P: Yes, yes, there are a few fishermen, and farmers. (Mumbling).

I: Where do the fishermen fish?

P: Them... they fish in the river.

I: Apart from chicken, ducks, cows, goats are there other livestock?

P: No, that's it.

I: Out of the ones you mentioned, which one is most common?

P: Most common? Cows.

I: Out of the 300 households, how many houses would have cows?

P: Cows (...) in *[village name redacted]* at this point cows, to think, (...) almost half of houses would have cows.

I: Out of the chickens, ducks and goats, what do they form?

P: Chicken, ducks would do one fourth (...) no, one third of the total number of houses! One third of the total number of houses.

I: Out of 300 houses?

P: Yes, out of 300 houses around 100 houses would own chicken and ducks.

I: Do they own a mixture of chicken and ducks?

P: Yes, a mixture of chicken and ducks stay in one household.

I: Are there people who own one or the other?

P: Yes, there are people who keep only chicken or cows.

I: Out of approximately 300 households, how many would own a mixture of all of the livestock you have mentioned?

P: A mixture of all kinds would be (...) 50 to 60 of the total.

I: The most common would be…?

P: The cows.

I: What's the main reason for the households keeping livestock?

P: The main purpose is making money.

I: As for the other animals, are there other reasons?

P: That's all the same! Making money! Only purpose is to earn money!

I: Do the villagers use the products for themselves?

P: They use some of them for themselves and the rest they sell outside.

I: Which one of them is sold the most?

P: The milk is sold the most.

I: And the others?

P: The ducks and the chicken and the rest are also sold outside.

I: Do they sell the eggs?

P: (nods)

I: So, they are kept for the eggs (...)

P: Meat. the eggs and the meat.

I: And for the ducks?

P: The same.

I: What are goats kept for?

P: The goat meat is sold for money.

I: For the meat?

P: Yes, for the meat. Certainly!

I: How about the goat's milk?

P: They drink the goat's milk at the house.

I: And the cows?

P: The cows are for the milk, and when they grow old they are sold for money. Clearly for the meat.

I: How many animals would people keep per house? For example, if a household keeps cows, how many (...)

P: The ones that own farms, in one house there would be 6,7,8, maybe 10 cows.

I: Do you mean the ones that grow crops?

P: Yes. But mostly they keep 1 or 2 cows.

I: So, most people own 1 or 2 cows but how many (...)

P: (Interrupts) very few of them have 5-6 or 10 cows.

I: As for the chickens, how many chickens would a household keep?

P: There would be 8-10, 15-20 chicken

I: Is there anything bigger than that? more than 15-20?

P: No! Chicken is not raised on a large scale.

I: As for the goats, how many goats would a household keep?

P: 6, 8, 10.

I: Are there any commercial farms where people might do large scale (...)?

P: Farm animals? No, nothing like that is there!!

I: How about poultry farms?

P: There are no poultry farms. Just around 8-10 or 15-20 stay in their houses. There are none that raise 200, 250, 300, 400 chicken.

I: In *[village name redacted]*?

P: Yes, I'm talking about *[village name redacted]*.

I: You said most of the houses keep cows, why is that?

P: Cows for the milk for themselves and to sell. Most of the houses that keep 1/2 cows consume some of the milk and sell most of it. Because as I said, they are poor, they think a cow produces 300 milk, in a day about 4-5 seer milk *[a "seer" refers to a traditional unit of mass or volume used across Asia with national and regional variations to its definition, in this case used interchangeably with liter]*. They would probably keep half seer for themselves and sell the rest. They would probably drink 1/2 liter and rest of 4/5 liter they would sell.

I: Compared to the other animals why would they raise cows?

P: Raising cows is more profitable!

I: Who in the household would own the animals?

P: The owner of the house. The senior person in the family.

I: Is it the same for all the animals?

P: No, it's different for other animals. In case of chickens and ducks the owner would be the mother in the family.

I: Why would the owner of the family also own the livestock?

P: The reason is to make money. Who would take income? Obviously, the owner. For example, the reason people raise cows is making money. Obviously, the owner of the house would take the money.

I: In that case, why is it women that own the poultry?

P: It's usually the women that take care of the poultry. The men don't usually take the money. The women keep it. The chickens, ducks etc. are usually reared by the women.

I: Why is that?

P: It makes less money, also it would be a different source of income. The men would work elsewhere. It is usually the poor that rear poultry. the women take care of the poultry and the men go to work outside.

I: A similar question. Would the men in the family take care of the cows?

P: If a household has 6, 8 or 10 cows, the men in the family would take care of the cows. but in case of 1-2 cows the women would take care of them.

I: Who would own and manage the goats?

P: The women.

I: And for the poultry?

P: The same.

I: Is there another reason?

P: It has less money. And the men don't usually look after them. It could be some additional income for the housewives to help them save up.

I: Where do people learn how to look after the livestock?

P: Where do I say here, they just learn it by themselves. A son would learn from his mother. They inherit the knowledge.

I: Is there any other source they would learn it from?

P: Erm... sometimes the government would organize poultry training for them to learn.

I: We are going to the next section now. Here we will talk about what people do when the animals get sick.

P: When the animals get sick, the local veterinarian is called. For example, every Anchal has a veterinarian. They are either called or visited. This is how people treat their animals. For example, if a cow gets sick, it would be brought to the Anchal *[referring to the local Grampanchayat building where the government Livestock Development Assistant practices from]* or the doctor will be called to the house. This is how the animals are treated.

I: Are there differences for different kinds of animals?

P: It's the same doctor. And they are all taken to him.

I: Are the chickens and ducks treated the same way?

P: They are all treated the same way. In case of the cows, the doctor is often called to the house.

I: In case of the poultry, the goats...

P: (Interrupting) they are either taken to the vet or sometimes they are given homeopathy medicines. They would go to the homeopath and say what's wrong. A lot of people in this area go to the homeopath, they would go to the homeopath and say what's wrong with the chicken or the goat.

I: Is it practiced in the cows?

P: Yes, but the cows are mostly taken to the vet *[referring to the LDA]*

I: Are all the animals treated the same way, in terms of medical care?

P: Yes, medical care is always given. If the animals are sick they are always treated. People are very aware in that sense.

I: Is the vet qualified?

P: Vet is qualified *[referring to LDA].*

I: Is the veterinarian trained and qualified or does he practice informally?

P: He's trained.

I: Is there anyone else they would go...

P: (Interrupting) Go to? There are a couple of others with short training.

I: What do you mean by "short-training"?

P: 6-month course on veterinary treatments. There is one such doctor in almost every village. They are called for treatment.

I: Would people seek advice about how to take care of the animals from other community members?

P: Yes, they help each other. When animals get sick, they help each other out.

I: Is there any situation where they would not go to the vet and look to treat in some other way?

P: Sometimes the doctor might not be available.

I: Do they go anywhere else for treatment?

P: They might go to the neighboring village or the village next to it, the doctor might be called from afar.

I: Would they go directly in case the doctor isn't available?

P: Yes, they go and buy the medicines from the pharmacy directly.

I: The way you said they go to the pharmacy, where else would they go for treatment in care if the vet isn't available?

P: Erm... what do I say, they go allopathy quack doctor (laughs).

I: Are there other options?

P: No, there's no other option. This is how the treatment goes on.

I: The way you mentioned they would sometimes go to the pharmacy and the other sources... is there a scenario that one would be taken to the vet and others would be taken to the pharmacy?

P: They are all treated the same way.

I: Is one animal given more priority than the others? for example...

P: No, no, the priority is the same.

I: When the animals get sick, where would they go first?

P: They would always go to the vet first. People are much more aware these days.

I: For what reasons would they go elsewhere than that?

P: That is when the veterinarian isn't available. They go to the vet first if he's not available then they would avail other options. Like humans also go to the quack doctor *[“quack” is a term used in India to describe a medical practitioner who practices without formal qualifications or training]* if the qualified doctor isn't there. Why would anyone go to the quack doctor instead?

I: How often is the veterinarian available?

P: He's mostly available.

I: How far is the vet from Sriphalberia?

P: 1-1.5 km.

I: What do people in the community think of the vet?

P: (laughs) what do I say... It would be better if there were more vets. Because the entire Anchal has one vet

I: Do you mean there's one vet per GP?

P: Yes, per GP *[Grampanchayat]* there's one vet.

I: So, when the GP vet is unavailable, they would go to other sources. How often does that happen?

P: Erm... approximately one fourth of the time.

I: So, one fourth of the times the vet isn't available?

P: It's mostly manageable!

I: You said there's a vet 1 km away from *[village name redacted]* and it's mostly accessible to the villagers. Are there any reasons people would go to other providers?

P: They don't prefer it. They would go to other sources when the vet isn't available.

I: So, there's no exception to this scenario?

P: Well, sometimes if people aren't as aware. they would probably go here and there.

I: what would be the number of people who aren't as aware?

P: Very few. about 5%.

I: In *[village name redacted]* how many of these providers are there?

P: There's two homeopaths and one allopath.

I: Is there a pharmacy or drug shop?

P: No.

I: Apart from the homeopaths and allopath is there anyone they go to?

P: No. Not in the same village. but there are other doctors in different villages.

I: Do they outside the village?

P: (Continues) For example, there's a vet *[referring to the ADV]* in *[village name redacted]* village. He goes to *[village name redacted]* to treat. He goes around the Anchal giving treatments.

I: And he's different to the veterinarian of the Anchal?

P: No, Anchal has a different doctor, he's just from the village.

I: Is he one of veterinarians with short-term training like you mentioned before?

P: yes, yes, he has some training. He isn't very qualified. He has a bike; he gets a call and he goes around the village.

I: What is he called? Is he also called a vet?

P: Yes, he's also called a vet.

I: Among the ones that you mentioned who treat animals, does anyone treat humans too?

P: The homeopath and the allopath treat humans.

I: So, they treat both humans and animals?

P: yes.

I: Are they qualified doctors?

P: yes. The homeopaths are qualified. The allopath isn't qualified. He has received one-year training.

I: Does the homeopath have a degree?

P: Yes, he has DHMS degree.

I: Does the GP vet provide human healthcare?

P: No, he just treats animals.

I: Is there anymore providers in the village that treat the animals?

P: To my knowledge, there's just one.

I: And you said they treat humans and animals?

P: yes, the homeopaths and the allopath treat both humans and animals.

I: Is there anyone apart from these?

P: (shakes head)

I: Are there any uniquely human healthcare providers in *[village name redacted]*?

P: They treat both humans and animals.

I: There's anything only for humans?

P: Now a days whomever treats humans also treats animals. (laughs) The two qualified homeopaths are good.

I: Out of different healthcare providers you have described, the different owners of chickens, ducks, and cows. Where do you they usually go for the treatment of their animals?

P: the chicken and the ducks mostly go to the homeopath, or the allopath. They aren't taken to the vet very often. The goats and the cows would be taken to the veterinarian.

I: If the vet isn't available where would they go?

P: Then even the cows and goats would be taken to the homeopath.

I: Do they go to the allopath?

P: not often.

I: Is there any difference in the way the owners of chicken or ducks or cows seek treatment from these different sources?

P: erm, what do I say, what we see in the village is that the chicken and ducks are usually taken to the homeopath and the cows are taken to the vet.

I: Where do they go in cases the vet isn't available?

P: Then they would be forced to go to homeopath as well.

I: When is the vet from the neighboring village used?

P: When the cows get sick, if the vet isn't available.

I: Would he be called before or after the homeopath is consulted?

P: Before. if he's not available in that case...

I: Does the homeopath give antibiotics?

P: (Laughs) I don't know.

I: The next section is specifically about antibiotics.

P: Go ahead.

I: Do you know whether antibiotics are used on livestock?

P: Yes, it's used.

I: Is there a difference in usage among different animals?

P: I don't know.

I: Among the doctors the villagers go to, which ones give antibiotics? as you said you didn't know if the homeopath gives antibiotics, how about the others?

P: To my knowledge, The GP vet, and the vet from this village gives antibiotics.

I: How about the Allopath?

P: Yes, He also gives.

I: Why are antibiotics used in the livestock?

P: Reasons would be (...) for diarrhea, fever, antibiotics are used.

I**: Antibiotics are used to treat diseases or are...**

P: (Interrupting) Yes, they are used to treat diseases not for the prevention.

I: How much people know of different diseases?

P: People who raise animals, approximately one fourth of them know. If the animals have fever, or has diarrhea, or Kath, mostly a cow has fever, diarrhea or has Kath on its legs.

I: What is Kath?

P: It's a disease, the cows can't stand on its legs.

I: Is the treatment based on the symptoms or on specific disease knowledge?

P: The symptoms. The treatment is based on the symptoms.

I: Are there any other reason that they would use antibiotics, for growth or preventing disease?

P: No, No!

I: Who gives the antibiotics to the animals?

P: The owner.

I: Do you know why?

P: The doctors don't come to give the medicines. They ask the owner to feed the medicines. The doctor gives the injection when needed, but the medicines are given by the owners.

I: Does the vet come to the household or do they go the vet asking for treatment?

P: as I said, when they call the vet he goes to the household.

I: So do people call the vet to come to the house or...?

P: (interrupting) in most cases people call the vet home.

I: This is the final section. Are there any situation where human antibiotics are used in animals?

P: (laughs) It happened in certain cases, which we have heard before. Mostly the quack doctors use this. They use human antibiotics on animals.

I: You have heard it?

P: Yes. What do these quack doctors do is, if the animal has fever, they would give human antibiotics which is meant to treat fever to the animals because they don't have medicines for animals. They just increase the power. (laughs)

I: Is there any other reason why human antibiotics are used in animals?

P: No, they do it because they don't have it. When they don't have animal antibiotics, they use human antibiotics. This is the reason.

I: Which providers do this?

P: The allopath, the quack doctor. Others don't.

I: Can you please confirm which doctor gives antibiotics to the animals?

P: The GP vet, the trained veterinarians in the village, and the allopath give antibiotics to the animals.

I: Which doctors give human antibiotics to animals?

P: (Laughs) Gives human antibiotics to animals? The local quack allopathy doctors.

I: Do you know of any situation where animal antibiotics are used in humans?

P: No, this doesn't usually happen (dismissively). No, this doesn't happen.

I: The following questions will be regarding the difference in perception between human and animal antibiotics. Do people in the village understand any difference between human antibiotics and animal antibiotics?

P: They don't understand the difference.

I: Do they understand what antibiotics are?

P: Some do. 2-3% of people understand.

I: The providers that give both human and animal antibiotics, do they understand the difference between human antibiotics and animal antibiotics?

P: They do.

JCA returns to his sheet of paper where the livestock systems described, and providers identified have been written down

I: The interview is basically finished; we just need to clarify a few things. If you just give us a little bit time. So, in the village we have chicken, ducks, cows, goats and some there is a mixture? How many villagers would own 8-10 cows and how many would own 1/2?

P: Let me think. How many would own 8-10 cows (...) how many people did I say owned cows?

I: half of the people...

P: (Interrupting) 10-12 houses who would keep 8/10 cows. the rest of them would have 1/2 cows.

I: Can you confirm that there's just one vet in the GP?

P: Yes.

I: Within the village there are two homeopaths and an allopath?

P: Yes.

I: And there is a village vet who has less training who comes to *[village name redacted]* to visit?

P: Yes. He visits. He sometimes just goes to the village to take a look around without even being called.

I: The GP vet, the village vet and the allopath provide antibiotics?

P: Yes.

I: Any of the others?

P: No!

I: Does the allopath provide human antibiotics to animals?

P: Or others also do. Imagine someone from the village has come to *[village name redacted- a neighbouring village]* they buy medicines from the pharmacy there. They would tell them what's wrong with the cow and the pharmacy would give them the medicines.

I: do they provide human antibiotics?

P: Yes, what else!

I: Outside of the village what other animal healthcare providers are there?

P: There are village quack doctors in other villages.

I: Is there anything else?

P: Apart from quack doctors there are some qualified doctors in other villages. You asked about the village, so I described the situation in *[village name redacted]*. But people from *[village name redacted]* would come outside the village to the market and get some medicine from the pharmacy. This is how it is. Or they would go to a quack doctor outside the village and get treatment from them.

I: Are these for human healthcare?

P: Yes.

I: Would these human healthcare providers also treat animals?

P: Yes. It's all the same now. (laughs)

I: What kind of antibiotics would these providers use on the livestock?

P: I don't know.

I: Do they use human antibiotics on animals like ones in the village?

P: Yes, yes, it's the same.

I: In which situations would the villagers go visit the providers outside the village?

P: It's like when they are on their way to somewhere, they would go and get treatment from these doctors. And sometimes people prefer one quack doctor to another. There's a difference in the quality sometimes.

I: Why would they prefer one to another?

P: Some of them have better experience and they give better treatment.

I: Is anyone of these outside vets preferred to the vet of *[site name redacted- site 1]*?

P: The Village vet is sometimes preferred. If he's not available, then they go to other vets.

I: You had mentioned in the beginning that people prefer the GP vet and now you just said that sometimes people prefer the Local vet to the GP vet, why's that?

P: When the GP vet isn't available, then the villagers call the village vet. If the GP vet refuses to go the household, probably the local vet would go. as I said the GP vet isn't always available.

I: We are finished.

END OF INTERVIEW
