# Supplementary material for: “If It Works in People, Why Not Animals?”: A Qualitative Investigation of Antibiotic Use in Smallholder Livestock Settings in Rural West Bengal, India
Source: Antibiotics (Basel). 2021 Nov 23;10(12):1433. doi: 10.3390/antibiotics10121433 (PMC8698124; doi:10.3390/antibiotics10121433)
Supplement: Supplementary file 1 [file antibiotics-10-01433-s001.zip › Supplementary S1_ Interview Transcripts/Site 1/Key Informant 2 (site 1).pdf]

Code for Study - ‘If it works in people, why not animals?’: A qualitative investigation of antibiotic use in smallholder livestock settings in rural West Bengal, India: Key Informant 2, Site 1
Date: 12/07/2019
Location: Site 1
Interviewee: Key Informant (a human homeopathic doctor) (n.b. this is a different human homeopath as the antibiotic provider (Homeopath 1)
Interviewer: Jean-Christophe Arnold (J-CA)
Transcription: Debanjan Debnath (DD)

I: Interviewer (J-CA)
P: Participant (KI2)

START OF INTERVIEW

I: Could you briefly describe the village?

P: It's on the river Hoogly. [information locating the village redacted].

I: What kind of community live in the village?

P: Fishermen. Most people are fishermen. They fish in the river, or the sea.

I: What are the other jobs that people do in the village?

P: Cultivation, a variety of cultivation. Tomatoes, crops. Although crops aren't very profitable right now it depends on the weather.

I: Apart from these what other work people might have?

P: There's one teacher, there are two doctors. Otherwise people are very poor.

I: How important are livestock in the village?

P: It's very important but it doesn't happen here as such. 30 years ago, the block had given some animals, it was a failure. They gave cows, or goats. That was a failure. There aren't enough fields, and also people need money. There are no farms, and no dairy.

I: What kind of animals are in the village?

P: They are in a very poor condition. They don't have their own house. If someone is keeping two chickens, they would just cover it with a net and keep it.

I: What kind of species of animals are in the village?

P: Cows, Goats, Ducks and chickens. Some here keeps different kinds of birds.

I: What's the most common animal in the village?

P: Cows.

I: How many cows would people generally own in a household?

P: 1 or 2. Very few people would own around 4. Because to raise cows you'd have to give them food. The ones that farm feed that straw.

I: How many goats would people own?

P: Around 4.

I: What about chickens and the ducks?

P: 4-6.

I: And ducks?

P: the same. No one does it in a bulk.

I: Do you people own a mixture of animals?

P: No, nothing like that. It's more of a hobby, not a business for them. They don't earn much from there.

I: Aren't there houses where there are both cows and goats together?

P: Yes, there are few houses like that.

I: How many houses would have that?

P: 4-6, in the village.

I: How do you know this?

P: The ones that raise animals come to me for medicine. We can see it around. Like that house at the back has 6. They also have to make profit from it. Why would they spend money on food unless they are making profit? You also have to spend time on them, take care of them, and give them food. People come to me for Homeopathy treatment. Someone cared for their chicken, I gave medicine, it got better.

I: For what purpose are the animals kept for?

P: Milk. Now cows aren't used for harvesting. It's done by the machines.

I: Talk about the rest, the chicken, the ducks, and the goats?

P: The eggs and meat from the ducks.

I: the chicken?

P: the same. Eggs and meat.

I: The Goats?

P: The meat. They sell it when they grow in numbers after 6 months to 1year. That's the benefit.

I: The products that they get from the animals, do they have it for themselves, or do they sell it outside?

P: They sell it outside!

I: Who owns the animals in the family?

P: The women. Housewives. Because the men in the family leave for work.

I: Who looks after the animals?

P: Housewife.

I: Anyone else?

P: People who are home they all do. The men go to work, the rest help out.

I: Regarding the ownership of the animals, is it the same of all the animals or is it different?

P: It's the same.

I: You will be able to say how the animals are looked after?

P: The housewife gives the food, takes care of them, they take them out, and bring them inside. It's their work.

I: How do people here learn how to look after the animals?

P: The GP gives them training. 7-15 days training.

I: Is it a 15-day training, or does it happen every 15 days?

P: No, it's a 15-day training. maybe once in a year it happens, whoever is interested to learn would go. It happens for the fish too. Once or twice a year the GP gives training. Interested people go, sometimes women are given chicken by a women self-help group. The women farm those.

I: How else do people learn how to look after the animals?

P: Some of them do it for generations and they learn from the family. If there's something new, or the government is helping somehow, they receive training.

I: There are no large-scale farms in the village?

P: No! Animal farming doesn't happen here.

I: What do people do when animals get sick?

P: They go to the GP doctor [referring to the LDA]. For something minor they come to us.

I: Who are the other animal healthcare providers in the village?

P: [name removed- pranibandhu], who lives close to [name removed- allopathic quack doctor] is the only one.

I: You said you also give treatment?

P: Yes, homeopathy. For diarrhea, worms, etc.

I: So, who else is there apart from you and [name removed- pranibandhu]?

P: There's [name removed- allopathic quack doctor], and no one else! You have to go to the GP.

I: Are they found in the village?

P: Yes.

I: Do people go outside the village for treatment of the animals?

P: The GP, if the GP refers then you go to the block.

I: This process of treating the animals, is it different for different animals?

P: Yes, different animals need different treatments.

I: I mean with regards to where people go and get treated, is it different for different animals? Imagine a cow is sick, or chickens are sick, would they be taken to the same doctor?

P: Yes, The same doctor at the GP. There isn’t any other veterinary surgeon in our village.

I: Is there anyone else who gives advice for animal health or treats them?

P: There's no one else!

I: Do you know whether antibiotics are used in animals in the village?

P: Yes, of course. How will you do without antibiotics?

I: For what reasons usually antibiotics are used?

P: For serious diseases it has to be used.

I: Is there any specific disease that antibiotics have been used for, do you know of them?

P: No, I don't.

I: Which animals are likely to receive antibiotics?

P: Cows. only cows and goats.

I: Out of the people who provide animal healthcare here, who gives antibiotics?

P: The GP vet [LDA]. He comes out sometimes to see patients.

I: Is there anyone else?

P: No!

I: Do you know if people in the village use antibiotics for growth of the animals or prevention of diseases?

P: No.

I: You don't know?

P: No.

I: When antibiotics are being used in animals, who would usually give the antibiotics to the animals?

P: Whoever looks after the animals, feed them.

I: Do you know of any guidelines for antibiotic use?

P: There are no guidelines.

I: Are you aware of any?

P: No.

I: Have you heard of any situations where human antibiotics are used in animals?

P: Yes. certainly.

I: When does it happen?

P: When they fall sick they might give any antibiotic. I have heard that it happens, but I don't know the details. The veterinary doctor would know.

I: Do you know why this might happen?

P: No I have no idea.

I: Have you heard of any situations where animal antibiotics are used in humans?

P: No, I don't know.

I: Do people know what antibiotics are here?

P: No, they don't know. They are illiterate, how would they know! They would only know if they knew anything about medicines in the first place!

I: Do you think people are aware of the difference between human and animal drugs?

P: Most people don't know.

I: Why do you think that might be?

P: Because they are unaware! If they were literate, and aware they would know the difference.

I: Thank you

END OF INTERVIEW
