# Supplementary material for: “If It Works in People, Why Not Animals?”: A Qualitative Investigation of Antibiotic Use in Smallholder Livestock Settings in Rural West Bengal, India
Source: Antibiotics (Basel). 2021 Nov 23;10(12):1433. doi: 10.3390/antibiotics10121433 (PMC8698124; doi:10.3390/antibiotics10121433)
Supplement: Supplementary file 1 [file antibiotics-10-01433-s001.zip › Supplementary S1_ Interview Transcripts/Site 1/Key Informant 3 (site 1).pdf]

Code for Study - 'If it works in people, why not animals?': A qualitative investigation of antibiotic use in smallholder livestock settings in rural West Bengal, India: Key Informant 3, Site 1

Date: 18/07/2019

Location: Site 1

Interviewee: Key informant (a high school teacher)

Interviewer: Dominic Day (DD)

Translation: Somraj Das (SD)

Transcription: Sayak Manna (SM)

D: Interviewer (DD)

B: Translator (SD)

I: Interviewee (KI3)

START OF INTERVIEW

D: Thank you very much for agreeing to talk to us

B: thank you Sir.

D: Can I start by asking you to describe *[village name redacted]* village?

B: Could you describe [village name redacted] village?

I: [village name redacted] is a large village surrounded by the river Hooghly. There are corn fields, and the river Hooghly passes by it. The area is predominated by Muslims and Hindus. Some Christians live here as well. Once the British came here and established their forts at [village name redacted]. If you go over here, you can find many forts and shrines of that time. The British came and established their forts, the debris of which to be found even today.

D: What communities live here now?

B: what kind of communities lives here by the way, right now?

I: Hindus, mostly Hindus. 30 to 40 percent Muslims live here.

D: How many people?

I: About ten thousands, many of them are poor who cultivate crops and catch fish to earn their living.

D: Is that the main economic activity?

I: They depend mainly on agriculture, they catch fish. They do farming, and produce vegetables. These vegetables are sold not only this village but outside of it. The fishermen catch fish and they sell it, not only within [village name redacted] but also outside this area. Fishes, cultivated lands and agricultural products are the things upon which the people of this village depend.

D: okay. What kind of activities aside from these do people do?

I: Some people are teachers; I was also a teacher for some time. Some people are employed in the government sector. I was also an employee of the West Bengal Government. I worked for more than thirty two years, after that I retired and most people are farmers.

D: okay. What kinds of Livestock are normally kept?

B: repeats.

I: People in this area, who are poor generally, keep cattle such as cows, goats and chicken. They at times also keep pigs. They feed the cattle grass collected from the cornfield. They feed the cattle with wheat and other agricultural products. And straws, straws means dried grass. This is also used as a feed for the cattle. Rice straws are mostly used as a feed for them.

D: okay. How would you classify the systems?

I: this system can be classified into two groups. One group uses it just to earn money, to live on and another group does it as a trade or business. Some people do this for commercial business purpose. Understood?

D: Yes. And which one would you say is the more common system?

I: What did he say?

B: which is the most common system between these two?

I: the common system is, those who are poor they usually keep cows and goats. And those who do it for the business purpose, they keep only cows, and buffaloes. They collect the milk and make sweets out of them. These sweets are sold in shops, not only in shops of [village name redacted] but outside as well.

D: So, what’s the main purpose of keeping livestock?

I: The main purpose is to earn money, which is necessary to carry out their (???) business.

D: So, do people farm fish in this village?

B: Is fish farming done here?

I: Yes. It is. They do. The ponds are filled with water and various types of fishes are farmed. They produce fish of various types. They collect the fish and sell the same to other cities like Diamond harbour and Calcutta. They produce the fishes in the ponds that they use for the agricultural purpose. And some other kinds of fishes are collected from the river, such as the Hilsa, they are sent to Diamond harbour and other cities including Calcutta. This helps them improve their economy. Understood?

D: Yes. What sort of proportion of people living in this village own livestock?

B: what is the ratio, what's the difference of people keeping livestock such as cows and goats and such?

I: The poor people keep about thirty percent of the cattle, use it twenty percent for the business purpose of commercial purpose.

D: Who normally owns the livestock in a household?

B: Who owns the livestock here?

I: the owners of the livestock are mainly villagers earning less than 6,000 rupees per month and those who do business (with livestock) earn more than fifty thousand per month.

D: and within the family, who normally owns them?

B: are the family members themselves the owner of the livestock?

I: The poor people, who go on doing business (in a smaller scale), do it along with their family. And those who do trading in a larger scale do it with other men employed, not with family members. They employ other people to carry on the business.

B: ownership varies.

D: pardon?

B: ownership varies.

D: Yes. Who normally looks after the animals?

B: usually who looks after?

I: of the business?

B: of the livestock.

I: those who are poor, they themselves look after their livestock and those who have a business purpose, have men employed to look after them.

D: Who within the family looks after them?

B: Sir, who looks after the livestock in a family? Is there anyone particular or every member of the family? That is the question.

I: that is what I answered just now. In poor families, all the family members take part in looking after the animals. And in business some people are employed, not from the family but from outside, some people are employed to carry out the business.

D: okay. And how are the animals normally kept?

B: how are the livestock kept?

I: (laughs) for the business purposes safe spaces are build. Cattles are kept there, fed and nourished well.

D: and is that the same for all different types of animals?

I: this system continues for all the types of cattle and livestock.

D: and also for the chickens and the ducks and other…

I: Well, some people, living just outside my house, they keep chickens for eating purpose as well as for business purpose.

B: you mean for personal consumption and commercial consumption.

I: yes. I mean for personal consumption and business purpose.

D: And how do people normally learn to look after the animals?

B: repeats.

I: Yes, this is a vital question. For the poor people, they learn it from their old parents. For the business purpose they learn it from their father, father's father and grandfather.

D: So same?

B: is it for the business purpose as well?

I: yes. It is the same for the business purpose.

D: so, what happens, what do people do when their animals become sick?

B: what do you do when an animal gets sick?

I: when the cattle suffer from any disease, they go to the Panchayet (referring to the GP). There is a system to provide antibiotics or medicines for the treatment of the cattle. But for business purpose they provide medicines from other places like some other town or Calcutta. If it is for commercial purpose

they buy it from outside and if it is for the people of the villages, then they are provided with the medicines from the Panchayet.

B: When the livestock gets sick in the domestic purpose they usually do it by themselves, they go to the Panchayet or GP (General Panchayet) for medicines. And for commercial purpose it differs, they take much more special care. Because it's commercial, they provide some kind of special medicines and advices as well.

D: who do they see in the GP?

B: Who looks after the cases in general Panchayet?

I: there are some selected doctors who look after the livestock. These doctors are appointed by the west Bengal government. Understand?

D: didn't get the last bit.

B: Those doctors of the GP are directly appointed by the health department of west Bengal government.

D: Is there anyone else that people might go to?

B: repeats.

I: yes, there are some doctors who people might go to for their treatment of their cattle. Doctor [*Name redacted]*, whom you have already consulted, is one of them. He is very helpful. And there are some other doctors, here and there, I can't name them. They might be called in, when an animal is suffering from some disease. They come and provide medicines for them. Actually they are quacks. They do not have any degrees.

D: would they talk to these people for advice about keeping their livestock?

B: how do these quacks advice them on how to keep the animals?

I: they go to the doctors and present their problems to the doctors. The doctors listen to the problems. And there after the doctor provide the medicine or other treatment to those suffering from diseases. It is effective. It provides results. I have seen that people with problems go to these quacks and they are provided with medicines, and then the animal gets cured.

D: is that the reason that people go to one quack, or another quack? What are the reasons that people go to the providers?

B: is there any other reason for the common people to go to these people? Why do they not go to someone else?

I: one reason is that, they are financially backward. It is difficult for them to pay more money for efficient doctors. For this reasons, they go to the quacks who take minimum fees. It is the answer to your question.

D: are there any other reasons why people… (go to them)?

I: the quacks are quickly available. Whenever they are called, they come. They come by bikes or cycles and provide medicines necessary for the cattle.

D: and do people go to the same providers for all the different animals?

B: Do people with various livestock go to the same doctor only?

I: No, no no. There are different kinds of doctors. Some treat cows, while other doctors treat dogs, some treat chickens. So, the quacks are of different types.

D: Okay, great. And what makes them different from each other?

B: how are they different from one another?

I: The difference is that, the quacks are not highly qualified. They learn their knowledge of medicines from their parents or grandparents. From this experience they provide help to the poor who call them for the treatment of the cattle. This is the difference, because they have no higher education in medicine, this is the reason. And those with higher education demand more fees which the poor people can't afford.

D: Do people perceive different quacks in different way?

B: Different doctors treat different animals. So, what is the basic difference?

I: Suppose a cow is suffering from a disease. It is a different case from a goat suffering from a disease. The ??? is different. Suppose chickens are dying in numbers then, they'll go to someone, a quack who has knowledge about poultry and chickens.

D: Does anybody else provide advice or care for the livestock?

B: sir, is there anyone else who can give information like you?

I: No, there is not a single man who can talk to you in English. I'm the only one. I'm the only one who can communicate with you. Others will not understand you. They are so illiterate or under educated that they can't understand your questions. Only some teachers are there, here we have a school, but school hours have already ended. They could have communicated with you.

B: And do they have the same knowledge that you have?

I: I'm in the member board of that school's managing committee. So I have a clear idea about the teachers of the school. I know who can answer your questions and those who can't answer your questions. There are three teachers who can answer your questions but they can't speak as fluently as me. You have to work a bit hard for them

B: That is not a problem, I'll manage.

I: Those who teach Biology and Botany, they can talk to you in this regard with much information.

B: he's saying that there is a high school over there. We can talk to the biology teachers from that school as they might have valuable information on livestock.

I: There are three high schools, in about two miles from here. They might be able to answer your questions but not as fluently as me. They might hesitate to answer your questions, though they have the proper knowledge, they can't express it in English. I am well known in this area for speaking in English, [*Life history redacted]*. For this reasons I am invited to cultural functions.

D: So, other than the people spoken about, does anyone else provide antibiotics for the livestock?

B: Do you know anyone who provides antibiotics to livestock?

I: yes, I know two doctors. I cannot remember their names, I am too old. I'm over 60 now. Those two were my students actually, they provide medicines to cattle. They live a little away from here. If time permits, I can take you, they live about half a kilometre away. I can't remember their names. They are my former students.

D: Can we have the information of these people?

B: He is saying that he can take us there as well. It's half a mile away. Okay. Let me ask him that. Sir, if we don't mind can you take us to them the next day when we come?

I: yes, if you come to me tomorrow at about 9 to 9.30 in the morning, I can take you to them. There are three doctors; two of them are my former students. They can't speak in English so you have to translate for them.

B: it does not matter whether they can speak English or not, since I'm here. Sir, if we come here by 10 tomorrow, will it do, because we'll be coming all the way from Kolkata.

I: By ten? Alright, I'll wait for you. Yes, I understand. I'll take you to the high school teachers and also to the doctors. They have dispensaries. You can sit and talk over there.

D: that sounds good. Thank you very much. We'll be here by ten.

I: How do you come from Kolkata?

B: we have a privet car. We have no place to stay here.

D: Can we get back to the interview and discuss these things after the interview? Okay?

B: well, he started it. Start questioning again.

D: Do people go to pharmacist or drug shops for drugs for their animals?

B: do people go to medical shops or drug store to get their livestock medicines?

I: obviously, they do. But those who do business, they for their business purpose go outside to buy medicines, because they are afraid. But the poor ones cannot buy medicines from outside by paying money. So, they go to the quacks. Some people who are poor go to the quacks, but those who are afraid, the businessmen, they are in reality rich, so they go outside the village to buy medicines. They might go to Calcutta or Diamond harbour.

D: and why do they go outside of the village?

B: repeats

I: the medicines which will benefit their cattle are not available in the village. So, they go outside, if they go to Calcutta or Diamond harbour, all the medicines that they require are available.

D: Why doesn't that operate in the village?

B: the animal doctors that you have in here, why don't they operate?

I: they do operate. Whenever they are called in, they come by bikes. If one family calls in a doctor, quack, he will come in by bike and provide the medicines necessary.

D: why don't officials, like government, operate in here?

B: why don't government doctors who are not quacks operate in here?

I: it is for the money. They know that they won't be able to earn more money, so they don't come here. There is a hospital about two kilometres away; you can have a talk with the government appointed doctors over there.

B: the dispensary we are supposed to go to, in about five minutes distance from there, we have a hospital where they have government appointed doctors. We can talk to them.

D: are they human doctors?

B: do they treat humans?

I: both of men and livestock doctor.

D: is that in [village name redacted] village?

I: yes, this is within [village name redacted]. It is a large village; we have three high secondary schools here. You may also talk to the school teachers who can provide with the information that you need.

D: Do you know the names of the Vets? The official vets?

I: At this moment I can't call up. [*Name redacted (IP3)]* is much helpful. He has sent you to me for information. I know he can do special medicinal work on cattle.

D: Could you ask him if he understands the term antibiotics?

B: Sir, do you know about antibiotics?

I: Yes. I do. I have to use antibiotics. Doctors advise not to use too much antibiotics.

D: what do you know about antibiotics?

I: I have no medicinal knowledge about antibiotics. Since, I'm a heart patient I have to use antibiotics. Some of the medicines that they give me are antibiotics.

D: Do people use antibiotics with their livestock?

B: repeats.

I: Yes, they do. Some of the families who have cattle they use it. It is just that since they are illiterate they cannot communicate with you well enough.

D: Do they go for all the different kind of animals?

I: yes, they do. We don't have much time otherwise I would have called the ladies who keep these livestock. They know better than I do because they themselves go to the doctors and quacks to take the medicines.

D: Do you know the main reasons people use antibiotics for livestock?

B: do you know the real reasons behind the use of antibiotics on livestock?

I: I don't know exactly the reasons but I do know that they help lessen the pain of the patient. But too much use of antibiotics is not good for health. This is what I know, I do not know if it is correct or not. This is my simple knowledge. If you take in a lot of antibiotics, it'll be harmful for health.

D: Do you know which livestock system is most likely to use antibiotics?

B: In what kind of animals, do people usually use antibiotics?

I: Chickens, goats and in some cases for cows.

D: why are these more likely?

B: why is it so? Why are these animals given antibiotics?

I: Because doctors who are qualified and have degrees, they are not available here. That is the main reason. That is why instead of going to the doctors they go to the quacks. One thing I've overlooked. We have a Christian missionary here, which was set up by Mother Teresa. They have many doctors here. There are many qualified doctors here who come from U.S.A, London, Germany. They stay there and they treat the people, not only the people but also the cattle. You can go to this Christian Missionary which is also situated in [village name redacted]. If you go there and talk to the doctor they will give you much information.

D: Do the antibiotic providers give the medicines to the animals or do they give them to the keepers?

B: he is asking if the antibiotic providers give the medicine themselves or…

I: No, they provide the medicines as prescribed by the doctors. They give whatever the doctor prescribes, that may include antibiotics.

D: and do the people follow the advice well?

B: Do the keepers follow the advice?

I: Possibly they try to listen to this and follow this. Because it is connected with the welfare of their own cattle, so they try to follow the advice.

D: Are you aware of any rules regarding when antibiotics should be given?

I: I'm not a doctor so I cannot answer this question.

D: Are you aware of any situation where human antibiotics are given to the animals?

B: Do you recall any incident when a human antibiotic was given to any animals?

I: I shall give you an instance. Once a mad dog bit me, so I needed some medicines, antibiotics medicines. Doctor prescribed me five injections. I took those five injections. After about a year, a cat bit me. I was bleeding. I went to the doctor and told him about the incident. He gave me the medicines, but when I mentioned that I have already taken the medicine for the dog bite, he said that he would not give me any antibiotics anymore. He said it is for life long, once you've taken it you are not allowed to take it again. This is my own story.

B: he says that once you've taken the vaccine of antibiotics, you won't need it anymore in your life.

D: So, is there any instance when a human antibiotic is used on animals?

I: I have no knowledge about this. It is a rare case.

B: Do you recall any such incidents?

I: No, can't think of any.

D: do people generally know that there's a difference between human and animal's medicines?

B: Do the people who keep livestock know the difference between human and animal's medicines?

I: yes they know. Whenever the members of their family are suffering, they go to the qualified doctors. They do not go to the quacks. The doctors prescribe the medicine and they take them. But whenever their cattle suffer from diseases they go to the quacks. This is the difference.

D: why is there a difference?

I: it is about life security. When a member of the family suffers from a disease, it is of more importance than an animal suffering from a disease.

D: what do people generally think of medicine?

B: what idea do people with livestock have of medicine?

I: They have very little idea. They should go to qualified doctors. But like I've already told you they have financial problems, so they can't afford qualified doctors who are not that much available and they charge more.

D: how do you think the quacks and doctors perceive these medicines?

B: What kinds of medicines do these quacks or these doctors give?

I: They (quacks) use medicines which they collect from somewhere. I don't know where they collect the medicines from. But MBBS doctors who are qualified, who serve the local people one day or two day per week, prescribe medicines that are unquestionable. But you can't depend wholly on a quack's medicines; it may or may not yield results. Yet, the poor people go to the quacks for the financial difficulties.

D: And how do you think farms view medicines?

B: how do livestock farms view medicines?

I: I can't say much about that. How can I say this since I'm not connected with a farm? (Laughs) I shall take you to doctors who are my disciples; they shall give you all the answers you need. I have one student coming today; I shall know the names of the doctors from them and tell that to you. You can ask them the questions and they can answer.

D: Thank you so very much. I don't have any more questions.
