# Supplementary material for: “If It Works in People, Why Not Animals?”: A Qualitative Investigation of Antibiotic Use in Smallholder Livestock Settings in Rural West Bengal, India
Source: Antibiotics (Basel). 2021 Nov 23;10(12):1433. doi: 10.3390/antibiotics10121433 (PMC8698124; doi:10.3390/antibiotics10121433)
Supplement: Supplementary file 1 [file antibiotics-10-01433-s001.zip › Supplementary S1_ Interview Transcripts/Site 1/Key Informant 4 (site 1).pdf]

Code for Study - 'If it works in people, why not animals?': A qualitative investigation of antibiotic use in smallholder livestock settings in rural West Bengal, India: Key Informant 4, Site 1

Date: 18/07/2019

Location: Site 1

Interviewee: Key informant (Ex GP-chief)

Interviewer: Dominic Day (DD)

Translation: Somraj Das (SD)

Transcription: Sayak Manna (SM)

D: Interviewer (DD)

B: Translator (SD)

M: Interviewee (KI4)

START OF INTERVIEW

D: Thank you very much for listening to us!

B: Thank you for talking

D: Would you describe [village name redacted] village?

B: Can you describe [village name redacted] village?

M: [village name redacted] is cultivated area (he meant agricultural), most of the people are farmers and some are fishermen. They fish by the river and sustain themselves. This is what we are. As per the caste here most people are mohammedan (Muslim), people are Schedul caste and Schedule Tribes. These people lead a below poverty lifestyle.

B: What Sir is saying that in [village name redacted] maximum people are farmer associated to agriculture sector and many of them belong to the fisheries as well. If you talk about caste divisions, they're are mostly muslims. that's how he id defining [village name redacted]!

D: Ok, Great, could he explain how many people live in the village?

B: How many people stay in [village name redacted]?

M: In our [village name redacted] village near about 7000-8000 people, our subcentre had 5000 people, [village name redacted] mouja/village- 7000-8000 people.

B: [village name redacted] village has more than 7000-8000 people.

D: Okay and how many households?

B: How many houses, approximately?

M: Around 1500 houses!

B: Up to a1.5K.

D: Ok. Great. How would you describe the wealth of the village?

B: What's your village's property? What do you think? Has it increased or decreased? Any improvement or it decline (Question is twisted)

M: Is there any Improvement? Umm.. in agricultural sector there's some, initially there wasn't much water for irrigation, now we get some. The lives of fishermen haven't improved alot but yes there're mechanised boat and stuff that are provided by the government, which of course is of great help. Now they do fishing with some developed technologies. There are improvements but partly.

B: Sir is saying, that the wealth..the way he defined is...he's talking about the agriculture, few years back farmers were in much water crisis..irrigation?

D: Okay.

B: Now, the irrigation department has developed, farmers receiving plenty of water (NOT PLENTY) for agriculture to harvest something. He's just mentioned about the fishermen, nowadays government are providing new technology and boats for fisheries. This is how he's defining the total wealth of the village! Increasing the per capita income.

D: Ok , so which type of livestock are kept in [village name redacted] village?

B: What are the type of livestock the people of [village name redacted] keep? When everything is considered, types, kinds etc!

M: Mostly cow, goat, hen, duck.

Wife: Birds.

B: Birds don't come under livestock!

M: You can't say birds. Cows, goats, ducks and hens.

B: Mainly?

M: yes.

B: So sir is saying that cow duck chicken turkey, that's it. Goat!

D: Good, so is this all of the animals that are kept here?

B: So all these animals are kept as livestock here?

M: Yes!

B: Cow, goat, hen, duck and sheep!! By sheep I mean goat (Really?)

M: Well sheep are there but very less.

B: Sheep are less.

D: Sheep are (..?)

B: Sheep are too less in quantity.

D: How many households own each of these animals?

B: In your [village name redacted] how many families keep livestock?

Wife: At our place?

M: Not only our place, but entire [village name redacted]. Well the [village name redacted] is quite a big village, it is divided into 3 parts. [village name redacted] North, [village name redacted] Middle and [village name redacted] south. We are in south. The population I talked about was in total, i.e. 7000-8000. So, around 2000 families for sure, the ones who keep livestock.

B: sir is saying that more than 2000 households are keep livestock. And the village is too big, it's segmented in 3 divisions. We are in the south. There's north..

D: What's it?

B: North, south and another one is?

M: Middle.

B: North [village name redacted], Central [village name redacted] and South [village name redacted]. Right now we are in South [village name redacted] and around 2000 households keep livestock.

D: Is there more livestock keeping in any of these sections of [village name redacted]?

B: Apart from this south, if you go to North and Central [village name redacted], are there people who keep livestock too?

M: Yes, there are. Every village has some amount of livestock.

B: Sir is saying, you can find more or less livestock. If you are going to see there, they are much more quantity always than the quantity you expected as livestock. (What's the meaning of the sentence?)

D: An which animal would you say is most commonly kept in [village name redacted]?

B: Sir, the names of the animals you just took, out of them which animals is mostly kept?

M: Cow

B: Khau!

D: And do people keep them for commercial reasons?

B: Do you keep cows for commercial reasons too? I meant, do you get any financial benefit from them?

Wife: Yes

M: We use them mostly in agriculture, they give milk. Yes of course business is there!

B: Sir is saying that, they sometimes keep livestock for agricultural purpose like ploughing the total area with the bullock..one thing and they provide milk as well. So they keep livestocks for some kind of financila reasons as well.

D: Do people keep them for personal use or for selling?

B: The livestock you keep here are they only for personal use or you sell them too?

M: Yes, sometimes we do sell. We usually keep them for personal use. But we sell some of them as and when required.

B: Sir is saying, it depends. Most of the people..family keep them for personal use but when it comes that it's necessary to sell the livestocks, they would and they do.

D: ok, are there any large farms in [village name redacted]?

B: Do you have large farms? Where the livestocks are kept in large numbers?

M: No.

B: No

D: Okay. do people farm fish or.. okay.

B: Do you farm fish?

M: Yes there are some which are done individually. Most the houses have personal ponds, they do fish farming in those ponds. But fish farming in large scale i.e. in Lakes and large ponds doesn't happen here much.

B: Sir is saying most people have personal ponds, so they can maintain fishery as personal use, but there is no large quantity of commercial uses for fisheries.

D: Okay, Great. So what are the main reasons for keeping livestock?

B: Why are the major reasons to keep livestock?

M: There are totally two sides that are maintained. One is family. Suppose cow, if we say Rai cow (Here Rai is Radha a female Character from Hindu Mythlogy) we get milk from them, we drink some and the excess is sold. Fish is grown in ponds, We catch them for our daily use, we don't need to buy them and again the excess is sold time to time in the market. So we have two sides.

B: As he's saying, they have to maintain 2 aspects one is for personal consumption, when there's a surplus in the livestock population they usually sell them, such as they keep cows as livestock. Cows provide milk, sometimes for agriculture. Sometimes they keep chicken and they provide eggs. And when it's economically viable they ought to sell it. That's the thing.

D: Is that the scenario for each of these different animals that are kept?

B: The names of the animals you took, what do you think, for all of them the scenario is same?

M: Yes.

B: Yes, they all have same situation in commercial and personal (..?)

D: Ok. Umm.. which person in the household normally owns the livestock?

B: In a family who is the one who owns the livestock? Who buys and sells?

M: In that case I have to say, for hens and ducks, the wives of the houses are the main supervisors. For cows the head is males. The males of the houses takes care of cows and goats and women take care of hens and ducks, sometimes goats too. In our area the poor mothers take care of goats, hens and ducks. They sell their eggs and maintain their families!

B: He's saying it depends. Just like that if you keep chickens as livestock the women of the family take care of that. So you can call women of the house as owners. And suppose you are keeping cows as livestock, so men of the family take care of cows. So it depends. And there is one thing he just mentioned. there are many poor women in [village name redacted] who generally keep chicken as livestock and sell their eggs to maintain their family. so there is a financial way.

D: Ok great! thank you. So to your knowledge how are these animals normally kept?

B: Depending on your knowledge how are these animals normally kept?

M: We normally keep them in a shade. In villages keeping animals in high technology ways is not a thing. So keeping them in great health is not there. When I took the diary training, I remember we were told that make shade in north south direction so that, in the morning the heat of the rising sun in the east would be beneficial, it will be hygenic. And same in the evening. These were told to us. Maintaining in such manner isn't much possible. We maintain as per our abilities!

B: Well according to his knowledge, people somehow manage by their own techniques. If you roam in [village name redacted] you will not see any advance technology to keep them. Sir many years ago got trained about diary products (NOT PRODUCTS). He was trained to keep the milk (Milk wasn't mentioned) in a way, the sun rays will make the entire milk hygenic and at the end of the day in the evening when sun goes to the west, then remove (Keep the milk) towards that so...what's he saying that these kind of technologies can't be maintained in [village name redacted]! So people are managing somehow according to their knowledge and limitations..

D: Ok, and are they normally kept in the household with the family?

B: So the families are staying together also livestock are staying together, is it how things are?

M: In a family- ducks, hens, cows are everybody's property.

B: Sir is saying, yes it happens sometimes in some kind of households, you will see, they are keeping their livestock over there and keeping their family members there. So it happens here.

D: Umm okay, so how do people learn to keep livestock?

B: How do people learn about livestock, like what to do and what not to do. How do they get their knowledge on this?

M: How to say..umm..this...

Someone: This is passed on to generations.

M: They learn it from the family, from the villagers. There isn't any training specifically. There's no such chances.

B: He's saying it's all inherited, the knowledge transfered from your father to the son..your father got it from your grandfather. So there's no particular session or manual that these people usually follow to keep their livestock.

D: hmm.. okay, thank you, Can you say the next question is about health care providers.

B: Can anyone here give us information about health care clinic? Or someone who keeps knowledge about healthcare clinics, we want to question on them. You know, hence we are asking you these questions.

D: So, what do people do when animals become sick?

B: What do the people of this village exactly do when their livestock is sick?

M: Recently in the gram panchayat level (Rural Government level), there is a vet healthcare facility. There is one assistant who helps those who go to him. Or else they go to village homeopathy doctors or old experienced people who have their own measures to take care of things. Suppose you, who talked about your family... we knew when a cow had an ulcer in its leg, we have seen our fathers to take the cow dung, mix it in water, boil it and then apply in the ulcer. And that worked. Even the cows have some disease in their throat region called Eso, small gall like eruptions. Just like we have ulcers in our throat and tongue after catching cold. We have seen our fathers applying fig leaves and salt in the tongues of the cows by rubbing them together! We don't have any of those, now we have homeopathy doctors who treat them or we go the assistant who sits at the veterinary clinic, per block we have a surgeon. So there's 1 Veterinarian in 1 block area! These are the ways how it work. I won't say we have a great deal of things to work with. It's very neglected! This is all I can say!

B: What's sir's saying, that there is a GP/gram panchayat block called the health department. I have just told you about the last guy who gets his livestocks to the same person, he is an assistant doctor in GP/gram panchayat and people get their livestock there, when their livestock get sick. Sir is saying something else. In his childhood, just like we have cough and cold and we have swollen throat, as a human being we find difficulty to swallow something, in livestock, particularly in cow, they have the same thing, so they had some kind of leaf, leaf with salt and grab the cow's tongue out of.. and they are going to grab it, so it's going to work. And the entire concept is neglected. Sir is saying so

D: How (..?)

B: Well there's a one single guy who is a doctor or practitioner and there's more than 2000 family is coming. Sir's saying infrastructure is neglected!.

D: Okay. Does every different type of livestock are all treated by the same person?

B: The livestock you named, do they all go to the same person to get treated?

M: Yes..umm no, homeopathy doctors are there in every area. These homeopathy doctors are not qualified doctors. Work practitioner. They treat on the basis of whatever they know. And what I said about the health assistant per gram panchayat, it's not only for 8000 population. 30,000 people are dependent on one. I mean the total [village name redacted] GP, there are near about 30,000 people, for them there's only 1 arrangement.

B: Sir is saying that, it's not that, there is a homeopathic practitioner, called quacks because they are not academically qualified to practice, still they do..one thing...and GP of [village name redacted] is more than 8000 but there are a total population of [village name redacted] is 30,000 and 1 single health infrastructure is built for this entire population. 1 single practitioner per single district gram panchayat over 30,000 population, that's the thing he just mentioned!

D: Yeah! So do people often seek the care of the quack?

B: You mean seeking the care of the quacks?

D: Seeking the care of the quacks.

B: Come again I didn't get you.

D: uhh.. do people often ask the quacks for help with the animals?

B: Sir, the livestock keepers often go to quacks to seek advice? (Changed the question)

M: Yes

B: Yes

D: Yes. uhh..what are the reasons for going to these healthcare providers?

B: What do you think? Why do they go to them?

D: Is that the scenario for each of these different animals that are kept?

B: The names of the animals you took, what do you think, for all of them the scenario is same?

M: Yes.

B: Yes, they all have same situation in commercial and personal (..?)

D: Ok. Umm.. which person in the household normally owns the livestock?

B: In a family who is the one who owns the livestock? Who buys and sells?

M: In that case I have to say, for hens and ducks, the wives of the houses are the main supervisors. For cows the head is males. The males of the houses takes care of cows and goats and women take care of hens and ducks, sometimes goats too. In our area the poor mothers take care of goats, hens and ducks. They sell their eggs and maintain their families!

B: He's saying it depends. Just like that if you keep chickens as livestock the women of the family take care of that. So you can call women of the house as owners. And suppose you are keeping cows as livestock, so men of the family take care of cows. So it depends. And there is one thing he just

mentioned. There are many poor women in [village name redacted] who generally keep chicken as livestock and sell their eggs to maintain their family. so there is a financial way.

D: Ok great! thank you. So to your knowledge how are these animals normally kept?

B: Depending on your knowledge how are these animals normally kept?

M: We normally keep them in a shade. In villages keeping animals in high technology ways is not a thing. So keeping them in great health is not there. When I took the diary training, I remember we were told that make shade in north south direction so that, in the morning the heat of the rising sun in the east would be beneficial, it will be hygienic. And same in the evening. These were told to us. Maintaining in such manner isn't much possible. We maintain as per our abilities!

B: Well according to his knowledge, people somehow manage by their own techniques. If you roam in [village name redacted] you will not see any advance technology to keep them. Sir many years ago got trained about dairy products. He was trained to keep the milk in a way, the sun rays will make the entire milk hygienic and at the end of the day in the evening when sun goes to the west, then remove (Keep the milk) towards that so...what's he saying that these kind of technologies can't be maintained in [village name redacted]! So people are managing somehow according to their knowledge and limitations..

D: Ok, and are they normally kept in the household with the family?

B: So the families are staying together also livestock are staying together, is it how things are?

M: In a family- ducks, hens, cows are everybody's property. (The interviewee didn't understand the question, because it wasn't done in a right way).

B: Sir is saying, yes it happens sometimes in some kind of households, you will see, they are keeping their livestock over there and keeping their family members there. So it happens here.

D: Umm okay, so how do people learn to keep livestock?

B: How do people learn about livestock, like what to do and what not to do. How do they get their knowledge on this?

M: How to say..umm..this...

Someone: This is passed on to generations.

M: They learn it from the family, from the villagers. There isn't any training specifically. There's no such chances.

B: He's saying it's all inherited, the knowledge transfered from your father to the son..your father got it from your grandfather. So there's no particular session or manual that these people usually follow to keep their livestock.

D: hmm.. okay, thank you, Can you say the next question is about health care providers.

B: Can anyone here give us information about health care clinic? Or someone who keeps knowledge about healthcare clinics, we want to question on them. You know, hence we are asking you these questions.

D: So, what do people do when animals become sick?

B: What do the people of this village exactly do when their livestock is sick?

M: Recently in the gram panchayat level (Rural Government level), there is a vet healthcare facility. There is one assistant who helps those who go to him. Or else they go to village homeopathy doctors or old experienced people who have their own measures to take care of things. Suppose you, who talked about your family (about Benjamins parents I believe)... we knew when a cow had an ulcer in its leg, we have seen our fathers to take the cow dung, mix it in water, boil it and then apply in the ulcer. And that worked. Even the cows have some disease in their throat region called Eso, small gall like eruptions. Just like we have ulcers in our throat and tongue after catching cold. We have seen our fathers applying fig leaves and salt in the tongues of the cows by rubbing them together! We don't have any of those, now we have homeopathy doctors who treat them or we go the assistant who sits at the vetenary clinic, per block we have a surgeon. So there's 1 VS in 1 block area! These are the ways how it work. I won't say we have a great deal of things to work with. It's very neglected! This is all I can say!

B: What's sir's saying, that there is a GP/gram panchayat block called the health department (NO!). I have just told you about the last guy who gets his livestock to the same person, he is an assistant doctor in GP/gram panchayat and people get their livestock there, when their livestock get sick. Sir is saying something else. In his childhood, just like we have cough and cold and we have swollen throat, as a human being we find difficulty to swallow something, in livestock, particularly in cow, they have the same thing, so they had some kind of leaf, leaf with salt and grab the cow's tongue out of.. and they are going to grab it, so it's going to work. And the entire concept is neglected. Sir is saying so (SPOKE RUBBISH)

D: How (..?)

B: Well there's a one single guy who is a doctor or practitioner and there's more than 2000 family is coming. Sir's saying infrastructure is neglected!.

D: Okay. Does every different type of livestock are all treated by the same person?

B: The livestock you named, do they all go to the same person to get treated?

M: Yes..umm no, homeopathy doctors are there in every area. These homeopathy doctors are not qualified doctors. Work practitioner. They treat on the basis of whatever they know. And what I said about the health assistant per gram panchayat, it's not only for 8000 population. 30,000 people are dependent on one. I mean the total [village name redacted] GP, there are near about 30,000 people, for them there's only 1 arrangement.

B: Sir is saying that, it's not that, there is a homeopathic practitioner, called quacks (NO, wrong concept) because they are not academically qualified to practice, still they do..one thing...and GP of [village name redacted] is more than 8000 but there are a total population of [village name redacted] is 30,000 and 1 single health infrastructure is built for this entire population. 1 single practitioner per single district gram panchayat over 30,000 population, that's the thing he just mentioned!

D: Yeah! So do people often seek the care of the quacks?

B: You mean seeking the care of the quacks?

D: Seeking the care of the quacks.

B: Come again I didn't get you.

D: uhh.. do people often ask the quacks for help with the animals?

B: Sir, the livestock keepers often go to quacks to seek advice? (Changed the question)

M: Yes

B: Yes

D: Yes. uhh..what are the reasons for going to these housecare providers?

B: What do you think? Why do they go to them?

B: Sir he's saying that the unavailability of the practitioner guy, he comes around 10-11 and wander off by 4, this is the main thing. He is saying that there's a diamond harbour constituency consisting of 18 major areas. There's a one single major health infrastructure (..?) So unavailability of the proper qualified guy who is a practitioner as well. (?)

D: Okay ah..do any of these quacks work in [village name redacted] village?

B: Are there quacks in [village name redacted] village or no?

M: Quack doctor?

B: Yes there are.

D: How many?

B: How many of them?

M: 5 or 7 of them.

B: 5 to 7

D: 5-7! Could you ask him if the end of the interview if we got their names and may their numbers?

B: Will there be a problem if you will give us their names and numbers? Sources won't be disclosed!

M: Where I will get their phone numbers?

B: Only names will do too.

M: Name..the ones who practice homeopathy..

B: 5 or 6 of them in [village name redacted]!

M: One is here [name redacted]

B: Write down the name. He is quack…[name redacted].

M: He's a primary teacher and he does that..

B: He's a primary teacher cum practitioner. Where he could be found?

B: Where he can be found?

M: Near that crossing.

B: He practices next to that alley.

D: Okay, So do people tend to ask advice regarding the livestock?

B: Do people come to take advice from you regarding the livestock?

M: No! I don't have much knowledge about it so don't know.

B: No, he doesn't have much experience and knowledge about livestock.

D: I didn't mean to him.. I mean.. just do people generally ask anyone for advice?

B: People who keep livestock do they take advice from others?

M: Yes!

B: The ones who are not doctors.

M: yes the ones who are not doctors but practicing for a long time, they must've taken information from seniors.

B: Sir is saying yes they do, just because the other guy has much more experience in keeping the livestock.

D: Do any of these people provide medicines?

B: What do you think, the one to whom people go to for advice, do they recommend medicines too?

M: No, they don't know about meds. But suppose I cured my cow using certain meds from a particular disease I do tell may be this medicine can work. Usually where will they get meds? But they may say, " I used this and cured it so you can try using it too" that's it!

B: Sir is saying max time, it doesn't happen but sometimes if you have much more experience than me, then you will say I've tried this med you can try that as well, in such-n-such situations. If that situation develops.

D: Ok, And where would people get the meds from?

B: Where do these meds come from? (He changed the question!)

M: They get them from the medicine shops. Whatever idea I have, I think these meds are meant for human use and they use them here, as far as I've heard! The homeopathy meds are given to humans and are also given by them too. How much do they give it's known to them. In certain cases for cows and goats those meds are given. There're special meds which are exclusively meant for animals and are found in Diamond harbour. There are couple of shops there, so when they write/prescribe, the doctors.. people go there and purchase them. That's a rare thing.

B: Sir is saying, sometimes it happens, particularly in homeopath practicing, if anyone is homeopathy practitioner and making medicine for human can sometime tries on livestock. But it's a rare case but it happens. But most of the cases they acquire meds from local markets (..?) etc.

D: Okay, Is the homeopaths different to the quacks? Is he using different works for them?

B: The homeopathy quacks, are they all different from each other?

M: yes they practice individually. This is their personal business and source of income.

B: He's saying that 1 homeopathy practitioner is individual practitioner comparing another individual practitioner in homeopathy!

D: Oky, sure. Could you ask him if these 4 groups, if there's anyone else who might provide antibiotics?

B: Sir you just talk about the 4 places, the ones who give you meds. 1 is GP doctors (Gram Panchayat), 1 is quacks, 1 is homeopathy and another is local medicine shops, is there any way you can get the meds?

M: No, these quack practitioners are these guys.

B: He's saying quacks and homeopathy practitioners are same

D: Same? Ok, sure.

B: And there are no other ways to acquire meds other than these 4.

D: Ok.

M: I used to see chickens are having diarrhoea, the women of our locality used to go to the local allopathy medical store and bought tetracycline medicines! This is what doctors suggested. They used to buy in bulks and gave to the birds. These were known to them, they learnt it!

B: He's just giving us an information casually, that when a chicken facing a dysentery problem, the women of the house go to the allopathy med shops asking for tetracycline. And it worked, without consulting with the doctors!

M: May be the doctor told them before.

B: That is the thing, you may have consulted the doctor 6 months ago. The doctor prescribed you a med before 6 months now after 6 months you're facing the same problem, so without consulting you are using the same med.

D: Ok.

Wife : (not clear)

B: Well she's talking about the general longivity of the adult chicken that last for 1 year.

D: Ok, so do the human medicine providers are aware that the meds are used in livestock?

B: The ones who are giving meds for humans are they aware that these meds are used on livestock?

M: No idea.

D: No idea, ok. Would people...Who would people go to for medical advice regarding their livestock?

B: Whom do people go to in this area for medical consultation regarding the livestock?

M: I told you, to the village homeopathy doctors, they go to him and talk about the problem of their goat, the doctor gave them the med what is known to them. Or they can go to Panchayat office's animal health assistant, he might also give some advice!

B: Sometime to quacks homeopathy, sometime to GP practitioner and to allopathy.

D: Ok. Do people often go outside of the village for this?

B: For these advices do the people keeping livestock go outside village?

M: rarely when there's some critical problem. If it's serious, they might go to that Diamond harbour animal hospital.

B: It rarely happens. if you do not have that kind of extreme emergency, people don't want to go out of the village.

D: And why is that?

B: Why is it that they have to go outside?

M: If they can't cover. May be the meds they gave for the disease to control didn't work. Then they have to take them outside.

B: Sir is saying the local infrastructure is not able to handle or deal with the problem, so people have to go outside the village in emergency situation.

D: umm, can you ask him, if he is aware of the term, Antibiotics?

B: Sir, do you know what antibiotics are?

M: I know a bit.

B: Yes , he knows.

D: Okay, so could he tell me if AB are used in all these different livestocks.

B: Which AB are used in which livestock do you know that? (Question changed)

M: Na.

B: He doesn't have the technical idea about that! (Because he asked the wrong question)

D: Okay, do people use general medicines?

B: Can you tell which medicines are used when these livestock animals are sick?

M: No. No idea, I never take them, so no idea. But as I said the story of tetracycline. I heard that from local hospital that everybody is getting tetracycline. Why? Because the chickens are sick.

B: He doesn't know, he doesn’t have the technical idea about medicines, which medicines are applied in which kind of livestock. The only story he knows is the tetracycline one.

D: Ok. Does he know, if medicines are administered..do the owners or do the medicine providers give it.

B: Sir where do these medicines come from? Who takes care of these? Where do the livestock owners get these medicines? Who gives them?

M: The owner of the med shops, the pharmacists, the people associated with the medical structure.

B: Well, he's talking about the pharmacists and local retailers. They do not administer. (He is just making up the questions and answers!)

D: So, the livestock owner administer? Ok, he's probably not be knowing that. Other than tetracycline story does he know human meds used in livestock?

B: Apart from the story of tetracycline, are there any stories you can share. Where human meds are given to livestock?

M: I can't say specifically, but whatever little I know, the meds given my homeopaths are human medicines only! That's all I heard from the market!

B: (explained the same)

D: Ok, and the other way round? Animal ones are used in humans?

B: have you heard the reverse case whether humans are given meds of livestock?

M: No

D: That's fine, umm..so does he think that people see a difference between human and animal meds?

B: The livestock keepers around here do they know the difference between the meds of humans and meds of animals?

M: No.

D: How do households perceive meds?

B: The meds you buy for livestock, how do you buy them or store them?

M: Mainly when the requirement to buy meds happen, they go to the health assistant of GP office, he advices which meds to buy, he writes them, he tells them from which place at Diamond harbour to go and purchase the meds?

B: (gave a twisted yet close enough answer)

D: Okay and is it common to use meds?

B: The meds that are in use, is it too common to use them? Like on regular basis?

M: No no, only when there's a problem then, else not on regular basis. Nobody is that much bothered or careful about cows and goats! For an instance I give my own child horlicks (a nutrient supplement), not like that! When there is a disease and doctor prescribes something, then only they buy meds. We don't take care of our livestock to the degree we should, not that properly at all. The goats and cows in our

locality suddenly die, yesterday I heard some's cow died, at night the animal was in excruciating stomach pain, was jumping around and then died at night!

Wife: They fed them boiled bulgur, so gas developed in the stomach, the cow cried out the whole night and was in pain then died.

M: Excessive bulgur wheat was fed and so it died. This is all because of our lack of knowledge!

Wife: To get more milk, they fed it in excess. Because they sustain on the basis of selling milk.

B: He's saying not like that, if something happens, they do look for consultations. He feeds his child nutritional product to keep his child healthy. People don't do such things to livestock. D: And they use med for any other reason?

B: Do you use meds for other reasons other than the sickness?

M: No no! The ones who have cattle shades they might do it (I think he meant cattle business) I heard that. To get more milk, to have better health, they might use. The ones who have shades! In [village name redacted] GP, there are couple of shades.

B: (explained the same)

D: But he said It never happens in [village name redacted]?

B: It never happens in [village name redacted]?

M: In [village name redacted] village this doesn't happen but it's there in GP.

B: It happens in GP but not exactly here in [village name redacted]!

D: Ok.. all forms of animals go to all forms of these medical providers?

B: So you are saying the types of livestock go to all types of medical providers?

M: Yes!

D: Now we would want to be introduced to quacks and livestock keepers if he is aware of any, the names?

M: I know a few like *Name redacted (LK14)* who sits in near North [village name redacted]. The ones who does homeopathy people go to them. [name redacted] is in South and no one as such in central.

D: Name and number of livestock keeper and would be happy to share?

Wife: (Not understandable)

M: *Name redacted*.
