# Supplementary material for: “If It Works in People, Why Not Animals?”: A Qualitative Investigation of Antibiotic Use in Smallholder Livestock Settings in Rural West Bengal, India
Source: Antibiotics (Basel). 2021 Nov 23;10(12):1433. doi: 10.3390/antibiotics10121433 (PMC8698124; doi:10.3390/antibiotics10121433)
Supplement: Supplementary file 1 [file antibiotics-10-01433-s001.zip › Supplementary S1_ Interview Transcripts/Site 1/LK 1 (site 1).pdf]

Code for Study - ‘If it works in people, why not animals?’: A qualitative investigation of antibiotic use in smallholder livestock settings in rural West Bengal, India: LK1, Site 1
Date: 01/07/2019
Location: Site 1
Interviewee: Livestock Keeper (LK)
Interviewer: Jean-Christophe Arnold (J-CA)
Transcription: Debanjan Debnath (DD)

I: Interviewer (JCA)
P: Participant (LK1)

START OF INTERVIEW

I: What sort of animals do you keep in the household?

P: At first, we had "desi" cow. Then using injections, since bulls aren't there any longer, it's happening. Jersey, Punjab, Mini Jersey, as such. It was 35 Rupees before, now at the "Anchal" it's 40 Rupees for the injection. And for the doctor who visits the houses takes 300 Rupees, before he used to take 200 rupees now, he takes 300 rupees. So (...) he takes 300 Rupees, and we tell him that we are poor and urge him to take a bit less, he takes 250 Rupees now. Then if the cow has worms, he takes 30 Rupees, for "Kent" we put this and that. Sometimes we put the medicine on the cows, sometimes we feed it to them.

I: What animals do you have now?

P: We have Jersey, Punjab.

I: How many (...)?

P: (Interrupting) we have four. There's one Punjab calf. It's one year old. There's one "desi" cow. There's a Jersey cow. There's a big Jersey cow. It gives around 2.5 to 3 Seer milk. And the local breed cow gives around 1 Seer milk. Between the two calves one is Punjab and the other is Jersey. One calf is this big Punjab, and the Jersey is also another calf 1.5 year old. One is 1.5 years old the other is 1.

I: Apart from cows do you have other (animals)?

P: We have ducks. Four ducks. We have two drakes and "degi" (ducks).

I: What do you mean by "degi"?

P: I mean, the female that lays eggs (laughs).

I: How many male and female did you mention?

P: I have one son and one daughter.

I: Oh, no, I mean... (...)

P: Oh, the ducks? Two males, and two females.

I: Who owns the animals?

P: We both are the owners. We raise them, we are the owners.

I: Why do you keep cows?

P: We keep the cows because we suffer deprivation in the family, keeping the cows make a little money. We might sell them for some money. or when we can't bear the expenses of the family, we might sell the milk. This is why we keep the cows, no other reason. It's hard work, but it's an addiction for us, we can't live without animals.

P2: Time passes by, managing the cows. Like the cows just has been put out by us, I came back she (my wife) gave them water and put them under a tree, now I will go to the school to sell chickpeas, on coming back from the school we will go there again.

P: To improve the condition of the family, we work very hard. So that our family doesn't have to suffer. So that we can eat a handful of food. This is how we strive.

I: What do you get from the cows?

P: We get the cow dung, the faeces of the cow: we thump it to get fuel. And when it's time for the cow to give milk, the milkman comes, milks the cows himself and takes it. He writes down the amount of milk, for example, 2 Seer/3 Seer/2.5 Seer. At the end of the month the total is calculated, and we get something like a 1000 Rupees or 2000 Rupees. We get this income.

I: What about the ducks?

P: The ducks, we feed them rice powder and let them go into the water and eat snails, fritters. Then they lay eggs at night. What you call "anda" (laughs).

I: I know eggs. (dim)

P: We sell the eggs, and we do buy things for ourselves. Sometimes we eat it. We offer it to the guests.

I: Do you keep the milk or eggs for yourself?

P: Yes, enough for us to consume. And the rest we sell. He (pointing to her husband) has grown old. He has high blood pressure. He no longer wants to drink milk, and he has gas. Ma (the mother-in-law) doesn't like milk, she can't stand the smell. It's just me and my grandson who drinks milk. My son and daughter-in-law they have gone to their home. They also drink it.

I: What do you feed the animals?
P: Now there's a lot of grass in the field, they eat that we don't give anything else. And rice water we get it from our neighbors, they (the cows) have it and sleep through the night, in the monsoon we cut the grass, we mix it with the straw, and feed it to them.

I: What about the ducks?

P: There's rice water. We mix it with powder. They eat and go to the water, we feed them in the afternoon, in the morning. We also give them the spare rice.

I: Do you give them anything to make them grow?

P: To help them grow (...) we can't afford it. That we will give them Injections and protein rich food, we can't afford it, grass is all they have got. We can't afford to give them protein rich food (to make more money).

I: Where do you keep the animals?

P: There's this big cowshed, we keep them there. Where "ma" *[mother-in-law]* is cooking, there's a big cowshed, floored in bricks, at the side. Do you want to go and see it? (laughs)

I: I will go after the interview? Is that okay? (resumes) where do the ducks stay?

P: There's a shack for ducks built with bricks. There are jackals and foxes, that's why they are made like this. Once they take them, we can't get it back.

I: Who takes care of the animal in the house?

P: We all take care of the animals. Because not one person can possibly do everything, Ma (her mother-in-law), him (her husband), myself, when my son and daughter-in-law is here, they also take care of the animals. We all look after them.

I: Does any specific family member have any specific job? (for example, you might have something to do for the animals and your husband might have something else to do (...)

P: No. It's not like I won't do this, He won't do that. We all serve the animals.

I: Does anyone outside the household help you with the animals?

P: No.

I: ...To look after them?

P: No one from outside help us. What does the "Anchal" give us? Nothing. When we see the cow has a bloated stomach, or has diarrhea, we go for the deworming medicines, they give that, nothing else. "Anchal" does nothing for us. We do everything, seeing the doctor etc. on our own money. No one helps us.

I: In the way you look after the animals in the house, does anyone else outside your house help you with it? Do you employ other people? Or maybe your neighbors?

P: No, just the family members. We can't afford a servant. We ourselves do everything (laughs).

I: How did you learn how to look after the animals?

P: We have observed and learnt through experience. After we get the animals how to bathe the animals, how to give medicines to them… we have been raising animals for generations... we learn it from them.

I: Have you learnt anything from other sources?

P: No, no!

I: We are going to the next section.

P: Please go ahead.

I: We are going to ask you about how medicines are used.

P: How medicines are used (...) we use deworming medicines. When we see the cow isn't eating, or has a fever, looks tired, we check by the ears, they get fever in their ears, we see that the ears are really hot, then we know the cow has fever. Then I tell him (her husband) that the cow has fever. Then he goes and calls the doctor. We have the phone number of the doctor. We call him. He comes and sees the cow and gives injections. After that how we're supposed to feed the medicines on time(...) we take a bottle and we put it in their mouth to feed them.

I: Who is this doctor?

P: The name of the doctor is [name removed- animal development volunteer]. He used to come to the "Anchal", but he doesn't come there anymore.

I: What do you mean by "Anchal"?

P: Anchal is government (...)

I: Do you mean "Panchayat"?

P: Yes.

I: Okay, Grampanchayat.

P: Yes, Gram Panchayat. They used to come there. But now, you see, every year the doctors get fired, now he treats animals privately. We have his number; we call him and he comes.

I: Does he have a degree?

P: Yes. We have his number.

I: After the interview we will take his number so that we can talk to him as well. What do you normally do when the cows get sick?

P: We contact the doctor. Now, when we see the cow is unwell, then we don't let them get wet we keep them inside the cowshed. If we see that the cow is not getting well, we call the doctor.

I: Do you contact anybody else apart from the doctor?

P: Apart from the doctor, we contact the "Anchal" office. When we are unable to contact anybody, we use sapota, etc. herbs and crush them and feed it to the animals. If they have diarrhea, they get better. we use these home remedies sometimes.

I: When you go to the GP, who treats the animals?

P: The doctor.

P2: Sometimes they would organize camps, but now they haven't done it for 2 years.

I: Do you know what the doctor gives you for the cow?

P: The doctor would perhaps give the cows vitamins. When they have diarrhea, they would treat them accordingly. When they have a cold, they give a syrup. They give all this in the camp, that's all. Nothing else.

I: Are you talking about the camp?

P: Yes, Yes.

I: When you take the cow to the GP what does the doctor give you for the cows?

P: The same thing, they don't provide us with anything else! They only give us something when we ask them, otherwise they won't. They would probably say, "I'm prescribing the medicines, you get them." We don't get any real help. We have to buy everything. When the cows have fever, and we take them there, we have to pay there as well. They don't give it for us for me, before they would give it for free.

I: When the doctor prescribes the medicines to you and you buy them, what do you do next?

P: After they get well, we don't go anymore.

I: After you get the medicine from the pharmacy, what do you do with the medicine?

P: We feed it the way the way we are asked.

I: Do you feed it yourselves?

P: Yes. Then if the cow gets better, we don't go anymore. What will we go for if the cow gets better? We don't go to see him anymore. The cow got sick in the monsoon. We went to the doctor, after they got well, we didn't take them to the doctors after that.

I: Have you ever heard of the word antibiotics?

P: No.

I: Do you have any medicine in your house? Medicines or medicine packets?

P: No, it's been a long time since we got the medicines.

I: Not even the covers?

P: We threw them away.

I: Where do you get the medicine from?

P: We buy the medicine from [name removed- animal development volunteer] Doctor. When we call him, he comes and gives everything and takes the money. When the doctor from the "Anchal" prescribes medicines, we make the call and he comes and give us medicines.

I: Who prescribes the medicines?

P: Doctor.

I: The GP doctor?

P: Yes.

I: Then who gives the medicines?

P: The other doctor.

I: Other doctor from the same office?

P: Yes.

I: Do you get the medicines from the same clinic as the one you go to get the treatment?

P: Yes.

I: Have you ever gone to get medicines directly?

P: No.

I: What do you do when the ducks get sick?

P: When the ducks get sick, they faint and die. We don't get medicines for them. We don't know. And we don't get.

I: Why?

P: When we see their heads are turning, they are sick, they are not eating, they would eventually starve to death. Where will get their medicines? Who will give them? They don't have medicines for the ducks and the chicken from the "Anchal". They will ask, "How many animals do you have." If we say 10-12, they say, "we don't have it.". They say it to our faces. If you have 50-100 animals they would, then, give the medicines. For less than that they won't open a file of medicines.

I: Have you ever tried to give medicines yourselves?

P: No.

I: The medicines that were given to the cows, do you remember the names?

P: No, I don't remember the names. It's been a long time since we used them.

I: When was the last time since they treated cows?

P: It's been two years.

I: At what severity of sickness you go to see the doctor?

P: When we see that the cow is very tired and can't walk. the cow is shivering (makes a gesture). Then we go to the doctor.

I: How do you understand when you stop treatment?

P: When we see that after having the medicines the cow has begun to eat and it is standing up, and walking. Then we understand the cow is healthy. We keep treating the cows until they get well, once they get well, we stop the medicines.

I: When do you treat the cows with home remedies (using fruits etc.)?

P: In the evening.

I: What I mean is, for what sort of illnesses?

P: When I see that the cow isn't eating in the evening. They stay in the field the entire day; we don't notice what it's doing. We go put them in the field and come back quickly. When we put the cows back in the cowshed in the evening, the cow, we know, is supposed to eat like crazy, not eating, then we examine it by the ear and run our palms through the back. If the cow isn't eating there must be some sort of a problem. If we see the stomach is bloated, we thump the stomach, it makes a "thud thud" sound. Then we understand it has gas. We sometimes use some indigenous remedies. We make them close their eyes and throw "idur mati" (the soil that's been dug out my rats) on their stomach for three times, which reduces the bloating. If it works, all good, if it doesn't work, we understand it has eaten something poisonous, then we call the doctor.

I: You said that you treat the cows with home remedies?

P: Yes, Yes, zapota, "dulpo" grass.

I: When do you use them?

P: When we see that they have severe diarrhea and gas, we crush them and feed it to them.

I: For which reasons in the cows would you go to the doctor?

P: When they get wet in the water and they look very tired, we call the doctor. And if the cows don't eat when they get home, they have stomach upset, or the stomach is bloated. if they don't getter better after our indigenous remedies, we call the doctor. Do you understand?

I: Is there any medicine that is both used in the family and the animals?

P: Yes, sometimes when they have fever, we give the cows our medicines.

I: Can you describe which medicines?

P: Medicines for fever, or wounds. We don't remember exactly. The power of medicines used in humans and animals are different. We have less power, the medicines used in cows have high power. If we use one medicine in humans, we would use two in the cows. That we see works. When we see that doctor isn't coming, we do it.

I: Where do you get these medicines from?

P: There's a doctor in the hospital, we go to the hospital and they give us medicines. We make a card and get the medicines for free.

I: Do you ever have any leftover?

P: Sometimes there's some extra after we are done having them.

I: How is there extra?

P: Imagine the doctor has given us medicines for three days and I get better in just two then we don't finish the course. We have one day's medicine spare.

I: The medicine that you use in the family and in the cows, do you have it with you now?

P: No, I don't have it now. We haven't fallen sick recently, so we didn't get the medicines. (laughs)

I: Can you remember their names?

P: No, we are illiterate people. We can't read or write. How can we remember the names?

I: For which problems are the medicines given to you? Why do you usually go to see the doctor?

P: If we have fever. Now the doctor is treating me. I have pain in the legs, starting from the hips down the thighs. If I walk for too long, I can't sit. If I sit for a while, I can't walk for long. It's very painful. I have medicines for that. I can show them to you. Should I bring it?

I: Yes, please!

P: These are the medicines given to me for the pain and the gas. This one (pointing) is bought, the rest is given by the hospital.

The drugs are identified as ibuprofen, paracetamol and calcium citrate

I: The medicines that have been shown to us, in which cases would those medicines be used in the animals?

P2: Sometimes for fever we would use a double dose of the medicines.

I: How do you know how to use the medicines?

P2: There's a quack doctor in this area, sometimes we ask him. Since we are treating for a long time, we have an experience. We know which problem is treated with what.

I: Who is this quack doctor?

P2: [name removed- *referring to the allopathic "quack" doctor in Sriphalbaria]*

I: When do you go to see him?

P2: When the cow has some problem, we go to him and ask for advice. But we mostly go to the "Anchal".

I: In times you go to there, what would be the situation?

P2: In case we don't have time to go to the GP. For fever. He would perhaps double the dose of human medications for the cows.

I: Who does the quack doctor treat? (human health or animal health)

P2: Humans. There's someone here for Animal Health, He's called [name removed- pranibandhu]. He doesn't stay here but his house is in the other village, when we call him, he comes. [name removed- animal development volunteer] stays in Bhabanipur, we have their numbers, if the cows get sick, we call them.

I: Does the quack doctor sometimes treat animals?

P2: If we ask for it.

I: In situations where you ask him to treat animals, what do you ask him for?

P2: He's a relative to us, we would just go tell him the problem and he would help us out.

I: Where does he live?

P2: He comes to the house.

I: He comes to your house?

P2: If we call him, he comes. He just passed through.

I: Does he stay in the same village?

P2: Yes.

I: Where do you go when you fall sick?

P2: We go the hospital. I am taking medicines for the pain in my knees and hips. Once I sit, I can't get up. I am also gotten fat.

I: Where do you go apart from the Hospital for treatment?

P2: We would take homeopathy treatment, but then when my stomach got really swelled, we went to the hospital, they prescribed medicines. I am still taking the medicines.

I: Where is the Homeopath?

P2: In the village itself, he's called Devi Prasad Patra.

I: Does anybody else in the village treat animals?

P2: No.

I: Who treats humans in the village?

P2: [name removed- *allopathic "quack" doctor]*, [name removed- a *homeopath]* and we go to the hospital.

I: Who treats animals in the village?

P2: The GP vet, and we call [name removed- animal development volunteer] and Pankaj *[name removed- Pranibandhu].* They are animal doctors.

I: Can you explain the difference between the human drugs and animal drugs?

P2: Human drugs are different. Animal drugs are different.

I: What do you think is the difference between the human and animal drugs?

P2: There are different doctors for animals, and humans. The animal health doctor would understand animals, the human health doctor would understand humans. They would understand what happened to humans and give medicines.

I: Do you think there's a difference in the way medicines work in humans and animals?

P2: Yes, It's different. Humans and animals have different medicines.

I: We are finished.

END OF INTERVIEW
