# Supplementary material for: “If It Works in People, Why Not Animals?”: A Qualitative Investigation of Antibiotic Use in Smallholder Livestock Settings in Rural West Bengal, India
Source: Antibiotics (Basel). 2021 Nov 23;10(12):1433. doi: 10.3390/antibiotics10121433 (PMC8698124; doi:10.3390/antibiotics10121433)
Supplement: Supplementary file 1 [file antibiotics-10-01433-s001.zip › Supplementary S1_ Interview Transcripts/Site 1/LK10 (site 1).pdf]

Code for Study - ‘If it works in people, why not animals?’: A qualitative investigation of antibiotic use in smallholder livestock settings in rural West Bengal, India: LK10, Site 1

Date: 19/07/2019

Location: Site 1

Interviewee: Livestock Keeper (LK)

Interviewer: Dominic Day (DD)

Translation: Somraj Das (SD)

Transcription: Sayak Manna (SM)

START OF INTERVIEW

Translator: Let’s start questioning you.

Interviewer: Sit down, lower down

Translator: he will talk to me, I’ll tell you, you’ll tell me and I’ll tell him. Alright?

Interviewee: Thank you very much for agreeing to answer our questions.

Translator: Since you’ve talked to us, we thank you for it.

Interviewee: Thank you.

Interviewer: let’s start with, would you mind telling me which livestock do you keep?

Translator: what kind of animals do you keep usually?

Interviewee: We keep Cows, goats, ducks and chickens.

Translator: Cows, goats, ducks and chickens. Could you keep the sound down please?

(Someone: turn down the mobile.)

Interviewer: How many of each does she have?

Translator: how many do you keep of each, in number?

Interviewee: We have about 3 to 4 cows, goats about 5 to 6, chickens 15 to 20, 21, as much as they grow in number.

Translator: Cows around 3, goats around, how many did you say?

Interviewee: goats around 5 to 6.

Translator: Goats around 5 to 6, 15 to 16 chicken livestock, depends how much they wanna populate themselves.

Interviewer: Did she mention ducks as well?

Translator: Ducks?

Interviewee: Ducks, at times we may keep them, at times we might not. They roam around in the water, everyone says they are harder to manage, they eat more than others. Sometimes patient can't eat the eggs of the ducks, that is why we tend to not keep them.

Translator: Well, she's saying that they can keep them as a livestock. But it's too hectic to maintain them. Because sometimes they have to look for them over there, they can swim and they can cross over to some other villages and it's hard to find them.

Interviewer: Who owns these animals?

Translator: all in all who is the owner of these animals?

Interviewee: The owner is the one who keeps them, the father of the house, if we sell them we can invest the money in the household, so the husband and wife, both are the owner.

Translator: well, she's saying that mainly the key member of the family is the owner. Beside that the spouse. And who cares for them are generally the owner of the livestock.

Interviewer: with the key member of the household being

Translator: father.

Interviewer: Could you explain the reason for keeping the animals?

Translator: Why do you keep these animals?

Interviewee: We keep them because if we sell them we get money, we can invest that money to our household, and the children can eat eggs that come from the chicken, the milk that comes from the cows. The neighbors can buy milk from us, and we use that money to buy rice and other groceries.

Translator: it's all about economical and economical help. They lay eggs, they provide milk. Personal usage, domestical usage and the commercial usage, they usually keep the livestock for.

Interviewer: what do they mean by commercial use?

Translator: well about their, they sell the meat outside in the market on open market, and that's how they earn money.

Interviewer: okay.

Translator: that's why they keep the livestock.

Interviewer: how important is livestock keeping economically?

Translator: as a business or as economically how important is keeping the goats and the cows?

Interviewee: it is important because doctors tell us to have milk, eggs. If we have them we will have vitamin, protein. If we sell them we can have a share of money. Our household can be developed. The girls in the house, they can have money in their hands as well, to buy things.

Translator: She's saying that doctor advises sometimes that you should keep livestock just because you can get protein out of milk and eggs, you can also sell them to the open market if you require that kind of money instead in an emergency basis. Beneficial to the poor people.

Interviewer: beneficiaries?

Translator: Beneficial for the poor peoples for keeping livestock.

Interviewer: is there any other economic activity that is carried out at home?

Translator: besides these, do you work otherwise?

Interviewee: sometimes, if we want to, we can work as a laborer at someone's paddy firm, sometimes they hire people from the mission, and we may go there. If we wish we can go there, if we think we would get enough livestock to look after, we may stay at home, look after them. The husbands would go outside to work for money while we stay at home looking after the livestock.

Someone else: the little we earn from the livestock can't support the family.

Interviewee: The women of the household get some money out of keeping the animals and taking care of them.

Translator: there are economical activities as well. The missionaries hire them as blue collar workers. In the season of paddy cutting, they cultivate paddy, for which they have been hired sometimes. There are much more other kind of financial activities as well they do practice.

Interviewer: okay.

Interviewee: when there are 8 to 10 people in a family, and they all got to eat, then we have to manage anyhow

Translator: she's talking about managing, you know, you have to acquire a more than the one source of income.

Interviewer: okay. Yeah. Um, so, could they explain what do they feed these animals?

Translator: what do you feed these animals, the cows and the goats?

Interviewer: the cows are fed on grass, cut and brought from the field. They are also given bran or chaff. We get food from them form the market as well. If they are sick we give them vitamin and medicine. We buy food for the chickens. Wheat is bought. We feed them these.

Translator: Well, she's saying that, green grass, rice ???,when they get sick doctors usually provide them vitamins proteins ??? and that's how they keep maintaining them, and that's how they feed them.

Interviewer: okay. Do they ever give them, ahem, do they put anything in the feed for growth promotion?

Translator: to get more milk out of them, to have more eggs, did you ever give them any medicines?

Interviewee: yes, if we give them vitamins, and feed them with proper food, then we get more milk. We also feed them flour. Vitamins tablets are fed, Tablets and medicines.

Translator: she's saying that yes, sometimes they feed the animals with vitamins with the expectations to get more food, milk and eggs.

Interviewer: okay, uh, and where do they get these products from?

Translator: where do you buy these tablets from?

Interviewee: there's a government doctor in the locality, if we go and tell him, he sends all the medicines. Bring the stool (bench) *Name redacted (Family member)*. Bring the stool here in the front.

Translator: She's saying that, they usually get the medicines from the local market, basically from the doctors, practitioners.

Interviewer: from the practitioners? Okay. Can you also ask them at the end of the interview, um, they got stool for you. If at the end of the interview will it be okay, Could you ask that if at the end of the interviews, could we look at these feed additives in the house?

Translator: medicines you are talking about?

Interviewer: not the medicines, the feed additives.

Translator: the things that you feed them, the vitamins and all, could you show those things to us at the end of this interview?

Interviewee: No, they give us from a file. When we go with our own bottles, they pour some liquid medicines in the bottle. They wrap the tablets in a paper and give that to us.

Translator: They can't. Where they go to get these medicines from, they give them a single product wrapping in a newspaper, so there is nothing to see.

Interviewer: So, there's nothing left to see in the house.

Translator: because they are not provided with the total file, they just rip it up, and give them a single file. They come here and mix it with the food.

Interviewer: Do they give these to all the livestock, all the different forms of livestock?

Translator: do you give it to all the livestock, all that we've seen here?

Interviewee: no, one medicine can't be given to all. There are distinctions to it, cows' medicines are given to the cows, and the medicines for the chicken are given to the chickens. For chickens there is a liquid medicine that is mixed in the rice or put directly inside their mouth. When a chicken is sick one type of medicine is provided and when the cows are affected different types of medicines are given.

Translator: Well, what she's saying is it differs. If you are taking care of the livestock of cows, there is a particular medicine for the cows and it differs from ducks to chicken. And it differs like that.

Interviewer: okay.

Translator: No single medicine can not be effective for all of them.

Interviewer: okay.

Translator: All of the forms of them.

Interviewer: Um, can they explain why?

Translator: why do you do it? These different usage of medicines?

Interviewee: when we describe the disease to the doctor, he advises us to feed the medicine of cows to the cow and the medicines of chickens to chickens. Doctor tells us, this one is for the cows; this one is for the chickens. If we mix up the medicines it would not work.

Translator: Well, what she's saying is that the doctor usually refers them to feed them in a different ways. Supposedly this medicine is for the cow and this one for the chicken.

Interviewer: okay.

Translator: doctor advises them in that way.

Interviewer: Mmm.

Translator: that's why they do it.

Interviewer: Does the doctor tell them for how long or how much to give them?

Translator: does your doctor tell you how much to give them, for how long and in which way?

Interviewee: yes, doctor gives us a type of tablet. And he asks, how many chickens do you have? If I say, I have five chickens, then he would say mix these medicines in the feed of the five. Sometimes they give

us medicines for the cow; it has to be fed twice daily, sometime two tablets at each time, making it four tablets in total in a day. Sometimes we mix it in the rice, sometimes with water.

Translator: Well, what she's saying is yes, there is a procedure to feed each and every livestock. Supposedly a cow, if the doctors giving you a capsule or something, he advises you to mix it with water, three times, four times in one day, or two days.

Interviewer: Okay.

Translator: That's exactly with the other livestock forms like chickens and all.

Interviewer: Okay.Great. Could they, could you ask them why, for what reason, well, do they always go to the same doctor?

Translator: Do you go to the same doctor over and over again?

Interviewee: We have only one doctor in our locality. If he is transferred from this area, that is a different issue, but we have only one doctor here. If he doesn't have the required medicines, he might write a prescription, and then we can buy it from somewhere else.

Translator: it's about the GP health concept. There is the single doctor who is the practitioner, if the doctor gets transferred from there; another new comes along, still they have to face one doctor who is in GP health sector.

Interviewer: Okay. can you ask them why do they go to that person?

Translator: why do you always go to that doctor?

Interviewee: we go to him because if our cows get sick we have to go there. (someone in the background: we get the medicine free of cost). In our locality there is only one doctor for animals, there is no other doctor who has his chamber here, so we can't go to other doctors and pay a fee to them, just like doctors for humans. This doctor is provided by the government, so we go there.

Translator: Hmm, availability. Cause there is no other better option in here around except the one. And that lady said one brilliant word; it's all free of cost.

Interviewer: okay . sure. So, would they ever for any reason go to a different person?

Translator: is there any reason to go for the different person?

Interviewer: would they ever go to someone else?

Translator: Do you usually go to anyone else?

Interviewee: No, we have one doctor here in this locality, he looks after the health of the cows, he has been provided to us by the government. The people of our locality only go to them. Whatever happens to our cows, chickens, ducks, goats, we go to the same doctor. If the doctor doesn't have some of the

medicines then he writes a prescription, and then only we buy the medicines from other shops. When in some case the disease become serious, and if he feels that he can't save the life of an animal, then he writes prescriptions and we buy the medicines from the shops.

Translator: Well, they don't usually do that, they particularly like to attend the same doctor every time. After he prescribes something, and tell him it's not in here right now, go to that place or that store to buy, they do that.

Interviewer: okay. Umm, do they go to a particular store to pick up the medicines?

Translator: Do you usually go to any particular drugstore to buy the medicines?

Interviewee/someone else: how can we buy the medicines all by ourselves if the doctor doesn't prescribe?

Translator: No,no. I'm asking if you go to any store that you go to again and again when the doctor prescribes.

Interviewee: No, no no. We can go anywhere. I can buy it from here, if I don't feel like it I can buy it from somewhere else as well. I may go to Kolkata and at the time of returning I may buy the medicines from the city. There are no rules that I have to buy the medicines from that shop only.

Translator: It's nothing like that, if she feels that that particular provider is not good for me, or I don't like him, I don't feel it good, then they can shift or they can change.

Interviewer: okay. What reasons do they have to go in to the drug shops?

Translator: why do you go to these shops?

Interviewee: we need to buy the cow's medicines. The doctor has prescribed to the medicines, for the cows, for the chickens. To prevent them from diseases I have to buy them and feed them the drugs. Sometimes if I don't get the medicines from one shop I might have to go to a different shop. I have to look for it and have to bring home the medicines. That is why I go.

Translator: Well, what she's saying is, they have to, cause they need to keep their livestock healthy and that's why they need to go to the shops, buy the prescribed drugs and feed them to keep them healthy.

Interviewer: Would they ever ask advice from the drug shop owner about their livestock?

Translator: did it ever happen that the man from the shop told you to take this medicine or that?

Interviewee: No, no no. if it is not prescribed we do not go to the shops. If the doctor prescribes it, then only we go to the shops. We do not make decisions ourselves to buy whatever medicines for the cows. We first go to the doctor, if the doctor doesn't prescribe anything, then we might go to the shop and say, the cow is suffering from diarrhea, then the shop owner would have given us tablets and would advise us to go to the vet.

Translator: one second, what they are saying right now is that, they never do that without the single consultation of prescribed drug, they never take consultation from regular medicine shops.

Interviewer: okay.

Translator: that’s what they exactly do.

Interviewer: yeah, yeah. Um, going back to the livestock, could they describe how they house the livestock?

Translator: how do you keep the livestock the cows and the goats?

Interviewee: for the cows we build hut, if needed we tied him up with bricks, we put up mosquito nets, we have to take care of it, we have to provide them with water, medicines. In the evening we give them their feed and then put up the mosquito net. In summer we tie them up in open space so they can feel the air around them. We clean the feces up, and put the chickens in their pen. In the day time we cover their pens with net so that they can’t go out and get into the garbage area. This is how we take care of them.

Translator: They do a lot of stuffs. For particularly they do make shades for cows, they usually tries to net them so mosquitoes cannot harm them or make them sick. If it is necessary then they have to air the cow to be cool. Now if you look at those livestock which are chickens they specially use nets to confine them, make them much more reliable and comfortable at the same place. That’s why they do a lot of stuffs.

Interviewer: Did they ever ask someone to come in from outside of family to help?

Translator: To do all these work, does ever anyone from outside the family come? To help and all?

Interviewee: No, We do it by ourselves only. When the mother is not present, the children, the girls take care of the livestock. When mother is present she watches them, and the girls go to school. The members of the family only take care of the animals. Why would someone from outside the family help? Everyone has their own livestock to worry about. One is busy with his own household, his own business, and his own cattle. I’m busy with my cattle. When my girl is not at home I usually look after them, when on Sundays she’s at home, she looks after them while I go out of the house.

Translator: Well, there’s none to come over here, outside her house because everyone in here has their own livestock. They keep busy with them. Why should they come over here and help them?

Interviewer: yeah.

Translator: So, they have to do all things by themselves.

Interviewer: okay. And where did they learn how to look after the livestock?

Translator: how did you learn to look after the livestock, how to feed them? Where did you learn it from?

Interviewer: (laughs) we first bought a chicken. We observed and gradually learned what to feed it. She laid eggs from which came out chicks. We thought of what should we feed those chicks in order to make them live. We fed it with powder food, so that it grows up faster. When it grew up a bit, then we fed it with other kind of food items as well. If they are faced with diseases we go to the doctors. Same with the cows, at first we offer freshly cut grass to the cow; if he can't digest the food properly we go to the doctors and give them medicines. Thus we gradually learned how to take care of them.

Translator: what she's saying is, first of all, they bought a chicken, they tried to observe how they survive, after this they wait for lay eggs out of the chicken, exactly another one came in the family and if the livestock get sick, they goes to the doctor and comes with the advice, that's how they are getting evolved in the learning.

Interviewer: okay. Could they describe what happened exactly the last time one of their animals got sick?

Translator: when was the last time any of your animals got sick in here? When was the last time any animals got sick?

Interviewee: umm, just eight to ten days ago, our little calf, this cow got sick. He was suffering from diarrhea. We went to the local doctor and he gave us medicines. He was infected by helminthes worms.

Translator: well, what she's saying is that, that calf you just saw, it just got a disease called worm disease, stemmed in the intestine. It's not an old one, it's just seven days ago's case.

Interviewer: uh okay.

Translator: doctor came here and made them some kind of medicine. And now he is good.

Interviewer: was this the.. um.. GP doctor?

Translator: where was the doctor from? Which doctor?

Interviewee: A vet doctor for the cows. He is from our locality. He is the sole doctor in here.

Translator: she is saying that GP doctor because she is the only one in the GP.

Interviewer: okay. alright. Does she know the name of the doctor?

Translator: Do you know the name of the doctor?

Interviewee: No, no. we don't know the name. (someone : we have the number though) if you have any kind of problem we phone the doctor, he comes over to our home and sees the cow. He brings his own bag of medicines, if needed he provides us with medicines. And if it is not needed at the moment, he would call us and give us the medicines later.

Translator: they do have the number but they don't know the name of the doctor. If they have any kind of emergency as they have the cell phone number of the doctor of the GP, they call him over, he usually comes here and looks over the scenario. That's all. He takes care of the livestock.

Interviewer: is there any situation that they'd ever use anyone else? Is there any situation they'd ever go to someone else?

Translator: has it ever happened that you visited someone else?

Interviewee: No, here we don't have that kind of system or facilities. This doctor may stay here for six months, after six months he would be transferred and a new doctor will be sent by the government in his place, in his department. Then we go to that new doctor.

Translator: he is the only GP guy, they do not have any choice. He is the only one they have to go to everytime, or every situation.

Interviewer: Um, okay. Do they know what product was given?

Translator: Do you know what kinds of medicines are given?

Interviewee: we know that they open the pack up, the meds for helminthes is about this long, the meds for diarrhea is roundish, and there is powder as well. When they give long tablets, we usually feed it with leaves and the cows chew it up. Sometime we bring a bottle, and they pour the syrup in our bottle.

Translator: well, what she's saying is that she's elaborating the sizes of the medicines but particularly she doesn't know about the names of any.

Interviewer: okay. Okay. What's would be the most common reason they'd ask this healthcare advise for livestock?

Translator: come again.

Interviewer: what's the most common reason they'd ask for healthcare advice?

Translator: what are the reasons you go to the doctor?

Interviewee: when they have diarrhea, their excretion smells bad, they have helminthes.

Translator: they are talking about the decentries, diarrhea, and worm cases. Those are the common cases they go to the doctor for.

Interviewer: okay. And do they seek healthcare advices for all of their animals?

Translator: Do you go to the doctor for all your animals?

Interviewee: whichever livestock we have, like cows, goats, ducks and chickens, for each of them we go to the doctor.

Translator: they usually go to a same doctor for all kinds of animals.

Interviewer: have they ever asked for medicines and not been given?

Translator: has it ever asked that he has given the medicines and that did not get to you?

Interviewee: No. he directly gives it to our hands. He calls us up and gives us the medicine directly. When he makes house visits, he examines the cows, the chickens and tells us to go and get the medicines from him. When we go then only he gives the drugs. If at times he has medicines in his bag with him while making house calls, then he gives it to us here.

Translator: it doesn't happen like that. They are given the medicines hand to hand most of the time. There is no situation currently that they have to face that.

Interviewer: okay. Who normally gives the medications to the animals?

Translator: in the household?

Interviewer: yeah. Who actually gives it to them?

Translator: who usually gives the medicine to the animals?

Interviewee: the person who takes care of the animal, the guardian. Suppose I take care of the animal, but at times I can't handle the animal all by myself, so I call for someone to help me. When my husband is not present at home, I call a man from next door to help me feed the medicine.

Translator: Usually she is talking about the serve the medicine by herself, but sometimes it becomes difficult, such as in cow's case, it is difficult to grab them, to grab her so in that case they hire help or they look for men to help them.

Interviewer: okay. Would they ever use medicines for other than treating illnesses?

Translator: tetanus?

Interviewer: other than treating illnesses?

Translator: For what kind of diseases do you give medicines, besides the diseases already mentioned before?

Interviewee: Beside those diseases at times our chickens have problem with their feces, they feel drowsy, they have runny diarrhea, gas problem, and fever of cows, coughing, skin problems, even our chicken had pox. Thus whenever we face problems we go to the doctors.

Translator: Well, she's talking about the same diseases, diarrhea, infections, and dysentery these other things.

Interviewer: but nothing else?

Translator: No. usually.

Interviewer: okay. um, have they ever noticed that the medicines doesn't work?

Translator: has it ever happened that the provided medicine did not work?

Interviewee: No brother, suppose today a medicine is provided, but the animal shows no sign of improvement, so we go the next day and tell him that. He might up the dosage a bit and gives us another medicine, and that might work.

Translator: what she's saying is that, sometimes it happens that, the pill, or the medicine doctor gave her didn't work. After the second day they have to go and tell the doctor that it didn't work. Afterwards they increase the dosage. Then it's gonna work.

Interviewer: okay. So have they ever used these products on the family?

Translator: have you ever used these medicines on humans, besides using on cows and goats?

Interviewee: No, no no. the doctor for cows is different. To get medicines for humans we need to go to a different doctor and for the animals to a different one. (laughs) the medicine for the chickens are to be given to the chickens. Those who want to commit suicide, they would take such medicines. (laughs)

Translator: What she's saying is that it differs; human medicines for humans, livestock medicines are for livestock. If you want to have commit suicide then you can try that.

Interviewer: (laughs) so, what do they see to be the difference between human and animal medicines?

Translator: what do you think is the difference between human and animal medicines?

Interviewee: A man may have pox, similarly a cow or a chicken may have pox as well. Since the species are different, therefore medicines must be different as well. When a chicken has diarrhea the medicine is different than when a man has diarrhea. All the species are not the same.

Translator: Well she just gave an instance as well. We have decentries and chickens have decentries too but that differs so the medicines are different.

Interviewer: would they ever ask for advice for the livestock from the people that they get medicines for themselves?

Translator: has it ever happened that the doctor of the people, whom you know, from them you've asked advices for the animal's health as well?

Interviewee: No.

Translator: no.

Interviewer: have they ever used medicines given to them in a livestock?

Translator: have you ever used the medicines of cows and goats on yourself?

Interviewee: No, no, no no. those who think about committing suicide they would do that.

Translator: if you wanna commit suicide you can try.

Interviewer: Are the providers for the animals different from the providers of themselves?

Translator: human and medicine providers, and livestock providers?

Interviewer: yeah, yeah. They go to different people for that?

Translator: do you go to different store to buy medicine for humans and animals?

Interviewee: No, the same shop but the medicines are different. From the same shop we get the medicines for humans as well as for the animals.

Translator: what she's saying right now is that the same providers have two separate divisions, one for human medications and one for livestock medication. If you go there with a prescription written for livestock medications, the provider of the medicines will give you the livestock and the same provider will give you humans if you went to the prescription of human.

Interviewer: okay. so same provider. Could they tell us the names of the providers or their numbers?

Translator: from which shops do you generally buy these medicines?

Interviewee: sometimes we don't get it from here. We go to different shops.

Translator: where do you get it from here?

Interviewee: In *(town name redacted)*,(???) we have the market over there. There you can find many shops. Supposedly we search in five shops and don't get the medicines in those shops, then we go to the (Diamond??? Name of a place) and get the medicines from there. If we want we can get the medicines from Kolkata as well.

Translator: what was the name of the shop that you mentioned?

Interviewee: ??? bazaar.

Translator: how far is it from here?

Interviewee: from here you can take a toto and it'll cost you five rupees.

Translator: no, we have a car with us, how long would it take?

Interviewee: about ten minutes.

Translator: it's a place she's mentioning where the livestock providers and the medicines provides have shops. Can I write it down?

Interviewer: yes, please.

Translator: *(Local town name redacted)*??? What's the spelling? Can you spell it in English? D?

Interviewee: you'll search for it, you'll find it. (a man says)

Interviewer: Do they have a number to this place?

Translator: I suppose you don't have any numbers. Do you?

Interviewee: No, no. You can't go to the shop today. It's closed today.

Translator: we'll leave today anyway. (to the man) the shop is closed today . (to the interviewer)

Interviewer: Is it in ??? village?

Translator: it's ten minutes from here.

Interviewer: Great, great. I forgot to ask, one question in the beginning. Um, so, do they get, what products do they get from the cows and the goats?

Translator: Do you know what kind of medicines do you get?

Interviewee: No. we don't know. They tear the pack and then give it to us. From one file, not all the medicines are given to one, suppose there are ten medicines in a file, so it'll be distributed to ten animals. We are given three to four tablets and told to give it to them in the space of two days. We don't know the names of the medicines.

Translator: well, she told us that before that they were given bases on one single unit out of the total file, in wrapping paper.

Interviewer: I meant products.

Translator: they don't even know the name of …

Interviewer: in terms of meat and milk, as in those products.

Translator: meat and milk?

Interviewer: yeah, meat and milk from the cows? As in agricultural products they get from them. Is it just meat, just milk? So can you ask them?

Translator: what are you saying? Basically what you are saying, I'm not getting it.

Interviewer: So, in a cow, you can kill them for meat or you can get milk from them and for goats you can do the same. I'm asking what do they…

Translator: So, you mean the commercial

Interviewer: yeah, what do they get from the cows and goats?

Translator: when the cows and goats give milk, do you sell them for meat? Or for economic benefits, like you said, that these animals give milk, do they only give milk, or do you sell them?

Interviewee: when they are pregnant, for three months, after the calf is born, we get more milk, then we keep 500ml for us and sell about 2 liters to our neighbors and relatives. They give us money. With that money we buy the food for the cow, the vitamins and pay doctors fees if needed.

Translator: well, what she's saying is that they don't sell it when they are providing milk. Afterwards they try to sell it for more additional income.

Interviewer: okay.

Translator: Same with the eggs. She didn't mention that separately but it is the same way.

Interviewer: okay, great. Alright.

Translator: they usually deliver it from the neighbor, keeping the 500 lt, 500 ml for themselves and selling the 200, 2 litres more to the neighbors and others.

Interviewer: then they sell them for meat?

Translator: Sometimes.

Interviewer: Alright, great. Okay. That's all my questions. Thank you very much. Thank you, sorry.
