# Supplementary material for: “If It Works in People, Why Not Animals?”: A Qualitative Investigation of Antibiotic Use in Smallholder Livestock Settings in Rural West Bengal, India
Source: Antibiotics (Basel). 2021 Nov 23;10(12):1433. doi: 10.3390/antibiotics10121433 (PMC8698124; doi:10.3390/antibiotics10121433)
Supplement: Supplementary file 1 [file antibiotics-10-01433-s001.zip › Supplementary S1_ Interview Transcripts/Site 1/LK11 (site 1).pdf]

Code for Study - 'If it works in people, why not animals?': A qualitative investigation of antibiotic use in smallholder livestock settings in rural West Bengal, India: LK11, Site 1

Date: 19/07/2019

Location: Site 1

Interviewee: Livestock Keeper (LK)

Interviewer: Dominic Day (DD)

Translation: Somraj Das (SD)

Transcription: Sayak Manna (SM)

D: Interviewer (DD)

B: Translator (SD)

LS4: Interviewee (LK11)

START OF INTERVIEW

D: Thank you for answering few of the question.
B: Thank you for talking to us.
D: Could you please describe which animals you keep?
LS4: Will I able to understand him?
B: Don't worry I am here. There will be no prblem.
LS4: Oh well I just can't understand what to answer?
B: Just answer my questions. What type of animals do you keep and pet?
LS4: Well I have 4-5 cows and 50-60 chickens and ducks.
Someone: Yes, what type? Jersey cow or imported cows?
LS4: Oh one minute. Only one will speak, it's getting recorded.
B: What's he's saying, He keeps livestock as plenty of cows and more than 50 ducks and chickens.
D: Ok, Great! How many cows does he have?
B: How many cows?
LS4: 12.
B: More than 12, one dozen.
D: 1 dozen. And ducks and chickens?
B: More than 50!
D: Each?
B: Each..
D: Each? Okay! Who owns these animals?

B: Who owns these livestock?
LS4: Me!
B: And you are the owner of this house too?
LS4: Yes!
B: They are the owner and they are the owner of this house as well.
D: Ok. Do they look after the animals?
B: Do you look after the animals?
LS4: YES.
B: Yes.
D: Okay. Do they get help outside the family?
B: Do you get help from someone outside the family?
LS4: No.
B: NAO!
D: No, ok. And how do they normally keep the animals?
B: Umm.. how do you keep the animals?
LS4: Well these are exactly how they are kept. You can see them.
B: Like this only? Do you keep them separately in shades and..
LS4: No no! Just like this only.
Woman: This is how we keep, why not take couple of photographs?
LS4: During winters we cover up. Do this and that!
Women: We let them graze in meadows and keep them here.
B: He is saying that this is how they keep.
D: This is how?
B: Making a shade for them in summer and monsoon and afterwards this is it! (NO, It wasn't said). This is how they maintain.
D: Okay and the ducks and the chickens?
B: Apart from the cows and goats, how do you keep the ducks and the chickens?
LS4: Well, there's a small room that's made and they are kept in that! We feed them inside!
B: Well he's saying that there're particular shades for those, where they keep them and that's it!
D: Ok, what products that they get from the animals?
B: Medical?
D: No
B: feeding (..?)
D: (,,?) products like meat, milk (..?)
B: What do you get from these?
LS4: For an instance, cows help us to earn 50-60 thousand in a year by giving us milk. And 30-35 thousands after selling the eggs of the hens.
B: Umm, what's he's saying about..is it annually?
LS4: Yes it is.
B: they generally make money by selling milk, is more than 50K per annum and chicken and eggs 20/30K per annum. Total 80K per annum.
D: Ok, ahh, and (..?) meat?
B: Do you sell for meat?

In group: YES!
Woman: When a hen stops laying eggs we sell it off in 400-500 rupees! We have red poultry chickens, they weigh 4-5kgs, so we sell at 400/500/700, the indegenous ones are 300 rupees! When we see they don't lay any more eggs, we either sell or eat amongst us!
B: What ladies saying that it depends. When it get oily and fatty, they stop giving or provide milk, they sell it for meat. That's the way they do it.
D: Will you mind asking if the man answers.
B: We want you to say, because you signed for the answers.
D: Do they use these products domestically?
B: Do you use them for home? The milk, eggs you get do you use them at home?
LS4: No, we sell.
B: You sell the whole of it?
LS4: No, we have some too. We have 1 L milk and may be 1 chicken in a month.
B: Well, maximum or most of them they use it for commercial but they keep 10 or 5% of the total livestock they keep for the personal usage.
D: OK, right. What do they feed their animals?
B:What do you feed them?
LS4: We give milkon mash, straws/hay, chaff, grass, vitamins etc
Woman: leafy vegetables.
B: Those bottles right?
Woman: They also eat jaggary made of sugar cane juice.
B: What's he is saying is exactly feed them, green grass, rotten grass, rice straws and these kinds of vitamins suppliments as well.
D: OK. Do they give anything for growth promotion?
B: Do you give anything that produce more milk or eggs? (Dom, never asked this question)
LS4: Yes, these vitamins.
Woman: Oye! See those big jute bags? We have to get them. Each bag weighs 60 Kilos!
B: What are those, vitamins?
LS4: Yes!
Woman: Yes those are vitamins.
LS4: It has roughages, corn, bread crumbs.
Woman: And then we make a mash!
B: Well they do, they do use vitamins
Woman: Look at this..
B: This they generally use a mixtures of maizes, wheats and et cetera! And this make them more productive!
Woman: Since the morning, we have to give 5 kgs!
D: Do they put any medicines in that?
B: Vitamins!
D: Medicines!
B: In there?
D: Yeah!
B: Do you put any medicine in this?
LS4: No.

Woman: Medicine is given separately!
B: No. They don't use any kind of medicines in there!
D: Ok. umm.. what did he learn how to look after the animals?
B: How did you learn to keep them? Or maintain them?
LS4: This is our business. We were told how to do it by our fathers and grandfathers. We are doing exactly that that!
B: It's heritance!
D: Could he explain what they do when the animals get ill?
B: what?
D: Can he explain what they do when one of the animals become ill?
B: What do you do when your cow or buffalo or livestock get/s ill?
Ls4: Doctor!
B: They go to doctors.
Women: His fee is 200-250 rupees! If he comes he will charge 500 rupees! So he is contacted directly, when called he would come and check. We don't know anything. When one of them is ill, we call and the doctors come! Reputed doctors!
D: What's she saying?
B: They are talking about the doctors, their charge is more than 250 every time, expensive!
Woman: well the government has made some arrangements in every locality.
D: Could you remind them that it's got to be the man that's answering?.
B: Sir, you please say something. Because your words need to get recorded!
LS4: Only if you ask question then I will answer.
B: I'm questioning.
LS4: You both are talking inbetween you! Yes I could understand you will trnslate!
(UNNECESSARY TALKS)
D: could he describe who these doctors are?
B: Who are these doctors that you visit?
Woman: From the Government.
LS4: Government doctor.
Woman: We have to pay them to get them here!
LS4: If we take them there, then we don't need to pay but if he's called here, he will charge!
B: Well what's he saying is, it's maximum time of the government, and if you are not going to the government doctors then you have to pay to the private (..?) consultancy!
D: Ok, and why do they go to these providers?
B: Why do you go to them?
LS4: Where shall we get it in free? Who will check for free? They say bring your cow to our chamber we will check it for free but not everything is free.
Woman: Nothing is free, we have to pay for everything.
B: In private? They say it's free of cost if you go to the government and if you are not going to the government, you have to go to the private. And you have to pay (..?) for that aswell.
D: hmm hmm, can they describe why they would go to the private?
B: Why do you have to go to private sometimes?
LS4: There are some pros and cons. If there is sickness, if there's fever, some cold or cough, for these reasons.

B: well it depends, if their livestocks are having some cold and cough or some kind of sickness, they go to the private usually (Usually?)
D: Ok, And is it same for all of the livestock?
B: Other than cows, is it same for the other animals? (the question is NOT specifically asked)
Unanimously: YES!
B: Yes!
D: Can they explain a situation where they last used antibiotics?
B: when was the last time you gave "medicine" to your cows? Antibiotic.
LS4: Only today we gave.
B: Is it antibiotic?
LS4: What? What is antibiotic? I don't understand!
B: Well they have applied a medicine today and he is not sure if it is antibiotic or not! (The person doesn't know what is antibiotic, he confessed and didn't say he doesn't know whether it is antibiotic or not)
D: Ok. Could we look at this medicine?
B: Do you have that medicine? The cap or something?The one you gave today
[Woman: Why is he showing the same medicine, we have other medicines too
LS4: Shut up you!] (Happened inbetween the talks)
LS4: Show it please.
B: Please keep them, we will see after the interview!
Woman: These are what we got, given to us by the government. We have to buy, we have to buy everything!
D: So why was this medicine given?
B: Why? Why do you give these medicines?
LS4: How? Well in an entire day we have to give them 25 gms! Per cow!
Woman: It's 100gms! He doesn't know anything!
LS4: The calves get 25gms and adult cows get 100gms.
B: He's talking about the dosages. 25gms for calves and more than 100gms for adults for the livestocks.
D: But why did they give this medicine?
B: Why do you give THESE (should have said this) medicines?
LS4: For better health and more milk. Disease will get cured!
B: They are talking about to keep their livestock healthy, fit and far away from diseases that's why
D: Ok and who provided them with this?
B: Who gives you these medicines? Where do you buy them from?
LS4: From shops, *(Local town name redacted)*
B: *(Local town name redacted)*. There's a shop in *(Local town name redacted)*, that's some kind of place I guess.
D: Yeah, Is this like a drug shop or pharmacy?
B: Is it medicine shop?
Unanimously: Yes!
B: yes, it is.
D: Can he explain where this shop is in *(Local town name redacted)*?

B: Where is this shop exactly?
Women: 46 crossing at Sarisha!
B: How far it is?
LS4: 20 mins in bus.
B: 20 mins from here.
D: Have they ever noticed that a medicine is given and it doesn't work?
B: Has it ever happened that you got the medicine but it didn't work?
LS4: Yes, many times! That medicine is not over and again a new medicine is prescribed. A lot of rejected medicines are lying with us.
B: He's yes yes it happened many times.
D: Okay, and what does he do when it happens?
B: What do you exactly do when it happens?
LS4: Doctor writes the meds, we go to *(Local town name redacted)*and get them.
B: When they don't work?
Women: Yes!
B: He is saying, when he faces this kind of situations or problems they go to the same doctor, get another kind of new medicines, go to the shop, buy them again, feed them again.
D: ok and do they most commonly go to the doctor for?
B: Usually to which doctor you go?
D: Here only, *(Local town name redacted)*. Government cow doctor.
B: That GP guy, the only one who practices around here, they go to the same doctor.
D: For what reason they (..?) commonly?
B: Why do you go, for what problems?
LS4: Many reasons, just like humans.
B: Exactly what problems?
LS4: Mostly cold, cough, fever! Fever is common, they stop eating. This year our 10/12 cows suffered from eso. The disease is eso! Ulcer in tongue! For that 50,000 rupees is gone! 1 cow has almost became useless, it used to give 15kgs of milk! Now she jus doesn't give milk.
B: I see, the geberal diseases specially cold, cough and (..?) other than that there's a particular disease which makes your oral area wounded and your feet wounded! after that they almost speand more than 50K and lost a cow!
D: Ok. Who normally gives the medicines to the animals?
B: Who gives the medicines to them?
LS4: We!
B: He does!
D: And is he told how much and for how long?
B: Is it told?
D: Yes!
B: So are you told how much to give?
Unanimously: The doctor writes it!
B: Well he's saying he's always advised by the doctors! How to give him and how much to give (..?)
D: Okay..and he has ever given medicines to animals that are meant for the family?
B: Has it ever happened that animals were given human medicines?

LS4: Yes!
Women: Yes of fever. We call the doctor and talk about the fever, they prescribe the human medicine.
B: Fever, it was fever!
D: Does he know which medicines he gave?
B: The specimen you want to see?>
D: Can you ask him which medicine (..?)
B: Which medicine was given, is it known to you?
LS4: Nah, I don't know.
B: No he doesn't know!
Women: These medicines, we don't know the names!
D: Can he explain why he gave the human meds?
B: Why suddenly you chose to gave it?
LS4: The doctor wrote and he bought!
B: It's the doctor who told him to get.
D: ok. So does this doctor provides medicines for both humans and animals?
B: Come again?
D: So does this doctor normally provides the medicine for humans and animals?
B: The doctor you go to? does he give meds to both human and cows?
Together: NO No no, only to cows!
B: Only cows? No the guy is the practioner of the livestock.
D: Okay, Erm, does he sometimes get advice about the medicines for his family from this man?
B: The doctor you go to, does he advice if a member of your family is sick?
LS4: Na.
B: No.
D: Erm, and has he ever used medicines that were meant for the animals in people?
B: Have you ever done the reverse thing? Given people the medicines meant for animals?
LS4: No.
B: Nao!
D: So do they ever get medicines from people (..?)
B: Come again?
D: Do they ever get medicines frompeople who give medicines to humans and animals?
B: Has this ever happened that you went and saw he gives both animals as well as humans?
Women: NO!
D: Can they explain what they see as a difference between human's and animal's medicines?
B: What do you think, what's the difference between the medicines of humans and animals?
LS4: No I can't say!
Woman: Oh they are different!
B: He can't explain it.
D: No?
B: NAO!
D: That's fine.. umm..can he now provide the name of the person that provides him with animal anti.. eeeehh animal medicines?
B: Do you know the name of that person/s from who you buy medicies?

LS4: The shop I buy the medicine, the guy's house is at *(Local town name redacted)*. Mohammedan (Muslim), he has a big shop!
B: Ok, what's the name?
LS4: His name is *Name redacted (IP4)*
B: The guy who provides medicine is called *Name redacted (IP4)*
D: *Name redacted (IP4)*, does he have a number?
B: Number?
LS4: I had the number, now it's lost!
B: Na. He lost it.
D: Does he know where we can find?
B: Where can we go to get him?
LS4: *(Local town name redacted)*, *Address redacted*! Go there and say, we want to go to *Name redacted (IP4)* med shop!
(Unnecessary commotion/talks)
D: Does he know, this man is private or government?
B: Is his shop private or government?
LS4: No private!
B: He runs a private shop.
D: Is this man formally qualified?
B: Do you think he has any training?
LS4: I can't say, his father was a big doctor.
B: No does he have any training?
LS4: I can't say it.
B: He can't say, that but he knows the father of this guy was a Doctor -_-
D: umm and does he get advice about his human health from this man?
B: The shop you just talked about, when anyone in your family falls sick..do you take advice?
Woman: NO!
B: He just sells meds for animals?
Woman: Yes!
LS4: He sells meds for pigeons, hens, dogs, cows buffaloes etc
B: He is saying that, *Name redacted (IP4)*, the guy only runs livestock. He doesn't sell the human products.
D: Okay, is there anybody else that he would go to for medicines?
B: Do you go to any other shops other than this?
LS4: No
D: No, Can you thank him very much for his time?
B: We troubled you a lot. Thank you!
D: Thank you very much
