# Supplementary material for: “If It Works in People, Why Not Animals?”: A Qualitative Investigation of Antibiotic Use in Smallholder Livestock Settings in Rural West Bengal, India
Source: Antibiotics (Basel). 2021 Nov 23;10(12):1433. doi: 10.3390/antibiotics10121433 (PMC8698124; doi:10.3390/antibiotics10121433)
Supplement: Supplementary file 1 [file antibiotics-10-01433-s001.zip › Supplementary S1_ Interview Transcripts/Site 1/LK12 (site 1).pdf]

Code for Study - 'If it works in people, why not animals?': A qualitative investigation of antibiotic use in smallholder livestock settings in rural West Bengal, India: LK12, Site 1

Date: 22/07/2019

Location: Site 1

Interviewee: Livestock Keeper (LK)

Interviewer: Dominic Day (DD)

Translation: Somraj Das (SD)

Transcription: Sayak Manna (SM)

D: Interviewer (DD)

B: Translator (SD)

S: Interviewee (LK12)

START OF INTERVIEW

D: so, the first question I'd like to ask is which livestock do you keep?

B: which livestock do you keep?

S: Basically we keep only cows. A long time ago we used to keep goats as well. But now people here don't keep goats anymore. Maybe in other region they do keep goats. So, we just keep cows.

B: basically he keeps cows now. They used to keep goats but right now they are not interested in goats as a livestock.

D: okay. And how many cows?

B: how many cows?

S: I have in total 4 cows.

D: And for what purpose do you keep them?

B: why do you keep them?

S: So, that we can get milk from them and we also collect cow dung. Sometimes,… uh..basically for milk. We also get the calf.

B: basically he keeps them for milk and cow dung.

D: Any other reasons at all?

B: Any other reasons for keeping cows beside this?

S: Besides using the milk, we keep the cow dung as well. We dry them up and use them as fuel. We also use the urine of the cow to make “Charanamrita”. As we are Hindu, we make charanamrita out of the urine of cows and use it during funeral and other holy Hindu pujas and ceremonies.

B: Basically except milk, they use the dung for burning; they use the dung as a fuel. He is talking about a religious aspect, in Hindu religion the urine of the cow is used in worship ceremonies and funeral ceremonies. This is the reason.

S: and from cow milk we can make a lot of sweets, various types of sweets. Some people come and buy this milk, and they can make a lot of sweets from it. When you return, on your way back, there are many sweetmeat shops here, you can taste the sweets from here, they are so sweet. You can buy a roshogolla, which is very famous in Kolkata, it taste so good. It’ll only cost you 5 or 6 Indian rupees. This is very famous in here.

D: okay. I got that. So, do you sell this milk or dung commercially? Or do you use them at home? Or both?

S: Every year a cow breeds a calf, so we get the milk from them. In one more way we use cows, when we grow paddy, we use cows for ploughing the fields. But these days, we have tractors, so we don’t need cows to work in the field anymore.

B: Sir is saying that there is one thing; a cow breeds one single calf per annum. They make the use of cows to plough the field. He’s talking about the technology that how tractors have replaced the cows in field.

D: So, does he use his cows for ploughing?

B: Do you use your cows for ploughing as well?

S: No, no. we use it just to get milk, this is my personal cow not for farm. If you go from this way, you’ll find a lot of farm over there. In a farm you’d find 20, 30 cows. But I have four cows only, for personal use.

D: So, do you normally consume the milk yourself or sell the milk?

B: do you use all the milk that you get or do you sell?

S: What we get is more than we need. So, people from five to six families may come and buy milk from us. Four cows produce a lot of milk. So, some people come and buy and drink this milk.

B: Did you get it?

D: I think so. Yeah. Who looks after them?

B: Who looks after them?

S: My mother, my aunt, my uncle, sometimes me. All of our family members take care of the animals. This is kind of a part time job. Sometimes we go for our company's job and after coming back we take care of the animals. We think cow to be the god. Hindu people consider cow to be a god, so we want to take care of the cow properly.

D: how economically important are the cows in this household?

S: it is very good for us because not only milk but also, we can keep our health properly. You should know better, that it is scientifically proven that consuming milk and eggs are good for our health. That is why we think having milk is really beneficial for us. And did you tell him about the charanamrita?

B: yes I did.

S: okay.

B: He is talking about the urine, that we have a religious value attached to it. He's insisting me to make you understand of the value of cow's urine in Hindu ceremonies and religious practices.

D: okay.

S: we don't use the skin to make leather and all. Many people do after a cow dies, but we don't.

B: and there is a tannery. But they do not usually deal in that kind of stuffs after the death of the livestock.

S: we don't like this, some people do but we don't. We don't like to produced leather out of the skin. Some people also consume cow as a meat. They may eat the cow after it is dead, or butcher the cow for meat but we, the Hindu people don't like this.

D: okay. So, what do you normally feed your cows?

B: What do you feed them?

S: Green grass, hay, rice stalks and husks of coconut. There are machines that produce the husks of coconut and at the same time produce the coconut oil. So we give the husks to the cows.

B: So, Sir is talking about the diet which is green grass, rice stalks and a kind of thing which they call 'khol'. Khol is just a thing. If you put tinier size of coconuts and put a machine on it, the machine is going

to squeeze the entire coconut, and there will be raw coconut oil, and after that whatever is remained that is called khol, in their term, they feed this as well.

D: why do they feed this?

B: why do you feed this?

S: this is good for cows. They like it better when we mix khol with regular green grass. Just as we like chicken, mutton and prawn, similarly cows like coconut husks. They feel very happy when we feed this to them.

D: Do you add anything else to the feed?

B: Do you add anything else to feed them?

S: Glume/Bran. We feed them Glume, and water is a must. We feed them Glume of grass peas, khesari. They like it.

B: Sir is saying that there is water that is of course required; there is one thing that is called bhusi which comes out of pea or pulses. They keep it dry and they turn it into a dust form and they add it to the feed.

S: and cow, they like guava very much. Do you know guava? They like it better.

D: yes.

S: we have five to six spare guava trees, whatever we from these trees, we feed them that. They like it very much.

D: so, leftovers? Was that the food that you leave, that you sometimes give to the cows?

B: well, what he said is that they are rejected guavas.

S: I'm showing it to you. (Exits)

D: interviewee has left the room to show us guava.

S: these are such guavas, this is riper. We don't like such ripe cows so we give it to them.

D: okay. And why do you give it to the cows?

B: why do you give it to the cows?

S: they like it better. When I give it to them, they come running, they want to have more.

D: I see. Okay. Thank you.

B: it is salted curd lassi.

D: Thank you.

S: you are most welcome.

D: So, where do you keep your cows?

B: where do you keep the cows?

S: we've arranged a room, cow shed, there we tie the cows with ropes and keep them there. And during the day we tie them up in fields or side of the road, so they can have grass.

B: So, what he's saying is that, they maintain a separate space which is called Goahl in Bengali, it is basically a cow shed. And in the daylight they usually keep them outside, so that they can have fresh air and fresh grass and all.

D: and is this the same in every season of the year?

B: do you keep them like this in every month of the year or every season?

S: In every season we keep them like this only. Only in monsoon we keep them here and arrange their feed here.

B: Sir is saying almost but in the monsoon they try to keep the cows in the goahl which is cow shed and they provide food and everything in that shade because it's monsoon out there.

D: okay. Great. And where do they learn how to keep these cows?

B: how did you learn how to keep the cows?

S: it's a family tradition. My father used to do it; he learnt it from his father and so on.

D: and where is your main source of information now? What's your main source of information about keeping cows now?

B: where do you get your news (info) from now regarding your livestock keeping?

S: West Bengal government has provided us with a veterinary doctor in each Anchal or blocks. A few villages come together and make a block. We have a government provided veterinary doctor in every block. We can go there if we face any problem or we can go to another vet who is a privet practitioner who is a non government doctor.

D: and why do you sometimes go to the government doctor and sometimes go to the non government doctor?

S: Suppose it's night and the government doctor's chamber is not opened and he' not there, then we go to the privet doctor if our cows are unwell.

D: Is there any other reason, why you would go to the local doctor?

S: No, for this reason only we go to the local doctor. Actually the government vet doctor is very good, the best in west Bengal. But the problem is that they are available only from 10 to 5. And they may come 4 days, 5 days a week, at times 6 times a week. But if we face problems, say about 7 in the evening, we can't go to them, because it's not open.

D: okay, thank you. Could you give me the names of the government and the non government doctors that you go to?

S: yes of course, *Name redacted (Public Private VPP1 Site 1)*. And the government doctor, I don't know the name of, but now they are available, from 11, or 10.30 to 5 o'clock. I forgot his name.

D: okay. Do you have his number?

S: number? I guess I do. Let me check.

D: okay, Interviewee has left to get the number of the government doctor.

B: we've heard his name before for someone else. Check your list.

D: okay. Thank you.

S: welcome.

D: so, I'd like to talk to you about sort of what you do when your animals get ill, if that is okay.

B: he wants to know what you do when your cows get sick.

S: when the cows get sick we at daytime go to the government doctor and if it is night we go to the privet doctor. *Name redacted (Public Private VPP1 Site 1)* is a very helpful person.

D: could you describe the last situation when your animal was ill?

S: last time when one of our cows was in labour pain,(or had a calf recently) we went to the government doctor, he came quickly and he solved this problem properly. Before this also, when our cows give birth yearly, they come in and they help us.

D: Could you explain why you usually go to the government doctor?

B: why do you usually go to the government doctor?

S: First of all this is free of cost. We need to pay no money to go and see him. It is profitable for us. And secondly this doctor is a very helpful person. If something goes wrong while with him we can complain about him to the police and his job is under the line. So, the privet doctor is not better than government doctor.

D: Could you explain common reasons for why you'd seek healthcare for animals?

S: when they get wet, at times they don't eat properly, even though they are provided with good food. Sometimes oxen fight with each other and we also have cases where dogs come and fight with the cows. They might bite the cows. Then we usually see healthcare.

D: How easy do you find it to get hold of the medications?

B: how well can you access the medicines?

S: we don't have that much of medicines in the Anchal, block. (Government doctor). We can say we have a medicine problem, even the doctor is good but the medicines are not always available. Two three types of medicines are available, not all. So basically we have to buy medicines from other places, from pharmacies. We have to pay for those medicines.

D: And do you buy these drugs on the advice of the animal health providers?

S: well, I can tell you this, if we get all the medicines for free of cost, we'll be happy.

D: but do you only ever get drugs from the shop on advice of one of the doctors?

B: do you take advice and then buy the medicines?

S: obviously. Yes. We take advice and buy the medicines. But let me tell you one thing, keeping cows is an expensive job. Mostly poor families used to keep cows for the milk, but these days it's getting harder to keep cows. If the government helped them with any kind of subsidies, or maybe they could provide with the hays for the cows, then it would be really beneficial for a lot of families.

B: Sir is saying that in nowadays, keeping the livestock is too expensive particularly for the poor families in this area. They try to cultivate with the help of the livestock. But the cost of maintaining livestock is quite expensive. And he wants the government to support him, support the poor families with straws, medicines just for the maintenance of the livestock.

S: Maybe government is giving these things, but the common people are not getting it. Government might think that they are sending straws, and other items for us, but the common people are not getting the things.

D: and why do you think this is?

B: why do you think so?

S: maybe government is sending it to the block. And politicians are getting hold of it. While some enjoy the benefit, those who are actively into politics, local commoners are not getting benefitted.

D: thank you. I'm not sure if he has answered this or not already. So, does he buy medicines when he has not spoken to the healthcare provider?

B: Have you ever bought medicines without the advice of the doctor?

S: No, we don’t buy medicines without consulting the doctor. First we consult the doctor and then only we get the medicines.

D: and do you go to the same people regarding your own health as well as your animal’s health?

B: and do you go to the same doctor for your own health?

S: No, no. that is a different thing. Our doctor is different doctor. They are all M.B.B.S. and F.R.C.S. London, that sort of things. But animal doctors, Veterinary doctors and M.B.B.S doctors are different. We go to a different place.

D: Okay. The drugs that you buy are they for the same places, for animals and for people?

B: do you buy the medicines from the same place for both animals and for humans?

S: Most of the medicines are same. Our medicines and cow’s medicines and dog’s medicines are the same. If they have fever, and we have fever, the medicine for the fever is the same.

D: okay. So, could you tell me where this drug shop is where you get this drug from?

B: Could you please tell where this medicine shop is?

S: It is in *(town name redacted)* more, it is in our block. There are in total three pharmacies, namely – ‘Ma Medical Hall’, ‘Green View Medical centre’, another one… I forgot.

D: Could you name the medicines that you gave to both people and animals?

B: Could you name the medicines that are being given to both animals and humans?

S: yes, wait for a moment.

D: Interviewee just left to get the medicines.

S: Liv 52, Liopeptine, Paracetamol, Dontl, AtoZ. Spelling might be different, I forgot. We have another doctor, a house doctor; he uses these kinds of tablets. You know what, once our cat got sick, and we went to the government veterinarian doctor, and he told that our medicines, that is human medicines and the pet animal’s medicines are the same. They are giving these paracetamol for the fever.

D: so, would you use these?

S: yes, we have already used this. Now our cat is dead. 15 to 20 days ago he died. But the dog is good. We have a crazy little dog.

D: and do you ever use medicines that get for yourself and your animals?

B: the medicines that you get for yourself, do you get it for the animals as well?

S: when we get order (advices) from the doctor then only we buy medicines for them. And our medicines might be same but we are not using it for ourselves.

D: So, you use medicines on yourself that you got for your animals?

S: No, no no. We don't use their medicines. But this paracetamol, if we have some problems, we can eat it because it's the same medicine. If we have fever, without going to the doctor we may take this medicine. We don't take liopentine, it is only for animals.

D: okay. Could you explain, do you understand the term antibiotic?

B: Do you know what antibiotic is?

S: No, I don't know about antibiotic.

D: So, would you go to your human doctor about advice regarding your cow's health?

S: No, no no. We do not go to the regular doctor for animal's advice; we go to veterinary doctor for that. For animals we go to vet doctor, for us we go to M.B.B.S and F.R.C.S. doctors.

D: So, apart for paracetamol, what do you see is the difference between human and animal medicines?

B: repeats

S: I don't know much about medicines. The veterinary doctor tells me that some medicines works the same for animals and humans. So, they give the medicines.

D: who administers the medicines to the cattle?

B: who takes care of the giving medicine to the cattle part?

S: we do not provide medicines to the cows when they are not sick. When we see that the cow's health is not good, then only we go to the doctor. Otherwise we don't go to the doctor.

D: and when they are ill who then administers the medicines?

B: When the cow is sick, who gives them medicines?

S: We do. Sometimes my mother, my uncle, aunt, sometime I do, or other family members.

D: and are you instructed by the medicine provider on how much to give and for how long?

B: Does the man from the medicine shop tell you how to give the medicine and how much to give?

S: No. The veterinary doctor and pharmacy man both instruct on this matter. Suppose doctor tells me that I can give the medicine in the morning and in the night, sometimes I might forget then if I ask the man from the pharmacy shop, they would also tell me the same. There is no doubt that both are good.

D: and to go back to the beginning, sorry, because I forgot, what other economic activities happen in this household?

B: repeats.

S: No problem it's okay. We not have much industry over here that is a problem. We have a lot of able engineers and trained professionals here but not job opportunities. Business is also not good here, it is very bad because we have only one shop, you've already seen. And from this shop we may earn monthly three thousands. Even if we want to work in a hotel we can't. we do not have four stars and five stars hotels here. They are in Calcutta. And it is almost three hours journey to Kolkata. To go and come each day is not possible for us. So, we want more industry over here.

D: So, what do people in this household do other than looking after the cows?

B: repeats

S: My mother is housewife, my father is a seller. I am an hotelier. *Life history redacted.* After five to six months we'll go to a different place for a different job. My uncle is a labour and my aunty is a housewife. So, this is what it is.

D: thank you.

S: Welcome.

D: Going back to the medicines, do the providers tell you what products are they giving to the animals?

B: the people from the pharmacy, do they tell you the names of the medicine when the hand you over the same?

S: yes. They provide us with the names. They tell us and they write the names and ask us to go to the pharmacy.

D: Um, is the list that you gave us previously, are those all the medicines that you have been provided with?

B: Repeats.

S: No, I forgot some names that were given to me long back. But the list I gave you, that were given to me, just a month, or fifteen days ago. We are using these medicines that are why I remember these. And the rest of it we forgot, it was long back.

D: with your permission is it okay if I see and take some photographs with some of these medicines?

B: Repeats.

S: obviously. But my dog is in another house. It is my father in law's house. He has only one daughter, so we need to take care of them. You can take pictures of the cows though, no problem. Maybe they are kept outside, beside the road. Two of them are over there.

D: that'd be great. Thank you. I think that is all my questions. Very much appreciate it. Thank you very much.
