# Supplementary material for: “If It Works in People, Why Not Animals?”: A Qualitative Investigation of Antibiotic Use in Smallholder Livestock Settings in Rural West Bengal, India
Source: Antibiotics (Basel). 2021 Nov 23;10(12):1433. doi: 10.3390/antibiotics10121433 (PMC8698124; doi:10.3390/antibiotics10121433)
Supplement: Supplementary file 1 [file antibiotics-10-01433-s001.zip › Supplementary S1_ Interview Transcripts/Site 1/LK13 (site 1).pdf]

Code for Study - ‘If it works in people, why not animals?’: A qualitative investigation of antibiotic use in smallholder livestock settings in rural West Bengal, India: LK13, Site 1

Date: 25/07/2019

Location: Site 1

Interviewee: Livestock Keeper (LK)

Interviewer: Dominic Day (DD)

Translation: Somraj Das (SD)

Transcription: Sayak Manna (SM)

Dom: Interviewer (DD)

LSK: Interviewee (LK13)

START OF INTERVIEW

Dom: Ok so the first question I’d like to ask is, which livestock do you keep here?

LSK: We have cows, bulls and then the pigs, and we have dogs also… depends… that’s it

Dom: Okay, and do you know how many of each of these animals you keep?

LSK: The exact number I don’t know because this matter they look after it, their own department. But I think the cows and bulls in total are around 9 in number and the pigs are like around 15. Dogs we have 6 and the chickens I don’t know exactly

Dom: sure, and for what purpose do you keep these

LSK: Well it’s actually a kind the involvement of our, the… the patients plus the workers. WE have so many widows, ladies, so they are in need of work, right? Three of them are employed to look after all these and the three of them, three families, we employ them and pay them monthly. And plus sometimes we have a kind of walk around (?) with our patients and we get them to do whatever they can with their disability, ok? So in that way we have kept it. It’s not for any gaining in business or that we profit from it, no. And most of these things you know, sometimes we give up. Somebody needs like that. The pigs are sent into Kolkata. The cows, mostly we sell them and we again recycle that money into it’s own… where the departments buy them their feeds.

Dom: And do you get any products out of them that you use?

LSK: The manure we use for the garden, it’s very useful. That’s one thing. And plus, see sometime, all of them they don’t take the beef but the pork we eat. But the cows… in the surrounding area they eat, the people they take it, but here mostly we have mostly, the majority is Hindu so we don’t use all that, we just sell it. And chicken and all, eggs we eat here sometimes it helps. And dogs are for the security, night guard (laughs).

Dom: And did you say previously that you keep pigs.

LSK: Yeah yeah, that's what I've… yeah pigs we have.

Dom: Pigs as well. And, is it for all the different animals, is it the same people that looks after all of them?

LSK: Yes yes yes

Dom: Umm, and what products do you use the pigs for?

LSK: Well that's what I said, it's for the meat. Plus the waste is used for the manure for the vegetable garden. It's a mixture of manures. So also the cow's dung we use for the mixture of manure. All that, whatever we have access (…?) all this vegetable form our garden is organic, it's all from that.

Dom: And the ducks?

LSK: The ducks are there also. They're just for a little bit aesthetics (laughs), ok? Just since we have got a big place, the ducks are swimming in the pond it looks good. Ok?

Dom: OK.

LSK: You know it's not for the, for the, anything. And sometimes they're a little more, in the evening we decided ah we don't need one or two… (Laughs). It's a meat say, a meat.

Dom: And who owns these animals?

LSK: We, we the missionary. It belongs to us

Dom: As a company, sort of..?

LSK: As a property, it's a kind of income stream

Dom: Ok, thank you. So what do you tend to feed all the different animals?

LSK: See the cattles, the cows are being fed with the mix they are… daytime taken out for grazing. And in their timing they are being fed with straw and the grass mixed together, cut. And also we have, we get little bit product from that liquor factory. There's a waste of that liquor, it's called syrup here locally. It's mixed in that… this one, give them and they eat well with that. When the grass is mixed with that they eat quite well. It's a kind of a (..?) we can see the changes in them when we started feeding that. So only that's the things we feed.

Dom: And the other animals?

LSK: The pigs we have the paddy husk and a little bit sometimes with the rice and the broken rice. And mixed with syrup again… and all the rest of our food waste whatever we have. All that we bring in together. And vegetables all that we shun, waste and all we cut together, mix, cook and give.

Dom: Do you add any other products to the food?

LSK: It's mostly nature things in general

Dom: And is that the same for the chickens?

LSK: The chickens, maybe we buy for them the type of broken wheat type of thing- chicken feed. So that one. Previously, we used to give the bulger (?), you know bulger? That American… the bull wheat. So that we used to give to them so now it is we are not getting. We now buy feed for the chickens.

Dom: And is that a sort of… commercial feed?

LSK: pardon?

Dom: is that a commercial feed?

LSK: Oh yeah yeah. In the way we go and buy. Yes it is…

Dom: Thank you. And how do you tend to house the animals?

Lsk: They are quite ok here. It is in the good (…?). The nature wise they are also resting in good place (laughs). And a surrounding that is not polluted. So far I have not seen, I've not seen them getting so much sick. Sometimes they, the nature always and (…?) wise, there is a sickness. Mpre than that one no. I have not seen a chicken die out of sickness here, or cow, or even pigs. I am here, I am here for last 2 years.

Dom: So you just tend to let them roam around the missionary?

LSK: yeah yeah yeah

Dom: And… how do people, the people who look after them, where do they get their knowledge to look after them?

LSK: well it's actually whatever knowledge, practically acquired knowledge you know? From their own background. That's with their only. We don't have any technically you have acquired knowledge. They're not technically trained people, those that look after them. Uhh, they're practically acquired knowledge from their, from looking after their own chickens, pigs, cattle at home.

Dom: So I'd like to talk to you know about what you do when one of your animals becomes ill

LSK: Uh we call the vet, that's what I told you yester… the other day also. His credibility I don't know actually how much he's really certified or not, I don't know. But since he was kind of… not appointed but he was being called here around this place when year out he was all the time, so we called him. And I have seen him giving single kind of medicines so whatever, according to the sickness of that time. SOS type, no? medicines. As per necessary drugs.

Dom: So for what reasons do you call out this particular person?

LSK: Sometiems they have this diarrhoea, loose motions, and sometimes they are not eating well and sometimes we see them in pain. All that time. Struggling to get up or something like that then they are informed because they are being watched. Those three ladies are like mummy to them. She learns how sick there, necessarily know that, ok? So then they call brother 'suchandsuch' animal is sick we need to call the doctor… vet doctor. So we give him a call and he comes and checks and he gives medicines.

Dom: And why do you choose to call out this man?

LSK: I think he was the man available in this area and he has got that little credibility that he is doing well. What he does, he does well and in that way we call. And so far no complaint from our part also. I mean there he came and treated, I saw them getting better so. And none of them died so so far so…! (laughs)

Dom: Any other reasons

LSK: No that's all I can think

Dom: Ok… So when he comes does he normally administer the medicines? Or… Does he normally administer the medicines?

LSK: Yeah yeah, himself. He brings the medicines, required medicines. See it's a kind of practical thing here I think. Whenever he's called, it's not that he does a prescription and we go and bring the medicine. So they bring all the medicines. They know generally, they're told, actually they enquire the symptoms of the sickness and accordingly they bring the medicines for the sickness and actually we pay them, medicines. And he administers first dose and then he'll tell those who are looking after, the concerned peoples. So you are giving the morning this much the afternoon that much and evening that much and like that. They're doses are being poured to them and they rightly feed them on time.

Dom: Ok, right ok. Umm, sorry just… So, are you aware of what products he tends to administer to the animals?

LSK: Simple type, I have seen him giving a simple type of medicine. Or not actually the name of the product I don't know but it's like the fever medicines he almost gives that type of (…?) only, and the diarrhoea, that kind of diarrhoea product, that's according to the sickness. He gives administrative medicines (…?) I've seen it. And some… between that… This one is a little bit of weak and all he'll give them every time and certain timings, ok you have to give that weak guy (??)

Dom: Yeah. And are you aware of the term antibiotic.

LSK: yeah yeah.

Dom: Are you aware of whether or not he prescribes these?

LSK: I have seen him prescribing (por-i-teen) for when they are having diarrhoea, something like that… think so, antibiotics. After that no… only for that reasons, otherwise no.

Dom: Ok. And has it… have you ever been aware of it not working?

LSK: That I cannot say because afterwards they are ok (laughs). So I think that it worked on that one but… not the others, I haven't seen them giving antibiotics all the time antibiotics, no. No misuse or abuse of the antibiotic, no, none. And we call him as per need only. Only he is very much needed, otherwise we have so many workers here, coming from village background. And they have their own way of treating them with herbs, generally herbs. From this leaves, that leaves they make medicine and try to give them. We try first this one. See these animals are also actually… if they treat them naturally, if they become ok they are more healthy. And we increase, like are giving much more allopathic medicine. All this drugs going in them, actually in a way we are making them dependent on that one you see? So we are aware of that, we try to treat them naturally with the knowledge of the common people, the local people ok? They have also… they were doing this before no? Before all that medicine came in, allopathic medicine came in, before then. So they were treated naturally before, so that we try. And if it is need then only we call.

Dom: Ok. Do you know what these natural medicines normally consist of?

LSK: I'm not that aware but they know that. Mixture of herbs and all that one, no? Some leaves, all that one.

Dom: So at what point would you then decide that you thought allopathic medicine was required?

LSK: Yeah, if we see the conditions not improving, deteriorating much more. So then we call.

Dom: Ok… So do you go to the same person or a different person for the medical needs of people?

LSK: people?

Dom: yes people

LSK: Oh we people? No no no, we don't work with the same people. Ok we work with the vet, no no.

Dom: Would you ever ask advice about human medical care from the man you call out for your livestock?

LSK: No, no no no. Why should we do that? He's the vet? No, no

Dom: What about the other way around? Would you ever ask about the medical care of your livestock here from someone that provides care to humans?

LSK: No, we keep it separate. They're two different… We have this vet, the vet is for the animals and all the physicians and doctors, are for the people.

Dom: And in your eyes, what do you see is the difference between human medicine and animal medicine?

LSK: For me, I cannot say exactly. I do not have that information myself ok? Because I have not done this one before, then this place. *Life history redacted* But I have seen in my one place that the animals are being treated before with these natural things. And later the medicines came in and all that one. But I have no knowledge about how, what kind of, this one drugs they are giving. Is it the same drugs we are having and they are also having, and what are the causes and all that one? So that I don't know

Dom: So could you explain why you don't… why you keep the human medicines to the people and the animal medicines to the animals?

LSK: It's a very obvious thing. The obvious thing is, for the animal medicines, from my knowledge… maybe the component is the same, whatever it is, for the fever and all. But the doses wise, they are heavier, they are much more this one. Larger physical structures, so there is more the drugs and quantities wise also. We need less quantity wise. Plus I'm not that good in medicines so I don't know but I think that human being's medicines are concentrating more on physical or human disease and also same, similar as animals' medicine is being product is concentrating on their disease so in that way the separations will be there.
