# Supplementary material for: “If It Works in People, Why Not Animals?”: A Qualitative Investigation of Antibiotic Use in Smallholder Livestock Settings in Rural West Bengal, India
Source: Antibiotics (Basel). 2021 Nov 23;10(12):1433. doi: 10.3390/antibiotics10121433 (PMC8698124; doi:10.3390/antibiotics10121433)
Supplement: Supplementary file 1 [file antibiotics-10-01433-s001.zip › Supplementary S1_ Interview Transcripts/Site 1/LK14 (site 1).pdf]

Code for Study - ‘If it works in people, why not animals?’: A qualitative investigation of antibiotic use in smallholder livestock settings in rural West Bengal, India: LK14, Site 1

Date: 26/07/2019

Location: Site 1

Interviewee: Livestock Keeper (LK)

Interviewer: Dominic Day (DD)

Translation: Somraj Das (SD)

Transcription: Sayak Manna (SM)

START OF INTERVIEW

D: Interviewer (DD)
B: Translator (SD)
J: Participant (LK14)

D: thank you very much for answering my question.
B: Thank you for giving us time
D: First I want to ask is what livestock you keep?
B: What livestock you keep?
J: Yes I keep.
B: what types?
J: These are indegenous cows. Only indegenous cows.
B: He only keeps cows.
D: How many cows?
B: How many?
J: I have 3.
B: around 3.
D: How do you house the cows?
B: House the cows?
D: Yeah how they housed?
B: So how do you keep them exactly?
J: We have rooms for them, in those rooms food is given, they feed on them, when the food gets over we refill them. We give grass, chuffs, roughages, grams and hay.
B: There is a cow shade for housing them and usually they feed them grass and some sort of things that keeps them healthy.
D: Alright, is it the same all year around?
B: Is it same for the rest of the year?
J: What? Food?
B: Yes!
J: Oh yes the food like this for the entire year.

B: This is same all year around.
D: Okay, and can he explain in details what he feeds them?
B: What do you feed them can you repaet?
J: Will I say it again?
B: Hmm.
J: I give grass, hay, roughages, cuffs, flour, potato skin. I boil them all together and make a slurry. 1 cauldron full of food, you can see, how do I feed them if you would come to my home.
B: Ok. He's saying that green grass, rice straws and some kind of wheat grass and grains that they usually feed them all the years
D: And does he add any products to the food.
B: Do you add any medicine to the food you give? (products and medicines are two different things)
J: No, no medicine.
B: Any medicines with the food?
J: Medicines? where will I get them from? Here in our area we don't get medicines.
B: He's saying no he doesn't add any kind of suppliment with the food. (But he questioned about meds not suppliments)
D: Could he explain for what purpose does he keep the cows?
B: Why do you keep them?
J: For our income! What shall we eat then? We keep them for our food. We sell milk and with that I buy hay and roughages for them and run our family.
B: He's saying for basic income, they usually acquire milk out them and they usually sell them and that's how they run their family financialy.
D: So do they use the milk for themselves?
B: The milk you get, do you use for yourself too?
J: For our drinking purpose? Yes of course, I do drink milk.
B: Yes, they usually use them for their own domestic purpose as well.
D: As well as selling them?
B: Yes
D: So whom do you sell milk to?
B: Whom do you sell?
J: To the local villagers of this area!
B: He;s talking about local villagers, they usually sell their product to, specially milk.
D: Ok, uhh.. Do you get other products from the cow?
B: What do you get from the cows other than milk?
J: The milk is Rs40/ Litres. Suppose I get 3 or 4 or 5 Litres. So 5x8 is 40. I will get Rs4000. If I spend 2000 rupees I will still have 2000 more. Because I have to buy hay, I have to buy food for the cows. After that whatever is left we use it in our family. We save it in the bank. When we have to buy hay we go to the bank, withdraw 5000-6000 rupees. Same is done when I have to buy rice for the entire season.
B: He's saying that, he sells on the litre basis. Supposedly he's selling 1 L of milkwhich is 40 rupees. And they usually calculate like that way. Basically they receive...acquire...generate money at the end of the month around 20-40 thousand (When this was said?) They have to reinvest in buys rice straws to feed them and the half of the money they save it.

D: Did you asked what other products do they getfrom the cows?
B: hmm. I did. What do you get other than milk?
J: We don't get anything other than milk.
B: Well they don't get anything except milk.
J: There's nothing other than milk. We get the milk, we sell them. May be keep 500ml for our personal consumption.
B: He's saying no, generally he doesn't get anything except milk. And sometimes they keep around 500ml for domestic uses and rest they sell.
D: And who owns the cows?
B: who owns it?
J: I own them.
B: He's the owner.
D: And who looks after the cow?
J: I do it. We both husband and wife take care of them.
B: (Answered rightly)
D: Do you get help from outside the family?
B: Do you get help from outside to maintain these cows?
J: NO. No-one.
B: No one.
D: Could you tell me, how you learn, how to look after the cows?
B: How did you learn to look after the cows?
J: I am keeping cows for ages. Our fathers keep them and now we are keeping them.
B: He's maintaing livestock for many years and he said his fathers and grandfathers were keeping the livestock as well.
D: Ok, and if he had a question to whom would he go to?
B: Who do you go to when you face a problem?
J: We go to the district or call for a doctor.
B: Generally if they have any problem or question, they generally go to the GP health centre. Otherwise there are much experienced doctors to whom they would prefer to go for livestock.
D: And for wht reason do they go to these people?
B: For which reasons you go to them?
J: When the cow is down with fever, have temperature, diarrhoea, then we go. When medicines given by town-people don't work we call for better doctors. Of course the cost exceeds, more than 600 rupees. But we have to do to save the cows.
B: They generally go for diseases like dysentery fever etc. That's why they prefer going to GP, if it doesn't work at GP they go for experienced doctors, which costs them around 500 rupees.
D: And for what reason do they prefer to go to GP?
J: I've done insurance too, but they gave us nothing, the Government cheated us. They came took pictures of out cows and promised to give us medicines every year for the diseases. But they never came even for once! I paid for insurance but it wasn't of any use!
B: (He explained it right) He's complaining about the system. Repeat the question?
D: My question was why does he prefer to go to the GP?
B: Why do you prefer to go to GP for your cows? You said you go to the health centres right?
J: Yes

B: Why there?
J: when we face problems we have to go. Will I stand and see one of my cows is dying? So I called the big doctor, he came, diagnosed, wrote me a bill and I just paid!
B: He's saying they sometimes go there. Because they can't sit and see their livestock dying. So they go there. If the GP practitioner prescribes some meds or injections, they usually pay the bill. They get the meds to keep the livestock healthy
D: And why do you choose to go to this person and not anywhere else?
B: Why do you go to him and not to anyone else? (He can't put forward the question right, he should be more specific)
J: What? At District? Yes, if there are minor problems I go to the district. They give meds and incase they never work we go for better doctors. Or call them here.
B: In general diseases they prefer to go to GP, if the problem is crtical they go to experienced doctors (made up answer)
D: Why do they first prefer to go to GP?
B: Why do you go to the district?
J: In district they give free meds. So we go there.
B: GP provides free meds.
D: Could he explain the role of GP person exactly?
B: against livestocks?
D: Just the role of GP person.
B: (confirming the question). What is the role of that GP person you go to?
J: There are doctors for all the areas who practice there, there they prescribe meds for cow, goats, hens for free. If there are other meds that can't be provided, they give us in writing and we purchase from outside. They never gave us any vitamins. Not for the cows! We buy them for Rs 250-300 and feed the cows. The cows are now pregnant, so we put the vitamins in rice starch and feed them. Becasue of which the milk production will increase and the calf will grow better.
B: Generally most of the doctors practice in GP, so they go there. After that he's complaining that they do not prescribe vitamins. And there's a calf around, he is big, you know and so they need vitamins and they are going to make in an organic way. Because GP doesn't prescribe any vitamins and all
D: Ok, and for reasons he would go to the more experienced?
B: Why do you go to the more experienced doctors?
J: We go to *(local town name redacted)* for the experienced doctors! There we ask the doctors to come with us here!
B: Why?
J: When the doctors come we have to pay them Rs 300 and separately for the meds.
B: Why do you go?
J: When we see the condition of the cow is real bad, then we go to the experienced ones! When the district doctors give up, we take to the experienced ones!
B: There are experienced doctors in *(Local town name redacted)* area, they charge them around 300 rupees. And when GP doctors can't handle the cases, they tell them to go to the doctors in Sharisha.
D: Okay, the doctors in *(Local town name redacted)* are Govt or Non-govt?

B: Are the doctors in *(Local town name redacted)* Govt?
J: Yes they are Govt doctors. They come in the off time. For an example after 4pm. And if they have to come here, then they take off, ride on their bikes, come here, treat and go off! They will also give the meds.
B: They are generally Govt doctors, they come here in their spare time (WHAT?) after 4pm. But in critical situation if you call them, they will come here, get the job done and go back.
D: The people of GP are govt or non govt?
B: (Repeated correctly)
J: Govt, all govt!
B: In GP there are GOVT too.
D: Ok, so could you ask him if he understands the term antibiotics?
B: Do you understand antibiotic?
J: YES!
D: So do the doctors often prescribe antibiotics?
B: Do the doctors give AB?
J: Yes when we ask them to give, then...
B: Well he says..
J: They give AB and also give meds for the worms that grow in the stomach of cows. They give immediately when asked for the meds. When a cow will deliver a calf, after 3 months from delivery one (he meant medicine/AB) will be given, again one will be given after 6 months and then once in every month.
B: Well they insist doctors (..?) for their livestock, then they prescribe it. Otherwise if there's a worm disease which happen in their intestines, if they complain then they will provide the meds as well.
D: Okay, So what are the most common reasons for prescribing AB?
B: (Repeated rightly)
J: AB is given when the cow is seen to be in serious problem.
B: When they see that there is a critical situation, they tell them to prescribe AB.
D: Could you elaborate what he means by critical situation?
B: What you mean by serious problem?
J: You can understand a cow's health just by touching its ears. If there is fever or not, you can get it quite easily. Then we seek the help of a doctor.
B: If you touch a cows ear you can get whether there's fever or not, in that case he can understand whether AB is required or not
D: Any other reason.
B: Other than fever, for anything else?
J: What?
B: Apart from fever is there any other reasons it is given for?
J: Yes! In that case we call for the district doctors, he comes and tells us what has happened or not. If he can't detect then he asks us to go for the experienced doctors! Of course they refer to us. So we call accordingly. I also have to pay the district doctors when he is visiting me here. If I go the district then things are free but if he comes here I have to pay!
B: Except fever, if there is more criticality they go to *(Site 1 name redacted)*, the practitioner come here, provide AB. If you go there they will not charge anything, but if they come here they

will charge you.
D: Can you explain the symptoms of other critical situations?
B: Other than fever, the diseases for which AB is prescribed, what are their symptoms?
J: The symptoms are, the cows will have teary eyes, will sweat profusely and will wobble.
B: There will be tears in cow's eyes, feeling numb and there is fever, those are the symptoms.
D: And is he often get to take his cows to the GP?
B: Can you often taken them?
J: No. I call the doctors here! I go there, talk to them, tell them about the problem, pay them and ask them to visit. Nothing works without money!
B: He generally call the doctors and pay charges.
D: Does he find it difficult to get hold of AB?
B: Do face problems in getting AB?
J: No.
D: Who administers the medicines?
B: Who gives the meds? After they give you the meds who gives them to the cows?
J: I give myself! If it's an injection, then the doctor gives it and they charge for it!
B: He's saying generally doctor prescribes and gives instruction, that 1 thing, otherwise he does it. If there's an injection involved then the practitioner generally do it.
D: Okay and if he is giving it do they normally give instructions about for how long and for how much? Do they?
B: Do they instruct how to give, when to give and how much to give?
J: They instruct everything in writing! They write everything in bengali and also on the medicines too. When to do and what to do.
B: (repeated right)
D: Okay and has he noticed that it has failed?
B: Have you seen the meds are not working?
J: yes!
D: And why does he think that it happened?
B: (repeated right)
J: Suppose I have fever, they didn't give the med I required, they might give something of much high power, in that case the cows will have more problems, things like this have happened! In that case we call the experiend doctor from *(town name redacted)* to treat! He will then give injection either 2 or 3!
B: (Explained the same)
D: And can he explain why the meds didn't work initially?
B: What do you think, why the meds didn't work initially?
J: Well, suppose I have some problem in my stomach, I can't figure out, the doctor disgnosed me and gave a med, but it never worked. So what will I do? I will go to a better doctor. he will prescribe either injection or tab and I will listen to him.
B: (explained right)
D: And could he explain how you believe AB work?
B: How do the AB work?
J: It works amazingly!! If they are given at right time, it works just fantastically! But bathing is ESSENTIAL. Everyday at least from head to back washing is necessary, whether you are having

meds or not! You have to wash the cows every day!
B: (Explained more or less correctly)
D: And why does he think that is compulsory?
B: Why cows needs to be washed everyday?
J: If not washed cows will get tired! In many homes they also have fans. And 6/7 buckets of water is essential! Se there the bickets are loaded already! Now we will give grass, hay and roughages and put them back to shade. They will eat everything. If they are not washed they will feel hot. It's good if you splash water on them daily!
B: He is saying the shower is NOT compulsory everyday! The cows will be exhausted, there will be much more problem. They usually consume more than 6/7buckets of water everyday, they need to fed everyday. So if you don't shower they will get annoyed and irritated.
D: Ok Does he ever ask advice about human health care from the people he goes to for vetenary (..?)
B: Have you taken advice for human health from the vets?
J: No.
B: Na-o!
D: Why not?
J: Well there's a problem, the cows will suffer. (I think he understood the question wrong, I think he thought of taking advice for his cows from human doctors)
B: He said there will be a problem, cow will get (..?)
D: Okay, and does he asked human doctors about the health of his animals?
B: Have you gone to the human doctors to take advice about your cows?
J: They cannot treat! You will get many doctors for humans but you can't find doctors for cows!
B: The doctors can't advice livestock, they generally see a difference in doctors.
D: So the doctors do advice about..
B: He believes that they cannot.
(Unnecessary talks)
D: Can you asked whether or not he ever used human meds on his livestock?
B: Have you ever used human meds on cows?
J: You can't give them to cows! If I am sick.. listen properly, I have fever, doctor gave me a med and it will get cured. If the cow is sick...if we will get 1 drop of medicine, a cow needs 6 drops of it. That's why there's a problem.
B: They do not usually apply human med on livestock, if human will need 1 dose, the livestock will be needing 6 dosages of the same product.
D: Can he explain me what he thinks is the difference in animal and human meds?
B: What's the difference you think between the meds of animals and people?
J: The difference in humans and cows is...umm...the cow's strength/power is much more than the human's. 1 drop will work for human but for the cow you need to give 10 drops.
B: (Explained poorly)
D: Is there any situation where he used AB for both humans and animals?
B: Has it ever happened that you sed the same AB both in human and animal?
J: You CAN'T give it. Why can't you give? I got an AB, I got 1 drop and it worked for me, in case of cows they needs 10 drops of the med, only a vet would know how much to give, a human doctor cannot say how many drops they would need. The one who gives meds to human will

NOT give to cows. The doctors for cows are different, they studied different things and learnt them, that's why for cows we need cow doctors!
B: (Explained rightly)
D: And when they get the AB from, is that different for the livestock and people.
B: AB are different for humans and animals, where do you get them from?
J: If I don't get them from the district I go to *(Local town name redacted)*. In *(Local town name redacted)* we have the office, I get it from there!We have to buy them too.
B: (Explained rightly)
D: For both animals and people?
B: (repeated right)
J: Separate medicines. For humans the power is less and for cows it is more!
B: Where you buy them from? Do you go to the same place?
J: No they are from different places. If meds of cows are given to man, then the man will die.
B: If you apply human meds on livestock the livestock will die and livestock med in human, they will die.
D: Ok, could he say the names of the people, who provide him drugs or vetenary health care?
B: Do you know the names of the people who give you health care facilities? The ones who give advice for cows health?
J: They are doctors from districts, from *(town name redacted)* area and *(Local town name redacted)*
B: It's all in GP, other than that *(Local town name redacted)*.
D: And what's the name of the place in *(Local town name redacted)*?
J : BD office
D: Okay brilliant, well thank you very much.
