# Supplementary material for: “If It Works in People, Why Not Animals?”: A Qualitative Investigation of Antibiotic Use in Smallholder Livestock Settings in Rural West Bengal, India
Source: Antibiotics (Basel). 2021 Nov 23;10(12):1433. doi: 10.3390/antibiotics10121433 (PMC8698124; doi:10.3390/antibiotics10121433)
Supplement: Supplementary file 1 [file antibiotics-10-01433-s001.zip › Supplementary S1_ Interview Transcripts/Site 1/LK15 (site 1).pdf]

Code for Study - ‘If it works in people, why not animals?’: A qualitative investigation of antibiotic use in smallholder livestock settings in rural West Bengal, India: LK15, Site 1

Date: 27/07/2019

Location: Site 1

Interviewee: Livestock Keeper (LK)

Interviewer: Dominic Day (DD)

Translation: Somraj Das (SD)

Transcription: Sayak Manna (SM)

Q: Interviewer (DD)
A: Interviewee (LK15)

START OF INTERVIEW

Thank you very much for answering my question.

Q: Which animal do you rear?

A: Birds and Cows.

Q: Which birds?

A: Pigeons and lovebirds.

Q: How many numbers of animals do you have?

A: I have 16 pigeons, 2 cows and one lovebird.

Q: How do you keep them?

A: I keep the pigeons in a 2”×2.5” cage, I feed them grains and provide medicines monthly like deworming at each 3 months interval. I also offer vitamins (e.g. recovit®) for both newborns and adult birds.

Q: What do you feed to the cows?

A: I offer the mixture of paddy straw, grasses, flour and extracted water obtained after boiling rice.

Q: Where do you keep the cows?

A: Here (pointing towards the shed).

Q: Do you keep the cows in same way as they are now?

A:Yes.

Q: Why do you keep these three kinds of animals?

A:I like to keep the birds. I love the animals.

Q: What do you get from them?

A:Cows give milk and dung.

Q: Do you get anything from birds?

A:We bred the birds and get chicks. We can sell them and earn money.

Q: What do you feed to your birds?

A:Wheat, pulses, mustard seed and also some supplementary medicine like vitamins.

Q: Do you mix any medicine with feed?

A: Yes, today I have given vitamins with feed.

Q: Can you show me the vitamins you use?

A:Yes.

Q: Okay. Not now, show me after the interview.

Q: Who is the owner of these birds? Who looks after these birds?

A:I am the owner and I take care of them. When I go to work, my mother looks after them.

Q: Does any outsider help you?

A:No, no.

Q: Do you people do the same job?

A:Yes.

Q: What are the other economic activities you people do?

A:We work in a manufacturing company (produce winter hand gloves).

Q: How much these livestock help you economically?

A:Economically it is very important but more than that I love to rear them.

Q: For what purpose you use the livestock products?

A:We sell the milk for feeding the infants. We use cow dung as a fuel for cooking purpose.

Q: From where did you learn rearing animals?

A: It needs lots of hard work to understand & learn. My mother got a cow from my grandmother, she reared it then…..I have learnt from her and also from my neighbours.

Q: What is the main source of information about livestock?

A:From neighbours.

Q: What do you do when livestock get sick ?

A: We give them medicines in different conditions like cold, inappetance. In case of birds we deworm the birds before laying starts and also give vitamins. In chicks we use liver tonic, energy medicine so that they grow well.

Q: What do you do when cows get sick ?

A: We provide medicines in the form of injections after consulting a Doctor. When cows come in heat, Artificial Insemination is done. We also give vitamin tablets. Sometimes we ourselves give these medicines without consulting a Doctor.

Q: When do you call a doctor?

A: When cow comes in heat, when cow is going to parturate, when cows get off-fed etc. People like us who rear the animals know the situations to call a doctor.

Q: What are the symptoms of the cows when you call a doctor?

A: When cows are not eating, dull and depressed, or showing the signs like mucus discharge from the vagina, bellows. We also call a doctor when cows show lameness, swelling in legs, ulcer in hooves and maggot wound in hooves etc.

Q: Do you know what medicines the doctors give?

A:We don’t know. The doctor comes with a syringe and medicine vial. They inject and go.

Q: Do they(doctor) come once?

A:When we call them, they come at once.

Q: Do they come many times or once?

A: If the cows are not recovered after giving medicines then we again call them for second time depending on the situations.

Q: To which doctors do you go?

A: Usually we go to the doctors of Gram panchayet(GP). Sometimes we call the doctors in our home too, if it is not possible then we go with our cows to the GP.

Q: Why do you go to the GP?

A:As it is free of cost.

Q: Is there any other reason to go to the GP?

A:No, no. If only cows are sick then only we go.

Q: Is there any situation when you take advice other than GP doctors?

A: Yes. When they don’t come then we go for other doctors. When it is Sunday or the doctors are on leave then we go to other doctors and we have to give them fees.

Q: Who are these other doctors?

A: They are not local. We call them and they come from outside.

Q: Why do you go to these doctors when GP doctor is available?

A: GP doctor is there but when it is Sunday we have to go for them. GP doctor is not available everyday in a week, they come to this GP for 2 to 3 days. If it is emergency and the GP doctor is not available then we have to call them

Q: Do you go to the same private doctor again and again?

A:Yes.

Q: Why?

A:Because he is the only one in this locality, so we have to go to him.

Q: Do you ask advice about your health to these doctors?

A: No, no. It's different.

Q: Do you ask advice to human doctors for your animals?

A: If the illness is mild like cough, cold sometime we go to the human doctor but otherwise if it is serious then we have to go to the veterinary doctors.

Q: What products do you get from human doctors?

A:They give the same medicine which they give to human.

Q: Did it happen that they gave medicine but it didn't work?

A: No, It worked.

Q: Do you know the term 'antibiotics'?

A: Yes, I know.

Q: Do the human doctors give antibiotics to your animals?

A:Yes, they give.

Q: Do you know the name of some antibiotics?

A: No, don't know much. When pigeon get cold we give O2 tablets , in diarrhoea we use terramycin .

Q: Why do you go to human doctors for livestock?

A: If it is normal cold, cough then it works. But if it is severe then we have to go to the veterinary doctors.

Q: Do you observed the difference between human and animal antibiotics?

A:No, no. As it works in human, it also works in animals too.

Q: Do you ever used veterinary antibiotics in human?

A: Ha ha(laughing…) . No, never.

Q: Why?

A: It’s not given

Q: Do you have any antibiotics in your house now?

A: Yes, I have antibiotics for birds. Here you see……
