# Supplementary material for: “If It Works in People, Why Not Animals?”: A Qualitative Investigation of Antibiotic Use in Smallholder Livestock Settings in Rural West Bengal, India
Source: Antibiotics (Basel). 2021 Nov 23;10(12):1433. doi: 10.3390/antibiotics10121433 (PMC8698124; doi:10.3390/antibiotics10121433)
Supplement: Supplementary file 1 [file antibiotics-10-01433-s001.zip › Supplementary S1_ Interview Transcripts/Site 1/LK16 (site 1).pdf]

Code for Study - ‘If it works in people, why not animals?’: A qualitative investigation of antibiotic use in smallholder livestock settings in rural West Bengal, India: LK16, Site 1

Date: 19/11/2019

Location: Site 1

Interviewee: Livestock keeper (LK)

Interviewer: Mathew Hennesey (MH), accompanied by Dr Indranil Samanta

Transcription: Soumen Samanta (SS)

MH- Matthew Hennessey

IS- Indranil Samanta

MH- Could you just repeat as because we are recording the type of livestock you keep here?

LK- Where are the other one? You have 3 people.

IS- He said he wants to see the river.

IS- So for how long you have been keeping cows here?

LK- I had been rearing cows for almost 40 years. The nearby all doctors know, my cows were the best. I used to get prize every year in cow show. I took good care of my cows so I got the high milk also.

IS- He used to get prizes from block level for best cow purpose. He is now having goats, no cattle.

MH: How many goats are there?

LK: At present 3 are there. One is male, 2 are female.

MH: What do you use them for?

LK: I am a poor man; I am a beggar (workless), doing no work. Just do little bit cultivation. I am having male goat which I use for natural service. (Mating to other female goats in his surroundings).

IS: That is his income source.

MH: What do you use the female goats?

LK: It gives kids; if they grow then sell it.

IS: Do you take milk from it?

LK: Yes.

MH: Do you sell milk?

LK: When I had cow then I used to sell it.

IS: no, not cow, from goats?

LK: Nobody buy it here. And that much milk is not obtained. The milk which is produced it is sufficient for their kids.

IS: So it is not sufficient for sell.

MH: What do you do when the goats become unwell/sick?

LK: There are doctors in our area, that *(Person's name redacted)* doctor (pranibondhu), and doctor in our Block (V.O), I visit them. Last time I was going to sell my 2 goats at 10,000 each for 20,000 rupees. But suddenly they got fever, and then I called *(Person's name redacted)* doctor. He came for 3 days and took 1200 rupees but couldn't cure. They died.

MH: Who is the another doctor?

IS: It's veterinary officer in BLDO office.

MH: If you have a problem in these goats now, who will the first person you will contact?

LK: our nearby *(Person's name redacted)* (the pranibondhu). And in Panchayet he comes at 11a.m. and goes at 4p.m. His house is far away. We call nearby *(Person's name redacted)*.

MH: How does he contact the pranibondhu?

LK: sometimes call by phone or go to his house and take medicine.

MH: Does the pranibondhu come here if you call?

LK: Yes.

MH: If you go to the block, does the block V.O comes here?

IS: Don't you go to the block?

LK: Yes, I go. If I call him (*(Person's name redacted)*) and he say that he already has gone to visit a case far away, and then I go (to block). Otherwise there is also a person named *(Person's name redacted)*(paravets). I also call him sometimes.

IS: Does the block doctor come here?

LK: No, no.

MH: Do you have to take goats with you if you go to the block or just you only?

LK: Yes I have to take it with me.

MH: How do you carry the goats there? How do you do that?

LK: If it is too much ill I take it by reserving a toto (a battery run three wheel van); otherwise by walk.

MH: If you go to the block V.O and if it need some medication; where do you get the medication from?

IS: Do you the block give medication or do you have to buy it?

LK: No, no. If they are having they give; otherwise if they are not having they write and we buy it.

MH: from where do you buy?

LK: if it is not available at *(local town name redacted)*then we have to go to *(local town name redacted)*. In *(local town name redacted)* there is a shop, had to go there.

MH: Did you ever go to the shop directly for medication before going to the pranibondhu or vet?

LK: No, suppose how much fever of goats are, our one goat is not eating properly from tomorrow, what they usually eat, they are taking less now. That's why after consulting the doctor whether it's fever or not we take medicine.

MH: Do you having any medicines here that you use to treat goats?

LK: As last time the goats died, some medicine was extra/left over; that is having now.

MH: Would you be able to show that medicine?

LK: He shows it.

MH: Okay. And would you use any of these medications now?

LK: No.

IS: (in a low voice) this is actually painkiller. Meloxicam.

MH: Okay. What type of livestock is there in this village?

LK: Here cattle, goat, poultry, duck.

MH: Is there anybody who keeps large number of livestock in this village?

IS: is there any farm here?

LK: no, no farm is here. Few people have some 8, 10-20 numbers of poultry. No large farm is here.

MH: How many cows are there in a house hold?

LK: I had previously 14-15 numbers of cows always. Now my health is not good, secondly, when I was going to take 'dikkha' (consecration) of God, they told if I obey this religion I can rear animal, can eat milk from it but can't sell the cows.

IS: In other house.

LK: In other house there are cows, some have 2, 5 or 6. My son has 2 cows, this house person has 4 cows. They are giving milk. Like this 2-4-6, they have.

MH: May be we can try to visit somewhere else having 6 cows, does he know where it may be?

IS: Is there anybody in your surroundings who is having 4-6 cows?

LK: You want to talk.

IS to MH: Will you visit now?

MH: May be afterwards, is that possible?

IS: Okay, he agrees.

MH: What do you feed your goats?

LK: grass previously; but not able to harvest grass now. So feed these grams's bran.

IS: Is these (grams' bran) bought from market?

LK: Yes, bought from market. This grams grinding is 25rupees and this wheat one is 23 rupees. (Per kg).

MH to IS: (inaudible)

MH: What are the main types of problem the goats get?

LK: Disease means sometimes diarrhoea, by feeding something or going our block; he give diarrhoea medicine and it cures. The 2 which died, I called *(Person's name redacted)*, it was 105° fever. He gave medicine that way, but it was not eating, fever also not decreased. Then in *(local town name redacted)*, our BDO office, we take the goats by toto (vehicle) there. There they said that fever is present; gave medicines. This medicine I bought from *(Person's name redacted)* and this one from *(local town name redacted)*I got (showing some medicine). They (block) didn't take money. But after taking it from there, it died.

MH: So if he goes to block office he need not pay for any medicine.

IS: If it is available, they give it free of cost.

MH: If you go to pranibondhu, what type of cost is involved with that?

IS: He said that he took 1200 rupees.

IS to LK: With fees and medicine?

LK: Yes, with fees and medicine.

MH: Was it for one visit?

LK: No, he came thrice. I am poor man, I can't do work much. He will get 150rupees still from me. I couldn't give him. I was dependent on those goats (income from services) but as it died I am in problem now.

MH: What is his full charge?

IS: How much *(Person's name redacted)* take from one visit?

LK: 150 rupees.

IS: Excluding medicine, 150 rupees.

MH: so medicine is more costly. Okay.

MH: Would any people use this medicine (to Human)?

LK: No, no.

(LK picks up a phone call)

MH: Would there be any time when people give human medicine to animal in this village?

LK: No, I can't say. I don't know.

MH: Thank you for telling about your animals. It is very useful.

LK: It’s okay. I stay here alone.

IS: Do you not have family?

LK: I have, they live in deeper area. My daughter-in-law lives in Kolkata. I stay here alone.

MH: Oh, great.
