# Supplementary material for: “If It Works in People, Why Not Animals?”: A Qualitative Investigation of Antibiotic Use in Smallholder Livestock Settings in Rural West Bengal, India
Source: Antibiotics (Basel). 2021 Nov 23;10(12):1433. doi: 10.3390/antibiotics10121433 (PMC8698124; doi:10.3390/antibiotics10121433)
Supplement: Supplementary file 1 [file antibiotics-10-01433-s001.zip › Supplementary S1_ Interview Transcripts/Site 1/LK17 (site 1).pdf]

Code for Study - ‘If it works in people, why not animals?’: A qualitative investigation of antibiotic use in smallholder livestock settings in rural West Bengal, India: LK17, Site 1

Date: 19/11/2019

Location: Site 1

Interviewee: Livestock keeper (LK)

Interviewer: Mathew Hennesey (MH), accompanied by Dr Indranil Samanta

Transcription: Soumen Samanta (SS)

MH: Matthew Hennessey
IS: Indranil Samanta

MH: What type of animals do you keep?

LK: You can see here, all are foreign which we call in Bengali as Jersey.

IS: So he is rearing 3 jersey cattle.

MH: What do you keeping for?

IS: What are the other animals do you keep except cattle?

LK: I don’t keep goats,

IS: Poultry?

LK: No.

IS: What do you keep the cattle for?

LK: It fulfils my milk need, and I have some lands, in that the cow dung is used as fertilizer, plus I can make others income.

I have lands, otherwise I have to sell the paddy straw; as I have cows they eat it. I don’t have to think for selling (straw).

MH: Do you sell milk to anybody else?

LK: Yes.

MH: Whom did you sell to?

LK: In *(shop name redacted)*.

IS: There is liquor factory nearby, *(shop name redacted)*, he sells there.

MH: Do you take the milk yourself there?

LK: Yes.

MH: What do you feed your cattle?

LK: some gram's dust, 5-7 things mix, maize dust, cakes etc. mixing them properly feed to them. Plus boiled broken rice, with that rice bran is added.

IS: Do you feed something extra from outside?

LK: From *(shop name redacted)* they told, now its price is 1000rupees per 50kgs. I used that feed before but I saw that not much profit is there. Now from *(shop name redacted)* the syrup (sugary) I take 10kgs and one nutrition /vitamin called it cows horlicks; 3jars they give in a month, 3 jars for 3 cows, they help as I give them the milk.

MH: Who looks after the cows?

LK: Me and my wife both of us look after.

MH: Do you pay anyone else from outside of the family to help you?

LK: No.

MH: What happens if your cows become sick or what do you do then?

LK: Call the doctor. I have phone, I call him.

IS: Which doctor do you call?

LK: for small problems nearby *(person's name redacted)* doctor whose house is just beside the road; and if any serious things happen then I go to Sarisha, at 246more one doctor is there- *(person's name redacted)* doctor, he is surgeon doctor.

IS: Government doctor

LK: Previously was in government, now he is retired but he treats now also.

MH: The local doctor, do you know what type of doctor he is?

IS: This *(person's name redacted)*doctor, is he government doctor?

LK: No, no, no. like *(person's name redacted)* doctor, he is ku (quacks)doctor. He takes less fees and also less cost of medicine. Previously used to call *(Person's name redacted)* now it's *(Person's name redacted)*.

IS: He is quacks doctor, his rate is low than *(Person's name redacted)*, the pranibodhu.

MH: Did you ever use the pranibondhu?

LK: Yes. We are rearing from childhood time, we used to visit *(Person's name redacted)*.

IS: previously he used to visit *(Person's name redacted)* but his rate is quite high.

MH: What was the last time you used the doctor?

LK: One month back.

MH: What was the problem?

LK: The cow's milk gland had been swollen suddenly. Just after the calving I saw the mammary gland swelled. That's why visited the doctor. First *(Person's name redacted)* could not understand then at last visited to *(local town name redacted)*.

IS: It cured?

LK: Yes, got well.

MH: Do you know what treatments the local doctor gave and what the *(local town name redacted)* doctor gave?

LK: Then I have to bring the prescription.

IS: Can you show us?

LK: Yes, in home it is.

IS: It will be good then.

(LK shows the prescription).

(MH observes the prescriptions, IS comment both the prescriptions is of same doctor -*(Person's name redacted)*, the retired vet. IS thinks that local doctor didn't give any prescriptions. MH agrees)

MH: where did you buy the medicines from?

LK: from *(local name redacted)*.

IS: What is the shop name?

LK: It's their shop, his son made the shop.

MH: Did you do any treatments regularly with the cows when they are not sick?

LK: regular means the ointment which you can see there, I use it when mosquito bites in teats. (To prevent it)

MH: Who give the treatments to the animals? Who give the different treatment to the cows?

IS: Who give injections to the cows?

LK: Called *(Person's name redacted)*.

MH: Did you pay the local doctor (*(Person's name redacted)*) money to do that?

IS: *(Person's name redacted)* also had to be given money?

LK: yes, of course, had to give money. No work without money.

MH: How much you have to pay?

LK: If he comes minimum 50rupees. And If I call *(Person's name redacted)* I have to give him 150rupees.

MH: How much do you pay to the *(local town name redacted)* doctor?

LK: 100rupees if I give he takes.

MH: That doctor does he comes here or you have to go to *(local town name redacted)*?

IS: Does this doctor comes here if you call?

LK: Yes.

MH: Does he charge same when he comes here?

LK: little bit more and I have to give the toto (vehicle) fare. If I go there he takes 100rupees and if he comes he take 200rupees and vehicle fare.

MH: Did you ever use human medicine to treat the animals?

LK: No, no.

Yes, sometimes like I have allergic tablets, id I see allergic swelling in cows then I feed 4 instead of 1. In human it is 1. And if I see diarrhea, in shop the human diarrhea medicine 500mg, I give it 2000mg means 4 tabs for cows.

MH: What medication do you use if the animal has sudden diarrhea?

LK: ‘enterozyme’, ‘metrzyl®’ (Metronidazole).

MH: Where do you get it from?

LK: from local shop on road.

MH: how many days you give it for?

LK: sudden, if emergency once or twice. Then call the doctor. Emergency, emergency.

MH: Are there times when people take these animal medications?

LK: No. It’s dose is high. Suppose if I use 5rupees medicine here, in cows it will be within 4rupees. Suppose in human 4 medicine cost 4 rupees, but in cows 3rupees medicine needed. But if it is emergency then I use that. And in cows high dose needed. But in human not so high dose needed.

(LK shows one medicine which was used on mammary gland abscess. And tells this time one serious conditions happened, the mammary gland hardened. Shows Mag Sulf powder for hot fomentation).

MH: What did you do if this treatment not works?

IS: Did this cure your animal?

LK: Yes.

IS: If it was not cured then what would you do?

LK: Again I have to go.

IS: He is saying that there was no injection in this prescription but the other prescription had injections. That time I called *(Person’s name redacted)* to push these injections.

(The LK is arguing about the chick and goat distributions from the block)

(MH wants to take photo for documentation and ask something to IS)

MH: Okay, Thank You.
