# Supplementary material for: “If It Works in People, Why Not Animals?”: A Qualitative Investigation of Antibiotic Use in Smallholder Livestock Settings in Rural West Bengal, India
Source: Antibiotics (Basel). 2021 Nov 23;10(12):1433. doi: 10.3390/antibiotics10121433 (PMC8698124; doi:10.3390/antibiotics10121433)
Supplement: Supplementary file 1 [file antibiotics-10-01433-s001.zip › Supplementary S1_ Interview Transcripts/Site 1/LK18 (site 1).pdf]

Code for Study - 'If it works in people, why not animals?': A qualitative investigation of antibiotic use in smallholder livestock settings in rural West Bengal, India: LK18, Site 1

Date: 21/11/2019

Location: Site 1

Interviewee: Livestock keeper (LK)

Interviewer: Mathew Hennesey (MH)

Transcription: Soumen Samanta (SS)

MH: Mathew Hennesey
SS: Soumen Samanta

MH: Thank him for agreeing to talk to us.
MH: I will ask him some questions about the types of animals that he keeps here.

SS: What types of animal do you rear?
LK: Only cows.

MH: How many cows?
LK: 5-6.

MH: How long do you have cows for?
LK: from my grandfathers' time. More than 30-35years.

MH: What do you use the cows for?
LK: if cows are there, as we can't take milk buying it, so we can take milk from them. Second think is that few fuels we get from cow dung and can sell it some amount.

MH: What do you do with the milk?
LK: sell it and take in home also.

MH: What percent do you sell of the milk?
SS: How much it gave milk?
LK: 1,5kg (litre) around, not same in all day, it depends on their eating.

SS: How much do you sell of it?
LK: 1.25litre we sell and 250ml kept for home.

MH: Where do you sell the milk?

LK: Milkman takes it and supply to sweet shop 'Anup' of *(shop name redacted)*. He comes and draws the milk from cows and takes it with him. Someday 1litre, someday 1.5litre.

MH: Do they come every day?
LK: Yes, everyday.

MH: What do you feed the cows with?
LK: fresh grass, buy paddy straw and it is given,

MH: Do you give them anything else?
LK: No, no.

MH: What types of problems do the cows get?
LK: problems means fever then I call babu (indicating the homeopathic vet with us). Then, like human has many problems cows also face many problems. This babu cures him.

SS: What symptoms do you observe? Not eating?
LK: yes, not eating, standing silently; then I call this *(person's name redacted)*and he tells "I am coming"; comes and tell this happens. One cow was not coming in heat for 1 year then I called this doctor and he visited and gave homeopathic treatment. I don't call others except them (*(person's name redacted)*).

MH: Do you treat with any other people?
LK: No, no. this one doctor always. Also there are some in surroundings but I don't believe them. It's not satisfactory. If I call him he tells that within 1 or 2 hours he is coming.

MH: Why do you not satisfied with the other doctors?
LK: satisfy means they are not like my mind;

SS: Do they can't cure?
LK: No, not that they can't cure.

SS: then?
LK: With him (this doctor) we have good relation; money matters like what I give he takes but they not reduce any money. Like if medicine is of 50rupees, he doesn't takes his visit, what he takes from others I don't know but from me he does this.
Sometimes if medicine is of 200rupees and their (Other doctor) visit is 100rupees total 300rupees. And babu (this doctor) take only 200rupees of medicine not take that fees. He never takes his fees. There are doctors also in his (*(person's name redacted)*) village and in our villages too. But they are not satisfying.

MH: Do the people ever use human medicine to treat their animals without speaking to the doctor?
LK: No, no.

MH: Would any people ever take the animal medicine for themselves?
LK: No.

MH: Why not?
LK: It’s not done (not usual).
Whether human medicine cam ne used in animal or animal medicine can be used in human I don’t know. Doctor can tell this.

MH: Do you know what antibiotics are?
LK: No, I have not heard the term.

MH: Do you know any name of the medicine that is commonly used for treating the animals?
LK: No, no.

MH: you take directions of the doctor?
LK: the doctor writes, we have not studied that much; the doctor write in paper and we go to take the medicine. He told when to feed them.

MH: Do you buy medicine from the doctor or do you buy from the store?
LK: Doctor writes and we go to take; otherwise if medicines are present with doctor he gives.

MH: Which do you prefer to do?
LK: if doctor is not having then he writes, if he is having then he gives.
Tells this medicine is not present, you go to the shop for it as early as possible. Then I go.

MH: thank you. Do you have any questions for us?
LK: what I will know?

(person’s name redacted)(the homeopathic vet): If you want to know anything?
LK: you are asking, what is the reason for it?
MH: we are trying to understand how antibiotics are used.

SS: you have not heard the term antibiotics. Antibiotics are given in case of infections. It is a medicine. Now whether you use it or not, how much you use it; we wanted to know that.
LK: No, I don’t know it. We don’t use it.

SS: they didn’t hear the term, whatever happens they consult to this doctor.

MH: Do you want to know any more to explain about the projects?
SS: As he not heard the term I think that much knowledge he is not having.

SS to LK; Sometimes it happens that these antibiotics are used rampantly, then one time it not works, resistance develops. To know that properly, is our work. May be in your area it is working but in some places it’s not working, so we are collecting these data.
