# Supplementary material for: “If It Works in People, Why Not Animals?”: A Qualitative Investigation of Antibiotic Use in Smallholder Livestock Settings in Rural West Bengal, India
Source: Antibiotics (Basel). 2021 Nov 23;10(12):1433. doi: 10.3390/antibiotics10121433 (PMC8698124; doi:10.3390/antibiotics10121433)
Supplement: Supplementary file 1 [file antibiotics-10-01433-s001.zip › Supplementary S1_ Interview Transcripts/Site 1/LK19 (site 1).pdf]

Code for Study - ‘If it works in people, why not animals?’: A qualitative investigation of antibiotic use in smallholder livestock settings in rural West Bengal, India: LK19, Site 1

Date: 21/11/2019

Location: Site 1

Interviewee: Livestock keeper (LK)

Interviewer: Mathew Hennesey (MH)

Transcription: Soumen Samanta (SS)

MH: Matthew Hennessey

SS: Soumen Samanta

LK: Livestock Keeper

MH: Thank him first.

MH: What types of animals do you keeps here?

LK: only have cows.

MH: How many cows?

LK: One adult cow and 2 calves.

MH: What do you use the animals for?

LK: so that milk can be consumed and rest for selling.

MH: How much would you keep for yourself and how much for sell?

SS: How much milk do you get? And among that how much you keep and how much you sell?

LK: If 1kg (Litre) milk is obtained, 250ml we keep, rest part we sell it.

As it is deshi (native) cow, so not much milk is obtained.

MH: Who do you sell the milk to?

LK: Here at *(Local town name redacted)* one sweet shop is there, *(shop name redacted)*. Their people come and draw the milk as we can't draw the milk. They draw and give us and rest they take with them.

MH: Oo, they do the milking?

SS: yes.

MH: So the guy who was milking outside?

SS: You were there?

LK: I was restraining the calf.

SS: So who was milking?

LK: Their people.

MH: They come every day for milking?

LK: yes, everyday.

MH: Do they pay him money?

SS: Do you pay him money for that?

LK: No.

MH: So he doesn't get money for the milk?

SS: So no money he takes for milking?

LK: I don't have to give money for milking, the milk price they give me at the end of the month.

MH: What do you do if the cows become sick?

SS: If he can't come then what do you do? (SS heard wrong question)

LK: we can't do milking properly, after the calf takes it, my mother try it. So not much milk obtained that time. If they do milking it is 1kg but if we do it is 250-300ml.

MH: Okay, what happens if your cows become sick?

LK: then the doctor who came (the homeopathic vet), we call him. Then he come and see; and give medicines and it cures.

MH: What happened when last time the animal get sick?

SS: When problem in cows was seen last time?

LK: Last year once, and not this year but insemination was done.

SS: this doctor does that?

LK: No, he doesn't. Another doctor is there. He did it.

MH: Who is the other doctor?

LK: His house is at *(site 1 name redacted)*, he stay at Kolatola.

SS: Is he paravet/pranibondhu/..?

LK: He did all type of training, he did vaccine also.

SS: Is he connected with G.P?

LK: That I don't know whether he is connected with G.P or not but he does vaccination everywhere.

SS: AI also?

LK: hmm, give vaccine and also give medicines in fever. And this doctor who came here (homeopathic vet) gives only medicines.

MH: If your animal is sick, whom would you prefer to call first?

LK: call them both, if this doctor say he is coming within sometimes he come; and if he say that he is not able to come that time then I call that doctor (another) and he comes.

MH: If both one is available which one would you prefer?

LK: I first call this doctor (homeopath one); if he can't come then I call the other doctor he comes.

MH: Why do you call the homeopath first?

LK: this doctor (Homeopath) is nearby, and that doctor (Other) is busier, he gives vaccines so he is not available all time. I have both the doctors' number. If my cows get sick if one say that he can't come today then what can happen we don't know so (keep both number)

MH: Do you know what types of treatments he gives?

LK: No, I don't know that much, the doctor gives. And if he says this medicine twice a day or 3-4 times a day, then I give that accordingly. And give his visit. (money)

MH: D you know what antibiotics are?

SS: Did you hear the term antibiotic?

LK: yes heard, antibiotic means than will prevent the disease progression.

MH: Where do you get the medication from?

LK: The doctor keeps medicines and if not having then he instructs us to go the kolatola market and I get it from there.

MH: What would you do if the cow is not getting better?

LK: mostly the cow cures by the medicine of this doctor. It didn't happen that our cows not get well in his hand.

MH: so always works.

SS: Yes.

MH: Would any people gets the animal medication themselves when they are sick?

LK: No, no, no. we don't know whether it could be taken.

MH: Would they ever use the human medication to treat the cows?

LK: What doctor writes we buy it.

SS: Is that from human medicine shop?

LK: Yes.

MH: Did you ever go to medicine shop directly without speaking to the doctor?

LK: No, no. How can we know what happens to cow? What medicine they will give by guessing it we couldn't be able to understand.

MH: The person who comes here to milking, does he ever bring medication?

LK: No, only for milking.

MH: What do you feed the cows with?

LK: paddy straw and grass.

SS: Any cakes?

LK: No our cow not take that properly if you mix them. From where we bought the cows, perhaps it was not used to feed those there. Only grass and straw. The grass is cut and mixed with straw. And the 'fan' (water after boiling rice) also given.

MH: Do you have any question to ask us?

LK: what can I ask?

MH: Okay, thank you very much.
