# Supplementary material for: “If It Works in People, Why Not Animals?”: A Qualitative Investigation of Antibiotic Use in Smallholder Livestock Settings in Rural West Bengal, India
Source: Antibiotics (Basel). 2021 Nov 23;10(12):1433. doi: 10.3390/antibiotics10121433 (PMC8698124; doi:10.3390/antibiotics10121433)
Supplement: Supplementary file 1 [file antibiotics-10-01433-s001.zip › Supplementary S1_ Interview Transcripts/Site 1/LK2 (site 1).pdf]

Code for Study - ‘If it works in people, why not animals?’: A qualitative investigation of antibiotic use in smallholder livestock settings in rural West Bengal, India: LK2, Site 1
Date: 01/07/2019
Location: Site 1
Interviewee: Livestock Keeper (LK)
Interviewer: Jean-Christophe Arnold (J-CA)
Transcription: Debanjan Debnath (DD)

I: Interviewer (JCA)
P: Participant (LK2)

START OF INTERVIEW

I: What animals do you keep?

P: I have chickens, goats. Nothing else. I used to have cows, I sold them off, then I got the goats.

I: How many chickens and how many goats do you have?

P: 8 chicken. 9 goats.

I: Why do you keep goats?

P: Name?

I: What do you keep the goats for? I mean, what do you get from them?

P: I just love them, so we keep them. We like raising them. Like, some people like to keep cows for the milk. Like that I like goats a lot.

I: So, you keep them because you like it?

P: Yes, because I like it, also in case there's a problem, I sell them for money.

I: And chickens?

P: Eight of them.

I: Why do you keep them?

P: My daughters love to eat chicken. I don't eat chicken, I daughters, my son eats them. We are raising them for the meat.

I: Other than the meat is there any other reason you keep the chickens for?

P: In time for festivities we would butcher one and eat it. I don't eat.

I: Is there any other reason?

P: No.

I: How important are the goats for the family economically?

P: Not much.

I: Who owns them?

P: Me. These are all mine.

I: How are the goats fed?

P: We leave them in the field, they eat there. If it's raining, I go cut the grass and bring it here. Like you can see there. (points out to the cut grass)

I: Do you give them anything to help them grow?

P: No, I don't give them anything, it's just the grass. If they get sick, we have to give them tonics and medicines. vitamins.

I: What sort of tonic?

P: They have their own doctors, like we have our doctors, they have their doctors. We go to the doctor, we tell them, the doctor would give medicines. We feed the medicines to the animals.

I: Do you know any of the names of the medicines?

P: I have them. (Goes to get medicines). This medicine is for the goat, when it has diarrhea, we give it to them.

[The medicine identified is ciprofloxacin hydrochloride. There is one tablet remaining from a cut blister pack with three blisters. It is indicated on the packaging that it is for veterinary use]

I: Do you know what antibiotics are?

P: I don't know.

I: The medicine that you showed us what would you call that?

P: That is called a tablet.

I: Where did you get it from?

P: There's a veterinary doctor at *(Local town name redacted) [referring to the Site 1 Livestock Development Assistant]*. I got it from there.

I: Where is *(Local town name redacted)*? Are you talking about the GP office?

P: (Mumbles) Yes, the GP office. There's the "anchal" (GP), he sits just around there.

I: When do you go to the doctor for them?

P: When they fall sick, I go and get it. (...) You have to carry them in your lap.

I: You take them there?

P: You carry them in your lap. I used to carry their (pointing to the kids) mother in my lap back and forth, then it died. I raised the kids by feeding them powdered milk mix with water.

I: Do you go to anyone else except for the doctor when they get sick?

P: No, No, we only take them to the doctor.

I: So, you only take them to the doctor you mentioned?

P: Yes

I: Why don't you go to other places?

P: Yes, we also take them to another doctor *[referring to the Pranibandhu]*. Where else would we go? There are just two doctors, one sits at the "Anchal" other one is him.

I: The medicines you keep for the family, did you ever use them on the animals?

P: No, no!

I: Not ever?

P: We shouldn't give human medications to animals.

I: Why not?

P: It won't suit them. If we take their medicines, it won't suit us. If they take our medicines, it won't suit them.

I: Can you explain why it won't suit?

P: How I will say that! We have a different stomach; they have a different stomach. that's why it won't suit.

I: Has there been any situation where you have taken medicines meant for the animals?

P: No.

I: For what problems in the animals do you look for treatment?

P: They don't eat grass when they are sick. The way we don't find anything tasty when we are sick, the same way they won't like the grass. After they are given medicines, they like to eat again.

I: Are there other symptoms other than reluctance to eat?

P: No!

I: After you get the medicines, who feeds them?

P: I do. The way you feed milk to a baby with a spoon. We mix the medicine with water and feed it to the goats the same way.

I: What do you do when the chickens get sick?

P: If they have white stool, we get medicines to feed them.

I: What's white stool?

P: They die, if they excrete white stool. We need to give them medicines then.

I: Where do you get the medicine from?

P: *(Local town name redacted)*. Who give medicines for them (the goats), he has them too.

I: Is this the GP vet?

P: No, He's there too. But that's his own shop. He learned to practice himself.

I: You go to this other vet?

P: Yes, I go there. He learnt to practice by himself, and now he gives medicines.

I: Is it in the GP office?

P: Yes. He sits in a place beside the office.

I: Is it inside the office building?

P: No, outside! There's one in Site 1 (GP).

I: And?

P: There's another vet, we go there.

I: What's the name of the doctor?

P: I don't know.

I: What is the name of the place?

P: *(Local town name redacted)*, he comes to *(Local town name redacted)* in the morning, and *(Local town name redacted)*in the evenings. He has shops in two places.

I: Is it a doctor for human or animal health?

P: Just them (animals).

I: And that's where you take the animals?

P: Yes, if you call him, he also comes. If it gets difficult, we call him, and he comes.

I: So usually you go, and sometimes if there's a problem you call him to come. Is that it?

P: Yes.

I: It is the same person treating both the goats and the chickens?

P: Yes.

I: Do you treat them in any other way?

P: No. If their body feels hot, I give them a shower. If it's really hot and sunny outside, like we'd feel hot, they would too. So, I take them to the pond and give them a shower.

I: Where are the goats kept?

P: They just stay there. (pointing ahead to the cowshed) If it's raining, I cover it up. During the winter I cover the floor with straw.

I: The baby goats, as we can see are in the house...

P: They just stay here; we don't have a problem with that. We sleep with them in the bed. they (the baby goats) are very good. We don't feel disgusted by them. My husband also does the same, sleeps with them in the bed. There are jackals who will tear them apart if we leave them in the field. That's why we have to watch over them. After they feed, we bring the back home.

I: Where do you keep the chickens?

P: They stay in the shed, we cover it and put bricks on them otherwise jackals would come and take them. unless they are covered up well, jackals would prey on them.

I: Where is this shed for chickens?

P: it's just there. (points out to a small space surrounded by bricks)

I: Is it inside or outside the house?

P: It's inside. Everything is inside the house! They stay here (A space inside the main building of the house covered with fishing nets) until 10-11, then they are put outside to the other space.

I: Who takes care of the animals?

P: I do, my daughter-in-law, my daughter does whenever anyone gets time.

I: Do you have any specific roles?

P: No!

I: How did you learn to look after the animals?

P: We grew up with cattle and goats. We used to cut grass from childhood. My father's house in Howrah Zilla. We used to cut grass there. My father used to have 15-20 cows. We would cut grass, and straw. My father gave one cow, it didn't have a baby, so we sold it off.

I: Does anyone give you advice on how to treat the animals (or when to take them to the doctor)?

P: I do it myself, no one advices me. I don't ask anybody.

I: Where do you go to when anyone in the family gets sick?

P: We go to the Diamond hospital.

I: Is there anywhere else?

P: No! It's a government hospital, we go there. There's also a government hospital in *(Local town name redacted)*.

I: Have you ever asked for advice for animal health from these doctors?

P: No. When they are sick, we go to the doctor and ask for medicine. We check by the ear if they have fever, if the ear is hot, the doctor will give medicine if needed.

I: Which doctor are you talking about?

P: The animal doctor, the vet.

I: And have you ever gone to a human doctor for animal health problems?

P: No, not to the human doctor. when we go the animal doctor, they tell us to feed this or that to help them gain power, etc.

I: The interview is finished.

END OF INTERVIEW
