# Supplementary material for: “If It Works in People, Why Not Animals?”: A Qualitative Investigation of Antibiotic Use in Smallholder Livestock Settings in Rural West Bengal, India
Source: Antibiotics (Basel). 2021 Nov 23;10(12):1433. doi: 10.3390/antibiotics10121433 (PMC8698124; doi:10.3390/antibiotics10121433)
Supplement: Supplementary file 1 [file antibiotics-10-01433-s001.zip › Supplementary S1_ Interview Transcripts/Site 1/LK20 (site 1).pdf]

**Code for Study -** ‘If it works in people, why not animals?’: A qualitative investigation of antibiotic use in smallholder livestock settings in rural West Bengal, India: LK20, Site 1

**Date:** 22/11/2019

**Location:** Site 1

**Interviewee:** Livestock keeper (LK)

**Interviewer:** Mathew Hennesey (MH) accompanied by Pabak Sarkar

**Transcription:** Soumen Samanta (SS)

MH: Mathew Hennessey
PS: Pabak Sarkar
LK: Livestock Keeper

LK: We have poultry in our house.

MH: How many goats and chicken do you keep here?

LK: total?

PS: Yes.

LK: 10 chickens and 5 goats (3 kids, 2 adults)

MH: Where do you get the chicken from?

LK: from local, *(Person’s name redacted)*.

PS: From where do they come?

LK: (inaudible)

PS: So there are some businessmen, they bought it from them.

PS: How many years ago?

LK: 1year back.

MH: What do you use the chickens for?

LK: In home for eggs and we can’t buy to eat so sometimes we eat them.(for meat)

MH: How many eggs do you get each day?

LK: sometimes 2, sometimes 3 or 4.

MH: Do you ever sell eggs or chickens?

LK: No, eat them in home. We rear 2 to 3 birds (indicates they rear small number of birds). We give these children the boiling eggs. We are poor people. Can we be able to buy meat?

PS: They have large family so they use it for their own consumption, they can't buy the chickens. It's expensive so they just grow and sometimes have it.

MH: How often do you eat the chicken?

PS: How many times in a month..

LK: as we wish, if it lay eggs every day we eat it every day.

PS: And chickens?

LK: It can't be eaten below 6months age, unless it is 2-2.5kgs. When they grow up we eat them.

PS: How many times do you eat these homes's chicken in a year?

LK: more than 4-5times.

MH: What do you use the goats for?

LK: we sell it.

PS: How many do you sell in a year?

LK: not more, sometimes kids are born; sometimes die, like thus 1-2 are sold, this time it is 1.

PS: They generally sell the goats 1-2 in a year.

MH: Who looks after the animals?

PS: Who looks after the poultry?

LK: we do it.

PS: The ladies do it.

PS: And goats?

LK: We. (women)

MH: How do you feed them?

LK: Cut grass from the field, allow them to graze.

PS: In home anything?

LK: ‘fan’ (The extra water after boiling rice).

PS: and how to the chickens?

LK: Rice bran, grind rice, boiled rice, wheat.

MH: So you don’t buy any extra commercial feed for the chicken?

LK: No, we have in home ‘fan’; and the rice bran we buy.

MH: Do you keep all of the chickens in this house here?

PS: except this place (indicating the shade), where do you keep them?

LK: In back side of home also.

MH: Why you have made house rather than roaming around?

PS: do you not allow the chickens to roam around?

LK: yes, less; in afternoon for ½ to 1 hour.

PS: Why?

LK: if they roam they can eat something from outside;

Another woman: if they take dirty things/ rubbish they can die. That’s why we not allow more to roam around.

MH: What type of problems do you have with the chickens?

LK: when chalky white diarrhea occurs then our ‘murgiwala’(the guys who sold these chickens to the LK) has told us to feed one tablet then it will be cured. One O2 (ofloxacin) tablet they told; those from whom we buy chicks, they told us.

MH: Are there any common problems?

LK: No, if that diarrhea occurs we feed that tablet it gets cures.

PS: All times it cures?

LK: Yes.

MH: Do you know what type of medicine O2 is?

LK: diarrhea medicine, orange color tablet, when chalky stool occur and the birds get dullness, there is doctor, *(local town name redacted)* doctor; from there we take it.

PS: It's an orange color tablet; they get it from one of the IPs here who actually treats human. They told me the name of the IP and I have that information.

MH: Do you know it's an animal medication or human medication?

PS: Is it used for poultry or human?

LK: Poultry.

PS: So not given to human?

Another LK: no, they give it to poultry and also when human get diarrhea they give it to human also. Other 2-3 medicines also they give, among them it (O2) remains. The medicine is good.

MH: Do you go to anybody else when the chickens are unwell?

(someone ask what is happening here)

PS: Do you visit anybody else except this 'murgiwala' Ramprasad doctor?

LK: No,

Another LK: if goats have problems then we call a cow, goat doctor who goes through this route. They give vitamins, bolus; give injections.

When it felt sick, gave a tonic.

PS: Do you have the tonic? And do you know the name of that doctor?

LK: No, we don't know the name; we took it from *(local town name redacted)*.

When a goat was limping, (he) gave this tonic. (Shows the tonic) many medicines were there but that has finished. Many medicines he gave of 300-400rupees.

PS: Did he write anything?

LK: No, we went and told my one goat is limping; gave deworming medicines and told to feed everyone.

PS: Where in *(local town name redacted)*?

LK: in block.

PS: In government block office?

LK: No, near block, there is one doctor of cows and goats, he sees cows, goats there.

PS: Is he looking like a handicapped?

LK: Don’t know, my husband went.

PS: So her husband went to get the medicine from someone in *(local town name redacted)*.

MH: Did you get the medicine there after speaking to the roaming doctor here?

LK: No, we didn’t speak here. We don’t get him (roaming one) all time and previously we took medicine from him (one in *(local town name redacted)*) and it cured so my husband went there this time. This doctor roam in many places, we could not get him in phone; he goes during parturition of cows. As he is not available my husband went to *(local town name redacted)*.

MH: What types of problems do the goats get that they have to go for treatment?

PS: So she said the problem was in walking, it was limping.

MH: Are there any others problems do the goats get?

PS: She was telling the doctor gave some deworming medicines too.

MH: What do you do if the poultry and goats not get better in the treatments?

PS: Does it happen that after giving medicine the poultry not get better?

LK: No, this medicine is for goats.

PS: No, this is for goats; and for the poultry time..

LK: no, no, it cured. If we feed that medicine it get cure. No poultry has been died. The medicine is good. If it’s given they are fine.

PS: And in goats?

LK: if you feed them the medicine it gets better.

PS: No such occurrence that the medicine not worked?

LK: No.

MH: Do you know what antibiotics are?

(PS ask MH to start with another question and he proceeds)

PS: How often the diarrhea problems happen in a year?

LK: In poultry?

PS: yes.

LK: During summer it more happens.

PS: 2-3 times in summer?

LK: Yes.

PS: And this walking problem in kids?

LK: 1 week after it's birth. Then my husband went there. Previously in adult male goats once happened and after feeding medicines it cured. Why it not gets cured I don't know.

(someone asked MH and PS to introduce them)

MH: So they don't know what the medication is that they give to the goats?

PS: She doesn't know.

MH: Can you ask them what antibiotics are?

PS: During loose motion, diarrhea one antibiotics are given. Did you hear the term 'antibiotics'?

One LK: yes, given during illness.

PS: What type of illness? In headache?

LK: In headache, in stomach pain,

PS: They are guessing mostly and it is used for both human and animals.

MH: Do you have any questions to ask to us?

LK: What to say, what will you do with this information?

MH: We will use this information in our projects of the villages in this area to have an understanding..

(LK laughs as they do not understand MH's voice)

PS: our work is that, when poultry or cows get ill antibiotics are used sometimes; now it is seen that even after using that it is not getting cure. So whether that is also happening here or not, we are looking for that. To understand this we are taking information from *(site 1 name redacted)*. in some areas it is seen that this problem is happening.

LK: yes, last time the medicines in limping goat not worked.

PS: he is saying last time the male goats was limping the medicine worked well but for kids goat it not worked well.
