# Supplementary material for: “If It Works in People, Why Not Animals?”: A Qualitative Investigation of Antibiotic Use in Smallholder Livestock Settings in Rural West Bengal, India
Source: Antibiotics (Basel). 2021 Nov 23;10(12):1433. doi: 10.3390/antibiotics10121433 (PMC8698124; doi:10.3390/antibiotics10121433)
Supplement: Supplementary file 1 [file antibiotics-10-01433-s001.zip › Supplementary S1_ Interview Transcripts/Site 1/LK21,22,23 (site 1).pdf]

Code for Study - 'If it works in people, why not animals?': A qualitative investigation of antibiotic use in smallholder livestock settings in rural West Bengal, India: LK21/LK22/ and LK23, Site 1

Date: 26/11/2019

Location: Site 1

Interviewee: Livestock keeper (LK) (group of 3)

Interviewer: Mathew Hennesey (MH), accompanied by Dr Pablo Alarcon, Dr Ana Mateus, Dr Meenakshi Gautham

Transcription: Soumen Samanta (SS)

A: Ana Mateus
P: Pablo Alarcon
M: Meenakshi Gautham
MH: Mathew Hennessey
R1/R2/R3: Livestock Keepers

A:What type of livestock you keep here?

R: I have three types of domestic animals like cows, goats, chicken.

M: are you both from the same family?

R1: No. We live separately.

M: Then where do you live?

R1: I live nearby

M: what about the others?

R2: They go to field for farming

M: Do, we want to visit your house too, after we finish the tea we will visit your home. Will you show us your animals?

R2: It is 2mins away from here. Okay.

M: what type of animals you keep?

R1: I have cows, goats & chicken also.

M: who takes care of these animals?

R1:Me & my wife.

M: How many animals are there?

R1: 2 cows, 8goats, 2 chicken

P: & yours?

R2: I have 2 cows, 2goats & 1 chicken

M: How many family members are there?

R1: we are 5 people in the family.

M: who are they?

R1: My wife, daughter in law, grand children, son.

M: & Yours?

R1: wife, son, daughter in law, grand children total five people are there.

Ma: Do anybody keep fish here?

R1: yes.

R2: each of the families, own a pond. Most of villagers raise fish themselves.

Ma: How many ponds they have?

R1: we are three brothers. Our pond got devided (pukur kata hoyeche).

Ma: What type of fish they keep?

R: we have Rohu fish, Pangas, Tengra, pona, these are local varieties.

M: What is the various roles of this pond in their life?

R: for fisheries and people wash utensils and clothes in the pond. Take baths.

P: Do you also use the water for farming in the fields?

R2: No. Very little. In March April, we use the pond water for vegetation.

M: What agriculture do you have?

R1: Now we are growing cucumber, ladies fingers, long bean etc.

M: How do you look after the food of the fish?

R1: we use mustard husk and floor to feed them. The floor what they use for their own consumption, they use the same for the fishes.

Ma: do they do anything more to manage the fish?

R1: Nothing more than feeding them.

M: How many fishes are there?

R1: We do not sell these fishes we just catch these for our own feed.

M: What is the quantity of the fish they get & how consume a day?

R1: We do not keep records. If anyone or guests come to our house or on special occasion or on festival in the village we catch the fish for own purpose of the quantity is more than we need then the rest of amount (excess) will be sold to outsiders.

Ma: What do you do if the fishes get sick?

R1: There is one powder called cuorine. If the fish get affected with insects then we use cuorine. For cleaning the water we use Potas, Chun.

M: From where you get this cuorine?

R1: From shop. We mix it in a tub with water and spread it over the pond.

A: Which shop?

R1: the shop from where we get the cow's food, presricides etc. Sukhdebpur. They keep food for cows, fish etc. Animal medicine, insecticide etc.

Ma: why do you use all these?

R1: fish get affected with insects. They face problem in gills 'kanko'. Then we use cuorine.

If the water become dirty the fish start floating then we use calcium carbonate to clean the water. We catch the fish, check them whether they get insects or not if they do then we use cuorine & Potas (potassium permanganate). There are two kinds of potas use for plants and fish gills. For the fish we mix it and use it with water it becomes blue color. It looks like salt.

A: Do you notice other kind of illnesses in fish?

R2: No no.

R3: The fish become very thin & soft. They have infection (gha) on their skin.

A: What do you do then?

R3: The shopkeeper give us medicine and tell us to use them . They guide us .

R2: We go to them & describe the problem and symptoms that our fish has this this... Then they give us medicine and tell us to use them.

A: Do they give any medicine for this kind of problem?

R1: No. They only give potas.

M: Do they do anything to prevent diseases?

R1: No. If we see the fishes start floating then only we use these.

R3: People use potas even before anything happens to prevent the water to get dirty.

Pa: what about the other people?

R1: They do the same thing.

A: Do you have any sample?

All: No we don't have. We use it and throw away the packet. It comes in packet.

M: When is the last time you use it?

R1: About 1year ago. There is no disease noticed recently in fish.

R3: We mainly use in April May , When the water level get down. In rainy season, all are good.

Pa: Apart from the shop, do you you also visit any doctor or someone else for consultation or treatment?

R1: No. For the fish only visit the shop. *(Person's name redacted)* at *(local area name redacted)* who has same kind of shop, villagers go to him also. For agriculture, insecticide and fertilizer some Representatives come here but not for fish.

Ma: For the cows, goats and chickens if they feel unwell what do they do?

R1: There is a doctor (vet provider) for the cows. He visit us if we call him. He gives the cows Vitamin or other medicine whatever required.

Pa: What type of provider he is , can you explain?

R1: *(Local town name redacted)*Doctor. *(Person's name redacted)*doctor and *(Person's name redacted), (Person's name redacted).*

A: Are they formal provider?

R1: No no... Not all of them are formal.

R3: one is Baun doctor, he provides both allopathy and homeopathy medicines.

A: Are they only treat animals or also humans?

All: cows..

Pa: How many are there in this village, I mean in *(Site 1 name redacted)*?

All: many three. *(Person's name redacted)* and *(Person's name redacted)* and Baun doctor.

Pa: what is the difference between them?

R2: we don't know about any difference. People choose who they prefer. They prefer the doctor whom people can get better result. All used to call *(Person's name redacted)* earlier but he is now high demanding in terms of fees and also we are not getting good results. So we prefer the surgeon doctor now. *(Person's name redacted)* sir who is attached with *(Local town name redacted)* block development office. But now he has retired.

A to pa: three informal providers in *(site 1 name redacted)* and one formal doctor attached with Block development office.

A: what do the other villagers do?

All: they do the same. They call three of them.*(Person's name redacted)* also there

M: Do they come on calls or do they also do the rounds? What kind of services they provide?

Pa: what kind of services they provide?

R3: Like I called *(Person's name redacted)*. The cow has swelling in hudders. He first spend 2000rs on one practitioner but the problem came back then he called another practitioner he also gave him some medicine and injection mostly then again the swelling came back after 2days. He was fade up as he spent around 10,000 rs for the cow but the problem was coming back and back.he called homeopathic practitioner from another village called Baun doctor. He came and gave homeopathic medicine for one week and told him to report him after 2days. After two days the swelling was half. So then he said continue for the week. Then he came again saw the cow then he continued the medicine for two months. The medicine costs him around 60rs per week.

Pa: So the homeopathic practitioner treats only the animals or human also?

All: yes. Only for animals. Actually we are not sure. He tired onhis own wife and she got cured.

M: Do you have those strips of the medicine?

R3: yes. I can show you. I have the box and also the medicine which didn't work on the cow, I have that leftover medicine also.

Pa: How do you get the medicine?

R1: Doctor give us. We have to buy them from doctor. The informal providers dispense. Only *(Person's name redacted)* (formal one) writes prescription then we will buy it from shops.

A: From Where do you buy all these?

All: vet shop in *(local town name redacted)*we buy it from them.

Pa: Do you go to the pharmacy directly and ask for medicine?

All: No we only go with prescription. But the shop keeper give the medicine directly if we explain them the symptoms.

M: Do you remember the medicines given by the pharmacy owner?

All: No. All they tell us that give this medicine for that & that days and time... We don't know names.

A: Can you show us the prescription?

R3: I have prescription and I have left over medicine.

Pa: when do you need formal provider?

R1: whatever convinient to us we consult one of them. We consult the nearby one. If that person can not solve the problem within two to three days then we consult another provider.

Ma: Do they call the provider to come or usually they bring the animals to the provider?

P: what they just told now the informal providers usually come on call and the formal one also come on call but he also give medicine over phone by explaining symptoms.

P: How often he comes?

R1: whenever we call them. But we need to keep approx 1000rs for the treatment. This is his minimum treatment cost.

M: Do this includes medicines?

R1: It depends. Sometimes 1500 or 1000. *(Person's name redacted)* gives medicine within 200-250rs. Less cheaper.

Pa: Then why do you call Jofar sir as he is expensive?

All: He is the best doctor as he is surgeon.

M: In the 1000rs, for how many days he gives medicine?

All: medicine for 4-5days. Injection for one time.

Medicine For three days. It depends on the symptoms. If the condition is serious then he would provide medicine for longer days like absesbut for the fever he will give medicine for lesser period.

P:. Can you give example how will it cost for fever for bloating?

R3: 200-300rs. For fever he will provide 500rs medicine for 2-3days and for bloating it will be 150rs medicine for 2times only. For abscess(infection on leg) it will charge 1500-2000rs for long time upto it get cured including injection. Formal doctor gives medicine for 4-5days medicine along with injection.

Pa:. Which condition you treat by your self you do not require any suggestions from provider?

All: Never.

M; Whether they treat the animals by their own without consulting anyone as they keep leftover medicine also for the same symptoms.

R2 : if we notice the Same thing has happened(for same symptoms) then we use the leftover medicine and wait. If it doesn't get cured then we consult the provider.

R3: But I don't keep medicine. I complete the full dose else it can occur again. There might be some problem.

M: what do you do if the chicken fall sick?

R1: In summer we notice particular symptoms like white stool then use pottasium powder

We do not usually consult provider if we then they give the potassium only. We mix itin the food and water and feed them. The chicken die after 2-3days in case of whitish excretion.

X: who gives it?

R3: you can get it from *(Person's name redacted)* doctor.

Pa: Do you use antibiotics which you generally use for yourself do you also use it for treating the animals by yourselves?

All: No. We never use our own medicine.

We don't have any idea about the dosages. But when the goat has loose motion then also we ask the doctor what can be use to treat the goat.

M: what do you do then?

If the goat has loose motion then we sometimes give metrozile, enteroqunol very rare and if it is not so serious. For initial care we provide these medicine.

Then we consult doctor.

M: Do you give antibiotic to the animals?

All: No.

A: Do you know if the providers give antibiotics or not?

All: no. We don't know about antibiotics.

Pa: Whether they consult Other villagers/ farmers/neighbor or with leftover medicine?

All: No. We will consult provider. We don't use other's leftover medicine.

Pa: whether the human medicine is given to the livestock?

All: Only in case of loose motion. Metronidazole.

Pa: whether they use same compound for both human and animals? The animal drug to human?

All: no. The animals medicines are always in high dosages. The human medicine costs lesser than animals like the medicine for loose motion for human costs 2rs while tablet for cow one tablet costs 40-80rs. Why would we use that?

Ma: How often they fall sick?

All: Once or twice in a year. No body can say exactly.

My cows haven't been fall sick even once. For the goat I need to consult the provider 7days ago

My goat fall sick 15days back.

R3: My cow has narrow milk part so I used clove to get it Bigger after consulting with the cattle firm then the cow got infection and it started bleeding.

Ma: How much it costs for the treatment?

R3: person who comes for injection he charged 100rs perday for 7days. Medicine costs for 1500.

Pa: who came for injection?

R3: Baun Doctor came to give injection.

Pa: Can we get the contact number & show us the prescription and left over medicine even for the fish.
