# Supplementary material for: “If It Works in People, Why Not Animals?”: A Qualitative Investigation of Antibiotic Use in Smallholder Livestock Settings in Rural West Bengal, India
Source: Antibiotics (Basel). 2021 Nov 23;10(12):1433. doi: 10.3390/antibiotics10121433 (PMC8698124; doi:10.3390/antibiotics10121433)
Supplement: Supplementary file 1 [file antibiotics-10-01433-s001.zip › Supplementary S1_ Interview Transcripts/Site 1/LK3 (site 1).pdf]

Code for Study - ‘If it works in people, why not animals?’: A qualitative investigation of antibiotic use in smallholder livestock settings in rural West Bengal, India: LK3, Site 1
Date: 01/07/2019
Location: Site 1
Interviewee: Livestock Keeper (LK)
Interviewer: Jean-Christophe Arnold (J-CA)
Transcription: Debanjan Debnath (DD)

I: Interviewer (JCA)
P1 and P2: Participants- 2 members of the same household present (LK3)

START OF INTERVIEW

I: What animals do you keep in the house?

P: Just that one! I just have one cow, nothing else!

I: Who is the owner of the cow in the house?

P: I am the owner.

I: What's the reason for keeping the cow?

P: I am very interested in raising cows. My family doesn't approve of it. Despite my interest, they don't let me keep them. They don't like raising poultry so, I can't keep them. I have kept the cow for social reasons, and for the fuel (cow dung).

I: Do you get anything else from the cows (apart from the cow dung)?

P: Apart from cow dung, we get the milk.

I: What do you do with the milk?

P: We make "chanda" or "sweets"...

I: How do you use the milk?

P: We don't use it at all, we give it to the milkman they come and take it. we keep just enough for ourselves.

I: How important is the cow for the household?

P: The cow is very important. Cow can be very useful to us. We might need the dung, or the urine, we need various things from them.

I: How important are they economically?

P: Some would require one glass, some people would require half glass.

P2: If there are marriages or religious ceremonies the dung and the urine is required. They would probably come and take it home.

I: They are important economically?

P: Yes, Yes. During Pujas.

P2: For Kali Puja (Name of an Indian goddess), for marriages.

I: What to you feed the animals?

P: My cow eats more grass, and less "Khol Bhusi" (Rise Husk, Chaffs). We can't always afford to buy "Khol Bhusi"(Rise Husk, Chaffs). Cows in different area eat different types of "Khol Bhusi"(Rise Husk, Chaffs). In the villages we bring some grass. So, they eat more grass.

I: What's "Khol Bhusi"?

P: Like after you get oil from the coconut, you get "Khol". Just like that there's this other "Khol".

P2: If you feed the cow rise husk and chaff, the cow gives more milk.

I: Do you give anything to the cow to help the cows grow?

P: Yes, Vitamins. this and that. And we give deworming medicine and such to the calf when it's growing up.

I: Any other medicines?

P: No, nothing like that

I: What sort of medicine is fed to the animals?

P: When the calf is sick, we call the doctor, the doctor sees the cow and gives the cows the medicines it needs.

I: Who is the doctor?

P: We call [*name removed- Pranibandhu]* or *[name removed- Animal Development Volunteer].*

I: In which situations will you go the doctor?

P: When they get impatient to get impregnated. Or if there's a problem during the pregnancy: during these times we call the doctor.

P2: (Indistinct)

I: Could you please repeat that?

P: When we see that the cow is pregnant and there's some problem, then we call the doctor. If we see that there's a problem during the pregnancy, we'd call the doctor. Or when the cow has fever, for reasons like these we call the doctor.

I: Do you go anywhere else?

P: No, we don't go anywhere else!

I: Why don't you go?

P: I have a Jersey cow. It runs around a lot so we can't take it with us anywhere. Since I am alone, I get scared to go by myself. If we go to the GP, it will cost us less, but the cow would be disturbed by the cars and start running around, that's why we don't take it anywhere. Now when we see that we are helpless, we call the doctor home for check up!

I: Do you know which medicines the doctor gives?

P: We don't know which medicines the doctor gives, I feed the medicines in however amount the doctor asks us to give, we don't give more. If It's a calf the doctor will ask to give half of the tablet, sometimes the full tablet. This is the way we give the medicines.

I: Is the doctor for human health or animal health?

P: Animal health

I: Does the doctor give the treatment himself?

P: Yes. He has come many times.

I: have you ever gave the treatment to the animals yourself?

P: Yes, we also go to other doctors in the village. The woman, who had come, also has four/five cows. There a doctor who visits their house.

I: Have you ever given (fed) the medicines to the animals?

P: Yes.

I: Have the doctors ever given medicine to the animals?

P: No!

I: Do you have these medicines here?

P: No! The cow is grown and is going well now.

P2: When the cow gets sick, we call the doctor, the doctor himself gives to us the medicines, we don't get the medicines ourselves and feed it.

P: We throw it away after use.

P2: We'd have to twice or thrice a day for few days as the doctor would ask, then done. If there were 4-5 cows there would perhaps be some remaining, since it's just one...

I: For what problems would you take the cow to the doctor?

P: Sometimes for fever, stomach issues. When different problems occur, then we'd call the doctor.

I: Do you remember the names of the medicines you used?

P: No, from when the cow had worms/mites, there's this oil.

I: Have you ever heard the word antibiotics?

P: Yes, there was this camp in Thakurtala, the doctor had mentioned.

I: What do you know about it?

P: It's been a long time, I have forgotten. How will I remember it!

I: Where do you keep the cow?

P: There, at the front. Just at the entrance from the main road. The room with thatched roof.

I: Who looks after the cow?

P: I do!

I: Does anyone in the family help?

P: No, none of them can go! the cow attacks them.

P2: It's mother would never attack, but it does! I feel very scared.

I: Does anyone outside the household come to look after the cow?

P: No, It's all me!

P2: Starting from cutting the grass, cleaning the floors she does everything by herself, she takes good care of the cow.

I: How did you learn how to look after the cows?

P: We had cows in my father's house. I learnt from there.

I: Is there anywhere else where you would get advice on how to take care of cows?

P: No, they don't come anymore.

P2: They (the camp) had come once a long time ago. They told us how to take care, how to keep them.

I: Where do you normally get the medicines?

P: As I said, when we call the doctor, he gives the medicines! We don't give other medicines.

P2: We don't buy it from outside, the doctor gives it. When you need tablets, they give the tablet, when you need injections they would come and give it. We don't have to go anywhere else.

I: Have you ever gone to a human health provider for animals?

P: No, we always call the animal (cow) doctor.

I: Have you ever asked the human doctor for advice regarding the health of the animals?

P: No!

P2: The doctor would sometimes suggest this and that.

I: Does he ever prescribe medicines?

P: No

I: Is there any medicine that is used both in humans and animals?

P: No, we would never use human medicines on animals. The cow-tablet is this big, and the tablet for human is just this small. Of course, there's a difference.

I: You said they look different, is there any other differences that you might know?

P: My cow is healthy.

I: I mean the difference between human and animal medicines.

P: The animal medicines have higher power.

I: Has there been any situation where the household has used medicines meant for animals?

P: No! We keep it separately. We don't bring it at the living space. Lest the childen mistake them for something else. It's dangerous.

I: Which doctor do you go to for the family?

P: For the family we go to Dimond (the hospital). There are a few minor doctors in Dewantala, we go there sometimes in case of minor fever or things like that.

I: Where does this other doctor stay?

P: The doctor from the Hospital?

I: No, the other one, where you go minor treatments.

P: Just here at Dewantala, where the bazaar is.

P2: There's Doctor [name removed].

I: Is it inside the village of Sriphalberia?

P2: No, It's not in Sriphalberia, it's just at the marketplace.

I: Has there been any situation where you have gone to a human doctor for animal treatment?

P: No!

I: Is [name removed- Pranibandhu] is the one comes to the GP?

P: Yes, yes!

I: Other one is [name removed- animal development volunteer]?

P: Yes.

I: When you go see a doctor for the cow, do you decide yourselves when you are supposed to see a doctor?

P: When I see the cow is too sick, the doctor comes to the house when we call him. He treats me cow at my home.

I: Do you decide for yourself when to see the doctor? Does anyone else give you advice?

P: Yes. No, no.

I: Which doctor (veterinarian) comes to your house?

P: Mostly [name removed- pranibandhu]. He is the one to take care of the cow the most.

I: Who is the GP vet then?

P: We don't go to him.

I: Why not?

P: As I told you My cow is too aggressive, so I can't take it anywhere.

I: Who are these doctors then?

P: The ones that come to our house are outdoor doctors. One of them stays in Kalatala. The other one, in Bhabanipur.

I: The interview is finished.

END OF INTERVIEW
