# Supplementary material for: “If It Works in People, Why Not Animals?”: A Qualitative Investigation of Antibiotic Use in Smallholder Livestock Settings in Rural West Bengal, India
Source: Antibiotics (Basel). 2021 Nov 23;10(12):1433. doi: 10.3390/antibiotics10121433 (PMC8698124; doi:10.3390/antibiotics10121433)
Supplement: Supplementary file 1 [file antibiotics-10-01433-s001.zip › Supplementary S1_ Interview Transcripts/Site 1/LK4 (site 1).pdf]

Code for Study - 'If it works in people, why not animals?': A qualitative investigation of antibiotic use in smallholder livestock settings in rural West Bengal, India: LK4, Site 1
Date: 01/07/2019
Location: Site 1
Interviewee: Livestock Keeper (LK)
Interviewer: Jean-Christophe Arnold (J-CA)
Transcription: Debanjan Debnath (DD)

I: Interviewer (JCA)
P: Participant (LK4)

START OF INTERVIEW

I: How many animals do you have?

P: I have one cow, another calf who will be impregnated this year and another small calf.

I: Who owns the animals?

P: Now, it's me.

I: What's the main reason you keep the cows?

P: The main reason is cow dung, the milk for us to drink and also to sell.

I: How important are the cows for your household?

P: For the household we need more cows, but I don't have the place.

I: At present how important the cows (however many you have) are for your house?

P2: It's quite important. I'll tell you how. The cow is a source in income for us. Our family depend on the money we get from selling the milk. We also get fuel. There are many other ways that the cow is useful.

I: Economically how important are the cows?

P: We are fishermen. The cows are the primary source of income.

I: Do you have other sources of income?

P: We work as laborers in the field. We also go the sea for fishing. I just returned today, I came back after four days, the weather was bad.

P2: We have to work hard the entire day to make the ends meet.

I: What do you feed the animals?

P: We mainly feed grass and straw. Husk and Chaff would be good for the cows, we'd get more milk, more profit, but we don't get the money for it. We don't have the money.

I: Do you ever give the cows anything to make them grow?

P: No. Husk can Chaff would help, but we just feed grass and straw.

I: I'm just repeating the question, do you give the cows anything to help them grow?

P: No, we don't give anything, we give rice water, vegetables, grass, husk and chaff, we don't get anything from the GP. We boil some of them with flour.

I: Have you ever given medicine to help the cows grow?

P: No. Once they put vaccine. They put a number on the cow's ear. That's all they offered from the GP.

P: They just put a number on the cow's ears. Only that was given to us by the GP. Nothing else. Nothing to feed them.

I: Where are the animals housed?

P: Right there (pointing to the back) we used to put it to the other side, but then it was broken and made to the other side. We need a better cowshed.

I: Who in the household take care of the animals?

P: She does most of the work. (pointing to the wife of the owner)

I: Why's it she looks after the animals?

P: We all do, but she does it more.

I: Does anyone have specific roles for the cows, or does everyone do everything? Like a specific work will have to be done by someone specific.

P: Most of the work is done by my other only, because we don't have a lot of work otherwise. So, she goes back and forth from the field.

I: Does anyone else outside the household help the animals?

P: No, one does. If the cows fall sick, we go the GP for medicine. Or worms.

I: You get medicine from GP, you said. What do you mean by GP?

P: By Anchal I mean the doctor who sits there.

P2: We made the GP through election.

I: The doctor sits inside Panchayat office?

P: Yes.

I: For what reasons would the cows get treatment?

P: for worms, skin diseases, diarrhea. Or if the cows are reluctant to eat.

I: Do you get to anywhere else for the treatment apart from GP?

P: Sometimes, to other doctors.

I: Who are they?

P: [name removed- Pranibandhu]. We don't know the name of the doctor who sits in the GP office.

I: Whom do you see inside the village?

P: There's just one [name removed- Pranibandhu]! There's one who comes from outside, when we call him, he comes.

I: What's he called? Is it [name removed- animal development volunteer]?

P: Yes.

I: Do they also treat humans?

P: No! Just cows, goats, chickens.

I: Which doctor would you prefer the most?

P: [name removed- animal development volunteer] Doctor!

I: Why?

P: I will tell you why, when a cow needs to be impregnated, they put an injection to get it done. Or if the cows have worms, diarrhea, or doesn't want to eat we go to him, we like him. We go to the GP; the Artificial Insemination doesn't happen in one go. We have to, then, go again.

I: Do you know which medicines are given to the animals?

P: No, we don't know. When the doctor gives an injection, he gives it himself. We mix tablets with water and give it to the animals.

I: Do you have any of these medicines here, old ones or cases.

P: The cow had rashes on the skin, we have that medicine. Do you want to see it? After one of my cows gave birth, I didn't realize, but it used to scratch itself against the tree. We call it "Mayer Doya". It had sores on the skin.

I: Where did you get this from?

P: From [name removed- Pranibandhu].

I: You bought it from [name removed- Pranibandhu]?

P: Yes.

I: What do you understand by the word "antibiotics"?

P: For example, if we have pain and eventually there's an infection, isn't that what antibiotics are?

I: You said when you call [name removed- Pranibandhu], he himself gives the treatment if it's an injection, and you have to give the medication if they are tablets. Is it the same for all other doctors?

P: Yes. We go to doctor he says whether we have to give the medicine once or twice. If we need to feed it to the animals, we do it. In case of injections the doctor does it.

I: Are there any medicines that you both used in animals and in the family?

P: No.

I: It never happened? (confirming)

P: No!

I: Why don't you use animal medicines in humans?

P: Look, what we feel is that our diseases are diagnosed by human doctors and they know which medicine to give, the same way we rely on veterinary doctors to treat the animals. That's why we don't take animal medicines, neither do we give our medicines to animals.

I: Do you know which medicines are given to the cows?

P: The cow doctor would usually give tablets.

I: Are you usually told the name of the medicine to address it?

P: No, we are not told the name of the medicine. They just tell us when and how to give the medication.

I: Where do you go when someone in the family is sick?

P: We go the hospital.

I: Which hospital?

P: there's one at Dewantala, and the bigger hospital is in Diamond Harbor.

I: Both of them are govt. hospitals?

P: yes.

I: Have you ever asked for advice on animal healthcare to the human doctors?

P: No. No.

I: For example, in case of emergency?

P: In case of emergency, we call the animal doctor that we mentioned. We don't go to the human doctors. We call [name removed- second Pranibandhu] and he comes.

I: [name removed- animal development volunteer] is a vet? So, you don't seek advice from Human healthcare providers?

P: No, the work gets done by the vet. If there were a situation where the medicines they are giving didn't work, we would then seek medication.

I: The doctors that you mentioned, do you know if they are qualified?

P: I don't know about that. [name removed- Pranibandhu], I think has a degree. The GP vet is qualified, but we don't know his name.

I: When your cows get sick whom do you go to first?

P: We first go to our village doctor. [name removed- Pranibandhu]!

I: Who is this Village Doctor?

P: Just talking about the same doctor! If there's an emergency, we see other doctors.

I: Why do you go to [name removed- Pranibandhu] first?

P: He lives nearby.

I: Have you ever used medicines which is used in the family for animals?

P: No.

I: Can you say why?

P: We are scared if the medicines might harm the animals.

I: What difference do human and animal medication have according to you?

P: The difference is that humans can talk, we can express which problems we have. but the animals can't talk. We can feed the medicines, but they won't able to tell us if the medicine is causing a problem. So according to our understanding if we give the right medicine to the animals it'll work, unlike us they can't speak. We are scared that human medicines might harm animals.

I: If the cows are sick, has the been any situation where you do not seek treatment, and why?

P: We would want to treat the cows whenever it has some temperature or has diarrhea.

I: What I mean is, has there been any situation you don't seek treatment?

P: If we don't get this doctor, we get medicines from the shop and get the animals treated.

I: Which drug shop are you talking about?

P: There are medicine shops, when we tell them, they give the medicine to us. There are shops that are meant for veterinary medicines. We go there. When the cow gets sick, we always get treatment, we never just leave it like that.

I: Which pharmacy do you buy the medicine from?

P: There's a pharmacy at *(Local town name redacted)*.

I: What is the name of the shop? And can you directly buy medicines from the shop?

P: When the doctor has prescribed a medicine and the doctor doesn't have it so we go to the pharmacy to get the medicine.

I: oh, you go get it after the doctor prescribed it?

P: Otherwise where will we get it?

P2: Also, if the animal has a certain condition and we go to the pharmacy and say it, they will give medicines accordingly.

I: Do you get both human and animal drugs at this pharmacy?

P: No, just animal drugs.

I: Who's the owner the shop?

P: No, we don't know that.

I: Can you describe where the shop is?

P: *(Local town name redacted)*, 246 more. We just know about this one, there's nothing else we know of.

END OF INTERVIEW
