# Supplementary material for: “If It Works in People, Why Not Animals?”: A Qualitative Investigation of Antibiotic Use in Smallholder Livestock Settings in Rural West Bengal, India
Source: Antibiotics (Basel). 2021 Nov 23;10(12):1433. doi: 10.3390/antibiotics10121433 (PMC8698124; doi:10.3390/antibiotics10121433)
Supplement: Supplementary file 1 [file antibiotics-10-01433-s001.zip › Supplementary S1_ Interview Transcripts/Site 1/LK5 (site 1).pdf]

Code for Study - ‘If it works in people, why not animals?’: A qualitative investigation of antibiotic use in smallholder livestock settings in rural West Bengal, India: LK5, Site 1
Date: 01/07/2019
Location: Site 1
Interviewee: Livestock Keeper (LK)
Interviewer: Jean-Christophe Arnold (J-CA)
Transcription: Debanjan Debnath (DD)

I: Interviewer (JCA)
P: Participant (LK5)

START OF INTERVIEW

I: What animals do you keep in the house?

P: I have cows.

I: How many cows do you own?

P: As of now, I have seven.

I: How owns the cows?

P: I am.

I: Why do you keep the cows?

P: We make a living by raising the cows. This is the source of income for us. We have to live, don't we? We sell the milk.

I: You mentioned the milk, is there any other thing you might get from the cow?

P: No, we don't get anything else.

I: How important raising cows is for the household?

P: How much would we want for the cows ...

I: How important raising cows is for the household, (as you mentioned that you have seven cows) economically?

P: economically (...) it's not happening. we are suffering loss. How much are we spending?

I: No, not that. How important keeping cows is for your income?

P: 200-300 Rupees per day would be good.

I: What do you feed your animals?

P: We feed straw, and grass from the field!

I: Have you ever given them something to help them grow?

P: No, no, we get nothing! what will you give them?

I: How are they kept? Where are the cows kept?

P: They are kept in the cowshed here!

I: [For the purpose of the interview the cows are kept in an enclosure which is attached to the household. There are three cows and one calf here at the moment, it is clean and that's it.]

I : Who looks after the animals in the house?

P: I do, my sister does!

I: Do you and your sister do different work for the animals?

P: No, it's the same!

I: Does anyone outside the household help you to look after the animals?

P: No, No, we do it ourselves.

I: How did you learn to look after the animals?

P: We learnt it from the rural community. And there's a doctor at "Anchal", he treats them, that's how we learn.

I: Does the doctor advice you on how to look after the animals?

P: Yes, yes, they do. doctor...

P2: There's a doctor at "anchal". When we call them, they come to give us advice, and give treatments from worms and etc.

I: What's the main source of advice? Who would generally advice you on how to look after animals?

P: The doctor. The doctor from "anchal" advices us.

I: We are going to the next section. What do you do when your cows get sick?

P: We call the doctor; he sees the cows.

I: Do you go to the doctor or does the doctor come to the house?

P: When we call the doctor, the doctor comes.

P2: The doctor keeps busy in different places, when we call he gives us a time, for example if we call now, he might say he would come at four. Then he would come at four, assess the situation and give treatment. He would provide whatever is needed and give directions as to how you need to give the medication. If the problem still persists, he will come and give medication for two three days again, and if it's still there, we call yet again. This is how the treatment is done. And there's a camp once in a year, doctors from three four GPs come together for the camp. They ask us to bring the cows, and they see if the cows are sick, and all! The doctor takes care of everything that happens here, we don't do anything on our own.

I: What do you do when the animals get sick?

P: we call the doctor; he pays us a visit and prescribes medication. We buy them and feed it to the animals.

I: Where do you buy the medicines from?

P: There's a shop in *(Local town name redacted)*, we go there to buy medicine.

I: The shop that you mention, do you get medicines for both animals and humans there or just animals?

P: Just animals.

I: Who gives the medicines to the animals?

P: I have a sister; she gives the medicines.

I: When do you stop the treatment?

P: They doctor gives the medicines, when it gets over, we stop.

I: Do you remember which medicines were given?

P: No, No, I don't remember.

I: Do you remember the names of any medicines which were given to the animals?

P: No, we don't remember. Vitamins and such are prescribed, we get it and feed it to the animals.

I: Have you ever gone to the drug store to get medicines without consulting the doctor?

P: Yes, sometimes we get the deworming medicines ourselves.

I: Where do you get these medicines from?

P: From the shop in *(Local town name redacted)*.

I: When do you go the shop (to buy medicines) what do you tell them?

P: I would say that I need deworming medicines for my one-year old calf.

I: Are there any other problems in the cows he would get medicines for (other than deworming medicines)?

P: Yes, sometimes we get the vitamins and such from the shop when the cow is sick!

I: You'll go yourselves to get medicines from the shop?

P: Yes, we go ourselves.

I: You do have any medicine at home? (like old medicines, or the case)

P: Yes, I have them. The case for the vitamins and sorts.

I: Could you bring the medicines for us to see?

(participant brings the medicines described. They are identified as vitamin supplements for large animal use)

I: Do you get different medicines from the pharmacy other than these?

P: yes, we get whatever we are given. sometimes, we are given powder.

I: Do you know what antibiotics are?

P: Antibiotics? No, I don't know.

I: You mentioned you go the medicine shop without consulting the doctor sometimes, why do that?

P: Doctor also prescribes the same medicine. We go and buy it.

I: But why? if you could explain it a bit further?

P: The doctor isn't always available. And people at the pharmacy are also doctors. We tell them I have a cow and it looks sick, give us some medicine. We say it to the doctor, the doctor gives us the medicine.

I: There's a doctor there?

P: Yes.

I: What problems in your cows would you get medicine for?

P: For example, few months ago they had sores on the legs, sores on the skin. It happened severely. We had to get medicines at that time. We had to apply oil, and ointments. There were large sores on the leg. Few months ago, all of my cows were sick. It was on the legs, on the body, they could not walk. It happened severely!

I: Is there any other problem (you'd seek help for)?

P: In case of fever, yes.

I: What treatment do you usually get for fevers?

P: The doctor would inject it. They would know what they are giving.

I: Would you go the pharmacy directly if the cow has fever?

P: No, we see the doctor he would say whether he needs to give injection.

I: What do you have in your hand? (directed to a family member standing with the participant)

P: This is medicine!

I: For yourself?

P: No, for my mother. She has fever.

I: You mentioned you go to the doctor, or call him, even go to the medicine shop at times. what do you do the most?

P: We often to the medicine shop and get the medicine. If it's very serious then we call the doctor.

I: Why do you do that?

P: You mean the doctor?

I: You mentioned you go to the medicine shop the most, why is that?

P: We do it when we see that the cow looks sick, it's not getting better. Then we go to the drug store. There's a doctor there. He gives medicines when we ask him. We get the medicine and feed it to the animals.

I: Why don't you call the doctor?

P: If we call the doctor, we have to pay a fee. There's a lot to consider. That's why we don't call the doctor always.

I: When you go to the medicine shop to get medicines, on whose advice do you do that?

P: We go ourselves, no one advices us, we are the guardians.

I: Has there been any medicine that you used both in the cows and in the family?

P: No. (...) Veterinary medicines are for animals alone, not for humans.

I: Have you ever gone to a human healthcare provider to ask for advice about animal health?

P: We don't go because they say they can't give medicines for animals. When animals get sick.

I: You never go?

P: Sometimes imagine the cow is sick, we see the vet, sometimes a doctor [human] would prescribe human medication. So, we get that. but it didn't work!

I: Has there been any situation where the doctor has prescribed human medication?

P: No, they don't give human medication. An animal doctor would give animal medication. Also, there's a camp every year. They give vaccines, and vitamins. They always give veterinarian medication. No human medicines are given.

I: Which doctors do you go to for your animals?

P: There's a govt doctor at "Anchal". We go there.

I: You mean the one who sits at the GP office?

P: Yes, who sits at the GP office.

I: Anyone else?

P: No, he's the one.

I: Does that doctor come to your house?

P: Yes, when we call him, he comes. But we have to give him a fee when he comes.

I: Which doctor do you go to when someone in the family is sick?

P: We go to human healthcare providers.

I: Where do you go?

P: There are places in Dewantala, Diamond (Harbor)…

I: Are there any other doctor nearby that you'd go to?

P: No. (...) There's [name removed] who has become a Pranibandhu.

I: But isn't [name removed- Pranibandhu] an animal doctor?

P: Yes, we see him too?

I: For the family?

P: No, we go the human health care provider.

I: I was talking asking about human health care providers.

P: There's [name removed] in this village *[referring to the allopathic “quack” doctor (informal provider of human health)]*. We go see him sometimes. If there's something serious we go to the hospital.

I: You mentioned you go to the Govt. GP vet, and you call [name removed- Pranibandhu] home. Whom do you see more?

P: We see the GP vet more. [name removed- Pranibandhu] takes more fee.

I: Why do you go to the GP vet more?

P: We get the medicines for free there. That's why we go there more often. For example, this cow needs to be impregnated now, at the GP we get treatment for 35 Rupees. But if we call [name removed- Pranibandhu], he will take 200-250 rupees.

I: [name removed- Pranibandhu] takes the fee?

P: Everyone takes fee. The GP vet would take 35 Rupees, but [name removed- Pranibandhu] would take 250 Rupees.

I: Are you aware of the level of training of the doctors?

P: We don't know that.

I: What do you think what their level of training is?

P: [name removed- Pranibandhu] took training for Pranibandhu.

I: Have you got medications from the family directly from the drug store?

P: Yes, for example my mother is sick, I went to the drug store to get the medicines.

I: You mean a medicine shop?

P: Yes, we also go to [name removed, referring to the allopathic “quack” doctor (informal provider of human health)], for example my mother has diarrhea, so I got medicines.

I: [name removed, referring to the allopathic “quack” doctor (informal provider of human health)] is the doctor? And what about the drug store?

P: [name removed, referring to the allopathic “quack” doctor (informal provider of human health)] is a quack doctor. We also go to a drug store at Dewantala.

I: Have you given any of those medicines to the animals?

P: No, No, we only give animal medications to animals.

I: Has [name removed- referring to the allopathic “quack” doctor (informal provider of human health)] ever advised you on how to treat animals?

P: No.

END OF INTERVIEW
