# Supplementary material for: “If It Works in People, Why Not Animals?”: A Qualitative Investigation of Antibiotic Use in Smallholder Livestock Settings in Rural West Bengal, India
Source: Antibiotics (Basel). 2021 Nov 23;10(12):1433. doi: 10.3390/antibiotics10121433 (PMC8698124; doi:10.3390/antibiotics10121433)
Supplement: Supplementary file 1 [file antibiotics-10-01433-s001.zip › Supplementary S1_ Interview Transcripts/Site 1/LK6 (site 1).pdf]

Code for Study - ‘If it works in people, why not animals?’: A qualitative investigation of antibiotic use in smallholder livestock settings in rural West Bengal, India: LK6, Site 1
Date: 03/07/2019
Location: Site 1
Interviewee: Livestock Keeper (LK)
Interviewer: Jean-Christophe Arnold (J-CA)
Transcription: Debanjan Debnath (DD)

I: Interviewer (JCA)
P: Participant (LK6)

START OF INTERVIEW

I: Which animals do you have?

P: I have ducks and one cow.

I: We noticed... (erm) you have chicken, those are yours too?

P: Yes, three pieces.

I: How many ducks do you have?

P: 8 pieces.

I: How many cows do you have?

P: One.

I: Who owns the animals?

P: All of us, my son, daughter-in-law we all take care of them.

I: Who is the owner?

P: That's me.

I: Why do you keep animals for the household?

P: I am made redundant, can't do any work (showing a severed toe) Raising ducks, with the eggs and such, help us make some money, with that we buy things from the market. Now if there are a few eggs, since we can't buy fish or such, we can serve it to the relatives in case they pay a visit.

I: What do you get from the ducks and chickens?

P: We get the eggs, and if we need the meat, we butcher them.

I: What do you keep the cow for?

P: The milk! For the kids, for ourselves too!

I: Do you do anything else with the milk apart from having it among yourselves?

P: No, it doesn't get sold. When children are being brought up, we can't afford to buy it from the market, it's 30-40 Rupees a Kilo. We raise the cow, it's for ourselves.

I: How much do you depend on the animals economically?

P: As much we can afford, we try to maintain the food. When we don't have money, we would give lesser amounts.

I: How much do you depend on the animals (the ducks, the chickens and cows) for money?

P: Not much, it's small, I don't need it to be large. In this little space, 5000 Rupees would be enough for food, mending the house (for animals), and buying new animals.

I: In a month?

P: (nods)

I: What do you feed to the animals?

P: Maize, for the chicken.

I: What is it?

P: Corn, it is mixed with other chemicals, made into a pile and sold in the market.

I: What about the ducks?

P: Rice powder, and wheat flour, broken rice which we call "Khud" in the native tongue. We boil it together.

I: And the cow?

P: straw, rice husk, lime (Chuni Bhusi).

I: Do you give anything to help them grow?

P: No! We don't give any sort of injection!

I: What did you mean by Injections?

P: Well, for example, it has diarrhea. It might have cold and cough. Then we tell the doctor that it was coughing all night. The doctor, then, might give a tablet. Or, if it's required, they would push an injection.

I: What do you do when the animals get sick?

P: It's impossible to take the poultry for treatment often. There's a doctor here, when we call him, he comes. He sees the animals and gives the medicines.

I: How about the cow?

P: We need to take the cow to the doctor. Sometimes, if it's too sick, we call the doctor.

I: Who is this doctor you're talking about?

P: There's a government doctor who comes to Anchal *[referring to Site 1 Livestock Development Assistant].*

I: Do you get medicines from anywhere else but the doctor?

P: No! There's no one else to treat them!

I: Apart from the doctor do you go anywhere else for the medicines?

P: No!

I: Who gives you the advice how to take care of the animals?

P: Where we get the babies (of the animals) from, they tell us to give medicines twice or thrice a week. When they grow older, we are used to give more diluted medicines. When they get sick, we are to see the doctor.

I: Which medicines are you told you give them twice or thrice a week?

P: Vaccines, Vitamins.

I: Where do you get the medicines from?

P: There a shop in the market.

I: Where is this shop and what is it called?

P: *(Place name redacted)* and there's one at *(Local town name redacted)*.

I: The shop that you said you go to (at Kalatala), what sort of medicines are sold there?

P: Many types for different animals.

I: Are they for animals?

P: Yes, for animals.

I: Apart from animals are there medicines for any other use?

P: No, just animals. The fish, poultry, birds, cows, dogs all medicines.

I: Where in Kalatala is this shop?

P: When we get down at the market it's on the side. If you ask there anyone can show you!

I: Do you go to the medicine shop for any other reason?

P: I don't have other reasons.

I: You told us about the GP doctor, is there any other way you receive treatment when your animals get sick?

P: We go there when the animals get sick, for no other reason.

I: Do you go to other places (apart from the doctor mentioned before)?

P: No, no.

I: Why don't you go?

P: It's free of cost there! If that doctor doesn't come *[Site 1 Livestock Development Assistant]*, then we have to go to others in case the animals fall really sick.

I: Which other doctor are you talking about?

P: There a doctor at Kalatala *[referring to the Pranibandhu]*

I: Where is his clinic?

P: It's in the market, near the main road.

I: Who does the doctor treat?

P: Just animals.

I: (To confirm) Where you get the medicines from, you mentioned, and this doctor, are they different people?

P: They are different people.

I: When the animals get sick, are they treated the same way (or in different ways)?

P: No, all of them together. If one of them gets sick, we give medicines to all of them lest the illness catches the other ones.

I: Do you know which medicines are given?

P: How would we know which medicines are given!

I: Who gives them the medicines?

P: The wife feeds them the medicines!

I: Where do you get the medicines (which you give them) from?

P: We buy it, or it's given by the GP.

I: When you buy the medicine, how do you know which medicine to buy?

P: We tell the doctor what the problem is. For example, if we have fever, cough, body ache, we go tell the doctor, they give medicines accordingly.

I: Do you say this to the doctor or the drug store?

P: The doctor! if the doctor has the medicines, he gives us, or he prescribes it, we buy it from outside.

I: Have you ever gone there without consulting the doctor?

P: No!

I: Do you know what antibiotics are?

P: I've heard the name, but I don't know what it's used for.

I: Have you ever used antibiotic medicines?

P: (Shakes head)

I: You don't know?

P: No!

I: The medicines that you give (to the animals), do you know their names?

P: No!

I: Why not?

P: It's in English.

I: Do you have any medicine in the house?

P: No!

I: For what problems in the animals would you go to the doctor?

P: What I said. If they have white stool, they are not eating, they are tired, not moving. this is mostly the case.

I: Which animal are you referring to (in whom such problems occur)?

P: Chicken.

I: What about the ducks?

P: They don't get sick!

I: What about the cows?

P: They cough.

I: For the cough ...

P: Cough, then they would stop eating, for these reasons we would go to the doctor.

I: Has there been any medicine that has been used in both human and animals?

P: No!

I: Why not?

P: Human medicines have less power. Animals require more power.

I: What do you do when someone in the family gets sick?

P: We go to the hospital.

I: Which hospital?

P: There's a hospital in *(Local town name redacted)*.

I: Do you go anywhere else?

P: If there's something severe we have to go outside.

I: What do you mean by outside?

P: Diamond Harbor, Kolkata. For instance, I had to be operated in Kolkata.

I: Where you go for human health, have you ever asked for advice for your animals?

P: No! they don't do it there.

I: What do you understand about the difference between human medication and animal medication?

P: There is a definite difference. They have different diseases; we have different diseases.

I: Where are the animals kept?

P: There a room inside the house.

I: For which animal?

P: The chicken and the ducks stay separately in one room, there's a bigger space for the cow.

I: Who looks after them?

P: We all do!

I: Do people in family have different things to do for the animals? (or not?)

P: No!

I: Does anyone from outside the house help to look after them?

P: No!

I: How did you learn how to look after the animals

P: Feeding them properly, giving medicines at the right time...

I: But how did you learn to do that?

P: We observed and learnt from our older generations.

I: We are finished.

END OF INTERVIEW
