# Supplementary material for: “If It Works in People, Why Not Animals?”: A Qualitative Investigation of Antibiotic Use in Smallholder Livestock Settings in Rural West Bengal, India
Source: Antibiotics (Basel). 2021 Nov 23;10(12):1433. doi: 10.3390/antibiotics10121433 (PMC8698124; doi:10.3390/antibiotics10121433)
Supplement: Supplementary file 1 [file antibiotics-10-01433-s001.zip › Supplementary S1_ Interview Transcripts/Site 1/LK7 (site 1).pdf]

Code for Study - 'If it works in people, why not animals?': A qualitative investigation of antibiotic use in smallholder livestock settings in rural West Bengal, India: LK7, Site 1
Date: 03/07/2019
Location: Site 1
Interviewee: Livestock Keeper (LK)
Interviewer: Jean-Christophe Arnold (J-CA)
Transcription: Debanjan Debnath (DD)

I: Interviewer (JCA)
P1 and P2: Participants include two members of the same household (LK7)

START OF INTERVIEW

I: What animal do you have in your house?

P: One goat, around 10 chicken, and three cows.

I: Who owns the animals?

P: I am, who else? I look after them, I am everything.

I: What are reasons why you own the cows?

P: My son urged me to raise chickens. So, I started raising 10 chickens. Two of them died, and now I have eight.

I: You talked about chickens, what about the others?

P: And sometimes at religious ceremonies, if we don't have money, we can sell a goat for 5000 rupees. We can use that money for the family.

I: What about the cows?

P: Well, we can't afford to buy milk for our sons. So, we keep a cow for the milk. Some of it, we drink, the rest we sell off.

I: How important raising livestock is for the family economically?

P: Chickens give eggs for us to eat. And sometimes if there are guests in the house, if we can't afford to buy, we would butcher one and use for cooking.

I: And others?

P: I have just started raising a goat.

I: How about the cows?

P: We get the milk, a calf is born every year, we get the milk. We don't have to buy it from outside, children get to drink it at home.

I: What do you feed the animals?

P: The cows?

I: All of them, if you could talk about it individually.

P: The chickens I give rice, puffed rice whatever we have spare. The cows, we give straw and grass. The goats, we give grass and the rice water. Bur flower tree is supposed to be protein food for the goat. When we went to the meeting (the camp) they said rice water and some herbs for protein.

I: Where was this meeting?

P: Camps for animals are held from time to time. Doctors come there. How often the goats need to be impregnated, how often they give birth. In every six months the goats give birth. Every six months they need to be impregnated.

I: Are there other ways that you have learnt how to look after the animals?

P: Give them shower once a week.

I: Where did you learn that from? Has there been any other sources that you learnt it from?

P: No, no we haven't learnt from other sources. The way people generally do it, we have heard from them. And once we went to the meeting.

I: How often do you go to these meetings?

P: Not often, then we deposited money, opened an account so they called a meeting, we had a discussion there. But there hasn't been anything like we are doing this so we have to go the meeting. Or we know the work and we have to go the meetings, it's not like that.

I: How often does these meetings take place? Could you explain it a bit further? When was the last time there was a meeting?

P: A long time. Two/three years ago!

I: Nothing since then?

P: No! We didn't deposit money, and no meeting happened!

I: what money?

P: We opened an account with 100 rupees. It could be 50 Rupees or 100 Rupees from a company. We also lost the money.

P2: No, but I got it back! Once I did 20 Rupees, and then 50 Rupees, I got them both back. I didn't do it again. Who did after me, they didn't get their money back. The company got shut down.

I: Which company?

P: They ran this initiative with the name, "[name of initiative]". I did it too. So, they called for a meeting, and we had a discussion there.

I: What did you get the money for?

P: So, we opened an account. We were supposed to get twice as much interest in four year.

I: Who in the household looks after the animals?

P: I do.

I: Anyone else?

P: My husband does.

I: Do you have different jobs for the animals?

P: No (...) (indistinct)

I: What do you do when the cows get sick?

P: We call the GP doctor [the public VPP (livestock development assistant]. When we can take the animals to the office, we do that. Otherwise we have to call the doctor home in exchange for a fee.

I: What do you do for the goats?

P: It's the same thing. Either you call the GP vet, or some other doctor. It's the same.

I: Do you call other doctors for the cows?

P: It's the same. For the cows, the goats, the chickens it's the same doctor. We go to the GP and see if the doctor can save the animals!

I: Why? Why don't you go to other doctors?

P: We don't get other doctors here. The GP vet is cheap. When we call other doctors, we'll have to pay more.

I: You said you go to the GP for the goats, and other times you call the doctor home. You don't do the same thing for the cows?

P: Yes, it's the same for the cows as well! We go to the GP vet, we seek other doctors in case he's not available!

I: When would you call other doctors?

P: When this doctor isn't available on phone!

I: Who's the other doctor? (other than the GP vet)

P: He's also some GP vet, when our GP vet isn't available, we would call him.

I: What's his name?

P: *[name redacted- public-private VPP1 (pranibandhu), site 1]*

I: What about the chicken?

P: It's the same one. When the chickens have flu, they come to give injections. They charge one Rupee per Chicken.

I: When you call the doctor for treatment, where do you get the medicines from?

P: They, themselves give the medicines. If the GP vet is giving the medicines, it's a bit cheaper. If we're calling doctors from outside it's a bit more expensive. Both the cost of the medicine and the doctor is bit more.

I: Who gives the medicines to the animals?

P: We do it, the doctor gives instructions for when to give it.

I: Where do you get the medicine from?

P: The doctor keeps medicines. If you tell them what the problem is, they carry it with them during the visit.

I: As you mentioned you sometimes use human drugs in animals in case of Diarrhea, why do you do it?

P: sometimes if we can't buy medicines, and the goat has diarrhea, we would use the diarrhea medicine from the house for them get well soon. It would probably work.

I: Do you understand a difference human drugs and animal drugs?

P: No, we don't understand. Cows generally require higher power of medicine.

I: Has there been any other situation where you have used human medication in animals?

P: No, not we don't use other medicines!

I: Have you ever used animal medications in the family?

P: No!

I: Why not?

P: Animal medicines has a higher power and the composition is different. That's why there's a difference, we can't use them in humans.

I: What about the medicines for the chickens and goats?

P: They have different medicines.

I: Why can't they be used in humans?

P: No, it won't work at all!!

I: but why? (laughs)

P: That we don't know!

I: If you don't use animal medications in humans, why would you use human medications in animals?

P: We just think that if it works for humans, it might work in the cows for the same problem. That's what we think.

I: Where do you go when people in the family get sick?

P: Diamond Harbor hospital is there. Then there are other doctors in [village in site 1 name redacted] and neighboring areas. But they aren't qualified. To see a good doctor, we have to go to the hospital in Diamond Harbor.

I: The doctors you mentioned, do they give advice regarding the health of animals?

P: No! Animal doctors, as I said are different.

I: They (the human healthcare providers) never advise you on animal health?

P: No, they won't treat animals. If we ask for advice, they would perhaps give us an idea what we could give them.

I: Can you give us an example of what sort of suggesting the human doctor might give you regarding animal health?

P: For example, if the cow would have sore in its cheeks, the doctor would ask us to heat some water and rinse the mouth and the tongue. A very general idea.

I: Have the human health providers ever given medicine for the animals?

P: No, only in case of Diarrhea he would probably suggest that we use two tablets, instead of one in animals, and see if it works.

I: Do you know what medicines they are?

P: No, can't tell you names, never read them.

I: Do they prescribe the medicines?

P: No, only the animal doctor would prescribe.

I: When the human health care provider tells you which medicines to use (for diarrhea and such), where do you get the medicines from?

P: The doctor himself gives it. Only in case that he feels it's important.

I: Which doctor are you talking about?

P: Just in general.

I: Do you have a name?

P: [name redacted- name of a human healthcare professional]

I: Where is his clinic?

P: [name of village in site 1 redacted]

I: Do you have a number?

P: No!

I: Where in [name of village in site 1 redacted] it is?

P: (Mumbles) (indistinct)

I: As you mentioned you would sometimes go to a human healthcare provider for advice on animal health, in which situation would that happen?

P: The other doctors weren't available. The doctor at the GP isn't always available, he would only come on specific days.

I: How often does it happen that GP vet isn't available?

P: It happens a lot. For example, if something happens right now, we won't the GP vet, he's on leave.

I: For which animal would you usually seek help from human healthcare provider?

P: Mostly for the goats. it's when they have diarrhea.

I: If the GP vet isn't available, what do you do?

P: We call some other doctor. He comes if he has time, otherwise we have to wait!

I: In which situation would you go to the Human Healthcare provider?

P: The GP vet's clinic is in [name of village in site 1 redacted], only in case we don't get him, then. Mostly we don't have to! Maybe if it's Sunday, the holiday.

I: How often does it happen?

P: Not very often, if the situation is really bad, and we are not getting a doctor. If it's on Sunday, then.

I: You don't get any doctor on Sundays?

P: All of the doctors are on leave, mostly they don't want to come!

I: The interview is finished.

END OF INTERVIEW
