# Supplementary material for: “If It Works in People, Why Not Animals?”: A Qualitative Investigation of Antibiotic Use in Smallholder Livestock Settings in Rural West Bengal, India
Source: Antibiotics (Basel). 2021 Nov 23;10(12):1433. doi: 10.3390/antibiotics10121433 (PMC8698124; doi:10.3390/antibiotics10121433)
Supplement: Supplementary file 1 [file antibiotics-10-01433-s001.zip › Supplementary S1_ Interview Transcripts/Site 1/LK8 (site 1).pdf]

Code for Study - 'If it works in people, why not animals?': A qualitative investigation of antibiotic use in smallholder livestock settings in rural West Bengal, India: LK8, Site 1

Date: 18/07/2019

Location: Site 1

Interviewee: Livestock Keeper (LK)

Interviewer: Dominic Day (DD)

Translation: Somraj Das (SD)

Transcription: Sayak Manna (SM)

Interviewer (DD)

Translator (SD)

Participant (LK8)

START OF INTERVIEW

Interviewer: okay, could you describe what animals you keep?

Translator: what kind of animals do you keep exactly?

Interviewee: Goats.

Translator: He only keeps those goats as his livestock.

Interviewer: how many of them are there?

Translator: How many?

Interviewee: about six.

Translator: there are plenty of them, maximum six.

Interviewer: Six, okay. And who owns these animals?

Translator: Who is the owner of these goats?

Interviewee: Mother.

Translator: is your mother the owner?

Interviewee: She is the one who looks after them.

Translator: we are not talking about looking after them. Who is the owner?

Interviewee: ??? (Maybe nods, nothing audible)

Translator: what he's saying is that the owner of these livestock is his mother.

Interviewer: okay. Who looks after them?

Translator: who looks after them?

Interviewee: Mother.

Translator: Mom looks after these livestock.

Interviewer: Okay. Great. For what reasons do you keep them?

Translator: why do you keep these goats?

Interviewee: we like them, that's why, for that reason only people keep animals. The time passes smoothly; they are taken to the field, they are fed and taken care of.

Translator: well, let me speak, one second. Well, he's saying that they keep them as a livestock because they like to keep them.

Interviewer: because they like to keep them?

Translator: Just like a dog.

Interviewer: okay.

Translator: they like to play with them. Run them in the ground with. They want to walk them around.

Interviewer: So, how important are they economically for the household?

Translator: you keep these goats; do they bring you any monetary benefits?

Interviewee: yes, at times it does. When they grow too old, or they increase in numbers, then we have to sell them. We can't maintain a large flock. Suppose in place of these six goats, the number increases to eight or ten, the rainy season is coming soon, now we have to cut the grasses from the field and feed them. Who would do all these works? Then two or four are sold off, we'll have six or four to ourselves. All in all we have four to six goats with us all the time, sometimes it becomes eight, and then we have to sell a few of them off.

Translator: well, he's saying that it depends. Sometimes it depends that if they get old they sell them in the market for money. Or sometimes the population becomes overpopulated; the environment for goat keeping sometimes gets overpopulated…

Interviewer: Yeah, too many goats.

Translator: Supposedly they are six, if they become eight or nine, they usually sell three of them to make it balanced. That's the way. It depends.

Interviewer: Yeah, Um, so what's the main economic activity in this household?

Translator: how do you make a living? What work do you do?

Interviewee: I work as a mason (*Life history redacted)*

Translator: well, his basic profession is that he is a mason *(Life history redacted)*

Interviewer: (laughs) so, do you keep these animals as a form of food ???too?

Translator: how do you take care of the food habit of the animals?

Interviewer: we primarily feed grass, wheat and flour. These are fed to them.

Translator: well, what he's saying is the food ?? means he sometimes feed them wheat, dust wheat, green grass etc. There is nothing specific about the food security or something.

Interviewer: so, that's just what they feed the goats.

Translator: Regular grass, green grass.

Interviewer: okay. Um, so do you give anything for the growth promotion?

Translator: what?

Interviewer: Do you give anything for the growth promotion?

Translator: do you promote such things? Like you feed them food, then do you sell them? Do you take them to the market and sell them?

Interviewee: No, there is no such system.

Translator: No.

Interviewer: okay. And how, where do you keep the livestock generally?

Translator: where do you keep the animals?

Interviewee: we keep them here only, in the veranda. We clean up a bit here, well, since its rainy season, it's a bit dirty now, but we clean this are and keep them here.

Translator: Well, what they are saying is currently they keep their livestock right here but afterwards when the monsoon will be gone, they keep them on the other side by cleaning and making the place suitable for the livestock.

Interviewer: okay, great. Does anybody else help them to look after the goats?

Translator: who else looks after them beside you?

Interviewee: we ourselves from our family take care of them, my mother and my brothers. The four, five of us take care of them.

Translator: well, it's family members. Mothers, sons who are responsible to keep and maintain them, there are no other to help them.

Interviewer: okay. How do they learn how to look after the animals?

Translator: how do you take care of the animals?

Interviewer: we make them bathe once a week or once a month. We clean them up. We take them outside and feed them.

Translator: Well, they're saying that once in a month or twice in a month they wash them. They take them for walking outside, they feed them. That's how they maintain them.

Interviewer: okay, but who taught them how to maintain them?

Translator: how did you learn to maintain these goats? Someone must have taught you.

Interviewer: yes, of course. We've been keeping goats for about 15 to 20 years now. Father used to work somewhere, he learnt it from there. Then mother kept them ever since. Now mother is older now, so I and my brother help her in maintaining the goats sometimes.

Translator: well, it's more than twenty years they've been keeping the livestock. Their father knew how to keep them, and they taught their sons. Now they maintain according to the advice of their father.

Interviewer: okay. Brilliant. Can you explain that now we're going to refer to the questions of medicines?

Translator: Now we are going to question about the kinds of medicines that are being given now.

Interviewee: Medicines are given by doctors, we don't know much about that. (someone else: we never given medicines to our goats). We haven't asked about the medicines. If ever we are told to give medicines we go and get them, and feed them accordingly. We don't know the names or the qualities of them. We never asked about it.

Translator: what he is saying is, they do not know the technical matter of the medicine, if livestock gets sick they take them to the doctor, doctor takes steps as necessary. But they do not have any idea about the medicines or antibiotics and all.

Interviewer: uh, who is this doctor?

Translator: Do you know the name of the doctor?

Interviewee: No, I don't know the name of the doctor.

Translator: they do not know the name of the doctor. We are gonna do that over there.

Interviewer: pardon?

Translator: we're gonna do that over there.

Interviewer: okay. How do, uh… okay… so, do you ever take advice about how to look after your animals?

Translator: do you ever take advice on how to keep your animals?

Interviewee: we didn't have to because mother has been looking after them for about twenty years and so. (Someone else: more than that) She has that experience now. She knows what to do, how to keep a goat healthy that is why we don't need to call anybody else.

Translator: he's saying that it's been more than 20 years so they have the expertise to keep the livestock healthy. And that is why they do not require any kind of additional information from outsiders.

Interviewer: okay. Could they describe the situation when last time something got, uh, one of their goats got ill?

Translator: when was the last time any of your goats got sick? Months or years back?

Interviewee: the last they got sick maybe, six or seven months back. The kid died actually. Maybe from whatever it ate, or it might have had a gas problem, it had runny diarrhoea. We gave it medicines, but it didn't work. And it finally died. It must have been more than six months, maybe about a year. Otherwise the kind of treatment my mother does, there's no reason for any of the goats to get sick. My mother can take care of them very well. She takes care of their food habits more than she takes care of us. She is worried about how to keep them, where to keep them. She is obsessed with them. This is the situations. She goes to the field in the morning, brings freshly cut grass for them, then she gives them the starch of boiled rice, she tears the packs of flour and gives those to the goats. She is very much into them. This is how mother takes care of them.

Translator: what he is saying that it's been six months or more than that. There was an instance; there was a cub that got dysentery and many kinds of problems. They were feed some kind of medicines, they don't have any idea about. But the cub did not make it.

Interviewer: pardon?

Translator: the cub did not make it.

Interviewer: oh, okay.

Translator: Alright? And they are saying that they have too much of expertise. These kind of incidents do not take place. It has been six months, or a year, after that there Is not incident like that.

Interviewer: okay.

Translator: yeah.

Interviewer: Could they explain why did they go to the provider that they did? Why they chose that person.

Translator: why do you go to the doctor? You go to the same doctor, isn't it?

Interviewee: yes, if we need to go, we are supposed to go to that one doctor only because we don't have any doctors besides him here.

Translator: and what about the medicines?

Interviewee: we have only him here, so he only looks over the whole situation. All the cows and cattle we have here are looked after by him. So we go to him only. And there is another doctor in Ashapur, a different village, who may come but we have to pay a fee to the other doctor, but the doctor in our locality provides service free of cost. So we go to him.

Translator: well, what he's saying is that there is only one doctor around here. So don't have any choice. There is one place which is not here, that is Ashapur, if you go there sometimes they charges you in currency. That's why they go here, it's also near.

Interviewer: and does this person, is this person qualified?

Translator: All in all, what do you think, does the doctor hold degrees?

Interviewee: Yes, since he's a government paid employee, he must be qualified. Those who don't hold degrees won't be government employee. Isn't it? He is a government employee to treat the animals of the locality. There are lots of cows and goats in our village, but we don't have any vet doctor except for him. That is why he's been employed by the government.

Translator: well, he said that the government sent him. So, he hopes that the doctor is qualified. That's why they go to the person.

Interviewer: okay. Can he describe how he administers the medicine?

Translator: from where and how does he give the medicines?

Interviewee: we get the medicine from him only. He has medicines with him. From him we bring the medicine. If there is a doctor there must be medicine.

Translator: and how do you feed the goats?

Interviewee: As the doctor instructs, in the morning we have to feed them, we break the tablets and mix it in water, then we open up his mouth and pour the water it.

Translator: Same thing, he gets medicine from the doctor, he comes with the advice and the medicine. They dilute it with the water and they feed them.

Interviewer: is it a tablet or liquid?

Translator: is it tablet or watery substance?

Interviewee: Tablet.

Interviewer: okay. Do you have any left in the house that I can look at?

Interviewer: Do you have any bottle of medicine, or any tablet strips? Anything will do. Do you have anything? Or have you thrown it away?

Interviewee: No, we don't have any of those left.

Interviewer: Do you find it easy to get hold of the medicines?

Translator: Do you face any kind of problem to get the medicines? to bring it, or to store it, or to buy? Anything?

Interviewee: No. we don't face such problems.

Translator: No, he doesn't face any kind of problems.

Interviewer: Do you ever find the medicines you're given don't work?

Translator: come again.

Interviewer: Have they ever noticed medicines they're given don't work?

Translator: have you ever felt that the medicines you received don't work?

Interviewee: No, I have never thought so.

Translator: They yet haven't had that kind of experiences.

Interviewer: okay. Can you ask if they, well, it is not in a logical order, but can you ask whether or not, um, is there any other reasons that they chose the provider that they did?

Translator: why do you go the same doctor over and over again? Don't you have any other options?

Interviewee: in our locality we have that one doctor only. That is why we go to him. In about six months or so we go there, not all the time. Whomever we find nearby, we go to him.

Translator: it's about availability.

Interviewer: So, would you ever use human medicine in your livestock?

Translator: did you ever use human medicine to goats?

Interviewee: Um, no. it never happened so. But at times their skins get torn; maybe they fell down from somewhere, so they got injured. Then we use human medicines, the ointments are applied. This happened a few times.

Translator: He is saying that it doesn't work like that. He never uses human medicines. But sometimes, in some kind of situation when the livestock get wounded, or get scar on their skins, then they apply some kind of lotion or something that the human uses.

Interviewer: And who provides these medicines?

Translator: when you apply those human medicines, or ointments, where do you take that from?

Interviewee: we have many practitioners, and hospitals here. These doctors have their chamber here. We get the medicine from them.

Translator: Are they from this area?

Interviewer: yes, in this area only. Where you people got down from your car, right next to it is a chamber of a doctor. If a doctor writes a prescription, then we can take it to some other shop and get the medicine. They will take the fee.

Translator: what he is saying is that, there are many doctors for human who practices. Sometime you gonna visit them, you gonna tell them about your problems, and they're gonna prescribe you, they're gonna provide medicine from the counter and they're gonna charge you for that.

Interviewer: And do they know that these medicines are being used on animals?

Translator: do you think the medicines that are used on people are also being used on the animals?

Interviewee: No, I don't think so. The medicine for animals is different and the medicines for the human being that is different. The diseases of human beings and of animals can't be the same. That's why the medicines are different too. The doctors, who treats and gives medicines for the goats and cows, can't usually provide medicines for the human.

Translator: he thinks they don't. He has his own philosophy, that is, human medicines do their duty in much more effective way and cannot be effectively used in livestock.

Interviewer: they can or can't?

Translator: can't. If you are having a problem and you are being prescribed with a medicine, that medicine according to his philosophy won't work into livestock. And there's a vice versa.

Interviewer: okay.

Translator: the livestock medicine cannot be effective on the human's body.

Interviewer: okay. So what does he think is the difference between human and animal medicines are?

Translator: what do you think is the different between the medicines that we take from the medicines that are given to the cows and goats?

Interviewee: there must be some difference. Otherwise why would the doctors be different? The doctors of cows, goats, birds and dogs do not treat the humans. People otherwise could have taken their livestock to their own doctor. That is why I think so. Even if people do so, I think they do wrong by taking their livestock to regular doctor instead of a veterinary physician.

Translator: he made a brilliant answer, he said that, if there are no differences then why there are separate doctors for human and separate doctors for animals.

Interviewer: okay, yeah, that is a good point. Has he ever used animal antibiotics on humans?

Translator: if you ever get sick, I suppose you don't take animal's antibiotics or their medicines?

Interviewee: No.

Translator: No, they don't.

Interviewee: their medicine is completely different. When we bring medicine for the animals, we are given two day's medicine but when we bring medicines for the humans, at least three day's medicine is given. We keep their medicine separately, if they are cured and there are still some medicine left, we throw those medicines away. We don't keep it.

Translator: well, what he's saying is that there is a difference between human antibiotics and animal antibiotics. If you're prescribing antibiotics for humanstock that is going to last for more than two days, but in the case of human problems it'll last for more than two days.

Interviewer: okay. Does he ever take advice from a human doctor for his animals?

Translator: have you ever taken advice from a doctor who treats human, for the problems regarding your animals?

Interviewee: no we haven't taken such advices. Yes, maybe at times when a goat is about to give birth, and she is suffering from the pain, then mother might call someone up for help. She might call someone who is keeping goats for long and has enough experiences. She wants to make sure the health of the animal is taken care of properly.

Translator: have you taken advice from the doctor who treats humans?

Interviewee: no.

Translator: what he's saying that he did not ever take advices from any human doctor practitioner. But when any of the livestock has any massive labour pain, they try to find guys who also have the same livestock for help.

Interviewer: so, he takes advices from people who also keep goats?

Translator: as well.

Interviewer: as well.

Translator: particularly they never took advices from a human practitioner.

Interviewer: okay and would he ever take advices for human health from the livestock medicine provider?

Translator: have you ever done the opposite of it? Have you ever taken advices for human health from the vet doctor?

Interviewee: No. it never happened so. And it is also not supposed to happen this way. This is well out of norm. (system).

Translator: it has not happened. They did not do it. They haven't done it yet.

Interviewer: okay, correct. I guess that's pretty much all my questions. So, would you mind asking him if he has the name or number of the human provider that ??

Translator: now tell us please the name and number of the people who keep cattle such as cows and buffaloes in large numbers.

Interviewee: We don't have such places here. We have a few cow shades, I can show you that. There are poultry birds, cocks as well.

Translator: do you have peacocks too?

Interviewee: No, not peacocks, but birds that are usually kept as livestock, small birds.

Translator: okay understood.

Translator: what he is saying is that, there is a livestock of cows which is limited, one or two consist of. And there are birds; we have nothing to do with birds. So, do you want to see that limited livestock it consists of maximum two, three cows?

Interviewer: Do I? yeah. Yeah. Of course.

Translator: Alright, you show us around a bit.

Interviewer: what I asked was the name and the number of the service provider.

Translator: he is going to take us there.

Interviewer: to the human provider? Okay.

Translator: just do one thing; make an appointment with that retired guy.

Interviewer: yeah.

Translator: are you going to interview him. Because 2.10, if we are leaving right now, then we'll be missing their (husks???) interview. We need to get back.

Interviewer: yeah, it's fine.

Translator: what was the name of the doctor you mentioned? Do you know the name of the government doctor?   (different voices, incoherent speeches)

They are talking about the government doctor. Do you want to interview the government doctor?

Interviewer: No, I was talking about the one that they use, the uh uh…

Translator: that is the government doctor they are talking about, so they barely know the name of the government doctor, because in Indian system the doctors don't say their names.

Interviewer: so they don't know.

Interviewee: whenever we have problems we take them to Panchayet hospitals. It is nearby. It is in jamuntala, 10 minutes from here.

Translator: we can't go now. We don't have the time.
