# Supplementary material for: “If It Works in People, Why Not Animals?”: A Qualitative Investigation of Antibiotic Use in Smallholder Livestock Settings in Rural West Bengal, India
Source: Antibiotics (Basel). 2021 Nov 23;10(12):1433. doi: 10.3390/antibiotics10121433 (PMC8698124; doi:10.3390/antibiotics10121433)
Supplement: Supplementary file 1 [file antibiotics-10-01433-s001.zip › Supplementary S1_ Interview Transcripts/Site 1/LK9 (site 1).pdf]

Code for Study - 'If it works in people, why not animals?': A qualitative investigation of antibiotic use in smallholder livestock settings in rural West Bengal, India: LK9, Site 1

Date: 18/07/2019

Location: Site 1

Interviewee: Livestock Keeper (LK)

Interviewer: Dominic Day (DD)

Translation: Somraj Das (SD)

Transcription: Sayak Manna (SM)

Dom: Interviewer (DD)
Benjamin: Translator (SD)
LSK: Interviewee (LK9)

START OF INTERVIEW

Dom: Thank you very much for talking to us.
Benjamin: Many thanks to you for talking to us.
D: So first thing I wanna talk about is..so the livestock that you keep, if that's okay?
B: First of all he wants to know from you about the livestocks. For how long you are rearing these livestocks? How are you keeping them?
LS Keeper: I am keeping them for 7 months. I've actually seen it on internet and bring them from Barasat. Okay? I brought them around 50 pieces for myself.
B: When did you bring them exactly?
LSK: It's 7 months now. Now if you calculate you can get it.
Outsider: Since January.
LSK: I have brought them since January.
D: What..
B: Exactly what he just says that it's more than 7 months, he bought that livestock by searching in the internet and there is a place called Barasat.
LSK: Yes, Barasat.
B: Where he bought this exactly chickens and the total livestocks.
D: Okay
B: From
D: Okay
D: So, just you know if you can never ask any questions without me asking
B: No. Yes just asking (Not clear)
D: So, could you..could you describe exactly which animals you do keep there?
B: You please exactly say.. I mean..What type of livestocks you rear?
LSK: I do almost everything, I do (does he mean "breed" or "keep" here) the local (he meant

indigenous)-chickens too. I searched them on net and bought them. I got 50 pieces, I have also other...(didn't understand) but due to lack of money I can't. Because in this small space it can't be done, if I have to do shade/coop I need bigger space. Else you can't do it.
B: So what's he saying the he generally likes to keep many kind of or every kind of livestocks but just because as I say, I told you that he is facing some financial crisis.
D: Yeah
B: He already (got/bought?) more than 50 livestocks, he wants to increase the numbers but right now he's facing some financial crisis that's why he's not able to make it right now.
D: Oh-kay. Erm.. could ask him, which species of animals he keeps?
B: What are the types of species do you maintain in your livestocks? Animals?
LSK: Local (here he meant desi/local chickens) and turkeys. Indigenous and Indian turkey.
B: He generally likes to keep Indian turkeys and indigenous chickens (or/called? origin??) cattles that we saw there, (?) and chickens.
D: Okay, I mean that's (..?..)
D: Erm..could he tell me how many of the chickens and the turkeys he keeps?
B: In numbers?
D: In numbers, yeah!
B: How many..in numbers how many animals to you keep, approximately?
LSK: 50 pieces of turkeys were bought. And indigenous was around 20 pieces.
B: So he is saying that Turkeys..Indian turkeys are 50.
D: 50?
B: Yeah and these..how many indigenous chickens you said?
LSK: 20 pieces.
B: And indigenous cocks and chicken that you saw there is around 20!
D: 20? Okay! Great! And is there reasons why he keeps more turkeys than the chickens?
B: Do you have any reasons for why you have 50 pieces of turkeys and 30/20 pieces of chickens? Why more turkeys?
LSK: Turkey looks a little better and I heard there's more profit in turkeys. So I got more turkeys.
B: First of all he finds Indian turkeys more beautiful than the indigenous ones and second one...the second is economical viability of turkeys are more than the indigenous species.
D: And why is that?
B: Why do you think that these turkeys will be more profitable?
LsK: Turkeys will always be more profitable because turkey is 350-400 rupees per kilo.
B: Kg?
LSK: Yes, and the indigenous chickens are 120 rupees kilo!
B: Well he's talking about the meat, that turkey costs more than...300 to 350 per kg. Where the indian chickens .. How much did you say for the indigenous chickens?
LSK: 120.
B: Where the Indian chickens are around 120 per kilogram, that's why the viability of turkeys are much more than Indian.
D: OK.
LSK: The indigenous will grow 2-2.5 kilos and the turkeys will be 15-20kgs.
B: I see. What's he saying right now that, that turkeys are much more viable in quantity, it's around..1 turkey gives you over 15-20 kgs, whereas Indian or indigenous origins of cocks just

like you saw there.. How much you said about the indigenous?
LSK: 20
B: it's going to give you maximum 20 kgs. So the difference is big. (P.S. it will be 2-2.5 kgs and NOT 20 kgs, the interpreter needs to concentrate more).
D: Ohkay..ok..Umm, could he describe for what reasons he keeps these animals?
B: What are your exact reasons to keep them?
LSK: Actually I keep them for my business.
B: I see. And then?
LSK: But now the market for these is not stable.
B: So the market isn't the same?
LSK: No, the market isn't the same. Now we butcher and sell some here. Also I feed some of the shops here.
B: The meat?
LSK: Yes. It tastes very good, even better than mutton.
B: The turkey?
LSK: Yes, the turkey.
B: What's he saying that, he started keeping livestocks first of for the business concept, for selling the meats. and afterwards..right now the market is in the turmoil. The market is not right, it's going slow, that's why they willing to..they usually transfer them into meats and supply them in...you have seen the resturants? As a customer they try to feed them from this.
D: Okay and any other products that they use?
B: Apart from turkey and chickens anything else?
LSK: Yes, we have asked for Campbell ducks but we didn't get them yet.
B: Campbell ducks?
LSK: Yes, they are 3 kilo or 3.5 kilos red Khaki ducks.
B: Are these indigenous?
LSK: No not local but from outside.
B: Campbell ducks?
LSK: Yes Campbell.
B: Well he..where are those?
LSK: Well they are in *(town name redacted)*and *(town name redacted)*.
B: No where are they here? Do you keep?
LSK: No, I don't have them yet. Since the coop is not made yet so we didn't get them!
B: Currently he is not having any kind of other products. He is talking about different kinds of ducks, indian ducks. But he is not maintaing them as a livestock.
D: Ok, so umm, does he get any other products? Such as eggs… no don't say eggs. Does he have any other products other than meat?
B: Other than meat, what else do you sell, if you can say from the business perspective?
LSK: Eggs.
B: Eggs and meat?
LSK: Yes
B: And?
LSK: And nothing much but we do get the chicks.
B: Well he is (saying the eggs), (..?) In comparing..?.. He generally uses or sells meat in the

(session of eggs?). He sells eggs too.
D: (?) Okay and does he sell these for commercial purposes or personal purposes?
B: Do you keep them for business as well as for your personal use too?
LSK: Actually we keep them for business only but as we couldn't get hold of the market right so we butcher and sell them here.
B: Well he said, he started this concept of livestock in the terms of business but the market is not right (that properly go) that's why he is using that in the domestic way too? (How it is a domestic way?)
D: Ok.
B: When the market will be good (...?)
D: Okay! And is it recently? Is it recent that the market that he hasn't been selling it commercially? Started recently?
B: Since when...when? how?... by recently I mean when did you last sell for the business?
LSK: About 2 months.
B: It's recently more than two months.
D: 2 months? Okay! Erm..can he explain how important economically these livestocks are for him?
B: For his family?
D: For..yeah his household.
B: How important are these livestocks important for your family? (He changed the question completely!!)
LSK: of course it is important!
B: How important you think are these for your business or for personal use?
LSK: It is very important. I am not talking about my age (he meant he isn't old yet) but because of lack of space I can't do much. I mean, my this room is very small, in this space you can't do much. Also food for it (what is he talking about?) is a problem, greens are its food. We are not getting cabbages..umm..leafy vegetables are something that we aren't getting now. We don't even have algea, it eats algea, we are not getting that even. Now we feed rice husks/chaffs and (..?).
B: Okay, let me convey this to him. Well, keeping the livestocks is very important that's why, he wants to make those cages much more bigger as I just told you, he's facing some financial problems that's why he's not able to make it large right now. And the second thing he's saying about is diet. These chickens need green diet, especially they consume cauliflowers and cabbages etc. Right now this is not the monsoon (He meant season) of that, that's why they have to feed them the straws and paddy straws (chaff and straws are not same) instead of green vegetables.
D: Ok. I wanted to talk him about..umm.. what he feeds the animals, what he feeds livestocks. So can he describes exactly what he feeds?
B: Can you elaborately talk about what you feed them as their diet?
LSK: Rice and cabbage leaves, leaves of cabbage and cauliflower. Kalmi(a type of leafy veggie) leaves, rice chaffs, also mach.
B: What does mach mean? Corn?
LSK: No mach is chicken food. Grains.
B: Okay. That's all?

B: Well as I said that he feeds them cauliflowers, cabbages, paddy straws, some kind of paddy straws. And there is a concept called corn, there is a tiny little things called mast.
D: Maize?
B: No mast, it's not maize. That's why I just asked him. He said it's a proper food for the livestocks. It's kind of tiny, little dusty! Locals call it mast. So these are the major 4/5 things that they feed to the livestocks.
D: And is it same for turkeys and the chickens?
B: Do you feed both the turkeys and the chickens the same type of food?
L: Yes.
B: Yes it's all same for both the species.
D: And this mast? Does he buy that?
B Do you make mast at home or buy?
L: No I buy them from the market
B: He generally buys it from the local market.
D: So, why does he use this?
B: Why do you use these to feed them?
L: These have proteins in it! Mach has protein in it. Also leafy veggies have them too! So they grow very fast on them. Also during summers we put ORS in their water.
B: ORS water?
L: Yes
B: Anything else?
L: These are what we give.
B: Well he feeds them because it's too much nutritious, it's a high protein diet. Sometimes, especially in the summer time, they used to give the ORS. ORS along with the mixture of regular water. So that they can grow faster and stay healthy and hydrated. That's why they gave them that.
D: And do they give anything for growth promotion?
B: Apart from these do you give anything for the body mass to increase?
L: Yes, we give vitamins.
B: He feeds many kinds of vitamins and suppliments for much more growth (the keeper didn't talk about suppliments though)
D: Is this part of the mast?
B: Is it a part of the diet? (He changed the question completely)
L: We put it in water!
B: No, you feed them so many things, so do you mix them with the food?
L: No, we put them in water, I mean there are 20 of them, we put 20ml in 5L of water.
B: So what's he's saying is..
L: Do we have the medicine now? The bottle? (Must be asking to someone)
B: He wants to show us.
D: Can you ask him to show us after the interview?
B: Please keep the bottle, first finish the interview!
B: So basically he is saying that he uses that vitamin along with the mixture of the water. And it's a part of mast! (NO, it DEFINITELY ISN'T. that Mast/mach is broken grains). It's a part of mast, it's a part of entire diet. Along with that ORS concept!

D: Ok.
B: He wants us to see that bottle but we will see after the interview.
D: Okay, thank you. Where does he get these products from?
B: where do you buy these products from?
L: At Bantala we have a animal product market. There is a Veterinary.
B: What's the name?
L: His name is *Name redacted (Veterinary para-professional)*.
B: *Name redacted (Veterinary para-professional)*? Well he buys these kind of products from adoctor in the local market, his surname is *Name redacted* (*Veterinary para-professional)*. But he can't remeber the full name.
D: Ok. Can you ask him, if after the interview he could tell us where to find this man?
B: Can you exactly tell us the address? We will contact him.
L: What day is today? He's not available today. he sits only once a week.
B: he is not available today, you can meet him once in a week! When can we reach out to him?
L: Tuesday and Saturday.
B: Tuesday and Saturday.
D: Tuesdays and Saturdays? Okay thank you very much.
B: Should I get his number?
D: yeah, we will at the end of the interview?
B: after interview kindly give us the phone number.
D: So, how are the livestock housed? Where do you house the livestocks!
B: Where does he hold the livestocks?
D: Where does he House the livestocks!
B: How exactly you keep them? These livestocks? during day or night?
L: During day they are set free, by night they are sent back to that room.
B: All of them?
L; Yes, everyone! there are first and second floors
B: and?
L: And the indigenous just roam around here and there!
B: As you just said, (..?) housing the species in a different way. Turkeys another part, indian chickens another part. (The Keeper said, all birds are kept together). In the day light they are all free to roam around and after sunset they are all separated in their own sections of the cages.
D: So at night they are kept sepately?
B: yes (...?)
D: Is this the same all year around?
B: So apart from you, do all the keepers keep their animals exactly like you? Is this the usual rule?
L: No, do you think everyone will keep them in the same way?
B: Well, he is saying that everyone around here cannot maintain the same rule like he is maintaing!
D: Pardon?
B: What he is saying right now is everyone around here who maintain the livestocks do not maintain the same rule to house livestocks like the way he is doing right now.
D: Ok. So it's different for them. So does he keep his in the same way all the year around?

B: Do you keep them in your own way or follow the other people around
L: No, I keep them in my way.
B: He has it's own maintenance procedure to house his livestocks.
D: Pardon?
B: He has his own maintenance procedures to house this livestocks.
L: They are kept in a certain way so that they don't get cold or feel hot.
B: He is saying that he has to be very specific and careful whether they are getting any kind of flu in winter, That's what.
D: Ok. And who looks after the livestocks.
B: Who takes care?
L: I do as well as my wife.
B: No one else?
L: No.
B: Well he usually takes care of the livestocks and in his absence his spouse takes care of the livestocks.
D: spouse? Okay. Great. Does anybody else from outside of the household that look after them?
B: Anybody from outside your house who comes to take care of them?
L: No.
B: There's no one like that.
D: And do people have particular roles or does everyone do everything
B: Do you and your wife take turns or?
L: We do as per our time.
B: Well it depends on the spare time, if he is around, he is home, he maintains!
D: Could you ask him how he learn how to keep livestocks?
B: How did you learn how to keep them and feed them?
L: Actually I have learnt these by following internet from mobile. Also taken advices from the doctor, the deadly disease that they get is Ranikhet.
B: Ranikhet?
B: He said, he learnt it by surfing internet and some advice from the local doctors. And he worries about the most dangerous disease called the Ranikhet!
D: Ranikhet?
B: RaniKHET! It's some kind of synonymous (..?)
D: Ok. Erm, are these the same doctors as he gets his foods from?
B: The regular ones?
D: Yeah, can you ask him if he's the same doctor, he gets his foods from?
B: Do you go to the same doctor?
L: Yes.
B: He maintains the same regular doctor from where he gets his medicines.
D: OK. Umm. Can you ask him why he uses this.. this doctor?
B: Why do you take suggestions from this doctor, why not go to the other doctors?
L: Because the doctor is close by, also when there is a disease, if we give him a call, he can come here. There is no one. (..?)
B: What he's saying basically, his stays very near, first of all. Second of all in any kind of

emergeny if he calls him, he will be here in few minutes. And 3rd one is availability. There is no other doctor, right now here, other than him. Or else he would have to go to Diamiond harbour which is far away from that, to get another one, that's why he maintains the same doctor.
D: And..umm..could he tell me. Can you tell him I'm going to ask him about..umm.. can you ask him if he knows the term, antibiotic?
B: The vaccines you give..
D: Not vaccines!
B: They won't understand the difference between antibiotic and vaccine!
D: Can you ask the same (..?)
B: Do you give antibiotic?
L: Yes we give!
B: Yes now he is understanding!
D: So he knows antibiotics? That's great!
D: Can you tell him, I'm going to ask him about his use of antibiotics?
B: Can you tell something about antibiotics, what antibiotics you use? When you give?
L: These big ones d get their antibiotics. The doctor tells us. 2 drops.
B: By big ones you mean turkeys?
L: Yes turkey!
L: And the small ones get 1 drop of (..?). The first antibiotic that we give is usually dropped in the eyes.
B: Eyes?
L: Yes and the second one is given in the mouth! And the third one is injected near the wings! After the use we dispose them by burying under the soil far away so that the virus can't be spread!
B: What's he saying, that in case of turkeys they use antibiotic doses 3 drops. In case of turkeys just because they are bigger.
D: 3 drops?
B: 3 drops! They uses the liquid form. They use the liquid form one in the chicken concept, the indigenous chickens, they uses 2 drops. And in the turkeys they usually use one 1 drop in mouth..on cheek as a vaccination in the armpit. In this case, this is the rule.
D: As a vaccination?
B: Yes, as a vaccination!
D: What do you mean by vaccination?
B: what he is saying is about the antibiotics.
B: You are talking about antibiotics? (Questioning the keeper again)
L: Yes
B: He is talking about the antibiotics.
D: But vaccination is different from antibiotics.
B: Yes he's talking about antibiotics not vaccination.
D: You just said vaccination so..does he mean vaccination or does he mean injection?
B: When you give injection is that vaccine or antibiotic?
L: Vaccine.
B: No I'm asking about antibiotic.
L: Oh, antibiotic is what we just put in the water.

B: He uses the antibiotics with the mixture in the water!
D: He puts them with the water?
B: Yes!
D: Okay! When I said I will ask him about the antibiotics did you him what he do with the antibiotics? If I say I am going to ask him about it just tell him I am going to ask him about it, don't ask him about it.
B: I didn't ask it. He just started with the antibiotic and convert the entire converstaion into vaccination. (..?) I confirmed that!
D: So I will start again, so can you explain what he does when one of his animals gets sick?
B: When your animals get sick what do you do?
L: If they are sick, the problem is they discharge white faeces, the chickens look sick on seeing that we go to the doctor immediately. The doctor gives medicine like antibiotics and some tablets, I can't recall the names. We are asked to dissolve them in water and give them. After giving them they recover.
B: What he's saying, when he sees the symptoms of sickness just like slowing down, have any kind of dysentries, then they usually call the doctor. Doctor comes around, have a look, diagnos, then prescribe some antibiotics and (..?)
D: they get healthier?
B: Yes!
D: So this doctor (..?) who provides the treatments? As in who actually gives the treatments! Who administers the treatments?
B: So when the doctor visits, who administers it? Government or administrative department? Who funds it?
L: I think the Government gives.
D: Pardon?
B: He says the Government adminstrates!
D: Adminsters means? Give the medication to the animals?
B: Do you give the medicine or the doctor gives.
L: No, we get the medicines from the doctor and give them to the animals. (He gives the medicines, not the doctor)
B: He consults with the doctor, doctor gives him the medicine and he himself give the medicines to the livestocks.
D: Okay, is this same for both the chickens and the turkeys?
B: Is it same for indigenous chickens and turkeys?
L: Yes for Indigenous chickens and turkeys it's actually the same. Antivirus is same! Antivirus is used for the stool. When there is whitish stool discharge or loose motion then we give antivirus!
B: What's he's saying, in both cases it's kind of same. Antibiotics are given when dysentry and some kind of weakness they can see.
L: Also due to the use of vaccine our birds have very less diseases! Very less! Almost there isn't any bird diseases!
B: So he is taking about vaccinations.
D: So now vaccinations?
B: So he is saying that they use vaccination in proper way that's why disease is almost not

there! They are healthy.
D: Could he describe what vaccines and when he gives them
B: Can you tell what types of vaccines you give and when?
L: Vaccine..umm.. I have forgotten the name, also I didn't write the name. They give a file (which is of 100 power), we don't give it entirely. There is a date. We give them as per the dates and then we stop giving it to them. We throw them away. Bury them!
B: Actually he forgot the name of the vaccination. Doctor gives them in a this kind of container. You can feed that to 100 chickens but in one case...if he sees, if there is 1 or 2 livestock or chicken is sick, they usually use particular for them, for those chickens. And after that he threw them away. He doesn't use that.
D: OK, Sure. So he gives the vaccine after they become ill?
B: You give vaccine after they get sick?
L: No, you can't give them vaccine at the time they are sick. When they are sick you can't give them vaccine. Only after they recover from the sickness we can give them vaccines. When they are sick, they are first treated, after they recover then only we give them vaccines! Vaccines doesn't need to be given every time. They already got 3 vaccines, again after 6 months they will get vaccine. That is in the coming month they will get their vaccines.
B: Okay, let me tell him now. He's saying. if the livestock is sick you cannot vaccinate it. You have to treat them properly afterwards you can vaccine them. And they have already given the vaccine.
B: When did you give the vaccine you said?
L: It's almost six months now!
B: It's been almost six months, they have done (he meant got) 1 vaccine. (Basically the keeper said 3!)
D: Ok, Alright. Can he describe the last situation where he gave the antibiotics?
B: Exactly when was the last time you used antibiotics?
L: Antibiotic was used two months back. (..?) there was some loose motion 2 months back, They gave antibiotic capsule. The capsules were dissolved in water and given.
B: He's saying that it's been two months that he used antibiotics. He was given two tablets (Capsule!). He was supposed to mix that with water and fed the livestocks, because the symptom was dysentary type.
D: So it was mixed with water. So was it for the chickens or for the turkeys?
B: Did you give it to the chickens or turkeys?
L: Both turkey and Indigenous chicken.
B: Both cases.
D: Both? How often what he say he has to normally treat?
B: At what intervals you get them treated? After how many days or months?
L: We don't treat them until there's a sickness.
B: Unless they are not sick, they are not treated!
D: Ok, sure And is it normally antibiotics that is the normal treatment? What is the normal treatment?
B: What is the normal treatment you go for? To keep them healthy?
L: To keep them healthy, during summers ORS water is given. And some medicinal herbs are fed! Also neem leaves!

B: Ok, What's he is saying, to keep livestocks healthy, he usually use, during summer ORS solutions with water and there are some kind of vegetables there, I don't even know the name of them. He usually makes them consume to maintain the livestocks healthy!
D: Is it as a treatment he gets those?
B: treatments?
D: Yeah, was that as a treatment?
B: Do you give these as a treatment?
L: Yes to keep them healthy.
B: To keep them healthy.
D: To keep them healthy (..?) He described for what reasons he uses antibiotics?
B: Can you tell me for what reasons you use antibiotics?
L: It is used so that there is no loose motions. You know about pox? So that they don't get pox. Apart from this, there is something else which is done, in 1L water 2 cloves of garlic and few inches of ginger is added and boiled till it is reduced to 500ml or 100ml and then the water is mixed with the livestock water, so that they don't fall sick or get diseased.
B: what's he is saying right now is...that...when he exactly sees the symptoms of the dysentery and the sickness, at that point he uses those medicine or antibiotics and afterwards he uses the conventional way to maintain the livestock health. They generally take 1L water, 2 pieces of garlic, a bit of ginger and make it boil, till the entire solution comes to 500ml and they feed them! (NO! They mix this reduced solution to their water and then feed them)
D: So that doesn't include antibiotics?
B: No. He's telling me about a remedy to maintain the livestock healthy.
D: Ok, yah. So when and what reason does he use antibiotics?
B: So can you say again, what are the reasons you use antibiotics?
L: Antibiotic is used for runny stool/loose motion. Chickens have drowsiness as sickness, to give them relief from such sickness it is used. Also to get cured from pox! So to get relief from these 3 diseases, antibiotics is used!
B: He basically used antibiotics to stop dysentry, chicken pox, there's a common disease he is talking about, it is called chicken pox! And there is a disease, he is talking generally, livestocks generally slows down or gets unenergetic sometimes. So if he finds these kinds of symptoms, in these cases they use particularly antibiotics!
D: Ok, and is it easy for him to get antibiotics?
B: Do you get these antibiotics quite easily?
L: Yes! It's there in every store, in local medicine shops. You can get them. Other than these you don't get anything else.
B: he is saying it is too much available in each and every medical store surrounding.
D: Does he use it on the advice of his doctor or is he.. yeah ask that question.
B: He can continue, I can compile later.
D: No, No ask this one!
B: Do you always buy these antibiotics only after the doctor prescribes you?
L: Yes after discussing with doctors.
B: Each and every..after taking the decision he buys, all these medicines.
D: So without (..?) with the doctor?
B: So you don't buy anything without consulting with the doctor?

L: No
B: ..
D..(..?) Do you ask him if he's ever got antibiotics for animals from his human doctor?
B: Have you ever got antibiotics from human doctor instead of animal doctor?
L: Yes!
D: Yes? Can you ask him why?
B: Why did you take it?
L: Well, the antibiotic for humans and the livestocks are same!
B: I see! He's saying that antibiotics which is regularly used for humans and livestock is same.
L: Rest everything is different!
D: So he doesn't see.. can you ask him why is this perception of human and animal antibiotics are?
B: Perceptions?
D: Yeah
B: Don't you think that they are different?
L: No! No!
B: No, he doesn't see any perception that there is adifference.
D: He doesn't see there's a difference? Okay, that's pretty interesting.So does he ever gets advice about his animals from doctor? (..?) Does he ever take advice about treating the animals.
B: So you take advice from your doctor?
L: Yes I do.
B: He every time takes advices from doctor.
D: From Doctors? Okay sure! Can you ask him who these doctors are?
B: You mean names?
D: Nah no names..No.. just the.. like are they...could you ask them if these people are qualified?
B: The people you take your medicines from, are they have degrees of veteniary?
L: Yes
B: Well he said they are qualified and they have a degree.
D: They have a degree?
L: which is why I was never at a loss. The number I bought, they just remain so, not one dies.
B: He say that they are having degrees and they are qualified that's why everytime he consults with themand brings medicines from them, the medicines on the advice of the qualified doctors, that is the prove that his livestock is healthy! Every time!
D: Okay..erm.. does he know how antibiotics work? Could you ask him?
B: Do you know how antibiotic works?
L: Yes, after consulting with the doctors I got to know antibiotics work on loose motion, pox, from cold. These are the things.
B: what's he's saying that he knows from the advices of the doctor is to prevent dysentery, prevent from cold and coughs they use antibiotics. But he's not saying how to use, how does it work.
D: Ok. Does the doctor tell him how long he would be using them?
B: So the doctor tells you for how many days you will use?
L: Yes, he tells how many times I have to give it to them.
B: He always mentions about the time duration, every time he is provided. (with the

medication)
D: The duration. And does he tell how is the amount that should be given?
B: Amounts means money? Or the dosage?
D: No. Amounts means the dosage, yes!
B: How much to give, when to give, all these things are told by the doctor?
L: Yes! It's not even that expensive. It's just 12 rupees! 2 capsules is just 12 rupees.
B: What he is saying that every time he is mentioned about the dosage by the doctorsand he is saying that medicines are cheaper, around 12 rupees each of a unit
D: Does he see how much of the medication he should give?
B: Does the doctor tell you how much to give and what not to do?
L: Yes the doctor tells, in 1 L water, 2 capsules are to be dissolved!
B: well, yes doctor everytime consults with him, use this in 1 L water with 2 capsules and dilute them and then feed them! (..?)
D: Ok, erm, and they say how many days (..?)
B: For how many days you are asked to use?
L: For two days.
B: Two days? They usually use for two days.
L: We get the result in two days. In one day only we get the results if they are recovering or not.
B: Generally what he is saying is you will see results within one day. They continue, or the doctor tell them to continue for more 2 days? Total 2 days.
D: And does he always use it every 2 days?
B: Do you always use for 2 days?
L: yes two days!
D: Has he ever noticed that it not worked?
B: Did you ever notice that the medicine didn't work? Has it ever happened?
L: No it didn't happen to me.
B: He hasn't got any experience like that. Every time positive!
D: Ok sure! Could you ask if has used any (..?) for animals and in people?
B: Human and Animal?
D: Yeah
B: Have you used any such medication that worked both on animals as well as on humans?
L: No. I only use that Antibiotic. I actually get them for chickens! It's a medication for humans. Rest no other medication will work on them which are for humans.
B: Well he's saying that human antibiotics sometimes doesn't work on livestocks, that why most of the time he buys after consulting with the doctors, and specially for livestock medicines. And he knows that human medicines sometimes doesn't work on livestocks that's why he doesn't buy.
D: Ok, great! Does he use livestock antibiotics in people?
L: (Irrelevant discussion with wife)
B: The medicine you buy do you use them on yourselves when you are sick?
L: NO! (But in the background the wife says "yes'!)
L: How can I do, medications for animals will not work on humans.
B: That's the thing I was talking. Livestock medicines cannot work on humans (..?)
D: Ok.. Erm.. So do you go to the same provider for your animals and for your family?

B: we are almost done, only 2/3 qustions are left. We know you are getting late!
D: Could you ask him if he goes to the same provider for his livestock as well as his family?
B: Do you go to the same medical shop?
L: Yes
B: Yes the provider is same every time!
D: Could he explain why does he goes to this provider
B: Why do you go to the same shop every time?
L: I get better results, My chickens are not dying, getting the perfect medications.
B: He's getting the best result. (..?) going to the same retailer or provider. Because he thinks, the provider is giving him the best and genuine medicines. So his livestock is healthy. That's why he goes to the regular places!
D: Ok, good! I forgot to ask him one question. So could you ask him if the eggs.. the eggs he gets from both the turkeys and chickens.
B: Do you get eggs from both turkey and indi-chickens?
L: The indigenous haven't given eggs yet but turkeys are giving.
B: So when they lay you get them from both?
L: No the indi-chickens are not laying. It's almost time, after few months they will lay too,.
B: Its seasonal, it's not about right now! The indi-chickens re not laying eggs, it's not their season time but turkeys are giving the eggs, that's why he is taking the eggs from turkeys! It depends on the season.
D: OK. But he gets eggs from both?
B: Yes! But it's seasonal!
D: 1 last question. Which I forgot to ask. Does he consume meat and the eggs, his family do they consume them?
B: Apart from the business do you or your family eats the meat of these birds?
L: No my family doesn't eat the meat! We sometimes eat the eggs. No one eats the meat. We don't use meat for every type.
B: He's saying usually they often consume eggs.
L: I ate, I loved it!
B: They often consume eggs but except him the entire family doesn't consume meat (..?) but he personally eat those meat and he finds them tasty.
D: ok. great. Thank you very much!
THANK YOU
D: Can he show me the antibiotics?
B: Do you have any bottles or containers for antibiotics?
L: No it's in capsule form and now I don't have! No we throw them away.
B: They use and throw them away.
D: Okay no problem thank you very much!
