# Supplementary material for: “If It Works in People, Why Not Animals?”: A Qualitative Investigation of Antibiotic Use in Smallholder Livestock Settings in Rural West Bengal, India
Source: Antibiotics (Basel). 2021 Nov 23;10(12):1433. doi: 10.3390/antibiotics10121433 (PMC8698124; doi:10.3390/antibiotics10121433)
Supplement: Supplementary file 1 [file antibiotics-10-01433-s001.zip › Supplementary S1_ Interview Transcripts/Site 1/Para-vet 1 (site 1).pdf]

Code for Study - 'If it works in people, why not animals?': A qualitative investigation of antibiotic use in smallholder livestock settings in rural West Bengal, India: Para-vet 1, Site 1

Date: 26/07/2019

Location: Site 1

Interviewee: Para-vet- Antibiotic Provider

Interviewer: Dominic Day (DD)

Translation: Somraj Das (SD)

Transcription: Sayak Manna (SM)

Q: Interviewer (DD)

A: Interviewee (paravet)

START OF INTERVIEW

Q: Thank you very much for agreeing to answer my question.

A: You are welcome.

Q: So, the first question I would like to ask….. what's your role in the community?

A: My role around this community is as a para vet means medical support towards the animal health.

Q: And who do you normally serve?

A: Villagers who rear animals.

Q: And is it normally the same people coming back many times or lots of different people?

A: There are two kinds of things, the new people come and old people also come around the year

Q: Ok, and what is the area do you cover?

A: Around 5 KM.

Q: ok, and it's including [village name redacted] village?

A: Yes, it's including [village name redacted] village.

Q: ok, Aaa Could you describe about your carrier in this profession?

A: Well, it's been 5 years in this profession and I usually visit the emergency case in day or night, it doesn't matter. I usually like to serve them on an emergency basis…… that's all to say.

Q: And what is the common problems people call you for?

A: Basically, people call me for first-aid but when the situation gets worsen, I use to take advice from my teacher and generally I do not handle serious or major cases. I use to refer it to the Veterinary Officer.

Q: Okay, okay, and why did you choose this carrier?

A: Well, I generally like this profession because I think that educated person gets scared to go around the livestock and that is why I choose this profession.

Q: Okay, hm hm and what did you do before this?

A: Unemployed.

Q: Thank you, so could you tell me about your antibiotic uses?

A: I do not apply the potential antibiotics. I generally use easily available safe antibiotics. In serios problem where it needs to use potential antibiotics, I used to refer to Veterinary Doctor.

Q: Okay, could you explain which antibiotics you normally use?

A: Tetracycline, Enrofloxacin, Ampicillin-Cloxacillin etc.

Q: Ok, thank you and what diseases you normally treat?

A: Basically, I treat very normal diseases and incase of serious diseases I use to refer.

Q: Could you explain what do you mean by normal diseases?

A: Fever, diarrhoea, retention of placenta etc.

Q: Okay, thank you and so do you stock any medicine that is required in human and animals both?

A: Yes, I use penicillin.

Q: And for which disease?

A: Black quarter. And I also used Avil (Chlorpheniramine maleate).

Q: And when you prescribed antibiotics do you administer it or give it to owner to administer?

A: I usually told the animal owner how to apply it.

Q: Are they follow your instruction?

A: They usually abide by the instruction.

Q: Could you explain what your usual instruction are?

A: I told them to administer the medicine very carefully.

Q: Do you explain that how much and how long they should give it?

A: 3 days twice daily.

Q: And why do you suggest this time?

A: For better remedy and sometimes I told to extend for few more days when the situation gets worse or referred it.

Q: And why do you think that it sometimes doesn't work?

A: I think that the same antibiotic which was administered few days ago doesn't work for the next time.

Q: Okay, so do people ever ask you for advising about human health?

A: Haha (Smiles) Yes, sometimes occurs

Q: For what reason do they normally come?

A: Basically, for joint pain.

Q: And do you give advice?

A: No, no ...(smiles), sometimes people make mock of me.

Q: Okay…(smiles) and is there any situation where you give people antibiotics?

A: No no.

Q: Um, is there any situation where people have used antibiotics which were given for animals?

A: No but sometimes I advised them this drug are for animals not for human use.

Q: Why do you think that people should not use this drug?

A: Because both the drug is made of different things and for different purposes.

Q: Do you know what is the different between human and animal drug?

A: I heard it sometimes but I do not have the specific idea about it.

Q: Could you explain how the antibiotics works?

A: Antibiotics make antibody and in this way they works.

Q: Is there any situation that you refused to give antibiotics?

A: Yes, in pregnant animal and in some normal cases.

Q: What do you mean by normal cases?

A: Cases where without antibiotics the animal can be recovered by other non-antibiotics medicine.

Q: What are the things other than antibiotics?

A: Non-antibiotics.

Q: Such as?

A: Melonex, Paracetamol, Nimesulide etc.

Q: Is Melonex contain meloxicam?

A: Yes.

Q: Okay, if I asked about your training do you have any problem?

A: No, I don’t have any problem.

Q: Could you describe your training?

A: Well, there is a farm in [*Life history redacted]* where I got my first training then from our local Veterinary Doctor around 4 years ago.

Q: Who provided this training?

A: There is a doctor in [village name redacted*]* named [*Name redacted]*.

Q: So, for training you have to stay there or not?

A: Yes

Q: And for how long did he train?

A: 1 month.

Q: Could you describe what type of training is this?

A: Animal husbandry, artificial insemination.

Q: So, is that training valuable for what you are doing now?

A: Yes, it helps me to grow better quality.

Q: What do you mean by better quality? I didn’t understand.

A: I can face some critical problem because of that training.

Q: Do you continue your training during your job?

A: Firstly, I was trained then I got the job.

Q: Do you have any training now?

A: Actually, I am working with doctor so when face any critical situation, I asked him for his advice.

Q: And are you part of any professional association?

A: No

Q: Could you explain that which kind of drug can be used in human and animal both?

A: I never use any animal drug to human but sometimes I used human drug in animal disease.

Q: Is there any human drug other than penicillin you used in animal?

A: No.

Q: Is there any situation that you prescribed any antibiotics in humans?

A: No, as I said earlier.

Q: Well thank you very much for your help
