# Supplementary material for: “If It Works in People, Why Not Animals?”: A Qualitative Investigation of Antibiotic Use in Smallholder Livestock Settings in Rural West Bengal, India
Source: Antibiotics (Basel). 2021 Nov 23;10(12):1433. doi: 10.3390/antibiotics10121433 (PMC8698124; doi:10.3390/antibiotics10121433)
Supplement: Supplementary file 1 [file antibiotics-10-01433-s001.zip › Supplementary S1_ Interview Transcripts/Site 1/Public-private VPP 1 (site 1).pdf]

Code for Study - ‘If it works in people, why not animals?’: A qualitative investigation of antibiotic use in smallholder livestock settings in rural West Bengal, India: public-private VPP 1, Site 1
Date: 11/07/2019
Location: Site 1
Interviewee: Public-Private VPP 1 (Pranibandhu- Antibiotic Provider)
Interviewer: Jean-Christophe Arnold (J-CA)
Transcription: Debanjan Debnath

START OF INTERVIEW

I: Can you explain your role to the people of [village name redacted]?

P: We work as Pranibandhu for the people of [village name redacted]. It is also my own village. I was born there. The villagers, my neighbors have been close to me! I was born there, I got my veterinary training from there, I treat animals in the village, the villagers respect me. And they call me for treatment.

I: Whom do you treat?

P: The village is extremely poor. They depend on their daily wage for their day expenses. They are not rich. We help the animal’s owners in the community. They are very needy.

I: So, whom do you treat? Animals?

P: Animals.

I: Other than [village name redacted] where else do you provide treatment?

P: Apart from [village name redacted], I would look after the entire GP, now I have come to [village name redacted] (a neighboring village), I look after the entire GP.

I: Can you describe your career up to now?

P: It was good.

I: Could you explain it?

P: I was very needy then. We had farming land in the village. then after I took my Higher Secondary Exam, I assisted the veterinary surgeon for some time, then they put me in touch with the Pranibandhu initiative. This is how I came into this career.

I: How long have you been doing this work for?

P: A long time, minimum 20 years.

I: Is it govt. service? or private?

P: It's private. We have been trained by the govt. We have been given some instruments, and we work with that. We, Pranibandhu from all 21 blocks, also protested against the govt. so that they help us, there has been no help. They say you have been made independent by the training. We had signed a paper saying we couldn't demand a job from the govt. The way there's "Sikhabandhu" (Friends of Education), "Krishibandhu" (Friends of Farming), they get no salary. Still "Sikhabandhu" gets some sort of salary. The para-teacher, I mean. But we get nothing. They would give a small incentive; it's also stopped now.

I: Do you provide antibiotics for the animals?

P: Yes.

I: Do you keep antibiotics with yourself?

P: Yes.

I: Which antibiotics do you stock and why?

P: We keep Enrofloxacin, Ampicillin, Cloxacillin, Terramycin, procaine penicillin, Terramycin tablet… things of this sort.

I: Why?

P: In our area the kind of cows you see and the sort of illness they get, these are the antibiotics that work the best.

I: Do you stock any human Antibiotics?

P: No, I don't keep it with me. If it's needed, I get it from the market.

I: Do you buy it yourself?

P: We might prescribe it to the patient, and they get it. Sometimes we, ourselves, get it from the store. They might say that they couldn't buy it.

I: Why do you chose to treat animals with Human Antibiotics?

P: In case of small animals, like dogs, goats etc. the veterinary antibiotics are of high power. For example, they are 3 grams. I would need 1 gram. So, I can't use them, so I need to use human antibiotics.

I: How are these drugs given to the animals?

P: In most cases the drugs are injected. while feeding it, the drugs are dusted and mixed with water and given to the animal using a bottle.

I: What I am asking is in which form the medicines are given? as Injections, you mentioned.

P: Yes Injections. Otherwise we would prescribe the drugs for the patient party to get.

I: In which situations would you use antibiotics in animals?
P: For fever. There's an illness called B2 in cows, where the leg gets swelled, in those cases we use 20L or 40L penicillin. Or Metritis, prolapse and etc. In case of delivery, if there's a lot of blood loss or hemorrhage we use high antibiotics in those cases. We use Ce.

I: Do you use drugs differently in different species of animals?

P: Yes, for different animals we use different drugs.

I: How is that?

P: For cows, for example, we use drugs of Ampicillin Group.

I: were you saying something else?

P: No, so for cows we use that. And for dogs, since they are delicate, we can't force the drugs like we do in cows. So, if we use those antibiotics there, it won't work. The client would get annoyed if it needs to be done more than once. Hence, we use higher antibiotics there.

I: What about chickens and goats?

P: There aren't poultries here as such! People who keep chickens and seek treatment, they don't get vaccines. For example, if someone has two chickens, they won't get them vaccinated. When "Ranikhet" occurs *[local term for Newcastle Disease*], then drugs like Suldil or Sulmet, or Ciprofloxacin powder or Hostacycline powder is used.

I: what about ducks?

P: People rarely seek treatment for ducks here. We usually use drugs like Terramycin on them.

I: When you're using an antibiotic how do you decide which antibiotic to use?

P: For example, in case of cows we measure the temperature using the thermometer in the rectum. And if the cows have cough, or cold, has respiratory infection, we use drugs from Ampicillin group.

I: Do you use antibiotics for any other reason? (other than treating illness)

P: No, no! No other reason.

I: When you prescribe an antibiotic to the clients, what do you tell them?

P: People who raise cows here, they have no idea about them. It's not possible for them to know which cow has which illness. We decide ourselves, and accordingly we give them suggestions. They can either get the drugs themselves, or we keep antibiotics with us, we would instantly give the treatment. Or they may buy it and get someone else to do it. Because they are completely unaware, how would then know what problem the cow has. They depend on us.

I: How do you know if the treatment has worked?

P: When the cow is sick it stops eating, that's what we have learnt through our experience and through learning medicines. Whenever a cow has some serious disease it will stop eating, its regular life will be interrupted. It will be tired. After using antibiotics if we notice that the temperature has gone down, and the cow is eating from the next day, we understand the drug is working.

I: When you realize that some medicine is not working, why do you think that happens, and what will you do in that situation?

P: In case the medicine doesn't work (...) there's no system here to get the blood of the cow tested. it doesn't happen in this country, might happen in other places. From our experience, what we do is when an antibiotic doesn't work, we provide a higher antibiotic. For example, if the cow has a simple cold, but has developed some respiratory infection, have facing breathing problems then we would change the antibiotic and use something stronger.

I: Why do you think it doesn't work at times?

P: If we use the same antibiotics repeatedly, the cow develops resistance.

I: How do you think animals become resistant to antibiotics?

P: What do you mean?

I: How does antibiotic resistance come?

P: If you use the same antibiotics repeatedly over time it stops working. For example, the medicine called Butox which is used for ectoparasitic infestations in Cattle has been used ever since I started my career as a veterinarian, but it doesn't work anymore. The same way the insects in plants they grow resistant to same kind of pesticide if used over time.

I: Have people ever come to you for their own treatment?

P: No! Sometimes even if they do, we say no! we are veterinary doctors, we don't treat humans.

I: Why don't you help people with their problems in those cases?

P: (laughs) there are many human doctors here, and not many veterinary doctors. So, I took up on this career because I realized the animals here are deprived of good treatment and they die without proper treatment. So, I wanted to only treat animals (cows) not humans. It makes me feel good.

I: What's the difference between animal antibiotics and human antibiotics?

P: As much as I know, human antibiotics are 500mg, or 250mg, Ampicillin for example, but in case of cows, it's 3g or 3000mg.

I: Do you know of any other differences?

P: I know this much that a cow would need a higher dosage. Both of them are of the same Ampicillin group, but veterinary medicine is very powerful, and human medicines are just 500mg, not that much.

I: Are there any situation where you wouldn't provide antibiotics despite being asked for it?

P: As long as we have it, we give them. When I don't have them, I prescribe it and they get it from somewhere else. We don't always have a stock, and veterinary medicines are very expensive. One 3g Amoxicillin is 106 Rupees. Amoxicillin or Floxacillin. We don't have a lot of capital. We do whatever we can manage, besides there are other expenses, such as in this house. (we prescribe the medicine, sometimes we also get it from the store ourselves"

I: When you prescribe some antibiotic, do you mention that it's just for animal use?

P: No, no one is interested to know. There aren't such people here. All they care about is the health of the animal.

I: (laughs) what i want to know is, when you give drugs to the animals, do you mention it to the client that it's just for animal use?

P: No, we don't say that. But no one wants to know if the medicine is for animal use. Since we treat cows, the clients know the medicines are for cows.

I: What sort of training did you receive?

P: We received training on artificial insemination as "Pranibandhu".

I: anything else?

P: And we were trained for piggery at [village name redacted].

I: Do you have to follow any regulations on drug use?

P: No, we don't follow anything as such! but there are some seasonal cases that we take care of every year. For example, at the end of the season, when it rains cows have diarrhea for that we prescribe medicines (name of the medicine is indistinct).

I: According to your training as Pranibandhu (and such) what sort of drug or treatment are you authorized to use?

P: The training we received as Pranibandhu has been on artificial insemination. We weren't asked to prescribe medication. If you ask me now how you are practicing, we are only supposed to do vaccination, and artificial insemination. But if we did just that we won't make enough money, so we, on our own, have learnt how to use Antibiotics from other veterinary doctors. For example, we would accompany them during their visits and learn which antibiotics is used when. This is how we leant it.

I: Did you any other training?

P: No, just the pig and Artificial Insemination.

I: Do you have opportunities for more training?

P: No. There are no opportunities. The govt has given us nothing of that sort.

I: Would you be interested if there were opportunities?

P: I'd be very interested. I am very eager to learn. If you help us find an opportunity, I am truly very interested. Especially for dogs.

I: Do you have any idea on guidelines on antibiotic prescription?

P: No, we don't know.

I: Are you a part of any organization?

P: No!

END OF INTERVIEW
