# Supplementary material for: “If It Works in People, Why Not Animals?”: A Qualitative Investigation of Antibiotic Use in Smallholder Livestock Settings in Rural West Bengal, India
Source: Antibiotics (Basel). 2021 Nov 23;10(12):1433. doi: 10.3390/antibiotics10121433 (PMC8698124; doi:10.3390/antibiotics10121433)
Supplement: Supplementary file 1 [file antibiotics-10-01433-s001.zip › Supplementary S1_ Interview Transcripts/Site 1/Public-private VPP 2 (site 1).pdf]

Code for Study - ‘If it works in people, why not animals?’: A qualitative investigation of antibiotic use in smallholder livestock settings in rural West Bengal, India: public-private VPP 2, Site 1
Date: 12/07/2019
Location: Site 1
Interviewee: Public-Private VPP 2, Animal Development Volunteer- Antibiotic Provider
Interviewer: Jean-Christophe Arnold (J-CA)
Transcription: Debanjan Debnath

START OF INTERVIEW

I: What's your role for the people of [village name redacted]?

P: People are mostly either farmers or livestock owners in the village. There are quite a lot of cows in the village. Whatever advice you'd need, from the birth of a calf, how to raise them properly, so that they give more milk, get impregnated on time, won't be a loss, won't fall sick, we help the villagers with all of that. Since we are private, they have to give us some fee too. Let me start with the treatment, they have to be given deworming medicines from one month from birth, then give 6 kinds of deworming medicines in the next 6 months. With good species of cows, we advise they have to give them good food with the right nutrients. If someone can't give calcium, the limestone that is used for coloring the houses, I advise them to soak it in water and give that water to the cows. If people can't the deworming medicines on time, we ask them to go to the GP. If cows are fed properly, in 17 months they will be ready to be impregnated. Then we'll do Artificial Insemination. With regard to treatment, antibiotics are used more than it was before. Antibiotics should be used as less as possible. but we have to use it still, because of the competition. If we can cure the patient fast, then they would call me. If it takes such a long time, they would want to seek other doctors. But when I used to cover a large area only by myself, it wasn't like this. In case of fever we would use drugs like paracetamol. and eventually if the drugs aren't working, we used antibiotics. Now, in pressure of the competition we use antibiotics to treat the disease faster. Whenever we use antibiotics, we have to keep in mind that antibiotics are very harmful for the health of the animal, so we have to also give vitamins. So, the cows are healthy. This is how it goes.

I: You said the competition is increasing, whom are you competing against?

P: I have been the only veterinarian here for 25 years. Then there came the GP vet, now there's [name redacted- public private vpp 1], who's also from the same village though now he lives somewhere else. The GP vet is changed every two years. So, for the popularity whomever can give the most time, see it's two in the afternoon that I haven't eaten, so I have to go whenever I am asked. It builds a good relationship. I also try to prevent the diseases. For example, BQ, HFMD harm the cow's health a lot. And the treatment is also quite costly. And you have to give antibiotics. So, we give treatment in advance!

I: Whom do you treat?

P: All the animals. Cattle, Chicken, ducks, etc.

I: Who else do you provide treatment for outside of [village name redacted]?

P: I go to all the villages in 4GPs. I am a AD (Animal Development) volunteer in [GP name redacted- Site 1] GP. In 1947 when I started, I was the only one in the entire 8 GPs. Five of us got the training but rest of them couldn't do this job. It's more useful that the veterinary doctor would go to the household and give treatment, because the owners can't otherwise bring their animals to the clinic always. When we go the houses and give treatment, the animals are treated better and they

don't die the way they used to. The govt. hasn't given us permanent jobs. Noticing how effective we are, the government introduced a new initiative called "Pranibandhu". [name removed- public-private vpp1] is Pranibandhu. They don't have a certificate, we have one. We also have more training.

I: What's the training?

P: Animal development volunteer. There were few parts to it. We were made customs after some training, then after more training we were supposed to give a position called LDA (Livestock Development Assistant) but it didn't happen because of several political schemes. But the ones who were finally given a 1-year diploma training and posted as LDA, they are much less experienced that we are. We are much more popular in the area. People don't see them much, because the animals end up dead, and they have less practice. And since they are govt. employees and they get a salary they are not as responsible. For us, this is our source of income, we take care of the animals so that they survive, the clients and I would depend on each other. That's how we make money. We spend time explaining what is good for the animals, and less expensive. Since we were so effective the govt has introduced Pranibandhu to do Artificial Insemination. After Pranibandhu arrived our area for operation has reduced. That's where the competition come from. Even though I wasn't very qualified, I would try and figure out how different diseases were to be treated, from one doctor to another I would roam around and work with different surgeons. Hence, I am so experienced that I do a lot of operations (caesarian etc.) too. But the new comers who come after training get posting don't operate. They take us along to do operations. this is how it's done!

I: Are you a private practitioner?

P: Yes, definitely private. I am a [GP name redacted- site 1] GP ADV. The Artificial Insemination that we do, get the tools from the govt. We are affiliated to the govt., but we don't get a salary.

I: What did you do before becoming an AD volunteer?

P: After completing school, I used to work at a human pharmacy, then this scheme came along. I knew people in the community for the good work, so I was suggested to be a part of the ADV scheme when it was suggested. So, I have always started to help the people, improve the community, and make money in the process in care I don't get other jobs.

I: Do you keep antibiotics?

P: Yes.

I: Can you describe which antibiotics drugs you stock?

P: From the beginning Oxytetracycline, Ampicillin, cloxacillin, gentamicin, (indistinct) and the latest ones, marbofloxacin. The antibiotics that work well are stocked.

I: Why do you stock these particular antibiotics?

P: Earlier we would not use so many antibiotics, we would just use anti-analgesic or anti-pyretic but now a days these medicines don't work. That's why we keep the antibiotics. For example, mastitis would get cured just with enrofloxacin, now "ceftriaxone sulfa trim" isn't even working. Now we have to use latest antibiotics (marbofloxacin) that are also very expensive, each of them is around 800 rupees. Still they don't always work.

I: Why do you think the drugs aren't working?

P: We first give treatment for a few days and we see that it's not working.

I: Yes, I am asking why do think that happens?

P: Now the food that's given to the animals aren't nutritious enough because everything is made artificially. So, the antibiotics aren't working anymore. The resistance is growing.

I: What do you normally do when you see that the treatment isn't working (when you see the medicine you've given isn't working)?

P: In case of Mastitis for example, we first give primary medicine. If it doesn't work, we go the second stage, and then to the last stage. Then it eventually works. As I said before enrofloxacin would work, but now even ceftriaxone sulfa trim isn't working. Then we have the latest ones like marbofloxacin. Then it works.

I: What do you mean by Higher Antibiotics?

P: According to the time, the latest antibiotics.

I: For which animal do you give antibiotic treatment?

P: All of the animals. The cows, goats, even the chickens. There are some vaccines for diseases like R2BF1, pox, etc. But people don't get the vaccines because of the money. They want to do whatever they can without spending money. Without vaccines the chickens can't be saved, then we have to use antibiotics.

I: Which animal do you give antibiotic treatment the most?

P: The cows.

I: Why the cows?

P: Because we treat the cows the most. They also see the diseases in cows the most.

I: Apart from treating the diseases, are there any other reasons you'd provide antibiotics for?

P: No.

I: Do you stock any human antibiotics?

P: No, we don't keep it.

I: Why not?

P: We don't treat humans, but at times we keep human antibiotics for the animals itself. Also, sometimes I'd just give medicines (for gas) to people around me if they need it. There are certain cases where we use human medications in animals. For example, in case of dogs we'd generally use human medications (of all kind, antibiotics, deworming medications). If I need veterinary gentamicin/oxytetracycline and I don't have it, so I buy human gentamicin and use.

I: As you mentioned you sometimes don't have animal antibiotics, why don't you have it?

P: I am chiefly an animal doctor; I don't have the degree required to treat humans.

I: Why don't you have the medicines?

P: Human medicines...

I: No, Animal medications.

P: No, it's not that we don't, we do.

I: As you mentioned that sometimes you might not have veterinary medicines.

P: In case my stock for the day is over, or I treated more patients than usual. It'd take a long time for me to go to [nearest town name redacted], or it gets delivered from Kolkata. So, I will get the human medication from the store and get the work done. We have to stock most of the medicine in advance. We can't afford to go to the store to get it immediately.

I: What's the difference between human and animal antibiotics?

P: The cows need more 10x powerful medicines. For example, we'd have to use a 2ml human gentamicin in a cow, we'd have to use 5 of them to match the need. it becomes more expensive than the veterinary alternative of the medicine which is 30 ml. That's why we don't keep human antibiotics that much.

I: For what problems in cows would you use human antibiotics in animals?

P: Well the first problem would be that I don't have the veterinary medicine. earlier the human antibiotics used to be less expensive. So, we would use human medications more, now we don't anymore. For example, we would use comostar to stop bleeding there was no veterinary alternative, so we had to use human medications. Now it has come to the market, so we use that. For example, dicyclomine hydrochloride was not in veterinary medicines so we would human medications in high quantity. But now that it's there we don't have to do it anymore, and we have to spend less**.**

I: When you're giving medicines how are they given to the animals?

P: If we are injecting it, it's in that form. If clients ask for it, we'd go and give the antibiotics three days in a row. But then there are side effects, so we give vitamin injection. And if they can't afford to call us every day then we give oral antibiotics.

I: When you give antibiotics who feeds them?

P: The owner feeds them. Some of them dust it and mix it in the water and give it to the animals. Some of them just mix it with food. Some just wrap it in a leave and give it.

I: When you are giving the medicines to the clients do you tell them how the medicines should be given?

P: Yes.

I: When you give antibiotics to the clients do you tell them how the antibiotics work in the animals?

P: Yes, we discuss it openly so that they know and get interested. I tell them how the antibiotic functions or how the disease won't go unless you give the antibiotics. We try treating with other medicines, if they don't work, we tell the clients that we need to use antibiotics now. We tell them what sort of medicine they can use for giving antibiotics to the animals and we also keep a sample. wait let me show you.

[he shows the administrating bottle]

I keep four bags, I keep antibiotics, vitamins, etc. there. I keep this bottle to give the medicine to the cows. the thick bottom and narrow neck of the bottle helps, I have also removed the metal ring

around the neck. I suggest that clients also get a bottle like this and feed the animals as the press their tongue down. Because if this goes into the breathing pipes cows might get pneumonia. They would need different medication then, and sometimes, around 1 percent, might also die from this. So, I make sure that they understand.

I: Do the owners know that you're giving antibiotics?

P: Yes. Every time. I explain which are antibiotics, medicines for fever, or for stomach issues, deworming medicines, digestive medications for bloat. Everything is explained.

I: Do you help people with human healthcare?

P: I help people with their problems voluntarily sometimes. I used to work at a human pharmacy. And one of my skills is that I can identify which human medication can be used in cows, I can use human medication to treat animals. So, with the experience has helped me gain a lot of knowledge. I am as good as a surgeon. Because they don't want to give the time. For example, during delivery the surgeons don't want to go, and they will ask the clients to go for a caesarian which is extremely expensive. I do these jobs. The surgeons don't want to handle the dirt. That's why people call us more. And to answer your question I keep the medicines for pain and gas and fever, when someone is hurt or in need, I would give it.

I: Do you also give antibiotics like this

P: I can give saline to people, so people ask me to come if they need saline. So, I do like this in this area a lot. Even I can give a IV and other critical injections (which are to given in the joint) unlike other doctors and practitioners in this area, despite having been trained in Kolkata. Some people need regular injections I would go and help them voluntarily free of cost.

I: Have there been any situation where you've given humans animal antibiotics?

P: No.

I: You mentioned that you helped people with their problems by giving injections or saline. What sort of experience do you have that enable you to do this?

P: I give IV, saline. When I used to work with the doctor, I saw how to give saline. Then doctors would send us to do his job here and there. That's how I learnt.

I: Do you anything about guidelines of antibiotic use?

P: I have learnt a little bit from books. I don't remember all of it. All the antibiotics have side effects. I don't know which effects they are. That's why we give vitamin along with antibiotics to minimize the harm.

I: Do you have any idea about regulations in antibiotic use?

P: There are some antibiotics that work in three days. Some antibiotics take 5 days, some take 7 days. Some antibiotics need to be given twice a day, every 12 hours, some 24 hours, some 36 hours. That's how it's used. The course that I had ADV, it was on some diseases. we have been given training on veterinary fast aid so we can practice.

I: Did you have any additional training?

P: Piggery training, how to raise pigs. AI, and things of this sort. We would then report on the problems we are facing during practice. And after obtaining that report they would give us refresher training. Two-five days each year. They let us know how the diseases should be treated.

I: Are you a member of any organization?

P: No.

I: How long are you doing this job for?

P: [life history redacted].

I: We are finished.

END OF INTERVIEW
