# Supplementary material for: “If It Works in People, Why Not Animals?”: A Qualitative Investigation of Antibiotic Use in Smallholder Livestock Settings in Rural West Bengal, India
Source: Antibiotics (Basel). 2021 Nov 23;10(12):1433. doi: 10.3390/antibiotics10121433 (PMC8698124; doi:10.3390/antibiotics10121433)
Supplement: Supplementary file 1 [file antibiotics-10-01433-s001.zip › Supplementary S1_ Interview Transcripts/Site 1/Veterinarian 1 (private) (site 1).pdf]

Code for Study - ‘If it works in people, why not animals?’: A qualitative investigation of antibiotic use in smallholder livestock settings in rural West Bengal, India: Veterinarian 1, Site 1

Date: 18/11/2019

Location: Site 1

Interviewee: Key Informant (KI) and Antibiotic Provider. A retired government veterinarian with a pharmacy next door, now practicing privately

Interviewer: Mathew Hennessey (MH) accompanied by Pabak Sarkar (PS)

Transcription: Soumen Samanta (SS)

MH/PS: First tell us something about you. How you have been attached with this and how long are you doing this work?

KI: You want to know the relation with livestock owner with me?

PS: No, totally. Means how did you start this treatment? How did it start? Everything if you kindly tell us.

KI: I am a son from a farmer family. I am attached with cow, goat, and poultry, duck, all since my childhood. We had 25-30 cows, 50 goats, 20-25 poultry, and 20-25 ducks also. *Redacted personal history.*

MH: What work do you do now in retired life?

KI: I am retired now, I don’t do that (much) practice. I manage those cases which the pranibondhu can’t cure, or those cases farmer seeks for suggestion, those cases I tackle. Those complicated cases, chronic, severe cases where the farmer not get the results. Not many cases, less.

PS: so the pranibondhu come to seek suggestions from you?

KI: Not pranibondhu, they also come for advice; also those cases which the farmer not gets result from pranibondhu or other doctors; I treat those cases. The practice which you mean sitting the whole day, I don’t do that. The complicated cases, old cases. Some farmers also directly comes; farmers mean farmer of khatal (Small dairy farm), they come to seek advice I advise them. I reduced my practice since last 4 years, after my slip disc operations I can’t walk more properly. The practice you say I don’t do that.

MH: Why would they come to you rather than pranibondhu?

KI: They know they can’t get that much service from them (pranibondhu) so to whom I am previously known they come directly.

PS: When you worked for the government were you posted at Sarisha or other places?

KI: previously at [names of two different districts redacted], and so many districts; at the last stage I was posted here.

MH: Where do you see the animals?

PS: Where do you see the sick animals? Do they take the animals to you?

KI: They come with previous prescriptions, I see the details; which I think that I have to see the animals or who request much I visit their places.

PS: otherwise? If you see that in prescriptions..

KI: if I think there is possibility I prescribe; and those I think I have to see the animals I go there.

MH: How many pranibondhu do you work with?

PS: He doesn’t work with pranibondhus.

MH: sorry, how many pranibondhu do you have contact with?

PS: In this area how many pranibodhu do you know?

KI: if you count it’s 25-30 persons.

PS: How many works at [name of Site 1 GP redacted] among them?

KI: In [name of site 1 GP redacted] there are 2 pranibondhu. And experienced person is 1, he took training from [name of institution redacted] 18days training; short course training 15days. There is one, named [name of para-vet]. And pranibondhu is 2, [names of pranibandhus redacted].

PS: Is there any person whose house is not in [name of site 1 GP redacted] but treats at [name of site 1 GP redacted] area?

KI: [name of pranibandu redacted] made house recently in side [name of adjacent GP to site 1 redacted]; and [name of pranibandhu redacted]’s house may be at [name of site 1 GP redacted].

PS: Which village?

KI: opposite side of the village [name of village redacted].

PS: [name of an area in site 1]?

KI: No, that is the other side. I can’t remember the name at this moment. Just in south.

PS: [name of an area in site 1 redacted]?

KI: [name of area in site 1 redacted].

PS: and [name of pranibandhu redacted]?.

KI: Both of their houses is in [village name redacted].

PS: Where do [name of para-vet redacted] lives?

KI: Same village or side village.

MH: What type of contact do you have with them?

KI: I have not that much contact, if the face critical cases they ask me. Rarely. Like 7 day interval or 15 days interval or once in a month as cases may be. Ask me what to do or not.

KI: They may come or ask sometimes over phone.

MH/PS: Are there other persons who work on animal health care here other than pranibondhu?

KI: Recently one post govt. created “Pranisebi”, ‘pranimitra’ they do.

PS: Is there anybody like this in [name of site 1 GP redacted]?

KI: Yes. May be I don’t know properly. They have been posted since last 1year.

PS: Where can we get information about them can you tell please?

KI: All clear information about them you can know from BLDO. [name redacted]. He has all information who is posted and where they are posted.

MH: Are there any organizations of pranibondhu?

(KI: she came from [name of village redacted], 2-3 village apart, asked how she can increase milk)

KI: No.

PS: No groups work on animal health here.

MH: Does the pranibondhu form any organizations?

KI: not organizations but may be an association which is involved with the movements/ (protest or lobbying to government about their demands).

MH: Do you name that full?

KI: No, I don’t know the full name of the association, if you want to know I can ask over phone.

MH: Is the shop next door is yours?

KI: not mine, it is of my son. Connected.

MH: Is that veterinary drug shop and feed shop?

KI: Yes.

(MH instructs something to PS about questions type)

PS: Can you say how the medicines come, who are related with that? From where does the medicine come here?

KI: from wholesaler it comes.

PS: How many wholesaler..

KI: No wholesaler here, they are at [names of two towns outside site 2 redacted].

PS: These two?

KI: Yes.

PS: What is it's (shop) name?

KI: [name of shop redacted] medical hall and the other one is [name of shop redacted].

PS: In [name of town outside site 2 redacted]?

KI: [name of shop redacted].

PS: So antibiotics also come from them?

KI: Yes.

PS: Is there any of them whom you take more antibiotics from?

KI: As per requirement, not more or less.

PS: so all your medicines come from these shops. There is no specificity.

MH: Why did you choose one store you instead of the other store?

PS: So how or when you select this shop and when the other?

KI: One may go out of stock, have relation with both the two.

PS: So if you want to take, whom do you call first?

KI: both of them have good relations with us; I want to take from both of them. It is not like that he is my first preference.

MH: Do you get more antibiotic from one than the other?

KI: No, no such thing is there.

MH/PS: We want to draw a map of from where the antibiotic comes to you and..

PS: What is the name of this shop?

KI: Medico.

MH: Do you get antibiotics from anywhere else other than these 2 places?

KI: No. sometimes I write but they take from other shops.

PS: How many shops like this shop are there in this area?

KI: One at [name of village redacted], and another at [name of village redacted].

The wholesales also directly give antibiotics to pranibondhu. They use it.

PS: Do the pranibondhu also take antibiotic from you?

KI: very less as the directly get from wholesales now.

One business people give them directly. One wholesaler shop is also there at [name of towb outside site 1 redacted] like [name of shop redacted] and [name of shop redacted], they give them directly.

PS: to whom they give?

KI: Pranibondhu.

PS: So the pranibondhu take very less medicine from here, there is one wholesaler at [name of town outside site 1 redacted] who supply medicine to the pranibondhus.

PS: Less means..

KI: those are not supplied to them.

PS: Okay, what type of antibiotics do the pranibondhu take or what type of antibiotics are used here?

KI: here the small dairy farmer use antibiotic themselves. They use Penicillin and Strepto-penicillin randomly without taking any suggestions. They when bring a cow and it is in stressed condition they use it for 5-7 days. I can’t say how much they are using. We forbid them not to use antibiotic unnecessarily. But the medicine companies goes there and making them understand if

cow is in cough and cold, give this antibiotic course, give this with that etc; they make them understand. If they come to us I say if it is not sick no need to give it (Antibiotic). But thy most of the time do that, if any sort of cough-cold seen they use Penicillin, Strepto-penicillin, amoxicillin, ampicillin.

PS: Where do they but it from?

KI: they buy…. The companies directly give them also. The company representatives give them directly, those thing I have heard.

These things are done by those small dairy farmers (of 'Khatal') but the village farmers don't do that. The small dairy farmers who keep 20-25 or 50 cattle (10-200 numbers also which KI later told) of cattle, they do it. In [name of town outside site 1 redacted] there are almost 50 numbers of such 'khatal' is there. The MR goes to them and make them understand. They do it for their profit (in business). When they (Farmer) see it's not working then come to me.

PS: Is there this type of 'Khatal' at [name of site 1 GP redacted]?

KI: In [name of site 1 GP redacted] very less. One man recently made a 'khatal' with 15-16 Gir cattle. I went there once. It is of not more than 2-3months old. No such other 'khatal; is there.

PS: Where is this Gir "khatal' in [name of site 1 GP redacted]?

KI: Go to [name of site 1 GP redacted] bus stand, contact with [name redacted];

PS: Can you tell the name?

KI: Ask there, they have ships. Go bus stand ask for khatal, I can't remember the name now, it is of 2-3days relation so the name is not coming in my mind. Tell there who is having ships, very rich person, made 'khatal' recently. See in bus stand there is one manufacturer of bus body parts; opposite to that one ground is there, there it is.

PS: I know the place but not the khatal.

KI: it is of 3months old khatal. He brings good cows. He brought one bull also.

KI: Do you want to go those large 'khatal'?

PS: We are mainly now working at [name of site 1 GP redacted] area.

KI: No large 'khatal' is there. In [name of adjacent GP redacted] you can have lots of these small dairy farms.

MH: How do those cattle owner made contact with the medical representatives?

KI: they themselves find who is having 'khatal' and go there.

PS: Who? Company people?

KI: Yes. (the MR themselves find the khatal and go there). They go to one farm then ask him who has farms like this.

MH: Are the MR comes from different companies?

KI: Yes.

PS: Can you say some of those companies name?

KI: [names of five pharmaceutical companies redacted], many others.

PS: These all contact ‘Khatal’ directly?

KI: I don’t know whether all goes or not; I know [name of pharmaceutical company redacted] goes. [name of pharmaceutical company redacted] goes. They go I know but can’t say clearly who properly goes. The small companies also go.

PS: Can you say some name of the small companies?

KI: [name of pharmaceutical company redacted] not that much, I have to ask my son; he keeps those information.

MH: Do these MR give them antibiotics directly?

PS: Do these MR directly give them antibiotic or they say that here you can get antibiotic?

KI: They do sampling. (give samples). Some may sell, I don’t know.

PS: So mainly they do sampling?

KI: yes.

PS: Then from where they ask to buy the antibiotic from?

KI: It is difficult to say.

(KI’s son enters, he asked him about the small companies; he said that no such go nowadays, previously [names of three pharmaceutical companies redacted] used to go, now they don’t go.

(KI offers MH tea)

(One farmer came and ask the doctor a case of mastitis in his buffalo, the doctor ask whether to write costly or cheaper antibiotic; the farmer replies costly; and also says he himself gave Amoxicillin but not get results; and ask the doctor for higher antibiotic than that. The KI wrote an injection and ask him to give feedback 3days later.)

[PS ask something to an MR (of [name of pharmaceutical company redacted] company) who came to visit the doctor (inaudible)]

(The MR telling the doctor about a product [brand name redacted] (Sulphamethoxazole + trimethoprim) and tells the dose as 10-15mg/kg intramuscular, long acting; and if necessary to repeat it at 48hours later and request the doctor to prescribe it in any types of infections.

Also tells about a product [brand name redacted] which is toxin binder cum liver tonic tells the dose as 40-50ml in cattle and horse daily once.

Also tells about [brand name redacted] which he tells to use it in udder problem and milk abnormality, and tells in prevention of mastitis to feed it daily one bolus for 10days.)

MH: When you work as a private vet; how private are in this area?

KI: private means as we get retirement we become private. (and one name like him he tells..Inaudible)

PS: And is there anybody who is posted in govt. and practice privately?

MH: May be there but who do practice it's difficult to say.

PS: So there is one solely private veterinarian in this area.

KI: No, no solely vet is here.

MH: Where do you get your medicine from?

PS: So you or other person like you, from where do you get medicine?

KI: from 3-4retails shops.

MH: Would you choose one shop instead of other?

PS: Do you take medicine or you have a shop?

KI: shop cum medicine.

PS: So for him it's a practice cum medicine shop. Others (Vet) get medicines from the other retails shops. According to the facility they prefer.

MH: Do the retail shop offer them credit or any incentive?

KI: not in credit but if has extra relation that thing is different. Like if I know you well, you are honest, then I can give you credit.

PS: If any doctor goes to a shop and ask medicine in credit?

KI: if a doctor goes he (shop) surely will give as he knows the money will not be wasted.

PS: If any common people goes?

KI: then He should think whether his money will come back or not? It's depending on the relation. But generally nobody gives in credit.

MH/PS: Is there other people your retail shop sells to other than pranibondhu, private vet, farmers (khatal) and local farmers?

KI: No.

PS: Suppose any shop who keeps animal feed; do they take medicine?

KI: No, feed shops don't keep medicine.

They keep the supplements i.e. medicine-cum-feed supplement. Keeps those few but not more.

MH: No antibiotics?

KI: No, like Vitamin A it is feed supplement and also a medicine.

MH: So we are not missing anybody who takes medicines from retail shop?

KI: No such.

The farmer takes medicine as per prescriptions either from block or other vet doctors or from LDA.

MH: Do they come sometimes without prescriptions?

KI: Yes, sometimes. When the not get the doctor at night or out of office hours. Then comes like animal is having diarrhea what can be done.

MH/PS: Do the khatal owners also take medicine from retail shops? I am telling about antibiotics.

KI: Not much, they also take it after doctor's advice.

MH: Who is the biggest customer from the retail shop among these 4 (pranibondhu, private vet, farmers (khatal) and local farmers)?

KI: Number of prescriptions if you count it more general local farmer; but if you see quantity it's khatal farmer.

MH: If we think about volume what percentage goes to the khatal farmer?

PS: From a shops total sell of antibiotics, how much goes to the khatal farmers?

KI: It not calculated that way.

PS: Assumption.

KI: 60-40 i.e. 60% to khatal and 40% to general farmer.

PS: And the doctors? How much percentage?

KI: Around 5%

PS: So khatal 60%, Vet 5%, rest local farmers.

MH: So what about pranibondhu?

KI: Pranibondhu- 10%,

LDA, compunder and doctors -10%,

Khatal farmers -50%.

General Farmer -30%.

MH: Out of the total sell in the shop what proportion are antibiotics?

KI: assume it as 5-10%. Rest 90% is others like feed supplements, dewormer etc.

MH: Is there any differences of profit from how much they get from antibiotics and from other medicines.

PS: Which is having more profit?

KI: feed supplements.

MH: What are the main types of antibiotics do you sell?

KI: Ampicillin, Amoxicillin.

PS: Is it quantity wise?

KI: Yes.

PS: Which are the costly antibiotics?

KI: Costly means Marbofloxacin. And in medium range Ceftrixone, Ceftiofur.

PS: In which conditions you need to use these costly antibiotics?

KI: Mastitis, Pneumonia.

Enrofloxacin is low cost medicine; previously it was used more, now it is reduced.

PS: Among these which antibiotics are used for poultry?

KI: Tetracycline, oxy tetracycline, doxycycline, trimethoprim, levofloxacin; in poultry very less. Previously it was more now it has reduced.

PS: Previous means?

KI: 20years back.

PS: Why it has reduced?

KI: Many things are there, previously many poultry were here; later when the eggs were imported from Andhra (Other state); may be there some business policy. They started to give at low cost, and their (local) farmer run in loss then they stopped farming, etc. Many people say this. It may be the fact. The mash cost has also increased. . May be there some other reason needs research to find it. Previously huge poultry were here, like in 20 out of 50 families. 20 -25 years back.

(PS telling KI where he will go to take interview in his next days & KI wanted to introduce MH and PS to BLDO if they go with him to the BLDO office today.)

MH: Do you have any concern of antibiotic qualities of different companies?

KI: Yes, I think there are different qualities. I am telling my real experience. I went Makka for Haj (one religious event). There my wife's ocular infections occurred. We went to buy Moxifloxacin eye drop. They said one India made product (Moxifloxacin) is there which is 100rupees and one USA made product (Moxifloxacin) which is 1000rupees. Same medicine same drop, one is 100rupees and another is 1000rupees. So I asked why is there so difference in cost and they said definitely there is a difference in it's salt, efficacy. So I believe there is a company difference.

PS: Is there any parameter to judge the quality?

KI: It's practical (experience), practically some part and some as mental.

PS: Mental means 'if price is high quality is better' this view?

KI: It's honesty of the company.

PS: So he thinks reputed company produce quality antibiotic.

KI: It's mental. There is no system of examining the antibiotic quality in laboratory in India. There is no good laboratory here. And this is not done I think. So for that practically you have to see which one is effective and which one is not working. As for example I give one company's Amoxicillin it's not working; in same type case other company's amoxicillin I gave it gave good result. It doesn't mean that the previous one is bad quality, it may be that it's sensitivity was lowered, the cow was in stressed condition or any other reason. The salt composition is also can't be differentiable and also sometimes it has to differentiate. Like from my previous time we were

using antibiotic of [name of pharmaceutical company redacted] (now [name of pharmaceutical company redacted]). Then there were 3 antibiotics- Penicillin, Strepto penicillin and the other one is Oxytetracycline. These 3 antibiotic was there in market. Those produced very good result then. Almost 95% was successful. Now they are in the back line. It can't be dependable now; that may be due to change in microorganism or may the quality have reduced. One thing I think the Calcium, previously it was produced after processing the oyster. Now those are not available so not produced. Then calcium was needed for 10 cows now calcium is needed for 100cows. They are getting less vitamins. So the it is being fees admixture product. So they have to feed the feed supplement. We are seeing that collection (of oyster) is not happening but the supply (of calcium) is fulfilled. So has it that much strength? So the calcium which could be for 10days, it is given for 100days now. It is natural I think. Previously we collected those oysters but now those are not found (very less). It may be vanished due to environmental pollution. So from where they are giving the supply? Previously the dead cow's bones were collected for calcium production. So where is it's source. They may adulterating it or may dividing it's amount. So it is matter of thinking; what is the source. Like you say [brand name redacted] made from oyster shell, where they are getting it from? Those (oyster) are not found in Bengal, may be you have to found in other states or country. (repeating the story)

MH: Are there certain manufacturer which you think produce lower quality medicine?

PS: You were telling about faith, is there some company which you can believe and to some not?

KI: you can believe. But upon small company there is less faith. You can't say bad unless testing it. I think [name of pharmaceutical company redacted], [name of pharmaceutical company redacted] they are big companies, they should not produce lower quality medicine. They should maintain the quality (for their reputation), these all I think. The small company producing lower quality you can't say; you come to know that after using that; whether working or not. This [name of pharmaceutical company redacted]'s amoxicillin works, so you can't avoid it. So small company means low quality you can't say. They produce well; sometimes we see in newspaper about adulterated/fraud medicine. Those are dangerous. If medicine goes directly to wholesale then to small shop; if any conspiracy occur in this chain then it problematic. They also have been caught sometimes.

PS: Is there any incidence like that in this area?

KI: One product –[brand name redacted (Ivermectin) was copied from one company in the middle. They couldn't copy it total get up, if you observe minutely; 2-3% they couldn't copy then you can caught it. When I was posted at Malda, one product of [name of pharmaceutical company redacted] was copied; they could not copy the logo properly. The thief must leave 1% clue by which you can catch him. It's all over the world. In Kolkata once one steel product has been caught by seeing it's logo; almost crore of rupees. Which company mostly runs in market they copy their product.

MH: When human antibiotics are used to treat animals?

PS: Does it happen that human antibiotics are used in animal?

KI: yes. I do it sometimes. Suppose in dog, do and human dose almost similar according to body weight. If I find the antibiotic is not available in veterinary then I use the human one. Mostly in do it is done. And suppose in village area the veterinary antacids are unavailable; in that case I say [brand name redacted] syrup (human antacid) 100ml to feed or to feed 10tablets.

PS: In antibiotics? Like [brand name redacted] (metronidazole)..

KI: Metronidazole is very much available in veterinary now. What I use is Azithromycin, it works better; azithromycin is not available in veterinary. I use it sometimes. I used in dog and birds; it gives good result as it is not used previously. It is not that like randomly used, use when needed.

MH: Where would you get the human drugs from?

KI: Human retail shop.

MH: Are there any times when people may have to take animal antibiotics?

KI: No.

MH: What happens if antibiotics are expired?

KI: they take it back.

PS: Who?

KI: the wholesaler who give that. They return it back.

MH/PS: As our project is to make interventions of antibiotic use (proper use). What are the main challenges? Or is there any way by which we could make interventions?

KI: Farmers are doing it regularly. Suppose today I wrote him amoxicillin or Ceftriaxone in cough and cold, he did that and got result; so next time when it's happening he don't come. I don't take money or they give forcefully. Or some comes to take advice. They think if I go to doctor I have to spend 200-300 rupees; so they do it themselves. If you want to prevent it, how can you stop them; they do it to reduce expense. He uses the antibiotic in similar way for 5days and if see it not works then come to doctor again. Most of the times it work. Some clever farmer do it. Like if gas problem they face take antacid, if headache, take paracetamol. If it is not a rich country it is very difficult to control. Rich country is America; I don't know much there it (system) is. I don't think that it can be prevented even if after making them aware. Because it is available in market; if it was not available in market (then it could be different). Now even the grocery shop keeps it now- like antacid, metronidazole. It is impossible to stop, as it has already spread up to grass root level. It is possible if all people are rich, if they are become conscious like paracetamol is safe but also

to visit a doctor before to take it. This is the thing. It’s impossible to stop. They all have ego; like they think doctor has knowledge, they also have experience. Whatever he learns he try to apply first. If not works (then he comes), these are impossible to stop; you have to think a lot how to stop.

MH: Okay.

PS: In [name of site 1 GP redacted] you have told one new khatal has been started; besides that where can we get such place in [name of site 1 GP redacted] where more number of animals or poultry we can get? We are searching both small farmer and large farmer.

KI: many small farmer you can get there, as it is rural area. Many have 15-20goats or 4-5 cows.

PS: if you can say 1-2 of their names we can meet them later even if not today.

KI: near [name of village outside of site 1 redacted], he keeps many goats.

PS: That is outside of [name of site 1 GP redacted].

KI: oo, yes. You know the alcohol factory, the village at the opposite side of that, there many farmer are there who keeps more number of animals. I can’t say about last 2 years, if you go and search you can get. There beside the river if you ask a person that who has 10-15 goats, you can find.

PS: Like this, poultry of 30-50 numbers?

KI: 15-20 poultry you can get; but the 50..

PS: Who are using mash like feed, such..

KI: now who have 5-10 birds they also take mash from here. They know the benefit of mash. Here we sell A grade mash. You can ask [para-vet name redacted] or [pranibandhu name redacted] (pranibondu) as they roam around (practice inside) the village. They can tell. Large farmers comes very less, that are in other side. May be there are 50 birds household they can say you.

MH/PS: Do you have any question for us?

KI: my question is here our govt. offers MCH course, and there they provide FRCS at London. What is the course difference in MCH and F.R.C.S.

MH: that is MRCVS, ..you have to practice as a vet in UK, you just need a (bachelor?) degree for masters.

PS: What kind of courses they offer?

KI: Masters of veterinary public health, masters in one health epidemiology or

PS: Is there any critical care or surgery..

MH: Then you have to get a certificate, it needs 2years (?),.. or you can do specialized residency (?) at the university for (inaudible).. you can get the diploma in that field. Then people call you a specialist.
