# Supplementary material for: “If It Works in People, Why Not Animals?”: A Qualitative Investigation of Antibiotic Use in Smallholder Livestock Settings in Rural West Bengal, India
Source: Antibiotics (Basel). 2021 Nov 23;10(12):1433. doi: 10.3390/antibiotics10121433 (PMC8698124; doi:10.3390/antibiotics10121433)
Supplement: Supplementary file 1 [file antibiotics-10-01433-s001.zip › Supplementary S1_ Interview Transcripts/Site 1/Veterinary drug shop 1 (site 1).pdf]

Code for Study - ‘If it works in people, why not animals?’: A qualitative investigation of antibiotic use in smallholder livestock settings in rural West Bengal, India: veterinary drug shop 1, site 1
Date: 12/07/2019
Location: Site 1 in nearest town and closest veterinary-specific drugs shop to livestock keepers of Site 1
Interviewee: Veterinary Drug Shop Keeper- Antibiotic Provider
Interviewer: Jean-Christophe Arnold (J-CA)
Transcription: Debanjan Debnath

START OF INTERVIEW

I: What's your role for the people of [village name redacted]?

P: There are a lot of houses with cattle in [village name redacted]. The Pranibandhu’s treat the animals in the village. They send some of their clients to my shop for the medicine, and sometimes if they can't manage, they refer the clients to my shop. So, the people get the treatment here.

I: Do people of [village name redacted] come here directly without consulting the doctor?

P: Many of them know that they get good treatment here. We hear their problem and give medicines accordingly. and it works! they only pay for the medicines, and consultation comes free.

I: For what reasons would people usually come here?

P: In most cases, they come for pox and diarrheas in Goats, mastitis in cows, and general cold and fever is there. There's a kind of diarrhea which is caused by flukes. A lot of people come for that.

I: Flukes?

P: It's a kind of a worm that grows in the liver, the Pranibandhu’s can't diagnose them. After suffering for a few days, they would send the patient here.

I: Do you give antibiotics to animals?

P: Yes, I do! I used to practice as a veterinarian.

I: For what problems in animals would you give antibiotics?

P: For fever, cold, diarrhea, mastitis, skin diseases in cows, hand foot and mouth disease, these generally.

I: Do you see the animals before you give them medication?

P: No, I don't see the animals. I base my diagnosis on what the client is saying. I use my experience to understand what the clients are trying to say and what might have happened. They are not always right, since I used to practice, we have an understanding as to what the disease might be.

I: How do you decide which antibiotics are used in which situations?

P: I know which medicines are used for what. From our experience we know which medicine will work best in a certain case. For example, for stomach issues ofloxacin works well. From our experience we know in which cases, which antibiotic would work faster. We also had to study

books regarding treatments, and all of that contributed to where I am now. I have an experience for over 15-20 years.
I: What sort of experience do you have in veterinary practice?

P: I used to practice on field. I did it for about 7-8 years. I have also done a three-year diploma on it. So, eventually it happened.

I: Which antibiotic do you store?

P: Oxytetracycline, Ceftriaxone, Ceftiofur, Ofloxacin, Amoxicillin, Ampicillin, Enrofloxacin.

I: Anything else?

P: Apart from this we have Sulphur Drugs. Trimethoprim, Sulfadiazine.

I: Why do you keep these specific antibiotics?

P: These are the ones that work well! In primary cases Enrofloxacin or Sulfadiazine or Oxytetracycline gets most of the work done! Then the latest ones like Ofloxacin, Cetirizine are used for quicker action. The client that can afford it, we give them a bit more expensive antibiotics such as Ceftriaxone, Ceftiofur, Ofloxacin. These drugs are a bit expensive. The less expensive ones are also good. If people can bare the expenses, we give them the better ones. So, it keeps both.

I: How do decide on the duration of antibiotic use?

P: Usually we give for three days. If the patient doesn't get better, we extend it for three more days. Total 6 days. While the treatment is going on, it depends upon the condition of the patient, if the antibiotics needs to be changed, we do that, or if we see that the patient is getting better, then we extend it for three more days. That's how it's done.

I: We extend the course if the patient doesn't get better?

P: If we see that the medicine is working and the patient is 50% better, then we extend the medicine for three more days. If it doesn't work, we change it to higher antibiotics after three days. That's how it works.

I: Why do you think people come directly to you for treatment?

P: I am good at diagnosing, compared to others. So, patients come to me directly, they have been benefited before. And they might think we will get a better treatment here, that's why they probably come. Because from their previous experiences they know that if they come here, their animals get cured.

I: What do you think people think about the other vets? (the GP vet, the Pranibandhu's)

P: They delay the treatment deliberately and make the clients spend more. They want to make more money out of it. Maybe they could cure the disease in two days with better treatment, but they would make it last longer so that they get the fees. So, all these things work in them. That's why people in these areas aren't very happy with them. Since people don't have an option, they go to them first, and to come here is difficult because of the distance. But they don't like them, if they could they would come here instead. That's what they say. They say it takes such a long time to treat a disease. It almost costs half as the animal itself. If the cow is around 10,000 rupees, the treatment hasn't been good. That's why they are angry. But they don't have an option.

I: Do you stock human antibiotics?

P: No.

I: Does anyone come to you for human health problems?

P: No.

I: Why do you think that happens?

P: They know that it's just a veterinary shop. We specialize in veterinary medicine. So, they know that won't get help for human health here.

I: When you provide medicines to people, who gives it to the animals?

P: The owner of the animal would give the medicine to the animals.

I: They themselves give all sorts of medicine?

P: Yes, now when it comes to injections, for whom who can't give injections, call the Pranibandhu’s to give the injection in exchange for money.

I: Which forms are the medicines in?

P: They are in tablet forms, injection form, for poultry they are in liquid form. They are in these three forms.

I: When you give these medicines to the clients do you explain how it should be given to the animals?

P: Yes, yes, we explain everything. how to divide the dose, etc.

I: When you are giving antibiotics to the clients, what do you tell them if you can give an example?

P: When I am giving the medicine...

I: What do you tell them as to how ... (Interrupted)

P: How to feed them?

I: Yes, if you could give an example.

P: So, imagine there's one tablet for 80kgs. Now a goat is 15-20 Kgs. We ask them to divide it into three or four portions and have it twice a day. If it's an adult cow it'll be around 80 kgs, so one tablet in the morning, another one in the evening. that's what we tell them. According to the weight of the animal, we ask them to divide the tablet into smaller proportion. In case of Injection 1 ml is for an animal of 10 kgs, for a 100kg animal, they would need 10mls, everyday!

I: Have you ever told your clients how the antibiotics work in animals?

P: We tell them that if the antibiotics work in two days the animal will get better, then you continue the medication for two more days. In two days, you can understand whether the health of the animal is improving.

I: Do you tell them how the medicine is going to work in animals?

P: No.

I: Why not?

P: They just want to see the results, how the medicine is working is not important to them. All they care about is that the animal is getting better, how doesn't matter.

I: How do you understand that the treatment is working?

P: The animal wasn't probably eating, when it starts eating, we understand it's improving. When an animal stops eating, it must be very sick. When it starts eating again, that's the main sign that it's getting better.

I: But how do you understand that they are getting better?

P: They come and tell me.

I: How often do people come and tell you whether or not animals are improving?

P: I might give someone medicine for one day, then I ask them to come and give me the results on the next day. If the medicine works, then you have to continue medication for another day. We sometimes give medication for two days in the beginning, or even just one day. In most cases animal would respond to the medicine in one day, unless it's a critical condition. For animals that are in critical condition we suggest that they will have to be given medication for at least two days, then to come and give us the results. Sometimes people also take my number, so they consult over the call. Mostly they come and talk.

I: How frequently does it happen?

P: Within 24 hours.

I: If the treatment doesn't work, why do you think that happens?

P: If we don't get desirable results in two days, we recommend the patient to go and see the surgeon. Maybe there's something serious which you're not being able to understand, or I can't understand from here. Then, we put them in touch with the veterinary surgeon.

I: Why do you think the treatment doesn't work from time to time?

P: It hasn't been diagnosed properly. The client is not being able to say it properly. Because my treatment depends on what the client is saying. It's possible that he's not able to explain the situation properly. This is one situation. Or maybe what he thinks has happened to cow didn't happen, he doesn't understand why the cow is sick.

I: What do you do then?

P: We put them in tough with a veterinary surgeon and ask them to go for treatment. If they can afford it, they see the surgeon.

I: Which veterinary surgeon are you talking about?

P: There's a veterinary surgeon in the block.

I: Where's this block?

P: just two mins away.

I: Do you believe that people of [village name redacted] go to the block veterinarian for treatment?

P: There are health centers in [village name redacted], they go there.

I: Let me ask you the question again. And I am talking about people of [village name redacted]. When the medicine doesn't work what do you do?

P: We tell them to go to the healthcare center, if they can't solve the problem, they would refer to a senior doctor.

I: What do you understand about antibiotic resistance?

P: After overusing an antibiotic, it doesn't work anymore. that's what antibiotic resistance is. It might have worked before but for using it too much it doesn't work anymore. That's what we call antibiotic resistance.

I: Are there any situation where people have come to ask for antibiotics, but you haven't given them?

P: No, it didn't happen! If they ask, we give.

I: Why?

P: Why did we give? To cure the diseases the antibiotic was given. He wouldn't want antibiotic for no reason, the animal must be sick. That's why we give it to them.

I: Out of all the people that come here to medicine, how many would you give antibiotic treatments to?

P: The ones that need antibiotics, we give them. Sometimes people can't buy antibiotics because they are too expensive. We, then, ask them to go the health-care center, they get treatment for free there.

I: What sort of training did you receive?

P: I did a three-year diploma course from a private veterinary college. After that the veterinary surgeon in our area, I worked under him for two years. Then I practiced for around 7-8 years then I opened this shop.

I: Do you know any guidelines for antibiotic use?

P: Well, yes, we know which ones are safe for pregnancy, which ones work better in certain cases. For example, there are certain antibiotics for certain diseases, we know about them. We also maintain antibiotic dosage lest it's the animal is too weak.

I: Apart from these do you know anything about regulations and guidelines of antibiotic use?

P: No, nothing else!

I: Did you do any additional training for this job?

P: No, no other training.

I: Are there any opportunities for further training?

P: No, I don't have an opportunity and also, I don't have the time for more training, I am busy with the business and the shop. I won't be able to take up on such an opportunity even if it comes now.

I: Are you a member of any organization?

P: No.

I: Is this a private organization or Govt.?

P: Private.

I: Why did you decide to do this role?

P: I took training, and I studied, then I practiced as a veterinarian. People eventually began to know me, then I chose this as my career.

I: What do you mean by practicing as a veterinarian?

P: After people got to know about me, they would come to me for treatment when their cows got sick. They would call us, and we would take money in exchange for our services. This is how it started.

I: Did you have a license?

P: I had passed from a private veterinary college. I had that degree. To practice as a veterinarian, you don't have to get license here.

I: It's not required for someone to get a license in order for them to practice as a veterinarian?

P: No, the quack doctors that practice here, I have never seen anyone get a license. They just do it like that.

I: Thank you

END OF INTERVIEW
