# Supplementary material for: “If It Works in People, Why Not Animals?”: A Qualitative Investigation of Antibiotic Use in Smallholder Livestock Settings in Rural West Bengal, India
Source: Antibiotics (Basel). 2021 Nov 23;10(12):1433. doi: 10.3390/antibiotics10121433 (PMC8698124; doi:10.3390/antibiotics10121433)
Supplement: Supplementary file 1 [file antibiotics-10-01433-s001.zip › Supplementary S1_ Interview Transcripts/Site 1/Veterinary drug shop 2 (site 1).pdf]

Code for Study - ‘If it works in people, why not animals?’: A qualitative investigation of antibiotic use in smallholder livestock settings in rural West Bengal, India: veterinary drug shop 2, site 1

Date: 19/11/2019

Location: Site 1- identified to supply veterinary medicines to livestock keepers of site 1

Interviewee: Veterinary Drug Shop Owner- Antibiotic Provider

Interviewer: Mathew Hennessey (MH) accompanied by Pabak Sarkar (PS) and Dr. Indranil Samanta (IS)

Transcription: Soumen Samanta (SS)

MH- Matthew Hennessey

IS- Indranil Samanta

PS- Pabak Sarkar

SH – Stakeholder

XXXX – Name of shop

IS: Previously what did you do except this business?

SH: Nothing.

MH: What are the different types of medicines do you keep here? And what proportion of those are antibiotics?

IS: Do you keep medicine of all types animal; cows, poultry, all?

SH: Yes, all. Even 60kg tiger’s medicine also (of circus people).

IS: How much antibiotic do you keep now approx?

SH: Now it has decreased. Previously different types of antibiotic I kept.

MH: What type of antibiotic do you keep now?

SH: Ampicillin/cloxacillin, gentamicin, enrofloxacin, tetracycline and also some I have to see. Ceftriaxone, ceftraixone/sulbactam.

MH: Which of the antibiotic do you sell most?

SH: Ampicillin and amoxicillin. Oxytetracycline also.

MH: Whom do you sell those antibiotics to?

SH: To farmers.

MH: To what are the types of animal?

IS: All types of animal.

SH: All, except human. Now govt. separates veterinary, previously it was not. Previously in my father times human medicines were used.

MH: Do you keep any human antibiotics here?

SH: previously I kept, when my son was here. Not now. But it will be wrong if I say not. Two to four are there like pain medicine, eye medicine.

IS: I can see otrivin® there.

SH: Yes, some medicines are there. It will be wrong if I say not. I have to keep some pain medicine, injection of human. Now it has decreased. Govt. separates the human and veterinary sector.

MH: How govt. has done that change?

IS: From when govt. started this?

SH: Long ago.

MH: What did they do to implement the change?

IS: Actually his concept is like that govt. has implemented certain rules; that you can't use human medicine to veterinary purpose.

SH: But till now also human medicines are sold at veterinary. Now license is one for human and veterinary. But who now run a shop, for their advantage they keep in separate almira (cup board). We also can sell human drugs but have to keep separate almira (cup board) for that. But these kinds of shop (only veterinary) are less now. Most of the shop you will get veterinary with human.

PS: So why do you keep like this? (Not keep human medicine now)

SH: My son's time I used to keep both human and veterinary medicine. But as my son is not now, I didn't take the headache. Doctor used to sit here then (private chamber). My son has got a job so I do not take any headache now. If I get something to eat that's fine now. If I think more, there will be problem of blood sugar at this age.

MH: Who checks whether those rules are being followed or not?

SH: Drug control authority.

MH: How do they do that?

SH: Their force comes and checks how it needs to keep. They said these this front side of almira should be made up of glass. Now you can see in human counter also that it is made up of glass. Previously this system was not there. Now it should not be sold in open like previous, it should be under a permanent place (roof).

MH: How often would they come and check?

SH: It’s not fixed, in some year they come 3-4times per year or sometimes once in a year. If anyone is having problems they go to him repeatedly.

MH: Do they with warning or without warning?

SH: without any warning.

MH: If they found anybody is not following the rules, (what did they do?)

SH: give them warning. Suppose my almira is not having glass, so it’s a problem. They tell that within this date you have to put the glass on almira.

(Someone came and ask it whether it is a XXXX shop, the shop owner said yes. Then he asks for some medicine as his cow is giving less milk, and then someone from the shop tells that he is giving some medicine that would increase his milk. And he instructs the customer about how to feed the medicine, the owner talks with the customer. The customer brought a paper that one ‘ghosh’ {Who collects milk from a farmer} wrote about the shop name.)

MH: Where do you get these antibiotics from?

SH: Different wholesaler, company people.

PS: Company people themselves also come?

SH: Yes.

PS: Can you say some name of the companies who mainly gives these antibiotics?

SH: [names of pharmaceutical companies redacted]

PS: And how many wholesalers are there?

SH: Many. In [town name outside site 1 redacted] market and in [town name outside site 1 redacted]. I was also a wholesaler one time.

IS: Which one in [town name outside site 1 redacted] market? [name of wholesaler redacted]?

SH: [name of wholesaler redacted] and umm.(thinks).

IS: At [town name outside site 1 redacted]?

SH: And in [town name outside site 1 redacted] from [name of another wholesaler redacted]. His shop was founded after us.

(Some inaudible part. One customer comes to buy some medicine)

IS: And few companies also directly supply to him.

MH: How many these mixed company do you deal with?

MH: previously many were there. Now it has reduced. Previously all the company was connected to me once.

PS: Now how much? 4 or 5?

SH: Less, only 4 to5.

PS : And in wholesaler ? 2?

SH: From whom I want to take I take.

(One customer comes and consult to him for taking deworming medicine for his cow)

MH: (tells some in low voice)

PS: when you go to wholesaler and when to company?

SH: Its can’t be told.

PS: Means generally.

SH: How can businessmen say which product is needed when.

PS: If you need now, will you go to wholesaler or to company?

SH: I will see my advantage from where to take.

MH: So how many wholesalers he deals with?

IS: He has told already, previously it was many but now only 2.

MH: Are there some wholesalers to whom you do not deal anymore?

SH: connected to all a little bit; one is available to him, another to the other shops. We have to keep all types products. All products will not be available from a single wholesaler. Now company also increases and they also divide their some products to this shop and some to other shops. And some wholesalers also keep all types products.

PS: So now do you take from such a wholesaler where all types of products are available?

SH: It’s not fixed where I will go.

PS: So in case of antibiotic what do you do? Take directly from company or..

SH: From company.

MH: Do you take antibiotic from wholesaler too?

PS: Yes, take from them too.

PS: So if you buy 10 antibiotics how much from company and how much from wholesaler?

SH: It’s depending on the market. Like if in rainy season diarrhea, cough-cold some medicines needed much, in summer digestive medicine needed more. So it can’t be said. In that case doctor can say well.

IS: You tell please.

PS to MH: he is not sure about the proportions, like it varies from season to season, disease to disease.

SH: In India 6 seasons are there. So it varies in season wise.

MH: So he will go depending what he needs.

PS: Yes.

MH: So one person does not provide everything.

PS: Yes.

PS: So do you have to go and take these medicines or they all come and give you?

SH: Few come and few I have to go.

PS: In antibiotics?

SH: All same.

PS: So does the company come and gives more or the wholesalers more?

SH: It depends on.. Large company not comes to small distributors. Large company comes to those who are having havoc sell.

PS: So in that case from where do you take?

SH: For that wholesaler are there. And if problems are there from wholesalers for that retailers are there. Govt. has decided that so that nobody face problems.

PS: Do company peoples also come?

SH: Previously I took all from company directly. Now few companies come.

PS: Are they small company?

SH: Which one is small which one is large how can I say. Which one is large and which one is small can you tell me?

PS: SO if can say names.

SH: There are [names of pharmaceutical companies redacted] are these small or large?

IS/PS: these are large.

SH: Small which one? [name of pharmaceutical company redacted]'?

IS: yes.

SH: But now the sale of [name of pharmaceutical company redacted] is high in the market.

IS: Ok.

SH: Now most of the shop keeps more [name of pharmaceutical company redacted] products.

IS: What are the other companies like [name of pharmaceutical company redacted]?

SH: [name of pharmaceutical company redacted]

PS: So in [name of pharmaceutical company redacted] and [name of pharmaceutical company redacted], are the compmany comes or the wholesaler?

SH: Both, not fixed. If I need anything urgently wherever I get nearby I have to supply it from them, it may be wholesaler or retailer or company.

PS: Okay. In every month or week what is your general plan to take medicine from whom?

SH: That is fixed. (Either wholesaler or company)

MH: From what type of the retailers do you buy in emergency? Is a mixed retailer or a vet retailer?

SH: Will they come with medicine?

IS/PS: No, no. (Smiles)

SH: We are here to sell if you come with medicine. No need to worry.

MH: (Smiles)

MH: What type of the retailers do you buy from in emergency?

PS: In emergency the medicine you buy how far is that? [nearby town name redacted]?

SH: It’s a particular counter from Kolkata.

MH: If you buy from company, how do you deal with or how do you make contact?

SH: it’s from the company transport directly.

MH: Do you deal with any medical representative?

SH: Yes, all companies. If any product needs to promote; there is no retailer for that. PS: So how that come?

SH: Now govt. has started new rules that in hospital counter all the human medicine that are given all are generic. There is no representative for that. You have to take it directly from company. The profit in it, company takes from you 20 or 30% maximum. Rest 70%, company is giving to you for you to sell yourself.

PS: can you say any name of such company (called PCD company- promotion cum distribution)?

SH: [name of company redacted]’’

PS: Do they sell antibiotics?

SH: Who are having license they sell.

PS: This [name of company redacted]?

SH: No.

PS: Are there such company (PCD) who sells antibiotics?

SH: Yes, have some who make antibiotics but they not market it themselves. They use jobless youngs.

PS: What do those jobless boys do? They take those products to you?

SH: Yes.

PS: So they are not like representatives?

SH: Almost come in similar way/category. They are doing business on that. There are no rules and regulations on them. They can sell anywhere. There is no systematic way. Just if you have license it's fine.

PS: Which license?

SH: drug license for sell.

MH: Do these PCD companies offer you any type of incentives?

PS to MH: There is a margin.

PS: Besides this margin profit on medicine, do they offer you any incentive or gift?

SH: Yes incentive/gifts, what to say.

PS/IS: What type of incentives it is? And how many times in year they give?

SH: company gives I don't know.

PS: You get it right?

SH: what to say about that. Few companies give 5%, few give 10%; some says I will give you umbrella and some says I will give you car.

PS: Is there any difference between large and other companies? Or all talk like this?

SH: these are market policies, what will I discuss with you? If you make a company and how will you market it, it depends on you. It's not matter of us. I am only concern with what I will make my bread. It is clear thing. Doctor is with us now. Isn't it?

IS: So it varies actually between companies or wholesaler. There is trade secret he doesn't want share if their % is less.

MH: What types of customers comes here most?

PS: Of cows. Previously it was poultry customers but now it is gone out of business.

SH: Here govt. polices are wrong that's why poultry customer gone out.

PS: What is that?

SH: In other places where people are rearing up to2000 to 5000 birds, but here people are getting 50 or 10. So what will he do with the 10 birds? Will he eat those or will he rear them?

IS: That is government distribution program.

SH: I have seen it as these are given for their eating, are they giving them for doing business?

IS: That is through self help group.

SH: Self help group will not give you 2000birds you want to take from them. Previously bank gives loan for poultry but now they are not giving.

IS: Yes, one thing you are saying is right.

SH: Yes, from block they giving just 10 birds, are the giving for rearing/improvement of poultry industry? Are they giving for their feeding purpose or for taking votes in election? If in that way govt. not take any policy, poultry farming will never be possible here.

MH: Does anyone come to you to buy antibiotic who are not farmers?

SH: Farmers, pranibondhu, quacks also.

PS: How do you differentiate pranibondhu and quacks?

SH: Quacks means they did not get certificate. Doctor can say this. Govt. has given a paper to pranibondhus but not to quacks who are trying to treat in their own.

MH: How many pranibondhu do you deal with regularly?

PS: How many pranibondhu come to you in a month?

SH: All the pranibondhus of the block.

MH: Can you estimate it?

SH: in each G.P one pranibondhu is there. Block knows total how much pranibondhu it has. In one block 8-10 numbers are there.

PS: And quack doctors? (who comes to you to take medicine)

SH: almost double of pranibondhu.

MH: Are there any private vets come to buy antibiotic here?

SH: if needed they take.

PS: That with prescriptions or they come to take medicine here?

SH: Both. But most of the time they give prescriptions.

PS: You are talking about govt. doctors?

SH: Govt. doctor mostly prescribe.

PS: Are there private doctors also?

SH: No. Some were from milk project. Previously was but after coming this govt. they are not present. (murmurs something)

MH: who are the biggest customers among these farmer, pranibondhu, quacks and vets?

SH: Mostly farmer.

MH: Can you say what proportion of antibiotics you sell to farmers among all antibiotic selling?

PS: Who asked it before, he couldn’t answer.

PS: So among these who take more antibiotic?

SH: Everyone takes when they need. Previously antibiotic was needed more in poultry but it gone away. In cows fewer antibiotics is needed. Previously in poultry antibiotic was used mostly. Now if people’s cow gets sick they come to buy, otherwise they not come. So how can I say it.

PS: Ok. Do the pranibondhus take more antibiotic than farmers?

SH: One farmer has 30-40 cows, and such pranibondhu also are there who visit 2 cows.

(Dairy farmers/khatal farmers take more antibiotics)

PS: Here the farmers means farmer of ‘khatal’?

SH: Yes, assume it as khatal. Here are very few cows (local farmers) are present. It is not Bihar (another state) where everybody will keep a cow, it is West Bengal. The peoples are lazy, they not rear themselves more, do it by others, in khatal. People will buy milk from khatal but will not rear themselves.

MH: What do you do with expired antibiotics?

SH: Company takes it back. It doesn’t expire mostly.

MH: What do you think about good or bad quality antibiotics?

SH: Doctor can say this. We just buy and sell.

PS: No, in way of selling.

SH: we just take money and sell it, they know, go to their khatal, they can say which one is good or bad. To us all are good. When govt. is allowing it in market, all are good to us. They must be releasing it after testing it. So why will it be bad. Many say that generic products not work. We never say that generic product not works. Previously antibiotic was in combination, now it may be in separate, in generic form. This is the difference.

Some people are telling generic product is bad (rumor). I never thought that generic is bad. We see that sitting here. Large company also are releasing generic product. In human all are giving in generic form, so why it is bad? The same thing when sold in brand it cost increases. Govt. wants that all can get the medicine but some people thinks that what profit they will get by it.

I want that there should be more generic product in the market. Why people will spend more money on that. Suppose if you buy paracetamol of company it is of 2 rupees but if you take it in generic form it is 50paise. So 1.5rupees is getting wasted. Is there any difference?

IS: No.

PS: Same story with veterinary medicine also?

SH: same. But I stand with generic products. And some shops also tell I sell company products not generic. Why they do this I don't know.

MH: Do you have any feed supplement which is having antibiotic in them? Are there any antibiotic that can be added with feed or water of animals?

SH: no antibiotic are given in feed.

But when they use it as feed supplement antibiotic has one function there. (Inaudible). But it is rarely used.

PS: Has it been used for cows or poultry?

SH: For all. Sometimes in rainy season feed toxicity comes; when the feed gets fungus, antibiotics are given with them otherwise it will harm cows or poultry who take it.

IS: Does it keep the feed in good condition or the poultry in good condition?

SH: the poultry. It destroy the fungus. In godown (factory) the feeds are made with different raw ingredients. So any one can have contamination.

IS: So who gets benefit in that by giving antibiotics?

SH: Now if in rainy season diarrhea occurs, then they have to give something to prevent it. But it's very less. If there is any chance of contamination like if our feed gets fungus in freeze in 2days we either throw it away or fry it again. Same thing.

MH: What type of antibiotic do they use for that?

SH: they not use costly antibiotic. Use some tetracycline group.

MH: Do you think that the ways antibiotic are being used are needed to be changed?

IS: Do you know that this extra use of antibiotic is going to dangerous for human?

SH: It’s bad.

IS: How?

SH: If you take antibiotic lameness occur, getting thinner.

IS: Do you know that over use of antibiotic leads to resistance?

SH: yes, but what will I do knowing that.

IS: According to you what should be done to prevent it?

SH: but if cows get fever if I not use oxytetracycline, will that fever get down? So I have to use for again whenever is needed. Because I have to save (animal). Is there any way that without giving antibiotic fever can be reduced? Or is there any way that Govt. of India didn’t circulate among us.

MH: Thank you very much.
