# Supplementary material for: “If It Works in People, Why Not Animals?”: A Qualitative Investigation of Antibiotic Use in Smallholder Livestock Settings in Rural West Bengal, India
Source: Antibiotics (Basel). 2021 Nov 23;10(12):1433. doi: 10.3390/antibiotics10121433 (PMC8698124; doi:10.3390/antibiotics10121433)
Supplement: Supplementary file 1 [file antibiotics-10-01433-s001.zip › Supplementary S1_ Interview Transcripts/Site 2/Human Drug Shop 2 (site 2).pdf]

Code for Study - ‘If it works in people, why not animals?’: A qualitative investigation of antibiotic use in smallholder livestock settings in rural West Bengal, India: Human Drug Shop 2, site 2

Interview Date: 1/15/2020

Interviewee: Human Drug Shop- supplying antibiotics to livestock keepers in site 2- Antibiotic Provider

Interviewer: Mat Hennessey (MH), supported by Soumen Samanta (SS) and Pabak Sarkar

Transcript prepared by: Soumen Samanta (SS)

MH- Mat Hennessey

S- Soumen Samanta

P- Pabak Sarkar

All answer (A) by SH

XXXX = Name of the shop

P: Do you keep both human and veterinary medicine?

A: Yes.

P: Do you keep antibiotics also?

A: Yes, yes.

P: Tell me some name of those. First human then animal.

A: For human: levofloxacin, azithromycin, cefuroxime, cefixime, ofloxacin;

very few brands run in this market.

For Animals: Ofloxacin+ornidazole(For human also this combination available but different formulation is there, human formulation is not used in veterinary medicine), amoxicillin+clavulanic acid, cefixime+sulbactum, Moxifloxacin, ceftriaxone, oxytetracycline. [these he mentions later 12:00min]

P: Besides these, do you keep other brands?

A: Yes, 1-2 packets of other companies. I have told the major brands of my shop.

P: Do keep same medicine of other brands?

A: Yes but very less.

P: Can you tell me some of those?

A: Like levofloxacin of [brand name redacted] and [brand name redacted] ; ofloxain of [brand name redacted]

P: Where these medicines come from?

A: Comes from [name of local market redacted.

P: How those come? Any specific shops?

A: No specific, from here and there.

P: How do you decide from which shop to take?

A: All products are not available all time (From a single shop). So we have to buy from [name of local market redacted]

P: Then?

A: Kuli(porter) are there who pack and transport it to [nearby town name redacted].

SS: It's all in your cost?

A: Yes, all at our cost.

P: So first you have to go [name of local market name redacted], then you have to buy medicines, then you have to take kuli (porter), then he sends it by transport.

A: Then transport gives it to boat.

P: [name of village redacted]?

A: No, [name of village redacted].

P: Then? From [name of village redacted] to here.

A: From [name of village redacted] it comes to [name of nearby town redacted], from there kuli pick up and supply it to our shop.

P: How many times does this happen in a month?

A: 4 times in a month, weekly once.

P: Which date?

A: No fixed date is there.

SS: Do you yourself go there?

A: Me, my brother.

P: Besides this, is there any other way from where you buy? Many times medical representatives.

A: No. We have GST (tax). We don’t take that way.

P: Does the PD company give you medicines?

A: Yes.

P: Do they supply antibiotics also?

A: Yes.

P: Which antibiotics do they supply?

A: These medicines that I have told before. Doctors don’t prescribe much variety.

P: How much antibiotics do you buy from [name of local market redacted] and how much from PD company?

A: 90-95% from [name of local market redacted] and 5% from PD. PDs medicine don’t run much.

P: Can you say why? I am talking about PD’s antibiotics.

A: If doctor prescribe more then only it can run. The made the doctor to write by visiting.

P: So by only these 2 way medicine comes. No other way is there.

A: No.

P: Who take antibiotics from your shop?

A: Patients.

P: Patients comes and ask this medicine..

A: They come with prescriptions and we serve it.

SS: Did it happen that they forgot or unable to take prescriptions for buying medicine.

A: Rantac (Ranitidine), histac like medicine are sold but antibiotic without prescription is not sold here. Antibiotics are mostly scheduled 1drug.

SS: It can't be sold?

A: No, not that. It is restricted.

P: Besides patients, does any shop buy from your shop?

A: No, it is retail counter.

P: Here are some village practitioners, do they buy medicine from you?

A: Yes, but less.

We are also having a wholesale shop in the 1st floor, from that shop we send medicine to counter.

P: Counter means? These retail shops here?

A: Not here. Other retail counters of different island. This one is retail shop. There is no link.

P: no, no. But your wholesale has another licence. From that shop

A: Wholesale occurs to different island shops.

P: Is it having different names?

A: Yes.

P: Can you tell me that name?

A: [name of distributor redacted].

Shop owner (A): Why are you actually taking these data? On antibiotics.

P: Because we are seeing that after using antibiotics also, some are not working. If it continues then a life threatening condition may appear. So we are trying to understand why it's not working. That is our main work.

P: So from [name of distributor redacted] distributor, it goes to retails shop or village practitioners.

A: We give it to shops. Not others.

P: Okay.

P: So from here most medicines go to patients.

A: Yes.

P: How much is that? 90-95%?

A: which one?

P: That go to the patients. From XXXX retail counter, patients are taking 1005 or 90-95%? Antibiotics.

A: Sold to the patients with prescription only.

SS: No other takes antibiotics?

A: We don't give. If they write in a white paper, we don't serve them.

P: In case of veterinary antibiotics?

A: Most antibiotics are prescribed here from [name of site 2 block redacted] Block Aminal Hospital.

SS: What type of antibiotics they use?

(A customer comes without prescription and asks for a pain medication, then shop staff advise her to visit a doctor)

A: amoxicillin + potassium clavulanate injection, cefixime+sulbactam.

P: The human patients who come, where those prescriptions come from?

A: [name of site 2 block redacted] hospital and also some doctor has chamber here also.

P: So they do prescriptions and their patients comes here to take the medicine.

P: From hospital the patients come or only from those private doctors only/

A: From hospital also come.

SS: Does they prescribe generic name?

A: Most of the time brand name is written. Also write some composition.

P: For animal you told it's from block hospital, in case of human from where?

A: Hospital and those doctors who are doing chambers.

SS: If total 10 prescriptions come here, how much come from hospital and how much from these chamber?

A: From hospital it is 8 and from them it is 2.

P: Do you have experience that people have taken medicines from you but they reported that it is not working?

A: We don't sell lose antibiotic, very less we sell, what doctor prescribes we sell it.

P: Do you tell the people that 'antibiotic dose should be completed'?

A: Yes. We don't sell without 'full course' antibiotic.

P: Why?

A: Antibiotic resistance will happen.

P: Can you tell me about 'antibiotic resistance'?

A: As far I know if you don't complete antibiotic course, next time it will not work. You have to give high dose then. If that also not completed, then more high dose antibiotic to be given. Then one time no antibiotic will be available to give.

Sometimes peoples are not able to take full course antibiotic due to lack of money. Then we have nothing to do. Suppose 10 numbers is written then we have to release them giving 5. We told them that another 5 are due, you should take it, don't stop a day. We write them that 5 is due.

P: Where do the patients come from?

A: [names of three GPs outside of site 2], from all the 14G.P. the people comes here.

P: Do you have seen any antibiotic that was previously prescribed much but now it has reduced?

A: One antibiotic cure one type of disease. Thus doctor prescribes. Particularly it can't be told. I am not long in this line.

P: How long are you in this line?

A: 7-8 years.

P: That's not less. (laughs).

SS: If total 10 prescriptions come, in how much the antibiotic is prescribed?

A: 3-4 prescriptions/10 prescriptions.

SS: If your daily total sale is 100rupees, how much percent come from antibiotic selling?

A: Less. Suppose 5%.

P: So which one is more?

A: Normal. Now market is poor.

P: And how much is that for veterinary antibiotics?

A: 5 or less than 5%.

P/SS: Did it happen that people came for veterinary medicine but due to non availability they took human antibiotics?

A: No. In my shop no alternate medicine is given. What brand is written there if it is not here, we don’t give medicine at all.

P: What do you do with the expiry antibiotics?

A: We keep the medicine separately 2months before their expiry date. Our medicine is returned.

P: Can you tell me the return process?

A: If we give the expiry medicine back, they give us computerized expiry bill.

SS: Do they take back at the same price?

A: No, at a little reduce price.

SS: How much?

A: Not exactly how much I didn’t calculate. After GST it’s complicated.

A: Where does he come from?

P: He came from London, Royal veterinary college.

MH: Could you show me the animal antibiotic he has?

A: Moxifloxacin. And shows some.

Mat ask some previous questions to pabak which already been asked.

Both veterinary and human drugs are supplied from the wholesale counter to retail counters of different islands.

MH: Do the pranibondhu, pranimitra buy medicine from here?

A: Yes.

MH: Do they buy from wholesale or retail shop?

A: Retail.

MH: What type of antibiotics do they buy?

A: Generally don't take antibiotics.

MH: What type of medicine do they buy?

A: Calcium, iron tonic and digestive preparation, anti-bloat medicine, multivitamins.

MH: When pranibondhu and pranimitra buy medicine, do they buy on credit or by cash?

A: Always cash.

P: When you buy from [name of local market redacted], is that is also on cash?

A: Both cash and credit.

P: Is that something like rolling credit?

A: Yes.

P: Is that you have to clear in a year?

A: Yes, within a year.

P: Do you have to sell on credit anytime?

A: Yes.

P: How they pay it?

A: After the 1st due payment they take the 2nd credit.

P: So there is no long term due.

A: We do business in a small scale.

MH: Why do you not sell to the paravet/quacks?

A: We have GST, so it is problematic to give them bill.

MH: Does it not problematic with pranibondhu/pranimitra?

A: They take it from retail at a discount.

P: To whom you are giving discount?

A: To all. We give 12%discount.to some 15 also.

P: When you buy from [name of local market redacted], at what price do you buy that?

A: All over 25%discount.

SS: What about antibiotic discount?

A: Same type.

P: Do you have to keep the generic version of antibiotic also?

A: Yes.

P: Which one more?

A: What we sell from retail counter that is totally branded. If composition is also written in the prescription, it is not served.

P: What do you think to do for proper use of antibiotic?

A: Need awareness camp.

P: What type of awareness?

A: The shopkeeper should know this is antibiotic, who comes with prescription he/she also know whether antibiotic is present or not, shopkeeper should also tell to complete the course. If he/she don't take full course medicine he/she should be awarded to take the due medicines.

I follow it when I am present, also told others to tell it.

SS: Who run the shop in your absence?

A: Brother and these staffs. We strictly maintain this in our shop.

P: Does it happen that if you reach a target sale, company gives you incentive?

A: No.

P: Do the MR comes here?

A: Yes. In the wholesale shop some direct company comes.

P: Which company?

A: [names of three pharmaceutical companies redacted]. These are veterinary. As it is direct they have to come.

SS: Do they come and say dada keep these medicine?

A: Instead of us they go the centres. They visit to pranibondhu and pranimitra. They come once in a month.

SS: Do they visit block doctor also?

A: Yes.

P: Among human medicine which company visits here?

A: Very less. Like [names of two pharmaceutical companies redacted].

SS: You said they visit doctors and pranibondhu, pranimitra also, do you get pranibomdhu's prescription also?

A: No, they don't have prescription. They all get from the centres.

MH: In how much shop, the wholesale shop supply?

A: 10-12 counters.

P: Which area?

A: [names of three GPs outside site 2 redacted].

P: Is there any shop in [name of area in site 2 redacted] area?

A: No.

MH: How do you convince those shops to buy from him instead of other wholesale shops?

P: Do those shops also take medicines from others besides you?

A: Yes.

P: Why they do take you? What are the reasons?

A: Good relation. I give at cheap rate.

P: At what rate? (Discount)

A: I sell them keeping 3%rate for me.

P: Do the wholesale medicines also come from [name of local market redacted]?

A: Human medicine from [name of local market redacted]. Most veterinary medicines come directly from Companies and some from [name of local market redacted] wholesale shop also.

Q: Do the other shop prefer your wholesale shop than other wholesale shop?

A: It is very difficult to say. I don't know who take from whom.

P: Thank you.
