# Supplementary material for: “If It Works in People, Why Not Animals?”: A Qualitative Investigation of Antibiotic Use in Smallholder Livestock Settings in Rural West Bengal, India
Source: Antibiotics (Basel). 2021 Nov 23;10(12):1433. doi: 10.3390/antibiotics10121433 (PMC8698124; doi:10.3390/antibiotics10121433)
Supplement: Supplementary file 1 [file antibiotics-10-01433-s001.zip › Supplementary S1_ Interview Transcripts/Site 2/Informal Provider 6 (site 2).pdf]

Code for Study - ‘If it works in people, why not animals?’: A qualitative investigation of antibiotic use in smallholder livestock settings in rural West Bengal, India: IP6, Site 2
Date: 13/01/2019
Location: Site 2
Interviewee: Informal Provider of Human Health (IP)- Antibiotic Provider
Interviewer: Pabak Sarkar (PS)

PS: Pabak Sarkar (PS)
IPR3: Informal Provider (IP)

[START of RECORDING]

PS: Can you please describe us what types of patients generally come to you? What areas they come from?
IPR3: By area, people from neighbourhood come to me and some people from outside areas also come to me.
PS: What do you mean by outside?
IPR3: [name of area outside of site 2 redacted]
AB: From the other side?
IPR3: Yes from the other side of the river.
PS: And which areas from nearby neighbourhood do people come from?
IPR3: [3 areas outside of site 2 redacted].
AB: People from [name of area redacted] also come to you?
IPR3: Yes.
(People mumbling. Attending patient)
PS: So for the service you provide, what kind of antibiotics do you need to use?
IPR3: Many different types of antibiotics are used. There are many antibiotics and they are used. Do you need the names?
PS: If you can tell the names of a few that you use.
IPR3: Ok. Azithral 500 mg, 250 mg, Cifran CT, Cifran 500 mg, 250 mg.
AB: Both Cifran CT and Cifran 500?
IPR3: Not Cifran CT, it costs more. Antibiotiotic – ofloxin, tinidazole.. That is used for other purpose... these I keep.
PS: For what diseases do you generally use antibiotics? Like Azithral...
IPR3: For fever, cold cough. If there are some cuts or injury, then also, it is given.
PS: And cifran?
IPR3: Cifran is given if there are cuts and injuries. Given for fever too.
PS: And you were telling about Ofloxacin.
IPR3: OF tablet is used for fever or cough and cold
PS: This one too is for fever- cough and cold?
IPR3: Yes.
PS: And Tinidazole?

IPR3: That is for loose motion, dysentery antibiotic and this is given. Ciprofloxacin Tinidazole. Cifran CT. And there is Zocef.
PS: Ok. What is this for?
IPR3: This is for cuts and injuries. Like when the injury is massive. It is being given.
What type of diseases do people come with so that you have to give these?
IPR3: Here they come with fever, cough and cold. Loose motion, nausea, stomach ache. Then diarrhoea. For diarrheal cases I give saline
PS: Can you please show me the medicines that you mentioned?
[Shows Medicine]
IPR3: This is Azithral.
PS: Which company?
AB: [company name redacted].
IPR3: This is Cifran tablet. The name of the brand has been cut.
PS: Ok. You can keep them back. So, these medicines do you keep of same company or of different companies?
IPR3: These are from different companies.
PS: For azithral, do you keep it of different companies?
IPR3: I keep it of only one company.
PS: Where do you get these medicines from?
IPR3: I take it from the shops.
AB: in [nearby town name redacted]?
IPR3: There is Pharmacy shop at [nearby town name redacted]. I take medicine from there. [name of shop redacted].
PS: Where else?
IPR3: [nearby town name redacted]. I bring them from the shops in [nearby town name redacted] too.
PS: Which Shops in [nearby town name redacted]?
IPR3: [name redacted], [name redacted].
Ps: Does it happen that people bring medicine to you?
IPR3: Yes. The representatives come. Sometimes they leave medicine. The people from [name of drug supplier redacted] company deliver the medicine.
PS: Do they have antibiotics?
IPR3: They supply all types of medicines in general.
PS: Other than these, are there any supplier you use?
IPR3: No. These people supply. But I get most medicine from the shops.
PS: Which shop do you buy most medicines from?
IPR3: From the shop. [name of drug supplier redacted].
PS: so do you get most of the medicine from them?
IPR3: I get all types of medicine from them. As it is nearby, I get it from them.
PS: Are there any reason to go to [name of drug supplier redacted]?
IPR3: No.
PS: Are there any discounts available from the medicine that [name of drug supplier redacted] or [name of drug supplier redacted] supplies?
IPR3: Yes. There are some discounts available.

PS: How much?
IPR3: they give 10% discount more or less.
Ps: All of them?
IPR3: yes. All of them give discounts.
PS: [name of drug supplier redacted] too?
IPR3: yes.
PS: Do they supply medicine themselves?
IPR3: The personally come and deliver the medicine. We too get medicine from [nearby town name redacted]. And it is available in their shops too. When it is not available with them we have to get from the shops.
PS: Can you explain who are they?
IPR3: [name redacted] of [name of drug supplier redacted] In their stores, all representatives come. Most of them send medicine there.
PS.. AB": in addition, they come to you and deliver medicine at your doorstep?
IPR3: yes.
PS: Can you buy medicine with credit here?
IPR3: yes. From [name of drug supplier redacted].
Ps: And from MR?
IPR3: They give some discount on their commission.
Ps: Is there any credit system for them?
IPR3: The ones who come here take cash and when we go to the shop we get it with credit.
PS: And that is true for [nearby town name redacted]'s shops too?
IPR3: Yes
PS: People who come to you, do you have to give any discounts?
IPR3: By discount, sometimes have to give some. Sometime have to give for free too.
PS: In what circumstances do you have to do that?
IPR3: Those who cannot pay at that time. Also, I have to give credit in some cases. Took the medicine, did not pay. The amount stays due. It's a big problem in these rural areas.
PS: Roughly how many people get credit from you?
IPR3: Give and take 150 people.
PS: And are there people who pay full?
IPR3: The ones who take credit, sometimes pay full, and sometimes take on credit.
PS: And are there someone who pays in full?
IPR3: There are.
PS: So, what is the difference in them?
IPR3: This is a poor area. Once they have an income, then only they can pay. Some times there are dues that they cannot pay at the time.
PS: So, even unknown person can get treatment on credit from you?
IPR3: No. Why should people unknown to me take credit? They don't take any credit like that.
PS: There might be situation when the case is tough. How do you manage those?
IPR3: When I get tough or problematic cases, I refer them to [nearby town name redacted] Hospital.
PS: Do you consult any doctor?

IPR3: Yes.
PS: How do you do that?
IPR3: I call them over phone?
PS: Which Doctors do you consult?
IPR3: I call [name redacted]. There are others there. [name redacted]. Them and other doctors. One of my in-laws is a doctor. I even take is advice.
PS: Do you have any association for the village doctors?
IPR3: Yes.
PS: Can you please tell something about that?
IPR3: We have an association. There is [name redacted] –a dentist – in [nearby town name redacted]. All doctors are in it.
PS: What activities are undertaken there?
IPR3: We have monthly meetings.
PS: On what topics?
IPR3: Regarding doctor's stuff. So, that we don't get in to any problem. Then we get training from [nearby town name redacted] Hospital. BMOH has given us training. Sometimes we need to attend classes there. This time a lot of them have gone to [town name outside of site 2 redacted Hospital. The hospital here, had sent them.
PS: Have you gone there?
IPR3: Yes.
PS: What do they do in [town name outside of site 2 redacted]?
IPR3: They too are giving training. How to and not to treat and other things. I have just joined. A batch of 50 had completed. A new batch of 50 just started.
PS: How is it orgainzed?
IPR3: It is a 6 month training. And our doctor [name redacted] has organized a one year course. All doctors are teaching there.
PS: Where is it conducted?
IPR3: It is in [town name outside of site 2 redacted].
PS: Have you done that?
IPR3: Yes. I have taken the course. That is primary healthcare provider's course.
PS: So, in these courses you have done, have they talked about the antibiotics?
IPR3: Yes.
PS: Can you give us a description regarding that?
IPR3: They say you should not give antibiotics in haste. Prescribing that, would not be right. When in need, if there is no other way, then only it should be given.
PS: Do they inform about what antibiotics do in our body?
IPR3: It clears and strengthens capacity to prevent diseases. It should not be given for 1-2 days. It should be for 5 days, 3 days or a week.
PS: Do they tell which should be for 5 days and which ones for 7 days?
IPR3: No, not that in details. The general things. We had a doctor here – [name redacted] – might have heard his name. He used to treat for free at [name of dispensary redacted] dispensary. He was there for a long time, just passed away. I was with him.
PS: How long were you there?
IPR3: I was there for 17-18 years.

PS: What did you use to do there? How did you help him?
IPR3: Doctor used to sit and tell us, " remove the boils", for someone with boils. For someone needing teeth removal, we used to do that. Give injection, saline... things like that.
PS: You used to do that?
IPR3: Yes. He would instruct and we would do that.
PS: How many more like you used to be there?
IPR3: We were 6-7 people.
PS: So are they all working like this?
IPR3: No. Some of them have left. Like [name redacted] of [nearby town name redacted], me and there was another one [name redacted], his house is near [nearby town name redacted] Police station.
PS: Are they still practicing?
IPR3: Yes. They are. Then there was [name redacted]. Chamber in [nearby town name redacted] Bazar.
PS: So, when your patients come, what is the main thing they demand?
IPR3: We cannot demand and tell that they have to give 30 or 50 rupees. They don't want to pay like that. So, it has to be based on the price of the medicine.
PS: So, what do patients look for? What is their goal?
IPR3: To be treated with the least cost possible.
PS: In that scenario, how do you apply your knowledge of completing antibiotic course?
IPR3: That cannot be applied here. They say, " give me medicine for 2 or 3 days." I tell them, " it does not work like that." I tell them," in other places they will give you medicine for 5 days or a week. Two days medicine will not work. They say, " Don't have the money. Please give." That is a big problem. The course is not maintained.

PS: In your opinion, what should be done to make them complete the course?
IPR3: You have to give credit.
PS: But you already give credit. Does that help in course completion?
IPR3: They say, "I will come later and take the rest." But, does not come back if he/she feels relieved. That creates the situation. Sometimes they come back.
PS: Do you tell them what can happen if they do not complete the dose?
IPR3: Yes, I do. I tell, "The disease stays with you if you don't complete the course. The microbe will stay in your body and you do not get cured. " They say, " okay. If you can, give it to me. I will come back in two days." That is the situation. This creates the problem.

PS: So, do you get all the antibiotics that you need?
IPR3: more or less I get everything.
PS: Are there any antibiotics, which you think will work better, but it's not available?
IPR3: No, nothing like that. But if there is something, it will not be a problem.
PS: So, how do you get information about the new antibiotics?
IPR3: Representatives from various companies come. They talk about the new ones.
PS: You talked about [name redacted]. What other representatives visit you?
IPR3: Many companies come. They used to come more frequently previously. Not anymore.
PS: Why?

IPR3: It has gone down now a days. Now they only visit the shops. They don't come to us now a days.
PS: Then, do you have trouble getting information regarding new antibiotics?
IPR3: It is inconvenient. Five years back they used to come frequently.
PS: So, do you get information from [drug shop name redacted]?
IPR3: Yes. He tells us, "This new thing has come. They have given it to me. Take it." I bring it then.
PS: Have you got some new antibiotic from him recently?
IPR3: No.
PS: So, among the antibiotics that you have, which one do you need to use the most?
IPR3: Cfran, Ciprovin, then azithral, OF tablet.
PS: Which one is the most expensive?
IPR3: Zocef tablet is the most expensive.
PS: So, in what situation do you have to use Zocef?
IPR3: In severe cases. Then, a lot of times I write (prescribe) medicine.
PS: Where do the patients go for medicine when you write it down?
IPR3: They go to the medicine shop. We have only one medicine shop here.
AB: In which cases do you write?
IPR3: For severe cases. Sometimes I do not keep those medicines with me. Then I just write it down for them to get the medicine.
PS: If any medicine expires, what do you need to do?
IPR3: I dispose it.
PS: Don't they take it back?
IPR3: I don't know if they take it back I just dump it.
PS: Like here?
IPR3: In the river.
PS: And where do you store the medicine.
IPR3: Here. (in the clinic).
PS: As people come to you for their treatment, do they come for treatment of their cattle too?
IPR3: (chuckles) Yes they do.
PS: In what type of cases?
IPR3: The cow might have loose motion, or has acidity or fever. They say, " Give something." I give some (medicine)
PS: What medicine do you give generally?
IPR3:Antibiotic, analgesic medicine. And for acidity I give Magsulf or Divol. Antacids, like that.
AB: The one you give for humans?
IPR3: A bit higher power than what I give for human. The medicine is the same. Only the doses are different.
PS: For chicken too??
IPR3: Yes. I keep some veterinary medicine too.
PS: Where do you get them from?
IPR3: From the medicine shop.

PS: Dey also keep veterinary medicines?
IPR3: No he does not. In [nearby town name redacted], they keep.
PS: Who in [nearby town name redacted] Keeps?
IPR3: [name redacted].
PS: What is the name of the shop?
IPR3: (could not remember)
PS: What type of Veterinary medicine do you generally bring from him?
IPR3: Just one type of medicine. For loose motion.
PS: Have you noticed that sometimes people take medicine for their problem, but give the same medicine to their livestock?
IPR3: Most of the times they take advice.
AB: Do they bring (the animals)?
IPR3: Many times they bring. Many times they ask me to visit their home.
PS: Among 10 people, on average how many will do that?
IPR3: Rarely they come to me. There are veterinary places. There is [local NGO name redacted]. Most of them go there. One or two might come here.
PS: Have you noticed cases where the antibiotic that used to work well, is not working?
IPR3: Yes. It is natural that they are not working as well as they used to do.
PS: In which antibiotics did you noticed that?
IPR3: Cifran used to work well before. IT is not that good now.
PS: What do you do in that case?
IPR3: If this antibiotic does not work, other antibiotic is given.
PS: So, what are you giving when Cifran is not working?
IPR3: I give Azithral tablet.
PS: How often do you see the medicine not working?
IPR3: In some cases. Not that frequent.
PS: If you see 10 patients….
IPR3: 1 or 2 might have that problem.
PS: So, there are other village healthcare providers, do you follow their treatment pattern or medicine?
IPR3: Sometimes.
PS: Do you take their advice?
IPR3: Not really.
AB: As you mentioned, Cifran is not working. Why do you think it is happening? It can happen for other antibiotics too.
IPR3: Previously, it used to work extremely well. Now a days there are some faults coming up.
AB: Why do you think it is happening?
IPR3: The reason may be that they are not taking the medicine properly. They might be used to take it regularly before. I prescribe a dose of twice daily, may be they are having just one in a day. Took it one day and skipped next day. There might be problems like that. They don’t want to take the medicine in a proper way. That is another facet
PS: Do you think it might be due to the quality?
IPR3: Though medicine quality changes sometime. But?

AB: In what way (the quality changes)?
IPR3: The colour of the medicine may change.
AB: what is your idea about Antibiotic resistance?
IPR3: No. in general, there is no problem like that. That's what we see.
AB: Have you noticed any trend among the patients to take antibiotic without any reason?
IPR3: No. Nothing without a reason.
AB: Or people involved in healthcare, are they giving antibiotics to people without the need?
IPR3: No.
AB: As you mentioned the villagers are not aware about antibiotic use. What should be done to develop this? And what can be you role in that? And who else might have a role in this.
IPR3: We need to explain and tell them it is not done if this (antibiotic) is not used in a particular way. We have tell and explain.
PS: But you guys are already doing it.
IPR3: And the people teaching us are also telling that we need to counsel. And not use antibiotics indiscriminately.
AB: We are trying to device a way including everyone involved in healthcare delivery system. We have not decided the mode. But if a training is arranged or a meeting is arranged, will you attend it?
IPR3: Yes. It should be very good if it is done. It should be arranged in the main place, like [nearby town name redacted]. So that others (can join)
AB: But how can we make this initiative effective?
IPR3: In [nearby town name redacted] Bazar, above [temple name redacted] temple there is a place. Pankaj Babu, he is the secretary.
AB: What is he secretary of?
IPR3: Bazar Committee.
AB: That is for the venue.
IPR3: yes.
AB: How should the work be done so that people are benefitted?
IPR3: We need to have conversation by inviting all the doctors (RHCP included).
AB: Ok then.

[END of INTERVIEW]
