# Supplementary material for: “If It Works in People, Why Not Animals?”: A Qualitative Investigation of Antibiotic Use in Smallholder Livestock Settings in Rural West Bengal, India
Source: Antibiotics (Basel). 2021 Nov 23;10(12):1433. doi: 10.3390/antibiotics10121433 (PMC8698124; doi:10.3390/antibiotics10121433)
Supplement: Supplementary file 1 [file antibiotics-10-01433-s001.zip › Supplementary S1_ Interview Transcripts/Site 2/Informal Provider 7 (site 2).pdf]

Code for Study - 'If it works in people, why not animals?': A qualitative investigation of antibiotic use in smallholder livestock settings in rural West Bengal, India: IP7, Site 2
Date: 13/01/2019
Location: Site 2
Interviewee: Informal Provider of Human Health (IP)- Antibiotic Provider
Interviewer: Pabak Sarkar (PS) and Arindam Banerjee (AB)

PS: Pabak Sarkar (PS)
AB: Arindam Banerjee (AB)
IPR6: Informal Provider (IP)

[START of RECORDING]
PS: This medicines that you are dispensing how do you get that help from? Where did you take them from? How difficult it is? In what ways you receive? We are here to understand that.
Can you tell us a bit what kind of medicine do you need to use for primary health service?
IPR6: We buy medicine from [nearest town name redacted] area.
PS: Before that please tell us that what type of diseases you have to treat in this area?
Generally what type of illnesses people come with?
IPR6: As the hospital is very close by, people generally come to us if there is a big trouble at late night, (other than that) for common cold cough and fever and for stomach aches or stomach troubles. Hospital has instructed us that we cannot see drowning or poisoning cases and we don't see that too. Or electrocuted.
AB: What do you do in these cases then?
IPR6: We immediately tell them to go to the hospital if they come to us.
AB: [nearest town name redacted]?
IPR6: Yes.
AB: They will die on the road.
IPR6: We cannot do anything even if they die. Our hands are tied.
PS: So, those who come with cough and cold, fever or upset stomach, how do you need to treat them generally?
IPR6: We need to treat them only by giving medicine.
PS: What type of medicine do you give them?
IPR6: For example….. I need to tell the medicines I give. Right?
PS: Yes.
IPR6: Paracetamol, Calvam (Clavam: Co-amoxyclav from Alkem). Then , taxim – O (Cefexime from Alkem), Cefeximine, Cifran. These tablets….
PS: You mostly treat using these (medicine).
A phone call interrupts the conversation
PS: For which treatment these medicines are used?
IPR6: For fever and related ailments.
PS: And for upset stomach? What do you use for upset stomach??

IPR6: For upset stomach antacid is generally given.
PS: And what else? Does diarrhoea occurs in these areas?
IPR6: Yes. Diarrhea happens in this area.
PS: What medicine do you need to give then?
IPR6: The thing is we received training from the hospital. After training they had said, " In these conditions, don't give saline yourselves", ORS is given. In many case we have to release the patient without giving saline. If there is a situation that needs saline, hospital takes it up.
PS: Is any other medicine, except ORS, given?
IPR6: Yes, like Norflox, Trinidazole, metrogyl. These are been given.
PS: So, these medicines that you keep, where do you need to buy them from?
IPR6: [nearest town name redacted]. Drug house.
AB: Is that the name of the shop?
IPR6: The name of the shop is.. your… that… [name of drug shop redacted].
PS: Is there anyone else?
IPR6: There is [name of drug shop redacted]. There are many. Everyone can pick and choose.
PS: We want to know yours. Do you take from these two? Do you take from anyone else?
IPR6: No.
PS: Do you need to go to them?
IPR6: I go to them and get it.
PS: How many times a week or month do you need to go?
IPR6: See. One thing. The things that you asked it depends on the patients. Because the hospital is very near.
AB: Where?
IPR6: In [nearest town name redacted]. Our patient number is comparatively less. Where there is free treatment available…..
PS: Still you need to keep them
IPR6: I have to go in 1 to 1 and half months.
PS: What do you do if any medicine is finished before 1 to 1 and half month?
IPR6: Then I write that to the party.
PS: Where does the party goes if you write them?
IPR6: From [nearest town name redacted]. Whether it's [nearest town name redacted] or [name of town outside of site 2 redacted] more. They buy it from there.
PS repeats the information.
PS: Do they also take it from that [drug shop name redacted] or other places?
IPR6: There are many medicine shop. Now patient party buys from the place they wish.
PS: So, what is more often? You keep medicine with you or you write medicine.
IPR6: I have write more. I too have some medicine. I write 25 % of medicine.
PS repeats.
PS: So, the places you go in [nearest town name redacted], do you have to get medicine in cash?
IPR6: In cash and in credit too.
PS: Can you explain me the system of how some is in cash and then there is credit too?

IPR6: By credit I mean, they do business according to their capital. Now sometimes they give 12%... upto 18% they can give.
AB: What? Credit or Discount?
IPR6: Discount.
AB: And if on credit?
IPR6: It's similar.
PS: And in how many days you have to repay?
IPR6: Its haalkhata (Local year-ending system). And before that I should say that we have to give it completely on credit. And we have to do haalkhata too.
PS: So, do you have to give haalkhata on households or you have to give credit to everyone?
IPR6: We have specific patients. (We have) their or family's name. So, haalkhata notice goes to their house.
PS: Can you give me an estimate of how many families do halkhata?
IPR6: I have around 100 -125. Then again, these are with me. But I don't stay in the morning. In the morning they will go to someone else.
PS: How many of them are here?
IPR6: There are many like us.
AB: Give us a crude estimate.
PS: Like it is 5-10… or 50?
IPR6: 7-8 will be here for sure.
AB: In Ranipur?
IPR6: Not in [village name redacted]. My home is at [village name redacted], but I mostly sit in this place.
AB: What is this area?
IPR6: This is [village name redacted].
AB: 7-8 in [village name redacted] and in [village name redacted]?
IPR6: There is [name redacted], [name redacted], [name redacted], there is one called [name redacted] and I am here. So, for the time being there are 4 of us. [name redacted] sometimes come here crossing the river from that side.
PS: What side?
IPR6: from [village name redacted] doctor comes.
PS: So, according to you among them or in [village name redacted] area who people go to more? As you have a fixed clientele. Who has more ?
IPR6: [name redacted]. He has gone to [village name outside of site 2 redacted] for a camp.
AB: Whose camp is this?
IPR6: It is … [name of project redacted].. Medical Camp.
AB: Is this some kind of an association?
IPR6: Yes
AB: Are you its member?
IPR6: I was, but now I don't travel that much. I am old now. I can't take the train. I am 65 now.
AB: Do they have an office or who are the responsible people there?

IPR6: It’s in [nearest town name redacted]. Their head is [name redacted]. There is another one, of [name redacted], [name redacted]’s association.
AB: [name redacted]
IPR6: Hmm
AB: That is different.
IPR6: Hmm.
PS: As you give medicine, among them clavam, cifran etc. are antibiotic. What is the percentage of antibiotics that you need to give? Among the medicine that you give how much will be antibiotics?
IPR6: I use less antibiotics.
PS: Why so?
IPR6: Antibiotic is used less because people of this area don’t want to take medicine if 2 tablets end it.. if his fever gets fixed. Doctors said or we say too that this needs to be taken for 5 days. If his stool decreases a bit after taking one norflox. They will say, “ I will not have it anymore.”
PS: Why don’t they listen when you tell them?
IPR6: They don’t listen even if hospital tells them. There are heaps of hospital medicine in some residences.
PS: But why don’t they listen? There are so much medicine available. What’s the harm of 5 day’s medicine?
IPR6: (They say), “ I have been cured. What will taking more medicine do?” That is the tendency.
PS: Do they think 5 days medicine will be inconvenient?
IPR6: It is difficult to get that in to people’s mind. Doctors are giving it. “Have it for 5 days” we don’t take it after 2 days. In each household there are heaps of medicine lying around.

PS: So, why ask for 5 days if the body gets well in 2 days?
IPR6: But the thing is antibiotics have a specific course.
PS: So, what can happen if the course is not maintained?
IPR6: What can happen? Did that microbe die?
PS: But the fever got less. That’s what happened.
IPR6: And hospitals have called and said, “Except paracetamol you cannot give anything.” And actually, the patient numbers have really gone down.
PS: Why so?
IPR6: From the villages around [name of location redacted] it is still difficult to go to the hospital. But from here once you stand here you can directly reach the hospital.
PS/AB: How do they go from here to the hospital?
IPR6: By the road you people took. Or there is a ferryboat. Once you take the ferry, you reach hospital.
PS: What if someone gets sick at night?
IPR6: They come to us in those times.
AB: And what’s that far away village you talked about?
IPR6: [village name redacted].
AB: Is there anyone in [village name redacted]?

IPR6: I told you. [two names redacted]l.
PS: I saw a poster here for [name redacted]. What is his arrangement?
IPR6: I have not seeing him treating.
PS: I have seen the poster. It said he keeps medicine too.
IPR6: He has a room here. But that is Party office (for political party).
PS: The medicines that you take, Clavam, Taxim, Cifran, do you take it of various companies or the one particular company.
IPR6: Mainly, keep these companies. Be it Taxim or Clavam.
PS: But these are manufactured by other companies too. So, do you keep them too?
IPR6: I have these mostly. And I keep it for a reason that other company medicine have less 'effilacy'
PS: What is less?
IPR6: Effilacy (Efficacy), Because of that it is seen that it takes longer time to work. If a few patients have to be kept, it has to be through giving good company's (medicine). Otherwise in the morning they go to the hospital. I don't think the medicine used in the hospital are of good standards. People will take it and wait for 2-3 days. But they want to stay with us by taking 2-3 days of our medicine.
PS: Why is it so?
IPR6: Cause it is given by MBBS doctor.
PS repeats the statement
IPR6: After the night is over, they don't stay with us. If it works at night, then it's ok. Now I cannot run that much. Sometimes I come at dusk, sometimes not.
PS: So, how did you start? When was that?
IPR6: At least that was 30 years back.
PS: What was the system here then?
IPR6: There was no system here at that time. One of my sisters had thalassemia. There was no hospital here. At [town outside of site 2 redacted] there was a hospital. I went to that hospital for my sister's treatment. She cannot be saved from Thalassemia, but my sister was under [name of a formal doctor redacted], Gold Medalist. I used to go continuously to him. Then he used to keep the patient for a month or two at the hospital. We are people from far away. How can we commute, where can we stay? He said himself, "You stay here at this place of mine. When my patients come, make a line etc. From there it started. I got the initial training from him "
PS: There you learned about medicine and what to use when. Did you get any chance of training after that?
IPR6: Yes.
PS: Which ones?
IPR6: I have taken PVOH training from [site 2 name/gp redacted].
PS: What is that?
IPR6: Progressive Voluntary Organisation of Health.
PS: When was that? Around what time?
IPR6: 15 years back. Our President himself came to inaugurate it.
PS: How long was that program?
IPR6: That was 6 months.

PS: Have to go there daily?
IPR6: I used to stay there. Food, lodging etc. was all covered.
(takes a phone call)
From there I got from [nearest town name redacted] Hospital. That was once in a week training. There was [name redacted]. They had given a certificate for the 3 years program.
PS: How many years back was it?
IPR6: That's about 10 years (back).
PS: So, you have received training from time to time.
IPR6: They are giving it now too.
PS: What are they giving?
IPR6: They are giving at Bangur.
PS: Are you taking it?
IPR6: Its going on. But the matter is, I am going every time but they are not ….. me due to over-age. But, the learning doesn't end. They give one biscuit worth 5 rupees everyday at afternoon. Still I go there. Every Saturday. Over there my roll number is 104. But due to over-age, they are saying, "You don't need training. Now you can give us training." But yes. I have hospital certificate. They are not going to give any certificate. They have mentioned it clearly. Your record will be here. As we have a permanent roll number the (record) might stay with them. But we cannot keep in the counter (chamber).

PS: So, many people have trained you. Then you should be very well linked with the the health system. So, in that scenario, do people from company or medical representative come and talks to you?
IPR6: No. They cannot reach up to our area. They don't come this far.
PS: So, how do you get in touch with Medical Representative?
IPR6: We do not get in touch with them. That's why we have treat on the basis of some fixed medicine.

PS: So, when there is a new medicine or Antibiotic in the market, do you get information about that?
IPR6: Yes. When we go to the hospital, then we get some (information) from the doctors. And when anything new comes, doctors try to write that. Those prescriptions come back to us.
PS: So, you broadly follow them…
PS: Do you need to treat animals too?
IPR6: I should tell you now. At night someone says, "the calf is a bit feverish." But doctors to treat animals are available here. I say," Take two paracetamol. In the morning get it checked up by those doctors. Nothing else."

PS: Do they ask for the antibiotics that you generally use for human?
IPR6: No. The department itself is different.
PS: Not even in the case of ducks & chicken?
No audible response

PS: So the antibiotics and medicine you have has an expiry date. What do you do when it crosses the expiry date?
IPR6: We don't want to keep them. At most… let's say Clavam, it's not possible for us to buy more than 5 files. If it's seen that before going to market, those 5 files are finished, then we write it. But it's not true that nothing expires. Those are trashed.
PS: Do the sellers accept returns of expiry medicine?
IPR6: No. I don't know whether they accept back, I have never given back to them.
PS: So, in your long experience have you noticed antibiotics that are not working? Or high doses have to be given?

IPR6: Like sulfa group is not used anymore. I don't do it.
PS: Why?
IPR6: A lot of them get allergy.
PS: What else?
IPR6: Some of them get allergy by taking paracetamol.
PS: But still you need to give paracetamol. But in cases like cifran or norflox, that you stated. Do they work similarly as they used to do before?
IPR6: No. Not always in all the cases.
PS: Can you tell me about that a bit? What changes do you observe now-a- days?
IPR6: It takes time to work. I cannot say that it doesn't work completely. But its seen that action can be felt with 3 tablets than 2.
PS: For which tablets are you seeing this? For which antibiotics are you seeing it?
IPR6: Cifran. Norflox too. Clavam is working well still now.. Roxithromycin.
PS: and?
IPR6: Levofloxacin is not at all used.
PS/ AB: Why?
IPR6: In lot of cases that I know, I didn't get results.
PS: Why do you think this is happening? That you are not getting results from levofloxacin. At one time it used to work right?
IPR6: Used to (work). Now…Or OF – Ofloxacin. It still works well generally.
PS: It still works?
IPR6: In the initial stages it works well.
PS: Why do you think the action has decreased suddenly?
IPR6: (Inaudible) medicine… taking that has increased the strength of the body… for that reason.
PS: So, the work we are doing this time is regarding that the medicine used to work, but they are not working now. Not for all medicine our work is on antibiotics. Here a term is widely used – Antibiotic resistance.
IPR6: Resistance. Sir had taken our class too.
PS: Which sir?
IPR6: [name redacted]. I have his certificate too.
PS: Did he take class on Antibiotic Resistance too?
IPR6: One day's ordinary class. They had come to visit the Sunderbans. I don't remember the year.

AB: Where? In [village name redacted]?
IPR6: No. In [nearest town name redacted] itself.
PS: What is your idea regarding antibiotic resistance.
IPR6: Resistance is occurring. What idea should I give?
AB: If you can share your experience by giving an example? How you are understanding the subject?

IPR6: They have understood the nature of the body. So, they want a bit high level of ….
PS: Who have understood?
IPR6: The strength of the medicine is not enough for our body. Its asking for a bit more. OR they want a different drug.
PS: Is that resistance to you? So, what we are trying to do is we want to improve the awareness regarding antibiotic resistance on a collective level and like you said, " people are keeping (storing) the medicine". If that can be reduced with your help. We want to think about the ways that people use the antibiotic in the right way.
IPR6: In general, with loose motion hospital is giving 14 metrogyl. 14. "Have it for 7 days." After having it 1-2 days, every house is just stacking it.
AB: But, what is the next use of that stacked medicine?
Inaudible response
PS: If they get sick again?
IPR6: They are going to the hospital again.
PS: What is the benefit of stocking the medicine?
IPR6: They are throwing it away. That is not going to be sold and we don't collect them.
PS: These are the behaviours we need to understand. After all medicines are expensive.
IPR6: Try to understand the matter. People in our village are mostly have less understanding of education etc. Town people, People from [nearest town name redacted] understand when they are told that it should be taken for a long period. People here don't try to understand. It's okay if they get instant remedy.
AB: What is happening to the medicine eventually?
IPR6: They just trash it.
PS: But in last few years the roads are made, concrete roads are made. Issues of transport for people has decreased a bit. They are going to study. Then, there knowledge has gone up. Even after then what can be the reason for this attitude?
IPR6: Not in every one. And the Mohamadden [muslim] hamlet here are…
PS: Which way is that?
IPR6: [village name redacted].
PS: Is there someone in [village name redacted] who treat?
IPR6: Yes.
PS: Can you give us one or two names.
IPR6: As I told you. [name redacted].
(Talks about information on other RHCP)
PS: One more question. You are taking medicine from Annapurna in cash or credit. How you maintain accounts of how much you owe?
IPR6: They give computer bill.

PS: But if there is some due, how both of you keep the accounts.
IPR6: Suppose I took medicine worth 2000 rupees and gave the 1500. The 500 rupees I note down in a book or they wrote it on the cash memo. I don't bother how they maintain their books.
PS: But you go there quiet often, so there must be a relationship.
IPR6: They maintain books and documentation.
PS: You too keep such document about what's due?
IPR6: I have the cash memo with me.
[mumble about a location]
AB: As per the present situation of rural health service, what do you think is the biggest problematic area as per the questions we asked. Like the use of antibiotics and medicine or awareness. Where do you think is the biggest gap?
IPR6: Training
AB: Whose?
IPR6: Those who are involved in treating us.
AB: You mean the rural healthcare provider? And?
IPR6: If they get good training, they will be better healthcare provider.
PS: As we heard, the training is going on. How do you define good training?
IPR6: The training is like…. For the past few days, the training was on Anatomy. I don't know what's going to be the next (lesson).
AB: And what should be done to aware people from village?
IPR6: For them, we should sit with them at every village.
AB: Will it work if we sit? Who should we include in these sit-ins?
IPR6: If you come on call of village doctors, then it might work. Another big thing is who will be available after their regular chores. Awareness might not work if only we keep on telling. For your initiative, it might be through ASHA levels and other workers too.

PS: Who, in the household, would be best to train to modify behaviour of all in the family?
IPR6: It would be wrong to say the man of the house, but certainly someone in the house who understands.
AB: But do you think others will listen to the one who understands in the family?
IPR6: He will make them understand. Suppose the leader of the household is the old father. People can ignore his words. But the one who understands, even if he talks about these things while eating rice, people might understand one out of five words that he says. Suppose there are three or four brothers. If it is seen that the third brother understands the gravity…

AB: Ok. That is it.

[END of INTERVIEW]
