# Supplementary material for: “If It Works in People, Why Not Animals?”: A Qualitative Investigation of Antibiotic Use in Smallholder Livestock Settings in Rural West Bengal, India
Source: Antibiotics (Basel). 2021 Nov 23;10(12):1433. doi: 10.3390/antibiotics10121433 (PMC8698124; doi:10.3390/antibiotics10121433)
Supplement: Supplementary file 1 [file antibiotics-10-01433-s001.zip › Supplementary S1_ Interview Transcripts/Site 2/Informal Provider 8 (Site 2).pdf]

Code for Study - ‘If it works in people, why not animals?’: A qualitative investigation of antibiotic use in smallholder livestock settings in rural West Bengal, India: IP8, Site 2

Date: 16/01/2019

Location: Site 2

Interviewee: Informal Provider of Human Health (IP)- Antibiotic Provider

Interviewer: Pabak Sarkar (PS)

PS: Pabak Sarkar (PS) (or in bold text)

Informal Provider responses are not in bold text

PS- The villager usually go to a RHCP if they fall ill and the RHCP must give them medicines for their treatment. In this study we're trying to understand how you treat people, how villagers are getting benefited and from where RHCPs procure medicines, what are the concerns and general benefits for them.

As you are in this health service for around 10 years can you tell me from where people usually come to you are you need to visit them for your treatment?

I usually do the basic treatment of the people. I never go out of my village and my area is like in this gram panchayat only, booth no. 153. [name redacted] usually come here and treat people.

PS- What kind of illnesses you see here?

There are generally 7 to 8 conditions I treat like fever, throat pain, diarrhea, abdominal cramping, cold and cough etc. But if I see a person comes with fever, and it's not getting down for 4-6 days I generally refer to that patient to [nearby town name redacted] Health centre. I don't take risks in these cases.

PS- How you treat these conditions?

like for fever I give paracetamol, in case of diarrhea I give them Norflox TZ. In case of cold & caugh, I treat them with Amoxacillin , Mox, amoxicyline potassium calvum . I noticed that the formal doctors also give their patients same medicines. In case of cough I give them Ascoryl syrup.

PS- You use Mox, Amoxicyline… so these are more antibiotic right? So, what are the other antibiotics you use?

For children, I use Clavam and in case of adults I use Mox, Opex CV, Clavam 625, O2… ofloxacin, Taxim etc. You people know more than me.

PS- No we come here to understand the local problems and how you people are solving it with various kind of medicines.

As i said earlier that I only treat basic and in case of emergency I referred to the patient to goSaba subcenter. we are now going to [town name outside site 2 redacted] hospital for our training it's actually suggested by the [nearby town name] subcenter, they are arranging it.

PS- So from where you get these medicines?

I bring it from [nearby town name redacted] [drug shop name redacted] enterprise.

PS- Where is it?

It is in the [nearby town name redacted] market.

PS- Do you keep these antibiotics like Mox, Clavam, Amoxycyline from same brand or from the others also?

There are lots of companies like Ranbaxy, Sinha, Sarabhai, abott. I keep these medicines so that the villagers get right treatment in affordable price.I charge 10 to ₹20 extra an MRP.

PS- What are the factors you have to keep it in your mind while deciding the medicine brand ?

The stock of medicine mainly depend on the season. like there are people who tend to have fever in a particular season then I have to bring syrup for cold & cough and fever. like in winter, particularly we can see few fungus (chotrak), allergy and all that I just check the composition and take the Medicines accordingly. Like now the season is changing And the temperature is fluctuating so the children tend to have diarrhea at this time so I need to keep metrogyl syrup for diarrhea accordingly.

PS- So do you keep metrogyl from the same brand or from other brands also?

I need to check these as from the medicine shop this suggest me various kinds of brands also we meet various kinds of agents from different brands do they insist us to take an use their medicines, still I don't use randomly cause it can have negative side effects also. Agents from [two town names outside site 2 redacted] also used to come to me but I didn't try it.

PS- Don't they come to you now?

No. I don't take medicines from them.

PS- Do they visit other people here?

No I don't have any idea.

PS- Can you show us the medicines you have ?

yes I can show you some of them .

he showed them few antibiotics….

Actually the fact is I use medicines from good brands, feel it will not be beneficial for the patients. when I refer to the patient to [nearby town name redacted] subcenter I also mentioned about the medicines i used on this particular patient so that they can proceed accordingly.

PS- How many times you need to visit Paul enterprise for procuring the medicines?

Once or twice in a month.

PS- why do you go to [drug shop name redacted] enterprise only because there are lots of others medicine shops also ?

We were in the same college and he gives me the medicine in debt. I give him money in installment. Even when it is not possible for me to go and collect the medicines from their shop still they give the medicine if I send someone with the name of the medicine. Sometimes they call me and tell me we cannot give these medicines to someone without prescription, but I insist them to give one or two files for now.

PS- There are other RHCPS also can you tell me from where they procured medicines?

There is one more RHCP sits here. I don't know from where they bring the medicines from.

PS- What is his name?

[name redacted].

PS- How did you start this health service?

[name redacted] from [village name redacted], he once came to my house for treatment. he inspired me to come into this service. He is my guru. I assisted him for one year then I went to [nursing home name redacted], I learnt from them for 2 years then started my own practice.

PS- Notun Jivan Seva Sadan… what is that?

Nurshing home. Doctors from [town names outside of site 2 redacted] and many more places used to come there. We used to go to them and received trainings from them.

PS- What did they teach?

We used to ask them about different conditions how that can be treated white they are happening for say, fever or diarrhea… there are different types of diarrhea the when and how a particular type of diarrhea can be treated, that we learned from them. Then we contacted [nearby town name redacted] subcenter for trainings. We submitted our phone numbers and papers to them and whenever required they the information via MO ([name redacted]), Then we go for trainings. They sent names of 50 RHCPS to [town name outside of site 2 redacted] Hospital now they are giving us trainings. I think [name redacted] told you about this training…

AB-Yes he told us.. he is also attending it right?

No, he exceeds the age limit by the Government. He is now 61 years old and the Eligibility criteria for us is up to 45 years.

Am I right?

PS- Look we are not here to assess you rather we are going to everybody like you and trying to gather few information so that we can bring a good programme for you for your better practice.

So For this purpose, whom can be involved? how can we arrange this programme?

You can arrange seminar or one day training camp.

PS- As you need to use antibiotics, do you observe any changes in its effectiveness? For say, one antibiotic needs to be given for more days than previous time… or may be in higher dose…

No. it works well. Look the antibiotics are already in high power (bhari) so I need to use vitamin capsules with it like vitamin B complex, for children, A to Z drop etc.

PS- What do you mean by high power (bhari)?

For say clavam, it has three ingredients Amoxicillin, potassium calvum… so it is a high-power medicine.

PS- What would be the low power (kom ojon) antibiotic?

Taxim, O2 – Ofloxacin 200. The dosage depends on situations. for say there are Mox 200 and Mox 500. So it depends.

So what other doctors told you?

They are sharing their own experiences like problem of procuring medicines, referral system etc.

Yes I refer a patient on the third day.

PS- So, what medicines do you give to patient for initial two days? Take example of patient with fever.

Paracetamol with an antibiotic and antacid and if the patient feels weak then I give him/her vitamin as well.

PS- Do you ever noticed that one patient comes to you and then moved to another practitioner?

I cant say. I don't know.

PS- And the opposite scenario like he /she went to another doctor/ practitioner before coming here?

No. I never asked. How can I say?

PS- Okay. As you mentioned about [name redacted] , so do you have contact with any other doctors too?

It can be misleading to take advice from other practitioner so better I refer.

PS- Do you have or know any association?

[name redacted]'s Association.

I heard that [name redacted] also arrange classes for you…

Yes. He does. [name redacted] from [nearby town name redacted] passes the information among us. I have attended the training session twice that's it. I don't find it good.

PS- Then why are you attending the Government training?

In case of emergency, I can easily refer my patient to Government hospital instead sending him/her to the union practitioner.

PS- Have you ever noticed that there are various kinds of antibiotics in market, what say?

I don't know. As I only do the basic treatment, I can't tell you anything about it. I just tell them to complete the medicine until they feel better that's it.

PS- So do you suggest the same for antibiotics also?

For antibiotics, I tell them to take it for 7 days. In case of antibiotics, the effectiveness will minimize after 7 days you know…

PS- Do your patients give you the fees immediately?

No no. they don't pay me immediately. We have halkhata also. They pay me according to their convenience. There are 75-80 customer who has halkhata now.

PS- So how do you manage?

If a patient needs 200 rs medicine, I take 100 rs from them. I buy medicines according to the immediate need and affordability of the patient. If medicine cost exceeds 500 rs, I take the whole money from them. For me, it is not possible to spend 500rs on one patient on debt.

PS- Okay. As other practitioners told us that they noticed few of the antibiotics losing it effectivity, Do you agree with them?

Like? Give me the examples.

PS- Ofloxacin… sulfabactum…

You need to check the group first… Ofloxacin works well.

Cifran…

Yes Cifran works well in case of fever

Norflox?

Yes… it works well too.

Have you ever heard the term Antibiotic Resistance? Even from the trainings?

No.

Have you ever faced any difficulty in getting any antibiotic from market?

If the store running out of antibiotics, then I suggest the patient to visit subcenter. I never prescribe medicines.

Tell me more about this place, Rajapur…

Here people from different religion like Hindu, Muslim, Christian, Buddha stay together. Most of the people are farmers and they go to Kolkata for Daily wage job.

So they also keep poultry? And poultry needs medicines?

Yes. Here is a Model Farm. They bring medicines from Tagore society.

Do the veterinarians come here?

No. We take the affected chicken to them.

So in case of emergency, especially in nights, don’t you ever need to treat the chicken?

No. I don’t know how to treat them.
