# Supplementary material for: “If It Works in People, Why Not Animals?”: A Qualitative Investigation of Antibiotic Use in Smallholder Livestock Settings in Rural West Bengal, India
Source: Antibiotics (Basel). 2021 Nov 23;10(12):1433. doi: 10.3390/antibiotics10121433 (PMC8698124; doi:10.3390/antibiotics10121433)
Supplement: Supplementary file 1 [file antibiotics-10-01433-s001.zip › Supplementary S1_ Interview Transcripts/Site 2/LK24 (site 2).pdf]

Code for Study - ‘If it works in people, why not animals?’: A qualitative investigation of antibiotic use in smallholder livestock settings in rural West Bengal, India: LK24, Site 2

Date: 14/01/2020

Location: Site 2

Interviewee: Livestock keeper (LK)

Interviewer: Mathew Hennesey (MH), accompanied by Dr Meenakshi Gautham

Transcription: Soumen Samanta (SS)

In Bengali language

MH- Mat Hennessey

LK- livestock keeper

MG-Meenakshi Gautam

SS- Soumen Samanta

All answer by LK is as ‘A’.

The interviewer came to know that this farmer sold his cows.

Q: Why did you sell those cows?

A: very laborious task, no proper supply chain of milk is there. Because

SS: Get spoiled?

A: Get spoiled, have to sell in less price. In city, price of 1 litre milk is..

SS: 30/ 35 or 40 rupees.

A: 40 rupees solid, but here the best quality milk get maximum ₹20-25/litre not more than this. If proper supply chain was there then huge production could be done, here Jersey cow grow well,.

MG: Whom did you sell those cows to?

A: To my local peoples. Once I declare that my cow is for sale. People came to know by ear to ear. The last cow that I sold at *Sonaga* area, it gave milk of 15-18litre/day. Then how much milk it is? If the milk was sold at ₹40 then how much money could be obtained? Selling at ₹20 is of no value. That's why I don't feed them mash which is ₹36-38/kg. Milk price is less so why I should feed them mash? Fed them paddy straw,

SS: Grass?

A: No grass is available, I used to cultivate grass in field for cows but it is very laborious, I thought it is impossible for me, I told them (other member), if you can you do or sell them. And this cow was also under plan to sell, when it was not happening (not coming in heat/estrus) after giving many medicines by doctor from *(local area name redacted),* (His homeplace) with whom I had very good relation, whenever he (my son) call him he tells to send father (me) and to take medicine, then I finally think of selling it. And the day when the buyer came and the cow came into heat that day. I was lucky.

MH: Which doctor did you consult?

A: *(NGO name redacted)* doctor, his house is in *(local area name redacted)*, his name is *(person's name redacted)*, he is a doctor but he does research. As here is no doctor present he comes once/twice in a month.

SS: Is it Dr. *(person's name redacted)*?

A: Yes, yes it is Dr.*(person's name redacted)*. He is very gentleman and good guy.

MH: What did the doctor do?

SS: What the doctor did or how the cow came in heat?

A: He gave capsules (medicine) then suddenly one day the cow came into heat.

MG: What is the medicine? Do you remember any name? Any capsules?

A: That was white type medicine, I had prescriptions.. stop, let me find.

(MG was instructing SS and MH about questionnaire)

(He showed an old prescription in which Vitamin A and some mineral mixture was written).

(person's name redacted), when you told *(person's name redacted)*, his name came in my mind. A good person must be said as good, not to a bad guy as good.
MG: Where did you get this medicine from?

A: From there (*(NGO name redacted)*). And the powder, that was given by the doctor on subsidy.

MG: Do they charge for it?

SS: Did he take money for this?

A: No. From whom he took I don't know but from us always in subsidy. The capsule I had to buy. Sometimes when he came he doesn't want to take charge usually but we gave him something happily.

MH: Does the cow in calf now? Is she carrying any calf now? Is she pregnant?

A: She is pregnant now. It will calve in 'Falgun'(a Bengali calendar month; 2months later).

MH: Is he getting milk?
A: No, now she will not give milk. Until it gives calf it will not allow to come closer.

SS: Did you give her any medicine?
A: No, after coming in heat I didn't give any medicine.

SS: Was it artificial insemination or mated to a bull?

A: Artificial insemination.

SS: Where it happened?

A: If I call them (*(NGO name redacted)*), there is special cage for her in home. In this time more walking of the cow is not good, if walks (?) shake. For which it is kept in a particular place, that’s all.

MH: Did they charge for that?

A: They took 150 rupees.

MH: How many times they did artificial insemination?

A: Only once. Our cows conceive at one time, never twice. We tell the doctor, don’t miss, if you miss it's my loss and also your loss (reputation).

MH: What types of problem does he have with cows?

SS: What types of problems/diseases of your cows happen?

A: In rainy season more "eso"(FMD) occur and sometimes diarrhoea. After giving medicine it get cure.

MH: What do you do at that time?

A: Immediately I have to go to doctor to take medicine.

SS: *(NGO name redacted)*?

A: *(NGO name redacted)* or some people work here through *(NGO name redacted)*, I tell them.

SS: Is here their house?

A: Yes, he is from *(local town name redacted)*.

SS: Is it (paravet) *(person’s name redacted)*?

A: Yes *(person’s name redacted)*. He gives medicine.

MH: Why do you choose to call the *paravet* instead of other people?

SS: Why do you call *(person’s name redacted)*?

A: Doctor is not always available at *(NGO name redacted)*. When I call *(person's name redacted)* and tell to come to my home. He instantly comes with his bike.

MH: How much you have to pay for that?

A: Not much. We also not give. But he comes happily, ummm... He came at night during delivery of my brother's cow. The calf got stuck (dystocia). I called him to come for delivery of the calf at that time. He came.

SS: How much he took then?

A: 150.

SS: How much he took from you when he comes to you?

A: 150.
MH: How do you pay?

SS: How do you give the money? Cash?

A: If I am having cash money I give it. But if not then give it later. He will not come to collect, you have to go his place otherwise he will not come next time.

MH: How long after you have to give that?

A: Within 2-4 days.

MH: What type of treatments, medication is given?

SS: What type of treatments he does? Injections/ tablets/?
A: During delivery he gave injections first.

SS: No, when he came to you last, what treatments he did?

A: When he last came to me to do AI he washed hand with soap and put the AI gun pipe inside.

SS: That is the AI, any other oral medication?

A: No other oral medication was given.

SS: When was it?

A: 6-7months back.

SS: Had he came during fever also?

A: No that time we have to go and tell. Like here is wound in leg or the neck has swollen and bent. It's a dangerous disease. In that it is very difficult to save them. So to prevent that, you have to give medicine from before.

SS: Doctor comes and gives that medicine?

A: Here in south one *(NGO name redacted)* is there. There is also veterinary doctor. They come and make camp in our local places. Give vaccine to goats, poultry.

(It's 10.30 a.m. I have to go school to pick up my grandson. I am having 10mins for you.)

MG: Can we talk to your daughter in law after you?

A: No, she has gone to market. She is my daughter (indicating the lady). She will cook. I will go bank after that.

MH: How many poultry do you have?

A: 8

MH: Where did you get these from?

A: We are having group (Self help group). From group we got 9. In our home there was some *deshi* poultry. They are no more now. We ate them. In our area rearing of poultry is more. Now I am having only 8.

SS: From group you get the chicks?

A: From groups only once I got. Otherwise in our house.

SS: In house how do you get them?

A: buy eggs and incubate.

MH: How do you incubate those *deshi* poultry in home?

A: Buying the eggs and keeping the egg under a brooding hen for 21days.

MH: What do you use the poultry for?

A: Mainly for eggs, sometimes for meat also.

MH: Who looks after them?

A: All the people of our house, women.

MH: Where do you keep them?

A: In which way you came to my home, beside that way there is a shed for them.

MH: Is it separate from home?

A: Yes, a separate one.

MH: What type of problems of the poultry happen?

A: Dullness, anorexia, cough and cold, crackling sound comes from throat.

MH: What do you do when these problems happen?

SS: What do you do then or from where do you get the medicine?

A: From model we take, we call *(person's name redacted)*(paravet), come from *(NGO name redacted)*. He gives medicine.

MH: Do you have to take the poultry to him?

A: No, no. You have to tell the problems going to him. He gives the medicine.

MH: Where do you get the medicine from?

A: *(NGO name redacted)*.

MH: Do you get the medicine from any other places?

A: Yes, different personal chamber are there. In *(local town name redacted)* there is one govt. hospital. In gosaba from where human medicines are available the animal medicine is also available.

MH: Where would you get the medicine from the block or from shops?

A: From blocks all medicines we got. But many due to time not get as it opens in11a.m. and close at 4p.m. Then you have to go the side shop and tell.

MH: How do you know what medicine to get?

A: Subrata babu or from *(NGO name redacted)* they prescribe. He (*(person's name redacted)*) is also having medicine. Which medicine is not having to him he write and you have to buy.

MG: He said diarrhoea happens.

MH: What did he do when the cow get sick last time?

SS: Before AI, what problem happened? You said diarrhoea.

A: Oo diarrhoea, it happens sometimes.

SS: Then what do you do?

A: Then you have to go to Subrata and tell that the cow is in diarrhoea, and ask him to go with me to my home or to give some medicine. If he comes, good; otherwise he gave medicine for 3to 5 days, daily once.

MG: When it happened last time?

A: Not many days, about one month ago. She(cow) is having disturbance in liver. That's why diarrhoea occurs. Extra vitamin has been given, wheat flour. Don't give mash now. Flour is mixed with hot water then cooling it is given to feed. If it is more hot water then problem arises. Two time feeding is done. If given water at morning. It will not give mouth to straw.

(The old man want to leave)

MG: Do you having any paper (prescription)?

A: No, in diarrhoea sometime after feeding bamboo leaves it gets cured. As it is pregnant one, it is advised not to give more medicine.

MG: When the chicken fell sick what do you do?

A: Give capsules. After grinding it you have to feed them.

SS: Do you have any paper or prescription?

A: No, no. They only tell in mouth. White colour medicine it was. Had to grind it and divide. Suppose for 8 birds they divide the medicine into 8 and tells to feed the birds one day.

MG: Do you having any wrapper?
A: No, no, it happened many days ago. No wrapper is present.

MH: Do you give human medicine to animal?
A: No.
MG: Any wrapper?
A: (He started to describe the severity of 'bulbul'/ 'aila' storm in *(local area name redacted)*area. many people left, also tells that after 10-12 years later it will be under water).

MG: Where you will go then?
A: People who are having land in cities they will go there, who is not having they will die.

SS: Where did you go in last storm?
A: In *(local area name redacted)*health camp, in relief centre. That time we had no drinking water.

MG: How many people do live here?
A: Total 9, but in home now having 6 others are at cities to work there and to earn money for us.

MH: Do you sell the eggs?
A: Very less, mostly we eat it.
(The old man finally leaves)

SS: Does it ever happen that you bought medicine and it becomes extra. And then what do you do with those extra medicine? Do you throw it away?
A: No, if it is extra it is kept, if in future that is needed it is used.

MG/SS: What do you feed the chicken?
A: Mash, grind rice.

SS: And what to the cows?
A: Previously I feed them mash but now paddy straw and wheat flour, salt.

MG: Do you mix any medicine in the feed for the poultry?
A: Yes, vitamin medicine. (red coloured vial)

MH: How often do you use that?
A: previously in every month but now I had not given for many days.

MH: Why not?
A: Now those are not brought for many days.

SS: Did it happen that doctor gave medicine but it not worked?
A: Yes.
SS: When?
A: Many days ago.

SS: Then what do you do?
A: Again after 2-3days I go to the doctor. And he changes the medicine.
SS: Same doctor?
A: Yes. *(Person's name redacted)* (uncle), he is a close friend of my brother, whenever any problem happens, he looks after it.
SS: You need not to go other doctor?
A: No.
SS: In every case, it get cured?
A: Yes.

MH: Do you know what the medicine is?
A: No. It is oral medicines. When it changes it is also an oral medicine. My brother looks after these things.

MG: Do you have any medicine in house now?
A: Only having human medicine. In our house, everybody is ill. I am having diabetes for 16years.

MG: Do you hear the term 'antibiotics'?
A: Yes, heard it.
MG: From whom?
A: From my brother.
SS: What is it? Is it a food/ medicine or..
A: No, few days ago, I heard from my brother that it is given in disease.

MG: Did *(person's name redacted)* give antibiotics?
A: No, I don't know. Only know medicine is given.
SS: no wrapper?
A: No, in storm everything has been lost, which happened 2 months before.
