# Supplementary material for: “If It Works in People, Why Not Animals?”: A Qualitative Investigation of Antibiotic Use in Smallholder Livestock Settings in Rural West Bengal, India
Source: Antibiotics (Basel). 2021 Nov 23;10(12):1433. doi: 10.3390/antibiotics10121433 (PMC8698124; doi:10.3390/antibiotics10121433)
Supplement: Supplementary file 1 [file antibiotics-10-01433-s001.zip › Supplementary S1_ Interview Transcripts/Site 2/LK25 (site 2).pdf]

Code for Study - ‘If it works in people, why not animals?’: A qualitative investigation of antibiotic use in smallholder livestock settings in rural West Bengal, India: LK25, Site 2

Date: 14/01/2020

Location: Site 2

Interviewee: Livestock keeper (LK)

Interviewer: Mathew Hennesey (MH), accompanied by Dr Meenakshi Gautham

Transcription: Soumen Samanta (SS)

In Bengali language

MH- Mat Hennessey

LK- livestock keeper

MG-Meenakshi Gautam

SS- Soumen Samanta

All answer by LK is as ‘A’.

SS: Our work is not under Tagore Society but the doctors who are there, they are also involved in our work, we are working with Tagore society.

SS: What is your name?

A: My name is *(person’s name redacted)*.

SS: How many people do live in your family?

A: In my house total member is 7.

SS: What is occupation?

A: Cultivation (Paddy and vegetables).

SS: Any livestock?

A: 4 cows, 10goats.

SS: Any poultry?

A: No, no poultry. We have 2 hens.

SS: Ducks?

A: No.

MG to SS: Can you ask him how many fields/land he has?

SS: How much land do you have?

A: 3 bigha, 1 acre.

MG: Is that his main occupation? Or does he have other occupation?

SS: Is the cultivation is your main occupation?

A: Yes. Cultivation is my occupation.

MG: Do you yourself work in the field?

A: Yes.

MG: What do you grow in the fields?

A: Paddy, vegetables. Paddy one time in a year, in rainy season.

SS: Is these vegetables is yours?

A: Yes.

SS: Where do you keep these cows?

A: There, near my house. (indicating the shed, a few metres away from his house which is not attached with his house)

SS: Why do you rear these cows and goats?

A: It is India. Here we specially Hindus are related with cows. Human brain develops with milk. Milk is an ideal food, for getting that milk cow is reared mainly. The cow dung that is obtained that is used for fuel, fertilizer.

SS: And what are hens used for?

A: Hens are reared mainly for eggs, and also for meat too.

MH: What for goats?

A: Goats are sold mainly for meat for family income. No other source is there.

MH: Who do they sell the goats?

SS: Where do you sell the goats?

A: There is a market and also the ‘bepari’(who buy from farmer and sell in market) comes here.

MH: Who do look after these animals?

A: Family members.

MH: How do they look after the animals? How do they feed the animals?

SS: Can you tell us how do you rear the animals?

A: By feeding grass that is cut from the field, also by grazing, by feeding broken rice, rice bran/ dust too.

MH: Do they give the animals anything else?

SS: Do you give anything else as feeding other than this?

A: Sometimes something which I bought is given, mash type feed is given.

Q: What do you give to the poultry?

A: Mainly home food like broken rice, sometime mash also. To cow straw, grass, rice bran is given.

Q: Do they do any routine treatments with the animals?

SS: Do you follow any regular treatments for them?

A: Yearly 1-2 times vaccination is done. It is surely done. And when disease problem occur then treatment is done.

SS: Do you give them ‘krimir osudh’(deworming medicine)?

A: Yes, Deworming medication, vitamin medication is done.

MG to SS: What is this ‘krimi’?

A: Worm

SS: When did the cow/goat get ill last time?

A: Last time in this year a problem was seen in goat. We usually treat them in *(NGO name redacted)* animal department.

SS: What happened that time?

A: The goat was suffering from bloat. It was not eating, got illness, got fever. After giving medication from there it cured.

MH: Only one goat suffered?

A: No, two goats.

MH: What did you do then?

A: Went to *(NGO name redacted)* animal hospital. They gave medicine.

SS: Do you remember what medicine was that.

A: I don't know.

MH: Why did you go to *(NGO name redacted)* not to anywhere else?

A: Then I have to go through an old history. *(Person's name redacted)* is an important person in this area. He developed this area by starting good practise among us. By arranging meetings again and again, he made the people aware. He actually made us aware. We were a part of his work. We helped him in this work. I was one of his students. That's why.

MH: To whom do you go for vaccination and deworming?

A: They organize camps. From there we vaccinate our animals and also get the deworming medicine from there.

MH: Do you have to take the animal to camp or do they come to home?

A: Yes, we have to take them in the camp.

SS: Where do the camps occur?

A: Camps are held in particular local places like nearby temple ground. We have to take the animals there. Camp is held including 20-25 houses in an area.

SS: What problems were last seen in cows? Or what are the problems are seen in your cows?

A: Fever comes, mainly fever occur, bloat if take unwanted things, diarrhoea.

SS: Hoof ulcer?

A: No after vaccination it never came. It is called 'eso' (FMD).

When there is grass in field and cows are allowed to graze then diarrhoea occur. When feeding is more diarrhoea occur.

MH: What do they do when the cows have diarrhoea?

SS: What do you do when cows have diarrhoea? Anything or any treatments?

A: If you go to the *(NGO name redacted)* they will give you the medicine. Cost is very less. Or if you call them they come to home.

SS: How do they give medicines?

A: As cases occur according to the type they give. They give medicine dose wise like sometimes for 2 days and sometimes for 3 days. Sometimes you have to eat them for 7days. They give right dose and by that the cows get cured.

SS: Who give the medicine?

A: *(NGO name redacted)* people. There is doctor, veterinary doctor.

SS: But Dr. *(person's name redacted)* is available only for two times in a month.

A: If he not present also, there is one person named *(person's name redacted)*who is also expert. He is there for long times. He is not much less than a doctor. (*(person's name redacted)* is a paravet)

MH: Why do they call a paravet? Or at what situation do they call a paravet?

SS: When do you call that *(person's name redacted)* or *(person's name redacted)*?

A: When the case is very serious, get very much ill. If I call them they come.

MG: Can you tell the name of the paravet? Is that only one person?

SS: Can you tell the name of paravets? Is that only *(person's name redacted)*?

A: *(Person's name redacted)* also come. Doctor also come and *(person's name redacted)*also come. I have a good relation with *(person's name redacted)*. Whenever I go through hard times in my animals he saves me. I also have good relation with Dr. *(person's name redacted),* he also help us.

MH: If his animal is in critical condition why does he call paravets not *(NGO name redacted)*?

SS: The paravets are of *(NGO name redacted)* who get trained from there and stay there.

MG to SS: Can you confirm this?

SS: Is *(person's name redacted)* also from *(NGO name redacted)?*

A: Yes. He mostly looks after there. He manages efficiently.

MH: Do you know any name of the medication?

A: (Laughs) it is nothing to know the names. Medicines like paracetamol, antibiotic, ampicillin group medicine, vitamin b complex group.

MH: Do you know what antibiotics are/ what are the other antibiotics? What they do?

A: No, no. We don't want to know that much. When we face problem we go to them and it solved. Not want to go deeper.

MG: When last time the antibiotic was given to your animal?

A: Once fever happened, paracetamol could not decrease the fever of my cow. Then ampicillin was given.

SS: For how many days it was given?

A: I could not remember but 3 or 5 days course was given. And the cow cured.

SS: How it was given?

A: I think. It was twice in a day.

MH: How it was administered to the cow?

SS: Was it oral medication or injection?

A: I can't remember, it was oral medication.

MH: Who gives the medication to the cows?

SS: Do you yourself feed the medicine? Or other people?

A: Myself.

MH: Do you having any medicine package/ wrapper?

A: No, no.

MH: What did you do with the medicine package?

A: Throw it away.

MH: What was the last problem with the goats?

A: As I said before swelled belly (bloat), anorexia.

SS: What was the problem with the poultry?

A: Only two are having now. Nothing happened to them yet.

MH: Did you have any problem before with the poultry?

A: Yes, many times ago. After giving medicine that we took from *(NGO name redacted)* it got well. But we ate those poultry later. Dullness, depression, taking less food, watery diarrhoea.

SS: That time also did you go to the *(NGO name redacted)*?

A: Yes, yes. After taking medicine from there it cured.

Here is also government hospital at *(local town name redacted)*as my problem solved here I don't need to go to the hospital.

MH: Did you ever go to block hospital?

A: Yes went last time, willingly, as it is our hospital. I had a heifer which was not coming to heat. From here (*(NGO name redacted)*) they gave medicine and told that it will be all right. But without waiting for that medicine to effect I went to block. What medicine *(NGO name redacted)* people gave, they (Block people) also gave the same medicine. But I didn't tell that I had already taken this medicine before. Then taking that medicine I again went to *(NGO name redacted)* and told what medicine you gave us they also gave the same vitamin type medicine. Then I told what to do now. They advised to feed that medicine too. And the jersey came into heat and I told them then to do AI.

MH: How much does it cost in cow's diarrhoea in *(NGO name redacted)*?

A: Not that much, maximum 100rupees and minimum 15 to 20 rupees. Most economic, easily available is *(NGO name redacted)*.

MH: How much does it cost if you call the paravet?

A: 20 rupees for visit. Medicine cost is extra.

MG: If you go there?

A: They don't take that 20 rupees.

MH: How much does it cost in case of block?

A: They take 5 rupees.

MH: Would you buy medicine from anywhere else?

A: No, no. Whatever I need I got from them. Till now I need not to go to buy anywhere else. The *(NGO name redacted)* people give all the medicine.

MH: Would you have ever used human medicine in animal?

A: No, no.

SS: Your daughter was talking about some metrogyl/ nor metrogyl. (Metrronidazole)

A: If it happened, it's their suggestion. Whatever the *(NGO name redacted)* health department advise I follow. I need not to see any other place.

MH: Had you ever taken medicine that is made for animals for yourselves?

A: No, no, never.

SS: What do you do with the extra medicine that is left?

A: Most of the time they give limited dose wise medicine. Not like that they gave lots of medicine.

SS: Do you also use antibiotics for yourselves?

A: What the doctor told we had to follow that. When I was too much ill and I was admitted iin PG hospital(SSKM), Kolkata then they gave me antibiotics. Its natural. I got stroke.

MG: Who gave that 'ampicillin' that you told before?

A: I could not remember.

SS: Was it from *(NGO name redacted)*?

A: Probably from there.

SS: Did doctor *(person's name redacted)* or *(person's name redacted)* give that?

A: Doctor *(person's name redacted)* probably.

MG: Did they give the medicine or prescribed it?

A: Doctor wrote and the other people there gave the medicine from their clinics.

MG: How much did it cost?

A: They took 58 rupees and gave medicine for 5days.

MG: Do you have that prescription still? Or any other prescriptions?

A: No, I think. I have to search a lot to find. Assume that there is no prescription now. May be somewhere there.

MG to SS: Ask him do they give medicine when they fall sick or any other reason also?

SS: Do you give medicine when they fall sick or other time also?

A: In camps, deowming medication, vitamin medication is given. Twice in a year camp held here. In upcoming summer it will be held. And again it will be held after rainy season or before rainy season. And when ever needed if you go you will get medicine. There is no problem.

Again I am telling that during my father's time the condition was very bad here. After coming of *(person's name redacted)* in this place as a teacher, he felt our conditions and developed this area. He taught us how to live in a better way. He continuously made us aware. He made a beautiful environment for us. He set up health centre for us, animal hospital for animals, agriculture department from where we got seed, tractor etc for cultivation. The progress of we people here is due to Tushar Kangilal. If we don't take his name we will be in the list of unfaithful / traitor.

MG: What about the goats? Do you give any medicine for growth?

A: Yes, yes. In camps they give. And also if you go and tell they give. Today I was thinking that my goat is not growing well, I have to go there (*(local town name redacted) (NGO name redacted)*) once to take the medicine.

MG: Have you gone before?

A: Yes.

MG: What did they give?

SS: What did they give? Oral medications? Powder?

A: Yes, powder type medicine.

MG: Do you know what that medicine was?

A: No, no. I don't know.

MG: Do you having any medicine boxes, packets, papers or anything that you can easily show us?

A: No, no. I am not able to show you. During puja, total house was cleaned. Everything vanished.

MG: So all the medicine you got from *(NGO name redacted)*?

A: Actually for me the condition is like that I completely believe them and whatever they give I follow. (also told a story of Jesus, once there was storm and Jesus stop the storm..)

MG: Was there any major illness in your animals?

A: No. If any little problem arises, we instantly go to them. In that way we are educated, if any problem is seen we go there. Take medicine and it cures. Not allowed to worse the condition.

MG: How long you have these goats?

A: Many years. Every year the older one is sold and the kids are allowed to grow.

SS: From where do you buy the goats?

A: In our villages, from any house. The kids are bought at 500 to 600 rupees. And one thing is that the male goats are generally sold. The female goats are kept. They produce offspring.

MH: Do you use male goats for breeding?

A: I sell them. For breeding, particular some male goats are there in an area. They took 50 rupees for each time breeding. In my area there are 4-5 houses like that. 2 days ago one goat was breed at 50rupees.

MG: What contact do you have with the pranimitra (Lipika)?

A: They do not treat animals. They come in camps. They gave injection in camps (vaccination). Also she helps us in times we need.

SS: Did she ever treat your animal when your animal is sick?

A: No, I did not take help in that way. In any problem I directly go to *(NGO name redacted)*.

MH: Does *(person's name redacted)*(pranimitra) treat animals of surroundings?

A: Yes.

MH: Do you know what type of animals she treats?

SS: Does she treat all animals like cows, goats?

A: I did not take any help from her for treatments. But I have heard that she treats.

SS: Did it ever happen that treatment is done but it not cured?

A: No. Animal gets well after treatment.

MH: Do you have any questions to ask to us?

A: I wish to know from where you people came.

MH: England.

SS: He came from a Veterinary college of London. He came here for this project. We are staying here for 1 week.

SS: I came from Veterinary college of Kolkata. Dr. *(person's name redacted)* is a teacher of that college.

Household: so you people are doctors. (smile). Good, good. The society should progress.

(Household came to know Soumen is from his old place, he(household) offer tiffin but they refuse to take)

MG: Tell us about vaccination? In each animals separately, and how often?

A: Camps are held. All types of animals are vaccinated there. According to the environmental condition they arrange camps, twice or thrice in a year.

MG: When was the cow last vaccinated?

A: In the month of 'kartik'(a Bengali month). Three months back.

SS: All animals?

A: All animals got vaccinated that time.

MG: Had you take all the animals to the camps?

A: Yes, all animals.

MG: How did you take those animals?

A: It is very near where the camp was held. Camps are generally held at nearby places.

MG: Do you know what disease for the vaccine is given?

A: 'Eso'(FMD), 'gola fola' (HS) in cows.

MG: For goats?

A: I don't know for what in goats. In goats deworming is done.

SS: In poultry?

A: 'Ranikhet'.

SS: How long are you here?

A: *(life history redacted)*. Previously 30-32 cows were here, 4 to 6pairs bulls were here for ploughing. But now it decreased.

MG: What do you do when you people get sick?

A: There are *(local town name redacted)* health centre, govt health centre in *(local town name redacted)*, otherwise in Kolkata.

MG: Where do you go first?

A: *(local town name redacted)*health centre.

MG: Who is there?

SS: To whom do you go? Does any doctor present there?

A: Yes. Doctor is there. '*(person's name redacted)*', '*(person's name redacted)*' and also good doctors are here. Previously *(person's name redacted)* a surgeon of Medical college, used to come. Now also well doctors from kolakta come and do treatment.

SS: Did you ever take medicine own?

A: No, it is not right, I am not in that line.

MG: Have you heard the term 'antibiotics'?

A: Yes, it's nothing new. Allopathic treatments mean you have to go through antibiotics.

SS: Did you ever go to shop to buy medicine when you have diarrhoea?

A: No, no. *(person's name redacted)* didn't give us that education. He told us to drink boiled water or geolin(Water purifier) mixed water. Don't take rotten food. We don't take medicine of our own.

MG: Did anybody in your house take antibiotic recently?

A: No, no one ill now. I was ill almost 4years back.
