# Supplementary material for: “If It Works in People, Why Not Animals?”: A Qualitative Investigation of Antibiotic Use in Smallholder Livestock Settings in Rural West Bengal, India
Source: Antibiotics (Basel). 2021 Nov 23;10(12):1433. doi: 10.3390/antibiotics10121433 (PMC8698124; doi:10.3390/antibiotics10121433)
Supplement: Supplementary file 1 [file antibiotics-10-01433-s001.zip › Supplementary S1_ Interview Transcripts/Site 2/LK26 (site 2).pdf]

Code for Study - ‘If it works in people, why not animals?’: A qualitative investigation of antibiotic use in smallholder livestock settings in rural West Bengal, India: LK26, Site 2

Date: 14/01/2020

Location: Site 2

Interviewee: Livestock keeper (LK)

Interviewer: Mathew Hennesey (MH), accompanied by Dr Meenakshi Gautham

Transcription: Soumen Samanta (SS)

In Bengali language

MH- Mat Hennessey

LK- livestock keeper

MG-Meenakshi Gautam

SS- Soumen Samanta

All answer by LK is as ‘A’.

SS: As we may miss some points of our conversation, can I record our conversation? And as you are giving us the information I will give a consent form that you have to sign, and one copy will be with you and another with us. Do you agree?

A: Yes, Okay, no problem.

To make a farm huge money is needed. As we run a farm we follow this chart of medicine, vaccine 3 like F1, IB,

SS: Who does give those vaccines?

A: Myself.

SS making MH to understand the chart.

MG: What about the dosage?

SS to LK: here is written 5ml, for how many birds it is?

A: 100birds. It is for 100birds chart.

SS: Do you give this with water?

A: Yes. In the hopeer through which we let them drink.

MG: How often?

A: Daily once for 5days, all for 5days.

MH: How many birds do you have together?

A: I had 400 birds but 15 days ago I sold 100, then I had 300(100croiler+ 200broiler). Then in last few days I sold 50-60 birds a day. Now I am having around 150 birds.

MH: How long are you keeping birds?

A: I used to keep for almost 7years before the year 2009 (when the storm ‘AILA’ struck Sundarban). Then from last one and half year again I am keeping. In that time feed cost was 700-800rupees/70kg but now 2050rupees/50kg.

MH: Where do you buy the birds from?

A: Now peoples need is like they buy at the newspaper printed rate. If I say 5rupees more it will not happen.

SS: From where do you buy those birds? *(shop name redacted)*?

A: *(local town name redacted)*, through *(person's name redacted)* doctor(paravet), means a little less. No not less, he (*(person's name redacted)*) is also doing a business isn't it?

SS: He told *(shop name redacted)*then then *(shop name redacted)*gives you?

A: Yes.

SS: Is *(shop name redacted)* is that *(local town name redacted)*shop where all poultry items are available?

A: Yes.

SS: How many birds do you buy at a time?

A: 200-300. Our farm is not closed anytime, this time due to little

MH: How many people do live here?

A: In my house 9 people.

MH: How many people work with poultry?

A: I myself give most of the time and my son and husband also help.

MH: Do you have any other income?

A: One grocery shop is also there.

SS: Cultivation?

A: Have 2 bighas land. Paddy and some vegetables cultivation.

MH: How do you learn to look after poultry?

A: From *(NGO name redacted)* we learnt to rear poultry by a 7days training programme through group.

SS: Then?

A: We made a chart.

SS: From where you get this chart?

A: From there they give. We wrote it.

This *(person's name redacted)* is also from there, we took training from *(person's name redacted)*. *(Person's name redacted)*also took training from him. He is a male, so he practise outside and we are women, we can't go outside like him so could not progress.

MG: Who attended the training?

SS: Who attended like you?

MG to SS: just ask who attended?

SS: who attended the training?

A: women of the villages. Few rear 5-10 birds, nobody rear like me here.

MG: From here who attended?

A: I myself.

MG: What did you learn there?

A: How to keep chicks, how much place to provide, how to feed and water and how to vaccinate. All these they taught us.

MG: What did they teach about medication?

A: For 5days,..I don't know the english name so I wrote this chart from there. (The chart contains a schedule of medication and vaccination list). At 5days F1 (of Ranikhet) vaccine is given, at 15days IBD vaccine and at 25days F1 vaccine is given. These 3 vaccines are given.

SS: Who did the vaccination?

A: I myself.

MH: Where did you get the vaccines from?

A: From *(person's name redacted)*(paravet). He brings it and keeps in his freeze.

MH: How do you give the vaccines to the chick?

A: IBD or F1 is given through a dropper in eyes or nose. And when ranikhet comes, that vaccine is given below skin that also I can inject.

SS: When do you do that?

A: when the disease seen, when birds do chalky diarrhoea, that time. Otherwise not needed.

If we follow this voucher (chart) and give medication, ranikhet is prevented.

SS: you said about these 3 vaccines, all are given in eye/nose?

A: Yes.

MH: How do you know that it is ranikhet?

A: It mainly seen in summer time. Whitish chalky diarrhoea, dull and depressed, the comb becomes blackish, head get down. It starts with one or two birds and if you do not look after it then it spread in all birds. It is a viral disease. You have to isolate the affected birds immediately.

MH: Where do you keep those birds?

A: In a separate place.

MH: What happened to those birds?

SS: Do the birds recover?

A: Most of them die, specially the *broiler* birds, the white one. And the red birds which are called *croiler* survive, recover. If they (red birds) affected also they die only 1 or 2 in number; in broiler it is more.

SS: Are there both *broilers* and *croilers* in your farm?

A: Yes, both. *Croile*r is kept at one side.

MH: What do you do when you think there is an outbreak of ranikhet?

A: Then we took advice from doctor (*(person's name redacted)*) or from Dr. *(person's name redacted)*. There is also doctor at *(local town name redacted)*but we don't go there.

MH: How do you decide whom to contact?

SS: Whom to go? How you decide? *(person's name redacted)* or *(person's name redacted)*?

A: As *(person's name redacted)* is giving us and he is close to us we contact him first. Without saying him we can't go anywhere.

MH: What they advice or do?

A: They tell to keep separate the birds or there is medicine to spray in farm or to spray in birds also. We are trying our best but specially the *broiler* dies, *croiler* recovers

MH: Do you know what medicine he gives or spray?

A: No, some powder he gives. (Her son called the doctor and tells the name of the spray "vercon-s".

SS: Do you have other animals also?

A: Have 7goats (5 pregnant female and 2 male). They will give kid in March.

MH: Did she know what the medicine the doctor sprays?

SS: Her son is calling the doctor to know this.

MH: Where do you get the medicine from?

A: From that doctor (*(person's name redacted)*), whatever happens we take from doctor. Actually we have no money, whatever happens we took from doctor and return him later.

MH: Do you buy from anywhere else?

A: He (*(person's name redacted)*) is doctor so he divides the medicine in 5ml, 10ml like that. We are having 400-500birds so it may cost more. So I took a total vial and it reduce the cost at least 500 rupees.

She showed the feed packet.

SS: From where you took that?

A: *(Shop name redacted)*.

SS: from where do you buy this big vial?

A: All from Subrata.

MH: Why do you buy the medicine from the doctor not from the shop directly?

A: As I took all these in credit from him and after selling the birds I return the money. We have no principal (money). If I had money I could gain much profit.

MH: What are the types of problems you face with chickens?

A: “Antrik”(watery diarrhoea), “ bloody feces”(if a farm gets older it arises)[coccidiosis], pox etc. But after making this new farm these problems didn’t happen.

MG/SS: Why it so?

A: In new farm the disease is less and the birds also grow well. If you don’t give the 1-2months gap, the problem/disease arises more.

MG: How do you apply this in your farm?

A: There are separate cages. When the new chicks come, they are kept on newspaper in brooding condition. Then at the age of 5 days after giving F1 they are shift to the 2nd cage. We keep the birds on sawdust litter.

MH: How often do you change this litter?

A: When these get dirty, wet, foul smell comes.

SS: How many days apart?

A: Almost 7days apart.

MH: Do you discard the total litter?

A: Yes, we take it out totally and allow it to dry in sunlight. Then you have to filter it. Discarding the waste again it is dried and used again.

MH: What do you with the extract that you filter out of the litter?

A: It’s a good fertilizer, used in the vegetable garden and paddy field.

MH: Do you yourselves use them as fertilizer?

A: Yes, we spread them in the field during cultivation.

MH: At how many days do you sell the broiler?

A: 45days.

MH: And croiler?

A: Around 75days. Otherwise it will not come in size. They take more time.

MH: Do you have different schedule for *croiler*?

A: Yes, same schedule.

SS: Here is only 32 days schedule written. Then?

A: And again we give vitamin, liver tonic double (2nd time).

SS: Which one you give?

A: liver medicine, coccid(?), except these . If any problem seen, medicines are given accordingly.

SS: Which one do you give regularly?

A: Vitamin, livertonic, calcium these 3. And this (showed something), this also we give with feed all time for both birds. This is a vitamin.

MH: Do you give this (Enrofloxacin) again in *croiler*?

A: Yes.

SS: When?

A: After 45days, vitamin and liver tonic are given at afternoon and morning daily one time each. Then from 50-55 days again it is given. (enrofloxacin)

MH: Why do you give this again?

A: 9remain silent)

SS: Did *(NGO name redacted)* tell it?

A: Yes. It is for making strong.

SS: From training?

A: *(Person's name redacted)* told this and *(person's name redacted)* doctor also told.

MH: Do you know what type of the medicine it is (showing the enrofloxacin medicine vial) and why it is used?

A: I don't know properly. (Her son prompts then she tell) It is used when the bird feces become chocolate colour, it is given for 5days.

SS: IT is given in 50-55days to all. Then after if this problem happens?

A: Yes it is used.

MH: What do you do when blackish/chocolate stool occur?

A: Only give this medicine (enrofloxacin)

MH: How many days?

A: For 5days.

MH: Do you give it your own or consult to the doctor?

A: Yes I go and tell this problem and then he gives this. All time we do not go but we take advice of doctor.

SS: Does it happen that you need not to go to the doctor? You yourself give medication and it cured.

A: Yes.

MG/SS: How do you give when you give it yourself?

A: 5ml/100birds.

MG: What happens when there are two birds get ill in the flock?

A: Keep them separately and give them 3drops of the medicine orally to the affected birds.

MG: When occur in less, then how many birds get affected?

A: It not happens generally. If happens in 3-4birds, we give them through dropper catching each.

MG: What about the rest?

A: If seen in them, keep them separate. In others no medication is given. Here are 5 separate cages. We keep them outside or in separate cage.

SS: Keep them inside the farm or outside?

A: Most of the time keeps them outside. As it is virus type disease.

MH: How do you know which birds have chocolate stool?

A: You have to stay there for a time and observe. (Her husband: it’s a very delicate thing, dangerous, by chance if anything happens everything is gone. If it is *croiler* it gives some time but for broiler, no time, die in quick succession)

MH: You have 7 partitioned places in your farm, what are the age group in each?

A: In 1st place: 0-5days, 2nd: 5-15days, 3rd: 15-25days, 4th: up to selling. One part is kept gap. In another one there is croiler (it is kept in that part for whole time), and in 7th place it is place to keep feed, medicine etc.

MH: How often do you buy chicks (broiler)?

A: 15days after selling the birds.

SS: From where?

A: That *(person's name redacted)* brings.

MH: Is it same for the croiler?

SS: How often do you buy *croiler*?

A: 1month. This time I have not buy after 1 months because I have to sanitize the room, I will give a 15days gap, after selling I will sanitize the room with phenyl, bleaching and lime. I will spray that 'vercon-s'.

SS: How long these croilers are kept?

A: Only one and half months.

SS: Before that it was vacant?

A: No, when selling of previous batch was going on, the chicks were in brooding.

MH: The croiler room, is it mixture of all ages?

SS: Is the croiler are in same age?

A: Yes.

SS: without selling those birds, you can't transfer the new croiler chicks. They are in that room from 5 to 75days. So how does it happen?

A: Yes, After 5days brooding, chicks are transferred to croiler room. You have to give at least 3-4 days gap after selling all croiler. I sale the birds mostly at wholesale price. It is advantageous for me. They come and buy 60-70 birds a day, may be at 2-3 rupees less and again they come next day to buy. Then the other birds start to eat less (may be due to stress/fear), so almost 5-7 kg feed is saved, they are small birds so they get fear and it does not affect much on the body weight.

MH: At how much price do you get for the broiler and croiler?

A: 28rupees/broiler chick and 34rupees/croiler chick.

SS: How much price do you get during selling them?

A: Broiler at 90rupees/kg and croiler 125-130 rupees/kg.

SS: Croiler is tasty?

A: Yes.

MH: What do you feed them?

A: (showed the feed packet). This feed.

MH: Do buy it directly or through the doctor?

A: All through the doctor.

MH: Is it the same feed for both croiler and broiler?

A: Same. There are two types (according to size of pellet) of feed. Up to15days age small pellet feed and then large pellet feed.

MH: Do you give anything extra to the feed?

A: Give this powder (Showed the mineral mixture powder).

MH: And rest these you give with water?

A: Yes, with water.

(She again shows the powder [mineral mixture] which she mix with feed. Also shows the spraying machine)

MH: Do you buy all these at credit through the doctor?

A: Yes, all.

SS: What are the problems seen with your goats?

A: Cough and cold. But I can’t tell the medicine name.

SS: Who treats them?

A: *(Person’s name redacted)*.

Sometimes diarrhoeas, fever and ‘golafola’ happen.

SS: Does swollen abdomen occur?

A: Yes, if they take germs it occur.

In case of cough and cold, before going to the doctor, we rub mustard oil on forehead, horn, nose and it reduces. Then we need not to go the doctor. Honey and tulsi (basil) extract also works well.

MG: When was the last time when you gave medicine to the goat?

A: Before getting pregnant we dewormed, then give tetanus injection.

SS: You yourself do it?

A: No, all by doctor. I can do those but I am not confident to treat.

MG: So when did he come last?

A: 3months back.

SS: What happened?

A: No problem was there. He came to give tetanus and vitamin injection. In the middle when cold was here, it started to sneeze and going to the doctor I told that I gave mustard oil and it reduced. Then doctor told “it’s okay, sometimes these needs to be done”.

SS: No other problem?

A: No, they are well now.

MG: Do you ever use this poultry medicine to the goats?

A: sometimes when I feel that the goats are ill, I call the doctor and tell us to give this liquid (Enrofloxacin).

SS: How much and how long do you give them?

A: 2ml once daily for 2 days.

SS: In which condition you give them?

A: When I tell the doctor lets see my goat, what happens with my goat, they don’t want to eat. Then he (doctor) gives this.

SS: Do you ever give them yourself?

A: No, which I don’t know I don’t give.

MG: How long do you give this?

A: 2days, daily once , 2ml.

SS: Does the doctor not tell you to give this 3/5days?

A: No, after 2 days I call him to see if anything needed. Then he sees and say no more needed. If needed more I call him again.

MH: Does it ever happen that the doctor not able to came or he can't solve the problems?

A: No. If *(person's name redacted)* is not present here we go to the *(NGO name redacted)*.

SS: With goat?

A: No, I go and tell. If condition is like that his (*(person's name redacted)*) assistants also come sometimes.

SS: *(Person's name redacted)* is not always present?

A: No, no, only once or twice in a month. There are also people instead of him, the people who have learnt from him, *Sudhansu* and also 2 more people.

MH: Do you call pranibondhu or pranimita?

SS: Does the pranimitra (*(person's name redacted)*) come for treatment?

A: No. She comes sometimes with group, we don't had group, she told to make a group (self help groups).

SS: Does she treat anywhere else?

A: I have not heard her to treat.

Here through groups many things happen. Sometime chicks and kid distribution occur through groups. We deposit monthly 50 rupees and from that they also give loan.

MG: Are you a member of the self help group?

A: Yes.

MH: Did you ever access the block office animal hospital?

A: No, not generally, very less.

SS: Less means when?

A: Once I went when there was mastitis in my cow. Blood was coming out through the teat. We brought the doctor in our car. He came and advised for hot fomentation. He came on the first day and gave injection.

SS: How many days?

A: 3days.

SS: It cured?

A: That sir came one day and then his assistants came from next days. They came and injected medicine and forcefully let down the milk. Then we drew milk and gave hot fomentation.

SS: *(Person's name redacted)* was not there then?

A: He was there but he was then new in practise. He was not expert.

MH: Did you go to him (*(person's name redacted)*) first?

A: No, no.

SS: Did you go to *(NGO name redacted)* then?

A: Then *(person's name redacted)* was not there.

MH: How long ago was that?

A: 4-5years ago.

SS: When the medicines get expired, what do you do with that?

A: No, he (doctor) gives seeing the date (Expiry). This is a 400bird phile (vial/bottle), the 400 birds will sold and the medicine will also get finished.

MG: How much cost do you involve in medicine?

A: Around 1000-1200rupees per 100birds.

(MH took a photo of medicine)

MH: How long you had to wait for coming the block doctor here?

A: Within 1-1.5hour.

MG: Did you ever use these medicines for yourselves?

A: No.

MG: Did you use any human medicine for these animals?

A: No.

MG: Have you heard the term 'antibiotics'?

A: Yes. When you suffer from cough and cold...

MG: What does it do?

A: It's a tablet. In disease we have to take it.

SS: Who gives it?

A: Doctor in the village more. *(PErson's name redacted)*, *(person's name redacted)* (probably quacks). In *(NGO name redacted) (person's name redacted)*.

SS: They do chamber here?

A: No, *(person's name redacted)* is in *(local town name redacted) (NGO name redacted)*, we go there.

SS: For how many days does he give?

A: They prescribe to take it for 3-4 days. But sometimes if we see it has reduced then we don't take it more.

MG: Why do you discontinue?

A: It seems that I am well that's why.

MG: Why did you follow for 5days in poultry and not follow for you?

A: We took training for that, we see after giving 5days the chicks are well, so we give it for 5days. It is our fault that when we seem well we don't take medicine anymore. If you don't complete the course again the problem will arise after few days. If not reduced then we take for 5days.

MG: Do you know what will happen if you give that medicine 2days instead of 5days in poultry?

A: I don't know. I think we are careless, if we follow that, disease don't come easily. If we follow this chart diseases not come easily. In villages who rears, not give vaccine, etc; disease definitely comes there.

MG: How does the human doctor tell her about the course?

SS: Did the human doctor not tell like the 5days course?

A: They tell but we ourselves not follow that. We neglect.

MG: Why do you follow for poultry and not follow for yours?

A: Taking of more medicine is not good, we think this; if I am well then why to take the medicine.

MG: Why it is bad for health?

SS: This bad means what? If you take more medicine what will happen?

A: Many people say this. In older days if children got cough and cold, they did not go to the doctor. They used to treat by honey, basil.

But in today's perspective we have to take medicine.

MG: If poultry gets better in 2days, then what do you do?

A: No, we follow it for 5days.

MG: Do you not think that more medicine will not harm the birds?

A: We are rearing it, if one bird die then there is loss of money. So we follow for 5days.

MH: Did the doctor ever come and check this chart schedule?

A: Yes, he also knows the chart schedule.

SS: Does he come and check whether you are following or not?

A: No, I follow it myself.

MG: Is this pond yours?

A: Yes.

MG: Is there fish?

A: Yes.

MG: How do you look after the pond and fish?

A: We release the small fish in may-june. Fish also get disease. Then we check the water by potash ($KMnO_4$) and lime and have to mix the water.

MG: Do you give any medicine?

A: No.

MG: Do they get any diseases?

A: "Gha" (Patchy ulcer/wound type) seen sometime.

MG: What do you do then?

A: People say to apply salt in that part of that fish but we don't give. The affected fish floats. We took it away and bury in mud. This is also one type of virus.

SS: Don't you take that fish?

A: No. It is also a virus.

MH: Did you ever speak to the doctor?

A: No.

MG: Is there anyone who trained them fish culture?

A: We ourselves release them in pond, give some feed, they grow.

SS: What do you feed the fish?

A: Rice bran. Fish feed is also available here (*(person's name redacted)* chamber).

SS: Do the doctor treat fish?

A: I don't know whether he treats or not. He keeps feed that I know. We ourselves eat the fish that's why we don't use those feed.

MG: What do you think that feed harms the fish?

A:We don't know, we don't give that. But the commercial people (fisherman) fed the fish those and make them grow quicker. They sale the fish in the market. We will eat, why we will give that? Many people give urea in the pond to grow fish. Like the tree plants grow by urea, fish also grow.

SS: Did you ever give?

A: No.

SS: Did you take any training?

A: Yes, long before from *(NGO name redacted)*.

SS: What training did you get?

Small fish has to release in less water, a net has to drag in the water. Taking cow dung, cake in a packet, makes it float in the pond. Fish take that from there and grow.

SS: Did they tell about any medicine?

A: No.

MH: Do you eat these chicken?

A: Yes.

MH: How often?

A: When we want chicken in food we take it from here.

MH: Which one more?

A: More croiler, broiler is less.

MG: What do you think about how the medicine fed to chicken, how it will affect to you?

A: If you take from other place also, they also use medicine. Otherwise it will not grow and disease will come. There is some difference between home food and outside food.

MG: How often do you take chicken from here in a month?

A: Once or twice in a month.

MG: And fish?

A: 3-4 fish everyday. Twice daily.
