# Supplementary material for: “If It Works in People, Why Not Animals?”: A Qualitative Investigation of Antibiotic Use in Smallholder Livestock Settings in Rural West Bengal, India
Source: Antibiotics (Basel). 2021 Nov 23;10(12):1433. doi: 10.3390/antibiotics10121433 (PMC8698124; doi:10.3390/antibiotics10121433)
Supplement: Supplementary file 1 [file antibiotics-10-01433-s001.zip › Supplementary S1_ Interview Transcripts/Site 2/LK27 (site 2).pdf]

Code for Study - 'If it works in people, why not animals?': A qualitative investigation of antibiotic use in smallholder livestock settings in rural West Bengal, India: LK27, Site 2

Date: 17/01/2020

Location: Site 2

Interviewee: Livestock keeper (LK)

Interviewer: Mathew Hennesey (MH)

Transcription: Indrajit Patra (IP)

In Bengali language

MH- Mat Hennessey

LK- livestock keeper

IP- Indrajit Patra

MH- So could you (IP) ask her(LK) what type of animals she keeps here?

LK- Only the cows and poultry.

MH- How many cows ?

LK- Three cows.

MH- What is the use of cows ?

LK- The cow gives one offspring per year . We kept the cows for milk , we drink the milk so no need to purchase milk from market. And from milk we make 2 to 3kg ghee , we ate the ghee throughout the year.

IP-Did you sell the milk?

LK-No.

MH- What they do with those(calf)?

LK-In my life 8 to 10 calf is Bron, all the calf are female. So we kept female calf for milking.

MH- How many people live in this house?

LK- Four people.

MH- Any children?

LK- No , children.Our youngest family member age is 22 year.He study B.A. 3rd year.My granddaughter married with police officer of *(local town name redacted)* police station.

MH- They had any problems with these cows ?

LK- Till today, no problem with these cows, we offer feed with boil water and the salt .Up to this time they have no problem.

MH- What was the last problem they had with this cows?

LK- No no, no problem.

MH- Do they have any problems before with the cows ?

LK- Six months ago there was an incident of cow pox .And 2 calf and 1 cow is died.

IP- What you do when it happened?

LK- We give vitamin injection.

IP- Is the pox happen to all cow?

LK-All 5 cow affect with pox . Out of 5 cow 2 cow dead.

MH- Ok, who gave the vitamin injection ?

LK- *(Person's name redacted)* doctor of *(NGO name redacted)* came and gave the injection .

MH- Is he a veterinary doctor ?

IP- I don't know, she (LK) told that he is a doctor.

MH- What was his name ?

LK- *(Person's name redacted)* his name is *(person's name redacted)*.

MH- And how they contact with him?

LK- He (*(person's name redacted)*) is associated with the *(NGO name redacted)*.

MH-Have they paid for that?

LK- First we paid Rs. 300 then I paid Rs. 400 total Rs. 700.

MH- Seven hundred rupees for only the five cows ?

LK- Yes . In the camp they treat free of cost . But it is problematic to go to camp so we didn't go camp. It's better to call them and treat the animal and paid them.

MH- What was the problem about the camp?

LK- The camp is far away from my house. The camp is sometime occurring in *(local town name redacted)*or some time in football ground which is far away from my house, so that's why i did not go to the camp.

MH- And why do they call Dr *(person's name redacted)* from the *(NGO name redacted)* instead of any other people ?

LK- Because Sudhanshu at the *(NGO name redacted)* is nearby from my house.

MH- Did they go to the BLDO office ?

LK- No no no we did not.

MH- Why not ?

LK- Because this is far away from my house.

MH- Do they give any routine treatment medication to these cows ?

LK- We only provide the feed, no vaccine, no medication. I have no problem with the cows and the milch cows are in the tenth calving. First time it gave 3 to 4kg milk per day and now it gives only 1-1.5 kg milk.

MH- And if there was a problem, said that diarrhoea, fever, what was she do ?

LK- We go to the *(NGO name redacted).*

MH- And how many chickens do they have ?

LK- Five to six. We had sixty birds but all the birds are sold out.

MH- When they sale the bird?

LK- During the winter season we sale the birds.

MH- And why did they sale ?

LK- For one kg body weight we receive one hundred fifty rupees which is high demand and also we have lots of cows, but during the *aila* (a storm), there is problem of feeding, so after the *aila* we sold it. And during the time of *bulbul* (another storm) we kept the cows in a shed made by the *(NGO name redacted).*

MH- Once they have sixty birds, how did they keep them ?

LK- In this shed. The shed having 2 floor. Ground floor and 1 st floor.

MH- And from where did they buy these ?

LK- We produced chicks in our house.

IP-So you have male bird also?

LK- Yes, Now we have 3 male birds out of 6 birds.

MH- They give any medicationto the chicken ?

LK- *(Person's name redacted)* gives them vitamins, deworming and vaccines from 1 vial he vaccinated 100 birds.

IP-Do you have any documents?

LK- We have no documents because of *Bulbul* storm this house is some how destroyed.

MH- Did they have any problems with this chickens ?

LK- Sometime there is loose stools, chaky diarrhea.

MH- What they do when it is happened ?

LK- We go the *(NGO name redacted)*.

MH- Do they take the birds to the *(NGO name redacted)* ?

LK- We call to *(NGO name redacted)* , people of *(NGO name redacted)* came here and treat.We didn't go to *(NGO name redacted)*.

MH- Who would came here to treat?

LK- Dr.*(person's name redacted)*.

IP-Did you know what type of medicine is given?

LK- I don't know, Doctor know the name medicine.

IP- Is the bird cure?

LK- Yes, all time he cured the birds. Sometime we go to the local medicine shop at *(local town name redacted)*and from there we buy the medicines.

MH- What medicine did they buy from here ?

LK- Same medicine like veterinary medicine, we didn't know the medicine name.

MH- Where is the shop ?

LK- The shop is in the *(local town name redacted)*, I didn't know the shop name but the owner of the shop is *(person's name redacted)*.

MH- Is that in the *(local town name redacted)*?

LK- No, in *(local town name redacted)*.

MH- Oh in *(local town name redacted)* okay, and when they treat the birds for their chalky diarrhoea which birds get the treatment ?

LK- We only treat the sick birds not the other birds, sometime when the chick is hatched we take some medicine from the *(NGO name redacted)* but i don't know the medicine name.

MH- Do they want to hatch more birds ?

LK- No because of lots of problem in the house member .

IP-So what you do with the shed of birds?

LK- After the end of the winter seasons we will hatch the thirty eggs.

MH- Who looks after the animals ?

LK- My daughter in law.

MH- And where they go if they become sick ?

LK- We go the hospital near by which is present in the *(local town name redacted).*

MH- Do the doctors in the hospital give advise that how to treat the animals?

LK- No, there are separate departments for human and veterinary so the doctor did not give advice.

MH- That's great, thank you very much.
