# Supplementary material for: “If It Works in People, Why Not Animals?”: A Qualitative Investigation of Antibiotic Use in Smallholder Livestock Settings in Rural West Bengal, India
Source: Antibiotics (Basel). 2021 Nov 23;10(12):1433. doi: 10.3390/antibiotics10121433 (PMC8698124; doi:10.3390/antibiotics10121433)
Supplement: Supplementary file 1 [file antibiotics-10-01433-s001.zip › Supplementary S1_ Interview Transcripts/Site 2/LK28 (site 2).pdf]

Code for Study - 'If it works in people, why not animals?': A qualitative investigation of antibiotic use in smallholder livestock settings in rural West Bengal, India: LK28, Site 2

Date: 14/01/2020

Location: Site 2

Interviewee: Livestock keeper (LK)

Interviewer: Mathew Hennesey (MH)

Transcription: Soumen Samanta (SS)

In Bengali language

MH- Mat Hennessey

LK- livestock keeper

SS- Soumen Samanta

All answer by LK is as 'A'.

(Low sound some inaudible introductory part—

SS: we are looking after why some medicines are not working, where is the problems..)

LK- for 100 birds, 3 bags of litter material is used, and when it got wet it is discarded and not used that again.

MH to SS: Did you explained about the project? Different universities..

SS: I came from the west Bengal veterinary college, and this sir came from a veterinary college of London , one madam who is unable to came here she came from Delhi, and the tagore society doctor is also involved with this work. We are looking after both animal and human aspects, why the medicines are not working, what is the problems, and we also have a team who are taking data from human side.

MH: Can you ask her how many people do live here?

SS: How many people do live in your house?

A: 5 including son and daughter.

SS: What is the source of income?

A: Paddy cultivation, we are having a shop also and these poultry rearing and mushroom production.

MH: What proportion of their income comes from the poultry?

SS: If yours total income is 100 rupees, how much come from poultry?

A: My husband can tell you these things. I don't know.

MH: How long are you keeping these poultry for?

A: 3years.

SS: How many birds are you having now?

A: I had 600 before, but recently almost 350-400 birds died, rest are sold and now having 80-85. This mishap occurs first time for me.

MH: When did it start?

SS: When the mortality started?

A: From the last 10days. Each day 25-30, 35 birds are dying.

SS: What did you do with those dead birds?

A: It was not possible to burry all the dead birds, so we throw it away into the middle of the river.

MH: When do you sell the birds?

SS: At what age do you sell the birds?

A: At 40-45days, not less than 40days when it comes on an average 2kg body weight.

MH: Are all these birds that you have is 40-42days old?

A: Yes.

MH: What signs did you observed since last 10days when mortality started?

SS: What did you see with the birds that were dying?

A: Bloody diarrhoea, ranikhet, 'gum'(may be gumboro), 'hatufola'(swelling of leg joint), blood under wings or at the base of the tail, pox.

MH: Did she say 'ranikhet'? Why did she say that?

SS: Where did you learn the name ‘ranikhet’ from?

A: In ranikhet red feces occur, 1st and 2nd vaccine of ranikhet was done.

SS: Where did you hear ‘ranikhet’?

A: When I took the training from *(NGO name redacted)*. From there I have learned it. Training also take place in our primary school. Also took goat training.

MH to SS: When did she get the training for poultry?

SS: When did you get the poultry training?

A: I took 3 times. First one is 3years back then one and half tears back and last one is 9months back.

MH: What did you learn on the training?

A: Everything. What are the diseases of poultry like ranikhet, coccidia, pox,. What will happen in pox like cough and cold, dull, pneumonia, brooder pneumonia.

SS: Is treatment is also being taught there?

A: Yes.

MH: What type of treatment?

A: Like what to do in brooder pneumonia: have to keep in clear place, you have to feed ‘B90’ medicine for 48hours through water and also to spray it.

SS: What did they taught you in case of pox?

A: In pox: spreading of neem leaves in the litter, sanitization to be done surrounding the room by bleaching, to keep the birds in little warm place.

SS: And what to do in ranikhet?

A: In ranikhet: you have to give electrol(electrolyte) at noon time, to give meriflox (enrofloxacin).

Vaccine given at 5-7days and 19-22days.

SS: Do you give ranikhet vaccine then?

A: No, no. The vaccine is to be given before so that ranikhet does not occur.

SS: So when do you give the vaccine?

A: At 5-7days age.

SS: From where do you take the chicks?

A: *(Shop name redacted)*(a shop of *(local town name redacted)*) and *(NGO name redacted)* model. The supply in *(shop name redacted)*is more.

SS: Which one is preferable?

A: *(shop name redacted)*.

SS: Why?

A: They also fix where I would sell the bird, I need not to worry for selling. I f I take it from *(NGO name redacted)* then you have to pay the transport cost during selling in the market, but in *(shop name redacted)* they come and take the bids, bear the transport cost.

MH: If they take the birds from *(NGO name redacted)* model, how do they pay?

SS: How much you pay when you take birds from *(NGO name redacted)* model?

A: 30/32/40/45 rupees. Last year once I took at 52 rupees, sold it @ 120rupees. Chick cost almost same in both place. But the medicine cost is less in model.

MH: How did they pay for chicks when they take from model?

SS: How you pay?

A: We buy from any one place, like this time from model and next time from *(shop name redacted)*. Most time it is taken from *(shop name redacted)*. By cash we pay.

SS: Is it same for the *(shop name redacted)*also?

A: Yes, we pay the cash. I don't take it on credit.

MH: Is that same in both place?

A: Yes. I don't know whether credit system is there in *(NGO name redacted)*, I always pay cash.

MH: How do you look after the birds from buying of chicks to selling?

A: First I spread the straw then 'choter bosta'(type of jute bag) then newspaper. Then feed, medicine, water is given. First I give one medicine of blue colour (can't tell the name), then electrolyte, then ambiplex and enolyte.

SS: Is it elctrol?

A: No, enolyte is different, ambiplex is different. In this way they are kept for 5-7days under light. Then ranikhet vaccine is given and transferred to the next room. F1 of ranikhet is given on nose as a nasal drop or on eye.

There are 3types of feed according to size. Up to 7days small feed, then middle sized feed up to 30 days then rest days with large size feed is given. During 7-30days period some medicines are given like vitamin A liquid, *ambiplex*(B conciplex), *supercox* powder (Sulphaquinolone), *ebicon*, *meriquin* (enrofloxacin) and again vitamin A liquid. *Meriquin* is given 5ml per 100birds once daily for 5days from 15-20days.

SS: Anything else?

A: And *supercox*.

SS: How it is given?

A: According to water, *Supercox* powder 0.5gram per 10litre water for 3-5days from 10-15days for all birds.

SS: After 30 days what is the schedule?

A: After 30days, *piperazine, livol* (Liver tonic), *VitaA* are given.

SS: Do you repeat Meriquin, Supercox again?

A: No, Meriquin only given so that birds don't get cough and cold.

Within 40days age, vita A is given 3times.

SS: And feed?

A: After 30days large size feed pellet is given.

MH: Where do you know how to do these from?

SS: Where do you know all these from?

A: In training.

MH: Where do you get the medication from?

A: *(NGO name redacted)* or *(shop name redacted)*.

SS: Which one you prefer most?

A: Most of the time *(shop name redacted)*. From where we took the chick, we take the medicine from there.

MH: From where did you get the chick last time?

A: *(shop name redacted)*.

MH: How many chicks did you buy together?

A: 600.

MH: Is it every time 600?

A: No, sometimes 300, sometimes 400 like that.

MH: What make you decide how many to buy?

A: If I have total 600birds, first I buy 300 and after 15 days again 300. Then only it will be in running.

MH: Do you keep all these here?

A: Yes.

MH: When the birds started to getting ill 10days ago what did you do then?

A: First called *(person's name redacted)*(paravet)

MH: What did he do?

A: He came and saw.

SS: What did he give?

A: He gave meriquin @10ml/100birds and told to give in water for all the birds. Then Electrol, in evening water 'cold ruff' @10ml/100birds. All these were given for 5days.

SS: Then what happened?

A: They got more illness.

MH: Which birds were you treating? For all the birds?

SS: All birds were not, did you treat all birds?

A: Yes.

MH: Then what did you do after that 5days?

A: Then again called him. He told that he is not here and he can't do anything now. He told us to go to *(NGO name redacted)*. I went there. They (Not Dr. *(person's name redacted)*) gave one green vial medicine to give for 3days @10ml/100birds. By this also the birds were not getting

better and more illness found. Then I saw that one to two pox lesions arise. I used white vial medicine which *(shop name redacted)*gave @10ml/ 100birds.

(Her husband comes and asks for advice how to prevent such mortality)

MH: Then what happened after that?

A: The birds' death continues. That time the body weight was around 2.5kg and then it started to decrease with increasing mortality.

SS: Did it happen you are taking chicks from here and taking medicine from other place?

A: Most of the time we buy medicine from model as price is less and the doctor is also available. If any bird is ill, I take it to the model and take medicine.

SS: Do you know which doctor is there?

A: 3 doctors are there. (She might be telling the name of paravets, one she told is *(person's name redacted)*). They are available all time.

SS: All time means office time?

A: 10 to 4 o' clock.

MH to SS: What will they do now? The birds are still dying.

SS: So what will you do now?

A: I told the doctor this is the condition, what can be done? Doctor (*(person's name redacted)*) said to sell this phase birds, told *(person's name redacted)* and *(person's name redacted)* (of *(shop name redacted)* shop) both. *(person's name redacted)* told he will send doctor before giving me new chicks. And he will give chicks this time a little bit later.

MH: Is these chicks from *(person's name redacted)*?

SS: Yes.

MH: Did you take advise from other anybody else?

A: No, *(person's name redacted), (person's name redacted)* and *(shop name redacted)*.

MH: What about block hospital?

SS: Did you go to block hospital?

A: No.

MH: Why did you not go there?

A: I don't know whether doctor is present or not.

MH: What about the vet at *(NGO name redacted)*?

SS: Did you went to *(NGO name redacted)* doctor? The doctor who comes once or twice in a month.

A: He is *(person's name name redacted)*. Most of the days, he is not present there. If he is present he gives advice. This time the main problem is that I couldn't take the birds there. He do not stay there, this is the main problem.

SS: Who checked it as he is not present there?

A: His assistants, or he himself also. If he is there he checks it.

Q: Why did you not do that?

SS: Why did you not take the bird there this time?

A: The birds are dying at this selling age not from before. If it happened around 30-35 days I could take it. But if I give medicine at this marketing age the growth will be reduced.

SS: The medication that you take now, is that from this two places?

A: Yes.

MH: The medicine that you showed is that from *(shop name redacted)* cenre?

A: Yes.

SS: But the medicine cost of *(shop name redacted)*is more. So do why you buy from there?

A: If you buy 'loose' (small amount) medicine then its cost is more but if you buy total file then it is same, same.

MH: Did you take information about the vaccination before?

SS: So, what about the vaccination schedule?

A: *Ranikhet* and *gum* vaccines are given.

SS: When do you give them?

A: *Ranikhet* is given at 7 days during transfer of chicks to second room either in nose or in eye. And at 21 days it (2nd) is given in drinking water. And at 15days *gum* vaccine is given. If first ranikhet is not given through nose, it is given in mouth.

SS: From where do you buy the vaccine?

A: *(shop name redacted)*or *(NGO name redacted)*.

SS: Which one is economic?

A: Price is same in both places.

MH: From where did you bring the last vaccine?

A: *(NGO name redacted)*. In this flock the first vaccine (RANIKHET) taken from *(shop name redacted)*, 2nd from *(NGO name redacted)*(GUM). And the 3rd has not been given as it got cough and cold. *(person's name redacted)* told no need for vaccination after seeing.

No, it was given but at 27days.

MH: Did she give the vaccination?

SS: You gave the vaccination?

A: Me, myself. When doctor said for it I gave.

MH: Did the outside poultry also get illness during that time when your birds got ill?

A: No, nobody is having poultry in this season in my area now.

MH: And these chickens? (pointing towards the *deshi* backyard poultry which were roaming in front of his house)

A: These all are vaccinated.

MH: Did they become ill?

A: No.

MH: Does these *deshi* chickens also get medicine with your broilers?

A: Yes. At the same time these birds also get medicines and vaccination.

MH: Did you give the medicine to these birds also during the illness of broilers recently?

A: Yes. As its in the same place. I kept the broiler in roof as the air will pass through high level and will not affect these *deshi* birds at lower level.

MH: Do you give any regular treatments to these birds like the broiler birds above?

A: Yes. Same schedule is followed.

MH: Does it also given twice when the next flock of broiler comes above?

A: Yes.

MH: How old are these *deshi* birds here?

SS: Is it older than above?

A: Yes, these are 9months.

(some repeated words, the main Question is

Q: When one flock is sold out, with new flock it again get the same medicine. So how many times did it (backyard poultry) get the scheduled medicine that you follow for the broilers?

A: 5times.

MH: How do you treat those (backyard chicks)?

A: same treatment continues.

MH: What types of problems do you face with those outside *deshi* chickens?

SS: Do the problems happen with these *deshi* birds?

A: Very rare. Sometimes white diarrhoea, *ranikhet* is seen. But if you give them medicine they get cured easily.

MH: Which medicine?

A: Metaprint tablet.

SS: Who gives this medicine?

A: *(Person's name redacted)*.

SS: How much do you give?

A: It depends on the bird number. Like if 10 birds are there, 1/4th tablet is given. He calculate and tells.

SS: How many days do you continue it?

A: Once daily for 3days.

MH: Where do you get the medicine?

A: Doctor gives or if you go to *(shop name redacted)*.

MH: What do you use these *deshi* chickens for?

A: They lay eggs and also for meat purpose in home.

MH: Do you eat those broiler birds?

A: Very rare.

MH: Why rare?

A: My family don't want to take it. My son and daughter don't like to taste it.

SS: Do you like it?

A: I also don't want to take as I always handle it and always bad smell comes.

SS: Do these deshi birds not get smell?

A: No, these birds are kept free outside all the time but they are always inside.

SS: If you cook it then the smell go out?

A: If you boil in hot water then smell goes out, then also I don't like. The *deshi* birds are 3times more tasty than broiler.

SS: How many are they (*Deshi*)?

A: Chicks 10 and adult also 10.

MH: From where do you buy them?

A: We produce it in home. From eggs the chicks comes out. The broody hen helps in brooding.

MH: What are the other animals that you have?

A: Goats.

SS: How many are they?

A: 5.

SS: What problems do happen with these goats?

A: Fever, diarrhoea, ppr, pox, base of ear swelling. *(Person's name redacted)* make camps.

SS: Where does it happen?

A: In the primary school. It is very near to our house.

SS: How many times does it happen?

A: 2-3times per year.

SS: What treatments are given in camps?

A: Vaccination, vitamin powder is given.

SS: Who treats these goats?

A: *(Person's name redacted)* and also the pranimitra *(person's name redacted)* and *(person's name redacted)*.

SS: Do they (pranimitra) come all the time?

A: They come once or twice in a year but *(person's name redacted)* comes most of the time.

MH: When did the lady (pranimitra) come?

A: *(Person's name redacted)* send them.

MH: What types of problem they come for?

A: Don't come during illness, if illness ocur then *(person's name redacted)* comes. They come to vaccinate or for deworming and giving vitamin injection.

MH: Do you take the goats in the camp or do they come to home?

A: We have to take the goats to camp for vaccination but for other problems or purposes they come to home.

MH: What was the last illness with goats?

A: The goat aborted and was coming in oestrus repeatedly but was not conceiving.

SS: Then?

Q: Then *([person's name redacted)* came and washing was done. Then also it was not solved. Then the body condition of the goat was also detoriating. The surrounding all goats died that time. It happened after the vaccination in ashar (a Bengali month-june month). He doctor who came there told that vaccine could be done to the pregnant goats also 1days before kidding.

When we were under *(person's name redacted)*, he told not to vaccinate 2months of pregnancy.

SS: So who told that (to vaccinate upto1day before)?

A: The doctor who came from *Asansol* through *(NGO name redacted)*.

In every house, the goats were aborted, or the goats died during pregnancy.

SS: What do you do then?

A: *(Person's name redacted)* told he will come and see. But he will not be able to see who has been aborted, either we buried it or thrown it in river, so how he would get to know. Then he has tried his best but he could not solve the problem. He tried 8-9times. So he told me to sell it.

MH: Do you know what medication he gave?

A: No. But that time ppr vaccine was given. I don't know the name the medication. He himself come and treats.

MH: If one of the goat become ill now, whom would you speak to?

A: *(Person's name redacted)* or *(Person's name redacted)*.

MH: Why do you choose to go to him rather than other people?

(she is speaking in a phone call)

A: It is free treatment under him. My goats are tagged and I have a book with photo. (May be she is talking about the AICRP project on goat, as *(person's name redacted)* is in charge of that).

MH: DO you have any other animal here?

A: 2Cows and 2 calves.

SS: Does it giving milk?

A: Yes.

MH: What do you do with the milk?

A: I sell it in our shop.

MH: If the cow get ill like fever and diarrhoea, what would she call then?

SS: What problems occur with the cows?

A: Fever, diarrhoea, golafola, diarrhoea.

SS: Then what do you do?

A: We get them vaccinated by the doctor at the camps at 2and half months age.

Q: If the cows are having diarrhoea or fever, whom would you call to?

A: *(Person's name redacted)* , *(person's name redacted)* or we go there and take the doctor with us to our home. If *(person's name redacted)* is available he also comes here.

SS: Whom do you go first?

A: First I call to *(person's name redacted)*, if is available he comes. If he is not available then go to *(person's name redacted)*.

MH: Why do you go always the paravet *(person's name redacted)* first?

A: He is the local doctor and also available all the time.

MH: How much does he charge?

A: sometimes 50, sometimes 100rupees. During delivery 100 rupees.

MH: Is it including the medication cost?

A: Yes.

SS: Do you take medicine from any other places?

A: If medicine is not available to *(person's name redacted)* then he tells to go where it will be available like to *(local town name redacted)*to buy it. In last September when my one calf got ill, fever neck swelling then I had to buy medicine from *(local town name redacted)* medical shop that *(person's name redacted)* told.

MH: Do you know what medicine was that?

A: No.

(She showed one old medicine packet of Ofloxcin+ornidazole, which she fed to his calf ½ bolus twice daily for 2days). He also gave injections.

MH: Did these medicines work?

A: Yes.

MH: Do you know what is 'antibiotic'?

A: No.

MH: Did you ever use human medicine to treat animal?

A: What doctor *(person's name redacted)* tells that I buy from shop.

MH: Did you ever use animal medicine for yourselves?

A: No. It is high power medicine.

MH: Where do you go when you fall sick?

A: *(local town name redacted)*hospital.

MH: Where do you get the medicine from?

A: What needs to buy, we buy from *(shop name redacted)* pharmacy, *(shop name redacted)* pharmacy or *(shop name redacted)* shop.

MH: Can you ask her if she has any question for us?

LK: My birds are dying, so what can be done?

MH:
