# Supplementary material for: “If It Works in People, Why Not Animals?”: A Qualitative Investigation of Antibiotic Use in Smallholder Livestock Settings in Rural West Bengal, India
Source: Antibiotics (Basel). 2021 Nov 23;10(12):1433. doi: 10.3390/antibiotics10121433 (PMC8698124; doi:10.3390/antibiotics10121433)
Supplement: Supplementary file 1 [file antibiotics-10-01433-s001.zip › Supplementary S1_ Interview Transcripts/Site 2/LK29 (site 2).pdf]

Code for Study - ‘If it works in people, why not animals?’: A qualitative investigation of antibiotic use in smallholder livestock settings in rural West Bengal, India: LK29, Site 2

Date: 17/01/2020

Location: Site 2

Interviewee: Livestock keeper (LK)

Interviewer: Mathew Hennesey (MH)

Transcription: Indrajit Patra (IP)

In Bengali language

MH- Mat Hennessey

LK- livestock keeper

IP- Indrajit Patra

MH- Thank him(LK) for giving interview.

LK-Ok no problems.

MH- Very a beautiful place it is And what type of animals does he keep here ?

LK- goats , poultry, cat & dogs

MH- How many goat?

LK- 16

IP- How many poultry?

LK- 60 poultry.

IP- Poultry.

MH- How many?

IP- 60

MH- What type of they(Poultry)? Kuku, oh. Can I take a photo with you?

LK- Yes

MH-Kub Valo(Very good) and What types of chicken ?

LK- only the *desi* birds.

IP-How many desi birds?

LK- All bird are desi.

MH-What other type of animals ?

LK- Cat and dog.

IP-How many cats?

LK-4 cats

MH- And the dogs?

LK-5 dogs.

MH- How many people live here ?

LK- Five people. Me my wife my children and my parents lives here.

MH- How many adult and any children ?

LK- One children .

MH- Why do they use goat ?

LK- For the earning money,they have no land so they depend upon livestock .

MH- How do they earn money from goats ?

LK- The goats give birth and two to four time in a year and they sale the kids.After 1year we sell the goat at Rs. 4000 to 5000.

IP-Is seller man came in the house or you have to go to market?

LK- Saler man came into the house and they take the goats with them.

IP- What is the price of the goat?

LK- In one year they attending the 10kg body weight and the price is 4,000 rupees.

MH- Why do they keep the *murgi* ?

LK- For the eggs and selling of chicks.

IP-And meat?

LK- Actually I kept this birds for meat and hatch the eggs. And I sell the chicks.

MH- Did they sell the chicks?

IP- Yes.

MH- Where did they sale the chicks ?

LK- We sale it in the market.

IP- Would you go to market with the chicks?

LK- Yes.

MH-what is the price for one chick ?

LK- one hundred fifty per chicks.

MH- How old they(chicks) when he (LK)sold them?

LK-1 month of age.

MH-Did they sell eggs?

LK- No, We did not sale the eggs, we hatch the eggs and then we sell it into the market.

MH- Do they use adult birds for meat ?

LK- Yes we sale the adult birds.

MH- How old they are when sale them ?

LK- In one year near about 13 months.

MH- What is the weight ?

LK- Three to four kg.

MH- what is the price of adult bird ?

LK- one thousand to two thousand rupees for each bird.

MH- Do they eat chicken?

LK- Yes, we eat.

MH- How often the ate chicken?

LK-We love this birds because we rearing the so we did not ate it. We purchased the chicken from market for eat.

MH- What type of chicken they buy from the market ?

LK- *Desi* chicken .

MH- So why they do not eat their reared chicken meat and why they buy from market ?

LK- Because we love the birds.

MH- That's nice Haha (smiles) very sweet,and how often they buy chicken to eat ?

LK- we are poor land less people so we ate chicken once per two months.

MH- Is it *desi* **or assel** ?

LK- Tamil.

MH-Oh ,It is big.

IP-What is the age this birds?

LK- 1.5 years age.

MH- What is its use ?

LK- Male using for the breeding purpose.

MH-Okay, good **one'** *khub valo khub* **valo'** haha (smiles) how many male chicken ?

LK- Five male.

MH- And they give any type of medication to the chicken routinely?

LK- For the chalky diarrhea or green diarrhea we gives the gentamicin injection.

MH- Gentamicin injection ,and from where they get gentamicin injection?

LK-From *(local town name redacted)*Market.

IP- What is the name of the shop?

LK- From the "*Mamoni* medical shop"because of unavailability of medicine in govt. office BLDO office .

MH- How do they know to give gentamicin injection during chalky diarrhea ?

LK- In the BLDO office some assistant told them.

MH- When did they go to the BLDO office ?

LK- When the animals become too sick we go to the BLDO office and consult.

MH- When they last went to the BLDO office ?

LK- One week ago.

MH- And when they went to the BLDO office ,which person is present to speak there ?

LK- Usually we talk there with doctor, I have forget the name.

IP-Is he V.O.(Veterinary officer)?

LK-Yes

MH- Do they give any other drug to the chicken ?

LK- Calcium,supercox and vitamin B-capsule,vit E.Viminar also some time given.

MH- They have any other medicine bottle,can he show ?

LK- Yes we have medicine bottle.

MH- What to they use this medicine for ?

LK- For good digestion .

MH- When do they give to this birds ?

LK- In the time of indigestion due to liver problems we give it.

IP- You give it routinely or in disease condition.

LK-In disease condition only we give it.

IP- What did you mean by indigestion?

LK- Indigestion mean When birds food stored in the crop and the bird having weakness then we give this one

MH- And what about this one ?

LK- This is also for indigestion. Poultry having the main problems of diarrhea.

IP- When you give this(Vitamin)?

LK- This is vitamin. we give 2 to 3 drop with drinking water in healthy birds.

IP-In which age you gives it(Vitamin)?

LK- It can be give in any age.After giving the dewormer.

MH- Why did they do that ?

LK- When the bird is weak to cure the bird we do it.

MH- From where they get this medicine?

LK- From *(shop name redacted)* medical shop in *(local town name redacted)*.After consulting the doctor we purchased this medicine.

MH- How do they use this(medicine) now?

LK- By mixing with the water or hot rice.

MH-Why do they use this one ?

LK- Because of the weakness of animal.

MH- Can you give an example the ‘animal is weak’,what do they mean by that ?

LK- When chalky diarrhea is there and green diarrhea and also cold and cough condition.

MH- for How many days they give this ?

LK- Actually it have to given for 3 days.

MH-When was the last time they use this ?

LK- one week ago.

MH- Why was that ?

LK- Due to the change of the weather and when the birds died so for the precaution and prevention we give this.

MH- Which birds they give it to ?

LK- All birds as preventive measure.

MH- How many bird died ?

LK- No no no bird is not died .

MH- So during the weather change any bird died ?

LK- Bird are died previously,they seen that by their own express so due to the preventive measure they give it.

MH- So no bird this time ?

LK-Yes no bird died actually gentamicin injection having the good effect.

MH- Did they have gentamicin injection here?

LK-yes.

MH- Aha haa (smiles) okay , and how do they give this to the birds ?

LK- 0.5ml injection I/M, we give in thigh muscle.

MH- How many times ?

LK- During diarrhea we give the once daily for three days.

MH- For three days , and to which birds they give this ?

LK- No we give it to only sick bird.

MH- Where did they keep the sick bird?

LK- The bird kept in Quarantine .

MH- Where they keep in quarantine ?

LK- We having the another house.

MH- So if today he noticed one bird is sick what would they do ?

LK- Immediately the bird is sick we give that injection ,the gentamicin injection.

IP- Are you consulting with doctor?

LK- We are not consulting with doctor, we have reserved of gentamicin injection.

MH- Okay, what they do if they bird is not better ?

LK- When the gentamicin injection is not working the bird is died.It is the last suggestion.

MH- So what is the first suggestion ?

LK- Gentamicin is first and last suggestion and we having another one.

MH- Aha ha (smiles) what ?

LK- When there is chalky diarrhea greenish diarrhea ,and bronchitis we use it(medicine).

MH- Where did they get this from ?

LK- From the the same market.

MH-When would they use this Enrofloxacin ?

LK-In chalky diarrhea , green diarrhea and bronchitis etc.

MH- How you decided to gives Enrofloxacin or gentamicin in case of Chaky diarrhea?

LK- When the gentamicin is not working we give the enrofloxacin.

MH- Why do they use the gentamicin first and then the enrofloxacin?

LK- Actually Frist we observe the condition of the disease.If the birds is very much weak we did not use Enrofloxacin.

MH-Why they do that?

LK- Enrofloxacin is very much hard , bird may died due to high temperature.

MH- Okay, Do they know what type of medication these are ?

LK- Yes , this are the antibiotics.

MH-Have they heard about antibiotic resistance ?

LK- No , we don't know this.After givin such type of medicine (antibiotics) there is no problems.

MH- Ok fine,Do they give any vaccines to the birds ?

LK- From five to six days Ranikhet vaccine then 14th days.

MH- What did they give ?

LK- We having the literature according to literature we gives vaccine.

MH- Bengali literature ?

LK- Yes.

MH- Where do they get this from ?

LK- From market or Got from one training.

MH- Okay,wow, and from where do they get this ?

LK- One cooperative is there Rupayan. they give this book.

MH- This is the vaccination schedule ?

LK- Yes.

MH- So what does it say ?

IP- In five to seven days F 1 strain ranikhet one dose give in the nose or eye.In 12 to 14 days gumboro in the eye and in 15 to 20 days deworming and they(LK) give this also,in 25 to 30 days pegion pox

MH- Those are all antibiotics ?

LK- All are dewormer and vaccination.

MH- They are having lots of knowledge ?

LK- Yes.

MH- You read this book ?

LK- Yes. This medicine we use it for pox.

MH- Okay,from where they get this ?

LK- This is human medicine it can be given to all animals.

IP-From where you(LK) get this?

LK-This is homeopathy medicine we get it from *(local town name redacted)* market.

MH- Does doctor give homeopathic advise as well looking after animals?

LK- Yes .

MH- Okay,so he gives advise ?

LK- yes .

MH- Do they buy any antibiotic from homeopathic ?

LK- Yes, this medicine nux mother.

MH- For What purpose do they use this ?

LK- For GI tract problem, liver problems it can be given to cow goats and poultry also.

MH- How did they know the birds having liver conditions?

LK- From the book.

MH- Okay , good. its very impressive. all of the information they have .

LK- We have the homeopathic book .

MH- Okay Okay (smiles), That's really good, can you ask why do they go to the veterinary officer at the block BLDO and why do the choice to him and not the other doctor ?

LK- They (People of BLDO office) are giving medicine with free of cost ,or low cost with discount and one example that local doctor gives gentamicin for Rs. 40 but if we purchased the gentamicin from *(local town name redacted)* market it only Rs. 9.

MH- So 9 rupees for one vial is that correct ?

LK- Yes yes.

MH- Can you please explain who is Quack doctor ?

LK- *(Person's name redacted), (person's name redacted), (person's name redacted).*

MH- Do they use quack doctor for any other reason ?

LK- We call paravet for the vaccine .

MH- For which animal ?

LK- Goats .

MH- Just for the goats ?

LK- Yes and sometime for the saline .

MH-Saline,what for ?

LK- When there is fever we first give calpol for fever and then saline and for loose diarrhea.Digin also give for loose stool.

MH- How do they give the saline ?

LK- Saline sets bring with the paravet and they(Paravet) give saline .

MH- Where they give it to the animal ?

LK- In house

MH- Where the the animal they give it ?

LK- In skin(s/c) and in the jugular vein.(i/v).

MH- Okay ,Why do they call the paravet in this situation instead of the veterinary officer ?

LK- Because this is difficult to go the VO office with the animals .

MH- Okay and what about the pranibandhu ?

LK- We did not know.

IP- Was pranibandhu came here?

LK- Yes they having the duty to visit the house.they gives vaccine, dewormer.

MH- When was the last time the pranibandhu came here ?

LK- One month ago.

MH-And What for?

LK- From the BLDO office pranibandhu are send for normal visit.

MH-And did they pay for the pranibandhu for the visit ?

LK- They came for the normal visit and they give vaccine and we have to pay for one rupees for one bird. As we know how vaccine so pranibandhu did not vaccinate our bird.

MH-How much rupees ?

LK- One rupees/bird.

MH- And how much for goat ?

LK- No rupees for the vaccination .

MH- Okay,because this is the under the project ?

LK- yes yes.

MH- And if the paravet comes with the the calpol and saline how much its cost ?

LK- 100 to 150 sometime also come for kidding and charge is same and we also treat we neighbour houses.

MH-Can he explain more about that ?

LK- When there is a problem in kidding during delivery and some poultry bronchitis case we also treat and we provide one liquid .

MH- One liquid to treat the bronchitis ,is it antibiotic ?

LK- First give one liquid and when if it not work then we give other.

MH- And this is to the neighbour ?

LK- Yes and one liquid for all.

MH-Okay ,and where they get it from ?

LK- From *(local town name redacted)* market.

MH- How many neighbour houses they treat?

LK- 10 to 12 houses.

MH- Did they take any charge ?

LK- No.

MH- Welcome its nice , “**khuub valo haha**” (smiles) wow.

LK-We didn't change any money.

MH- Do they give routine antibiotic to the goat like chicken like tetracycline ?

LK- No no antibiotic we give only the vaccine tetanus before two month of kidding. The medicine we get from the govt office not work properly so we bought it from medical shop

MH-Can he give one example which medicine did not work properly ?

LK- We did not know any name.

MH- Can you ask why they give tetracycline when there is change in wheather?

LK-We give it all time.

MH- What they mean by there is change in weather ?

LK-Weather change means certain change in cold,cold to hot summer, winter.

MH- How many times its happened in the year ?

LK- During the rainy time, summer and winter three to four times in one year.

MH- What happens when they don’t do it ?

LK- The bird are died.

MH-Where did they go for health care if people are sick in the house.

LK-Frist we treat with homeopathy then we go to *(local town name redacted)* hospital.

MH-Do they dispensing the medicine to the neighbors.

LK-No(told by male farmers), Yes(told by his wife).

MH-Okay and any one of the hospital give advise about the animal medicine ?

LK- No.

MH- Any one of *(local town name redacted)* hospital give advise about looking after animals ?

LK- No.

MH-Thats great thank you ,can I take a photo of this book ?

LK- Yes.

MH- Ok thank you.(smiles)
