# Supplementary material for: “If It Works in People, Why Not Animals?”: A Qualitative Investigation of Antibiotic Use in Smallholder Livestock Settings in Rural West Bengal, India
Source: Antibiotics (Basel). 2021 Nov 23;10(12):1433. doi: 10.3390/antibiotics10121433 (PMC8698124; doi:10.3390/antibiotics10121433)
Supplement: Supplementary file 1 [file antibiotics-10-01433-s001.zip › Supplementary S1_ Interview Transcripts/Site 2/LK30 (site 2).pdf]

Code for Study - 'If it works in people, why not animals?': A qualitative investigation of antibiotic use in smallholder livestock settings in rural West Bengal, India: LK30, Site 2

Date: 17/01/2020

Location: Site 2

Interviewee: Livestock keeper (LK)

Interviewer: Mathew Hennesey (MH)

Transcription: Indrajit Patra (IP)

In Bengali language

MH- Mat Hennessey

LK- livestock keeper

IP- Indrajit Patra

MH- You(IP) can explain little bit more about the project.

IP-This is about the one health. This man(MH) came from londan and one madam ,she came from Delhi andI(IP) came from Kolkata , we are doing interviews about the livestock.I saw you(LK) rearing the sheep so we came here for your interview.

MH-How many people live in this house ?

LK- 4 adults, my son is 18 year old in this year he will appear for H.S.(Higher secondary) examination

MH- No children ?

LK- No children .

MH- What is the source of income ?

LK- My husband working as labour in cultivation .

MH- Any other source of income ?

LK- From the sheep, goat and cows.

MH- What proportion comes from livestock ?

LK- Here labor get Rs. 300 per day. But labor can't get work everyday.We get more money from labor less from animal. In some time when we sell eggs we getRs. 300 to Rs. 400 per months.When in any month we sell the bird in that particular month income from animal is more , If in any month we purchased chicks in that month , income from animal is more.

MH- Okay what type of animals keep here ?

LK- 1 cow, sheep 3 and poultry11 adults and 15 chicks .

MH- What type of poultry are there ?

LK- Mix of *desi* and kuroiler .3 kuroiler and other 23 are desi .

MH- okay, What they use the cow for?

LK- From cow we get calf and we drink the milk.

IP-Are you sell milk?

LK-Yes.

IP- What is the price of the milk?

LK- Rs. 26 per litter.

IP- Is miller man came here?

LK- Yes.From cow we also get cow dung.

MH- How often the milker come ?

LK- Every day in the morning. In the lactation phase 2 times milking is done ,first time we sold the milk to the milker man and second time in the afternoon we kept for comsumption.

MH- So how much do they keep each day ?

LK- 2 to 2.5 kg /day, previously we have another cow but that cow gives small amount of milk so we sell that and purchase the new cow.

MH- Do they do any routine treatments ?

LK- Routinely we used the vitamins and deworming .

MH- From Where did they get this?

LK- We get that from the model farm .

IP- Are you pay for that?

LK- Yes.

IP-Have you any documents?

LK-No , yesterday I trow out all the documents.

MH- Where is the moldel farm situated?

LK- In the *(NGO name redacted)* .

MH- Okay when was the last time the cow became sick ?

LK- last time 6 months ago before the Durga puja there is a problem of bottle jaw .*(Person's name redacted)* from *(NGO name redacted)* came and treat the cow. The medicine is given in the paper.We didn't know the name of the medicine.

IP- Is the animals cure?

LK- Yes.h

IP- What type of medicine he (*(person's name redacted)*) gives?

LK- He gives Tablet and liquid.

MH- For how many days ?

LK- For 7 days .

MH- Did the cow get better ?

LK- Yes

MH- And why did they prefer *(person's name redacted)***?**

LK- *(Person's name redacted)*work in *(NGO name redacted)* and his house in near from my house.

IP- Why you did not go to V.O. office (BLDO office)

LK- When the animal is not cure that time we go to V.O. office.

IP- Are there any case that you have to go to BLDO office?

LK- No, I don't need to go BLDO office.If we give more money to *(NGO name redacted)* worker then they will came in the house and treat the animal. *(Person's name redacted)* , *(person's name redacted)* and Other 4 to 5 people also stay in *(NGO name redacted)*.In every month we go there(*(NGO name redacted)*) and bring vitamin and dewormer.

MH- Who is *(person's name redacted)?*

LK- *(PErson's name redacted)*work in *(NGO name redacted)*.

MH- How much the cost of medicine ?

LK- 700 rupees for 7 days .And for vitamin Rs. 200.

IP- How much for dewormer?

LK-For dewormer Rs. 10 to 12, dewormer they give according to body weight.It will be easier to understand the cost of the medicine if I have the document.But yesterday I throw all the documents in water.Mostly we go there(*(NGO name redacted)*) for poultry medicine.They (People of *(NGO name redacted)*) have written about the medicine in there own khata for keeping records.

MH- Ohh its okay and what is other sheep for ?

LK- For sale purpose.

MH- For what ?

LK- For the money. After the 6 months of age, the animal become adult.We get 3-4 thousands rupees by selling them.

IP- What is the weight of sheep during the selling time?

LK- If sheep get good feed then it will often near about 5 to 7 kg body weight.

IP-Where you sell the sheep?

LK- Seller man came in the house and purchase the sheep.

MH- Do they do any routine treatment to the sheep ?

LK- We done the vaccine two time per year.

IP- What about dewormer?

LK-Ya we also offer dewormer and vitamin to our sheep.

MH- Have they ever done any vaccination of the animals?

LK- Somebody came and did the vaccination to all animals .

IP- In Which age group animal they give vaccine?

LK- They give vaccine to all animal only except the pregnant animals.

MH- Does she know what the vaccination is ?

LK- No , I don't know.

MH- And who had come to do the vaccination ?

LK- *Pranimitra* came to vaccinate the animals.

MH- Did they take any charge for that ?

LK- 2 rupees per sheep**.**

MH- Do they give any other treatments for sheep ?

LK- No,but once the dog bite to the sheep that time we go to *(NGO name redacted)* with sheep and they give vaccine.

IP- What is the cost that time?

LK- That time cost is little bit high, near about Rs. 150.

MH- any other treatments ?

LK- Some times there is a fever ,colic and we go to the *(NGO name redacted)* with the sheep and they treated the animals.

MH- Do they get treatments from anyother else ?

LK- No.Here animal is cure thatwhy no need to go any other else.

MH- And why do they keep poultry bird**?**

LK- We keep the birds for egg and meat purpose. We sale the meats.

IP- Are you sell the eggs?

LK- Yes , when 8 to 10 birds lay eggs that time we sell the eggs.

MH- How often they eat the meat ?

LK- 2-3 times in a month**.**

MH- Do they give routine treatments to the birds ?

LK- There is problem of chalky diarrhea , and loose stool and we go to the tagore

with the birds and bring the medicine.

MH- which of the bird get medicine?

LK- All the birds .

IP- Are the sick birds are quarantine?

LK- If the bird is very sick then we quarantine the sick birds.

MH- Why did not the prefer *pranibondhu*?

LK- Because *pranibandhu* did not come and we go to the *(NGO name redacted)* because its nearby her house .

MH- Do the mobile vet camp service nearby ?

LK- No.

MH- And where do they go when any other house member get sick ?

LK- We first go to the *(local town name redacted)* hospital and if not cured we go to the kolkata PG(sskm) hospital .

MH- Why do they keep sheep instead of goats ?

LK- Goat are always dead.

MH- When they have goats before ?

LK- 17 to 18 years ago when I have small children. I will try for goat rearing after few days because in goats there is lots of money.

MH- Ohh long time ago ,she has any medication for the animals we can look out ?

LK- No.I have clean my room yesterday because some people come in my house for marriage of my girl.

MH- Does,she knows about antibiotics ?

LK- I heard about the antibiotics but I don't know the name of the antibiotics.

MH- Does she gives any human medicine to the animals ?

LK- No.

MH- Why would they do that ?

LK- Because doctor is not prescribe to give human medicine to animals.

MH- What do they feed to the animals ?

LK- Grasses, Straw and water hyacinth.

MH- Do they buy any extra concentrates ?

LK- Mash feed for poultry .

MH-, When they do that ?

LK- One time in everyday we mix the mash with wheat and rice .

MH- Why do they give mixed with rice and wheat ?

LK- To reduce the volume of mash .

MH- Why do they give mash to the poultry ?

LK- For higher growth rate.

MH- That’s great,*dhanyabad* ,do they have few question about it ?

LK-We need emergency services for human here,It is problematic to go to Canning for treat at night.
