# Supplementary material for: “If It Works in People, Why Not Animals?”: A Qualitative Investigation of Antibiotic Use in Smallholder Livestock Settings in Rural West Bengal, India
Source: Antibiotics (Basel). 2021 Nov 23;10(12):1433. doi: 10.3390/antibiotics10121433 (PMC8698124; doi:10.3390/antibiotics10121433)
Supplement: Supplementary file 1 [file antibiotics-10-01433-s001.zip › Supplementary S1_ Interview Transcripts/Site 2/LK31 (site 2).pdf]

Code for Study - 'If it works in people, why not animals?': A qualitative investigation of antibiotic use in smallholder livestock settings in rural West Bengal, India: LK31, Site 2

Date: 17/01/2020

Location: Site 2

Interviewee: Livestock keeper (LK)

Interviewer: Mathew Hennesey (MH)

Transcription: Indrajit Patra (IP)

In Bengali language

MH- Mat Hennessey

LK- livestock keeper

IP- Indrajit Patra

MH- So you(IP) can thank him for talking to us(MH&IP).

LK-Welcome.

MH-how many do people live here ?

LK- Now 2 people husband and wife.

MH- And what type of animal he have ?

LK-Mainly we had poultry in that shed , the shed is 50ft by 10ft.

IP-How many poultry you have?

LK- We had 500 chicken.

IP- So now how many poultry you have?

LK- Now only 18 are present.

IP- When you have 500 chicken?

LK- 5 months ago.

MH- What happened to that 500 chicken?

LK- We sold the chicken.

MH-What is the price of the chicken?

LK-Rs. 125 per kg.

IP- What type of chicken you have?

LK- Kurolier birds.

IP- Are all the birds kurolier?

LK- Yes, all birds are kurolier.

IP- What type of chicken you have in this 18 chicken?

LK-In 18 chicken 8 chicken are Vanaraja.

IP- And other?

LK- Other 10 birds are desi.

MH- And this are?

LK-Desi .(Local birds)

IP-And this two cow?

LK- We are not owner of this two bi

MH- Where did he get the kuroiler chicks?

LK- Kurolier chicks came from Kolkata ,we purchased chicks from here.

IP-Where did you purchase kurolier chicks?

LK-I purchase the kurolier chicks from someone.

IP-Is that *(person's name redacted)*?

LK-No ,not from *(person's name redacted)*, we purchased chicks from our model farm *(NGO name redacted)*.

MH- Why does he keep the kuroiler ?

LK- We kept the Kurolier for business.

IP-Did you get any eggs?

LK- No, it is only for the meat purpose.

IP- Are you selling the birds in market?

LK- Ya, some time people came our house to purchase the birds and some time we go to market.

MH- How long he is being raised kuroiler?

LK- 3 month required for maturity of Kurolier, around 7 year ago I have start the Kurolier farming.

MH- How did he learn that how to look after the kuroiler ?

LK- We know it by ourselves.

IP- Are you doing any training?

LK-We did training from *(NGO name redacted)*.

IP- What is the time period of the training?

LK-3 month training. My wife is ill so we stop the farming.

IP-When you start the farming again?

LK-No chance to restart the farming because my wife is ill and I work as labor in build construction. Mainly my wife look after the birds.

MH- Who did the training ?

LK- *(NGO name redacted)* gave the training. I didn't know the name who gave the training. My wife did the training.

MH- So he will not anymore raise the kuroiler ?

LK- No .

MH- And does he know what type of treatments or medication should give to the kuroiler ?

LK- My wife can tell about the medication and treatment to us. People of *(NGO name redacted)* came here and they treat the birds and gave medicine.

IP-Have you any documents about the treatment?

LK- May be but I don't know. My wife know about this. But now she goes to BDO(Block development officer) office.

MH-What office?

LK-BDO office behind the BLDO office.

MH- Why does she some go BDO office or some time go to *(NGO name redacted)*?

IP- Why does she some time go BLDO office or some time go to *(NGO name redacted)*?(IP ask about BLDO office instead of BDO office because BLDO office treat the animals and BDO office is for the administration work)

LK- We didn't go to BLDO office we mainly go to *(NGO name redacted)*

MH-Why?

IP- She going to BDO office not BLDO office.

MH-What is BD(BDO office)?

IP- Block development officer office.

MH-Sorry?

LK- She going for personal work.

MH- Who goes to the BDO ?

LK- My wife .

MH-Why his wife goes to the bdo office?

LK- Personal work not related to animals.

MH- That her job?

LK-No she going to BDO for applying some money that are allotted by the government for aged people.

MH- So she works there ?

LK- No , she is housewife .

MH- Does he know what type of treatment should give to the kuroiler ?

LK-What did you mean by treatment?

IP- Treatment mean type medication during illness or any type of vaccination.

LK- *(NGO name redacted)* people came in the house and they treat .

IP- Are you going *(NGO name redacted)* with the birds?

LK- No, *(NGO name redacted)* is near by of my house and we have good relationship with *(NGO name redacted)* so they came here and they treat the animals.

IP- Are you call them?

LK- yes.

MH- And is it same for these poultry ?

LK- For these chicken we go *(NGO name redacted)* model farm and bring medicine.

IP-What type of medicine you bring?

LK- Mainly dewormer

IP-Did you bring vaccine?

LK- yes

IP- What type of medicine you bring for chalky diarrhea ?

LK- We go to *(NGO name redacted)* and bring medicine. My can told the name of the medicine, I don't know the name of the medicine.I am not remain in the house because I had to go for construction work.

MH-What type of medicine for chaky diarrhea ?

IP- Is she(LK wife) liquid or solid medicine?

LK-She (LK wife) always bring liquid medicine but don't know the name.

IP- Have you any prescription?

LK- Yes we have prescription but it will better if my wife may present here.My girl stay near to my house. Can I call her(LK girl)

IP- Yes.

MH- So Who is the main person for looking after the poultry?

LK- My wife.

MH- What type of things does she doing with animals ?

LK- In 0 to 5 days we give one vaccine to the kuroiler and same vaccine was given in 21 days in eye or nose and in 2 months age we gave another vaccine below the wings.

IP- Are you using any antibiotics?

LK- From the first day they take medicine.

IP- What type of medicine?

LK-All the medicine are provided through water mainly salt water is provided.

MH- Any other medication given ?

LK- If there is disease then we provide medicine.

IP-What type of disease?

LK-After fews when the poultry grow up then some time bloody diarrhea occurred.

MH- What medication they give to the bloody diarrhea ?

LK- I don't remember.

IP-Is the medicine solid or liquid?

LK- One powder and one liquid medicine is given to the birds.

IP- From where you bring the medicine?

LK- From the model farm of *(NGO name redacted).*

IP- Are you going to *(NGO name redacted)* with the birds?

LK- Yes, we going with the bird. After showing the bird they give medicine.

MH- Do they go anywhere else for medicine ?

LK- From *(local town name redacted)*veterinary hospital.

IP-Where from *(local town name redacted),* is that from BLDO office?

LK- Yes , from BLDO office they give medicine free of cost.

MH- When do they go to the BLDO office ?

LK- We go there some time.

MH- Can she give any examples when she go to BLDO office?

LK-Mainly we go to *(NGO name redacted)*, but when we didn't get medicine or vaccine from *(NGO name redacted)* then we go to the BLDO office.

MH- What does she mean that not getting medicine from *(NGO name redacted)*?

LK-That mean medicine is not available in *(NGO name redacted)*

MH-Is it only for vaccination or any other reason?

LK-Not only for vaccine but also when we didn't get medicine from *(NGO name redacted).*

MH-When was the last time they went to the bldo office ?

LK- 3 to 4 monthds ago.

MH- For what reason ?

LK- For vaccination of the chicks.

MH- What they use cows for?

LK-No we didn't have any cow.We have cow previously.

MH-Who is the owner of this cows?

LK-Our neighbor is the owner of this two cows.

MH-Oh so here just poultry ,Do they talk with *pranibandhu* or *pranimitra* ?

LK- Pranibandhu are some time came here.

IP-Why they came here?

LK-They mainly came in model farm, in problems if we call them they came here.Some time they came to tell us about the meeting.

IP-Are they came for treatment also?

LK- If we call them in cow or poultry problem then also they come here.

MH- How they decide to choose whether go to *(NGO name redacted)* or call Pranibandhu?

LK-First we go to *(NGO name redacted),* if anyone is not available in *(NGO name redacted)* then we call Pranibandhu.

MH- When was the last time *pranibandhu* came here ?

LK- long time ago near about 6 months. Mainly we go to *(NGO name redacted).*

MH- When the *murgi* with chalky diarrhea and they go to the *(NGO name redacted)* .how much the cost is ?

LK- Total Rs.50, This my wife .

MH- And if they call the *pranibandhu* ?

LK- When Pranibandhu came in this house they take fees.

IP- How much cost when Pranibandhu came 6 months?

LK- Near about Rs. 100 to 150.

MH-Oh it expensive, When they went to the bldo office ?

LK- In *(local town name redacted),* form making a ticket they take Rs. 5 .

MH- Why didn't they go to the bldo office first ?

LK- The car fare is more to go to the bldo office. And *(NGO name redacted)* is near to my house.

MH- How much cost is required to go to the bldo office ?

LK- Rs. 15 for going and Rs. 15 for returning total Rs.30 .And if we go there more time is also required.

MH- How much time does the car or bus take ?

LK- BLDO office having different process. So it will take 6 hour.

MH-6hour ,wow ok Why does it take long?

LK-Large people came here so we have to wait there.

MH- Does any mobile vet camp nearby here

LK- Yes, The camp occurred inside the *(NGO name redacted)*.

IP-How much distances from here to camp?

LK-Near by place, it will take 3 minutes, 500 meters.

MH- have they used the mobile vet service ?

LK- Only we go to camp for vaccination of birds.

MH- When was the last time they use ?

LK- long time,4 month ago.

MH- How much cost ?

LK- Free of cost.

MH- What they feed the poultry ?

LK- Wheat dust , mash feed and rice and kitchen byproduct.

MH- What do they use this birds for ?

LK- Those are RIR birds they lay eggs.

IP-Others?

LK- Others are male , they use for playing game(Fighting perpus).

MH- Is are Vanaraja or RIR?

LK- No this are RIR.

MH-This all are crockrel or male?

LK-Yes, this are male.

MH- How many eggs do the birds lay per day ?

LK- They lay eggs upto 80 days.

IP-When they start to lay eggs?

LK-In 6 months old.

MH- What they do with this egg ?

LK- Frist we ate if extra eggs is produced then we sell the eggs in market.

IP- Are you going to market or sell men came here for purchase eggs?

LK-No , We have to go market for selling the eggs.

MH- And where they get if anyother people of this house get sick ?

LK- first we go the *(NGO name redacted)* and then *(local town name redacted)* hospital.

MH- Whether they buy any medicine from the shop ?

LK- From the Shop situated in chandimore .

IP- What is the name of the shop?

LK-*(shop's name redacted)* enterprise.

MH- Do they ever buy medicine from *(shop's name redacted)* enterprise without going to see a doctor?

LK- No, first we showing doctor then We go to medical shop with prescription.

MH-Do they have buy medicine to gives the poultry?

LK-No

MH-Why not?

LK- We take animal medicine from *(NGO name redacted)* not from *(shop's name redacted)* enterprise.

IP- So in *(shop's name redacted)* enterprise animal medicine is available or not.

LK- May be available, we didn't know.

MH- Do they know what antibiotics are ?

LK- We have heard about the antibiotics.

IP-Where from you heard about antibiotics?

LK-We heard about antibiotics from *(NGO name redacted)*.

IP- What you heard about antibiotics?

LK- When the poultry having big disease like Gumboro, Ranikhet then antibiotics is use.

MH- Do they know any name ?

LK- No. Birds are vaccinate against those disease (Gumboro, Ranikhet), If vaccine is not done ,then this may occur then antibiotics is use.

MH- Do she know any thing about antibiotic resistance ?

LK- No ,. May be heard about but I can't remember.

MH- Does she have any old medicine packet ?

LK- No all medicine are finished.They (People of *(NGO name redacted)*) give medicine in a paper. But in this they are not responsible, they told us for bringing bottle for carrying medicines.(Then LK ask his mother is eye drops is remain or not). Ya this is the medicine paket ,this gives in the eye when the poultry having pox.

IP- From where you get this eye drops?

LK- From *(NGO name redacted)*.

MH- How long they have this ?

LK- We give the medicine for 10 days.

IP-How many time you use the eye drops per day?

LK- One time per day.

IP-Are they told you how many days for use it?

LK-They told me to use it upto the cure of the eye .

IP- When you purchase this medicine?

LK-25 days ago.

MH- How many birds need to treat ?

LK-We have 10 RIR and pox occurred in all birds out of 10 , two birds are dead due to pox. Pox not occur in desi birds.

MH-Sorry

LK-

MH- Why do they keep the RIR in cage other than free roaming ?

LK- Because dog will come and catch them.

MH-Where did they go in the night ?

LK- In this shed.

MH- Where did the *desi* go in the night ?

LK- Also In this shed .

MH- Do they stay together?

LK- No, they stay separately.

MH- Ok great you Does they have any questions for me?

LK- Where are you live?

IP- Basically I am from Kolkata, He(MH) from London, he is research assistant he doing research last time he went to *(area name redacted)*.

LK- We have one relative , he also BLDO.

IP- I am also Veterinary student, doing my masters.

LK- You may know him.

IP-What his name?

LK-Dr. *(person's name redacted)*

IP- Where he posted?

LK-He posted in *(local town name redacted)*

IP-May I heard his name

LK- Previously he posted in *(local town name redacted)*. He is from *(area name redacted)*.

MH-Ok
