# Supplementary material for: “If It Works in People, Why Not Animals?”: A Qualitative Investigation of Antibiotic Use in Smallholder Livestock Settings in Rural West Bengal, India
Source: Antibiotics (Basel). 2021 Nov 23;10(12):1433. doi: 10.3390/antibiotics10121433 (PMC8698124; doi:10.3390/antibiotics10121433)
Supplement: Supplementary file 1 [file antibiotics-10-01433-s001.zip › Supplementary S1_ Interview Transcripts/Site 2/LK32 (site 2).pdf]

Code for Study - ‘If it works in people, why not animals?’: A qualitative investigation of antibiotic use in smallholder livestock settings in rural West Bengal, India: LK32, Site 2

Date: 17/01/2020

Location: Site 2

Interviewee: Livestock keeper (LK)

Interviewer: Mathew Hennesey (MH)

Transcription: Indrajit Patra (IP)

In Bengali language

MH- Mat Hennessey

LK- livestock keeper

IP- Indrajit Patra

MH- Could you(IP) tell me what type of animal does he(LK) keep here ?

LK- We keep cows , goats and poultry .

MH- How many cows?

LK- We have 5 cows previously, among those cows 4 are already sold and now we have one milch cow.

MH-One cow now?

LK- Yes , one cow.

MH- How many goats they have ?

LK- We have 5 goats previously, among those 3 goats are already sold and now we have two goats.

MH- how many poultry ?

LK- We have 12 poultry previously, among those 9 poultry are already dead and now we have 3 poultry alive. Most of the poultry are dead that why we lost interest of poultry rearing.

MH- What happened with those nine poultry?

LK- First symptoms of Chaky diarrhea, some people told that it may be Ranikhet .

IP-What you have seen in those chicken?

LK-I seen that birds take feed in night but in next days morning birds are dead suddenly and birds can't digest their feed.

MH- Ok that very sad, What they do when this was happened ?

LK- We showing the bird to the doctor.

IP- Whom you showing the birds?

LK-Dr. *(person's name redacted)* , he is the students of Kolkata animal husbandry college. Some time we go to Model farm also.

MH- Did what?

LK- He gives injection.

MH-Where he(*(person's name redacted)*) came from?

LK- He came from *(NGO name redacted)* and he has also one chamber here..

MH-where is the chamber ?

LK- He has the chamber here in *(local town name redacted)*.

MH- what is the line of treatment he gave?

LK- He gave one injection .

IP- Do you know the name of the injection?

LK- No , I didn't know the name of the injection. Although he gave injection but no are alive.

MH- To the 3 other birds ?

LK- He gave injection to all the birds.

MH- How many bird does he give injections ?

LK- All 12 birds,first he gave injection in one bird then he came to know when he sow that the infection is spread in other birds .That's why in next day he gave injection to all birds.

MH- what happened after that ?

LK- Nothing is happened, all birds are dead.

IP-When the all are dead?

LK-In one to two days all birds are dead.

MH- Are the other 3 birds okay ?

LK- one bird we ate and another 2 birds are sold.

MH- Are this 2 birds are sold ?

LK- Yes

MH-How many birds they have today ?

LK- Now we have 3 birds and all the birds are newly purchased birds.

MH- What type of problem they have with goats ?

LK- Few days ago the goats suffering from fever.

IP-How you understand that goats suffering from fever?

LK- Goats didn't eat feed, we go to chamber, they measure the temperature with the thermometer and told us that goats are suffering from fever.

MH-Whose chamber ?

LK- *(Person’s name redacted)*chamber.

MH- Is this near by the house ?

LK- Yes, near by to my house.

MH- What happen then?

LK- He only give the injection.

MH- Do they know what type of injection ?

LK- No .

MH- were there any other medication given ?

LK- No other medications is given.

MH- what happened to the goat ?

LK- Then goats are dead.

IP-How many goats dead?

LK- 3 goats dead.

MH- When this was happened ?

LK- The goats did not dead in same day ,they died at one days interval.

MH- How long did it happen?

LK- Upto 15 days ,they survive upto 15 days and then they died .

MH- when this was happened,one months ago or 2 months ago or 3 months ago ?

LK- 1.5 to 2 months ago .

MH- why do they go to *(person's name redacted)* doctor other than *(NGO name redacted)* ?

LK- Because *(person's name redacted)* is near by of my house, we have to do our home work so we have less time for this and *(NGO name redacted)* is far away from my house.

MH-How much did *(person's name redacted)* charge ?

LK- It vary. Firstly he charge Rs. 90 then he charge Rs. 150 total Rs.240 to 300.

IP- After that?

LK- Goats are dead.

MH- Did he this amount for all 3 goats?

LK-Yes, it is for all the 3 goats.How we prevent this disease?

IP- You have to vaccinate those animals. Is nasal discharge is present?

LK- Yes, also difficulty in breathing. *(NGO name redacted)* people some time came and vaccinate the animals.

MH- Do they ever go the BLDO office ?

LK- In *(local town name redacted)* BLDO office, we went their few days ago and make Ticket of Rs. 2.

IP- When you go to BLDO office?

LK-Near about 6 months ago.

MH- And for what ?

LK- For bring deworming and vitamins .

MH- For which animals ?

LK- For the goats .

IP- Only for the goats?

LK- We have castated male goats due to over eating of leave , acidosis may develop, we went to BLDO office with is goats.And the goat was cure.

IP-Is the goats cure?

LK- Yes.

MH- Why they don’t go to the BLDO office when this problem was arised with the goats before.

LK- That time was the paddy harvesting time that’s why we had no time for going BLDO office, and we call local doctor.

MH- How long they take to go to the BLDO office ?

LK- 1hour for going 1 hour for returning,1hourwe have to stay there so total 3 hour.

IP-What is cost?

LK-Total transportation cost is Rs. 60 if we go Thier with the goats.

MH-For the transportation, go there and back?

LK-Yes, And Rs. 2 for making ticket.

MH- What do you mean by tickets ?

LK- Tickets mean registration fees.

MH- Do they use the mobile veterinary camp ?

LK- No, Mobile Veterinary camp is held here.

IP- Is any came with the vehicle doctor and Paravet , vaccinate and treat the animals?

LK- Yes ,One year ago some people came with vehicle and vaccinate and treat the animals.

IP-Where the camp occurred?

LK- One 1 km away from my house.

MH- Do they know about the use of antibiotics?

LK- During pregnancy cow gave injection of Tetanus and gives vitamin.

MH- Can they explained little bit more about tetanus?

LK-During pregnancy, two people came and gave tetanus injection.

MH- Do they know any antibiotics use in human?

LK-In fever cough and cold antibiotics is use.

MH- Do they know any names ?

LK- No,We have prescription, In fever we all take antibiotics.

MH- where is the local doctor ?

LK- In *(local town name redacted), (local town name redacted)*more.

MH- Did the local doctor give any advise and medicine about animals ?

LK- No, They didn't.

IP- Where from you collect the medicine?

LK-From *(local town name redacted)* Hospital.

IP- Why you did not take the medicine?

LK-We this medicine extra for future use.

MH- How many medicine Did they collect?

LK- This 10 medicine.

MH-They collect this 10?

LK- Yes

MH- Why they did not use ?

LK- For future purpose,we kept it .

MH- why did they collect ?

LK- If we have some problems, we use it for future.

MH-What type of problem they take this?

LK-In cough and cold and difficulty in breathing,we take this medicine.

MH- And if they get cough or cold ,how many they take?

LK- My sister told me to take this medicine twice daily.

IP-How may days you take?

LK-We take it for 5 days ,and my sister told me to complete the course.(LK told that she take the medicine for 5 days but IP wrongly told to MH that LK take the medicine for 10 days)

MH-What about the costs?

LK- We get the medicine from hospital so the medicine is free of cost because government supply this medicine. This is antibiotics this works very good.

MH- Why the hospital give them this tablets ?

LK- Because my sister working in the hospital and my gives me this medicine.

MH- So the sister work in the hospital ?

LK- Yes

MH- And with they keep always some antibiotics in their house ?

LK- Yes always.

MH-What they do if the breathing problem is not improved ?

LK- Then we go to the hospital.

MH- OK ok.
