# Supplementary material for: “If It Works in People, Why Not Animals?”: A Qualitative Investigation of Antibiotic Use in Smallholder Livestock Settings in Rural West Bengal, India
Source: Antibiotics (Basel). 2021 Nov 23;10(12):1433. doi: 10.3390/antibiotics10121433 (PMC8698124; doi:10.3390/antibiotics10121433)
Supplement: Supplementary file 1 [file antibiotics-10-01433-s001.zip › Supplementary S1_ Interview Transcripts/Site 2/LK33 (site 2).pdf]

Code for Study - ‘If it works in people, why not animals?’: A qualitative investigation of antibiotic use in smallholder livestock settings in rural West Bengal, India: LK33, Site 2

Date: 16/01/2020

Location: Site 2

Interviewee: Livestock keeper (LK)

Interviewer: Mathew Hennesey (MH)

Transcription: Indrajit Patra (IP)

In Bengali language

MH- Mat Hennessey

LK- livestock keeper

IP- Indrajit Patra

MH-Can you(IP)ask him(LK) if he (LK) has any questions about the project then we(IP&MH) start?

LK- Yes, What kind of project this is.Please tell me about the details of the project.

IP- Actually this is about the uses of antibiotics and it is about the resistance about the antibiotics like oxytetracycline work very well in past but now a days it(Oxytetracycline) not work properly. They (MH) will take information from you and they (MH) analysis the data and take a further step to stop it(resistance of antibiotics).

MH- how long ago household have been keeping the commercial poultry ?

LK- 7 months ago .

MH- why did household stop to keep the poultry ?

IP- Why you(LK) start to keep poultry?(IP did not hair the word " stop" instead of stop he hair start)

LK- We started the farm for money. In *(area name redacted)* have area great opportunity for poultry farming. Some people from *(local town name redacted)* , *(local name redacted)* they came here(*(area name redacted)*) and supply chicks. If government helps us or privately we start to produce chicks here(*(area name redacted)*) then here(*(area name redacted)*) reduce of jobless people. Here demand of the chicks is very high.

MH-what type of chicks dose he hatch?

LK- Desi, kuroiler,duck(khaki camble, Indian runner , Chinese duck)assel

MH- Where he get this egg from ?

LK- From the local farmers and also from my own farm. Mainly I had desi chicken farm.I also hatch RIR eggs.

MH-what is the capacity of the incubator?

LK- 2000 eggs

MH-How many eggs do you have now?

LK- Now 600 eggs.

MH-Out of 600 how many of each type ?

LK-100 is assel ,400 *desi* and RIR 50 approx and 50 Vanaraja .

MH-Is Vanaraja chicken?

LK- Yes, it is dual perpus bred for meat and egg both.

MH- So mostly desi?

LK-Yes because here desi poultry demand is very high.And meat protein is very high.In Calcutta also the demand of desi chicken is very high. And tourist who came to visit *(local town name redacted)*(Famous place of Sundarban) they also prefer the desi poultry.And the rate of the desi poultry is very high.

MH- How did you learn how to do this ?

LK- I got my H.S(High secondary)degree and our financial condition is not good then I go for cultivation.Then ila(Cyclone) heat here(*(area name redacted)*).Then I found that I can't do much more from the cultivation.I also wants to do big things though I have less financial support but much more willing power.Then I found that it is impossible to buy chicks and start business because for 100 chicks Rs. 3000 required.Then I wants to know to that how the eggs hatch. Then i go to Chennai for the Construction work for source of the money but it(construction work) is not my occupation is not my interest. I another aim for go there(Chennai) .I came to know about this technique in Chennai. In Chennai big poultry is there.Then I know that how to make the mechine(Incubator).They did not told the details about the mechine (Incubator). After getting knowledge from the Chennai I came at *(local town name redacted)*near the new *(local area name redacted)*in west Bengal.In *(local town name redacted)*also a factory for producing the incubator.I connect them with the help of internet. I go to *(local town name redacted)*and watched that how to make the mechine.They also not interested to told me how to make the incubator. Then i enlisted the raw material for making of incubator.Then I started the making the incubator in my house with the cheap material ,they(people of *(local town name redacted)*) uses the tray is about Rs. 500 but I uses here (my

own incubator) second quality materials , I uses wood, I only buy the mechine and tray.If I bought it (incubator) from market it price will be 60000.

IP- I also surprised about his (LK) work.

MH- Out of those egg what percentage was hatched ?

LK- approx 85% of hatching rate

MH- What was the cost of *desi* chick ?

LK- Rs. 30 per chick.

MH- What is the cost of RIR chicks?

LK- It also Rs. 30 per chick and same rate for Vanaraja.

MH- What is the cost of duck price?

LK-Rs. 45 per chicks.

MH- What is the cost of quoiler?

LK-The different in different Day

MH-What is the range?

LK- It's vary from Rs. 25 to 33.

MH- When the chicks hatched how long he keep here ?

LK- 4 days.

MH- What did you do in the 4 days with those chicks ?

LK- I kept the chicks in brooder , I make the brooder here with the help of bulb , heat is provided to brooder , with the activities of chicks we know about the temperature.If temperature is required to the chicks they came near to lamp.If temperature is not required for them they go away from the bulb.

MH- Wheather he give any treatments within this 4 days ?

LK-Yes, first vaccination in 4 days Ranikhet vaccine .

IP- When you do the vaccine?

LK-Vaccine is given in between 1 to 4 days. After the hatching I give fresh water to the chicks .And after 2 to 3 hours give the feed.

IP- What type of feed?

LK- Mainly the commercial feed(feed from market) also give supercox with water , then after 2 to 3 hour later i remove the water and give vitamins with the drinking water. Vitamine name is ambiplex.

MH-Where from the house hold buy feed ?

LK- From the market

MH-which market ?

LK-local market

MH-When dose he give the ranikhet vaccination ?

LK- between 1 to 4 days

MH- How many times ?

LK-one times

MH- Do household give any other medication ?

LK- No, I didn't do any medication.

MH- how did he sale the chicks ?

LK- My chicks supply methods is different.Other people use cage but use plastic tray I always gives it to the seller man . After transplantation of chicks seller man return the tray. It is cost effective.By this method I can save upto Rs. 50. local farmers are buy the chicks. Farmer came from Mallahkhali,Kumirmari and Rangabelia.

MH-Does the local farmer buy this on cash or credit ?

LK-cash only.

MH- where he get the medicine and vaccination ?

LK- (*Site 2 name redacted)* model farm in *(NGO name redacted).*

IP-Any other places?

LK- *(Local town name redacted)*market Like *(shop name redacted)* and *(shop name redacted)*

MH-What does he buy from *(shop name redacted)* ?

LK- Feed , medicine

MH-What type of medicine ?

LK-Ranikhet vaccine,Gumboro vaccine(IBD Vaccine)

MH- How many poultry he keep here ?

LK- 400 poultry but all are sale in tourism time.

IP- How many you have ?

LK- About 380 birds are sale out of 400.

MH- How many birds left now?

LK- Near about 30 birds.

MH- What type of bird are they ?

LK-all *desi chicken.*

MH-How much cost he sale ?

LK- That time weigh of the bird near about 1.5 kg. And price Rs. 250 per kg.

MH-In local market ?

LK- All customers are coming my house and they purchase chicken from here(LK house)

MH- How long *desi* take for ready to sale ?

LK-Approx 70 to 80 days

MH- During this 70 days how you look after them(Chicks)?

LK-I offer 3 time food in days and proper vaccination.

MH-What are the proper vaccination?

IP- Would you tell us the vaccination schedule?

LK- Between 1 to 4 days ranikhet first vaccine F1 starin ,

after 7 to 10 days gumboro vaccine (IBD),about 21 days booster of F1 strain

ranikhet vaccine,in 30 days I gives the dewormer like piperazine.

MH- How many time you gives dewormer?

LK- 1 time in 70 days.

MH-Dewormer given into the water or other ?

LK- Water

MH- Any other treatment or medication?

LK-In case of chalky diarrhoea or bloody diarrhoea supercox is given.

MH- When the bloody diarrhea happen what does he do

with the chicken ?

LK- I have checke the poultry every days. We kept the birds separately (quarantine it) and perfectly treat the chicken.

MH- Did you treat the every birds?

LK- yes, Supercox is given large amount in case of disease bird and small amount in healthy birds.

MH- Did you done any other medications?

LK- No, Birds are cure with the use of Supercox.

MH-Does he know what antibiotics are ?

IP-Did you use antibiotics with feed?

LK- I am not use any antibiotics in feed. I mix vitamin and calcium name is calfos DS.

MH- Did you know what antibiotics are?

LK- yes, I know that antibiotics increase the immunity power in disease time.

MH- Does house hold know what antibiotics do?

MH- Is there any other problemother than bloody diarrhea ?

LK- No

MH-What would he dowhen there is some problems ?

LK-I go to the *(NGO name redacted)* and post-mortem the bird

and burial the deaths birds .

MH-Who do the PM ?

LK- *(Person's name redacted)* paravet done the pm. If good medicine and management is given then less problem is occur.

IP- He (LK) made incubator himself.

MH-Ya he(LK) having good knowledge it impressive.

LK- I will show some things. I bring this for hydrophonic farming. It PH meter, It TDS meter.

MH- What is TDS.

IP- Total dissolve solid.

MH- Oh ,ya

LK- This is Isriel concept.This not properly use in India. I wants to experience this.This is new project.

MH-When you start that(Hydrophonic farming)?

LK- Heavy infrastructure is required for this farming.Now I am purchasing the equipment for starting the project.

MH-Take investment?

LK- Ya , First investment is done. But I have problems of money.

MH-Does he have any other animals ?

LK-3 goats ,We have many cow but now most of them are sell now 1 cow is present and 12 ducks

MH-Does he ave any problem with the goats ?

LK- No,and we had 2 cow and one is died suddenly.

MH-When the animal is dead ?

LK- 6 months ago.

MH- What happen with the animal?

LK-we don't know reason of the animals death. We sent the photo of cow to the V.S.(Veterinary Surgeon)

MH-Did die suddenly ?

LK- Suddenly .

MH-Who sent the photo to V.S.(Veterinary surgeon)?

LK-Uncle(*(PErson's name redacted)*, Paravet of *(Site 2 name redacted)*.) sent the photo to v.s.

MH-If he have fever with cow or goat what would he he do then ?

LK-We the *pranibandhu* .

MH-Do they give any human medication to animals ?

LK-no .

MH-Did he do any vaccination to goat or cow ?

LK- No vaccine is done in case of duck. In case of cow FMD vaccine is done.

MH- What for goats ?

LK- No

MH- Why not for goats?

LK- I did not get the vaccine . Because vaccine are supply from Government is not purchase from local market.And the cold chain is not maintain in Ranikhet vaccine.

MH- Does he have hear about mobile veterinary camp here ?

LK-Yes but place far away from the house.

MH- How fur away from this place?

LK- 3 to 4 km away from my house.

MH- When was the last time the camp occurred?

LK- Near about 6 months ago.

MH- Did people from this area go the camp 6 months?

LK-No I am not know properly .

MH-Did he go ?

LK-No.

MH- Thats great , Thank you.
