# Supplementary material for: “If It Works in People, Why Not Animals?”: A Qualitative Investigation of Antibiotic Use in Smallholder Livestock Settings in Rural West Bengal, India
Source: Antibiotics (Basel). 2021 Nov 23;10(12):1433. doi: 10.3390/antibiotics10121433 (PMC8698124; doi:10.3390/antibiotics10121433)
Supplement: Supplementary file 1 [file antibiotics-10-01433-s001.zip › Supplementary S1_ Interview Transcripts/Site 2/LK34 (site 2).pdf]

Code for Study - 'If it works in people, why not animals?': A qualitative investigation of antibiotic use in smallholder livestock settings in rural West Bengal, India: LK34, Site 2

Date: 16/01/2020

Location: Site 2

Interviewee: Livestock keeper (LK)

Interviewer: Mathew Hennesey (MH)

Transcription: Indrajit Patra (IP)

In Bengali language

MH- Mat Hennessey

LK- livestock keeper

IP- Indrajit Patra

MH- Could you (IP) ask him(LK) how many people live in this house ?

LK- 9 members.

MH- How many children ?

LK- 4 children .

MH- What are the different type of animals they keep ?

LK-5 cattle,18 ducks and 8 goats ,also the 60 Kuroiler.

MH- Why do they keep them?

LK-Milk and cow dung& fertilizer.

MH- How much milk they get each day from cow ?

LK-1kg per day per cow.

MH-What they use the milk for?

LK-Out of 5 cows only 2 cow in lactations period. Generally milk drink by the children in our house.

MH- Did they sell any milk?

LK- No

MH- Why they did not sell the milk?

LK- Because 2 lactating cow only gives 2 kg milk that why we did not sell the milk .

MH-Whay they use the ducks for?

LK-For the eggs.

IP- Are you consume duck meat?

LK-Some time we consume duck meat.

MH-How many eggs they get each day?

LK-4 to 5 eggs each day. All day duck did not lay eggs.

MH- Did they sell the duck eggs?

LK-Some time we ate the eggs some time we sell the eggs.

MH- How many they sell and how many they ate?

LK- We ate half and half sell in market.

MH- Where did they sell the eggs?

LK-In the local market.

MH-How often they eat the duck meat ?

LK- In one time in 2 months.

MH- Why they keep the goats ?

LK-for Meat , for the offspring for sell .

MH- Did they sell any goat?

LK- We did not ate any goat all goat sell in the market.

MH-Where they sale the goats ?

LK-The seller man came in house and purchase the goat from here .Some time we sell the goat in market.

MH- What they use for kuroiler ?

LK- Kurolier is not for eggs it rear only for meat purpose, and we sell the Kurolier.

MH - Would they ate themselves?

LK- No.

MH- Where they kept the cattle ?

LK- We kept the cattle in the shed.

MH- What do they feed to the cow ?

LK- Mash,straw,grass .

MH- Any think else?

LK- Ya ,little amount of concentrate.

MH- from Where they get the concentrate?

LK-From the local market .

MH-How often they give concentrate ?

LK- we are very poor so we gives little amount of concentrate during the lactations period.

MH- Do they give any other treatment or medication ?

LK- Some people from government came one to two time in year and we have one village doctor they treat the animal. Some people came from *(NGO name redacted)* and they did the vaccine.

MH- Is that *(NGO name redacted)* or *(NGO name redacted)*?

IP- No it is *(NGO name redacted)* it is another project.In *(NGO name redacted)* there is rule of ring vaccination to protect the Tiger. So they give FMD vaccine to the periphery.

MH- What was the last problem the cow had?

LK- Last time lesion of pox in whole body.

IP- What did you do ?

LK- Pranbandhu came and treated the animal give injection.

MH- Okay.and do they have any problem with ducks ?

LK- duck unable to stand and bloody diarrhoea.

MH- Which duck?

IP-The duck that they rear in their house.

MH- Is bloody diarrhea in this movement?

LK- No.

MH- What did they do when bloody diarrhea occur ?

LK- We go to medicine shop and bring medicine from there. And give medicine to disease bird. After giving the medicine some of them alive and some of them are dead.

MH- Where they go ,which shop ?

LK- We go to *(NGO name redacted)* and model farm.

MH-Can you tell them where is the *(NGO name redacted)* situated ?

MH-in *(local town name redacted)* .

MH- Do they know what medication is given to the duck ?

LK- No. We didn't know the name of the medicine.

MH- Is the medicine is liquid or anything else?

LK-Yes it liquid medicine.

MH-How did they give the medication to duck ?

LK- Some time we give through drinking water, some time we give the medicine with the rice, and some time we give it directly in the mouth.

MH-Where they keep the duck ?

LK- In this shed

MH-Do they let them out in the day?

LK- Yes , in day time duck scavenging their feed.

MH- You(IP) just ask them(Family members of LK) to stop because it(Conversation) is recorded.Or we(MH,LK&IP) can go to any other else.

IP- Brother(Family members of LK) Please stop to make noise because it(Conversation) is recording.

MH-Is that ok. Ok

MH- Where they keep the goat ?

LK- In the shed

MH- What they feed the goat ?

LK-Mash,grass & Wheat.

MH- from where they get mash?

LK- in local market.

MH- What type of problem with the goats ?

LK-bottle jaw,fever, and diarrhea.

MH- What they usually do when it is happened ?

LK- Some time animal cure some time animal dead.

IP-Is pranibandhu came and treat?

LK- Yes.

MH- Don't you(IP) suggest them(LK) the panibandhu. Is the panibandhu came in all the condition?

LK- Yes.

MH- Why do they call pranibandhu in case of goat instead of going *(NGO name redacted)* or *(NGO name redacted)* ?

LK-Because *(NGO name redacted)* is fur away from this place. Problem to go the *(NGO name redacted)* with the goat because road conditions is very bad.

MH- Why they use *(NGO name redacted)* instead of pranibandhu when duck ill?

LK- First treated by pranibandhu then if not cure then we go to the *(NGO name redacted).*

MH- Where they keep the kuroiler?

LK- In that she'd.

MH-How long they keep the kuroiler for ?

LK-we keep it for 2 to 2.5 months.

MH- All the kuroiler are same age or different ?

LK- same age

MH-from where did they buy the chicks from?

LK- From *(local town name redacted) (NGO name redacted).*

MH- When they buy the chicks in cash or credit?

LK- In cash. Some time in credit also.

MH-what type of treatment they give for 60 to 65?

LK- Medicine provide from the *(NGO name redacted)* and we purchasedfeed from local market ,one vaccine is done in nose in 1 to 4 days .

MH- Did they know the name of the vaccine?

LK- No, it given in 4 days.

IP-Then?

LK-Then upto 20 days no vaccine is done we provide vitamin 3 time in between 20 days. In 21 day another vaccine is done.

MH-How do they give the vitamins ?

LK-Mix with the drinking water and provide dewormer with the feed.

IP- When you done the deworming?

LK- After the 5 to 6 days of 21 days, it is 26th of 30th day old .

MH- Did they know the name of the dewormer?

LK- Doctor are given the dewormer we don't know the name of dewormer. The man (Worker of *(NGO name redacted)*) who supply the chicks he also gives medicine (Vaccine and dewormer).

MH- How they know how do this?

LK- Instruction given from *(NGO name redacted)* when they gives chicks.They told us the management procedures.

MH-Does they have instruction paper ?

LK-No. They (People of *(NGO name redacted)*) gives instructions by mouth.

IP- When you give the calcium?

LK- after giving the dewormer we provide calcium in 30 th days.

MH- Any other treatment in between 30 to 60 days?

LK- No

MH-Any problem with the kuroiler ?

LK-Liver problem .

MH-What happen to this one?

LK- Chaky diarrhea.

MH-When this problem start?

LK-It start from yesterday out of 50 chicks 1 chicks having this problem.

MH- You (IP) told them this is Ranikhet?

IP- No, Who told you(LK) this is Ranikhet?

LK- May be Ranikhet, the birds unable to eat feed.

MH- What treatment given the bird ?

LK-we go the *(NGO name redacted)* with the bird, usually 1 to 2 % mortality is normally seen in the birds .

MH- What treatment given to this bird?

LK- No treatment is done.

MH- When they go to *(NGO name redacted)*?

LK- No we didn't go to *(NGO name redacted)* for one chicks.

MH- Did they give any treatment to this bird?

LK- No

MH-What the do if the bird is not better ?

LK-We go to *(NGO name redacted)* for the post mortem for proper diagnosis.

MH- Do they take the bird to the model?

LK- If the bird is not cure then we go to *(NGO name redacted)*.we are waiting for few days.

MH-Do they have any medication or treatment here that we(MH&IP) can see for Kurolier?

LK- No we have no documents. Medicine is provided from *(NGO name redacted)*.

MH- Did they have any old packaging here?

LK- No

MH- How do they sale the Kuroiler ?

LK-We sell it per kg body weight in to the local marke

MH-Did they sell to the market or some body came for buy?

LK- People came here for purchase the Kurolier.

MH-Who came to buy ?

LK- Villagers bought the kurolier for ate.

MH- How many did they buy?

LK-Some time one person purchase one or two kurolier , some time in occasion person purchase 20 kurolier.

MH-When they buy next set of chicks?

LK-When all previous kurolier are sell after 10 to 12 days later next set of chicks are buy.

MH-Why they wait for 10 to 12 days?

LK- We have clean the shed and prepare the shed for next batch.

MH- What they use to clean the room?

LK- Lime and bleaching powder.

MH-What do they do with old littre ?

LK- Use as manure .

MH- What they do with manure?

LK-Use for cultivation.

MH-How much income from animals ?

LK- 2000 to 2200 for kurolier in one month if we consider the all animals then 20000 yearly we earn from animals.

MH- How much the total income?

LK- 20000.

MH- Per month?

LK- No per year

MH- Is that only from the animal?

LK- Yes from the all animals.

MH-Do they make money from other sources ?

LK- Cultivation .

MH-How much from cultivation ?

LK- We have only 1.5 bigha land for cultivation , it income near about 1500 per months.

MH- So double from the animal?

LK-Yes, most of the money came from poultry birds.

MH-What proportion of percent from kuroiler ?

LK- 50% half of total income.

MH- Can you(IP) ask them(LK) what antibiotics are?

LK-Ya we know about antibiotics.

IP-What you know about the antibiotics?

LK-Antibiotics use in fever.

IP- Where from you(LK) know about antibiotics?

LK- I know antibiotics use in human not in animal.

MH- Do they know any name of antibiotics ?

LK-No.

MH- Can they explained more how antibiotics use in human?

LK- Tablet Cetrizine is human antibiotics.

IP-Any more?

LK- No

MH- Why it(Tab. Cetrizine)use in human?

LK- In case of fever it is use.

MH-If someone having fever in this house where they go ?

LK- In local doctor . And *(local town name redacted)*hospital.

MH-Does local doctor give any advice for animal ?

LK-No.

MH-Can you ask when human medicine use in animals ?

LK-Yes, One human digestive syrup price is about Rs. 205 ,we forgot the name of the syrup we use the syrup for goat.

IP- In which animal you use it?

LK- In case of goat.

MH-Did they have any vial that we can see?

LK- Yes.

MH-Could we see that.

IP- Yes but after the end of interview.

MH- When may they animal medication to the human?

LK- No, you(IP) are from where?

IP- I am from *(town name redacted)*Veterinary college.

MH-Do they have fish as well ?

LK-Yes

MH-Any problem to fish?

LK- No, only the *bagda* problem in the fish the virus problem.

MH- What is bagda?

LK-Bagda is a virus disease. In this problem fish are usually dead.

MH-Do they give any medication ?

LK-We use lime and bring medicine from the market.

MH- Do they know what type medication is done?

LK- oxygen is given in the pond by machine.

IP- From which market you bought the medicine?

LK- In *(local town name redacted)*market.

MH- Did they have any medication here?

LK-No

MH- Ok ,Do they have any question ?

LK-No

IP- They ask the question in the start of interview.

MH- Ok they are happy?

IP- Yes

LK- Is this work(research and interview) start from here?

IP- No, It first start in *(local area name redacted)* now we (IP,MH&All team) work in *(Site 2 name redacted)*then we go to *(Site 1 name redacted)*in next.

MH- We go to *(Site 2 name redacted)*in November and project is going on for 2 year.How to improve the access of medicine.

LK- Is the project is only upon the animal?

IP- Not only the animal but also in human *(Kolkata institution name redacted)* is also associated with the project.
