# Supplementary material for: “If It Works in People, Why Not Animals?”: A Qualitative Investigation of Antibiotic Use in Smallholder Livestock Settings in Rural West Bengal, India
Source: Antibiotics (Basel). 2021 Nov 23;10(12):1433. doi: 10.3390/antibiotics10121433 (PMC8698124; doi:10.3390/antibiotics10121433)
Supplement: Supplementary file 1 [file antibiotics-10-01433-s001.zip › Supplementary S1_ Interview Transcripts/Site 2/LK35 (site 2).pdf]

Code for Study - 'If it works in people, why not animals?': A qualitative investigation of antibiotic use in smallholder livestock settings in rural West Bengal, India: LK35, Site 2

Date: 18/01/2020

Location: Site 2

Interviewee: Livestock keeper (LK)

Interviewer: Mathew Hennesey (MH)

Transcription: Indrajit Patra (IP)

In Bengali language

MH- Mat Hennessey

LK- livestock keeper

IP- Indrajit Patra

MH- Can you(IP) thank them for taking the time to talk us today?

IP- He (MH) thank you(LK)

MH-How many peoples lives in this house ?

LK- 6 peoples .

MH-adults and children ?

LK- one children .

MH- what type of animals do they keep here ?

LK- cows- 2 ,poultry -20 all desi *murgi* .

MH- Why do they keep the *murgi* for ?

LK- for the family income .

MH- what are the different type of family income they have ?

LK- working as labour.

MH- What proportion of income they get from animals and labour ?

LK- mostly came from the labour and less from animals .

MH- How do they make income from *desi murgi* ?

LK- we keep *murgi* for the eggs and meat purpose ,we generally eat the egg on the daily basis.if egg remain in excess amount , they sale it to the local market.

IP- Would you ate meats of poultry?

LK- No , it only to the sell to market.

MH- How many eggs do they eat each day ?

LK- No we are not eating egg in every day, we ate 4 to 5 eggs per month.

MH- How many eggs do they sale?

LK- we are not sale eggs in every time. We excess egg produce then only we sale it.

MH- How often they eat desi murgi ?

LK- When delicate came in house in special occasion we ate meat . Mostly 1 to 2 time per month.

MH- who eats the eggs ?

LK- all members of the family.

MH- What type of products are given to the birds as feed?

LK - They feed the kitchen by products,mash feed ,wheat , paddy.

MH- Do they buy any other special food ?

LK- mash and wheat .

MH- Why do they buy mash for them ?

LK- mash helps to grow of the bird properly and increase the weight of the bird.

MH- How often do they feed the mash and wheat ?

LK-We give it everyday in morning in evening and in afternoon we gives them rice .

MH-Do they give any treatment or medication to the desi murgi ?

LK- deworming and vitamins and some time in disease we gives medicine.

IP- What type of diseases?

LK- Disease like pox,weakness,chalky diarrhea, abnormal gait, dried due to vitamin.

MH- What do they do during the problems?

LK-we go to doctor for medicine.

IP-Which doctor?

LK- we go to the model of the *(NGO name redacted)* .

MH- Why do they go there ?

LK- Because we get medicine from there , here medicine is unavailable.

MH-Do they know what type of medicine should be given in this problems ?

LK-No , If we told the doctor , doctor gives medicine.

IP- Would you go to *(NGO name redacted)* with the poultry?

LK- Some time we go with the poultry , some time we only told the problem.

IP to MH- As they have no documents, they go to the modern farm or veterinary camp and take the medicine from the veterinary officers or pranimitra by telling the problem of the birds.Model farm gives medicine in a paper from big file(bottle).

MH-Do they give any other type of treatments to the birds ?

LK-No we bring medicine , vitamins from the government veterinary camp.

MH- When was the last time they went to the camp ?

LK-Around 2 months ago in November.

MH- Where was the camp?

LK- Camp occurred in the club beside the road which was near by our houses ,500 meter away from my house .

MH- Did they go with the bird?

LK- yes, we have go to camp with the goat ,cow , poultry and duck then they gives medicine, vitamin & vaccination.

MH- Are they told about vaccination?

IP- No, I think.

MH-Do they know about any type of vaccination ?

LK- During the health camp, they come to vaccinate the birds against pox,ranikhet disease or other diseases and the diarrhoea.

MH- Do they pay anything regarding vaccination or it is free of cost?

LK- Free of cost because it gives from government.

MH-What would be the cost if they go to the *(NGO name redacted)* ?

LK- Its depends upon the amount of medicine. In case 10 poultry birds cost may be Rs.20 or 30 .

MH- what was the cost is in last time ?

LK- we went to the model farm 4 to 5 months ago,due to inappittance and deficiency of vitamin in the birds and cost is 40 rupees .

IP-Is the bird cure?

LK- yes.

MH-How much the cost to reached to the model farm ?

LK- If we go with the bird with then the its cost 10 rupees because we it with the bag and if we go with the cattle then it cost 100 rupees .

MH-. And if they go out of the bird how much cost they have ?

LK- Without birds also 10 rupees .

MH- How long does it take to return from the farm?

LK- by foot 40 min required for transportation and 15 min required in model farm so total near about 1 hour.

MH- To whom they talk about the animal problems?

LK- we talk to the doctor of *(NGO name redacted)* .

MH-.Nobody else ?

LK- Here is no local doctor. Two local doctor sits in there(*(NGO name redacted)*). They also came here if we call them?

MH- When it happen?

IP- When you call the doctor last time?

LK- We call them 1 year ago for naval infection due to house fly and mosquito in the cattle .They came here and clean the infection with the forceps and gives medicine and cream.

IP- what they charge?

LK- They charge Rs. 100

IP- Is the animal cure?

LK-Yes. they charges 100 rupees and it cured.

MH- What type of medication they give ?

LK-They gave the betadine and one cream don't know the name .

MH- how much time the doctor takes to come here from *(NGO name redacted)*?

LK- 1 time and we look after for 7 days . We use betadine 2 time in a days.

MH- Any tablet or injection?

LK-No , only the betadine and cream.

MH- What did they do if the cow had bad fever or diarrhea and they could not take somewhere ?

LK- Primarily we treat the animal with heat garlic and oil . We use the garlic oil in the base of the ear and horn.If not cure then we call the doctor?

IP- Which doctor?

LK- Doctor of *(NGO name redacted)*, or Doctor of *(local town name redacted)*.

IP- What is name of the doctor of *(local town name redacted)*?

LK- *("Doctor" of local town name redacted)*

IP- *("Doctor" of local town name redacted)* or *("Doctor" of local town name redacted)*?

LK-No *("Doctor" of local town name redacted)*, Some time one doctor came from *(Local town name redacted)* name is *("Doctor" of local town name redacted)*.

MH- Is he a *paravet* ?

LK- No he is a doctor .

MH- What about the *pranimitra* ?

LK-No

IP- Is here any lady who treat the animal?

LK- Yes they came here and told that where the camp will be held etc

MH- What about the *pranibandhu* ?

LK- No.there is no *pranibandhu*.

MH- Where do they go for their own health ?

LK-We go the *(NGO name redacted)* in *(Local town name redacted)* hospital.

IP- If it is not cure then?

LK- Then we go to *(local town name redacted)* government hospital and if there is also not cured then they go the PG(sskm)hospital or *(local town name redacted)*Hospital.

MH-Do they know about the antibiotics?

LK- We know about the antibiotic from the doctor of *(local town name redacted)*and also from the human doctor .

MH-What did they know ?

LK- If there is a fever and cough have to administer antibiotics and we know about the course of antibiotics ,and if the disease is cured, course of antibiotics must be completed. If not completed the diseae may come back.

MH-Do they know why the disease came back ?

LK- No, We know it from the doctor. They told us to complete the course of antibiotics otherwise the virus may comeback and cough and fever occur.

MH- Do they keep any medicine here ?

LK-No, Only having cough syrup we mainly treating with Homeopathy.

MH- Do they have any animal medicine ?

LK-No

MH- What they keep this(Human medicine) for?

LK- For the cough.

MH-How many would they take each day ?

LK-In the cough and cold this mox (Amoxicillin) antibiotics is taken.It givn for 7 day in morning and night twice a day.

MH-Where did they get this from ?

LK- From the doctor of *(NGO name redacted)* they prescribed it and we purchased it from the market.

IP- From which market?

LK- In *(Shop keeper's name redacted)* enterprise shop.

MH-When did they buy this ?

LK-In cough and cold first treated with the homeopathy if not cured then we go to the doctor and doctor prescribe and we buy the medicine.

MH- How long ago ?

LK-1 year ago for tonsillitis and coughing of my son.

MH-When did they buy this from *(shopkeepers name redacted)* enterprise ?

LK-No we bought it from government hospital.

MH- When you get it(amoxicillin) from government hospital?

LK-Some time ago.

MH- Is there long time ago?

IP-Yes, long time ago.

MH- Is this medicine talking by any body now?

LK- No, long time ago some body take this.

MH- Do they use the medicine in the future ?

LK- No we are not taking this medicine because it is expired.

MH- When you this medicine to the animal?

LK- Yes some time.

MH- Did you give this medicine to the animal?

LK- No, because we are unknown about the medicine doctor know about the medicine after consulting the doctor we give the medicine.

MH- Why do they do that ?

LK-Because they don’t know about the medicine.

MH-Great,thank you , do you have any questions ?

LK-No thank you.

MH- Do they go the BLDO office ?

LK- We didn't go to the office often due to excess fare of vehicles.

MH- How much cost ?

LK- If we go alone Rs 24 required for up and down.

MH-How much the cost is if they go with their animal to the office?

LK- If we go with the cow then we have to reserve a car and 2 people have to go then more than 200 rupees required.

MH-How much time is taken to go there?

LK- If we go in morning 4 to 5 hours required.

MH- Ok I stop the recording.
