# Supplementary material for: “If It Works in People, Why Not Animals?”: A Qualitative Investigation of Antibiotic Use in Smallholder Livestock Settings in Rural West Bengal, India
Source: Antibiotics (Basel). 2021 Nov 23;10(12):1433. doi: 10.3390/antibiotics10121433 (PMC8698124; doi:10.3390/antibiotics10121433)
Supplement: Supplementary file 1 [file antibiotics-10-01433-s001.zip › Supplementary S1_ Interview Transcripts/Site 2/LK36 (site 2).pdf]

Code for Study - 'If it works in people, why not animals?': A qualitative investigation of antibiotic use in smallholder livestock settings in rural West Bengal, India: LK36, Site 2

Date: 18/01/2020

Location: Site 2

Interviewee: Livestock keeper (LK)

Interviewer: Mathew Hennesey (MH)

Transcription: Indrajit Patra (IP)

In Bengali language

MH- Mat Hennessey

LK- livestock keeper

IP- Indrajit Patra

MH- Can you thank them for giving interview ?

LK- Okay

MH-How many people live here ?

LK- My son and me live here.

MH-2 People?

IP- Yes.

MH-What animal are there ?

LK- 1 cow and 1 calf total 2 cow ,4 sheep,1 poultry

MH- Why they keep the cow for ?

LK- Cow dung and milk. We drink the milk first if extra milk produce then it sell to market.

MH- Why they keep the sheep for ?

LK- For sale the sheep and seller man come here and bought.

IP-When you sell the sheep and what is the price of the sheep when you sell it?

LK- In 6 month age of sheep we sell it and weight is about 4 to 5 kg and price is 2000 to 4000

MH- Does murgi here ?

LK- Yes.

MH-How many?

LK- One

LK-Why they keep that for ?

LK- For the egg and meat and extra are sale in the market.

IP- Are you ate the chicken?

LK- Yes, Some time.

IP- Are you sell the egg in market or sell it from the house?

LK- No , In case of egg we have to go to the market.

MH-Which market ?

LK- *(local town name redacted)* or *(local town name redacted)* when such.

MH-What was the last time the animal had an illness ?

LK- In the rainy season cow pox is there .

IP- What you have done then?

LK- we go the model and they give injection and orally tablet.

IP- Have you any documents like prescription?

LK- Ya , they have given the document but we are unable to kept it.

MH- Did the cow get better ?

LK- Yes

MH- Why they go the *(NGO name redacted)* ?

LK- First we call one doctor from *(local town name redacted)* I have forgotten the name and then she call paravet of *(NGO name redacted)* and nobody want to came that why we have to go to the *(NGO name redacted).*

MH- With the cow you go to the *(NGO name redacted)*?

LK-No, Some worker who work in the model I have seen the cow them.

IP- Are they doctor?

LK- No , they are helper.

IP-Are they writing about the medicine?

LK- No , they told me to go the *(NGO name redacted).*

MH- Are *(NGO name redacted)* people came here?

IP- yes, they are the helper of *(NGO name redacted)* they seen the cow and told her to go to *(NGO name redacted).*

MH- Did you know any name?

LK-No

MH- Did you pay?

LK- No I did not pay to the helper . I have pay to Model(*(NGO name redacted)*) near about RS. 108 to 109.

IP-Is this total cost transportation and charge of *(NGO name redacted)*?

LK- No transportation cost because go to *(NGO name redacted)* by foot it is the only the charge of *(NGO name redacted).*

MH- Why they call the doctor from *(local town name redacted)* first ?

LK- Animal is ill very much and he(doctor of *(local town name redacted)*) always come and that day he having problem that why don't came.

MH- Did you know the name of the doctor?

LK- No, We I call him , he came.

MH- When was the last time the doctor came?

LK- 1 year ago when the cow having fever and inappetide and given vitamins and other medicine I have forgotten the name of the medicine.

MH- How much he charged ?

LK- I have forgotten but it nearr about RS. 150 to 200, he did very good treatment.

IP- Is the cow cure?

LK- Yes

MH-When was the last time the paravet came here ?

LK-During the time of camp he came told me to go to the camp with the animal. In the camp vitamin ,capsules are given and some orally medicine and injection also given.

MH- Are the all animals given the injection and medicine or it only given to sick animals?

LK- No , it gives to all animal animal.

IP- Are you going to camp with the animal?

LK- yes.

MH- Do they know where the injection was given in cow ?

LK- In thigh portion .(Gluteal mussel)

MH- Do they know what type of injection was ?

LK- No

MH- How much cost in the camp ?

LK- No it is free of cost.

MH-When was the last camp ?

LK-2 months ago .

MH- Does she know when the next camp will be held ?

LK- No, Any information about the camp is not given.

MH-How did she got to know about the camp ?

LK-Some paravet came and they told about the camp.

MH- Okay Did she get any medication here we can look out ?

LK-Yes , one bottle I have.

MH-Do this poultry give egg ?

LK- No this is male ,one female is there which was eaten by the fox one month ago.

MH- Ohh nooo thats sad,Will she more poultry ?

LK- Yes 6 to 7 poultry is there but all are dead.

IP- When the poultry are dead?

LK- 6 to 7 months ago.

IP- All are dead?

LK- No out of 7 , 2 poultry are alive and 5 dead.

MH- Do they all died in same time ?

LK- No no in a consiquitive days .

MH- How many days poultry taken to die?

LK- near about 3 to 4 days. First chaky diarrhoea and inactive.

MH-What was the symptom when the outbreak occur?

LK-There was chalky diarrhea we go to the model and other place in *(local town name redacted)*

MH-. Can she explain what other place in *(local town name redacted)*?

LK-The bldo office near the *(local town name redacted)* post office.

MH- Why did they go the *(local town name redacted)* ?

LK- Sometime go the *(local town name redacted)*and bldo office due free of cost.

MH- How much cost to go to *(local town name redacted)*?

LK- Rs. 12 for going and 12 for returning total 24 .

IP- And what was the the cost if you go with the cow?

LK- Don't because we didn't not going with the cow.

IP-Ok

LK- Sorry I have to go to the market

MH-Okay thank you.
