# Supplementary material for: “If It Works in People, Why Not Animals?”: A Qualitative Investigation of Antibiotic Use in Smallholder Livestock Settings in Rural West Bengal, India
Source: Antibiotics (Basel). 2021 Nov 23;10(12):1433. doi: 10.3390/antibiotics10121433 (PMC8698124; doi:10.3390/antibiotics10121433)
Supplement: Supplementary file 1 [file antibiotics-10-01433-s001.zip › Supplementary S1_ Interview Transcripts/Site 2/LK37 (site 2).pdf]

Code for Study - 'If it works in people, why not animals?': A qualitative investigation of antibiotic use in smallholder livestock settings in rural West Bengal, India: LK37, Rangabelia GP

Date: 18/01/2020

Location: Rangabelia GP (Bagbagan)

Interviewee: Livestock keeper (LK)

Interviewer: Mathew Hennesey (MH)

Transcription: Indrajit Patra (IP)

In Bengali language

MH- Mat Hennessey

LK- livestock keeper

IP- Indrajit Patra

MH-Could you tell us how many people live here ?

LK- 4 people

MH- Any children ?

LK-No

MH- What is the main source of income ?

LK-Farming

MH- What type of farming ?

LK-This is the salted area that why Cultivate only the paddy.

MH- How much of that income comes from paddy and what from animal ?

LK-Now a days we are not able to rearing the animal due to small area, 90% from paddy, and rest from animal.

MH to IP- Could you ask them to stop the music?

IP- No, Because this is the cultural program.

MH- What type of animal they have here ?

LK-one cattle, two goats, fish with one pond.

MH- Any *murgi* (Poultry bird)?

LK- 4

IP-Any sheep?

LK-No

MH- What they use the cow for?

LK- Milk, dung and offspring.

IP- Are you drinking the milk? Or sell it.

LK-Most of the milk they drink small portion the sell.

IP- Is milker man came here for milking?

LK-Yes

MH- How much milk they get in each day ?

LK- 1 litter if animal may get good feed the milk may increase up to 1.5 to 2 litter.

MH-How much they drink?

LK-Half litter.

IP- What the price of the milk?

LK- Rs. 25/litter

MH-What they use the goat for?

LK-They rearing the goat for selling into the market.

IP- Are you(livestock Keeper) ate the goat meat (Chevron)?

LK-No

MH- How much money they get from goat to the market ?

LK- 1 year of age they sale the 10kg goat and get 5 thousand

MH- Why do they keep the chickens for ?

LK- for the eggs and selling the meat.they eat chicken 1 time per 2 month.

MH-What would the last problem in the cow ?

LK- The animal not came into the heat. They go to the *(NGO name redacted)* not the *(nearby town name redacted)* when the animal is not pregnant. Because *(NGO name redacted)* having good quality semen for AI the animal(cow).

MH- Are they going the *(NGO name redacted)* with the cow?

LK- some time.

IP- What is the cost if you(LK) go to the*(NGO name redacted)*?

LK- RS. 100 for transportation and RS. 100 fees total RS. 200. If they came here(livestock keeper house) they charge little more RS. 150.

MH- Is it cheaper if they(member of *(NGO name redacted)*) came here?

IP to MH- Yes

IP to LK- Why you(LK) are not always told them(member of *(NGO name redacted)*) to came here, because it is cheaper?

LK- They always not available to come here(Livestock keeper house)

MH- Are they take the cow to *(NGO name redacted)* for AI (Artificial Insemination)?

LK- Yes

MH- How many time they have to *(NGO name redacted)* for AI?

LK- 2 time.

MH- How much time taken to go *(NGO name redacted)* from their home?

LK- First we call them if they available or not Then we go 1 hour for going 1 hour for returning 1 hour stay there(*(NGO name redacted)*) total 3 hour.

MH-Are they go by walking?

LK-Yes

MH-When the cow suffered from a disease last time?

LK-Long time ago I forget and sometime go the camp with their(Livestock keeper) goat,cow and poultry

IP- When the last camp occurred?

LK- Before the rainny season. 3 to 4 month ago.

MH- What would they do if the cow started diarrhea tomorrow ?

LK-They go to the *(NGO name redacted)*. We told the problem and they give medicine.

MH- Why they go the *(NGO name redacted)* ?

LK- In *(local town name redacted)* there is also a government hospital but it is fur away form here and *(NGO name redacted)* is near by the house.

MH- No other local doctor ?

LK- One lady she is Pranimitra.

IP-What is distance of Pranimitra house from here?

LK- Near about 1 km.

MH- When would they speak with the *Pranimitra*?

LK-When disease occur. Some time she came for visiting. Before the camp she came here to told them there is a camp.

MH Can you give one example of time when they talk with *Pranimitra*?

LK- 2 months ago for diarrhea of goat.

IP- What treatment Pranimitra gives?

LK- Some vitamin.

IP- Is she gives any injection?

LK- No injection only oral medication.

IP-Is the goat cure?

LK-Yes.

IP- How many days the medication going on?

LK- one white tablet is given once daily for 3 days.

IP- Are you(LK) purchase the tablet?

LK- No, Pranimitra gives the tablet.

MH-How much they charge?

LK- No charge, Free of cost

MH- she comes here every day ?

LK- No , she come once in a month.

MH-When she gives the tablet to goat

LK- 2 month ago.

MH- Who gives the tablet to the goat in each day?

LK- First day Pranimitra gives the tablet then we gives the tablet each day. Then we told the Pranimitra that the animal is cure.

MH-Who gives the tablet in day 2 and 3?

LK- We gives the tablet in day 2 and 3.

MH- why did they call the *pranimitra* instead of *(NGO name redacted)* ?

LK- *Pranimitra* house is nearby to the house hold and having some training from Govt according to them. *Pranimitra* come every month.

MH- Is Pranimitra visit all the house?

LK- Yes

MH-why did she came in every months ?

LK- Pr*animitra* came in the house, to know any animal ill in the house. She wants to know how many animals present in the house.

MH- Does she charged ?

LK-No, In the camp also no charge.

IP- When the last camp occurred?

LK- September.

MH-Do they do any regular routine treatment ?

LK-No, if the animal is ill then only the treatment is given.

MH-What was the last time the chicken was ill ?

LK-They give the chicken one dewormer for prevention of disease. Some time in poltry chalky diarrhea, chicken pox.

IP-In chalky diarrhea what you (LK) do?

LK- we call the pranimitra she gives the medicine.

MH- What type of medicine they given ?

LK-Liquid medicine 3 to 4 days in water or some time in dropper in the mouth .

MH- Would the *pranimitra* ever did AI ?

LK- No , I have not seen it. May be she performed AI in other places but not in the Infront me.

MH- Do they know about the *Pranibandhu* ?

LK- No

MH-Where were the member go if there is illness in this house ?

LK- *(local town name redacted)* hospital

MH-Is this the first place ?

LK- Yes , previously we go here and there but now a days we straight go to *(local town name redacted)* hospital.

MH-Do they ever keep any medicine here ?

LK- we have the medicine but we throw it out due to expire.

MH-Why you keep the medicine?

LK- No we are not keeping any medicine with out consulting the doctor.

MH-Is that Zincovit in window?

LK- Yes, when I am ill doctor prescribed it.

MH-Do you have any animal medicine?

LK-No

MH- ok thank you.
