# Supplementary material for: “If It Works in People, Why Not Animals?”: A Qualitative Investigation of Antibiotic Use in Smallholder Livestock Settings in Rural West Bengal, India
Source: Antibiotics (Basel). 2021 Nov 23;10(12):1433. doi: 10.3390/antibiotics10121433 (PMC8698124; doi:10.3390/antibiotics10121433)
Supplement: Supplementary file 1 [file antibiotics-10-01433-s001.zip › Supplementary S1_ Interview Transcripts/Site 2/Para-vet 2 (site 2).pdf]

Code for Study - ‘If it works in people, why not animals?’: A qualitative investigation of antibiotic use in smallholder livestock settings in rural West Bengal, India: Para-vet 2, Site 2

Interview Date: 1/13/2020

Interviewee: Para-vet (Site 2)- Antibiotic Provider

Interviewer: Mat Hennessey (MH), supported by Soumen Samanta (SS)

Transcript prepared by: Soumen Samanta (SS)

MH- Mat Hennessey

SS- Soumen Samanta

All answer (A) by SH

XXXX – NGO

YYYY – Veterinarian

ZZZZ – Senior para-vet

MH: Thank him for the interview. We understand that he is working as a paravet. It’s great.

MH: How long are you working as a Paravet for?

A: for 14 years

MH: Where did you get training to be a Paravet?

A: From this XXXX.

MH: What type of training did you receive?

A: I completed 30days training programme from here. *Redacted life history*.

MH: What type of treatment they taught you?

A: Both on theoretically and practically. We had to give written exam. We got little practical cases that time. Whatever cases came to this hospital we had to check and they showed us how to do Artificial Insemination when a cow comes into heat, what medication to give during fever and how to dress a wound etc. Thus after spending 30 days we went our places and started practising and initially society (XXXX) gave us vaccines for birds(R2B, F1 etc.), cattle (HS/BQ, FMD). People then came to know us. We told them that I have learned from XXXX Training. I asked the people

to call me if they face any problem regarding animals. I will come and see if animal is affected with fever. If I cannot solve the problem, I used to call YYYY and took his advice and accordingly treated the animals. Thus 14 years passed away.

MH: Do farm contact him directly?

SS: Do you visit any farm?

A: No such large farm here now. One was under the [project name redacted] project. But I know people having 40-50 goats, they cultivate grass for the animals. And some pig farms having 10 to 12 pigs.Previously kept more in number but due to feed scarcity they reduced the number. Some poultry farms are also there. I treat them sometimes.

MH: What type of problems with the animals seen when the farmers call you?

A: Like goat is having diarrhoea, fever, foot wounds, water accumulation in head (Gid, a parasitic disease), cyst. Generally I do not do Artificial insemination in cows, *Pranibandhu* do these AI. Uterine wash of goats, cows, also during delivery(calving time) they call me.

MH: What type of medication do you use?

A: Gentamicin, Oxytetracycline, Amoxycillin, binocin (ampicillin/cloxacillin), Penicillin, Sulphadimidine, Atropine sulphate, meriquin powder(enrofloxacin), tylan powder(tylocin), oxytetracycline powder for poultry. Antihistamines like chloril(chlorpheneramine).

MH: Among these which drug you use most?

A: Enrofloxacin, Gentamicin, Oxytetracycline, Ampicillin/cloxacillin.

MH: In what condition you use these for?

SS: How do you decide which medicine to give when?

A: Like in fever, after using Gentamicin and chlorpheneramine if fever is not geting down then I use enrofloxacin if not works then Ampicillin/cloxacillin. I use first one for 3 to 5 days if not cured then i go the second drug for 3 days.

In last HS/BQ outbreak I used ampicillin/cloxacillin and chlorpheniramine, it worked better.

MH: In diarrhoea cases what drug do you use?

A: Human Norfloxacin/tinidazole, Sulphadimidine bolus.

MH: Where did you learn which antibiotics to be used?

A: From XXXX.

MH: How did you learn from them?

A: Plus I have read some books which YYYY gave us, also I have read some other books as well. And training from doctor.

MH: Would you ask for doctor's advice?

A: Yes. Also from previously posted doctor, from the doctor who came to train us. And have phone number of medical representatives (like [pharmaceutical company name redacted) also, they sometime comes and tell that this new medicine works better in this condition. And I used that in field condition, if it works I use it afterword.

MH: How would you interact with medical representatives?

SS: How do they come to you?

A: I am having phone number of them (like [name redacted] from [pharmaceutical company name redacted]). Sometimes I call him or he calls me. Whenever I am short of medicine, if I call him he tells that it could be available in this shop of [nearby town name redacted]. The medicine which they supply from Kolkata to these shops.

MH: How often you meet with them?

A: Conversation is going on regularly. In a month once/twice they call and ask how my practice is going. Also ask if their medicine is unavailable what I do as alternate. I tell them this one of this company I use. They come 2-3 times in a month.

MH: How many different representative do you interact with?

A: 3. [pharmaceutical company names redacted].

MH: In your daily work how many times do you use antibiotics?

SS: How many cases do you see daily?

A: On an average 10-15.

SS: Among them in how many cases do you use antibiotics?

A: Total 5 to 6.

MH: Among these 5 cases, which antibiotic is most commonly used?

A: Gentamicin and Enrofloxacin.

MH: How often do you use human antibiotic Norflox-TZ?

A: In diarrhoea cases only I use Norflox TZ. Along with this some anti-bloat medicine I use.

Dr. Indranil: Why you are using human Norflox-TZ for animals?

A: Once I used Sulpha drug for diarrhoea but there abortion happened. So I stopped using sulpha drug in diarrhoea cases. And no veterinary formulation of Norflox-TZ is available.

MH: Do you use any other human antibiotics?

A: No. And I use human Paracetamol and Meloxicam injection.

MH: Where did you get these antibiotics from?

A: From [nearby town name redacted} and from this XXXX Model.

Dr. Indranil: From which shop of [nearby town name redacted] do you buy these antibiotics?

A: [drug shop names redacted- human drug shops with veterinary sections]

MH: Do you get antibiotics from any other places except these three?

A: Yes, that Medical representative. He comes to [nearby town name redacted], stay there in a hotel for 1-2 days. I go and take from him what I want.

MH: From these 4 sources where do you get antibiotics most?

A: From this XXXX.

MH: What % do you get from here (XXXX)?

A: All antibiotics are not available at XXXX all time. Which I do not get from here I buy it from other sources.

MH: Why do you come here first?

A: As doctor comes here, Doctor's advice can be taken. And I learned from here that's why I come here first. Here only vet medicines are available. But in those shop both human and animal medicines are available.

MH: If you buy from these shops instead of XXXX, do you get any economic benefit?

A: In shop it is cheaper for me to buy.

MH: What about from medical representative?

A: It is most cheap. He also supply in [nearby town name redacted] shop, as I directly buy from him it is most cost effective.

MH: Is there any reason why he not get all the medicine directly from the M.R?

SS: Then why don't you take all the medicine from that M.R?

A: Yes, I am taking medicines from him since last two years.

MH: Do you still buy mostly from XXXX?

SS: You said mostly you buy from M.R now, do you buy from XXXX?

A: It is decreasing slowly. And slowly getting more known to him (M.R).

MH: Why it is decreasing?

SS: As when he call them(M.R) they come to home with medicine.

MH: At this moment, what % of antibiotics do you get from each place?

A: Antibiotics mostly get from [nearby town name redacted] and XXXX not from M.R. M.R generally supply other medicines like vitamins, fertility medicine etc. Antibiotics are mostly taken from XXXX.

SS: Does the M.R don't supply any antibiotics at all?

A: They can supply if I tell them. We see which antibiotic works better. But the antibiotics he offers usually I don't take it. As it don't work properly. Some antibiotic of some special company works better so I refuse.

MH: So what type of antibiotic should not be working properly?

SS: So which medicines of [pharmaceutical company name redacted] do not work properly?

A: Few days ago that M.R offered one antibiotic (I could not remember the name now) but I refused as the antibiotic gentamicin, ampicillin which I get from the XXXX model works better. I take those calcium supplements, vitamins, liver tonic from him. Generally don't take antibiotics.

(YYYY leaves)

MH: So when do you buy antibiotics from [nearby town name redacted] shops?

A: Weekly once or twice. Now a days XXXX model is open in office time only (11a.m. to 4p.m.). So if I need any medicine at off time then I have to go to [nearby town name redacted]. As those shops are open and anytime you can buy from there.

MH: When you buy medicine from these shops, do you pay them by cash or credit?

A: When I am having money I pay otherwise there is credit system which I pay later. There is one khata where my name is entered and they write the due amount.

MH: Does it same for both XXXX and [nearby town name redacted] shop?

A: No, in XXXX I have to pay cash always during buying.

MH: How long later you can deposit your due money in those shops?

A: Maximum one year.

MH: Is it more common for you to buy in cash or credit?

SS: Which one have advantage? With cash or credit.

A: advantage in all, if I am not having money in hand I go for credit purchase.

MH: Do they offer you any incentive to buy medicine?

SS: Do they give you any benefit? Or any discount? Or how much discount?

A: In case of antibiotics 15% off and in case of other supplements upto 25% off.

MH: How do you receive that 15%?

A: It's like the discount.

MH: Do they give you any gifts?

A: Previously they used to give (clothes, shoes etc.) but after implementation of GST, they don't give any gifts.

MH: How many times do you come here (XXXX) to buy medicine in a week ?

A: 1-2 times in a week.

MH: How many times do you go to the [nearby town name redacted] medicine shop in a week?

A: 2-3 times in a week.

MH: Do you have any preference to go in between these two shops?

A: [human drug shop with a veterinary section name redacted]. As there more veterinary medicines are available.

MH/SS: What type of animals do you treat?

A: Cows, goats, poultry, pigs, buffaloes and dogs.

Q: Among these, in which species do you use antibiotics most frequently?
A: Cows, sheep, goats and buffalo.

SS: Do you not use antibiotics in poultry?

A: I generally do not do much. Very less. I do not have any chamber, I travel in field by motorcycle.

MH: Do you visit any commercial/large farm here?

A: Here large mean people having 20-25 goats. I know such one person whom I visit.

Dr. Indranil: Where it is? Do you have phone number?

A: (inaudible), no his phone number is not here.

MH: What are the 2-3 most reasons for giving antibiotics to the cows?

A: In fever, cough and cold and golafola (HS/BQ) and diarrhoea.

Q: And in case of goats what are the reasons?

A: same, diarrhoea, fever, cough and cold.

MH: In case of buffalo what are the reasons?

A: Mostly diarrhoea and fever.

MH: You said about Norflox-TZ, where do you get that from?

A: From [nearby town name redacted] shops.

MH: Do you get from here (XXXX)?

A: No, not from here.

SS: From which shop?

A: From both shops.

MH: When he goes to visit the cases, how do they contact him?

SS: When you go to see the cases, how do they contact you? By phone?

A: Hmm (yes).

MH: Do you always go to the house of the farmers to treat animals?

A: Yes, I always go to their home.

MH: How much do you charge for each case?

A: It depends on the case type.100, 140, 50, 60, 70, it's not fixed, depends on the case.

SS: For cows how much do you take?

A: For cows now-a-days 100-150 rupees minimum.

SS: Including injections?

A: Yes. Here poor farmers are more, if I charge more they will not call me next time. So I have to maintain all side.

SS: In case of goats?

A: In case of goats I take 40-50rupees.

MH: Is that charge without medication?

SS: Is it like doctor fees or medicine cost?

A: No, If you says it's doctor fees, nobody will give you that much. You have to consider as medication.

MH: So this would be the total cost in visit.

SS: Yes.

MH: Do you charge the same on the next days?

A: Like that way, it depends on the medicine that I am giving. Like if i go on first day I took 100-150 rupees, in the 2nd time there is no need of antibiotics, or need of other medicine, it is within 50-100 rupees.

SS: If you give antibiotic on first day...

A: I generally do not give antibiotics on the first day. If it is not curing with other medicines (like in fever by antipyretic medicine) then I give antibiotics.

SS: How long do you give antibiotic then?

A: Minimum 3-5days.

SS: Then each time do they pay you 100-150 rupees?

A: No, on first day 150 rupees, then100 rupees and then 70 rupees. I complete it within 200-300 rupees in 3-4days.

MH: What do you do when the animal is not getting better with the treatments?

SS: What do you do when animal is not curing with medicines?

A: 'Not curing' like never happened still for me.

SS: Then do you consult doctor?

A: Last time one "*golafola*" case (swollen throat) I had seen, at last I sent it to the XXXX model hospital. Also they saw it. Sometimes '*je jabar se jabe, kichu korar thakena*'(who is going to die will die, you cannot do anything). Sometimes I consult the doctor, but it generally dies.

SS: Do the doctor not go to see the cases?

A: Doctor tell give this this medicine and see. If not cures by it, nothing can be done.

MH: Do you have contact with other paravets in this area?

A: In this area ZZZZ (salutation to senior), me and one new *paravet* is there.

SS: How long?

A: 4-5years.

SS: Can we get his numbers?

A: I have their phone numbers.

MH: What area do you work in?

A: [nearby town and village names redacted- 4 different areas]

MH: Do you work in [village name in site 2 redacted]?

A: Yes. [village name redacted] is a near place.

MH: Do you work with any *Pranibandhu*/*Pranimitra*?

A: No.

MH: We are looking about how antibiotics use can be improved. Do you look any ways that antibiotic use could be improved?

SS: Do you ever think how antibiotic use can be improved?

A: No, initially I got fear/hesitate to use antibiotics but slowly after practising I understand that which antibiotics works in which condition. Not get fear now.

SS: Do you think this 3-5days are okay?

A: Generally 3-5days antibiotics use work.

SS: If an animal get cured in 3days then do you go for 5days?

A: No, not. If it is not totally cured in 3 days then I go for total 5days.

MH: does he have any reason why the antibiotic would stop working?

A: Do you have any idea why antibiotics are not working?

A: No, have no idea. Then I call doctor why this medicine is not working.

SS: Then what the doctor say?

A: saline, etc what he tells I follow. Then if not works, then nothing can be done.

MH: How would affect this business/income if he is not able to get antibiotics?

SS: Suppose you are not getting antibiotics for use, how your income would get affected?

A: There will be a problem. But new company will come with new medicine as alternate. Govt. must take some step. I have seen the use of antibiotic from my grandparent's time. If antibiotic use is not there income will get down.

MH: So how would affect his income if he has not access to use antibiotics?

SS: Does your income will get down or?

A: Income will go down. If not using antibiotics in cows and goats, there is no confidence whether it will cure or not. If I use antibiotic I am confident that animal must get well. If I not use, it would feels bad in my mind.

SS: Will your name be defamed when after treating without antibiotic the animal is not curing?

A: Yes.

MH: So big/small effect will be there on your income?

A: Big effect.

MH: How many times in each day do you use those human antibiotics?

SS: How many times you use that Norflox-Tz daily?

A: Daily 2-3 strips are sold (20-25numbers of tablet).

SS: How many diarrhoea cases?

A: Daily 3-4.

SS: how much medicine do you give?

A: I give medicine for 3days at a time.

MH: What do you think about different qualities of antibiotics that you have?

SS: You use these antibiotics, which one seems best?

A: Binocin, gentamicin and enrofloxacin.

MH: Why do you think it is of best qualities?

A: These antibiotics cure animals, are working properly here.

MH: Do you have any idea which company gives the good qualities antibiotics?

A: Previously [pharmaceutical company name redacted] was good. I am using [pharmaceutical company name redacted] enrofloxacin now.

MH: Is it good quality or bad quality?

SS: Why good quality?

A: Good means working well. These 3 companies antibiotics run here, reach model or [nearby town name redacted].

MH: Where do you get the [pharmaceutical company name redacted] antibiotics from?

SS: where do you buy [pharmaceutical company name redacted] antibiotics?

A: Previously from XXXX model. Sometimes they keep, sometimes not. Previously they (company) used to arrange seminar 2-3/year but now it’s not happening. [nearby town name redacted] [drug shop names with human and veterinary sections redacted] shops keep now.

MH: Do you have any question to ask before we finish?

A: No
