# Supplementary material for: “If It Works in People, Why Not Animals?”: A Qualitative Investigation of Antibiotic Use in Smallholder Livestock Settings in Rural West Bengal, India
Source: Antibiotics (Basel). 2021 Nov 23;10(12):1433. doi: 10.3390/antibiotics10121433 (PMC8698124; doi:10.3390/antibiotics10121433)
Supplement: Supplementary file 1 [file antibiotics-10-01433-s001.zip › Supplementary S1_ Interview Transcripts/Site 2/Para-vet 3 (site 2).pdf]

Code for Study - ‘If it works in people, why not animals?’: A qualitative investigation of antibiotic use in smallholder livestock settings in rural West Bengal, India: Para-vet 3, Site 2

Interview Date: 1/13/2020

Interviewee: Para-vet 3 Site 2, Antibiotic Provider

Interviewer: Mat Hennessey (MH), supported by Soumen Samanta (SS). Dr Indranil Samanta is also present

Transcript prepared by: Soumen Samanta (SS)

MH- Mat Hennessey

SS- Soumen Samanta

Dr. Indranil- Dr Indranil Samanta

All answer (A) by paravet (SH)

XXXX – NGO

Dr. Indranil: Give us your introduction first?

A: *Redacted life history/*

MH: When did you take your training here?

A: When I did training, what to say, I was reading in 11-12, so young age. In the vacation we get, I took the training then. Then I started practising.

MH: The other training he did, how long ago was that?

SS: How long back you took training in [village name redacted]?

A: [life history redacted].

MH: What area do you work in?

Q: My project area is [village name redacted] but I practise not only in this place but also in total [other village names redacted]... everywhere. But main work is in this (two place), project work, but I have to go outside of that also.

SS: How much block/area?

A: Mainly [block names redacted], these areas.

MH: Within [gp name redacted- site 2], which he tells most interested in, what type of animals does he deal with?

SS: In [gp name redacted- site 2], on what type of animals do you practise?

A: Goat, sheep, cattle, poultry, duck. Many poultry farms are here, mostly their livelihood on poultry farms.

MH: Which of these do you see most commonly?

A: I have to see all, poultry cases are many, then goat, these two mostly come.

MH: How many cases do you see each day?

A: On an average 20-25.

MH: Mostly poultry?

SS: Mostly goat and poultry, right?

A: Yes.

MH: What are common type of disease and conditions he see each day?

SS: When people call you or what are the problems do you see each days?

A: The disease of cattle, sheep or goat what happens here, depends a little bit on season. In this winter season mainly "*golafola*"(bottle jaw), diarrhoea, 'sordi' (cough and cold), bloat, anaemia also occur much here. Just before rainy season poisoning cases come also.

MH: What about the poultry?

A: This time coccidian comes much, then ranikhet, gumboro.

SS: In ranikhet, do the birds die?

A: In ranikhet, after vaccination if we give a mix of gentamicin-levamisole-distill water punch it works better, we have seen. With this we use antibiotics. If we give this then it works well otherwise by only giving antibiotics, medicine we can not cure that much.

With this we give Supercox powder and a vitamin.

MH: What type of medication does he give for these conditions?

SS: What type of medication do you give in cases like fever, diarrhoea?

A: In fever, I use meloxicam- paracetamol group medicine. With that antihistaminic is also given. With this antibiotics are used but not always. Mainly try to cover without antibiotics. According to disease, Binocin® (Ampicillin/cloxacillin) or in cough and cold mainly I give meloxicam, in cold meloxicam-paracetamol works well. In cold, each antibiotic works in different time.

SS: In case of cough cold meloxim-paracetamol works, is there no need to give Binocin like medicine?

A: No. If ‘sordi’(nasal discharge in cough and cold) is there, I use meloxicam, meloxicam with paracetamol.

SS: Binocin?

A: Don’t try to use that. If it is not curing then I use otherwise not.

MH: How long afterwards?

SS: After how many days you use antibiotics?

A: 2-3 days.

MH: What type of antibiotics do you use?

A: Binocin (Ampicillin/cloxacillin), Meriquin (enrofloxacin).

MH: What do use in diarrhoea?

A: Sulphadimidine, Sulphatrim, Sulcoprim bolus, Duaprim bolus.

MH: And in bottle jaw cases?

SS: In ‘golafola’ case?

A: In ‘golafola’ case I use *Redema* tablet, *lasix*; with this Pentamox®(*amoxicillin)*(If not reduced) if needed. Or see by giving B-complex first. Give *redema* with B-complex; by it get reduced. If not reduced then use antibiotic. (some repeated part)

MH: After how long?

SS: You said 2-3days after right?

A: See for 2days.

SS: In bloat cases?

A: Atropine sulphate. And antihistaminic if more gas is formed, *Gasnil* powder, *bloatosil* syrup. Sometimes after trochariztion *bloatosil* is pushed in rumen. And fed *gasnil* after warming it.

MH: Do you use antibiotic in bloat?

A: Yes, antibiotic needed.

SS: What antibiotic?

A: In bloat *sulfa* group antibiotic is given.

MH: Is that straight way or after a period of time?

SS: Do you give this on the same day?

A: No, in every case we see a little (time) by *gasnil* powder or *bloatosil* syrup and *lasix*; If we do this then chance of increase (worsening) is very less.

SS: Reduce?

A: Reduce. Hardly use an antibiotic, very less. We try as much as we can avoid antibiotic, Antibiotic has to be given but not give because if I give antibiotic I have to go for it for 3-5days. Our tendency in villages is that if animal get little cured in 1-2 days then no need of doctor (next). Means I am rearing an animal, there is a cost involved in it, if I see it get reduced on first day then I myself will tell that doctor babu my cow is well now, no need to come. Then going there will be awkward (situation).

SS: Does it happen anytime that you gave antibiotic but on the next days they are not calling you?

A: No, no. When we give antibiotic we have a pressure (responsibility) then we tell them today I am giving injection and I will come for 2 to 4 more days like that. When I see it is light case, will cure in 2-3days, there, if you see you can understand how quickly it will recover.

MH: Out of 20-25 cases you see each days, in how many cases you use antibiotics?

A: If I see 20 cases, 10 cases. Fifty fifty.

MH: Where did you get the antibiotics from?

A: In our [nearby town name redacted] bazaar medical shops [names of two drug shops redacted with veterinary sections] and in case of poultry [name of poultry shop redacted] shop.

SS: What antibiotic do you get from bapigoal?

A: *Suldin*, *Meriquin, Enrocin* etc. [poultry shop name redacted] keeps each and everything of poultry. Like feed medicines etc. For other animals the medicine you can get from [names of two drug shops redacted with veterinary sections].

MH: Do you get antibiotics from other anywhere else?

A: No.

SS: What do you take from XXXX model?

A: Yes, from here we take it. It is our nearby place. If it is not available at XXXX then it is taken from market. First it is taken from here.

SS: Do any medical representative come?

A: No, not like that.

MH: Why is this place your first preference?

A: As I learned from here and it is a society. We believe a society never keep bad quality things. And as I learned from here we have a responsibility for it. If 2 rupees is profit then it should be of society. Society is for all.

MH: What would make you to go to the shops?

A: Not all medicines are available at XXXX. If I need a medicine instantly if I not get from here now then I go to those shops.

MH: Do you have any preference in between [names of two drug shops with veterinary sections redacted]?

SS: Do you have any preference? Which one is better?

A: No, same.

MH: What is the proportion of buying from here and here of antibiotics?

SS: Among XXXX model and [nearby town name redacted] shops, if you buy total 100 rupees antibiotic, how much from XXXX and how much from [nearby town name redacted] shop?

A: Here (XXXX) 80%, rest 20% from those two shops.

MH: Does it more cheaper in XXXX?

A: No.

MH: Does it more expensive than shops?

A: I see, some medicine you get at less and some medicine at high price. 2-5rupees difference. Then I take it from XXXX.

MH: Do you get the medicine here by paying cash or credit?

A: By cash, by cash (at XXXX). In [nearby town name redacted] it can be in credit. But here you cannot do it in credit.

MH: With what period of time you have to pay the cash in [nearby town name redacted]?

A: If I take today, one year after 'halkhata'; There is a thing called '*halkhata*'(a sort of Bengali festival during new year when all of the dues are cleared in a shop by the customers).

MH: Do they give any incentives?

SS: Do they give you any incentives or like discount or anything?

A: hmm (yes).

SS: How much?

A: Not same for all medicine, in one 20%, another 10%, 5%.

SS: How much for antibiotics?

A: 5-10%. Not same for all antibiotics. Average 10% you can assume.

MH: If you think all types of medicine he uses how, what proportion of those medicines are antibiotic?

Q: If you use total 5 medicines, how much is antibiotic?

A: In 5, it is one. One is antibiotic.

SS: What is the %? How much medicines do you use daily?

A: Daily many, 20-22.

SS: Among them how much is antibiotics?

A: 5-7.

MH: So it is about 25%.

MH: What does he think of how antibiotics are used in this area?

SS: In your area how antibiotics are used? What do you think about it? Do others also use like you or..

A: Practice varies among peoples, not same for all, someone think that if you give antibiotic first then it will recover quickly. I think it is good to treat without antibiotics first. Means if you cure without antibiotics it is good.

SS: Why do you think so?

A: Due to improper use of antibiotics it is getting resistant, I think we are going towards loss. It is seen that the antibiotic course is not completed in most of the times.

SS: Not becoming possible?

A: Yes.

SS: Does it happen to you also?

A: Yes, that is why I am slowly..

SS: Going towards the path that you told?

A: Yes, yes.

Let me tell, if I see total 20-25 cases, 8-10 are of fever. Mostly fever comes. In that case after giving meloxicam paracetamol and antihistaminic the patient gets well.

SS: No need of antibiotics?

A: No need of antibiotics. So why we will use antibiotic in that case?

SS: In which condition the 3-5days course cannot be completed?

A: Sometimes it is our fault;

SS: You are not giving time?

A: In any cause we aren't able to give time. After promising to go, are being not able to go, problems in patient's home to restrain animals. If large animal is there, in first day they manage 4-5people to restrain for giving injection but on 2nd day are not able to do that.

SS: You have to return?

A: Yes, yes.

MH: How much do you charge to treat an animal?

A: It depends on case and farmer's economic condition. I am giving an example, after treating fever in a goat, in one home I take 100rupees and also in another home I take 40rupees by giving same medicine. If a farmer's economic condition is bad I just take the medicine cost and transport cost. When I see the farmer can give more I take more.
SS: In case of large animal? Like in cows?

A: In fever, 100-110rupees.

SS: If it is a ‘valo party’ (economically good owner)?

A: If I have to go far, and a ‘valo party’ then 150-200 rupees.

MH: What do you do when after giving this treatment the animal is not getting better?

SS: Suppose your treatment is not working, the animal is not getting better, then what do you do? Your antibiotic treatment is not working.

A: Have to change the antibiotics, but sir in that time if it is weak animal by giving saline and with it if you give antibiotic it works better that I have seen.

SS: By same antibiotic?

A: No, change the antibiotic.

MH: And if they don’t get better after that?

A: It is very less that animal is not recovering, that I have seen. One thing, after so many year treatments, I think if I can give time on animal properly, if I have time and if ‘party’ is good (valo party) the animal must get cured if it is not in too bad condition.

MH: What type of antibiotics would he use next?

SS: Like in fever you give Binocin® (ampicillin/cloxacillin), if it is not working what is next?

A: If the owner is valo (rich/can afford) I give Clavum®(amoxicillin/clavulanic acid). (a costly antibiotic),{clavum is a human antibiotic}

MH: Where do you get this medicine from?

A: From [nearby town name redacted], here that is not available.

SS: It’s not veterinary antibiotic.

A: I have seen some human medicine works very much in animal.

SS: Like Clavum?

A: Clavum is 1 number, then Taxim (Cefotaxime), in dog cats

Dr. Indranil: Does these work in cattle also?

A: I have seen in mastitis my medicine is not working but clavum works.

Dr. Indranil: What do you think why it is happening?

A: Quality of human antibiotics is better.

MH: Why do you think that?

A: I see that when animal antibiotic is not working but human antibiotic works.

MH: Why do you are not use human antibiotic first?

A: Human antibiotic is more costly. I have to see my one goat price is 1000rupees but if I make a bill of 500-700 for treatment then the owner himself next time will say that doctor do not come next time.

MH: Does he use any other human antibiotics in addition to these two?

SS: Besides Taxim and Clavum, any other?

A: These two I use.

SS: Do you use norflox-TZ?

A: We are also having norflox-TZ, in human and veterinary also having TZ, but human norflox-TZ I use for poultry.

MH: For what condition?

SS: For what condition you use in poultry?

A: Diarrhoea of poultry.

MH: Does he use this in first time case of diarrhoea in poultry? This Norflox-TZ, does he use that immediately?

SS: If you see poultry diarrhoea, do you use this immediately?

A: No, no. In diarrhoea we use it in two ways. In case of farm I don't give it (human TZ) generally, in them I give veterinary medicine (TZ) as its cost is less. Bill is less. And here cock fighting occurs more, in that case the owner want to cure at any cost (can spend up to 50rupees/bird).

MH: When you treat diarrhoea in poultry, you just check the diarrhoea of that particular bird or all of the poultry?

A: For total birds, given in water. In farm it is seen that if one is ill today, 10 will be ill on next day if not treated all.

MH: Out of your 20-25cases in 10 cases when you use antibiotics how many have human antibiotics?

A: Maximum one.

MH: Where did you learn to use different antibiotics in different condition?

A: From the training I took here, and now I have to go to university every month. And university Sir (teachers) sometimes make discussion with us; And when people like me who are practising, project are going on 4-5places, get together then we exchange our views, I say mine, they say theirs, thus.

MH: Does he ever, as he said, going to inject antibiotics to an animal, do any of the farmers ask to buy antibiotic themselves from somewhere else?

SS: When you are going to treat the animals, and use antibiotics, if you have antibiotic you give, right? If you do not have antibiotics then do you ask the farmer to buy antibiotics themselves?

A: I prescribe it.

MH: How often does it happen?

A: Very less, daily one case. Otherwise if animal is ill, animal condition gets worse when he buy, then in 1-2days the animal get more illness.

(some interruption)

MH: If it is hard to get the antibiotic, if he could not buy from the shops, how much impact that would have on his job/income?

SS: If you are not allowed to use antibiotic from the shop

A: If not allowed to use antibiotics I think not much effect would be upon my income.

SS: In that it will get cure?

A: Yes, I think. A little effect will be upon me.

Dr. Indranil: It will not affect his income.

MH: How do you think what type of intervention could be used to improve antibiotics use in your area?

A: Actually antibiotic shouldn't be used unless it is very much needed.

Q: How would that be communicated to the people?

A: We have to make people understand. I or people like me, we are making the people understand in that way how we want for ourselves. Isn't it? It is competitive market. If a patient gets cured by his treatment in one day then I also have to cure the animal in one day.

SS: Using antibiotics?

A: Have to use antibiotics. So in that place if we try to make the people understand that antibiotic is damaging us, so avoid antibiotics. Without antibiotics animal can be cured and it will be your gain. The case which will cure in one day by using antibiotics, it will be cured in 3 days without using antibiotics. But you will be profitable. This we have to make people understand.

MH: Does he think ever happen people will take animal antibiotics?

SS: Do you think veterinary antibiotics are used in human?

A: No, veterinary antibiotic, it is written there that ‘not for human use’. There is no question of giving.

MH: Do you have any question to ask to us?

A: My question is, you came that’s why something I came to know. Some people with you came before and asked. It’s my pleasure that you came. Let’s see what can be done. How antibiotic use can be stopped.

SS: not to totally stop, we want how we can or by any regulations..

A:In case of poultry it is very much necessary to use antibiotic, in case of animal it is ok but in poultry without antibiotic it is very hard to stop the mortality. It is like that antibiotic has to be used everyday.

SS: What are those antibiotics?

A: In poultry supercox powder, metaprim, suldin, tylon, given in water; given in particular course but poultry is getting so much ill that antibiotic has to be given. Today we are giving the antibiotic and tomorrow it is sold, we are eating. And we are giving it knowingly or unknowingly.

MH: In poultry are they using antibiotic routinely?

SS: Daily.

MH: Are add to the food?

SS: Water.

MH: What type of drug?

SS: Sulfa group,

A: supercox, meriquin, enrofloxacin,

MH: everyday for 35days?

SS: for the whole 35days?

A: yes, for 35days. It is given in a particular course of 3-5days but it is seen that after maintaining that course also many disease arise. Like today bloody stool, on the next day watery diarrhoea the next day cough and could. Some farms are okay but in some farm if one disease decreasing then another one arising.

SS: So as preventive do they use?

A: Yes, (laughs), this kind of thing is happening.

MH: Are they using so far sulfa, enrofloxacin and tylocin all three in whole time?

SS: These all 3 antibiotics do use whole time like in 10days, 10days?

A: Not 10days each, if it is cured in 3days then no chance of giving again.

SS: you told as preventive measure that is given in water of feed. Which are those?

A: that is given for 30-35days, this is given for 3days, we must use antibiotic in 3 days(14th ,15th ,16th).

SS: Which one?

A: Supercox powder. In that age mainly cough and cold arise.

SS: And that enrofloxacin?

A: Besides this 14, 15, 16th day we are using antibiotic when birds get ill. Sometimes it is seen that this problems, in some farms it is seen once it get affected it continues up to its marketing age.

SS: In that case enrofloxacin is used all the time?

A: No, change is done. If it is checked it's ok but it is not checked (Reduced/cured) then we change the antibiotic again.

SS: If disease persists then

A: If persists then according to the type of disease we use different antibiotics.

SS: One antibiotic is not for all the time. Isn't it?

A: No, no.

MH: They are generally getting one antibiotic at least each day.

SS: (inaudible)

SS: It is given in food/water right?

A: yes.

SS: How many these type of farms do you visit?

A: 20-25.

SS: How much is their capacity?

A: 50-500.

SS: In every farm this antibiotic practise is there?

A: In some farms it is seen that without using antibiotic it is completed.

SS: No antibiotic at all?

A: Those 3days only. And 3 vaccines also we do.

MH: Where do you get the antibiotics from?

A: [poultry shop name redacted] and XXXX.

MH: Do they always use antibiotics in water or sometimes in food?

A: Always in water. Almost all poultry medicines are given with water.

MH: Do the poultry farms get medicine directly from the shop themselves?

A: No, they consult me first.

MH: So he buys antibiotic.

SS: So you buys only?

A: Yes.
