# Supplementary material for: “If It Works in People, Why Not Animals?”: A Qualitative Investigation of Antibiotic Use in Smallholder Livestock Settings in Rural West Bengal, India
Source: Antibiotics (Basel). 2021 Nov 23;10(12):1433. doi: 10.3390/antibiotics10121433 (PMC8698124; doi:10.3390/antibiotics10121433)
Supplement: Supplementary file 1 [file antibiotics-10-01433-s001.zip › Supplementary S1_ Interview Transcripts/Site 2/Public-private VPP 3 (site 2).pdf]

Code for Study - ‘If it works in people, why not animals?’: A qualitative investigation of antibiotic use in smallholder livestock settings in rural West Bengal, India: public-private VPP 3, Site 2

Interview Date: 1/16/2020

Location: Site 2
Interviewee: public-private vpp 3 Pranibandhu- Antibiotic Provider

Interviewer: Mat Hennessey (MH), supported Indrajit Patra (IJ)

Transcript prepared by: Indrajit Patra (IJ)

MH-Mat Hennessey

SH-Stake holder (pranibandhu)

IP-Indrajit Patra

IP- What is the name of this village ?

SH told to IP-This is the part of [village name redacted] and his (Pranibandhu) name is [name redacted]

MH- Can you ask what is his designation?

SH told to IP- He is a Pranibandhu in [GP name redacted- Site 2] GP. Redacted life history.

MH- Who did with you in pranibandhu training ?

SH told to IP- He got training with 14 other people and he was selected by the interview. The 14 people got training in two different places

MH-How the training taking for ?

SH told to IP- 5months of training , Government has to provide job if 6 months training is conducted

MH- what did he learn from the training ?

SH told to IP- During the training period he taught about the AI (artificial insemination), treatment of fever

,Black Quarter , Anaemia, FMD ,parasite and every type of first aids

MH- What type of first aids ?

SH told to IP- How to identify and diagnose the disease like BQ

MH- Can you give example how they taught how to diagnose the disease ?

SH told to IP - He found the oedema and feels the oedema and how to feel the swollen area,whether any sound is coming from swollen area

MH- What type of swollen?

SH told to IP- In BQ there is swollen and he told that there is the cracking sound present or not. Then the BQ portion they cut and then they tamponing the iodine and they use the antibiotic

MH- How was he taught about antibiotic?

SH- Antibiotic was Dicrysticine , Penicillin

MH- who toldhim about the antibiotic ?

SH told to IP- He told that he discuss about antibiotic with the teacher during the training period. After that we discussed in the centre with our 'Sir's what type of antibiotics should we use in gram-positive and gram-negative infection. Penicillum notatum is the main source of antibiotic.

MH- Which training he known about antibiotics?

SH told to IP- In the 6 months training and the teacher also told that Penicillum notatum is the main source of the antibiotic

MH- How to use the antibiotic ?

SH told to IP- He told that he first think about the body weight and then the calculate the dose and its use in twice daily .

MH- What he is talking about gram positive and gram negative bacteria?

SH told to IP- The gram positive and gram negative bacteria always multiply and are killed by penicillin as told by our teachers

MH- Does he use any other type of antibiotic ?

SH told to IP-Yes

MH- What type of antibiotics?

SH told to IP-Penicillin works well. Oxytetracycline is other antibiotic told by their sir

MH-What is the use of oxytetracycline ?

SH told to IP- He gives according to the body weight

MH-Could he gives an example of when he would use oxytetracycline ?

SH told to IP- The oxytetracycline is used in the case of fever.But he came to know that in oxytetracycline there is some problem like irritation, shock; so now he does not use oxytetracycline and uses other antibiotics like ampicillin

MH- what type of animal he is talking about the irritation, shock and fever ?

SH told to IP- In case of calf and goat the irritation and shock in animal and now he is shifted in the ampicillin and enrocin (enrofloxacin) instead of the oxytetracycline

MH-Why he is using these drugs instead of oxytetracycline?

SH told to IP-Because when he go to the treatment purpose then he found that oxytetracycline is not working after prolonged use and its used by the another Pranibandhu, quack and he did not get any result of oxytetracycline then he use the ampicillin and enrofloxacin when the oxytetracyclin is not working in the animal

MH- Can he give an example when the oxytetracycline is not working in the last time ?

SH told to IP- when there is panting, coughing, allergy in cows we started to use ampicillin With his own experience he came to know that oxytetracycline not working. This year lumpy skin disease (LSD) came here, 2-3 cows died with soothing diarrhoea. We could not save the animals.

MH-What is the difference between Pranibandhu and quack?

SH told to IP-the Pranibandhu they get training from the PBGS govt. and the quack they don't have any training from Govt

MH- Who taught him about enrofloxacin and ampicillin?

SH told to IP-He taught from the veterinary surgeon the doctor came in [village name redacted] they told them to use of antibiotics like ampicillin and enrofloxacin and in his own experience he found that this antibiotic is good

MH-Does he know any other quacks in [village name redacted] and does he have contact with them and talk to them ?

SH told to IP-Yes

MH- What type of talk with them?

SH told to IP- In every case they discuss with the VS with quack and they treat the animal.

MH- When does he is speak with the quack ?

SH told to IP-When Quacks are ask to him about the disease and he told them how to treat the disease

MH-Does he gives advice the quack ?

SH told to IP- Yes

MH-Does he know any other Pranibandhu is here and how he is contact with them ?

SH told to IP-In [block name redacted] block there 14 GP so 14 Pranibandhu is working and he has contact with them, I am in [gp name redacted-site 2]

MH- how they have contact with each other ?

SH told to IP-By the telephone and in every Friday all the Pranibandhu of the Block meets in the BLDO office

MH- what they discuss among the Pranibandhu ?

SH told to IP- They are going for the AI straw in every Friday and they having discussing about a disease when facing problem they discuss with the VO otherwise they go and came with the AI straw

MH- What type of advice given by him to the quack?

SH told to IP- 1-2 quacks may ask my advice. Quacks are taking for the advice of BQ, ROP (retention of placenta) and dysentery if not cured .

MH- Dysentery in which animal?

SH told to IP -In any cow

MH-Where he go now?

SH told to IP-He is now going for one ROP case in cow

MH- Who take advise from him ?

SH told to IP- 3 to 4 quacks

MH-Where the quack came from?

SH told to IP-In near by villages

MH- what advise he gives to those quacks ?

SH told to IP-In ROP case he advise to wash with the potassium permanganate and then orally give medicine (Uterofresh) and if there is no work with the medicine and potassium permanganate wash .In dysentery case injection of chromostat and Saldin (oral) and DNS saline was given.Sulphadimidine bolus are advised in diarrhoea.

MH- When the animal is not better what he do ?

SH told to IP- This medicine is enough and then go the deworming primarily with Albendazole (Albomar)

MH-How many animals he was treat ?

SH told to IP- Per day 6 patients he treat

MH- What type of animals are those?

SH told to IP-Mainly the anaemia patients

MH- What type of animals are they?

SH told to IP- 2 cows,3 cows and rest are the poultry

MH- How does he know how to free from anaemia ?

SH told to IP-They examine the mucous membrane, body condition of the animal , the animal is very weak by this way he diagnoses the case .

MH-What treatment he give ?

SH told to IP- He told that after the observation of anaemia and the bottle jaw condition. In Sunderban Amphistome is common , then he treat with the medicine like oxyclozanide of any company and they use the iron tonic fecolt by the cappharma. These medicines are not used in pregnant animals which may cause abortions.

MH- how often does he use antibiotics ?

SH told to IP- In from six patients he use in two patients antibiotics

MH- Which antibiotics he used ?

SH told to IP- Enrofloxacin and Binocin

MH- Does he have the binocin ?

SH told to IP- Yes

MH-How the cost for treatment of chromostat?

SH told to IP-9 to 10 rupees its a human drug and the dose is for the 150 kg he gives the 10ml

MH-How does he know how much to given ?

SH told to IP- He known the dose from the VS and LDA and his own experience he came to know that for the 150kg body weight 10ml chromostat is required and price is 9 to 10 rupees

MH- what is the cost of the medicine?

SH told to IP- He purchase the medicine for 9 rupees and he gives to the farmer in 10 rupees/ ampoule and vial is

contain 2ml. Per ampoule he got profit of rs 1.

MH-what other human medicine does he use to treat the animals ?

SH told to IP- in case of bronchitis and fever he use Benadryl , cipron in goats for coughing

MH-Which animal cipron is use ?

SH told to IP- For goat

MH- why does he use this medicine for goat ?

SH told to IP- Because in night he did not go to the farmer room that why he gives that tablet.

MH- What does mean by farmer room ?

SH told to IP- Farmer room means villagers kept the animal

MH- what medicine would he give them ?

SH told to IP- he give the ampicillin the veterinary medicine and injection zeet (antihistaminic),injection paracetamol and otherwise he use the meloxicam or paracetamol combination

MH- Does he use other human antibiotic ?

SH told to IP- Generally he does not use human medicine. Sometimes he uses human ampicillin

MH- Why does he use the human antibiotcs ampicillin ?

SH told to IP- Because due to unavailability in his bag of veterinary ampicillin so he purchase the human

ampicillin

MH- Does he give any human ampicillin ?

SH told to IP- No he has no human ampicillin .when its required he is purchase the human medicine .

MH- where he get from the human medicine and what type of places he buy from this medicine ?

SH told to IP-From [nearby town name redacted] [drug shop name with veterinary section redacted]

MH-Is this same place or different place ?

SH told to IP- Ok

MH- Is any other else ?

SH told to IP- They only purchase from the [drug shop name with veterinary section redacted]

medical store .

MH- Onlyfrom this place ?

SH told to IP- Yes because of the all medicine he got there

MH- From any other places he will buy medicine ?

SH told to IP -The medical representative give some sample he also use the medical representative sample

MH-Does he get sample of antibiotic from the medical representative ?

SH told to IP-Yes

MH- How and where does he meet with the medical representative ?

SH told to IP- They meet the medical representative in the block office in the BLDO office and the [pharmaceutical company name redacted] company came here

MH-whats medicine does he gets any medicine from [pharmaceutical company name redacted] ?

SH told to IP- From the [pharmaceutical company name redacted] they get antibiotics, liver extract, calcium mixture

MH- why does he not go to the shop in gosaba ?

SH told to IP- Because in [drug shop name with veterinary section redacted] he get all medicine so go to the [drug shop name with veterinary section redacted] and because of [drug shop name with veterinary section redacted] they having the good behaviour and good percentage,relaxation means the price relaxation.

MH- Is all the farmers work on that credit ?

SH told to IP -In 5 animal, 3 are giving the full payments other are giving the half payment.

MH- How much discount did he get?

SH_ I have to show you a challan/bill. (SH showed the bill)

IP- Can I take a photo of it

SH- sure

MH- Do MR offer any type of gift ?

SH told to IP- No

MH- when he has the sample from medical representative does he have to paid for this ?

SH told to IP-No it's free of cost.

MH- When he use cipron how he know that how much to give ?

SH told to IP-he know by his own experience first and for the 4 kg body weight of goat he give the 500mg

MH- What is the normal body weight of the animal?

SH told to IP-Its depend upon the age and the feeding pattern.

MH-does he have any guideline how to use the antibiotics in printed sheet ?

SH told to IP- No

MH- Does he is having in inspection that how is working ?

SH told to IP- he haveto go the BLDO office and submit the AI report., but there is no visit

MH- what type of report does he give ?

SH told to IP-Report of the AI how AI is perform and monthly how many AI is done by the BLDO office ,he submitted the report

MH- Any other informations ?

SH told to IP- No only the AI report.

MH- Is there any report about the treatment ?

SH told to IP- No

MH - How antibiotics is used generally to the animals in your area ?

SH told to IP- Those already told like oxytetracycline,enrofloxacin and binocin

MH-Does he is heard about antibiotic resistance ?

SH told to IP- He known about the antibiotic resistance from the VS and the BLDO office ,theVO and BLDO told him that the if antibiotic give in lower dose for prolonged period then the antibiotic does not work properly then he use the another antibiotics.

MH-Does he give any example of experience of antibiotic resistance ?

SH told to IP-he is saw that in bronchitis and mastitis case he use 500mg/kg body weight ampicillin and cloxacin and when its not work properly and then he use per kg body 1500mg/kg body weight he use and he seen that there is a marvellous result .

MH- Thank you. Did you known any commercial farm in this area ?

SH told to IP-Yes there is one commercial farm

MH-What type of commercial farm ?

SH told to IP-poultry commercial farm and there also hatching the eggs and thats only 6 months old farm

MH- What type contact does he have with the commercial farm ?

SH told to IP-They came here to talk and consult

MH- what type of animals they keep in your area?

SH told to IP- They keep the goat and garole sheep is very much common in sundarban previously.but they are not rearing the sheep because of in the goat they get more profit than garole sheep

MH- How many goats do people have ?

SH told to IP- Minimum 5 goats and cows also with combination of goats in a house

MH-how many cows ?

SH told to IP-1 to 2 per house .In total 5-6 households out of 10 household keep cowas

MH- How many poultry ?

SH told to IP- The chickens is not rearing in all the houses and in surrounding only 4 houses and near about 50 or 100 chicken is there

MH- So in 4 to 5 houses only ?

SH told to IP- Yes

MH-Are there any quack in that area ?

SH told to IP- 3 or 4 quacks are working in the area

MH- Does he have the number one of a quack for go and talk to ?

SH told to IP- No

MH- Would its possible to take one of the houses with 50-100 chicken for interview to the household ?

SH told to IP- Yes

Ok thank you...
