# Supplementary material for: “If It Works in People, Why Not Animals?”: A Qualitative Investigation of Antibiotic Use in Smallholder Livestock Settings in Rural West Bengal, India
Source: Antibiotics (Basel). 2021 Nov 23;10(12):1433. doi: 10.3390/antibiotics10121433 (PMC8698124; doi:10.3390/antibiotics10121433)
Supplement: Supplementary file 1 [file antibiotics-10-01433-s001.zip › Supplementary S1_ Interview Transcripts/Site 2/Public-private VPP 4 (site 2).pdf]

Code for Study - ‘If it works in people, why not animals?’: A qualitative investigation of antibiotic use in smallholder livestock settings in rural West Bengal, India: Public-private VPP 4, Site 2

Interview Date: 1/15/2020

Location: Site 2
Interviewee: Public-private VPP 4 Site 2 **(**Pranimitra)- Antibiotic Provider

Interviewer: Mat Hennessey (MH), supported Indrajit Patra (IJ) and Soumen Samanta (SS)

Transcript prepared by: Soumen Samanta (SS)

MH-Mat Hennessey

SH-Stake holder (pranibandhu)

IP-Indrajit Patra

SS- Soumen Samanta

YYYY – Para-vet name

MH: Say, thank you for giving interview.

SS to SH: Sir is thanking you for the interview.

MH: What is she doing now?

SS: Making broom sticks, to make a broom, one type of sweeping material.

MH: She is working as a *pranimitra*. Is that correct?

SH: Yes, have just learnt.

SS: For how long are you working as a *pranimitra*?

SH: One year passed; but our training was completed much earlier, just do vaccination in YYYY’s (a paravet) camp. Till now I have vaccinated 300 (animals).

MH: From where did you get training?

SH: [village name redacted].

SS: Is it any organization?

SH: The animal doctor there, they trained us.

SS: Block?

A: I was sent there from my block, through the “sangha” (self help group), through group (Self help group) 1 person was selected. Previously 2 were selected, then we didn’t come to know, they are working for last 3 years. This time they ordered “sangha” to send one person and “sangha” sent me. For 15days I went there.

SS: Who did train you there? [name redacted of a veterinarian] (salutation)?

SH: Yes.

SS: From where those doctor came?

SH: Just like our BLDO,

SS: Is it a separate block?

SH: Yes.

MH: How do you get selected?

SH: There are 180 SHG (self help group) under my [name redacted]. In one group 10-12 people are there. From that I was selected and sent

SS: From 180 group only you were selected?

SH: Yes.

MH: How many people did take training with you?

SH: 42 from different blocks.

SS: How long was the training?

A: 15days.

MH: What did you learn there?

A: How to vaccinate, what are the diseases and they taught us the primary treatments. We were told ‘you can treat better than a farmer but not like her who are treating for more than 3-4 years’. Like if a farmer instantly not gets to reach the doctor, then how we can manage that condition. Mainly it was on vaccination.

MH: What type of vaccination do you give?

A: In goat: PPR, goat pox. In cow: FMD. But I do not do FMD vaccination yet.

SS: They taught you?

A: Yes, from [village name redacted], also different kinds like castrating goats, or how to vaccinate cows, how to castrate male cows. Here not much cows are present, in [village name redacted] mission more cows are reared.

SS: Did they come or you went there?

A: Their staff came here to demonstrate us.

SS: Is it within that 15days?

A: Hmm (yes). Besides, we went to villages where cows were present and doctor gave us hand on training, how to give (vaccine), where to give.

SS: In goat you said goatpox, ppr; in poultry?

A: In poultry Ranikhet, other vaccines are also there like chicken pox, but we do pox vaccine very less. Mostly ranikhet disease is more

MH: Where do you get the vaccine from?

A: From BLDO office, and when not available I took it from shop.

SS: Which shop?

A: In [town name redacted] medicine shop, it can be bought.

SS: [drug shop names redacted]?

A: Those are human shop. Also there are some shops like [shop name redacted]. They keep mash, chicks, many more.

SS: Is that in [town name redacted]?

A: Yes.

SS: Do they keep all poultry items?

A: Poultry, cows; we also take from [NGO name redacted] model.

MH: How do you pay for the vaccination?

A: If we take it from tagore society we have to pay cash. BLDO office don't take money. But from other places (like [poultry shop name redacted]) take money. When we do from office, it's free. From BLDO office @ 1 rupee/2 rupees, we have not got any money yet. I do with the senior pranimitras. They give money to them (seniors). We learn with them.

IP: Per vaccination ½ rupees?

A: No, what we do with YYYY, per vaccine he gives 1 rupee.

SS: If you take from BLDO office it's free, but if take from [name of NGO redacted] model? How much do you have to pay?

A: I think 25-30rupees for F1,

SS: For 100 birds?

A: Yes for 100birds.

SS: And if you buy from those shops?

A: Almost same.

MH: How does she get paid for her work vaccination?

SS: She said per goat 1 rupees. Isn't it?

A: Yes.

SS: Do they pay that money instantly?

A: Yes.

MH: How did you get paid for the poultry?

A: When we go to the village for vaccination we take @1 rupees.

SS: And for goat also 1 rupees?

A: No, till now we didn't vaccinate by buying in goats. If it is given from BLDO office we it for free. In camp also we give it for free. Camp happens from BLDO office, from [project name redacted] project.

SS: That you have to do for free?

A: Yes.

MH: So how do you make money by vaccinating goats?

A: Money means I have not get money from vaccination. Last one camp I attended, in YYYY's area, per vaccine 1 rupees we got. We 3 women together did 600 vaccines, he gave 200 rupees each.

MH: You got paid by the camp?

A: The doctor came in the camp and gave us the money.

This time I did goat pox 353 numbers.

SS: Money?

A: We people did it together. The money hasn’t been given yet.

SS: At how much rate?

A: 1 rupee per (animal)

MH: How much time do you spend during vaccination?

A: We did 4-5camps in 2days.

SS: How much animals did you vaccinate in that camps?

A: Depends on area, if goat is ill is not vaccinated. Around 600 or more.

MH: What type of primary treatment do you do that you have learned?

A:Like any wound/ulcer on cow; due to lack of money I still didn’t buy any medicine and not did any treatments yet. They do, the other *pranimitras*.

SS: Where they are from?

A: [village names of 2 villages redacted].

SS: You didn’t do a single treatment yet?

A: No. Here are many *paravets* or quacks like YYYY babu, they do it.

SS: If your surrounding farmers call you?

A: If people call, what I need to say I say.

SS: How?

A: I say them to go to those doctors. I do just vaccination.

MH: What treatments did you learn in training?

A: Like if there is wound in goat or cow to give ivermectin and if it not reduced then go for antibiotics.

SS: Which antibiotics?

A: I did camp long ago, now I can’t remember. It passed 1 year when I took training. (she smiles)

SS: What do you know about ‘antibiotics’? penicillins?

A: Penicillin is antibiotic.

SS: What did you learn? Which you were advised to use?

IP: Oxytetracycline? Like that?

A: Like that.

They also gave a book.

SS: Also vitamin injections you have to do?

A: Yes.

MH: Do you know what antibiotics are?

A: When symptoms like fever occur, 2-3days passed but not getting down; if you give antibiotics it is cured quickly.

SS: How many days it has to be given?

A: 3days.

SS: If you give one day it get cured?

A: No. It has a dose like 3days.

MH: Do you know any name of antibiotics?

A: Oxytetracycline, meloxicam.

MH: Did you use antibiotics to treat any animal ever?

A: No, no.

MH: Did she give treatments to any animals?

MH: Did you get any chance to treat any animals?

A: No.

SS: Small wound/ulcer?

A: No.

MH: She mentioned quacks, what does she mean by quacks?

SS: She meant YYYY, paravet.

SS: How many paravets do you know except YYYY?

A: [names of two para-vets redacted].

MH: How many paravets do you know together?

A: [names of two para-vets redacted], YYYY, [name of another para-vet redacted], may be more but i don't know their names.

MH: Do you work with any of the pranimitra in this area?

SS: In this area who are the pranimitras like you?

A: No, 1 person per G.P.

SS: The other 2 person that you have told.

A: They went from G.P. they are 2, total 3 person in G.P. In every G.P 3 people.

SS: You know the other pranimitras?

A: Yes, I have their phone number.

MH: Do you know other pranimitras doing treatments to animals?

A: [name of pranimitra redacted] of south [name of GP redacted- site 2], she does treatments little bit. The other one I don't know.

They worked for free in their first two year service then they get a salary of 1500/month from block. I have been there for 1 year so I don't get.

MH: The pranimitra who is doing treatment, where does she work?

A: [names of two villages redacted].

MH: Do you having phone number of her who does treatments?

A: Yes. I can give you.

MH: Do you know what type of treatments she treats?

A: Cow, goats and poultry.

MH: What type of problems?

A: You can ask her by phone.

SS: Do you know?

A: I don’t go there. She tells when I ask. ‘‘I have kept some medicine, few people calls me’’.

SS: You never ask what type of treatments she does?

A: My house is not there. When we meet in any meetings,

SS: then you ask?

A: No, then mainly for what purpose we go there, after completion we return back soon.

SS: Do you know which type medicine does she buy?

A: No.

SS: She takes it from block?

A: No, she buys from [town name redacted] shop.

MH: Did you ever buy medicine from shop?

A: No.

MH: Did you take any extra training after that initial 15days training.

A: Here, after that training we went to the block hospital for 1months and seen the treatments of what animals came there for treatments. If need to inject medicine we injected it. Or any poultry comes we do.

SS: Who decides which injection to be given?

A: The V.O sir, his assistant, they tell. And how to check the temperature of an animal.

MH: How long?

SS: One month except the holidays.

MH: What are the other sources of income other than as a pranimitra?

A: Little cultivation, animal rearing, working as a day labour also. I also do self help groups and help the group members to get their loans, group formation etc.[life history redacted]. We had 5 bighas of land but had to sell 2.5bighas. I do cultivation myself.

MH: Can you say more about self help group? What they do?

A: Women in the group deposits money monthly in the bank, how they would get loan, I fill the forms and give it to the ‘sangha’; ‘sangha’ sigh it and give that to bank, like this.

SS: That means if any member wants to take loan they have to ask ‘sangha’?

A: Have to tell to ‘sangha’, to us.

SS: What about that depositing money?

A: Everybody have to deposit money monthly.

SS: How much?

A: Stats from 30, now they deposits 40-50 rupees every month.

SS: One group 40 rupees or one person?

A: Each, if 10 people are in the group, 400 rupees.

SS: What are the other function besides depositing money and giving loan?

I collect it and make them signed and I gave it to the ‘sangha’. The women deposit 30-40rupees each month. If they want to take loan they tell me and I go to the ‘sangha’ and manage that loan.

Many can give 100 rupees each. The lowest is 30-40 rupees each.

SS: And what more?

A: Sometimes group also distribute chicks and we made the list that which member will get the chicks. It is not possible to distribute chicks all the 10members of a single group, only 2-3 members were selected. We live in the village and I know who needs it. On that basis chicks/ ducklings are distributed, told them how to rear.

SS: Who told them that?

A: As pranimitra I also told them, senior didi used to tell those previously like how to deworm, or you should not release the birds all time.

SS: Are the didi of ‘sangha’ is pranimitra?

A: No, those didis(seniors) of ‘sngha’ are involved in to go here and there where self help group’s activities could be improved.

MH: How many members are there in your group?

A: 13.

SS: All women?

A: These all are women groups. Before 2013 men’s also had group. (??)

MH: How long are these groups been around for?

A: Before the year 2000 also, there were few groups but after that the group numbers has increased.

MH: Are this 180groups in block?

A: No, only in our rangabelia G.P.

Previously people took loan at 4-5% interest from ‘bandhan’, ‘jagaran’ banks. The profit in this group is, why peoples interest grows is – suppose you take loan at 1 rupees (%), when you return the 1%interest money to the bank through ‘sangha’, they will return you 30%of your interest money back to you. You will not get this facility anywhere.

MH: Who organizes the groups?

A: When groups are newly formed for 6months, government will give you 1.5lac rupees as loan. Then you have to fill up a form and ‘sangha’ will deposit that form in BDO office and government will give the group 15,000rupees that government will not take back. As in 6months a group’s principal amount is less so that they can utilize that money. Again after 6months, you have to fill up ‘cc’ form for 1.5lakhs loan, then each member take loan from that 1.5lakhs. Some may buy goat, or use that in cultivation and returns the principal with interest dividing in each months within 1 year. When they return those 1.5lakh at the end of the year, then the government gives you 2.5lakh loan through the bank.

SS: Which bank?

A: Our [GP name redacted- site 2][bank name redacted]bank- central bank. Then if you return 2.5lakhs then again 3.5lakh you can get as a loan. Maximum 3.5lakhs you can get.

MH: Is ‘sangha’ is a person or organization?

A: It is a committee of 5-6members. We all are members of ‘sangha’. 180 groups are registered under them. In new ‘anandadhara’ order every group has to give 50 rupees free fund each month. Later group can take loan from that. It (sangha) itself also give loan.

MH: Did they need permission of ‘sangha’ to from a self help group?

SS: Do ‘sangha’ take commission to form a new group? (wrong question asked by SS)

A: No, no, no. Our ‘sangha’ run 2 hospitals. They supply diet of patients in the hospitals. Our ‘sangha’ selected one cook for that. In [village name redacted] also one hospital is run. The profit from that, the government also may give some money to CSP, who do these activities. No commission from these groups member. Government gave 21lakhs that they can use in different activities, also they can run the money as loan. From that income they can give the women

members. Like if they give the group 1 lakh loan, then group should return it within 1 year. ‘Sangha’ need not to return the principal to the govt., they use the interest money in different activities.

SS: So after few years, the ‘sangha’ will have a huge fund.

A: Yes. This time they get the responsibility of selling paddy.

MH: What type of animals do you keep here?

A: I had a cowshed, now it’s destroyed. I had 2 cows, 5goats but had to sell the goats due to damage of shelter in last storm. I am keeping my cows in verandah /porch. I had 50 poultry but after the storm I have 8 now, I have sold the rest due to lack of shelter. After making new shelter I will rear again. I also make much money from poultry. Almost 50 birds I rear every year.

MH: How much she have the goats now?

SS: How much money did you get buy selling the goats?

A: 1500-2000 per large goats and for small goats 700-800 rupees.

MH: Do you have any goat left?

A: No. If I keep goat in verandah man can’t live there. I am hardly managing cows now. In this winter, in cold weather it’s problematic to keep them outside. I will break this house later, then I will make cow shelter in that side; 3 people lives here 1 quarter will be enough.

SS: What are the problems you face with the cows?

A: Its health was good but after the calving its health condition is detoriating and also not giving milk.

SS: Totally no milk?

A: Some evil minded person made conspiracy during calving that’s why it is not allowing to touch its teats. Giving milk to it’s calf but not to us; previously if you lie beside it, it didn’t disturb you.

SS: What are the other problems you face like any fever or like that?

A: No, by Gods shake no big problems occurred to my cows till now.

SS: No medication needed?

A: I sometimes feed deworming medication, then feed them vitamins.

SS: Did you vaccinate the cows?

A: One time.

SS: In camps?

A: Yes, I took them to the camp.

SS: You don't vaccinate cow by yourself?

A: No till now I haven't vaccinated any cows in villages.

SS: Only poultry?

A: Poultry and goats. Our FMD-CP is going to happen in February-March I think.

MH: Why do you not vaccinate the cows?

A: They not give me vaccines as I am new. After returning from my training as pranimitra FMD has not been done yet I think. They gave to seniors they did.

MH: Do you vaccinate other peoples cows?

A: No.

MH: So you do vaccination of goat and poultry only?

A: Yes.

MH: What do you use these cows for?

A: Since 'AILA' year I bought cows. Then I had 3 cows. The shelter was destroyed, I sold them. There was no place to live. We had to depend on supplied water by boat. What I will eat and what I will feed them. I had to sell them at 2100 for 3 cows, at very less price. One was milking then, I had to sell with calves. After that I could not buy cow due to money problem. Then after 2 years I bought a cow, after 3 years it conceived but aborted after 3-4 months.

SS: What do you get from cows?

A: I use cow dung for fuel, fertilizer, get milk and if I am having 2 calves I sell one calf when it get little older.

MH: Would you be able to vaccinate the cows?

A: Yes I can do.

MH: Why do you not do?

A: Block didn't provide.

SS: Why?

A: As the government has not provided any cow vaccine to the block since I joined as a pranimitra, so I did not get any chance to vaccinate the cows. When they will give I will do. My senior pranimitras did before. Govt. gave for poultry, duck goat that I did.

MH: Do you having any problem with the poultry?

A: In summer dullness, dropping of wings, whitish chalky diarrhoea, worms coming through feces. In pox also my few chicks died.

MH: What do you do then?

A: Go to the [NGO name redacted] model.

SS: Who is there in model?

A: Doctor [NGO vet name redacted], he not always present. [name redacted- member of NGO] is also there.

Also go in Block BLDO office and tell the problems and they give the medicine for free.

SS: In [NGO name redacted] you have to pay?

A: Yes.

MH: Which one do you prefer [NGO name redacted] or block?

A: [NGO name redacted] model.

SS: But block is cheap.

A: That is far away.

SS: How much do you pay to [Name of NGO redacted]?

A: 17-18rupees/10birds including medicine.

MH: What do you received for that 18 rupees?

A: Powder, liquid. Don’t know any name.

SS: For how many days do you continue it?

A: 3-5days as they says once or twice a day.

MH: Did you get treatments from anywhere else?

A: No, sometimes I feed herbal treatment, neem juice etc.

MH: Did you ever call the paravet?

A: Yes, when there was late in calving (dystocia) I called YYYY babu.

SS: How much money you had to pay?

A: 150rupees.

MH: Why did you call him not to the block or [NGO name redacted]?

A: In there you have to take the animals to there but he can come to home.

SS: Why do you rear these poultry? Eggs?

A: Yes.

SS: How many?

A: If you allow to graze freely, it will give eggs.

SS: Now from 8 hens how many eggs do you get?

A: No one is laying now. In the middle, one layed 7-8 eggs. Then again stopped, as it is caged. If you allow to graze freely, it give more eggs.

SS: That is one hen. What about others?

A: They are small.

In this winter picnic season I sell the adult birds to the shop at 250rupees per kg. The shopkeeper sells them at 300 or more per kg to the lodge, hotels. Last year I earned 6000 rupees.

MH: Does you eat these poultry for yourselves?

A: Yes.

SS: How many times?

A: Once or twice a month.

SS: You not eat outside poultry?

A: Yes but less.

I bought 39chicks to sell in picnic season but the prey animals like ‘kotas’ (fox types) ate many of them.

MH: How do you feed the cows and poultry?

A: Paddy straw, rice bran, grass, left over boiled rice also.

MH: Where do you keep the poultry now?

A: There. (indicates to a shelter in front of her house cover by polythene as her previous shelter has been destroyed by storm)

MH: How many people do live with you?

A: 3 (me and my 2 daughter).

MH: Where do you go when your daughter get ill?

A: To [name of NGO redacted] health camp mostly.

MH: Did you ever use human medicine for animals?

A: No.

MH: Did you have any old medicine packet of animals?

A: No, no such big problem occurred in my cow since last 3years. Only deworming medication has been done and vitamin powder.

MH: Do you have old medicine packaging of poultry?

A: No. If you want to see the human medicine I can show you.

MH: How old is this calf?

A: 1month 2-3 days.

MH: Does it take milk from the mother cow?

A: Yes.

SS: the calf is taking milk but the cow is not allowing people to drain milk.
