# Supplementary material for: “If It Works in People, Why Not Animals?”: A Qualitative Investigation of Antibiotic Use in Smallholder Livestock Settings in Rural West Bengal, India
Source: Antibiotics (Basel). 2021 Nov 23;10(12):1433. doi: 10.3390/antibiotics10121433 (PMC8698124; doi:10.3390/antibiotics10121433)
Supplement: Supplementary file 1 [file antibiotics-10-01433-s001.zip › Supplementary S1_ Interview Transcripts/Site 2/veterinarian 2 (private) (site 2).pdf]

Code for Study - 'If it works in people, why not animals?': A qualitative investigation of antibiotic use in smallholder livestock settings in rural West Bengal, India: Veterinarian 2, Site 2

Interview Date: 1/13/2020

Location: Site 2

Interviewee: Private NGO Veterinarian - Key Informant/Antibiotic Provider

Interviewer: Mat Hennessey (MH), supported by Soumen Samanta (SS) and Meenakshi Gautham (MG)

Transcript prepared by: Soumen Samanta (SS)

MH- Mat Hennessey

MG-Meenakshi Gautam

SS- Soumen Samanta

All answer(A) by A.

XXXX- Name of NGO

(Q: What types of antibiotic are used?)

A: Amoxycillin/cloxacillin, Ampicillin, Enrofloxacin, Amikacin, Cephalexin,

Oxytetracycline and Amoxycillin/cloxacillin powder are mostly used in poultry. And enrofloxacin liquid is used in poultry. Actually we are trying to restrict the use of antibiotics in *Sunderban* area. We are trying to aware the paravets and trying to improve the health status of the animals. Proper dose of antibiotics if they don't know, naturally antibiotic resistance and some other problems may arise. That will directly affect the human health. It's a problem.

MG: Can I just introduce our rest of the team?

You know West Bengal University of Animal & Fishery sciences is a collaborator and London School of Hygiene is the main implementer, the main research organization. Mat is from Royal Veterinary College.We have also more people over there but they are not here today so maybe next time you will meet them. Mat is a Vet. (Mat : I am a research assistant on this project .)

A to Mat : your specialization is in medicine?

Mat: Yes I did cats and dogs, small animal medicine and ultrasound is my speciality.

The other interviewer Soumen is the student of West Bengal Veterinary College. Pabak is from Liver Foundation. He is working on the human health part of this project and looking after the antibiotic supply chain which are used in govt., private sector and also from informal providers(quacks).

A: that means you are collecting data from quacks also?

MG and Pabak: Yes, yes.

MG: Liver foundation has a long history of working with informal providers before. It's led by Dr. Abhijit Chowdhury. The other member Sourav, Sourasree and Arindam is from there. That's our team.

MH: Thank you for your time. How long you are working in this area? What is the main type of work you do?

A: *Redacted life history*.

MH: Is this project is still continuing now?

A: The project is running in other side of *Sunderban* now but that revolving money is still utilised for that particular purpose. New project area was selected from last year. From 2018 another 2 GP was selected for that purpose. And previously it was [names of three nearby GPs to Site 2 redacted] GP. Now they are having their own fund also and revolving. Money is used for their own purpose.

MH: You said you are coming twice/thrice here in a month?

A: Yes, yes. On Saturday, Sunday. I utilise my holidays to spend with these people.

MH: How many people work in this society like you? Any other veterinary officer?

A: Block V.O. sometimes gives services to the peoples in holidays. But mostly he prefers to give service to the tribal people. In our [name of site 2 Block redacted] Block total 14 G.P is there. Two side Bali 1 and Bali 2 some tribal peoples are there. In [name of site 2 GP redacted] there is one tribal affected area, our university is also having one project on Goat improvement. One another project on tribal development agricultural support is going on, that is under [name of project redacted. This time 110 tribal women were taken to it for training purpose.

MG: What type of training was it?

A: Agricultural based enterprise. Not only Animal husbandry, pisciculture and agriculture (mostly paddy, horticulture and vegetable production). 5days training programme.

MG: The V.O. you have talked, is he in XXXX?

A: No, he is in formal structure (Government).

MH: In the BLDO?

A: No, there is one BLDO, one V.O and one another V.O. in ABHC (Additional Block Animal Health Centre)

There is also *Pranibandhu* and *Pranimitra* in each G.P. *Pranimitra* you can say formal or informal, they are not salaried person. *Pranibandhu* is for A.I. (artificial insemination) purpose and *Pranimitra*is for doing vaccination of small animals. One pranimitra (Subhra) will come probably.

MG: Tell me about the animal health system.

A: Now, the large animal population is decreasing gradually. There are some reasons. In the year 2005 when I came here for the first time in each family there were 5-6 cattle but now they prefer to rear only milching cows.

MG: Why?

A: Now people are calculating what the economics is. Actually bullock is used for ploughing purpose. Now due to mechanization power tiller, tractor came for agricultural purpose and there is no need to keep the bullock. And due to shrinkage of agricultural land and hallow land it is very much difficult to provide feed & fodder and the cost of purchased is increasing gradually. Thus people are thinking that if they keep one animal and it gives them 10-12 litres of milk, it is profitable. And if the marketing system is not proper, marketing system is also problematic here. But we are making the people aware to produce some value added products like ghee, cheese. And they are supplying milk to the local sweet shops.

MH: How do they do that? How do they supply milk to the shops?

A: They supply milk from one island to another island by boats; and by local transport like van, cycle, motorbikes. And previously Jersey cross breed (of cattle) was there but now due to change of breeding policy mostly Gir, Gir crossbreed are there as milching cows.

MG: So in artificial insemination(AI) what kind of breed they use in AI?

A: Mostly Jerseycross breed and Gir. (Dr. Indranil: Gir is a high milk yielding Indian breed). In India 4 milch breeds are there, out of that Gir is one. Some constrain is also here. The percentage of conception through AI is less in our country that is 35%. Sometimes people are less interested in AI, if he fails conception 2 times then he will be less interested for AI. Actually per AI it cost about 150 rupees. So two times fails means 150+150=300 rupees loss. Most of the peoples are poor and marginal. So how they will afford the money?

MH: What are the types of livestock people rear in this area?

A: Actually large animal rearing is decreasing. Mostly people are giving concentration on keeping goats and poultry. And one another important livestock of our *Sunderban*area is *Garole* breed

sheep, it is decreasing. It is famous for it's prolificacy. It is having "*fec B*" gene. If you compare goat and sheep, people prefer goat for its meat quality, prolificacy and skin quality.

MG: What is prolificacy?

A: That is litter size means from one mother more number of offspring is produced. Garole sheep is the only breed which can sustain in saline atmosphere. In India total 43 number of sheep breeds is there. Unfortunately the population is decreasing.

MG: Why?

A: Because people's mentality, they think that goat farming is more profitable than sheep. *Garole* sheep mostly produce coarse type of wool. And the wool marketing is not proper channelized, so sheep population is decreasing. *Garole* meat is also not preferred. In 2010, we organized an international level conference. Main objective was to aware people about *Garole* sheep keeping and to popularize *Garole* sheep among Indian scientists but we were not successful. One application we send to NBAGR(National bureau of animal genetic resources, Karnal, Haryana, India) that please change the name of *Garole* sheep to *Sunderbani*sheep. Then only people think that it is our property and we will conserve it.

Dr. Indranil: Actually *Garole* means foolish person.

MH: What about the poultry?

A: Ok Madam, one small animal we wanted to popularize that is pig. Some tribal people rear *desi* pig breed. Two years before I run a project under tribal Subplan, we supplied 40 number of *Ghoongroo* pigs. It is a recognized pig breed of our state. We have supplied but handling of that breed and proper health care, they were not interested of that breed. Now also 10-20 numbers of pigs is there but tribal people mostly prefer small *desi* pig. But there production, prolificacy is very less. Litter size is 5-6, where in case of *Ghoongroo* it is 12-14.

So large cattle breed, sheep and pig is decreasing. But goat and poultry population is increasing. If you go through the last animal census report, the rural poultry is increased near about 46% from 2012-2019. And commercial poultry has increased only 4%.

Dr. Indranil: Rural poultry means backyard poultry.

MH: Is there any commercial poultry in this area?

A: Commercial poultry means small broiler and Kuroiler/ coloured broiler farms are seen. Marketing age of broiler is 35days whereas marketing age of Kuroiler is 2months or 2 months 10days. Cost per kg is also more in Kuroiler. But other than broiler and Kuroiler,the people are rearing some family poultry. From our university regularly XXXX purchase 'vanaraja' birds. 50% is taken from our university. There is also 'gramapriya', 'nirvik', 'kalinga brown'…. these are

popular family poultry. Not only our university supply these birds but also central breeding farm, *Bhubaneswar* and C.P.R, *Hyderabad*. These are developed for free range / backyard rearing.

(Some conversation within themselves..)

MG: You said poultry population has increased 46%, is it all India, or in West Bengal?

A: Yes, all India.

MG: Where have you get these data from?

A: 20th livestock census(2012-2019).

MG: What about commercial poultry?

A: 4.6%, but increased. And the average value would be probably 14%, total population increased.

MG: What about total population?

A: Total poultry population is 851 million at present and probably 551 or 557 be commercial poultry population and 300something be rural poultry.

MG: In some year backyard poultry will take over commercial poultry.

A: *Sunderban* is world heritage site, if you stay here you will gather a lots of knowledge. Total 102 number of island are there among them 54 is human habitat.

(Some conversation within themselves)

MH: In [name of site 2 GP redacted], what type of peoples work with animals? You said pranobondhu and pranimitra, or any other else?

A: Mainly marginal farmers, and commercial means say 4-5 dairy animals, 100 to 200-500 numbers of poultry but this % is very less. Most of the people prefer to rear one to two numbers of non-descript or crossbred cattle.

Q: Are there any commercial farm that we can visit?

A: In Indian perspective it is not a commercial farm. But in rural area if one person is keeping 200 birds it means commercial. Actually if we go through the earning of common people there is mostly 3000-4000 rupees per month. These broiler or layer farms also earn 3000 or 5000 rupees. So if you compare poultry and some other then it is commercial to them. In [name of site 2 GP redacted] there are 50-52 number of small farms with 100-200 number of poultry birds. More than that only two farmers I know. One is [name redacted] in interior place. Mostly broiler farms, Kuroiler is very less, less means they are keeping hardly 40, 3o. People mostly keep 20-25

numbers, they rear then they sell and again next time keep and sell. Some people rear Kuroiler birds mainly for laying purpose, they rear upto 3 months then sell the male birds and keep the female birds and at the age of 5 monthsor five and half months it starts laying. Upto 1 year laying they keep then as a spent hen they sell it.

MG: What is that bird? (indicating a bird which came to the hospital)

A: It's Assel, a fighting bird. They have long shank. India's popular fighter bird though cock fighting is not ethical. As per their fighting capacity their cost varies. Long shank and lighter weight is the first criteria for fighting capability. During fighting they will fly up to 3-4 ft. And one blade is set just at the level of hock joint.

MG: So what types of peoples are involved with rearing?

A: It is not their principal source of income. Those keeping more than 100-200 birds, their 50% income is from those birds. And there is having some alternative source of income. Some people involved with agriculture, vegetation, paddy cultivation and some people are having small shops like tea, grocery. Young member of the family is earning from outside in India , they are working in some other state like Chennai, Gujrat. Main income is from that young member. And mostly women farmer and old peoples look after the rearing. From our project we are also giving support to the people by not only kinds by cash also. *TATA Chemicals* is also running a project 'TCSRD',

MG: Is it with animals?

A: About giving support to mangrove conservation and agricultural support also to poultry and goatary.

MG: what type of people look after these farms?

A: You are telling about age group or their principal income source.

MH: Where do people go when their animal get sick for treatments?

MG: Who takes care of the animals?

A: First they will go to the local paravets then either *Pranibandhu/pranimitra* then our local hospital (XXXX hospital) then block.

MH: In [name of site 2 GP redacted] how many paravets are there?

A: Near about 10 persons are working. More number of trained people is there but they are not practising. [name of para-vet redacted] (paravet) can tell more about this. 3 pranibondhu, per G.P. one *Pranibandhu* but from outside two *Pranibandhus* came and practise here.

MH: How *Pranimitra*..?

A: 3 *Pranimitras* are there.

MH: How the paravets differ from the *Pranibandhu*?

A: All are trained but the paravets are trained from this XXXX. They completed the paravets 35days custom service training programme. XXXX organizes one custom service per year. Nearly 30-32 people not only from [name of site 2 GP redacted] but from other islands also like [names of islands redacted*]* apply for this. Cost of training, previously we took 3500rupees for this residential training programme including tiffin, major meal, everything. But after 2017, no programme was organized. Now 7days training programme, 5days programme, 3days programme at field level is occurring.

(KI picks a phone call)

MH: What does *Pranimitra* do? The lady who came.

A: They are mainly for vaccination purpose. Door to door vaccine they do, mostly R2B, F1 for poultry and for goat pox and PPR/goat plague (*Peste des petitis ruminants* –a viral disease of goat)

MG: Are the *Pranimitras* govt. employees?

A: Not govt employee but govt affiliated. Government is not giving any salary but per vaccine they get 1 or 2rupees (Indian Rupees). They submit master role in that way.

MG: So how do they earn?

A: They submit the master role in the government office and as per the number they vaccinated their wages come.

MG: How much do they earn per month?

A: Per month they get 3000-3500rupees.

MG: So is it per animal vaccinated?

A: Yes, per animal vaccinated. Say per bird 50paise and per goat 1rupee.

Dr. Indranil: It is very less.

A: But the *paravets*, they will demand more, that is the difference, they are in informal structure. *Pranimitra* is formal structure.

MH: How much it cost to get the paravetat home to treat the animal?

A: It depends on the case. If large animal they demand more and if small animal it is less.

MG: What is more, what is less, just for example?

A: Say it depend on the type of disease, if they do injection, saline they will demand more cost. And if they give oral medication it cost less.

MG: What kind of services do they provide? The *paravets*.

A: For treatment purpose mainly and sometimes we organize awareness programme to the paravet that not only give service to the people by treating the animal, if the people is interested to treat their animal then only you can earn. But also your other objective is to aware people to keep animals, otherwise you will not earn the money.

MG: What are the kinds of illnesses they are treating?

A: Mostly they prefer to treat symptomatic treatments. Like if they see there is high rise of temperature either fever or hyperexia, they try to reduce the temperature by injecting either any antihistaminic or any analgesic in that way. And sometimes by antibiotics.

MG: What else?

A: In some other problems like diarrhoea it may be of parasitic reason or bacterial but as per there diagnosis they treat the animals. If there diagnosis it is parasitic they suggest some anthelmintics. They first try to treat the symptom (diarrhoea) then they suggest for deworming.

DR. Indranil: How do they diagnose parasitic or other?

A: Sometimes they collect the fecal sample and in our hospital egg count is done and our workers suggest some anthelmintics. It is done in block also. Sometimes symptomatic treatments like in bottle jaw condition they prescribe oxyclosanide, rafoxanide. It may be due to some fluke.

MG: Is there any on field support they get? Like if they face any problem or is there any supervision like that.

A: Some *paravet* consult with veterinary doctor. But it is not always, sometimes they call to the doctor, if the doctor does not receive the phone they treat the animal by their own.

MG: So to which doctor they will call?

A: Mostly block V.O or sometimes they call to me.

MH: On the days when you are not present here, what they do?

A: If they face any problem they make call to [name of veterinarian redacted]. [name of veterinarian redacted] will suggest. Sometime they go to the V.O office and the V.O who is present there he gives them suggestion. Our BLDO is there. Sometimes some paravets they are keeping all the mobile numbers of VO, BLDO of local area, gosaba, basanti block. Actually at present at [name of an adjacent block redacted] block our BLDO previously was posted at XXXX. That is

why he(BLDO) is also helping people. He worked here for 3-4 years after completion of his BVSc degree, before joining as VO, govt service.

Dr. Indranil: His home is in the nearby place. That's why.

MG: What is the organizational set up in animal health system? Can you describe the health system?

A: Two set of structure. Formal structure means in block level, BLDO is associated for all the administrative purpose. Say to organize training programme, supply of poultry birds and some other administrative activities. And VO(Veterinary officer) is totally for treatment purpose. And another parallel structure is there that is MVC. It is mobile veterinary clinic (MVC)

MG: It is the part of govt. structure?

A: Yes, it's the part of govt. structure. And there is another VO for additional block animal health centre(ABHC). VO [name of block of site 2 redacted*]* block and VO ABHC. BLDO, 2 numbers of VO and another VO for MVC. The jurisdiction of these two VO is different. Under block VO 7 G.P and under this additional VO 7 G.P is there. And in each G.P 1 *Pranibandhu* is there and average 3 numbers of *Pranimitra* is there. It is not that in every G.P 3 number is present.

MG: How many *Pranimitras* are there in [name of site 2 GP redacted]?

A: 3

SS: What about paravet?

A: Paravet is informal structure. They are trained from either govt.

MG: Are all these officers in position now?

A: Yes.

MG: Where these are available?

A: But MVC is something different. There job description is to organize 20 health camps per month. And mostly the gram panchayet/pradhan fix the place for MVC camp. 20 camps per month per block. So 14 number of G.P means sometimes one to two camps per G.P. per month. And informal structure is totally different.

MG: What is the additional VO do?

A: His responsibility is for 7G.P. All the *Pranibandhu* of this 7 G.P came there for collection of liquid nitrogen, straw, some other inputs.

MG: So the *pranibondhu* are more connected with the additional VO?

A: VO.

SS: Not for this VO?

A: For this VO 7G.P and for this 7G.P. total 14number of G.P, so you divide into 7 and 7.

MH: Do these offices have livestock development assistant? And pharmacist as well?

A: It is formal system, directly controlled by BLDO.

MG: No pharmacist?

A: In [name of nearby town redacted] no pharmacist is there. One LDA is there.

MG: Where is the LDA?

A: LDA is in [name of site 2 block redacted] block.

MG: What is LDA?

A: Livestock development assistant. And in additional centre no staffs is there. One contractual person is there for sweeping purpose. He is paid say Rs. 500/month.

MH: Do the veterinary officer travel out to visit the animals which come to the office?

A: Sometimes *Pranibandhu* prefer to come to the centre and doctor will suggest , doctor will prescribe. Otherwise if the case is critical then sometime VO go. He is present but in between Friday to Monday there is some problem. Actually Saturday and Sunday is holidays, Saturday is half holiday so Friday afternoon and Monday first hour the VO may be absent then some problem will arise. Then paravet and *Pranibandhu* give maximum service.

MG: What else the mobile VO do besides organizing the health camps?

A: His job description is to organize the 20 number of health camps only throughout the block.

MG: Nothing else?

SS: Treatments?

A: Health camps means vaccination, deworming, check up and others.

MG: What is the job description of *Pranibandhu*?

A: Doing artificial insemination. And to provide season based fodder seeds. And during animal census their responsibility is maximum, they collect data from field area. And sometimes some administrative jobs, awareness camps in presence of VO or BLDO.

MG: Now tell me about the informal system?

A: Informal system means say rural youth, they get trained from NGO or govt.

MG: Who are they?

A: Mostly unemployed youth. In [name of nearby town redacted] from XXXX and from [name of institution redacted] mission and from [name of institution redacted] training centre they get trained. In XXXX the name of the training is ‘custom service training programme’. That means primary care to the animal.

MG: What are they called?

A: The name of the training is ‘custom service training programme’, it is 35 days programme. 3 phase training, first is 10days then 11days, and then 14 days.

MG: What are they trained?

A: In first phase for basic management and at the last days of the training generally some format is given to the trainees to collect information from his local area. And in 11days second phase mostly type of disease and how to handle the disease like that and they collect information from the field. And in last phase treatments and others. And after completion of the training programme regularly they are invited to the centre. Regularly means once/twice in a year to refresh their knowledge. Some from our university or local BLDO, VO.

MG: How often is this?

A: once in a year or twice in a year. And up to 2017 near about 535 people get trained. But out of that near about 100-150 they are actually actively working. Others are associated with other type of works.

MG: What are the others about that trained from [name of institution redacted] ?

A: [name of institution redacted] mostly cover dairy farming, artificial insemination and fodder cultivation. And in our [name of institution redacted] they do subject specific training. Either cattle-poultry or pig-poultry.

Q: What do you call these people? Are these paravets?

A: They are paravets.

(So we would like to meet 2-3 of these people) [name redacted*]* is one of them. Actually he is associated with one of our university project.

MG: How many of these paravets are there right now? 100-150?

A: Not only here, throughout *Sunderban* area. Hingalganj, Hasnabad, 14G.P.s of Gosaba, Basanti, Canning-1, Canning-2. I am telling the numbers in about 7 blocks.

MG: In [name of site 2 block redacted] how many would be there?

A: Actually all the *Pranibandhu* before joining as a *Pranibandhu* they get trained from NGOs. They were previously paravets now they are *Pranibandhu*.

MG: In [name of site 2 GP redacted], how many of these are providing services?

A: 3-4 paravets are active. One *Pranibandhu* is there. *Pranibandhu* belongs to formal structure, paravets are informal structure. *Pranimitra* is also within the formal structure. Detail information they will provide. They are more associated with the animals.

MG: From you we just want to have tthee overview.

MH: Are there any other persons who may provide services to animals?

A: There are some physician that means they generally prefer human practice but if people come to them and tell about their animals' sufferings then sometimes some homeopathy doctor nearby that centre generally do some homeopathic treatment and sometimes ethno-veterinary practise. That is also popular in this place.

(Some conversation within themselves)

MH: Do you have any experience with problems with antibiotic resistance in this area?

A: Now we are actually facing that problem but we are trying to restrict the use of antibiotics. Sometimes one problem may arise, say one *paravet* inject one antibiotic and if the animal does not recover within one day, then they use another antibiotic. There is a common practice. But now we are trying to aware the *paravets* in that refresher training programme that at least you have to wait for 3 days. After 3 days if it is not getting better then you can consult and can use some other antibiotics. But in *Sunderban* area the XXXX is trying to implement the knowledge regarding the scientific use of antibiotics. Actually we are not using the new generation antibiotics. The entire antibiotics I told that are old antibiotics. So the problem of antibiotic resistance in livestock is generally less in *Sunderban* but in poultry the scenario is somewhat different. In poultry they are changing the antibiotic regularly. Because mostly they rear broiler or Kuroiler for commercial purpose (Earning 3000-3500rupees/month). Say,in respiratory infection, initially they use enrofloxacin if it does not work then tylosin is used.

MG: How do they decide to change it?

A: Say from our institution one chart has been prepared from day1 to day35. For first 4 days, or 5-8 days for 100birds the medicine and the dose is mentioned, these type, including antibiotics. Say from 0-4 days tetracyclin is used and from 22-25days another antibiotic say enrofloxacin is used for all the animals. Commonly they use these two antibiotics up to 35days. And if any problem arises in that period then they use some other antibiotics. Say tylosin for respiratory tract infection. Amoxicillin/cloxacillin for any other cases like whitish diarrhoea.

MH: Are they using these antibiotics for all the flocks?

A: For all the flocks.

MH: As a preventive?

A: Yes. Say as preventive the dose is 1gm/100birds then the treatment dose is just double. It is their common practice.

(We would like to see these charts)

MG: Do have any manual for training purpose?

A: One booklet developed on poultry. But already that is written in Bengali. But only one/two copy is there.

MG: We can make a photocopy.

A: Actually there is one book on 'scientific poultry keeping' by me. Previously 5000 copies were distributed among the farmers.

MG: So what is your perception of antibiotic resistance?

A: My suggestion is 3-4 days, if you maintain the proper dose for a specific period then the bad effect or the chance of antibiotic resistance could be reduced.

MG: What is the general situation here? Are you facing any resistance? Is there any data on prevalence of resistance in animals?

A: Mostly in poultry bird the use of antibiotic is not in scientific way. Because they are always thinking about early recovery and maximization of the profit or production. That's why they are not thinking about the quality of meat or what should be the effect in future. In livestock that type of problem is not there because mostly people maintain the proper dose for say 3-4days. the antibiotic resistance means the use of antibiotic 1 or 2 days, the case is very less. Today they are using antibiotic, the next day they are changing, the case is very less.

MG: These poultry farms, would you call them backyard farms or commercial farms?

A: We are telling that it is commercial. Because though in Indian prospect it is not a commercial farm. In India if it is more than 2000-2500, it is commercial. In *Sunderban* area 1000 birds mean large farm. We can say that in between 100-200birds, it is small farm. 500birds is medium sized farm, more than 1000 bird means large farm. In [name of site 2 GP redacted*]* only one person is having 1000 numbers of broiler birds. And in dairy 2-3 crossbreed or Gir they are keeping.

Just opposite to [name of site 2 GP redacted] one island is there, [name of island redacted*]*. One person named [name redacted], he was keeping 45numbers of crossbred cattle but now he is having only 10-12 numbers of cattle. He has reduced.

Pabak: Why so?

A: Because mostly channelization of marketing is a problem. He practised two times milking. Morning milk, he prefers to sell it either in local sweet shop or market. But in evening the utilization of the milk is very much difficult. We suggest him to prepare some value added products like channa, ghee. But he is telling that sir it is not sustaining.

MG: Have you any cases of resistance in livestock?

SS: Like you are giving medicine but it's not working.

A: Very less. Actually we are trying to reduce the use of antibiotics. We are giving more stress to improve the immunity status of the animal. If the health status and nutrition status is maintained, then I think they can resist all the ailments. But definitely some cases are there. But elaborate study is required.

Q: What is the facility for diagnostic for animal here? Do you have any lab?

A: No lab is there. Generally for parasitic disease the diagnosis is done sometimes. Faecal sample is collected. Sometimes if any problem arises we inform to our Block say 3-4 years back 1 anthrax like symptoms was observed. 4-5 cattle died overnight. Then the blood was collected and all the management was provided by formal structure that I know.

MG: Do they have any lab over there in the formal structure?

A: No lab. They sent the sample to govt. DI lab(disease investigation laboratory), *Belgachia*, Kolkata.

MG: Have you observed any zoonotic diseases?

A: Maximum poultry disease is having some zoonotic importance say Ranikhet disease (*Newcastle disease*). You might have heard avian influenza last time (2007) which spread here also.

MG: Had it happen here also?

A: One or two birds have been suspected.

Dr. Indranil: It is 2007 I think.

A: Huge numbers of poultry birds were killed.

(some conversation within themselves & [name of veterinarian redacted] showed some leaflets of how to protect the animal from parasitic disease, how to cultivate green fodder and vaccination to protect the animal from different type of disease)

MG: This the part of their awareness camp.

MH: We are also looking the overlaps between human and animal antibiotic use.

A: It is seen mostly in pet animal like dogs and cats, Human medicine is mostly used. Some commercial enzyme

MG: Any antibiotics?

A: Ceftriaxone in that locality, it is available that's why I am telling. And amoxicillin+ampicillin combination and Amikacin are used in this locality.

SS: Doxycycline?

A: Yes, doxycycline.

MG: Are dog and cats are present here in [name of site 2 GP redacted*]* area?

A: Generally as a pet animal some dogs, spitz are there.

([name redacted] leaves)

[name of veterinarian redacted: if any problem arise in *Sunderban* area, you call to [name redacted] he can do all types of help.

MG: Who is he?

A: He is the king of *Sunderban*. He is the overall coordinator of [name of site 2 GP redacted] project, XXXX.

MG: For animals or human?

A: Overall. But he is very much interested in ARD, because he was in charge of animal husbandry section for 20years.

MG: Is there any overlaps of antibiotic use for other animals? What about backyard poultry?

A: In backyard poultry generally people not use antibiotics. Only per one month or two month deworming and some sorts of vitamin, liver tonic and vaccination are done. Sometimes we organize vaccination camp (against Ranikhet) for them also.

MG: What about when the animals fall sick?

A: That I told you, to their local paravets. The commercial poultry farmers mostly come to us or government hospital. And in [name of nearby town redacted] market some wholesale supplier are there. Sometimes they also support to the farmers through medicine, feed, chicks and they will collect the *ready to sale* birds from the farmer.

MH: Do the farmer directly go to the wholesaler for advice?

A: Yes.

MG: Do these wholesalers are medicine wholesaler or feed wholesaler?

A: No, no. They provide all sorts of help. We suggest the people except medicine all sorts of help you can purchase from wholesale or market. For medicine better you contact either our hospital doctor, paravet or block hospital. That will be good for you.

MG: How many of these wholesalers are present in the market?

A: In [name of nearby town redacted] market there are 5 like these.

MG: Any names?

A: That information you can get from our staff. [names of three redacted also. That [name redacted] he is paravet but he has started the business.

MG: Do any of these also have human medicines?

A: No, no. Only for animals.

Pabak: Is Subrata babu a paravet?

A: He is a paravet, actually he is associated with our one University project AICRP on Goat Improvement, our [name of site 2 GP redacted field site

MH: Where do you purchase medicines for here?

A: If the amount is more, they prefer to purchase the medicine from [name of drug shop redacted], [name of drug shop redacted], [name of drug shop redacted] at Kolkata. Otherwise at [nearby town name redacted] 2 shops are there, one is [name of drug shop redacted] and another is [name of drug shop redacted]. They are wholesaler of specific companies like [name of two pharmaceutical companies redacted].

MH: Are these twp shops keep both human and veterinary medicine?

A: Yes.

Dr. Indranil: Do you have contact of them?

A: Yes, I can give you or from our office you can collect the number.

Pabak: we went there but it was very busy, if one suggests it will be easy for us.

A: Actually madam if you want to work with people of *Sunderban,* the people would think that what the benefits/inputs or reward they will get.

MG: But this one will be more with the health system, providers, and the people like XXXX.

A: Definitely we will help you. In the AICRP projects also XXXX mainly helps at initial stages. Now also if any problems arise locally, this type of organization will help you.

MG: We also want to work with the paravets, vets, communities, with the commercial farms also.

A: We have also some contact of them, as they regularly buy chicks from us.

MG: Are these twp shops [name of drug shop redacted] *and* [name of drug shop redacted] keep only veterinary medicine?

A: No, no, all types.

MH: Where the *Pranibandhu* and *Pranimitras* buy antibiotics from?

A: From those shops. And from our NGO clinic also they purchase.

MH: Do you have any Medical Representative (MR) who visits here?

A: Regularly visit, mostly 2 companies [names of two pharmaceutical companies redacted] MR meet with me every month. But some other also are coming.

MH: Are they visiting this week?

A: No, no one will come this week here. Sometimes they meet me at Kolkata.

Pabak: I think we met with [name of pharmaceutical company redacted*]*.

A: And huge quantities of medicines are bought from Kolkata wholesale shops.

MG: Do they (MR) talk about antibiotics also? Or what antibiotic do the sale? Are they of only these companies or others also?

A: yes, other company also, other than these 2 company they purchase medicine from Kolkata. Actually from these 2, they directly supply to XXXX. That's why they regularly visit.

MG: Which are the antibiotic do the sale?

A: Say [name of pharmaceutical company redacted], one antibiotic is Sulphatrim injection, amikacin injection and enrofloxacin. And [name of pharmaceutical company redacted] mainly supply feed supplements.

MH: Is that staff is from here who is looking after the goats?

A: His name is [name redacted], he is not paravet but he is working here for more than 1year. That is why he knows which one is giving for which purpose. When the experienced person prescribes, he gives the injection accordingly.

MH: So when you are not here, do they do that?

A: Some other people are there or sometimes they consult with me.
