# Supplementary material for: “If It Works in People, Why Not Animals?”: A Qualitative Investigation of Antibiotic Use in Smallholder Livestock Settings in Rural West Bengal, India
Source: Antibiotics (Basel). 2021 Nov 23;10(12):1433. doi: 10.3390/antibiotics10121433 (PMC8698124; doi:10.3390/antibiotics10121433)
Supplement: Supplementary file 1 [file antibiotics-10-01433-s001.zip › Supplementary S1_ Interview Transcripts/Site 2/Veterinarian 3 (public) (site 2).pdf]

Code for Study - 'If it works in people, why not animals?': A qualitative investigation of antibiotic use in smallholder livestock settings in rural West Bengal, India: Veterinarian 3, Site 2

Interview Date: 1/15/2020

Location: Site 2

Interviewee: Block Livestock Development Officer (veterinarian)- Key Informant/Antibiotic Provider

Interviewer: Mat Hennessey (MH)

Transcript prepared by: Indrajit Patra (IP)

Q- Mat Hennessey

A- Veterinarian

Q: First let us know the infrastructure?

A: In block level: BLDO (Block Livestock Development Officer), under BLDO two hospitals are there 1. Block Animal Health centre(BHC) 2. Additional Block Animal Health Centre(ABHC). And at G.P. level Animal development Aids Centre (ADAC) is there under BLDO, under BHC and ABHC. In each G.P. there are 3 Pranimitras and 1pranibondhu. This is our infrastructure. The staffs of BHC or ABHC are VO (Veterinary Officer), LDA (Livestock Development Assistant) and one Group D staff/peon. Same staff strength is in ABHC.

Q: What is the difference between BHC and ABHC?

A: Actually there is no difference; they are located in two different places. BHC is in here under my office at [name of nearby town redacted] and ABHC is at [name of island redacted]. It will take 4 hours by boat to go there. These two centre cover the total block area. Two separate centre two separate area. We have 14 G.P.

Q: In all the block?

A: Yes, all the block.

Every G.P has ADAC (Animal development Aids Centre) but only in 2 ADAC we provide staff. One LDA is present in ADACs(two), other ADACs are vacant. Totally government centre. In every G.P. *Pranibandhu* and pranimitra are present. 3 Pranimitras and 1pranibondhu per G.P.

Q: In [name of gp in site 2 redacted] is there ADAC?

A: [name of gp in site 2 redacted] also has 3 pranimitras and 1 pranibondhu. But no LDA, the ADAC is vacant.

Q: What is the relation of Pranibandhu and pranimitra with the government?

A: We select them. They provide vaccination and first aid. For this purpose they get something from the government. We selected incentive scheme. Incentive is given to them for their work.

Q: Can you give example of that?

A: They do vaccination and they get some money for that from the government. They get at the rate of rupees 5/large animal and rupees 2/small animal.

Q: Do the Pranibandhu also vaccinate?

A: Yes.

Besides these, different scheme is also going on like FMD-CP and PPR-CP two vital CP (control programme) is going on. To eradicate the FMD and in case of goat PPR, just like Polio in human to eradicate it. FMD eradication programme and PPR eradication.

Q: What is PPR?

A: It is an important disease of goats.

Q: What is the difference between pranimitra and pranibondhu in terms of their work they do?

A: No differences now. They are doing AI works also.

Q: Does pranimitras also do AI?

A: Yes. It was not before. But after completing training to them, they also do AI now.

Q: What are the types of scheme do you have to help the people with livestock?

A: Many types. Generally for economic development chicks distribution programme, goat distribution, pig distribution and also heifer distribution programme is done.

Q: Any other camp like awareness?

A: Yes, on regular basis we organize awareness camp, animal health camp in every month.

One infrastructure I missed is MVC(Mobile Veterinary Clinic).

Q: Who are the staffs there?

A: It consists of one VO, one LDA and one Peon. Actually their work is to organize animal health camps in every island. Minimum 20 health camps they perform in every month.

Q: What happens there?

A: It is an island based area. It is difficult to reach every place at proper time. It can reach there.

Q: Is there any camp going on today?

A: Yes, today also camp is going on.

Q: Where?

A: I have to see the list. They perform the camps everyday. 20days camp. Their work is only to perform animal health camp in different villages of this island.

Q: What type of works done there?

A: Animals are treated and vaccinated. AI is also performed.

Q: What type of treatments the animals get in the camp?

A: All type of treatments is done here in BHC. Not only deworming, they do operation also in centre clinic but not in the field. In centre all type of facilities available. They do castration also. They also do slight diagnosis. Laboratory facilities is available here.

Q: In which places the diagnostic facilities are there?

A: Here in BHC and ABHC. It is permanent centre. They are doing this things.

Q: What type treatment happens in the mobile veterinary clinic?

A: Mainly worm problem and gynaecological problem. Mainly these two types of problems are seen.

Q: When does the vaccination happen?

A: They are doing vaccination also in mobile clinic.

All the centres do vaccination with the help of pranimitra and paranibondhu. *Pranibandhu* and *pranimitra* also present when there is health camps.

Q: How do the people pay for the treatments through vaccination camps?

A: Actually in centre there is some registration fees. We are calling it user charges. We take 5 rupees from large animals and rupees 2 for small animals. Only in BHC and ABHC centres not in other areas. Not in the camps, in field everything is free. In camps all is free.

Q: Are there any type of regulation do you follow during treatments?

A: Prevention of cruelty regulation we have to keep in mind, only one.

Q: Do you follow any state level guidelines for use of medicine?

A: Actually there is no such state level guideline in veterinary. In human they have every guideline in treatments that you are talking about. They are doing something. No such permanent here.

Q: What is your view upon that?

A: It is needed. Surprisingly there is no SOP (standard operation procedures). SOP is not running here.

Q: What types of guidelines you think you needed?

A: There are some government guidelines regarding some disease control programmes like Brucellosis, FMD, PPR. But overall special no guideline.

Q: What are the problems, challenges that you face on animal health, well beings?

A: Here main problem is this is an island are. Till date people of different island people not know the activity of or the facilities they can avail from the government. We are doing awareness programmes.

Q: Why do they not avail?

A: Due to communication problem. Some island is there you can't go there. They are rearing mainly small animals like goat, sheep, chicks and poultry.

Q: What types of diseases mostly occur?

A: In goats PPR and goat pox mainly occur, we are trying to eradicate it. In poultry RD (Ranikhet disease) and in cow FMD. We are trying our best to eradicate these diseases.

Q: What type of people with livestock mainly avail the govt. facilities?

A: Mainly women. Actually women are interested regarding animal rearing. They are literate but not up to the mark. They can only sign.

Q: How would these women access the government facilities?

A: We are trying to train them about the modern technologies of animal health.

Q: Can you give us the example of this technology?

A: Different vaccination programme, hand on training also given like how to vaccinate them, we show them. Our main objective is only prevention. In case of animal prevention is the best thing. We always tell them vaccination is the best way to prevent diseases. In every awareness camp we are talking about the vaccination.

Q: How many people would come in one day?

A: 80-100.

Q: Does they have to pay?

A: No, free camp.

Q: What about the number of animals in the camp?

A: Large animal around 50-60 and small animal around 200 per camp.

Q: What is the proportion of animal that would be vaccinated?

A: Last year we have vaccinated all the large animals (FMD) of [name of adjacent GP to site 2 redacted]. It is about 50,000. We are trying our best.

Q: When did it happen?

A: Last August.

Q: What about the goat and chicken?

A: PPR vaccination is recently we are doing to cover all the population. Near about 50,000 goats has been vaccinated.

Q: Is this happening only through camps?

A: Through camps and by going home to home also. Pranimitras are doing in home to home.

Q: In poultry?

A: In poultry also RD vaccination is going on both in camps and home to home. We are distributing 28days chicks. Last year we have distributed 30,000 chicks. We are giving 10chicks per head to 3000 beneficiaries. [name of GP in Site 2 redacted] people also get these chicks. We are giving to self help group people through 'sangha' (committe).

Q: Who are the members of these 'sangha'?

A: If you want to get information take their name and phone number. Clear picture of [name of site 2 GP redacted] they can give you. Almost every women of each family are connected with them through self help group. There are 2-3 persons, you can contact with the secretary. Actually 'sangha' control 200 -500 groups, 10-12 women present in each group. Around 2000-5000 women are under control of them.

Q: What about the vaccination of those chicks that you are giving at 28days old?

A: We are giving two vaccines before 28days and the 3rd is given at 1.5months. The 3rd vaccine is given by pranimitras.

Q: Which vaccines is that 3rd?

A: R2B vaccine (Ranikhet).

Q: What breed of chicken you give?

A: RIR.

Q: Are they Kuroiler?

A: No, Road island red. It is for egg purpose.

Q: Where do you breed/hatch the chicks that you distribute?

A: Here is no hatchery.

Q: Where it is taken from?

A: It is taken from govt. farm?

Q: Where is the govt. farms?

A: [names of villages outside of site 2] farms

Q: How far way is that?

A: [name of village redacted] is in Kolkata. In south-24-pargana also [name of village redacted]. They supply the chicks.
Actually the chicks are first supplied to mother group, they brood the chicks and rear up to 28days. From there the chicks are taken for distribution to field level. This is the procedure. We are also having mother group at Biprodaspur that is in another island not here.

Q: How many times in a year do you distribute the chicks?

A: All the time of a year.

Q: What the mother groups do?

A: They take the day old chicks from farms. Rear up to 28days and distribute. This is the procedure.

Q: Have there been any problem in the scheme or any concern about how the schemes are working?

A: Till date there is no problem. They are doing vaccination in time. They are efficient for brooding. They have well infrastructure. They are rearing 5,000-20,000 chicks at a time.

Q: What about commercial farms in this area?

A: We arrange for them. Commercial broiler farms are here not any layer farms.

Q: How many?

A: Almost 200 in total block.

Q: What about their number of broiler?

A: 100-200 or 500 in each farm.

Q: How many of those commercial farms are there in [name of site 2 GP redacted] G.P?

A: 10. Here are also company people who supply everything to farmer and take the birds at last from farmer. Actually these are not commercial only 200-500 birds are there, not more than that.

Q: Do these farms use BHC or ABHC?

A: They take their many facilities from BHC and ABHC, they contact the VO and take him.

Q: What about commercial goat farm or dairy farms?

A: Actually there is no such farm like that. In 13number G.P. and even [name of site 2 GP redacted] G.P. peoples also rear goats. Many individual rear 20-25 numbers of goats in [name of site 2 GP redacted]. Minimum 10goats are there in many houses.

Q: And cows?

A: Rare. In [name of site 2 GP redacted] there is present but in other islands there is absent.

Q: Why?

A: Actually peoples are very poor. They cannot provide feed to the large animals. So they rear only small animals like goat, sheep and chicks.

Q: What about the medicine supply?

A: Government supply medicine here. Some they prescribe and the farmers buy it. In centre we provide only government supplied medicine. But it is not sufficient.

Q: Why do you think they are not sufficient?

A: Antibiotics of whole year stock is run only up to 4months. Less amount is supplied. All finished in 4months. Only deworming medicines are available throughout the year.

Q: What type of antibiotics do you have?

A: Broad spectrum antibiotic. VO will say. About treatment he (VO) can give you clear picture.

Q: Do you have any idea of proportion of poultry of commercial farms and backyard farms?

A: In case of layer it is 100% backyard. No layer farm is there. Broiler is always kept in shelter. In case of chicken meat 80% comes from backyard and 20% from commercial broiler farms.

Q: Do the people prefer the backyard chicken?

A: Yes.

Q: So where do the broiler go?

A: It is taken but the amount of production is less. Backyard poultry (deshi) are tastier.

Q: So where does the broiler used mostly?

A: Here in Gosaba. There is huge market but the supply is very low. In case of demand supply is only 40%. Only 30- 40% of demand is supplied from local farms and around 60-80% comes from other place.

Q: What type of people would buy broiler instead of buying backyard?

A: Middle class people. They prefer the *deshi*(Indigenous breed) first then broiler.

Q: Have you heard about antibiotic resistance?

A: Actually govt. supply antibiotics but maximum they had to buy from shop.

Q: Did it happen that medicines are given but it is not working?

A: Yes, this problem is there. They are not maintaining the antibiotic course. Actually if you provide all the medicines this is one of the reasons, party (owner) is not interested to purchase it.

Q: How much is given from the government?

A: The government is giving when they come on 1st day or 2nd day but they not come here from 2nd day or discontinue it. Actually VO will tell it.

Q: Suppose the government have enough antibiotics. Do you think they will complete the full course then or not complete?

A: Actually the animal owner is also negligent. If you give them medicine and it get little bit cure then not come again. It is one of the reason for discontinuation.

Q: Why is this?

A: Lack of awareness. They are not aware about course of antibiotics.

Q: When you have supply of antibiotics, does it come at a time?

A: It come 3times in a year. As we order them they try to give it. But it is depends on allotments, medicine purchase. They send us on basis of how much fund govt is giving. Last year we got more medicines but this year we got very less medicine. The budget is very less. They give us a list and ask to choose among the list what to take. If I order 50 vials of penicillin they provide only 7-10 vials. Actually it depends on fund. Last year many lakhs of rupees were in fund. This time they said very less funds is available. There is a fund problem.

Q: The fund of block?

A: No, it is of district level.

Q: At one time how much supply do you get?

A: What to say... they send quarterly medicine.

Q: What is this supply chain?

A: From district to BLDO, then BLDO to BAHC and ABAHC and ADAC.

Q: Who decides how much to give to each of them?

A: The district level decides it. Sometimes the VOs also decides the medicine that come in the block according to their need.

Q: Why so many of the ADAC are without a LDA?

A: The aid centres also get govt. supplied antibiotics from here. The post is vacant as the government has not recruited.

Q: Do those two LDA stay in their aid centre or they move around the aid centres.

A: They are fixed.

Q: Where are those aid centres?

A: In [name of GP outside of Site 2 redacted] GP, [name of GP outside of Site 2 redacted] GP and another one is in [name of GP outside of Site 2 redacted] GP. Not in [name of site 2 GP redacted].

We will come again and seek your ideas. We are working very closely with the state level and global think tank.
