# Supplementary material for: “If It Works in People, Why Not Animals?”: A Qualitative Investigation of Antibiotic Use in Smallholder Livestock Settings in Rural West Bengal, India
Source: Antibiotics (Basel). 2021 Nov 23;10(12):1433. doi: 10.3390/antibiotics10121433 (PMC8698124; doi:10.3390/antibiotics10121433)
Supplement: Supplementary file 1 [file antibiotics-10-01433-s001.zip › Supplementary S1_ Interview Transcripts/Site 2/Veterinarian 4 (public) (site 2).pdf]

Code for Study - ‘If it works in people, why not animals?’: A qualitative investigation of antibiotic use in smallholder livestock settings in rural West Bengal, India: Veterinarian 4, Site 2
Interview Date: 1/15/2020
Location: Site 2
Interviewee: Block veterinary officer (veterinarian)- Key Informant/Antibiotic Provider
Interviewer: Mat Hennessey (MH)
Transcript prepared by: Indrajit Patra (IP)

Q- Mat Hennessey
A-Veterinarian

K13:1/15/2020: Veterinarian [Site 2]

Q: Would you prefer English or in translation?

A: Translation.

Q: How long are you working as a Veterinarian here?

A: About 1 year 8 months.

Q: Where did you work before?

Redacted life history.

Q: And what type of work are you do here as a Veterinary officer?

A: Actually, Block animal hospital is related to treatment and disease controlling programme.

Q: What type of treatment do you do here?

A: In this area we generally treat the two types of livestock goat and cattle.

Q: And in what type of condition peoples generally bring goats here for?

A: Goats generally suffer from different bacterial and viral disease, fever, coughing, off-fed, diarrhea, and animal owner also come for deworming.

Q: And what types of problems with cattle?

A: Same types, off fed, worm infestations.

Q: What type of treatment do you provide for goats?

A: For off-fed condition probiotic bolus and also some ayurvedic medicine like Himalayan batisa, HB Strong is provided. For deworming we provide fenbendazole tablet and now Govt. has supplied Closantel oral solution.

Q: What types of treatments are given for fever, cough, and diarrhea?

A: We normally treated with the Govt. supplied medicines. Govt provide us antibiotics like injection oxytetracycline, oxytetracycline LA (long acting), enrofloxacin, sulphadimidine bolus.

Q: Any other antibiotics?

A: Yes,there are so many antibiotics like injection Ceftriaxone, amoxicillin+cloxacillin.

Q: And which of these antibiotics you use most commonly?

A: In maximum cases injection ceftriaxone and enrofloxacin.

Q: And why do you use those antibiotics most than others?

A: Actually, oxytetracycline and other antibiotics do not work properly.

Q: Why those are not working properly?

A: Because those drugs (oxytetracycline, sulfa drugs and other drugs) are already used previously by the quacks.

Q: And what do you mean by the quack doctor?

A: They are not professional doctor; they got information from some other trained doctor. They have no BVSc degree or other veterinary degree.

Q: Where they are getting training from?

A: Actually it is seen some centres provide some training illegally some treatment techniques, operation techniques from outside area (may be Kolkata or somewhere else). Here is no such centre.

Q: How do you come to know that oxyteracycline is not working?

A: We have seen the prescription of owner and ask what type of drugs they have used. They tell us that sir, he/she has come and given this drug for 5-10days and it is not working. Then we have come to know that.

Q: What types of people come to you to seek treatments here?

A: All are marginal farmer and the small farmer having only 10-12goats like that.

Q: Which area do they come from?

A: Actually it is an island area. Some people come with animal and some without animal. They are not able to come with animal as the area is surrounded with river.

Q: What percentage people come with animals and what percentage without animals?

A: Actually 20-22 people come here daily, among them 10 people come with animals.

Q: So most come without animals?

A: Yes, without animals.

Q: What are the animals they come with?

A: Goats, indigenous zebu cattle (Deshi) and sheep, poultry birds. Last 4-5years we have treated some Gir cross animals (cross breed Gir).

Q: Do the owner also take cows here?

A: Yes.

Q: Do cattle come daily?

A: No, no.

Q: How often do you see cow here?

A: Daily minimum 1 case.(may be talking about all cow cases including with animal or without animal)

He shows the 2018 case entry *khata* (case register) of a day.

Q: Is that 1/3rd cow 2/3rd goat?

A: Yes.

Q: What types of problem do the people come with?

A: Animal is thin and off-fed, they ask for deworming medication and vitamins. Last time we got some viral diseases, many lumpy skin disease cases.

Q: What did you do for that?

A: There is no specific treatments but we gave antibiotic oxytetracycline daily for 5days, anti allergic injection and paracetamol injection and told to apply a cream. Also use levamisole at alternate days.

Q: Where do the farmers get oxytetracycline from?

A: We prescribe them and give the total 5days medicine in a vial. They inject it with the help of their local paravets.

Q: How much does he (paravet/quacks) cost for the injection?

A: 20-30 rupees per injection daily. We are giving total antibiotic for 5days but other anti allergic medicines, paracetamol injections they have to buy.

Q: How do they know what to buy from the shop?

A: We give prescription.

Q: How much you charge here for 5days medication?

A: Only 5 rupees as registration fees. 1-7days per cow charge is 5 rupees.

Q: Among these cases you treat here, how much cases come to you that have already previously treated by quacks/another doctor?

A: 5-6 critical cases per 20-22 cases.

Q: Do the other 15 cases go any other place before coming to you?

A: They know this place and usually come. Either they taken medicine from the previous VO or know me.

Q: Why those 5 cases go to someone else before coming to you?

A: They (quacks) are locally available to farmer, if it is not cured then come here. Mainly distance problem is there.

Q: What is your understanding of antibiotic resistance awareness?

A: When we diagnose the disease, the animal is suffering from viral or bacterial disease. We provide antibiotic for bacterial diseases. We provide antibiotic in viral disease to prevent the secondary bacterial infection. When we give antibiotic for 3-5 days but the animal is not cured, the problem exists then we think that the antibiotic is not working. Then we change to another antibiotic.

Q: Which antibiotic you changed for?

A: In cattle I have seen some mastitis case where people come to us and say blood is coming through milk, swelling, pain. In that case I have given first Amoxycillin+cloxacillin injection for 3-5 days then animal owner came and reported sir it is not working. Then I had changed the drug to cefitriaxone with enrofloxacin injection. Then it worked.

Q: When these types of problem happen do you do any testing like some sample to send?

A: No, no, no sample is send. I don't know whether any such testing is done or not here.

Q: Do you have any guidelines of what antibiotic to be used in which condition?

A: We follow the treatment history, if owner come with past history of mastitis then we think what type of drug he used first time. If I think it is amoxicillin/cloxacillin then I give some other antibiotics.

Q: Are there any guidelines from the government?

A: No.

Q: Is there any regulation in terms of how you work in this area?

A: No. I treat in my own opinion.

Q: Do any higher level authority come and visit how you are working?

A: Yes. District level officer from Deputy Director come and visit.

Q: How often?

A: 3-4 times in a year. Actually we are having some disease control programme, they come and see the work.

Q: What else they check during visit?

A: In disease control program they go to the village and check whether vaccination has been done there or not.

Q: Do they don’t ask you about treatments?

A: No, no. we send the monthly report of what types of case/disease we treated and how many case we have treated at the end of the month. Not any report of what medicine we are giving.

Q: How often you have to report it?

A: Monthly.

Q: Have you any report now?

A: We are sending it online now-a-days, but it needs to be printed to show you.

(He showed one previous 2017 entry *khata*, the type of cases were contagious, systemic, surgical, gynaecological, parasitic like trypanosomiasis, immature fasciolosis, hump sore, hematuria etc. Most number of cases was with poultry) (Mat want a copy of that, he ensures to give it later)

Q: Do you have any interaction with the quacks?

A: Yes, sometimes they come. They consult also on some cases, operations and then I say I will see the animal first. Sometimes they take us to the village.

Q: What do you think about the antibiotic uses by quacks?

A: They primarily use sulphadimidine, oxytetracycline, amoxycillin. Mainly they use what they have with them. If they have oxytetracycline, they use it on every animal. They don’t diagnose whether it is bacterial or viral. If he has enrofloxacin he uses enrofloxacin for all animals.

Q: Where they get these drugs from?

A: Local medical shop.

Q: Are these human or animal drugs?

A: Veterinary drugs.

Q: Do you think they use human drugs?

A: Yes. Like Human tetracycline tablet, amoxyciliin+cloxacillin capsule, sulphamethoxazole+Trimethoprim tablet, sulphadimidine of human.

Q: What do you think why they use human drugs?

A: They have seen that it cure human and it will also cure animal.

Q: Do you think it is a problem or not?

A: They sometimes not give in proper dose. So sometimes it may not work, resistance may come.

Q: Do the resistance may come in animal?

A: Yes, may be.

Q: Why it may not work?

A: Inappropriate dose. Human and animal mechanism is different.

Q: Are there any situation you had to use human drugs.

A: No.

Q: How do you think antibiotic use can be improved in this area?

A: You have to talk to the medical shops so that they should not give any antibiotic without prescription. Strict rule should be followed for medical shop. They should check doctor prescriptions first then will supply the drugs.

Q: How would that be enforced?

A: By the government.

Q: If that happen what changes would happen in your works?

A: No problem for me rather we could restrict the use of antibiotic.

Q: Any other interventions that could be useful?

A: In this region there are so many areas and are far from my centre. It is always not possible for animal owner to reach us in time. Actually what we government sector we try our best to do all things to control the antibiotic treatment uses, practically it is not possible. Then we should use our information, knowledge to quacks and tell it will be very harmful for us if we use antibiotic indiscriminately in this region. We have to aware about the situations to the quacks or whoever doing the treatments.

Q: How would you will be able to tell the information to the quacks?

A: There have some information from us, it is seen that people who work with the animals Pranibandhu and pranimitras, they know the total how many people are engaged in treatments. Then we can come to know the total number of quacks in this region.

Q: How do you work with the LDA (Livestock Development Assistant)?

A: I have one LDA in my BAHC [block animal health centre]. I prescribe the drug that is supplied by the government and instruct him to do those drugs, he gives/inject the medicines.

Q: Does the LDA himself treat without consulting you?

A: No, if I am not available he asks me over phone.

Q: Does he treat outside the block?

A: Yes, he can do the primary treatments.

Q: Does the LDA of the aid centre also treat?

A: Yes, they treat their own.

Q: Do the LDA use antibiotics?

A: Yes.

Q: These same types or different types?

A: They also use the government supplied antibiotics.

Q: What about the ADAC where no LDA is present?

A: Livestock farmers from there come here to take the medicine.

Q: Is there any situation when you have to go to treat outside your block office?

A: Yes. When it is not possible for the owner to take the large animals to my centre.

Q: What types of problems you see there?

A: Fever, dystocia, mastitis, gynaecological problem of large animal.

Q: How much they have to pay for that?

A: They arrange transport and 200 rupees as visit fees.

Q: Did you have use antibiotics in gynaecological cases?

A: Yes, Gentamicin, ceftiofur Na, ceftriaxone. These are not government supplied medicine, I prescribe that. They buy from local shop.

Q: Why it is not available from government?

A: Government buys medicine in a tender process. The high cost medicines the govt. doesn't buy. The cheap medicine they buy.

Q: Who buys those drugs that you prescribe there like gentamicin? Is it paid by block office?

A: The patient owner themselves buy it.

Q: So is it part of your block office job or separate?

A: It is out of my duty hours. Separate.

Q: When do you do that?

A: After 3:30 or 4:30p.m.

Q: What is your office time?

A: 9:30a.m.-3:30p.m.

Q: How many cases do you see each day after office?

A: Nor everyday. It is frequent. One/twice in week.

Q: Would you take some drugs with you?

A: No. Sometimes I take drugs with me in dystocia case. The cost should be paid by animal owners.

Q: The drugs that come to the block office how often do they arrive here?

A: It is yearly supply. It comes once in a year. It is very less. If we treat properly it can be managed for 3-4months only.

Q: What does happen after the 3-4months?

A: Then we have again told them give some medicine for my center, then again send some. Then we provide medicines to the needy animal owners. Otherwise we prescribe them to purchase the drug.

Q: Which medicine most commonly do you run out of first?

A: Deworming and vitamins.

Q: What about antibiotics?

A: It get finished in 6-7months.

Q: Then what do you do before the next delivery?

A: Some we again want from government, some we prescribe.

Q: How do you decide to divide medicine between BAHC and ABAHC?

A: The district level decides it, mostly same amount they give to all centers.

Q: And in aid center with LDA?

A: Less amount than BAHC and ABAHC. Only the essential drugs. The district level authority also decides it.

Q: Antibiotic also supplied?

A: Yes, some antibiotics.

Q: Does the block buy medicine from the shop to store here when run out of medicine?

A: It is not in protocol. Govt. will not allow us to buy from local shops.

Q: Do you think this supply system is good or need any change?

A: No, need frequent supply. 3months interval they should check and supply.

Q: If medicines come frequently how you will be able to work?

A: We could be able to give more facilities to the animal owners with drug. We can arrange more animal health camps from that supply.

Q: Do you work with MVC (Mobile veterinary clinic) officer?

A: We have selected their place, they do not work in [name of GP in site 2 redacted] G.P. which is accessible for us but in other G.P. which is not accessible for us.

Q: Do they work more with ABAHC?

A: No, same like here. They work except the adjacent BAHC or ABAHC area. Like MVC work other than [name of GP in Site 2 redacted].

Q: Do you have seen use of veterinary medicine are used in human?

A: No.

Q: The ‘antibiotic resistance’ that you are talking about which antibiotic is resistant in your area?

A: I think Amoxycillin+cloxacillin, Oxytetracycline, Sulphadimidine is resistant in more than 70% population.

Interviewer: Do you have anything to ask us?

A: What will happen in your total work? Whom you are going give these data?

Interviewer: When we will finish this phase of work then we will think what type of intervention can be done with all the people, community and stakeholders. And we will try to improve the access of medicine, how antibiotic use can be improved. We are going to give these data to Royal veterinary college, West Bengal veterinary college and London tropical school of medicine
