# Supplementary material for: “If It Works in People, Why Not Animals?”: A Qualitative Investigation of Antibiotic Use in Smallholder Livestock Settings in Rural West Bengal, India
Source: Antibiotics (Basel). 2021 Nov 23;10(12):1433. doi: 10.3390/antibiotics10121433 (PMC8698124; doi:10.3390/antibiotics10121433)
Supplement: Supplementary file 1 [file antibiotics-10-01433-s001.zip › Supplementary S1_ Interview Transcripts/Site 2/Veterinarian 5 (public) (site 2).pdf]

Code for Study - 'If it works in people, why not animals?': A qualitative investigation of antibiotic use in smallholder livestock settings in rural West Bengal, India: Veterinarian 5, Site 2

Interview Date: 1/17/2020

Location: Site 2

Interviewee: Block mobile veterinary officer (veterinarian)- Key Informant/Antibiotic Provider

Interviewer: Mat Hennessey (MH), supported by Indrajit Patra (IP)

Transcript prepared by: Indrajit Patra (IP)

MH- Mat Hennessey

KI- Key informant

IP- Indrajit Patra

MH- Sorry to interrupt, you were saying before about the farmers using antibiotics, can you explain a bit more about that?

IP-What you are saying?

KI- Farmer had used antibiotics in poultry and in livestock animals.

MH- Could you give any examples of the antibiotics that they use?

KI- In case of poultry farm mostly quinolones and derivatives, enrofloxacin, ciprofloxacin, these type of antibiotics is rarely used.

MH- Are rarely used?

KI- Quinolone groups of antibiotics.

MH- And how do people know how to use antibiotics?

KI- Generally it is for preventative use or cure use, both uses are doing. In case of preventative, 1ml of enrofloxacin in 2L of water for five days.

MH- And how do people know how to do that?

KI- In field, we have seen it that the farmer, in case of broiler farming, this type of medicine is mainly used. In case of backyard farming, this type of medicine is not used. The people are very poor and this type of antibiotics are very costly. In case of backyard farming, then tetracycline drug, or amoxicillin drug, or ampicillin drug are used as because the backyard poultry farm these are used, indigenous breed it is resistant, it has got imunity power, in that case the narrow spectrum antibiotic in the case of backyard poultry farming, ampicillin, amoxicillin is best, the broader spectrum of antibiotic, tetracycline is rarely used, in case of backyard poultry farming. In case of broiler farming, that type of drug are not used.

MH- In backyard poultry farming, when do they use antibiotics?

KI- Generally it is routine practice, in case of first month of age.

MH- So they are using the antibiotics routinely?

KI- In case of chicks rearing, first moth of age, that type of routine treatment is done.

MH- Is this in backyard poultry?

KI- Yes in backyard poultry farming?

MH- And how to they know how to use antibiotics?

KI- They are taking advice from government institutions.

MH- Do they take advice from anybody else?

KI- Yes, when we are visiting the field we advise them to use this medicine. Beside this government sector there are already quack practice around, they are there, from them they are also taking advice.

MH- And how may quack doctors are in the area, in [name of site 2 GP redacted] GP?

KI- In every places there are two, one three types of quack practitioner are there.

MH- And when you said the mobile clinic gives advice to backyard poultry, how do you advise them about antibiotic use in the first month?

KI- This chicks at a very tender age, they are very susceptible to environmental disadvantages, they are suffering from cold and cough, there are environmental error, in

case of chicks they are rearing in very poor environmental condition, the environment is very poor, in that case there is a possibility to suffer from such type of pneumonia, coccidiosis, and any respiratory diseases, due to environmental conditions or management error.

MH- And so what do you tell the backyard farmers to do with antibiotics with the chicks, what do you tell them what to do?

KI- When we are visiting that place, they also take bird to us for inspection or to check their animals or birds, then we are trying to use our medicine and to prescribe them to follow these treatment for a period of 4 to 5 days, that is our instruction.

MH- Could you given an example?

KI- When in some cases there are chalky diarrhoea, in that case we already given injection of enrofloxacin or oxytetracycline, this type of medicine we are having, and also given a prescription to purchase form the market, to follow the prescription.

MH- Which market would they go to to get the drugs?

KI- In the local market this type of medicine is available.

MH- Can you give an example of the market?

KI-In the local market this antibiotics like tetracycline, amoxicillin, moxgate, that type of medicine is also available.

MH to IP- Can you ask him where the markets are?

IP-Where the local market?

KI- Local market there are medicine shop in their locality.

MH- And in [name of site 2 GP redacted] GP where are these medicine shops?

KI- Yes yes, for example in [name of site 2 block redacted] block there is a market, [name of nearby town redacted] market

IP-Is there any medical shop in [name of site 2 GP redacted]?

KI- There are no shops in [name of site 2 GP redacted] GP, they purchase medicine from [nearby town name redacted]

MH- And can you ask him, before we were talking about giving routine antibiotics to chicks, can he explain about that?

IP-How use routine antibiotics in backyard?

KI- When they are rearing chicks in some places, or in that type of chicks are supplied form private sector or in government sector, in that case these type of chicks from government sector we have already given advice to use that medicine for 5 days to stop chick mortality.

MH- Can you give an example of how old the chicks are and what medicine?

KI- Yes yes, generally we advise them to use electrolyte, or glucon D for first 7 days of age. And then the antibiotic is oxytetracycline powder.

MH- How old are they when they start to have the oxytetracycline powder?

KI- After four to five days.

MH- And how long for?

KI- For 5 days therapy.

MH- And that's for all the chick?

KI- Hmm, to check the chick mortality.

MH- Do you advise people with backyard chicks to do the same?

KI- Generally in case of backyard this type of advise is given to them. In case of broiler farming, this type is already in private sector they have some expert of their own, and they are doing as per the instruction of their management, private sector

MH- And could you tell me a little bit about how the mobile clinics work?

KI- Mobile clinics, when we are visiting several places, we are organising health camps. In these camps we generally give medicine for de-wormer, vaccination and treat ailing animals and already awareness of disease, in some outbreak or other incidents

MH- Can you ask which areas he works in? Which GPs?

KI- 14 GPs

MH- And is it…

KI- We have to do 20 camps in every month, one camp per day. .

MH- And when was the last time you do a camp in [name of site 2 GP redacted] GP?

KI- Last month, in [village name redacted]

MH- And what type of animals did you see in [name of village redacted] in the camp?

KI- All types of animals come there, some diseased animals, some preventative measures.

MH- Could you give some examples of the types of animals?

KI- Types of animals? Animals in case of cattle, goat, fowl, duck, dog, sometimes cat. These type of animals

MH- And how many animals would you see in one day?

KI- Cattle 60-70, goat 70-80, fowl average 300, ducks 50-60, dogs 2-3, cats 2-3

MH- And do all of these animals come to the camp…

KI- Sheep also came

MH- How many?

KI- Included in the goat numbers- 70-80

MH to IP- Can you just check do all the animals actually come to the camp? Or do some people come without the animals?

IP-Is people came with out the animal?

KI- All animals are they are not present there, they are having these animals, but they are taking medicines for de-wormer, some animals walk to us, we have to vaccinate the animals, these animals are not sick.

MH- So how many animals actually come to the camp?

KI- Cattle about 40-50, goat 50-60, fowl 100-150, duck 20-30, dog and cat 2-3

MH- And what type of ailments, disease do the cattle come with?

KI- In case of cattle, in the last rainy season we have an outbreak of epizootic lymphangitis, cryptococcus infection.

MH- And what treatment would you give for that?

KI- That outbreak happened August to September.

MH- Ah ok, and what treatment would you give?

KI- Treatment? Mainly, preventative medicine care is good for these case, we have to give antifungal, copper sulphate, and phenol to use and these sick animals, the supportive therapy. In case of supportive therapy we are using anti-systemic and broad spectrum antibiotic, oxytetracycline, generally no type of drug is not required, supportive therapy is doing.

MH- And if you give oxytetracycline do you provide, how do the farmers get the oxytetracycline?

KI- The animals come to use, we give the injection, and then to follow we have to prescribe for them to purchase from the market. The oxytet bolus, they have to purchase from market.

MH- Are there any other types of antibiotics that you would use with cattle at the mobile clinics?

KI- Yes, yes, generally we are using medicines like amoxicillin, ampicillin, gentamycin, enrofloxacin, ciprofloxacin that type of medicine we are generally supplied form our authority.

MH- And that type of conditions would you use these anitbitoics for?

KI- Generally for respiratory tract involvement, these cases, we have used amoxicillin, sometimes enrofloxacin with anti-systemic drugs

MH- And what would you use ampicillin for?

KI- Ampicillin, amoxicillin they are the same, a narrow type of antibiotic drug. So in that case both of medicine are used in these type of cases.

MH- And gentamycin?

KI- Generally, we would use in dermatitis cases, in case of eye infection, in case of gynaecological use.

MH- And ciprofloxacin?

KI- Ciprofloxacin is used in case of enteric fever or GI tract infection and gynaecological cases.

MH- And animals with diarrhoea?

KI- We would use tetracycline and sulpha drugs and astringent type of drug.

MH- Can you give an example of a type of astringent?

KI- Kaolin this type of medicine

MH- And out of these antibiotics which are the most commonly used?

KI- Astringents are available in the market.

MH- But out of the… (*list antibiotics*). Which of these do you use most commonly?

MH to IP- Can you explain that?

IP-Which antibiotics used mostly?

KI- Tetracycline group often used and enrofloxacin, broad spectrum antibiotic, this is why this type of medicine are generally used. Amoxicillin and ampicillin are used in specific case, in cold and cough and respiratory case, in the case of mastitis amoxicillin with sulbactam

MH- And for goats what type of conditions would you see?

KI- Goat generally outbreak of goat pox and PPR and we have in this area PPR, in that case we are covered in this outbreak with vaccine, goat pox and PPR vaccine, and when we organise health camps this type of vaccine is used.

MH- What other diseases would you see in goats.

KI- Generally loose stool due to parasitic infection and some cases have respiratory involvement, cough cold pneumonia

MH- What type of medicines would you give for loose stools and respiratory and cough

KI- In case of loose stool we give de-wormer like oxyclozanide and for fascioliasis liver fluke, and we have to cover when we find this type of disease from the stool exam, we have to cover albendazole type of medicine

MH- And for the respiratory disease, for the cough and cold and pneumonia?

KI- Amoxicillin and ampicillin, with anti-systemic – chlorpheniramine

MH- And with the poultry what type of diseases?

KI- Fowl pox and RD also fowl cholera these are the bacterial and viral disease also

MH- What type of medicines would you give for these?

KI- In the case of fowl pox we are generally use broad spectrum group of medicine, oxytetracycline group of medicine with local use of betadine or iodine, povidone iodine

MH- And for RD?

KI- In case of RD there is actually no treatment, and to maintain satisfaction we have to cover antibiotics, broad spectrum antibiotic tetracycline, enrofloxacin

MH- And for fowl cholera?

KI- Generally we use sulpha drug and chlorphenicol

MH- And are any of these of these antibiotics, the chlorphenicol, are any of them…

KI- Bactisol powder, oripriminfjection,bactisol powder sulpha and suplhadiazien and trimethoprim

MH- Are any of these antibiotics human antibiotics?

KI- Yes yes, the chlorphenicol is Paraxin human antibiotic, chloramphenicol composition.

MH- Any other human antibiotics used?

KI- Human antibiotics used- p-floxacin, norfoxacin,

MH- What would you use norfloxacin for?

KI- Norfloxacin, for GI tract infection in case of goat and sheep

MH- And why do you use norfloxacin, why do you use that drug?

MH to IP- Can you ask why he uses the norfloxacin the human drug?

IP-Why Norfloxacin is use?

KI- Norfloxcin gets some better result in case of goat

MH- What were you suing before the norfoxacin?

KI- When the norfloxacin is not available we use enrofloxacin, all are quinolone groups of medicine.

MH to IP- Can you ask him how did he know how to start using the norfloxacin?

IP-How did you know the use of Norfloxacin?

KI- Norfloxacin generally used in human medicine, we use for out family, in that case we are getting best results for some case of diarrhoea or GI tract infection for small animals, we use this medicine for sheep and goat already

MH- And how do you know what does to give to a sheep of goat for norfloxacin?

KI- In case for large sheep and goat 400mg tablet BID, for four days

MH- And how do you know to give 400mg of norfloxacin to the goat?

MH to IP- How did he learn to give 400mg?

IP-How did you know the dose of the durg?

KI- According to size and body weight, according to the size of the animal, this type of medicine is available in the market in 200mg and 400mg preparation, in the case of adult goat we are using 400mg like a human dose

MH to IP- Can you ask him who told him that was the dose to use? How did he decide that was the dose to use?

IP- He calculate according to the dose of the human

KI- As per bodyweight as per the size of the animal

MH- And where do you get the norfloxacin form?

MH to IP- Where does he get the norfloxacin from?

IP-Where you get Norfloxacin?

Ki- It is available in the market, local market, all medicine shop that type of medicine is available

MH- Are there any medicine shops in [name of site 2 GP redacted] GP?

KI- There are no medicine shops in [name of site 2 GP redacted] GP, they collect from [name of nearby town redacted]. Medicine shop there are generally situated at the market level, all panchat has one market also, all GP and that medicine are available there.

MH- So we had the chloramphenicol and the norfloxacin, are there any other which are used to treat animals?

KI – We are getting better results with that type of medicine otherwise we are not purchasing that type of medicine from our end, they are not available from out authority as there are veterinary formulation are not available

MH- And will the norfoxacin be given to any other animals other than goats

KI- Generally not used in large animals, generally only small animals

MH- And why not in large animals

KI- Cattle, that formulation is not available form the veterinary pharmacy

MH to IP- Can you ask him why they do not use the norfloxacin for the cattle?

IP- He is saying the preparation is not available for the cows?

MH- What preparation?

IP- It is a human drug.

MH- But why does he not use the human drug for the cattle?

IP- He says that for the body weight of the goat it is available but if you tink about the cattle it is 300kg bodyweight

KI- If the human drug is used then the fortune is very high, they will not do it.

MH- It is too costly?

KI- Ah ah

MH- And with the mobile camps that you do, how many people run that with you?

KI- Staff? We are a team of three persons, one veterinary doctor, one pharmacist and the last one is attendant

MH- And what does the pharmacist do? What is their job?

KI- They give medicine to people and to the animals also, give injections and medicines

MH- And the attendant

KI- They are a helping hand to give medicines and to control the animals

MH- And what type of training does the attendant have?

KI- They have experience through work

M- Do you they have any formal training?

Ki- Yes yes, they have from our end training also

MH- And the pharmacist, what type of training do they have?

KI- One-year course completed

MH- Where do they do that?

Ki- One-year training course form government institution

MH- And do the livestock development assistant?

KI- LDA? The pharmacist is doing LDA work. The attendant is also LDA, it is livestock attendant. In case of MVC he is attendant, in case of government sector he is assistant, it is different, he is also according to one-year training, government training, this is for MVC, attendant, it is different.

MH- And do the pranimethra?

KI- Yes yes we have three pranimethra in every GP, they also helping us in that places, we inform them to attend it

MH- And how many pranimethra will come to each camp to help?

KI- There are three in each GP, but sometimes one may not be available and there are two available

MH- So depending on their availability?

KI- Sometimes three are available and sometimes two are available

MH- Do you need to start your worknow, do you need to leave?

KI- Yes yes we have a programme

MH- Do you have any questions for me?

KI- This programme you are doing, for what purpose?

MH- We want to understand how people in these communities use health care and antibiotics, to try to think about interventions which may improve the use of medicines. With you work do you have any experience of antibiotic resistance?

Ki- Yes yes

MH- Can you give an example of antibiotic resistance?

KI- In case of mastitis, generally narrow spectrum antibiotics are used but in that time that medicine already resistant on this organism

MH- Can you give an example of a type of antibiotic?

KI- Narrow spectrum antibiotic is penicillin group of medicine, ampicillin, neomycin that is medicine that are now very resistant. In the case of Staph aureus, is a problem creating organism, in that case we are using amoxicillin sulbactam, that type of medicine is very effective, but penicillin streptomycin medicine is not effective, nowadays this type of medicine is not effective, but there are some broader spectrum antibiotics, cephalosporins, but they are very expensive, they are used in dairy type animals, high yielding animals, in that type of group cefoprazone and sulbactum group of antibiotics.

MH- And if we were to try and develop an intervention to improve how antibiotics are using in the area, what type of intervention could this be?

KI- Antibiotic use can be… actually it increases day by day, but if you try to improve the hygiene conditions then that type of medicine can be reduced, but often farmers are not aware about this route of infection

MH- Ok that's great, so if the hygiene was improved…

KI- Due to poor management, generally that is why these type of infection occur, unhygienic cattle shed and unhygienic milking, milking technique.

MH- Thank you we should let you start your work

END OF INTERVIEW
