# Supplementary material for: “If It Works in People, Why Not Animals?”: A Qualitative Investigation of Antibiotic Use in Smallholder Livestock Settings in Rural West Bengal, India
Source: Antibiotics (Basel). 2021 Nov 23;10(12):1433. doi: 10.3390/antibiotics10121433 (PMC8698124; doi:10.3390/antibiotics10121433)
Supplement: Supplementary file 1 [file antibiotics-10-01433-s001.zip › Supplementary S1_ Interview Transcripts/Site 2/Veterinary drug shop 3 (site 2).pdf]

Code for Study - 'If it works in people, why not animals?': A qualitative investigation of antibiotic use in smallholder livestock settings in rural West Bengal, India: veterinary drug shop 3, site 2

Location: Site 2- the shop supplied antibiotics to poultry keepers in Site 2

Interview Date: 1/15/2020

Interviewee: Poultry Shop- Antibiotic Provider

Interviewer: Mat Hennessey (MH), supported by Soumen Samanta (SS) and Pabak Sarkar (PS)

Transcript prepared by: Soumen Samanta (SS)

MH- Mat Hennessey

SS- Soumen Samanta

Pabak- Pabak Sarkar

All answer (A) by the shop owner

MH: Tell him thank you for the time.

SS: Sir is conveying thanks to you as you are giving your time.

MH: How long you have been doing this (running the shop)?

A: 15years.

MH: How did you start the work (business)?

A: Previously I worked in a poultry farm. I got experience from there then started this work.

MH: Is it your only business now?

A: No, only this shop.

MH: Do you keep poultry yourself now?

A: No, now I involve other people to make poultry farm.

SS: How long ago you stopped rearing yourself?

A: 3years. Have farm but I have not get time now.

MH: What services do you provide here?

A: We give chicks, feeds, medicines, vaccine.

SS: At what stage chicks do you sell?

A: Day old chicks.

SS: From where do you get?

A: [name of hatchery company redacted] hatcheries.

SS: Where it is?

A: Kolkata.

MH: What types of chicks are they?

A: Broiler and croiler also.

SS: From where do you get the croiler?

A: [name of farm redacted] farm.

MH: Do you sell more broiler or croiler?

A: Broiler.

SS: What is the %?

A: Fifty-fifty.

SS: Which one is sold more here?

A: Broiler.

SS: Why?

A: As broiler is cheaper, peoples demand is more for eating. As croiler market price is more and this is a poor area, most of the people not been able to eat it.

MH: At how much price do you sell the day old broiler?

A: It varies. Now the rate is 22rupees.

SS: And croiler?

A: 24rupees.

MH: What type of people do you deal with during selling of day old chicks?

A: Here all who come are commercial farms.

SS: Commercial means how much capacity?

A: 200-1500.

Pabak: Where the peoples come from (area)?

A: [names of villages within and outside of site 2 redacted].

Pabak: Is people come from [village name redacted] too?

A: [village name redacted] is under [name of GP (site 2) redacted]. So I don't mention separately.

MH: Do you know in [name of GP (site 2) redacted] how many commercial farms are there?

A: There is less in [name of GP (site 2) redacted]. more in [name of adjacent GP redacted]. In [name of GP (site 2) redacted] about 10 persons.

MH: How many day-old-chicks do they buy at a time?

A: It varies from 200-500.

SS: 500 maximum?

A: Yes.

MH: When they buy the chicks, do they pay cash or it is in credit?

A: People buy less in cash, mostly in credit. Like we give 80%, they give 20%.

Pabak: Can you describe the whole procedure?

A: Such as they pay the chick cost and we give the feed cost, medicine, vaccine cost. At the end of marketing age they sell those birds to us at market price.

Pabak: How they order chicks?

A: They book the chicks number 1 weeks before. And when it reaches a total order of 3000-4000, then I bring the chicks and distribute them.

Pabak: Do you supply these chicks to the house of farmer or they come?

A: No, they take it from here at their own cost.

Pabak: And during taking from company?

A: The company supply it up to [name of river redacted] (the other bank of this river).

Pabak: From there?

A: From there I have to arrange vehicle to take the birds here.

SS: Did it happen that farmer is not able to come to you and you supplied the chicks to his/her house?

A: No. No home delivery facility is available here.

MH: What type of medicine do you supply?

A: Very less antibiotic we do now, Neomycin+doxycycline, some enrofloxacin. Mainly vitamin minerals and liver tonic. We use antibiotic only when disease comes because antibiotics hampers the growth.

Pabak: In disease condition which antibiotic do you give?

A: Neomycin+ doxycycline, enrofloxacin, levofloxacin.

SS: Does the farmers ask for these drugs?

A: No, they come here with the problems.

SS: how much do you give them?

A: The dose is maintained by doctor.

SS: Which doctor?

A: Company doctors are there. Sometimes we (myself) do post mortem and send the picture to company doctors by Whatsapp because it is not always possible for the doctor to come here. They analyse and send advice like this is be given.

SS: When did it happen last?

A: 3-4days ago.

SS: what problem was there?

A: It is winter so brooder pneumonia case is more.

SS: So you have to send post-mortem picture.

A: Yes.

SS: What doctors advised?

A: They prescribed medicine.

SS: What medicine?

A: B904

SS: What is it?

A: It acts as a disinfectant.

SS: What to do with that?

A: That is actually a fungal infection. As it is reared on saw dust so if they do not disinfect the dust for long this respiratory problems happens.

If one rear the animals in closed condition, due to lack of oxygen ascites problem appear.

SS: How B904 is given?

A: In 3litre 1ml is given.

SS: After mixing with water, then?

A: Fed to them throughout 24 hours.

MH: You said 3 antibiotics, any other?

A: If get cold, then Levofloxacin+ Bromhexine(?) is given.

SS: How much do you give that?

A: 1ml/litre.

It is used in CRD.

MH: How do you decide which antibiotic to be given in which condition?

A: Dose is mentioned in vials. In CRD Doctor told this. Doctor of one company generally advice their company's medicine. or MR(Medical representative) also comes and tells their medicine could be given in which conditions. If you are having CRD problem, mycoplasma problem then you can use this.

MH: Which companies MR comes here?

A: [names of pharmaceutical companies redacted]. But [name of pharmaceutical company redacted] do not comes here but their product has high demand.

Pabok: Why?

A: They produce good quality medicine. If people need multivitamin they come and ask for [name of product and pharmaceutical company redacted]. So according to demand we have to keep it.

MH: When you use antibiotics do you follow any schedule or only during sickness?

SS; Do you follow any schedule of antibiotics?

A: Yes, for 4-5days.

SS: When do you use it? They keep the birds up to 35/42 days age, when antibiotics are used?

A: In this winter, for prevention of cough & cold antibiotics has to be given.

SS: How much do you give?

A: 1ml/litre.

Some use enrofloxacin some use neomycin+doxycycline .

SS: How much do you give this neomycin+doxycycline?

A: 1gram/litre drinking water.

MH: When you give these in the life time of those birds (0-42days)?

A: Doxycycline+Neomycin is given from 2-7day period then first vaccine is given.

MH: Do use it at later period of life of that birds in later?

A: No,

SS: If disease happens?

A: Then some better medicine like Levofloxacin+Bromhexine is given if it is due to CRD. In this winter no other problem occurs except CRD.

SS: Any other medicine?

A: In 15-16days there is chance of coccidiosis, which is called fungal infection. Then Amprolium is given.

SS: For 4days?

A: 15 to 20 days.

SS: Do you use Supercox?

A: It is used but less. We do not use supercox as it is sulpha drug. If you use this the growth will be hampered.

SS: Where did you learn it?

A: The company doctors say this. Amprolium not hampers the growth. We have to see, so that no growth is being hampered.

SS: Do you use other medicines except these medicines. Which medication do you give if after 25-30days disease is seen?

A: It depends on the birds conditions. There are not more diseases of poultry except CRD, Coccidiosis, Brooder pneumonia. These 3 are the main diseases.

SS: What do you give in CRD? brooder pneumonia infection ?

A: Give Levofloxacin and bromhexin.

SS: In coccidia?

A: Amprolium.

SS: And in brooder pneumonia?

A: B904 is given.

MH: What proportion of you business do you make by selling antibiotics?

SS: If you sell 100 rupees medicine, how much comes from antibiotic selling?

A: If I sale of 100 rupees, antibiotic 30%, and liver tonic 30% and 40% from vitamins and minerals.

SS: And feed?

A: That is different. You asked about medicine.

We always try to be away from antibiotics as it only causes loss of money and body weight, no profit of birds occur.

SS: Why do you think that antibiotic reduces growth?

A: Vitamin and mineral is supporting the body but antibiotic only act against disease. It is also costly. See this 50gram packet cost 155 rupees. (showing a medicine packet). So it is loss. If he feeds the birds 150 rupees vitamins, that helps in growth. But if disease is there then you have nothing to do, you have to use antibiotic.

SS: Where did you learn that?

A: Our company doctor said that try to avoid as much as possible.

MH: Where do you get the antibiotics from?

A: [name of stockist redacted] And also MR (medical representative) from [pharmaceutical company names redacted] and other MRs come here to give medicine.

SS: These also you have to go to take it?

A: No, they send it in our counter. All they send. Just I have to sit in my shop. You just make payment, goods will be delivered.

Pabak: How many times do you have to order this in a month?

A: It is not fixed, each companies are given order serially. Sometimes they (MR) come and ask for which medicine is going to be finished and later they send the medicine. Come once in a month. Also come to show their new presentation. Actually the representatives come, I need not to go. They tell like this medicine is working better as liver tonic now. We gave this medicine to purify water as here our pH of water is high. This (showing a bottle). Only for purifying water.

Pabak: So those representatives come and take your demand. Then how do you get that?

A: They send it through transport.

Pabak: From where they send it?

A: From where they send, where is their counter they know. We don’t know. We need not to do anything.

Pabak/SS: And in [name of stockist redacted]?

A: There you will get all the medicine, large store.

Pabak: How you tell your demand?

A: They also call and ask for our order. And send it by their transport. They all want to sell their products. Ask why you have not ordered in this month, I say market is not good now.

Pabak: Do they ([name of stockist redacted]) come to you?

A: Only comes during supply of vaccine. Vaccine is a live thing so it has to be send within one day. That time they come.

Pabak: Do they don’t come to take the order?

A: No, only company's representative comes to take orders. [name of stockist redacted] is stockiest. Suppose it has been seen that here is no stockiest for [name of pharmaceutical company redacted], then the company's representative comes and take the order then send it. They take it 1week, as it is far away.

MH: Do you have a preference from where to buy?

A: We always analyse the rates. Who is giving at less price, we take from them.

Pabak: Who is giving good rates of 'antibiotics'?

A: Nobody gives good rates of antibiotics. Actually selling of antibiotics is in loss. No profit is there. Profit is in vitamins, minerals. We gain profit even after selling the vitamins, mineral at discount. We ourselves don't get the discount in antibiotic.

MH: So what makes you to go the different shop or MR for antibiotics?

A: You can't get all medicine from one representative. All medicines do not work the same. Every company has a mother product.

SS: Like?

A: Like in [name of pharmaceutical company redacted] the mother product is [brand name of product redacted] (Enrofloxacin). They also have some other product like [brand name of product redacted- coccidiosis treatment] powder but it not work for CRD, it works in coccidia. If you tell about Neomycin+doxycycline, it is good of [name of pharmaceutical company redacted]. If go for calcium, [name of pharmaceutical company redacted] calcium is very good. Multivitamin is good by [name of pharmaceutical company redacted], that is popular.

SS: Does [name of pharmaceutical company redacted] have only this one antibiotic? Do they not tell you to take their other antibiotics?

A: No, they have. But if they tell to take other antibiotics I don't take it. They have 40-50 items. It is not like that every items are good and we will take that. We take it after sorting it out.

Pabak: How do you sort it out?

A: It depends on their work (efficacy). Like neomycin+doxycycline in also a product of [name of pharmaceutical company redacted], but it not works better than [name of pharmaceutical company redacted] neomycin+doxycycline. A farmer came and told us what medicine you have given, it's not working.

Q: Do you keep one antibiotic of only one company? Or more?

A: Yes, not much. Suppose [product brand name redacted] (enrofloxacin) of [name of pharmaceutical company redacted], but enrofloxacin of [name of pharmaceutical company

redacted] work better than that. We observed that during work. Before we used (Sulpha drug) a lot but one farmer came and told his birds reduced body weight of 500gm after giving it. But when we are giving amprolium it maintains the body weight. This experience I have got.

Pabak: He keeps the one which he seems best according to feedback from the farmer.

SS: Where do you use this ‘Suldin’(also a sulpha drug)?

A: It works better in local rural *deshi* poultry birds.

SS: When do you give that?

A: If farmers comes and tell my *deshi* poultry is in dull and depressed condition, then tell them feed this 5drops for 5days.

MH: Do the companies or wholesaler give any incentive for buying from those places?

A: Most people give incentive in vitamin and minerals like if you take 3 packets calcium, some gives one shocks or one handkerchief.

SS: In antibiotics?

A: Nobody gives on antibiotics.

MH: No discount?

A: No discount.

Pabak: At how much reduction cost do you buy those medicines?

A: Some give 30%, some 25%. It depends on brand value.

Pabak: Whose brand value is more?

A: Whose brand value is more his commission is less. [name of pharmaceutical company redacted] has no commission. Their brand value is so high. No people of [name of pharmaceutical company redacted] come. To whom I will want (incentive) if they not come. Like you came to me I am sharing my experience, if nobody come, will I find people to share my experience?

Pabak: Do [name of pharmaceutical company redacted] have antibiotic?

A: Not much. Probably have but we don’t take it. But their vitamin, mineral is very good. As no MR comes here for the company so to whom I will tell if any problems occur. Who support me I will see their sale. Nobody come from them why I will support their product. But they have many products.

Pabak: Do the people not demand that?

A: No. They do not send doctor. But others like [name of pharmaceutical company redacted], they send doctor who visit farms in their cost and see the problems. If a farmer wants to talk with doctor, they directly also can talk to him. Doctor makes suggestions for them. As I get these facilities from them and a doctor will give his company antibiotics. And he is getting result. One company's doctor will not tell other companies antibiotic. That's why we are linked with [name of pharmaceutical company redacted]. It's a free cost visit by the doctor. If we call them they have to come.

SS: No charge?

A: No charge.

When the one flock is sold out, they also come and spray medicine.

SS: What do they spray?

A: Their disinfectants, to kill the germ which is left by previous flock.

MH: Do the doctor also advise what to do?

A: They tell the farmer. And also to me that he told this farmer to give this medicine. The farmer can't understand the dose. So he tells me also. After visiting them he comes here and tells to give this farmer this medicine.

MH: Here we can see that there are 3 different types of enrofloxacin ([names of 3 pharmaceutical companies redacted]). Why so?

A: Above the 3, [name of pharmaceutical company redacted]'s enrofloxacin works better.

SS: Why do you keep the other two?

A: See, everybody can't able to take the medicine with high price.

SS: Is it like that the high cost medicine works little better?

A: Yes. It's % is more like double strength.

SS: Does the lower cost antibiotics work?

A: Yes, in less cold this works but in more cold time this double strength is given.

SS: Everything works?

A: Yes. Some people come and ask for the brand name [name of brand redacted]. Though each one is enrofloxacin. So I have to give him [brand name redacted].

(Shows varieties of enrofloxacin, their composition, price)

MH: In these all having 10% enrofloxacin, so why you keep these all?

A: Different farmer come ask for different medicine that they know.

MH: Is the price similar?

A: It’s different. (595,700 and 1000 per 1 litre in these 3 formulations)

Pabak: among these 3, which one has more sells?

A: [brand name redacted].

MH: Which one do you think of best quality?

A: [brand name redacted].

MH: Why?

A: Farmers get result.

MH: Do you have any certificate or qualification to run this shop?

A: Yes, I had to do drug licence.

Pabak: From where do you get the certificate?

A: From [town name outside site 2 redacted].

Pabak: What to inform them?

A: First you have to apply then they come and visit the shop. See the purposes wheather i am giving to farmers or wheather I am doing wholesale/retail.

SS: Did you have licence from beginning?

A: No. In the beginning we had not, later we did it.

SS: From when you are having?

A: 10years.

Pabak: How days after you have to renew?

A: Every year.

SS: How much does it cost?

A: Not more, according to govt. rate.

MH: Do somebody comes and check what you are selling?

A: Pharmacist comes.

SS: from where do they come?

A: Outside.

Pabak: How many times they come?

A: Twice in a year.

SS: Is it govt. pharmacist/

A: No, no. Private.

SS: Do they check?

A: No, they don’t. Their licence is needed to run a shop.

MH: Tell him thank you very much.
