# Supplementary material for: “If It Works in People, Why Not Animals?”: A Qualitative Investigation of Antibiotic Use in Smallholder Livestock Settings in Rural West Bengal, India
Source: Antibiotics (Basel). 2021 Nov 23;10(12):1433. doi: 10.3390/antibiotics10121433 (PMC8698124; doi:10.3390/antibiotics10121433)
Supplement: Supplementary file 1 [file antibiotics-10-01433-s001.zip › Supplementary Tables/Supplementary Table S1- Livestock Populations Site 1.pdf]

Table S1. Description of livestock populations kept by households in Site 1 (n= 23)

Livestock Keeper (LK)	No. Cattle	No. Goats	No. Backyard Chickens	No. Commercial Chickens	No. Ducks	No. Pigs
LK1	4	-	-	-	4	-
LK2	-	9	8	-	-	-
LK3	2	-	-	-	-	-
LK4	3	-	-	-	-	-
LK5	7	-	-	-	-	-
LK6	1	-	3	-	8	-
LK7	3	1	10	-	-	-
LK8	-	6	-	-	-	-
LK9	-	-	-	70	-	-
LK10	3	5	20	-	-	-
LK11	12	-	-	60	-	-
LK12	4	-	-	-	-	-
LK13	9	-	Present	-	-	15
LK14	3	-	-	-	-	-
LK15	2	-	-	-	-	-
LK16	-	3	-	-	4	-
LK17	3	-	-	-	-	-
LK18	6	-	-	-	-	-
LK19	3	-	-	-	-	-
LK20	-	5	10	-	-	-
LK21	1	-	-	-	-	-
LK22	2	8	2	-	-	-
LK23	2	2	1	-	-	-
