# Supplementary material for: “If It Works in People, Why Not Animals?”: A Qualitative Investigation of Antibiotic Use in Smallholder Livestock Settings in Rural West Bengal, India
Source: Antibiotics (Basel). 2021 Nov 23;10(12):1433. doi: 10.3390/antibiotics10121433 (PMC8698124; doi:10.3390/antibiotics10121433)
Supplement: Supplementary file 1 [file antibiotics-10-01433-s001.zip › Supplementary Tables/Supplementary Table S2- Livestock Populations Site 2.pdf]

Table S2. Description of livestock populations kept by households in Site 2 GP (n= 14)

Livestock Keeper (LK)	No. Cattle	No. Goats	No. Backyard Chickens	No. Commercial Chickens	No. Ducks	No. Pigs
LK24	1	-	8	-	-	-
LK25	4	10	2	-	-	-
LK26	-	7	-	150	-	-
LK27	3	-	6	60	-	-
LK28	4	5	20	85[1]	-	-
LK29	-	16	-	60	-	-
LK30	1	-	26	-	-	-
LK31	-	-	18	0[2]	-	-
LK32	1	2	3	-	-	-
LK33	1	3	30	30[3]	12	-
LK34	5	8	-	60	18	-
LK35	2	4	20	-	-	-
LK36	2	-	1	-	-	-
LK37	1	2	4	-	-	-

[1] The farm had 600 commercial chickens prior to a recent undiagnosed disease outbreak and recent sale of poultry

[2] The farm had 500 commercial chickens before their recent sale

[3] The farm had 400 commercial chickens before their recent sale
