# Supplementary figures and images for: “If It Works in People, Why Not Animals?”: A Qualitative Investigation of Antibiotic Use in Smallholder Livestock Settings in Rural West Bengal, India
Source: Antibiotics (Basel). 2021 Nov 23;10(12):1433. doi: 10.3390/antibiotics10121433 (PMC8698124; doi:10.3390/antibiotics10121433)

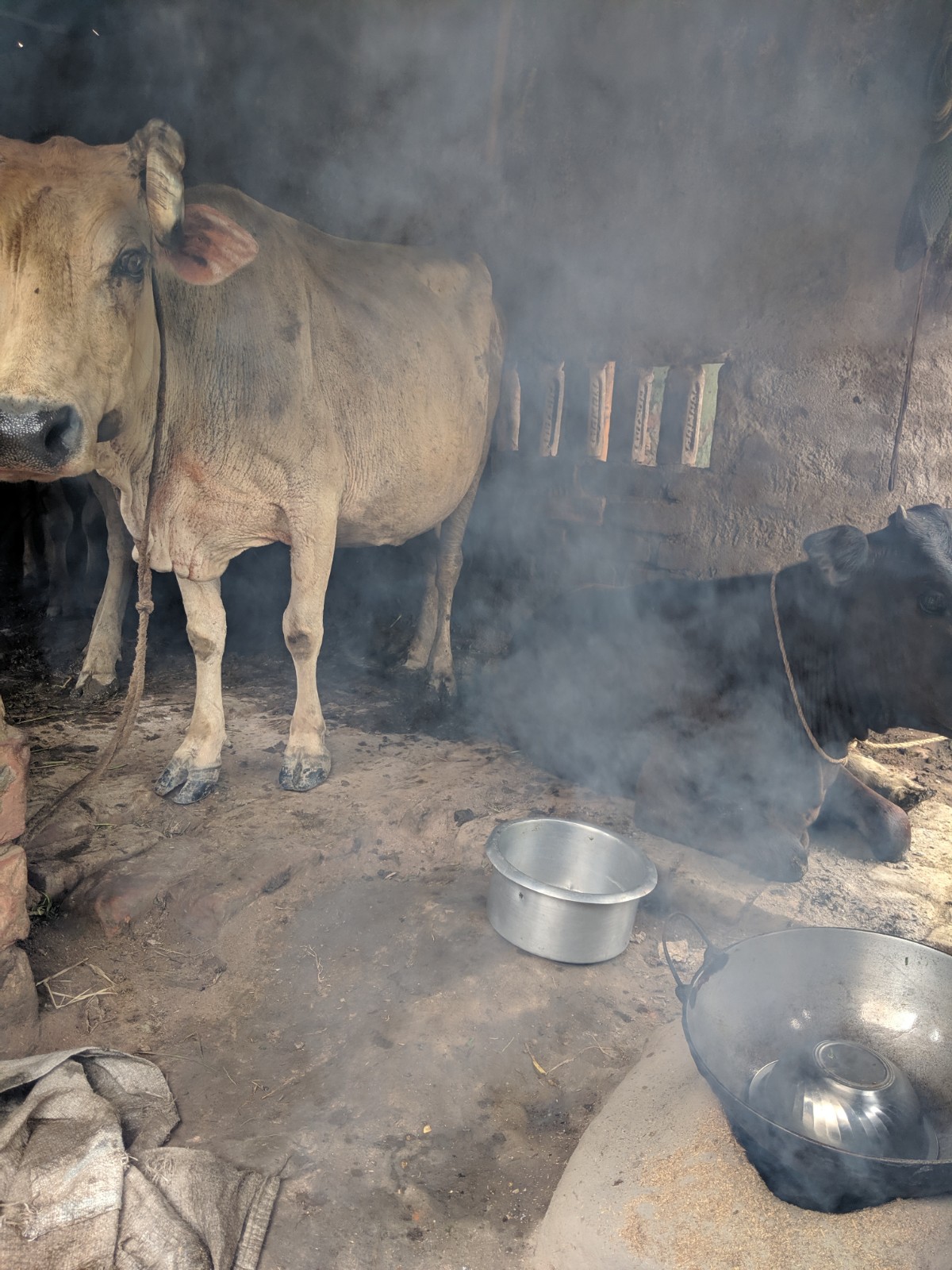

Supplement: Supplementary file 1 [file antibiotics-10-01433-s001.zip › Supplemrnrtary S2_ Site Photographs/Cattle housing 1 (site1).jpg]

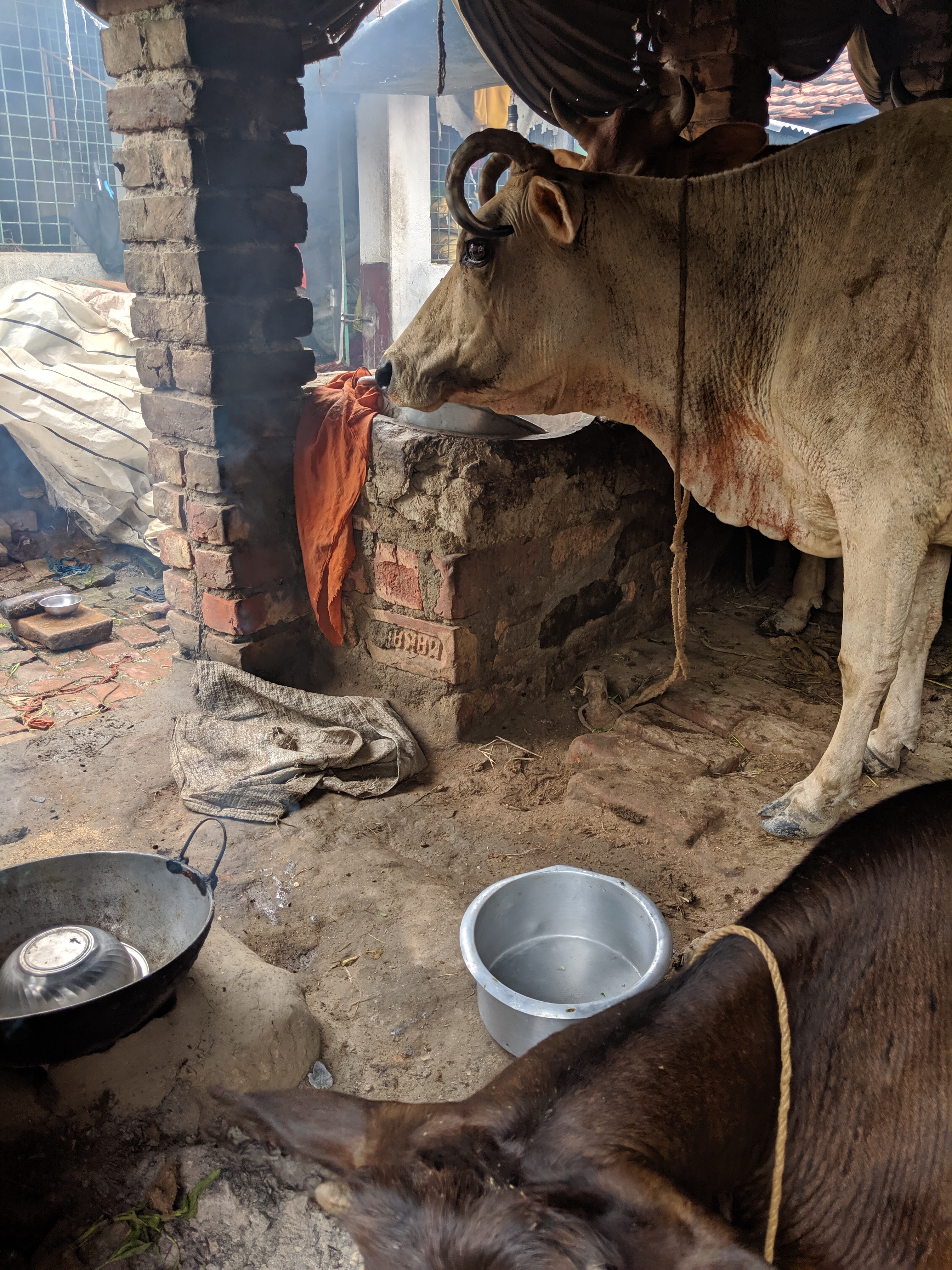

Supplement: Supplementary file 1 [file antibiotics-10-01433-s001.zip › Supplemrnrtary S2_ Site Photographs/Cattle housing 2 (site 1).jpg]

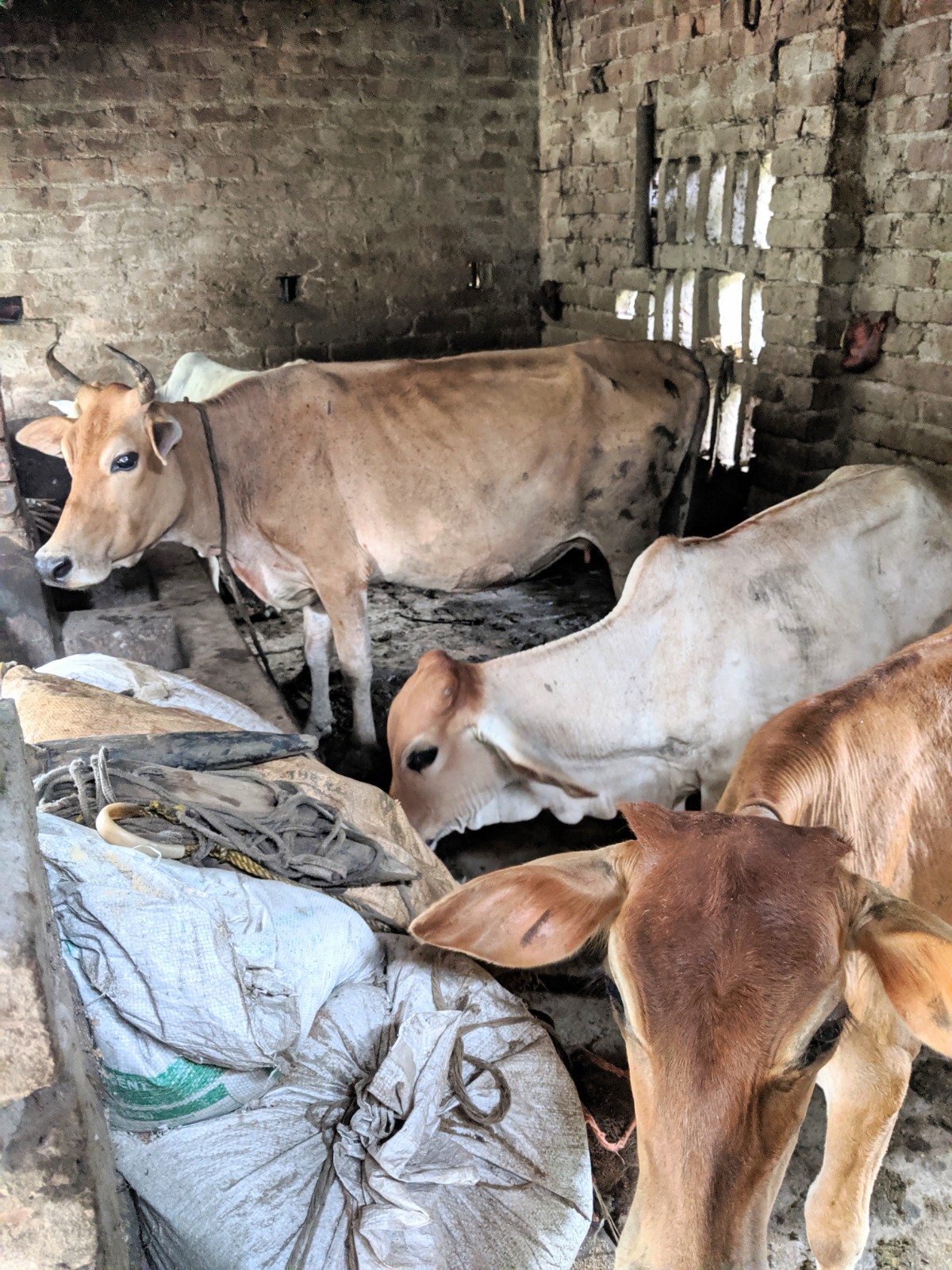

Supplement: Supplementary file 1 [file antibiotics-10-01433-s001.zip › Supplemrnrtary S2_ Site Photographs/Cattle housing 3 (site 1).jpg]

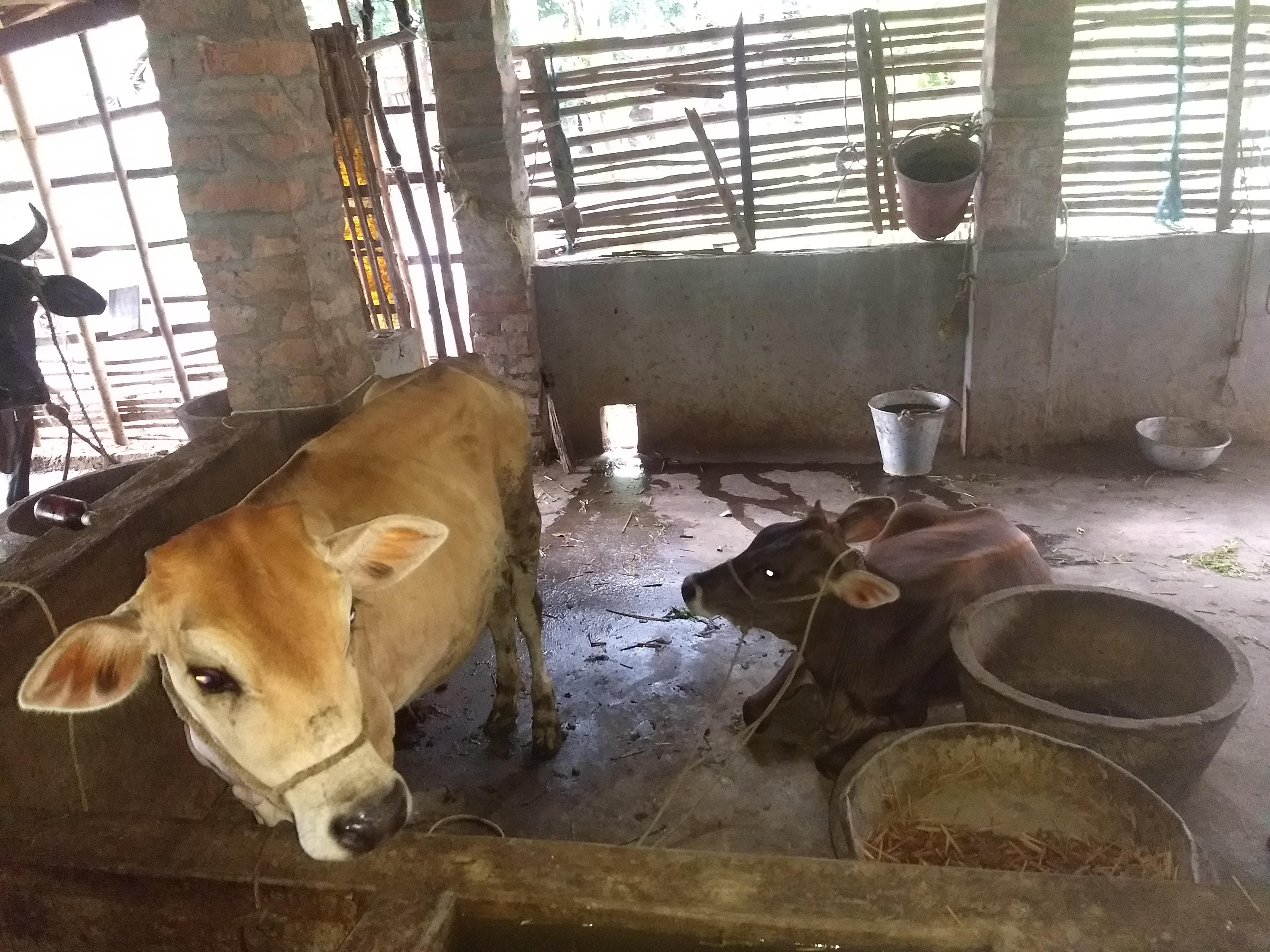

Supplement: Supplementary file 1 [file antibiotics-10-01433-s001.zip › Supplemrnrtary S2_ Site Photographs/Cattle housing 4 (site 2).jpg]

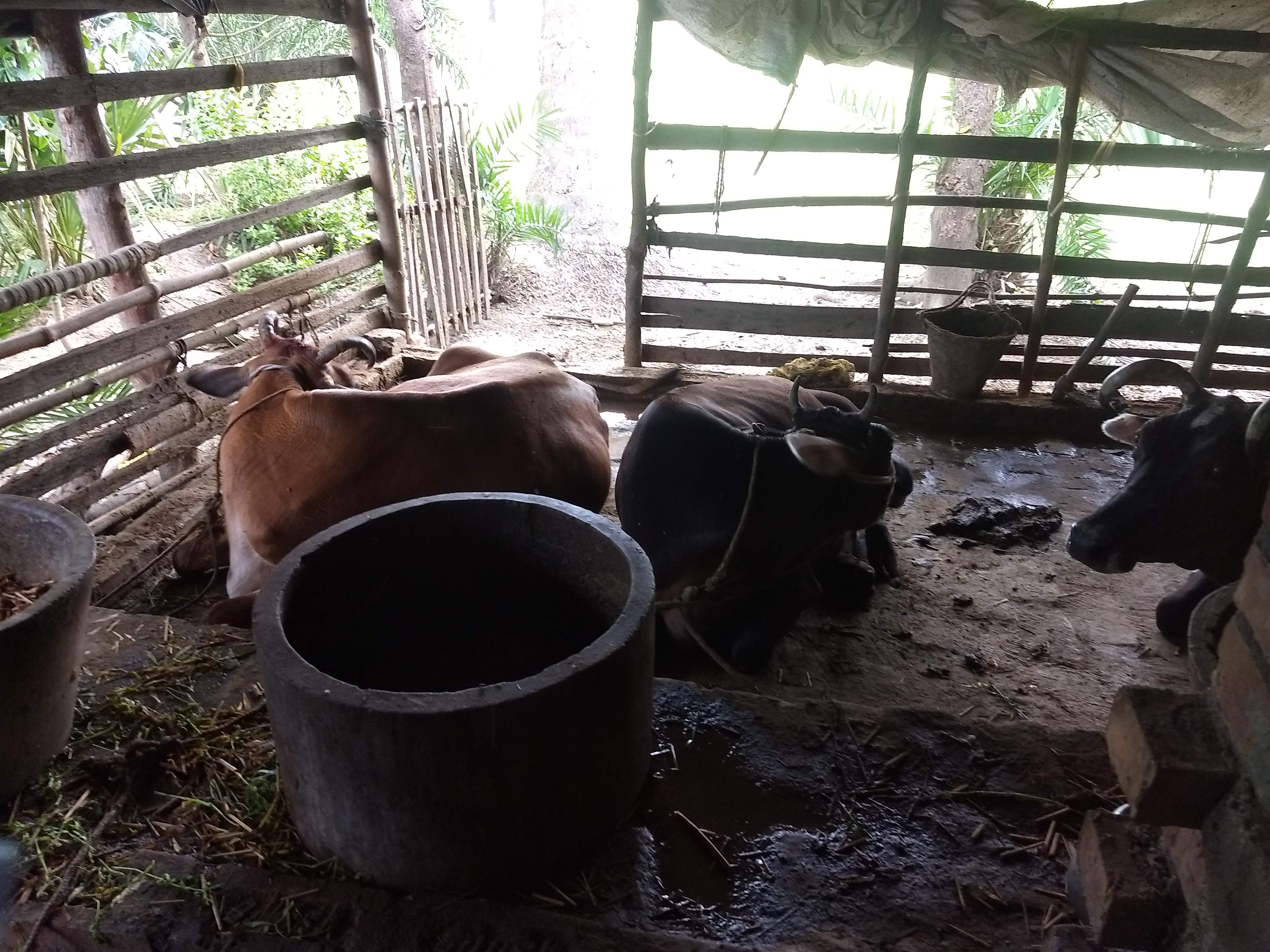

Supplement: Supplementary file 1 [file antibiotics-10-01433-s001.zip › Supplemrnrtary S2_ Site Photographs/Cattle housing 5 (site 2).jpg]

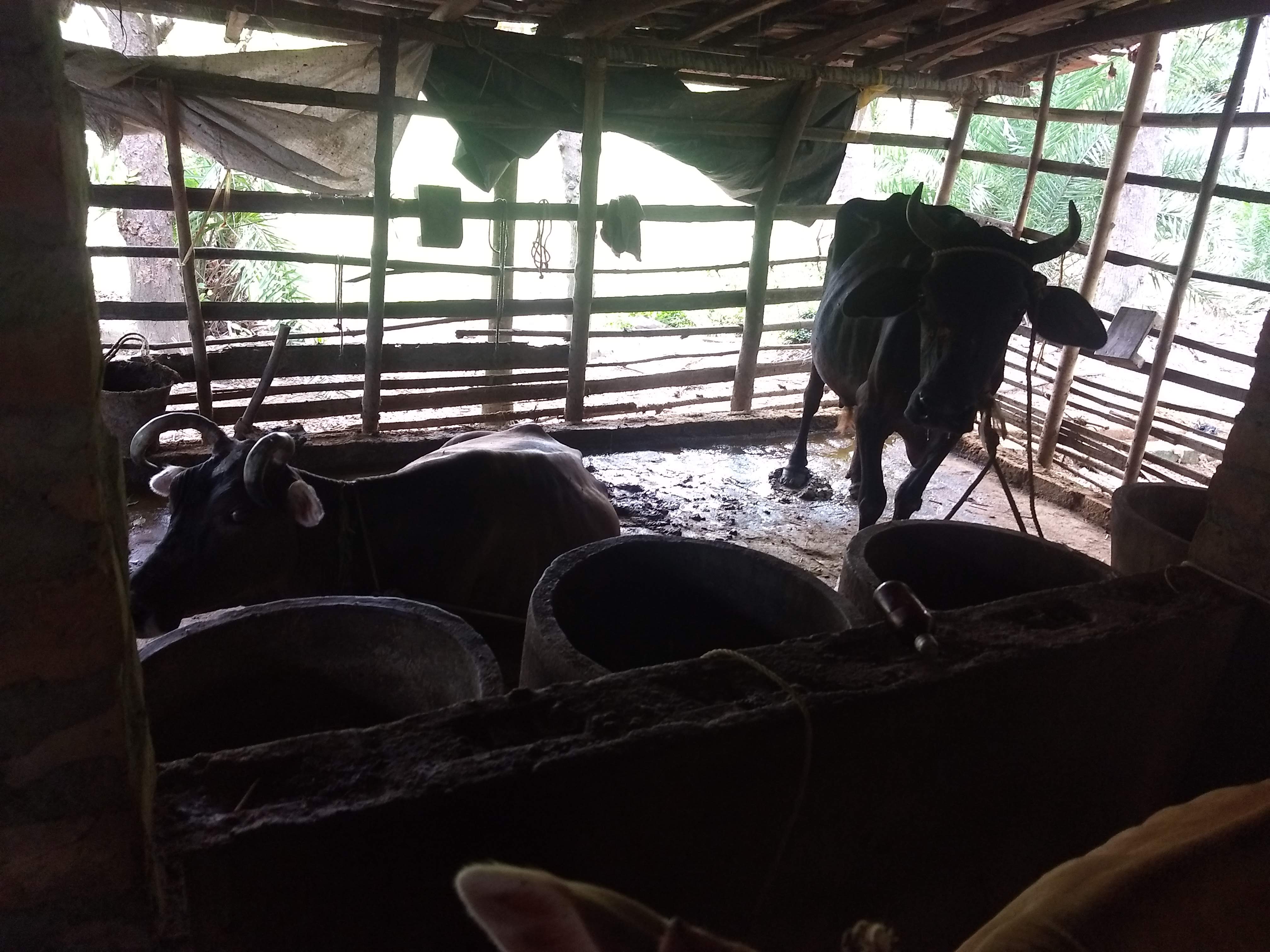

Supplement: Supplementary file 1 [file antibiotics-10-01433-s001.zip › Supplemrnrtary S2_ Site Photographs/Cattle housing 6 (site 2).jpg]

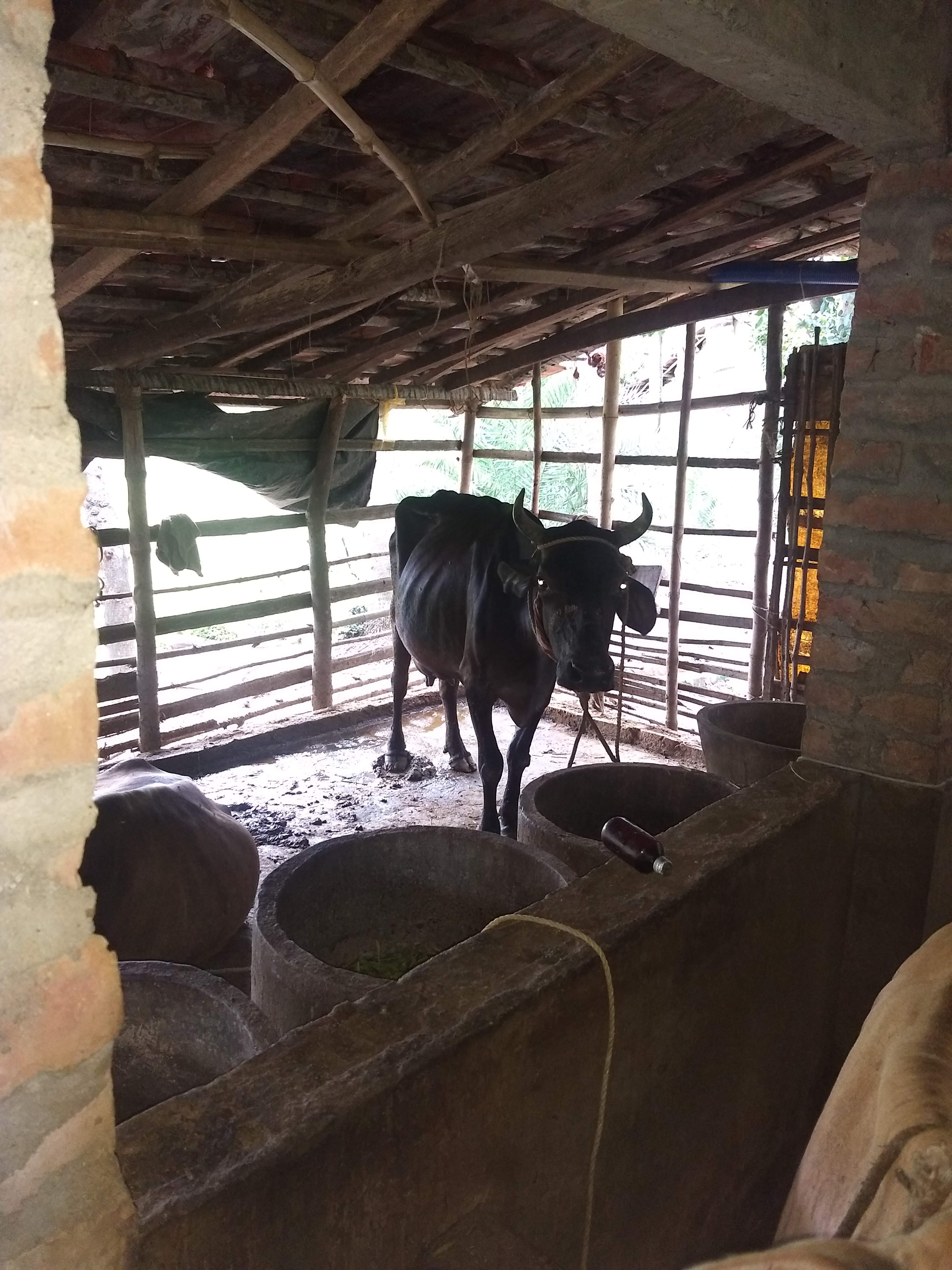

Supplement: Supplementary file 1 [file antibiotics-10-01433-s001.zip › Supplemrnrtary S2_ Site Photographs/Cattle housing 7 (site 2).jpg]

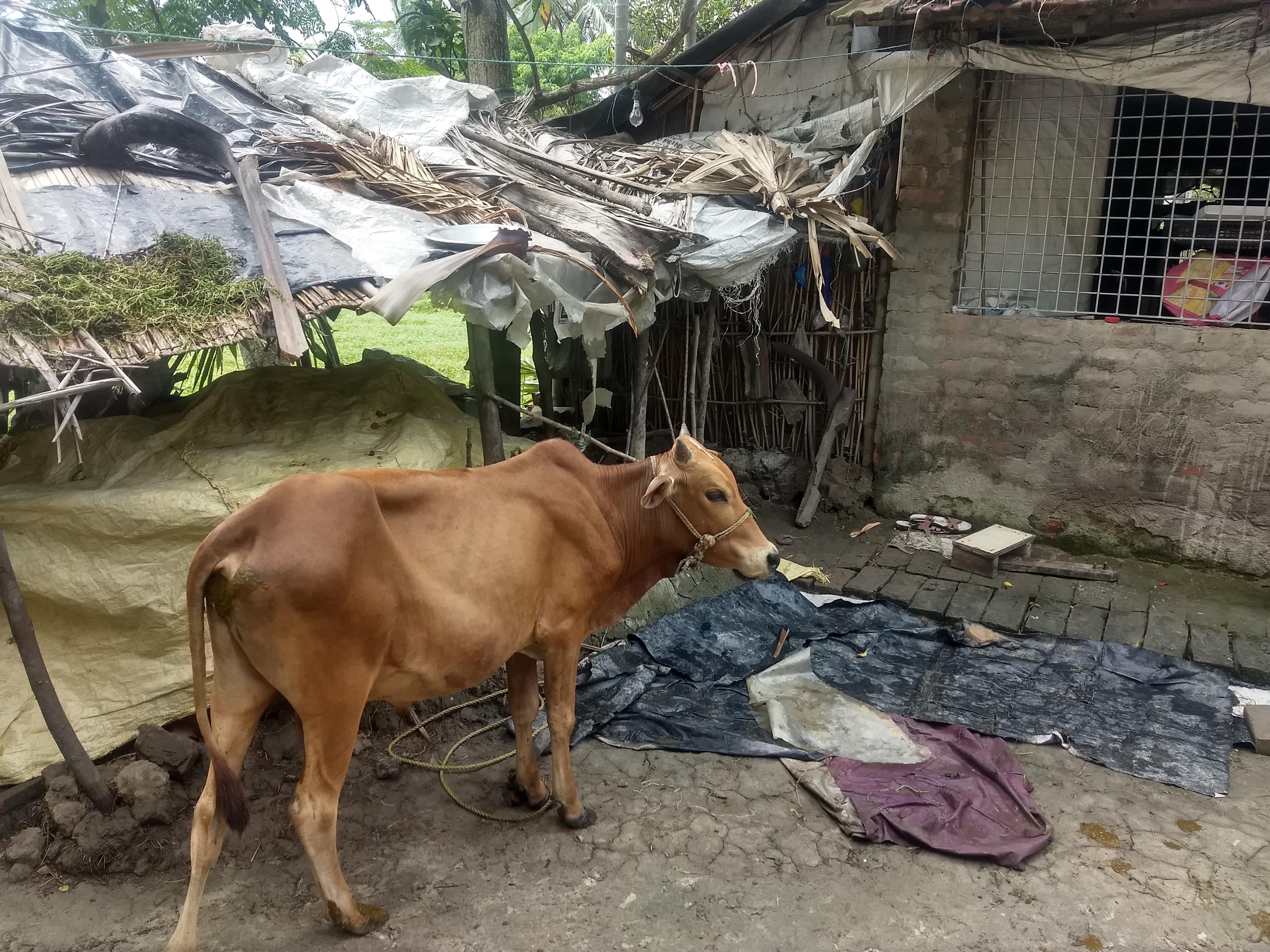

Supplement: Supplementary file 1 [file antibiotics-10-01433-s001.zip › Supplemrnrtary S2_ Site Photographs/Cattle kept in close proximity to household (site 1).jpg]

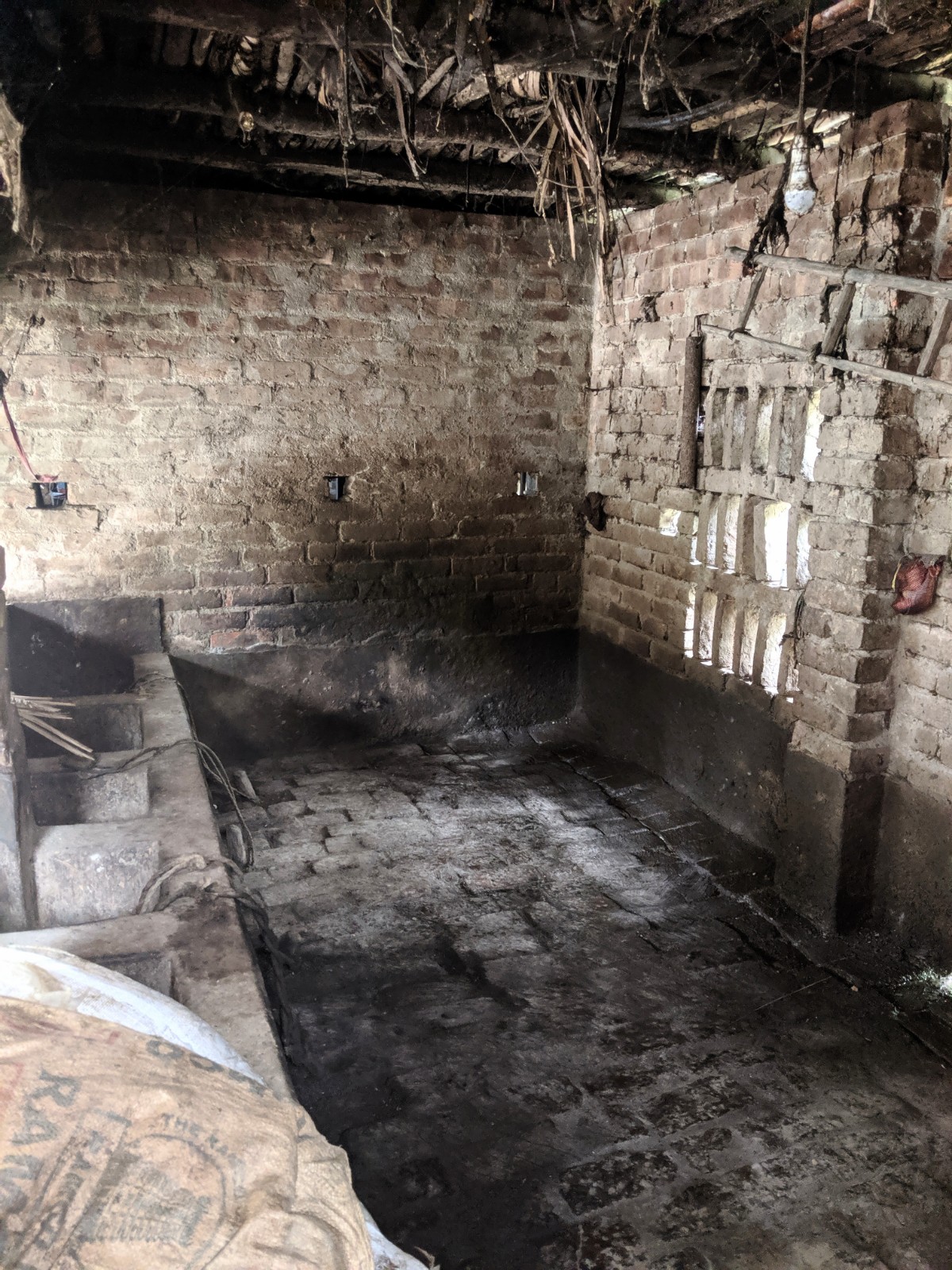

Supplement: Supplementary file 1 [file antibiotics-10-01433-s001.zip › Supplemrnrtary S2_ Site Photographs/Cattle shed 1 (site 1).jpg]

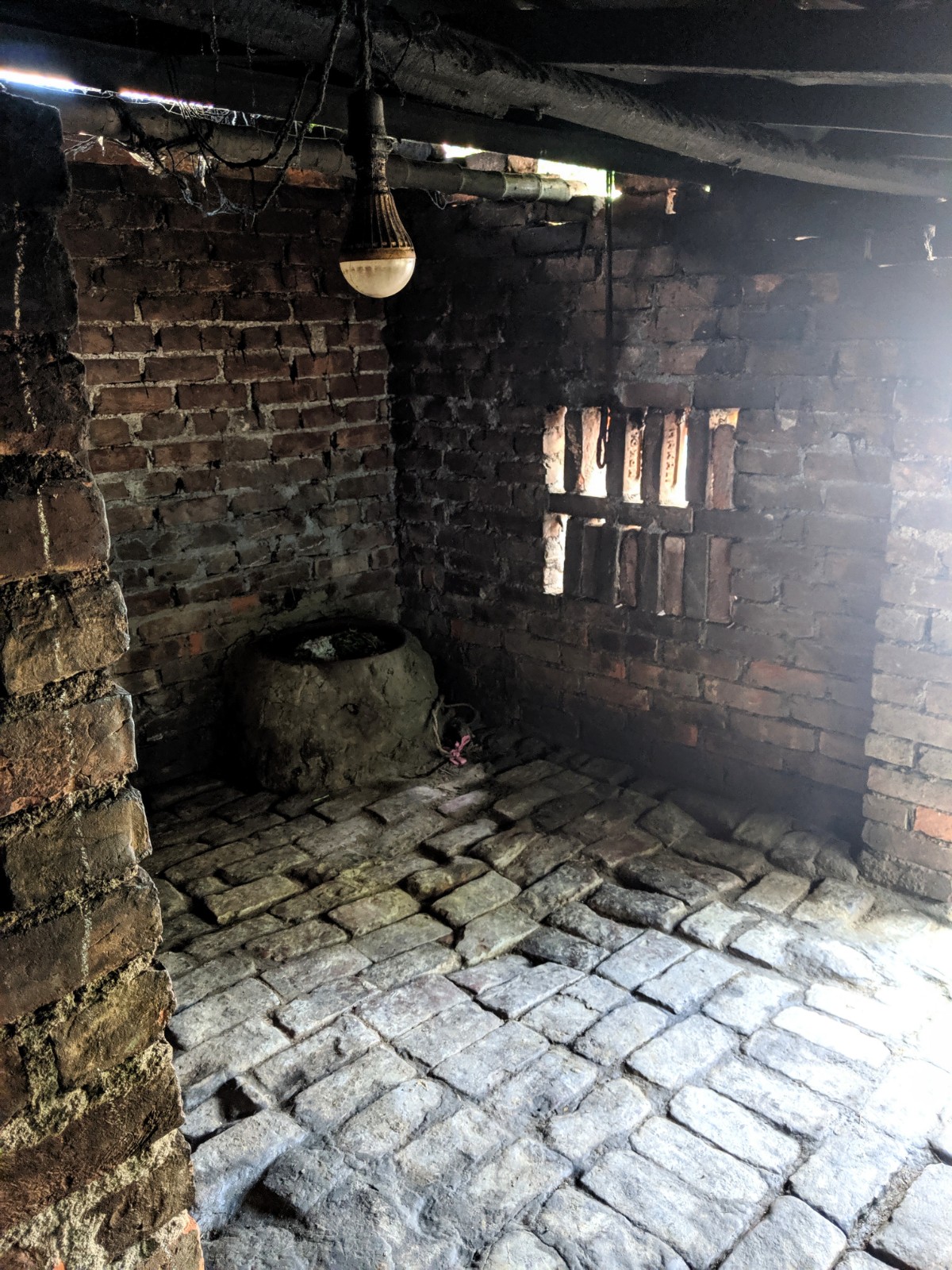

Supplement: Supplementary file 1 [file antibiotics-10-01433-s001.zip › Supplemrnrtary S2_ Site Photographs/Cattle shed 2 (site 1).jpg]

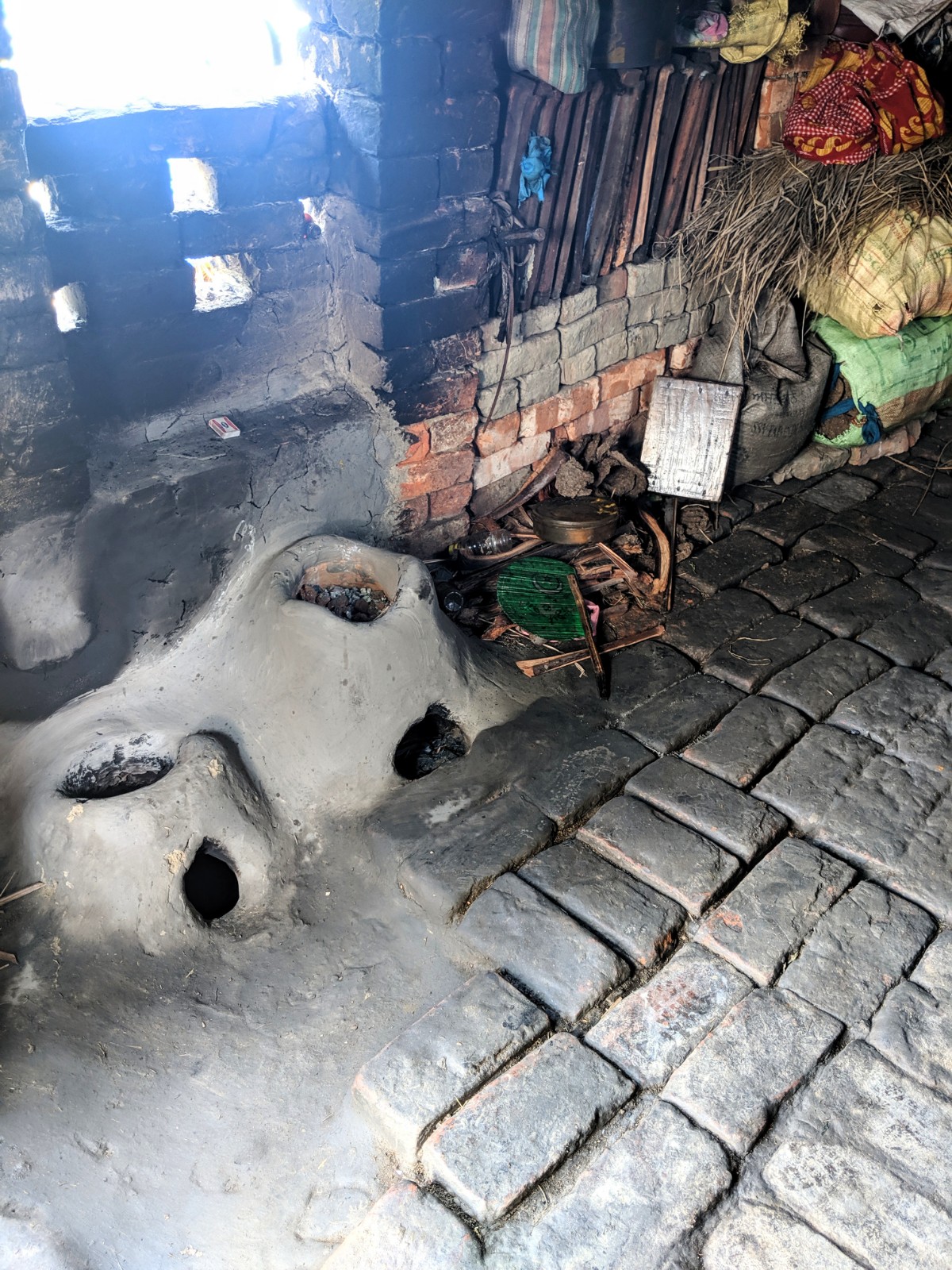

Supplement: Supplementary file 1 [file antibiotics-10-01433-s001.zip › Supplemrnrtary S2_ Site Photographs/Cattle shed 3 (site 1).jpg]

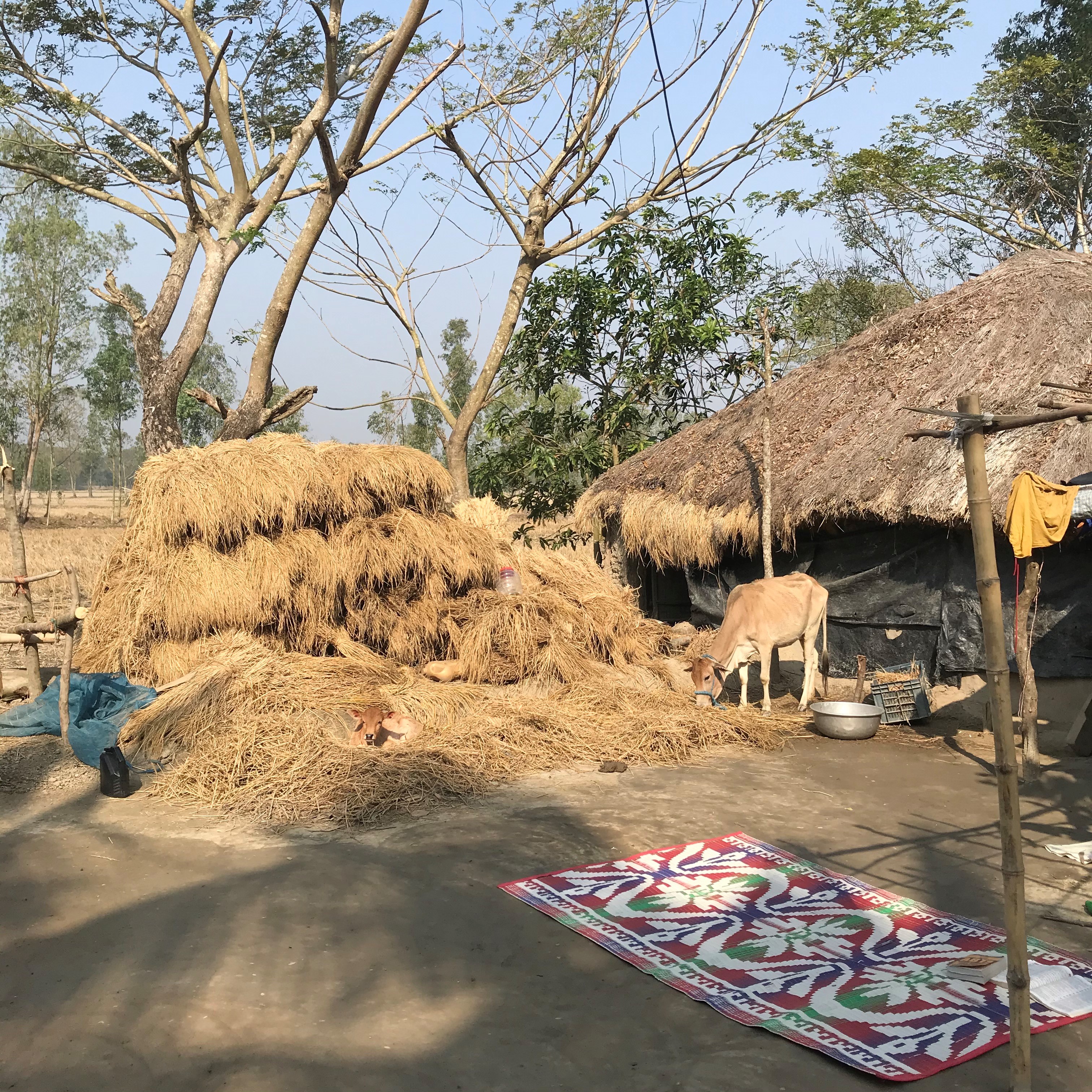

Supplement: Supplementary file 1 [file antibiotics-10-01433-s001.zip › Supplemrnrtary S2_ Site Photographs/Cattle smallholding (site 2).jpg]

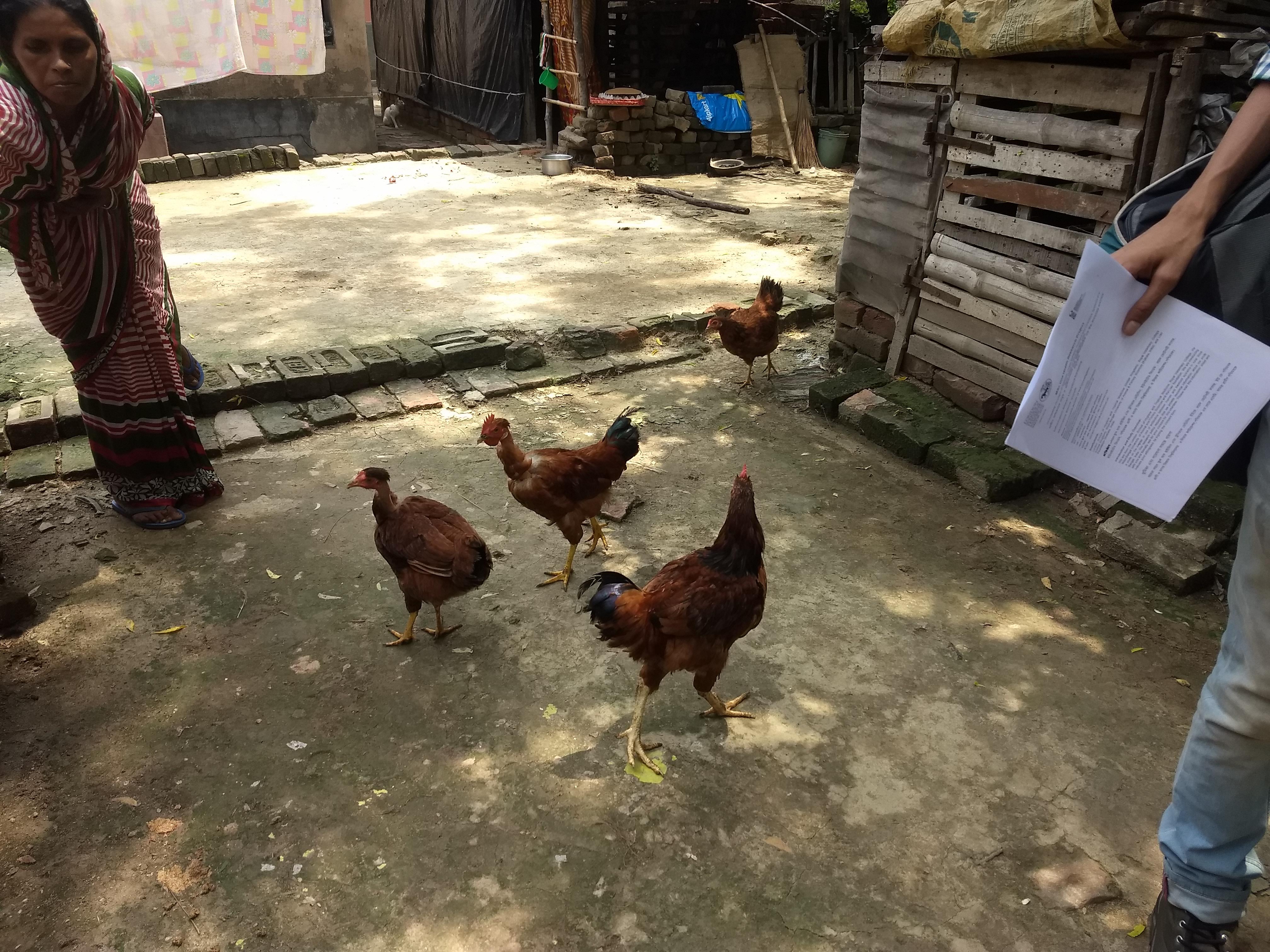

Supplement: Supplementary file 1 [file antibiotics-10-01433-s001.zip › Supplemrnrtary S2_ Site Photographs/Free-roaming backyard poultry (site 1).jpg]

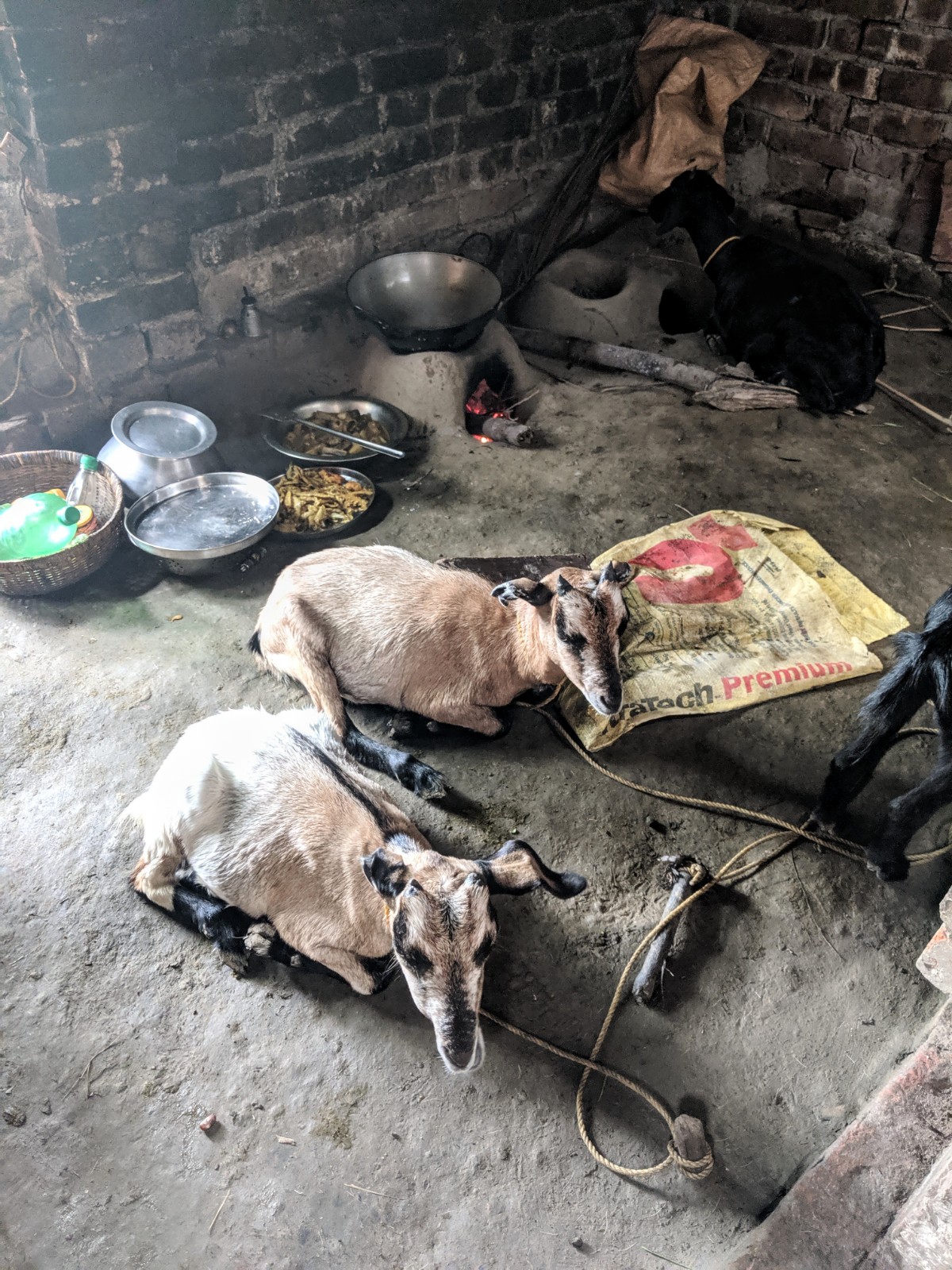

Supplement: Supplementary file 1 [file antibiotics-10-01433-s001.zip › Supplemrnrtary S2_ Site Photographs/Goat housing 1 (site 1).jpg]

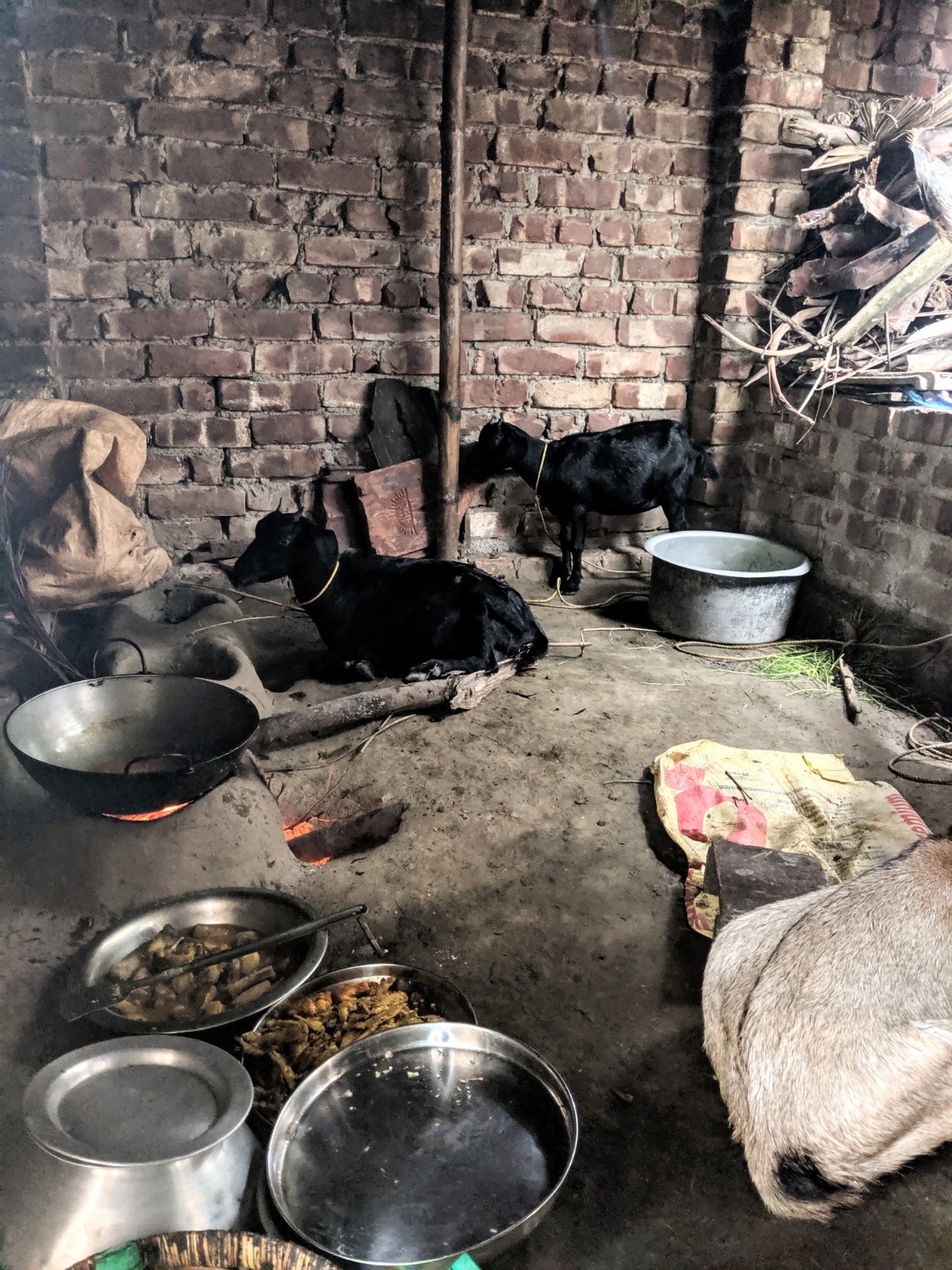

Supplement: Supplementary file 1 [file antibiotics-10-01433-s001.zip › Supplemrnrtary S2_ Site Photographs/Goat housing 2 (site 2).jpg]

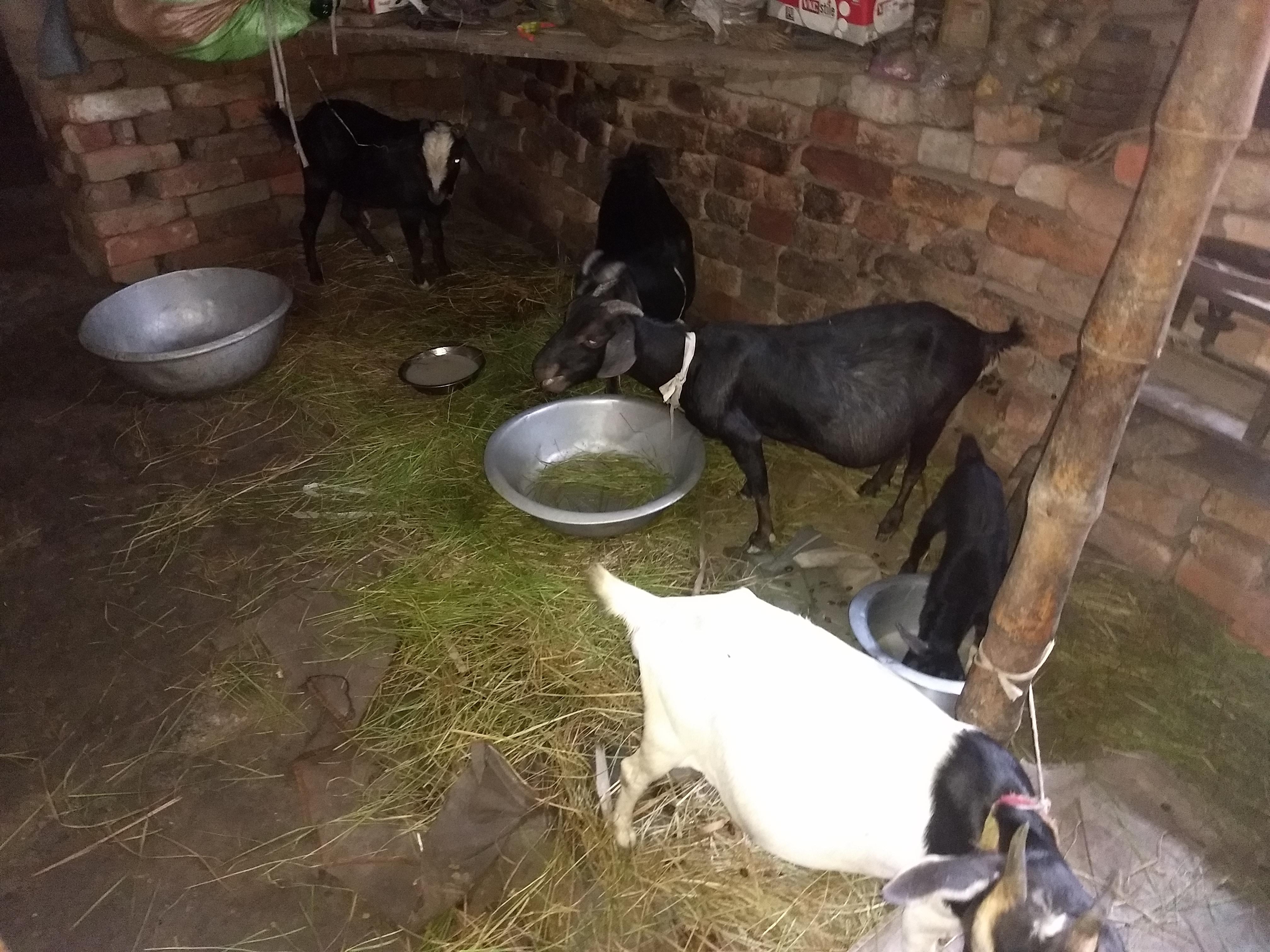

Supplement: Supplementary file 1 [file antibiotics-10-01433-s001.zip › Supplemrnrtary S2_ Site Photographs/Goat shed 1 (site 1).jpg]

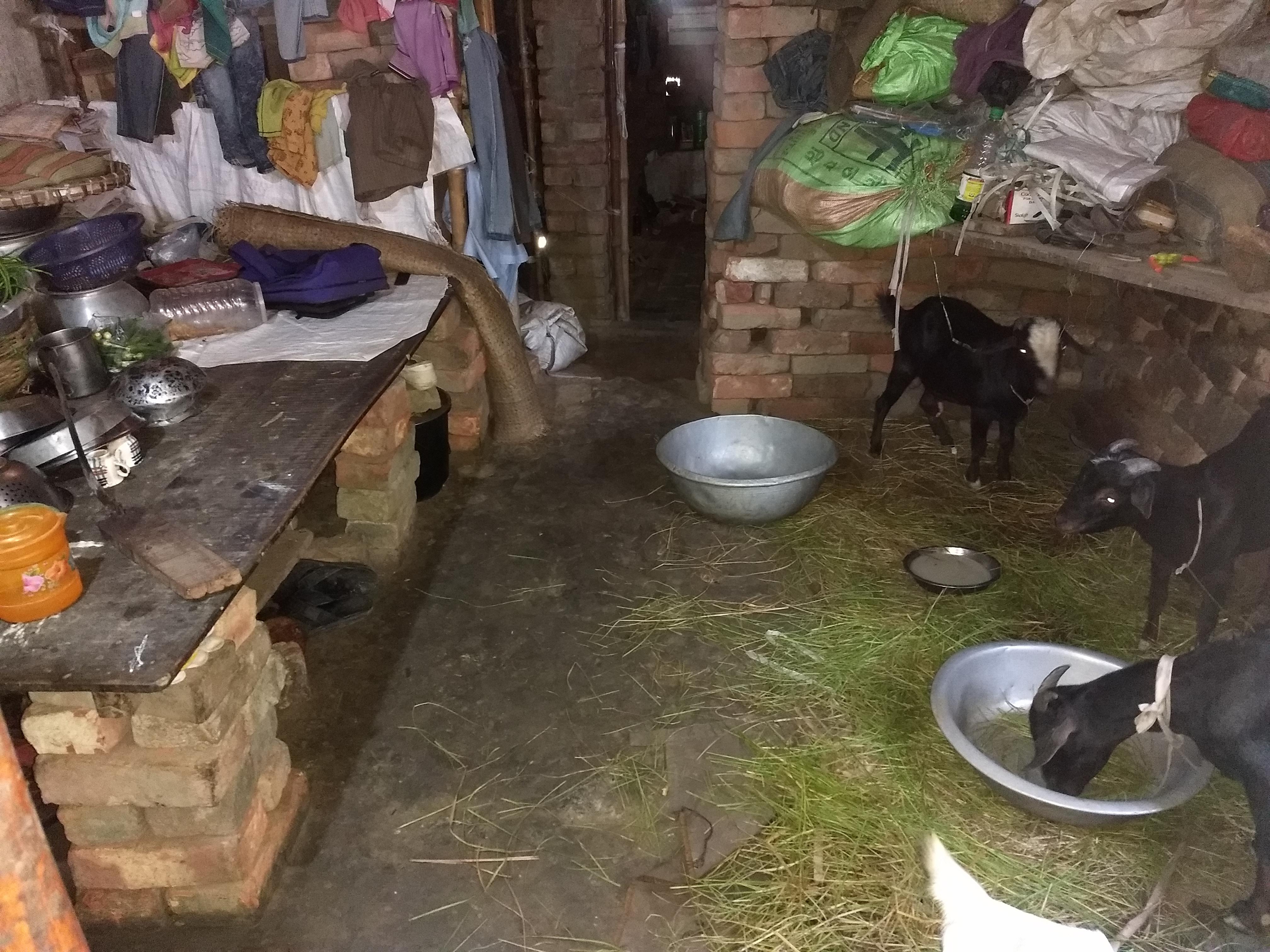

Supplement: Supplementary file 1 [file antibiotics-10-01433-s001.zip › Supplemrnrtary S2_ Site Photographs/Goat shed 2 with human food preparation area (site 1).jpg]

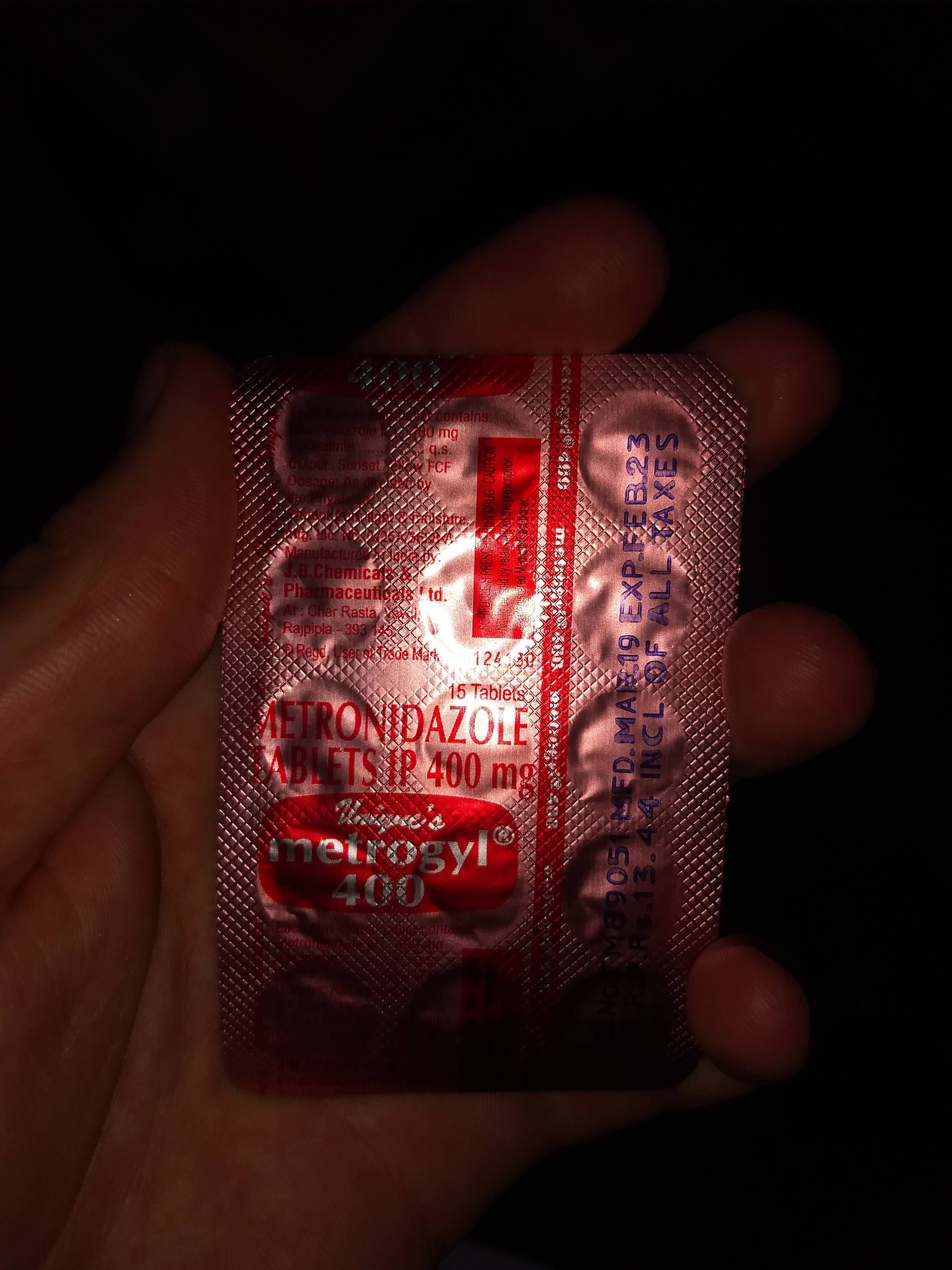

Supplement: Supplementary file 1 [file antibiotics-10-01433-s001.zip › Supplemrnrtary S2_ Site Photographs/Human antibiotic (metronidazole) used in livestock 1 (site 1).jpg]

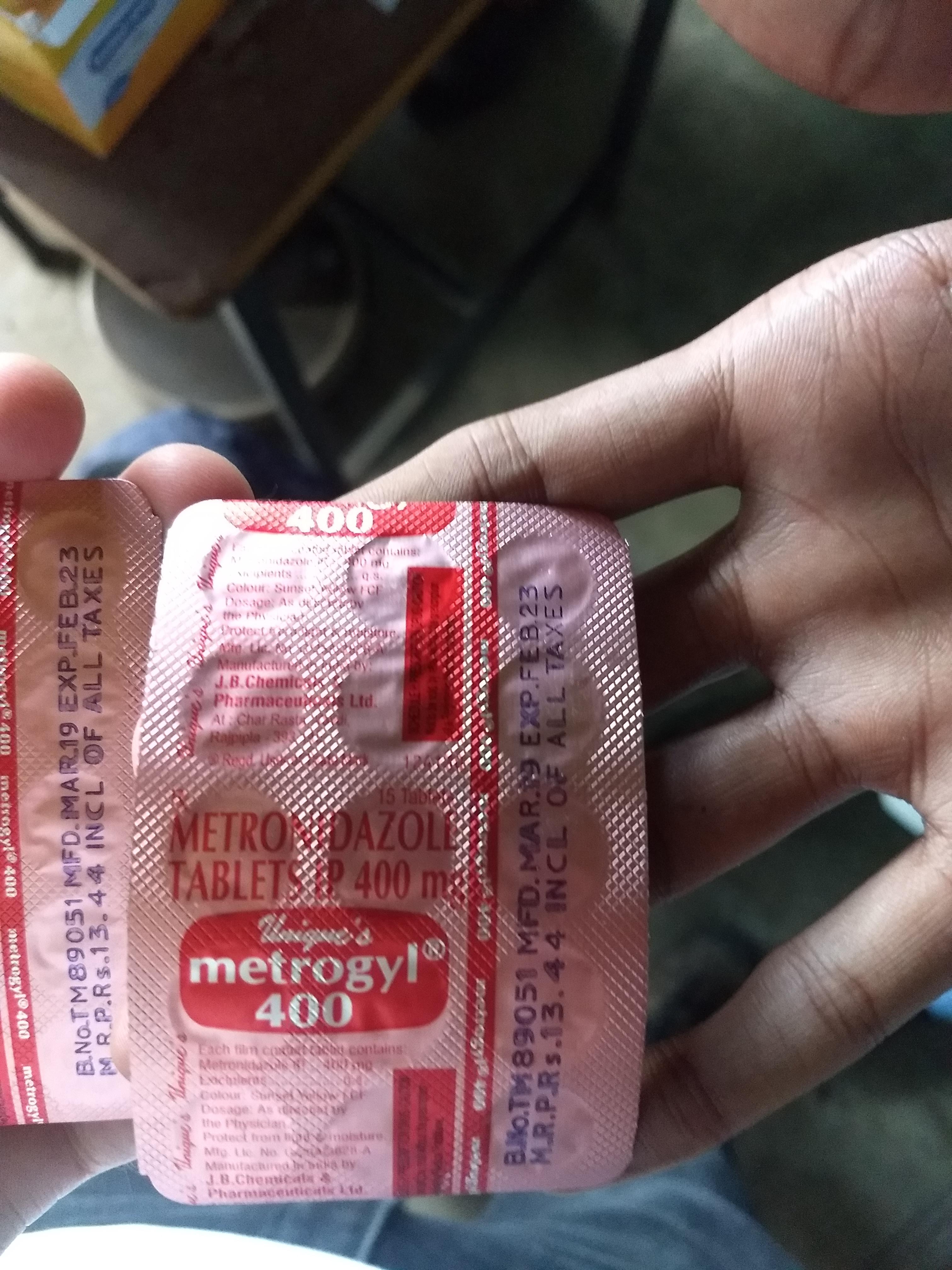

Supplement: Supplementary file 1 [file antibiotics-10-01433-s001.zip › Supplemrnrtary S2_ Site Photographs/Human antibiotic (metronidazole) used in livestock 2 (site 1).jpg]

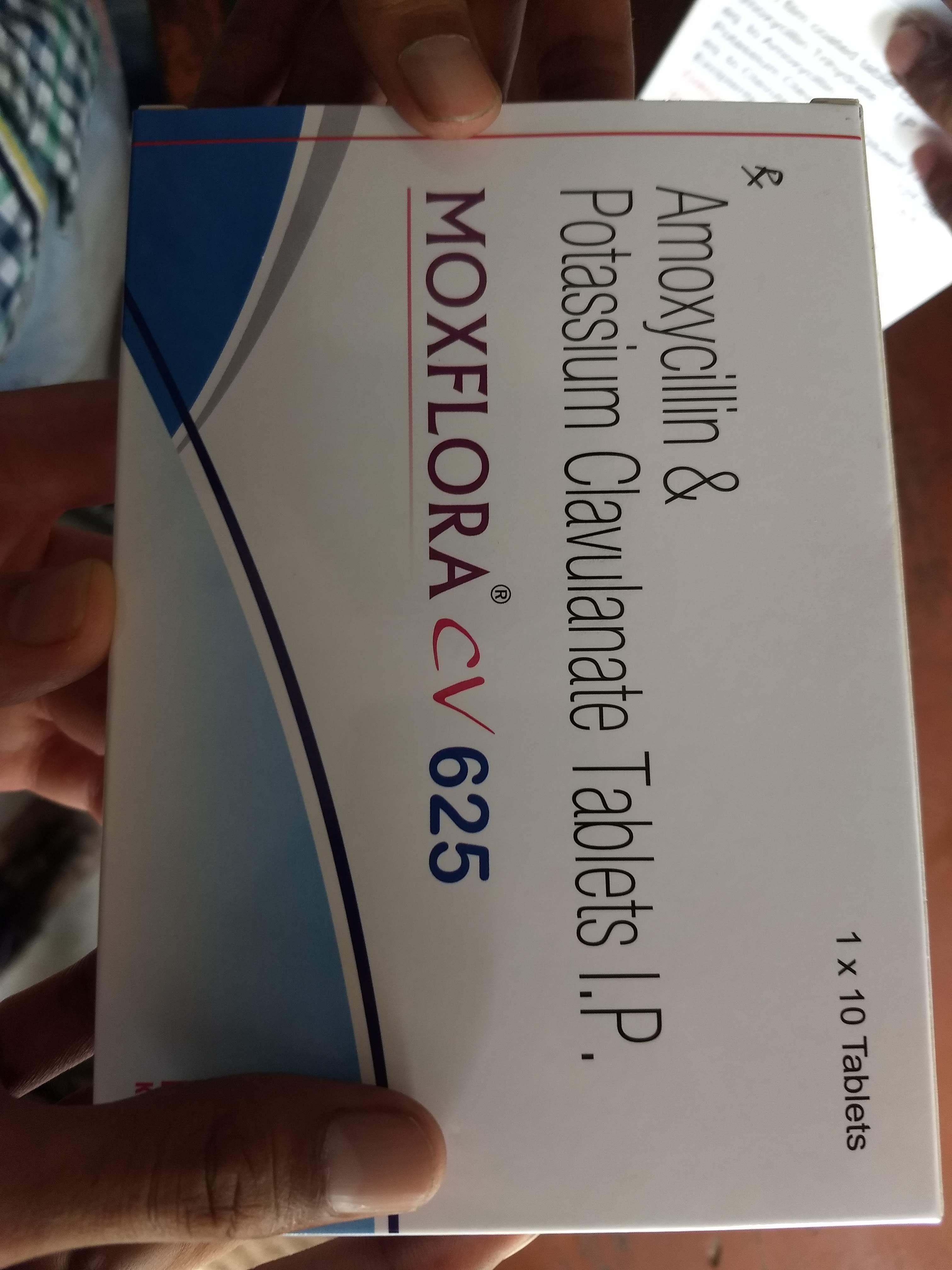

Supplement: Supplementary file 1 [file antibiotics-10-01433-s001.zip › Supplemrnrtary S2_ Site Photographs/Human antibiotic used in livestock -Informal Provider 1 (site 1).jpg]

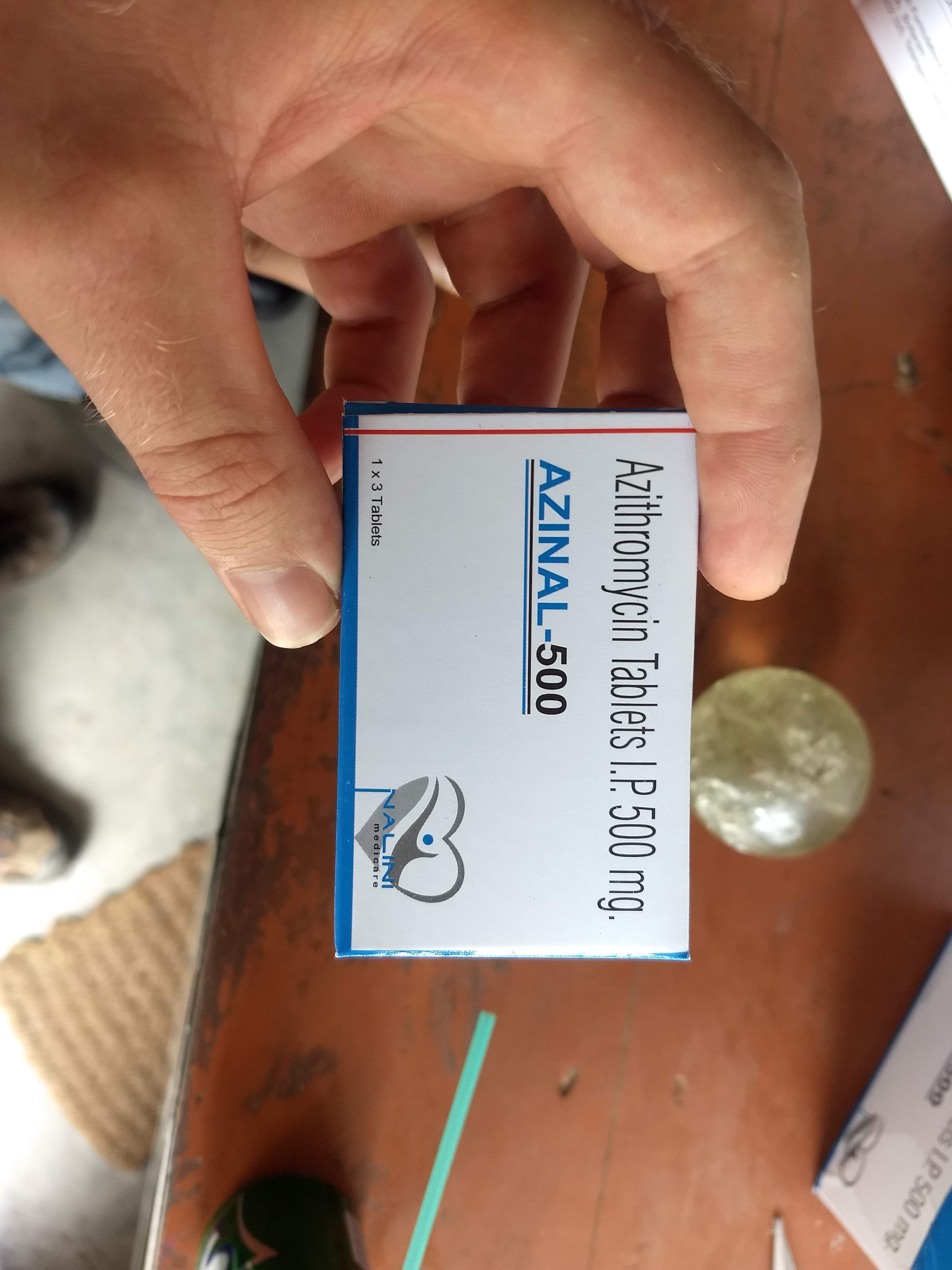

Supplement: Supplementary file 1 [file antibiotics-10-01433-s001.zip › Supplemrnrtary S2_ Site Photographs/Human antibiotic used in livestock- Informal Provider 3 (site 1).jpg]

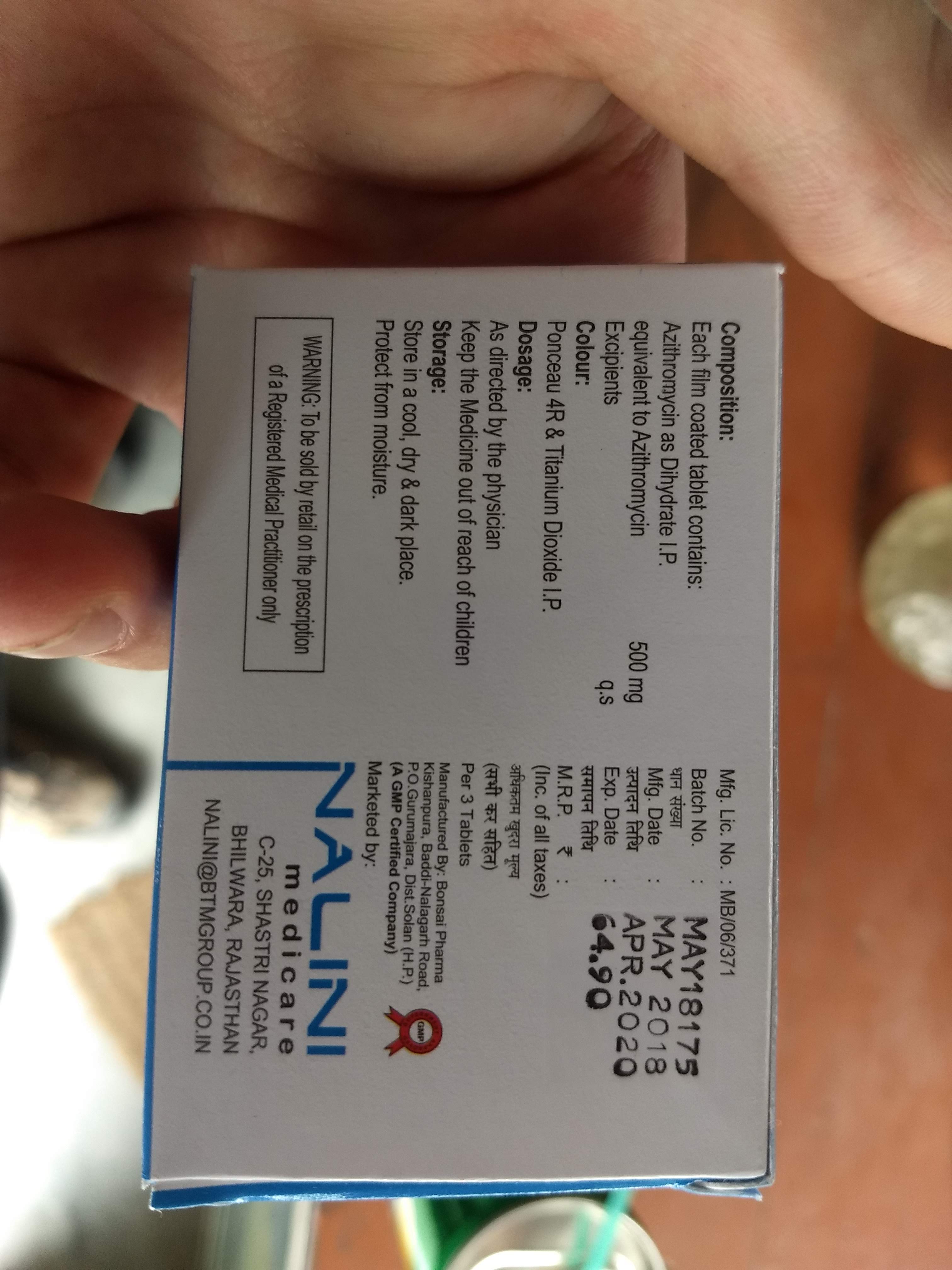

Supplement: Supplementary file 1 [file antibiotics-10-01433-s001.zip › Supplemrnrtary S2_ Site Photographs/Human antibiotic used in livestock- Informal Provider 4 (site 1).jpg]

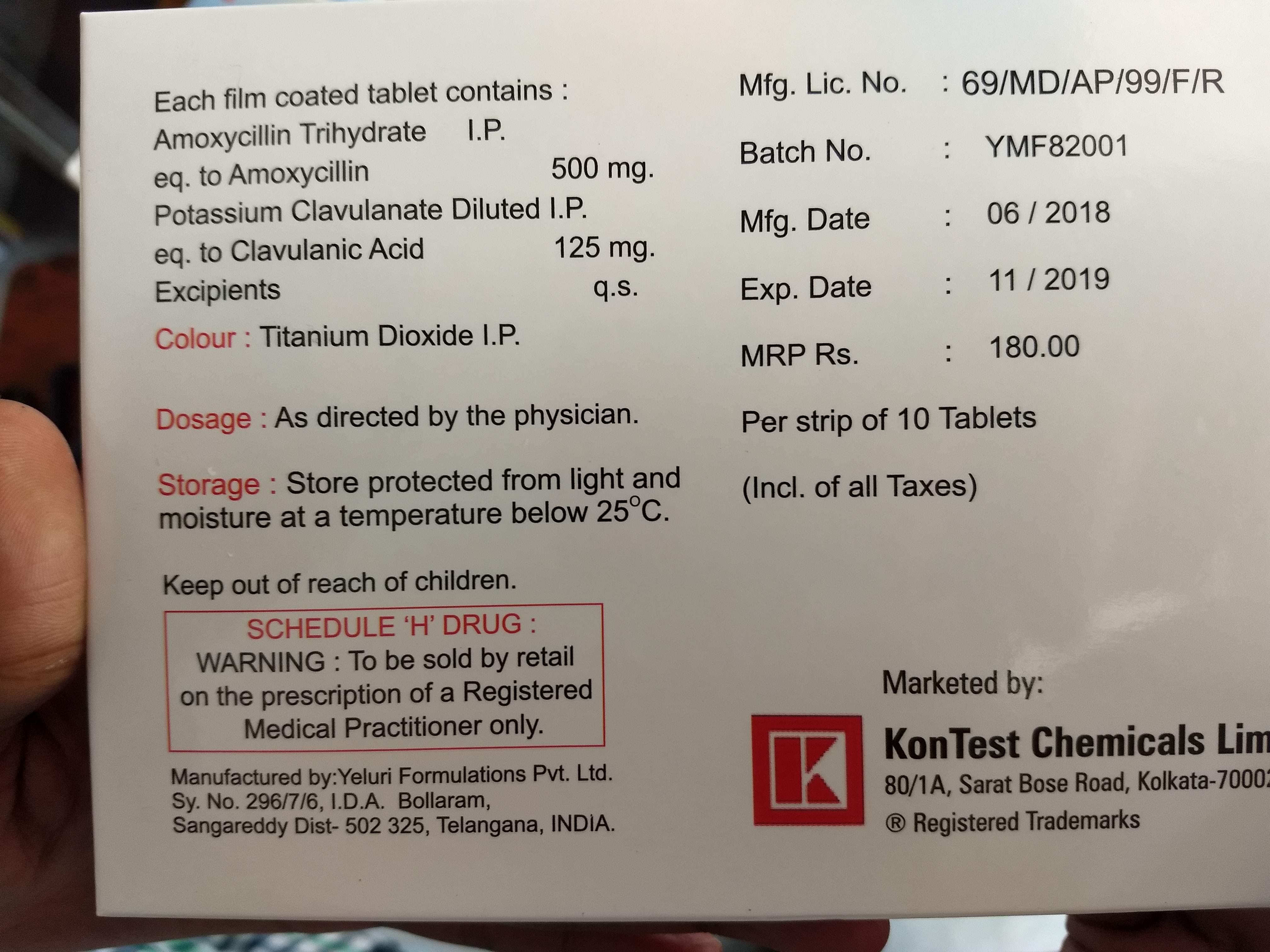

Supplement: Supplementary file 1 [file antibiotics-10-01433-s001.zip › Supplemrnrtary S2_ Site Photographs/Human antibiotic used in livestock-Informal provider 2 (site 1).jpg]

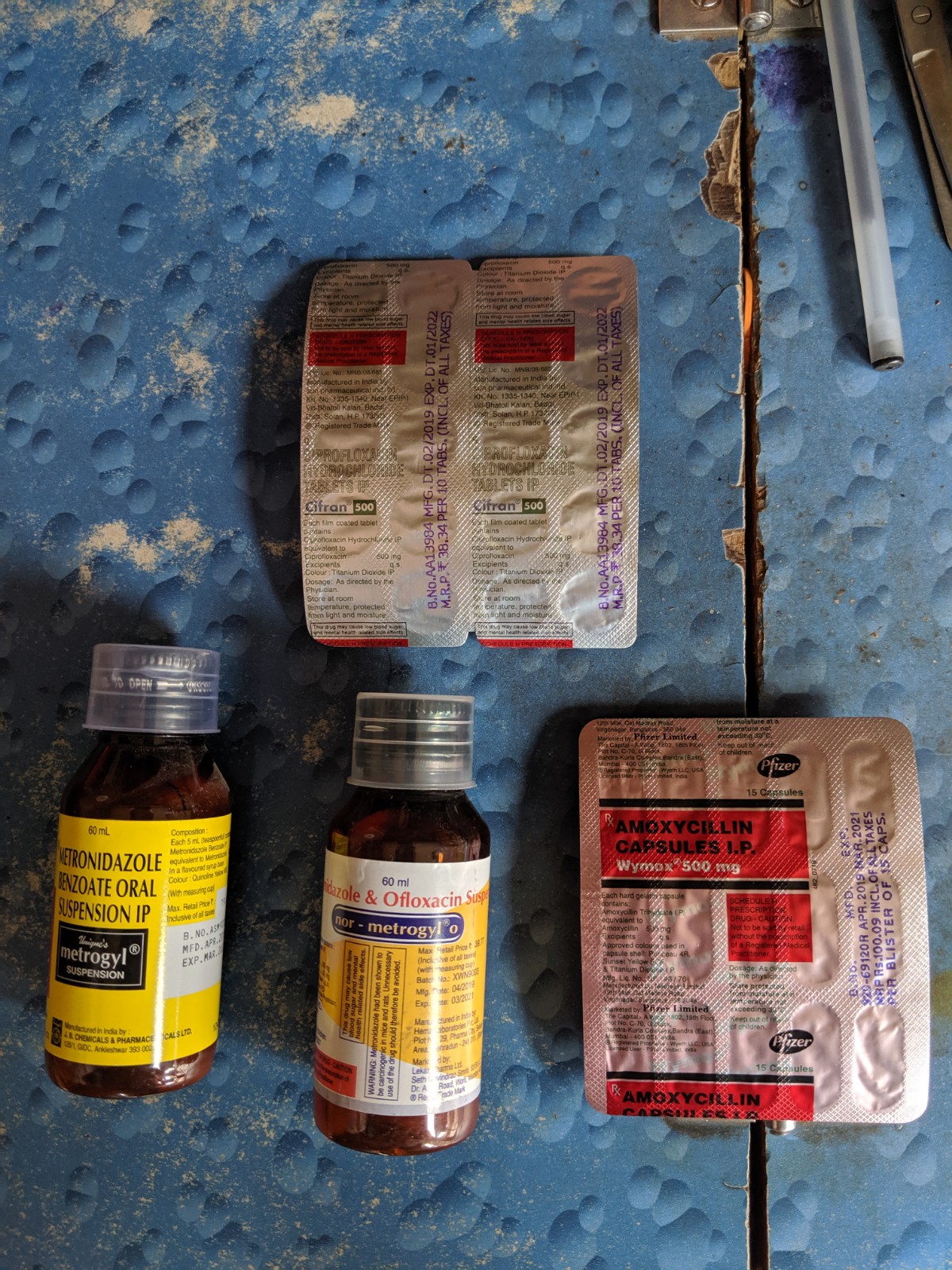

Supplement: Supplementary file 1 [file antibiotics-10-01433-s001.zip › Supplemrnrtary S2_ Site Photographs/Human antibiotics used in livestock human drug shop (site 1).jpg]

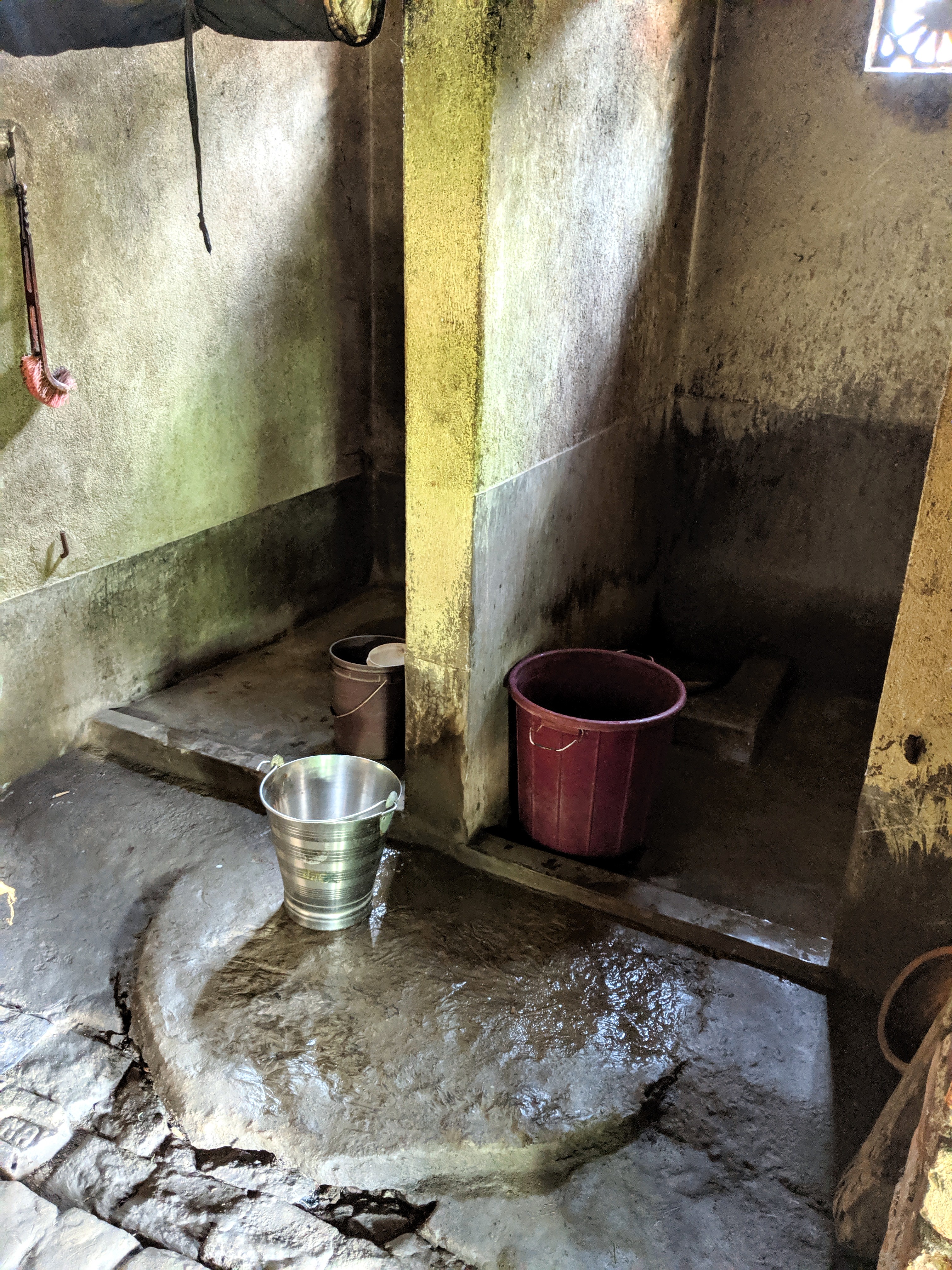

Supplement: Supplementary file 1 [file antibiotics-10-01433-s001.zip › Supplemrnrtary S2_ Site Photographs/Human waste facility in cattle shed (site 1).jpg]

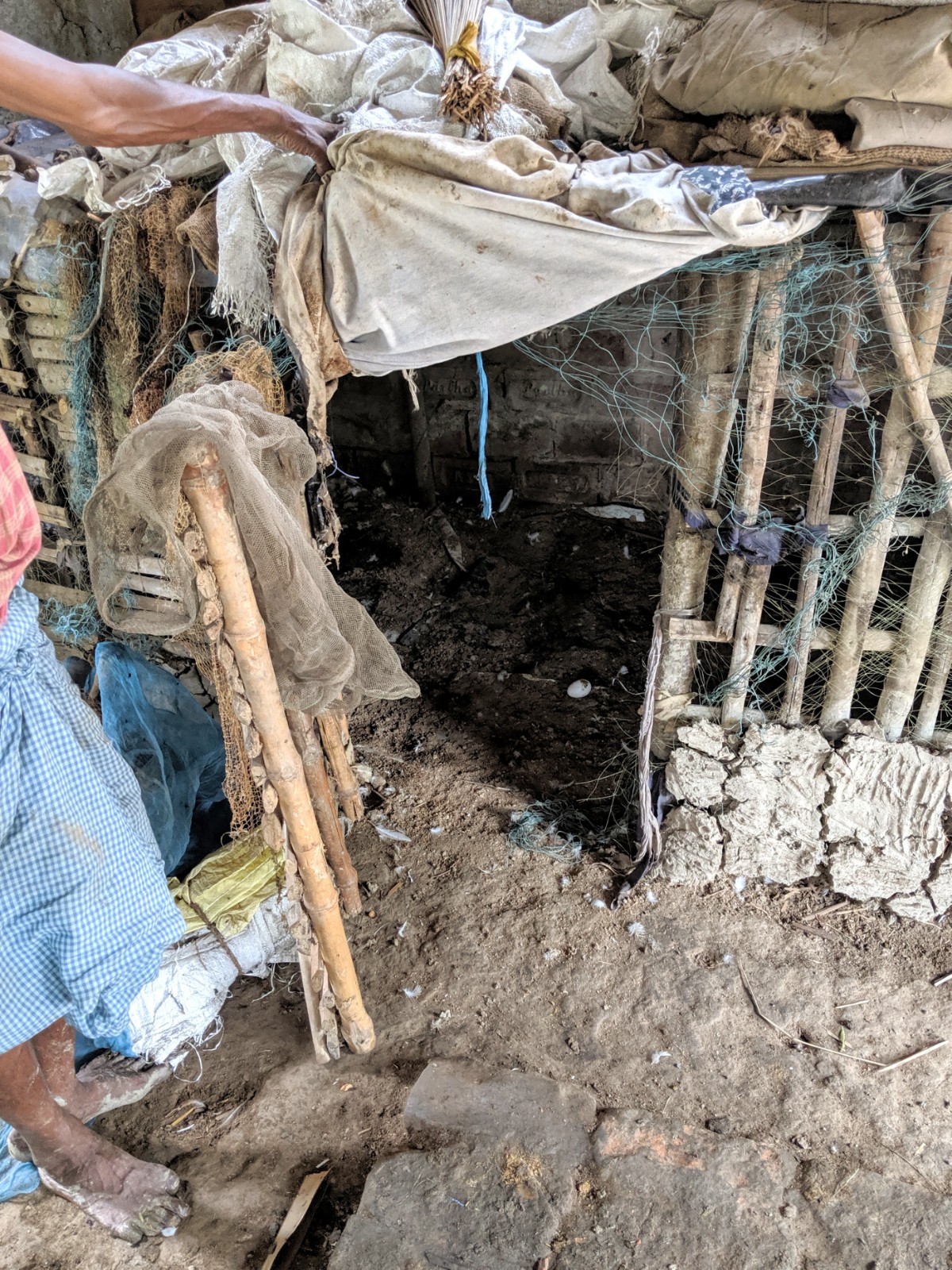

Supplement: Supplementary file 1 [file antibiotics-10-01433-s001.zip › Supplemrnrtary S2_ Site Photographs/Indoor poultry housing 1 (site 1).jpg]

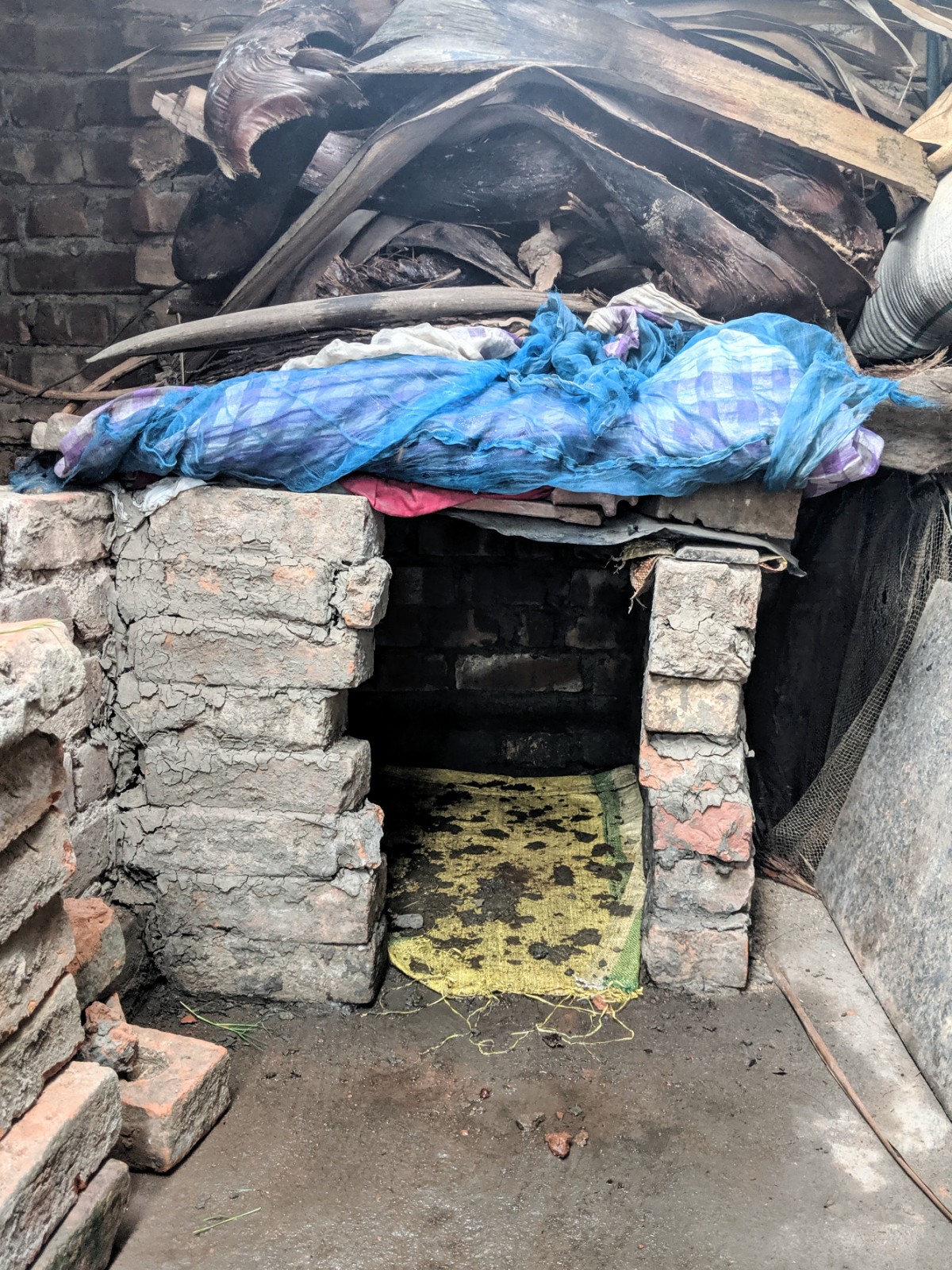

Supplement: Supplementary file 1 [file antibiotics-10-01433-s001.zip › Supplemrnrtary S2_ Site Photographs/Indoor poultry housing 2 (site 1).jpg]

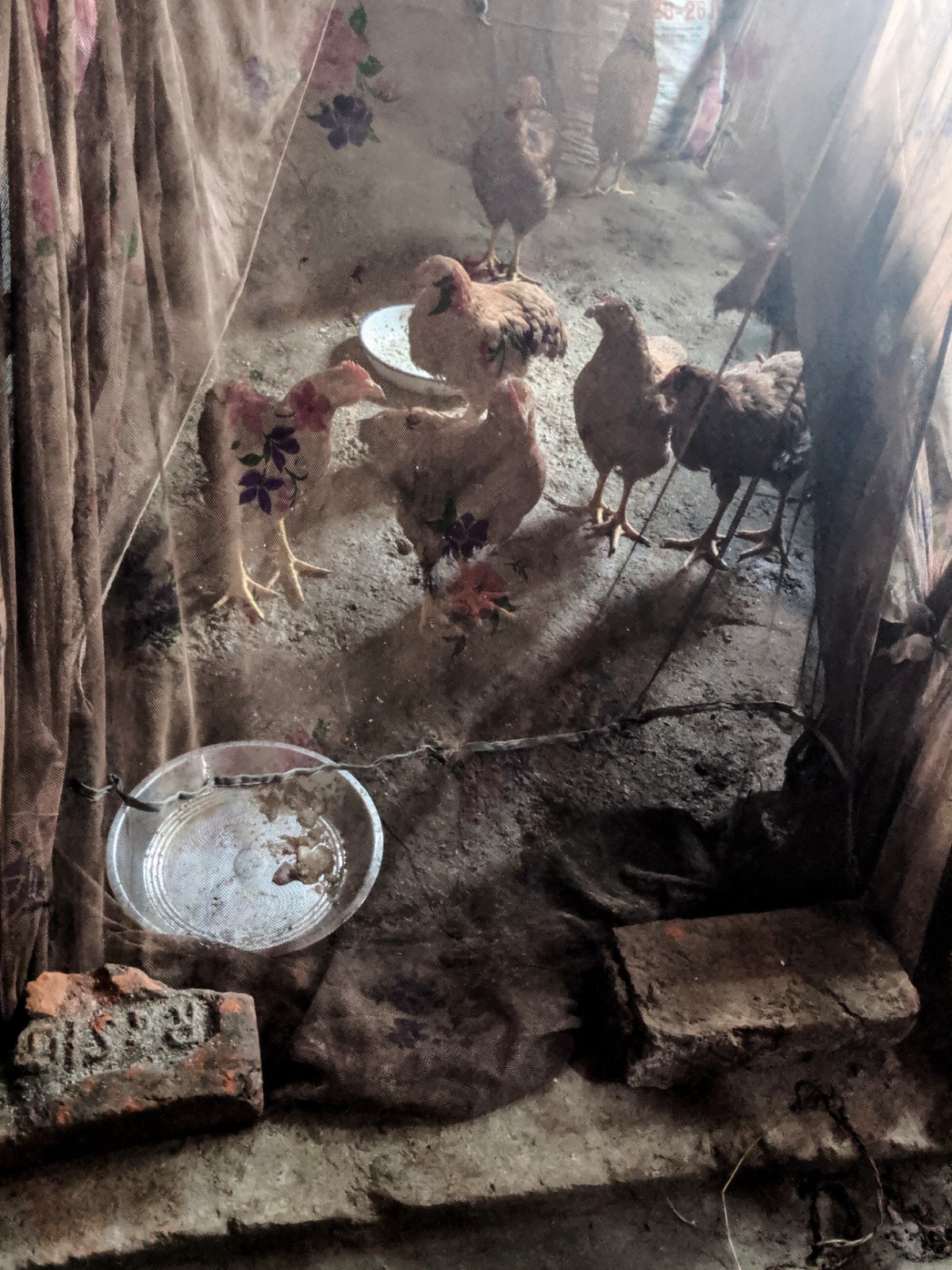

Supplement: Supplementary file 1 [file antibiotics-10-01433-s001.zip › Supplemrnrtary S2_ Site Photographs/Indoor poultry housing 3 (site 1).jpg]

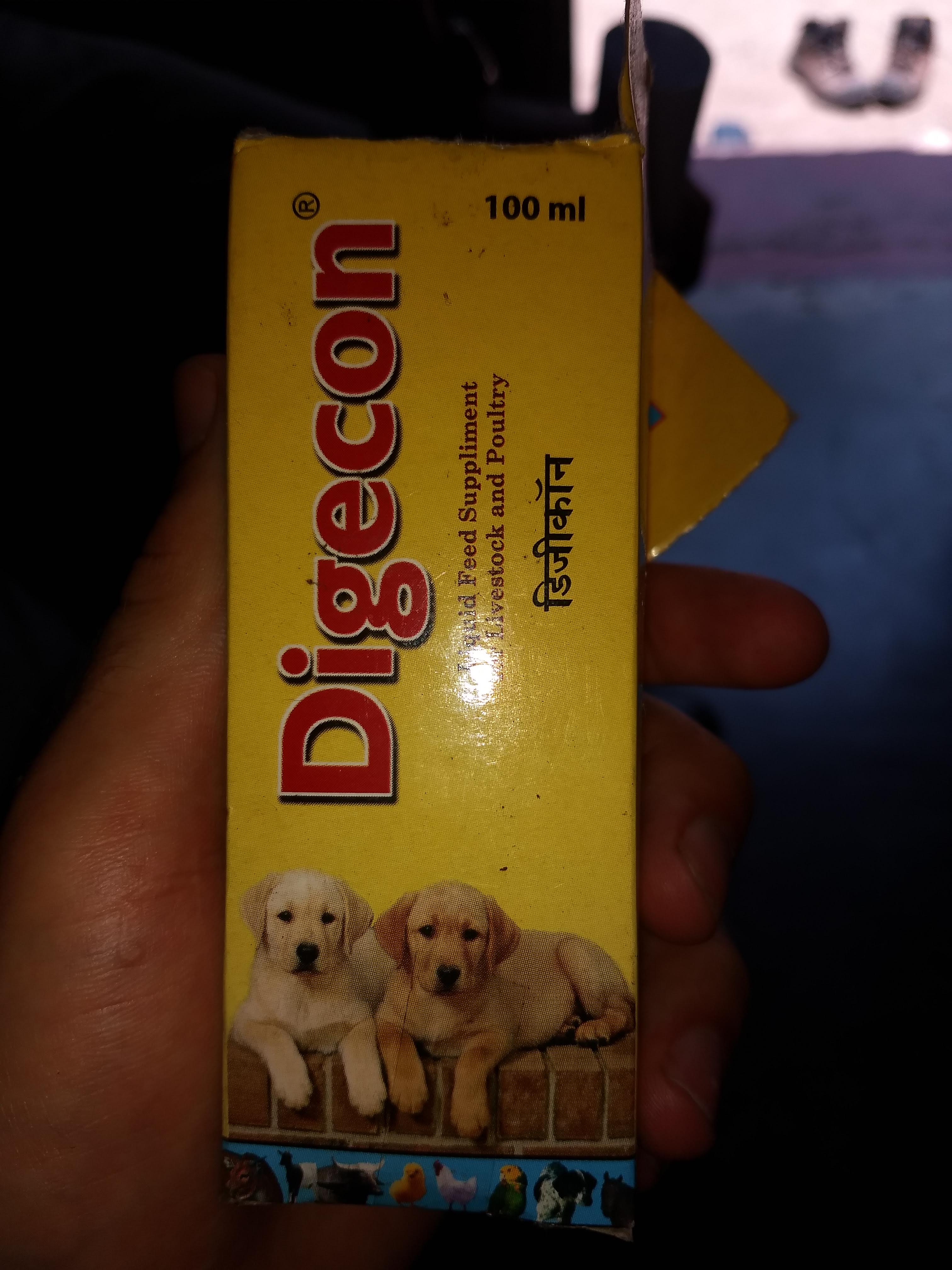

Supplement: Supplementary file 1 [file antibiotics-10-01433-s001.zip › Supplemrnrtary S2_ Site Photographs/Livestock feed supplement 1-household (site 1).jpg]

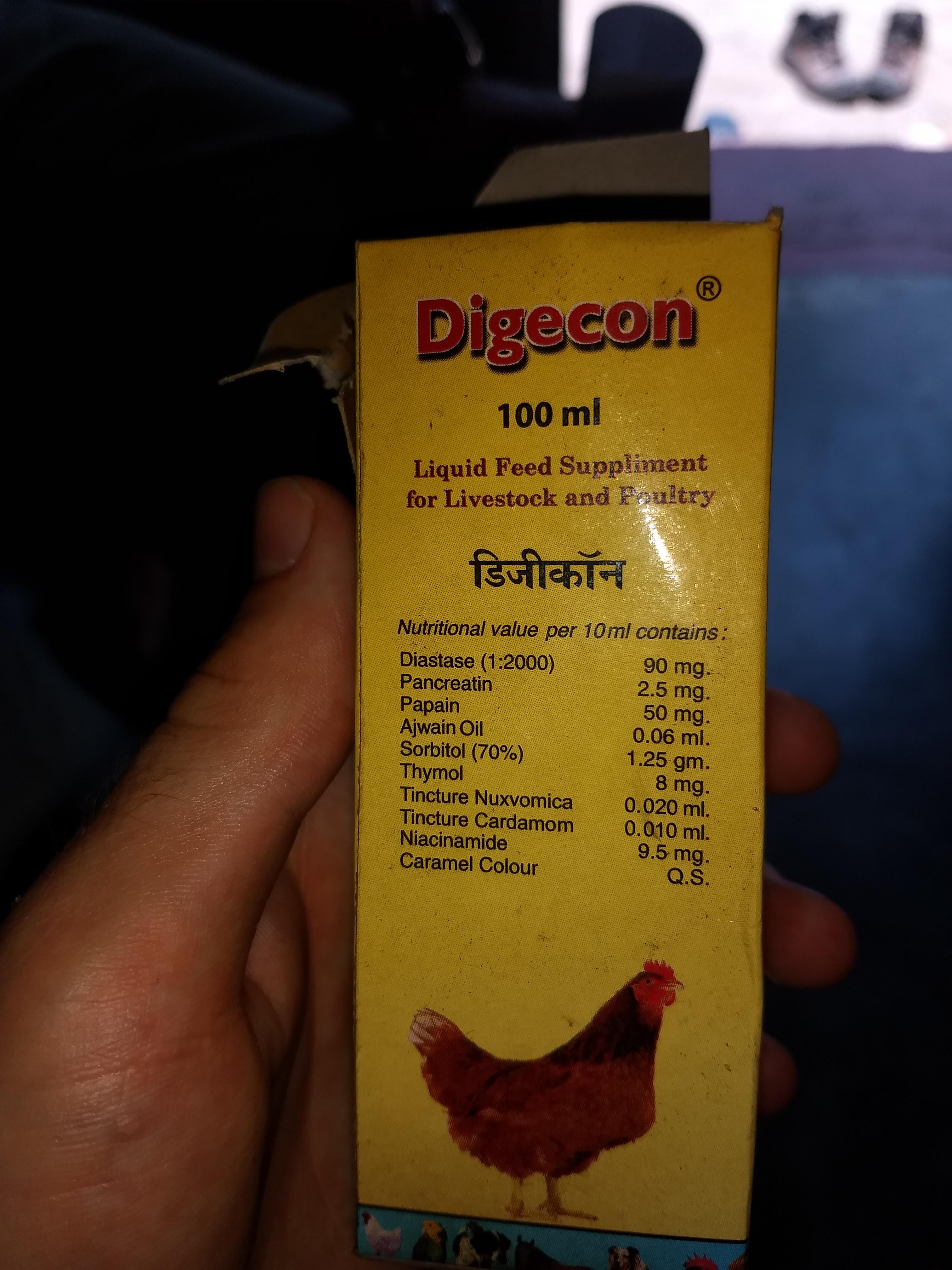

Supplement: Supplementary file 1 [file antibiotics-10-01433-s001.zip › Supplemrnrtary S2_ Site Photographs/Livestock feed supplement 2- household (site 1).jpg]

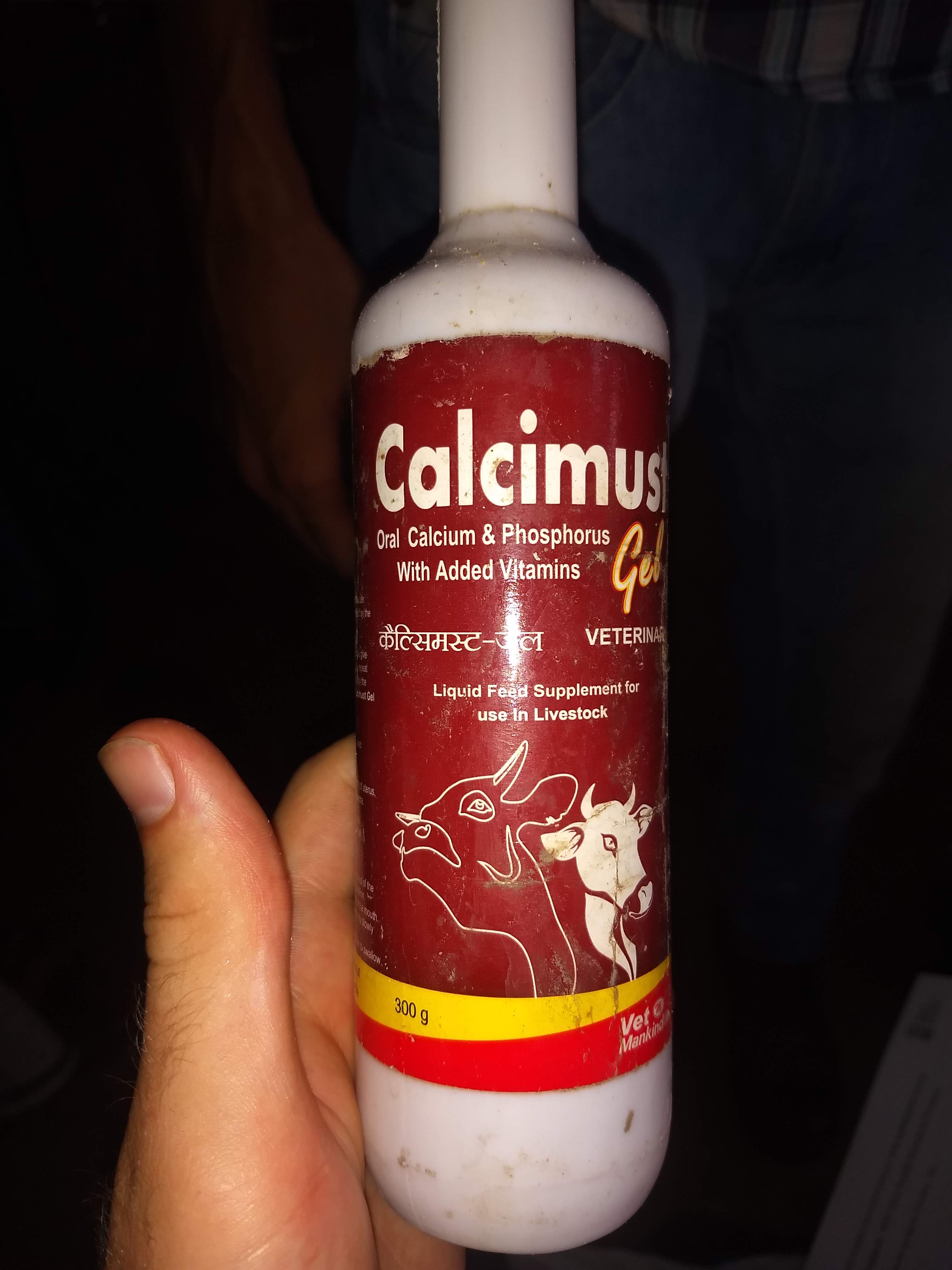

Supplement: Supplementary file 1 [file antibiotics-10-01433-s001.zip › Supplemrnrtary S2_ Site Photographs/Livestock feed supplement 3- household (site 1).jpg]

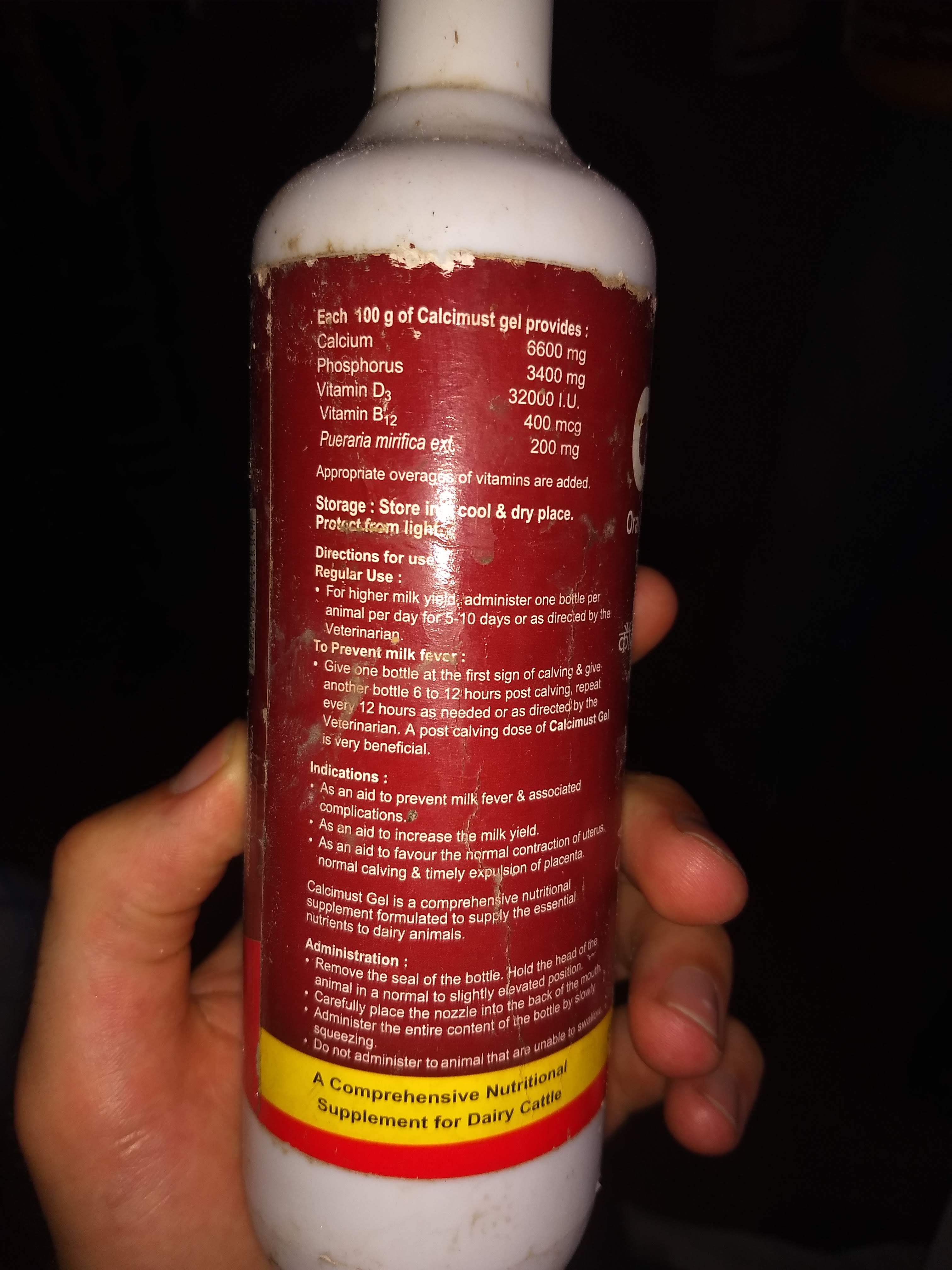

Supplement: Supplementary file 1 [file antibiotics-10-01433-s001.zip › Supplemrnrtary S2_ Site Photographs/Livestock feed supplement 4- household (site 1).jpg]

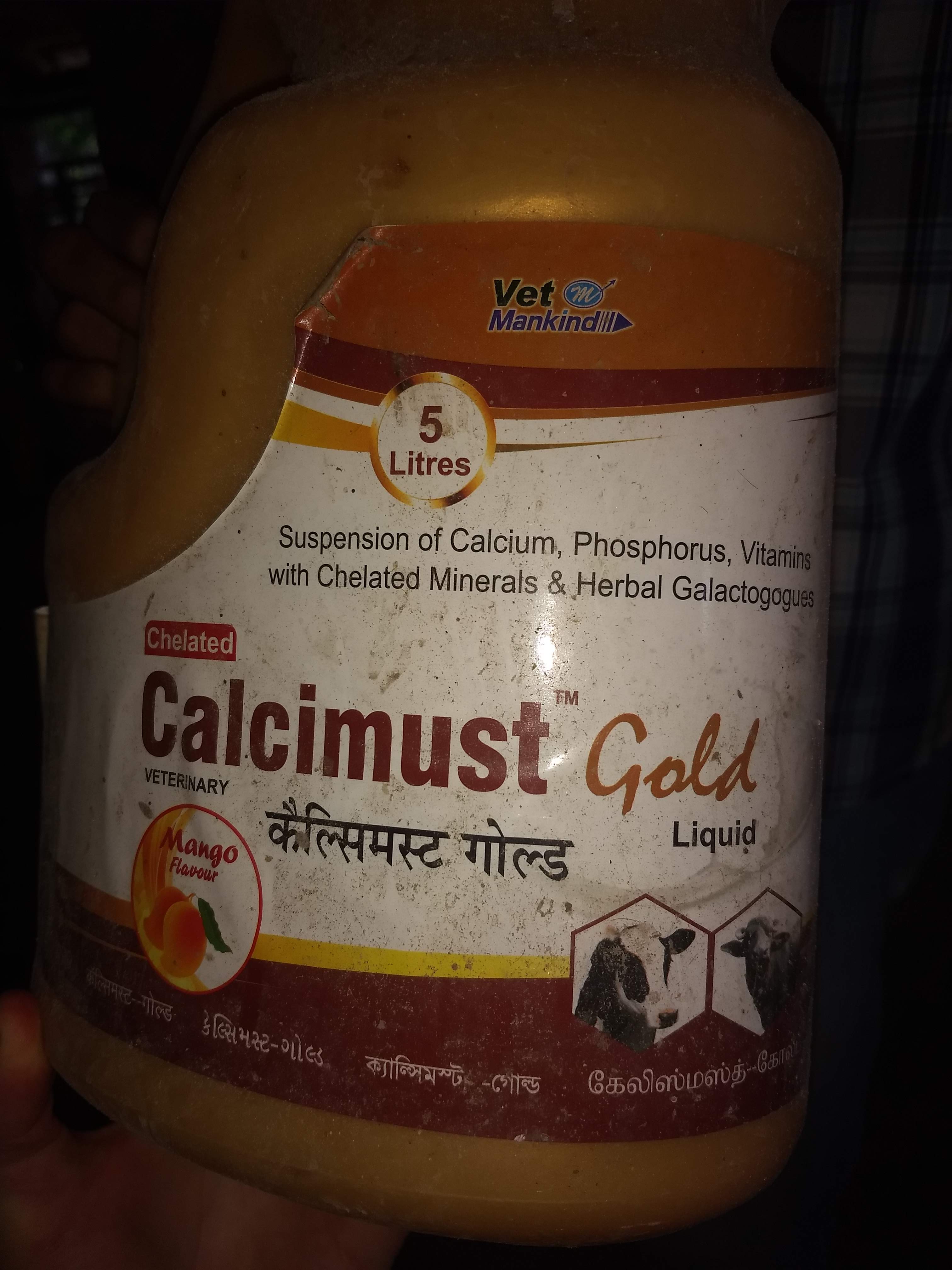

Supplement: Supplementary file 1 [file antibiotics-10-01433-s001.zip › Supplemrnrtary S2_ Site Photographs/Livestock feed supplement 5-household (site 1).jpg]

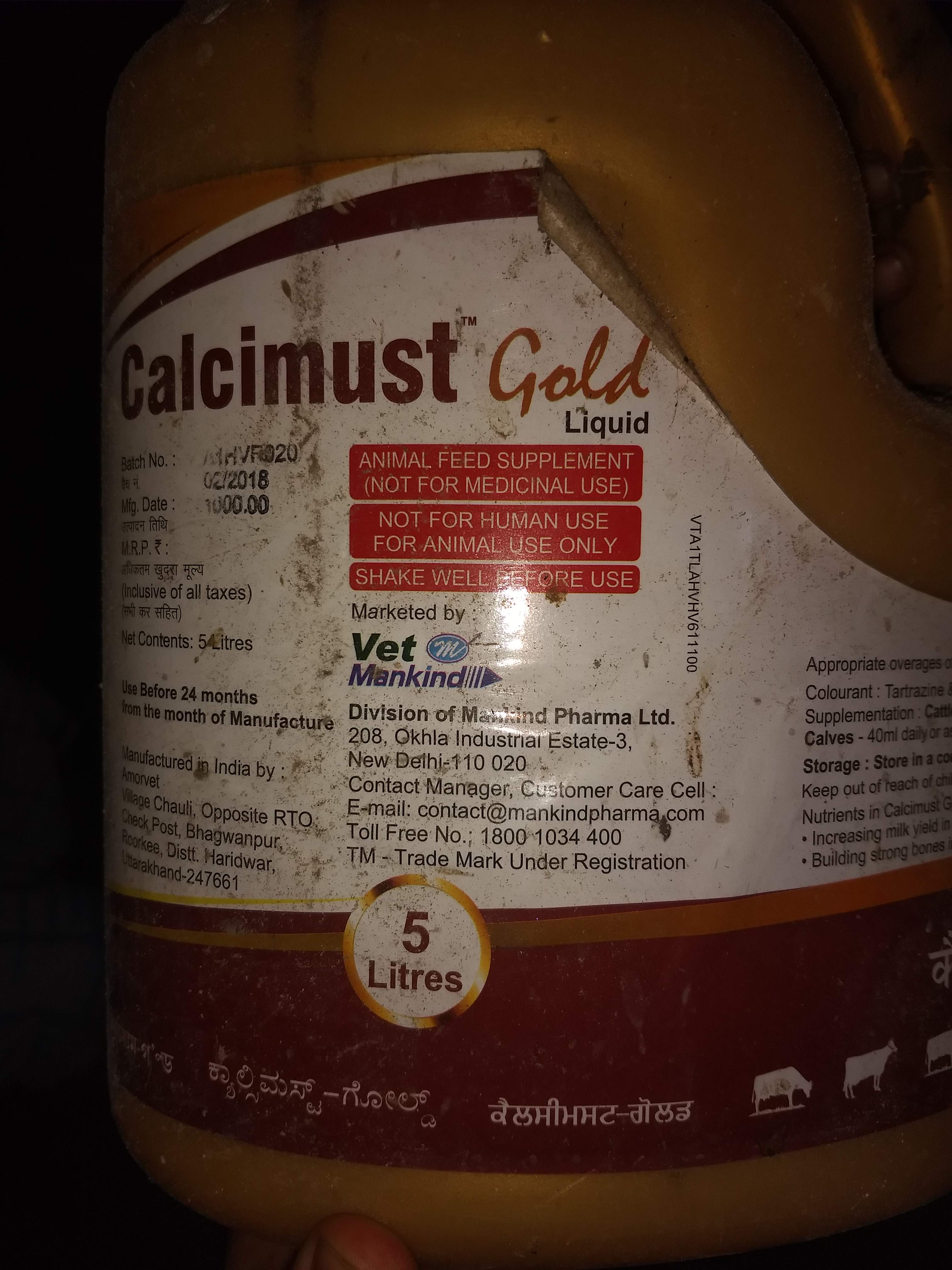

Supplement: Supplementary file 1 [file antibiotics-10-01433-s001.zip › Supplemrnrtary S2_ Site Photographs/Livestock feed supplement 6-household (site 1).jpg]

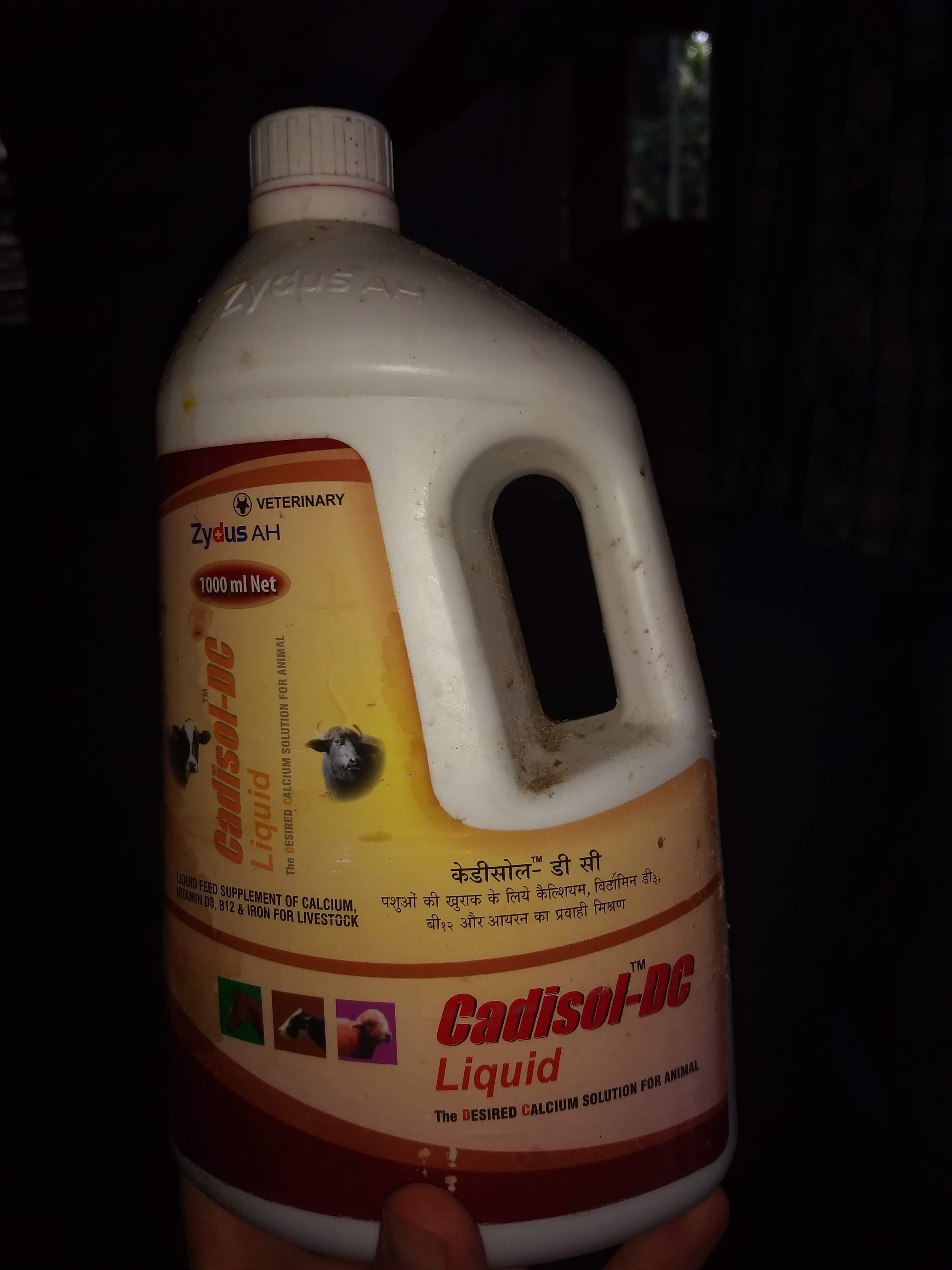

Supplement: Supplementary file 1 [file antibiotics-10-01433-s001.zip › Supplemrnrtary S2_ Site Photographs/Livestock feed supplement 7-household (site 1).jpg]

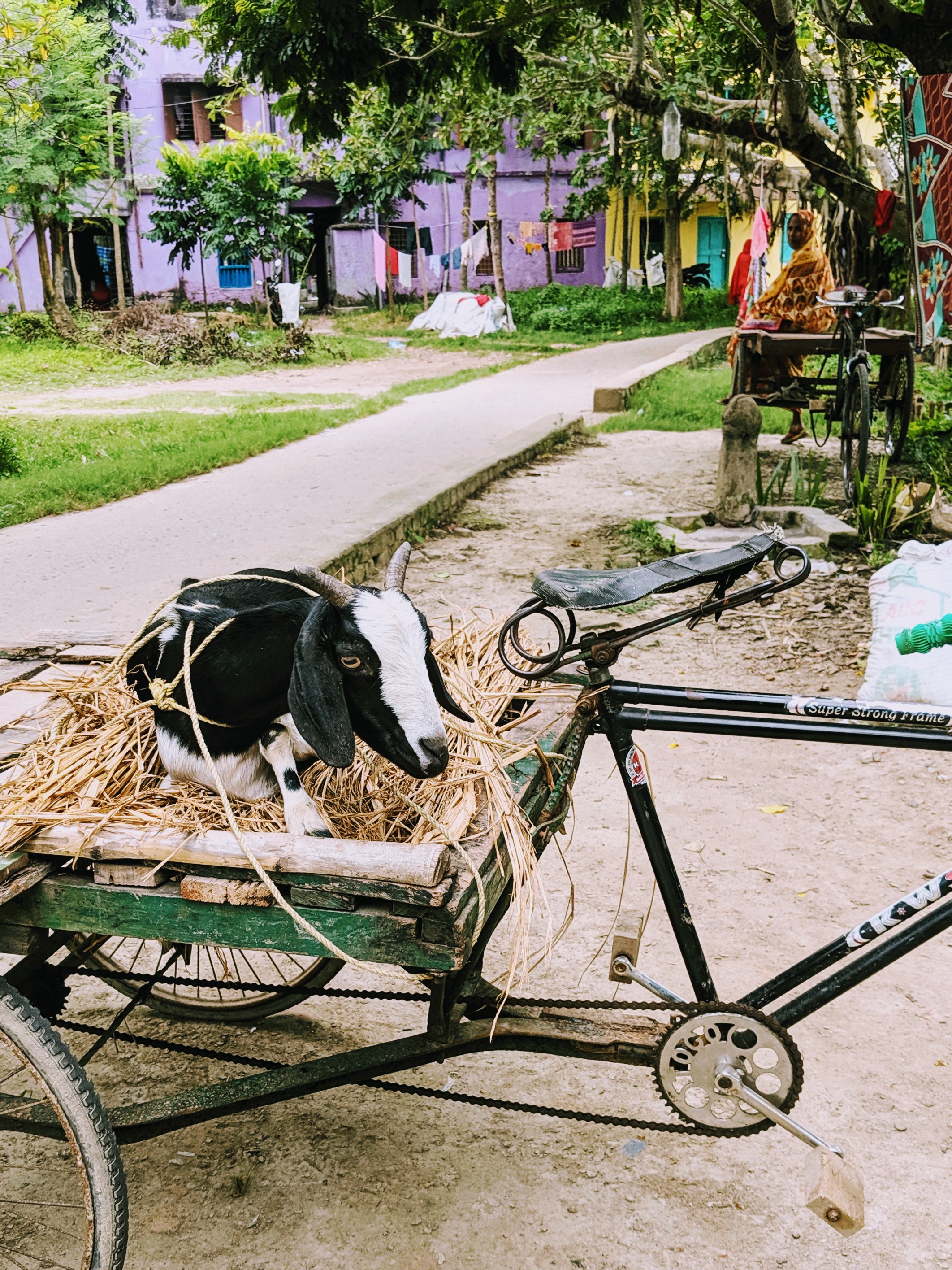

Supplement: Supplementary file 1 [file antibiotics-10-01433-s001.zip › Supplemrnrtary S2_ Site Photographs/Livestock transport (site 1).jpg]

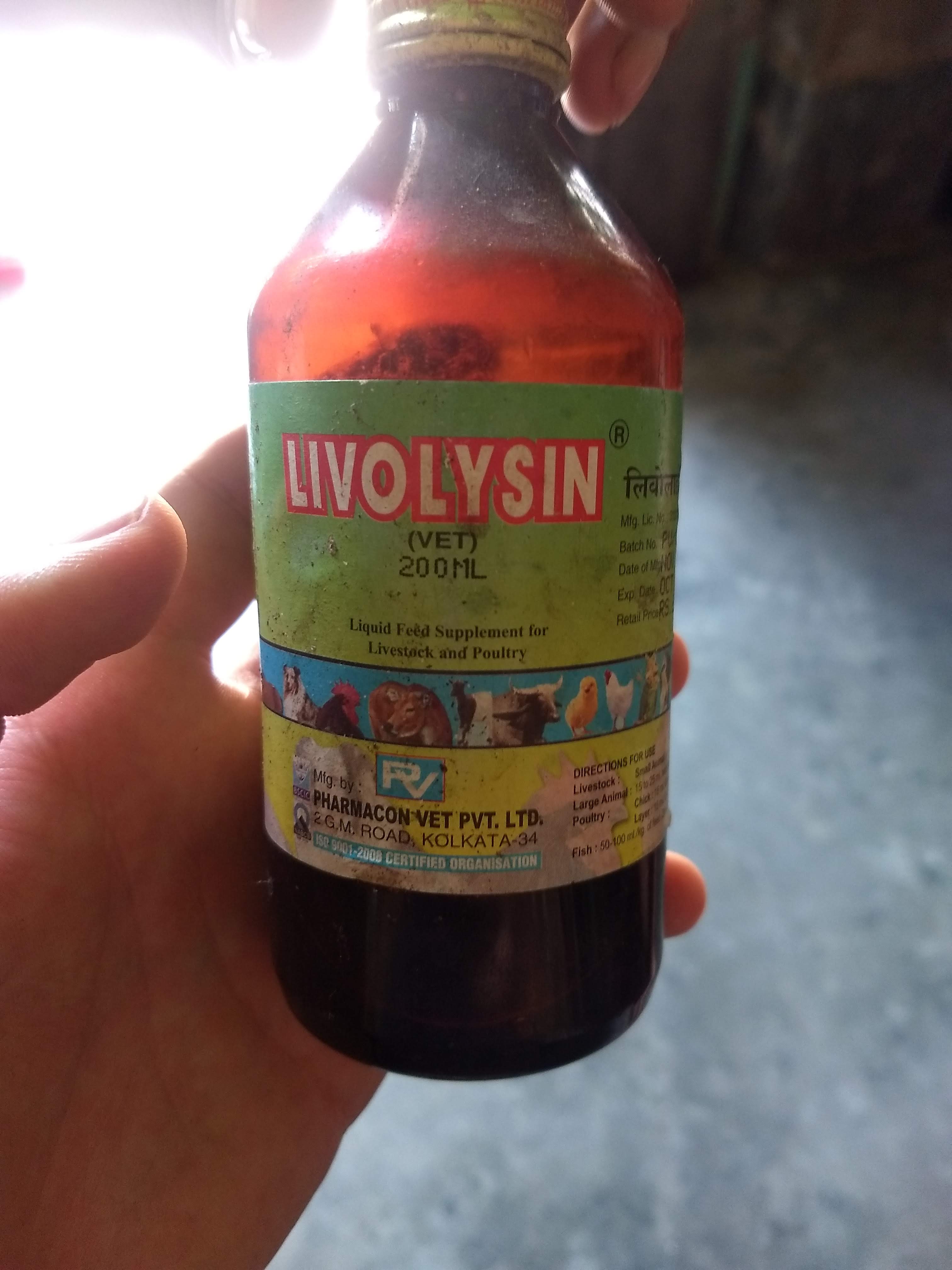

Supplement: Supplementary file 1 [file antibiotics-10-01433-s001.zip › Supplemrnrtary S2_ Site Photographs/Livestock vitamin supplement 1- household (site 1).jpg]

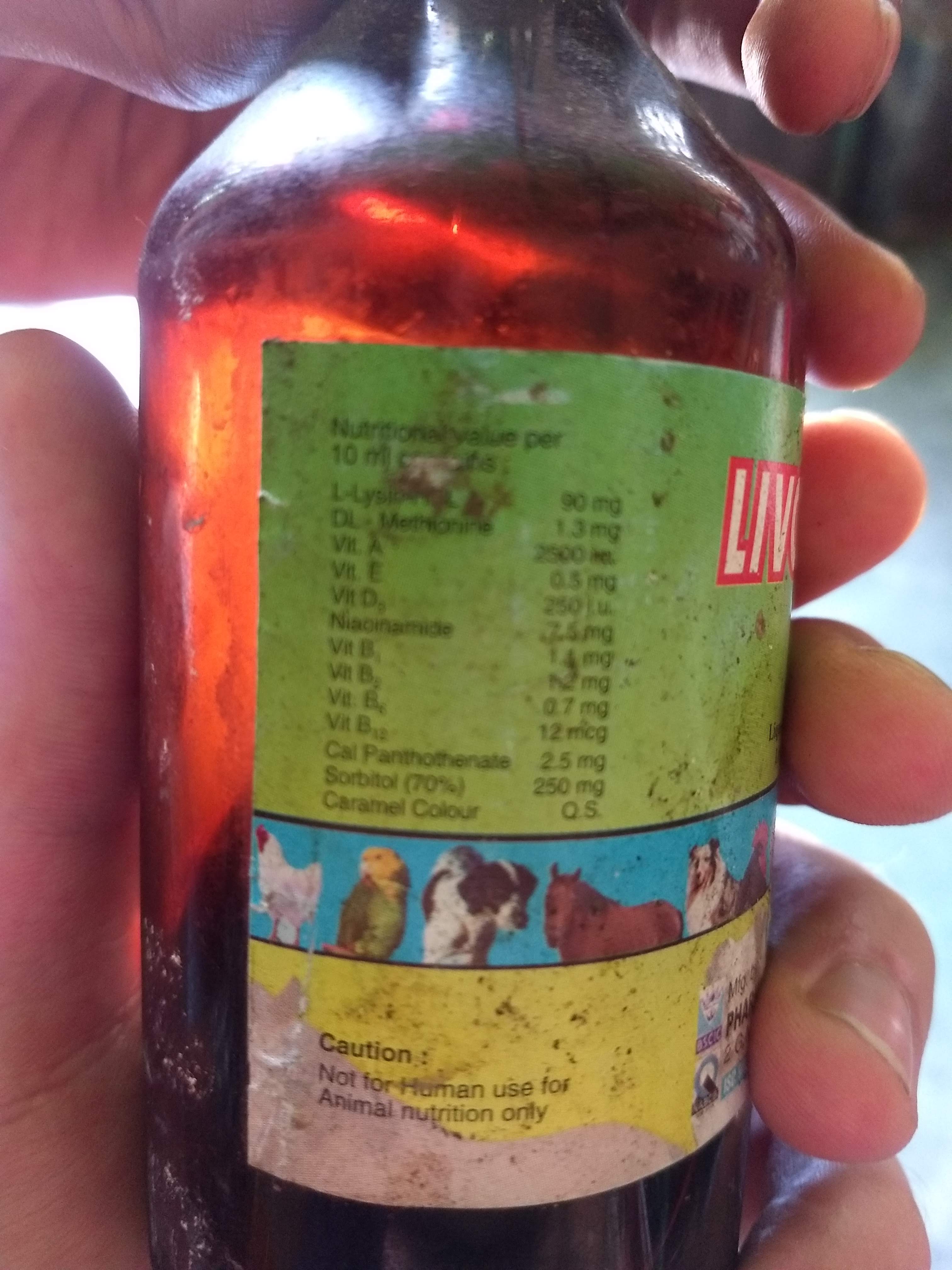

Supplement: Supplementary file 1 [file antibiotics-10-01433-s001.zip › Supplemrnrtary S2_ Site Photographs/Livestock vitamin supplement 2- household (site 1).jpg]

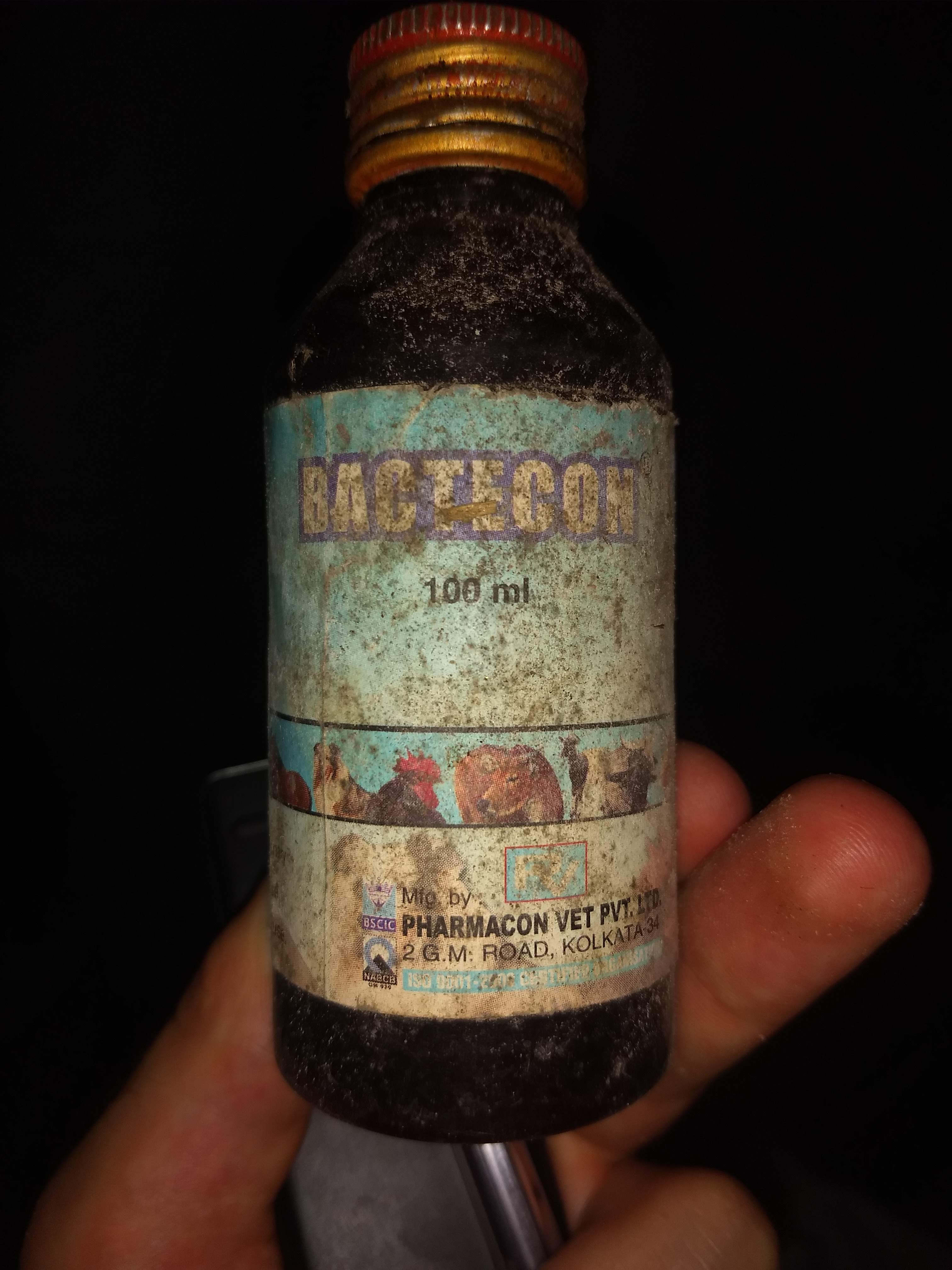

Supplement: Supplementary file 1 [file antibiotics-10-01433-s001.zip › Supplemrnrtary S2_ Site Photographs/Non-antibiotic wound medicine 1-household (site 1).jpg]

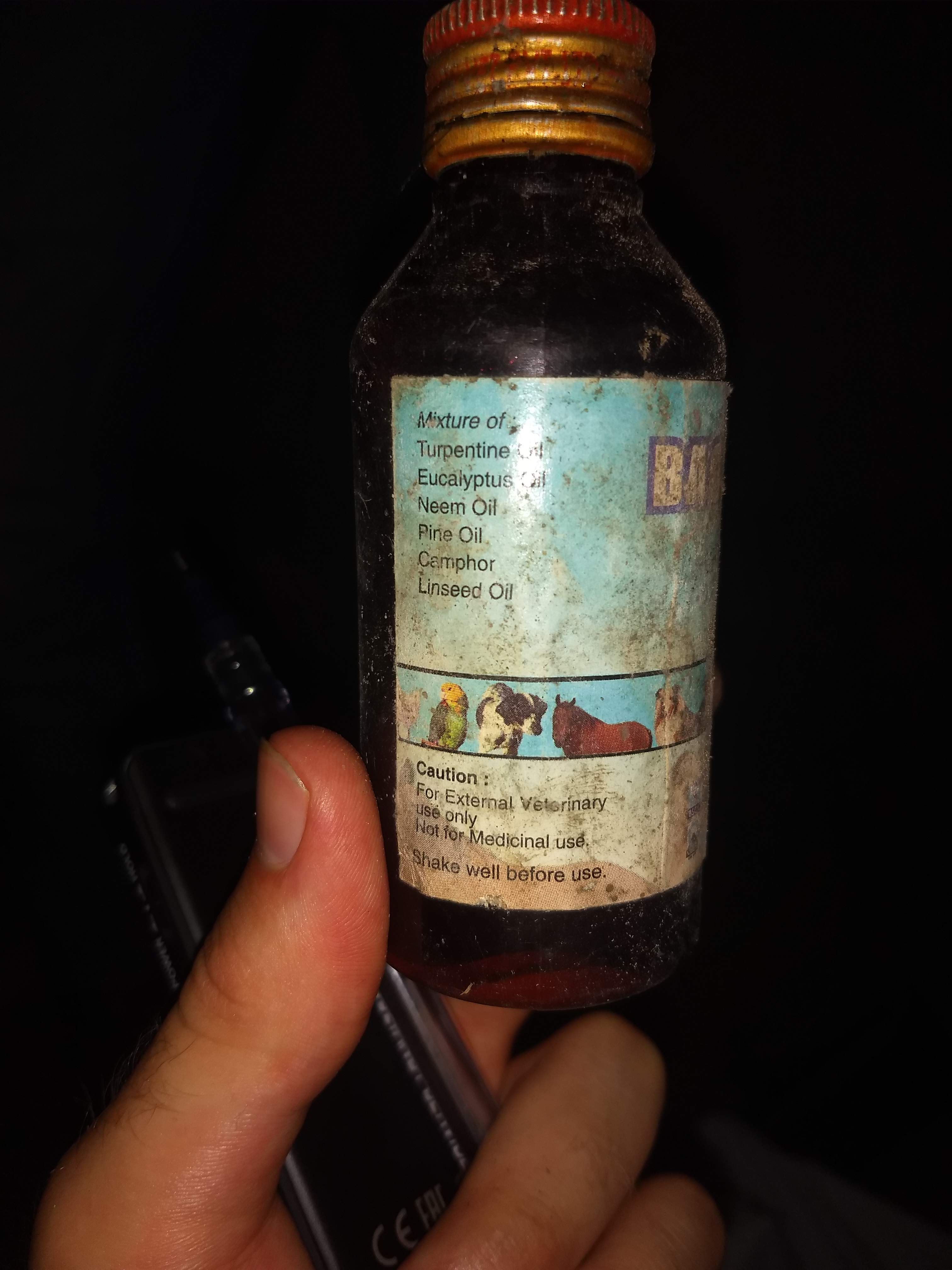

Supplement: Supplementary file 1 [file antibiotics-10-01433-s001.zip › Supplemrnrtary S2_ Site Photographs/Non-antibiotic wound medicine 2-household (site 1).jpg]

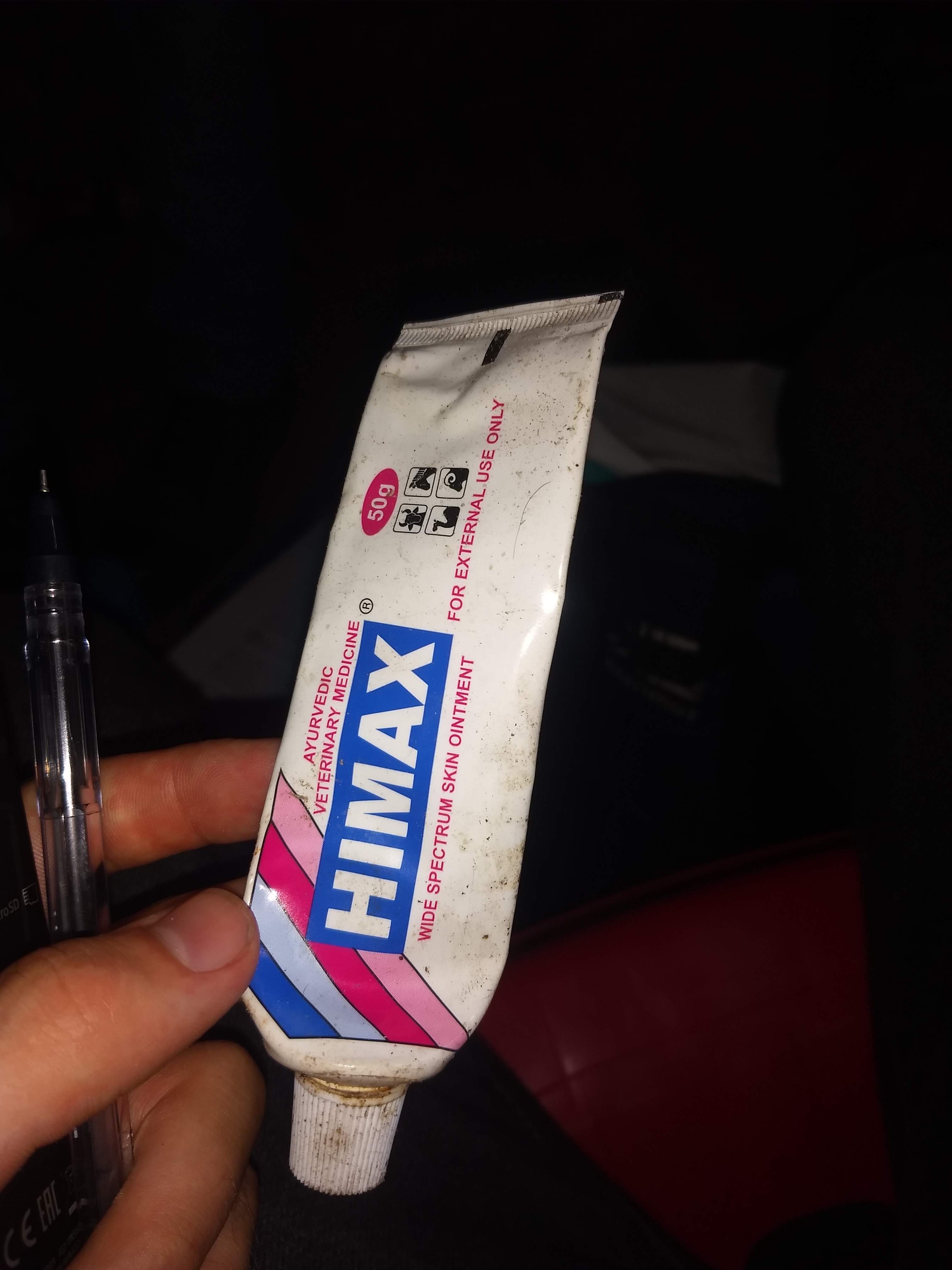

Supplement: Supplementary file 1 [file antibiotics-10-01433-s001.zip › Supplemrnrtary S2_ Site Photographs/Non-antibiotic wound medicine 3-household (site 1).jpg]

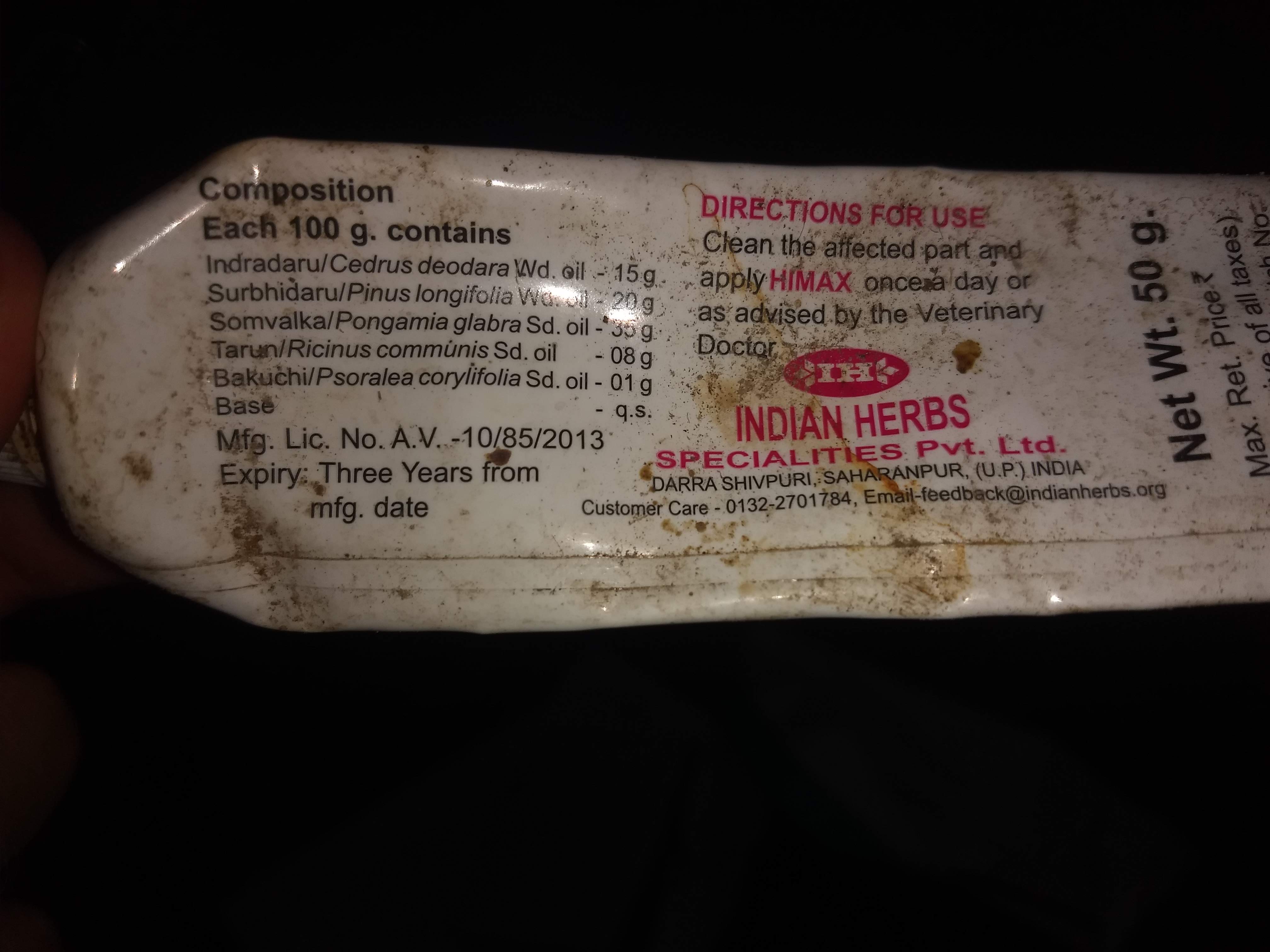

Supplement: Supplementary file 1 [file antibiotics-10-01433-s001.zip › Supplemrnrtary S2_ Site Photographs/Non-antibiotic wound medicine 4-household (site 1).jpg]

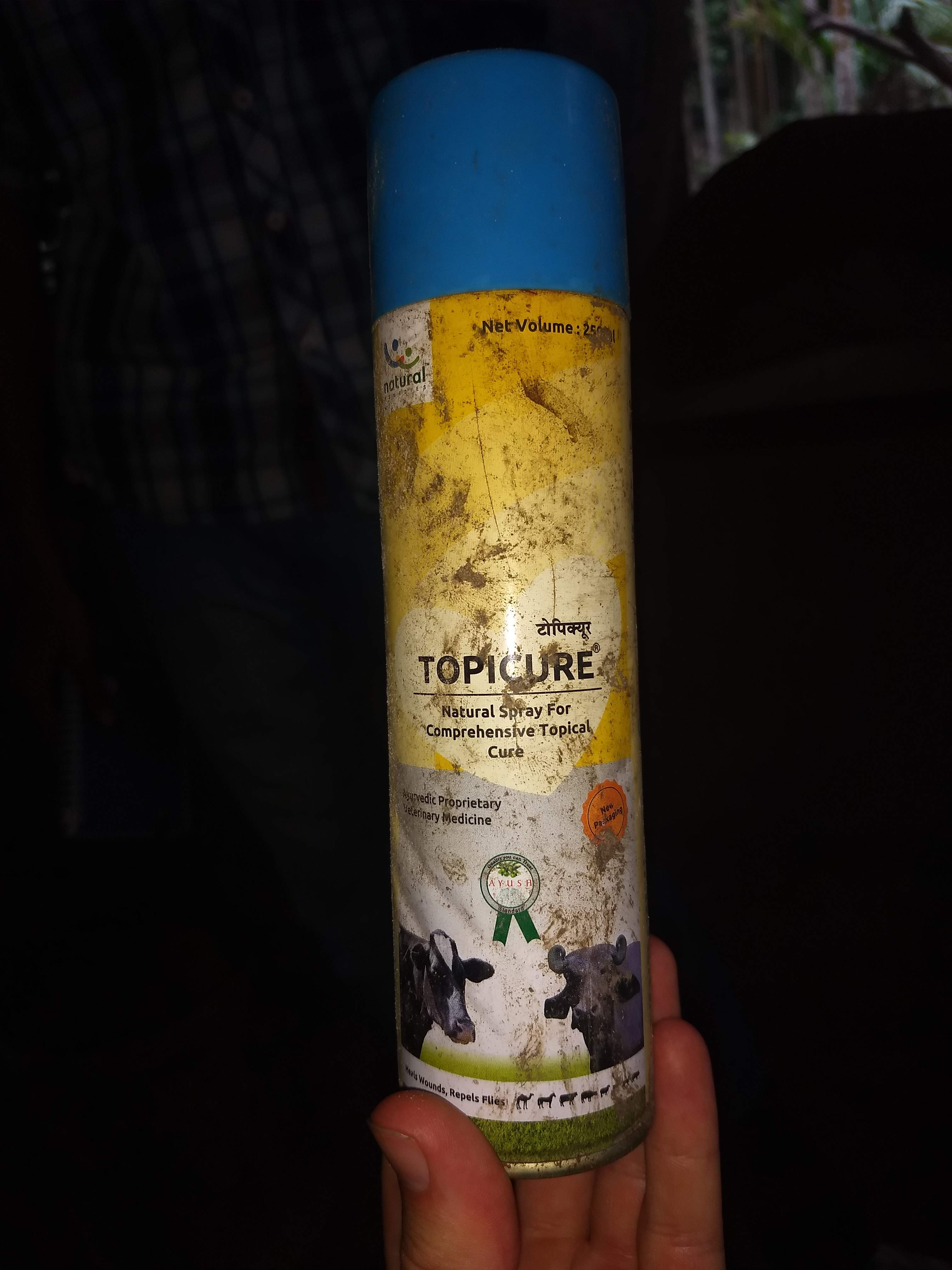

Supplement: Supplementary file 1 [file antibiotics-10-01433-s001.zip › Supplemrnrtary S2_ Site Photographs/Non-antibiotic wound medicine 5-household (site 1).jpg]

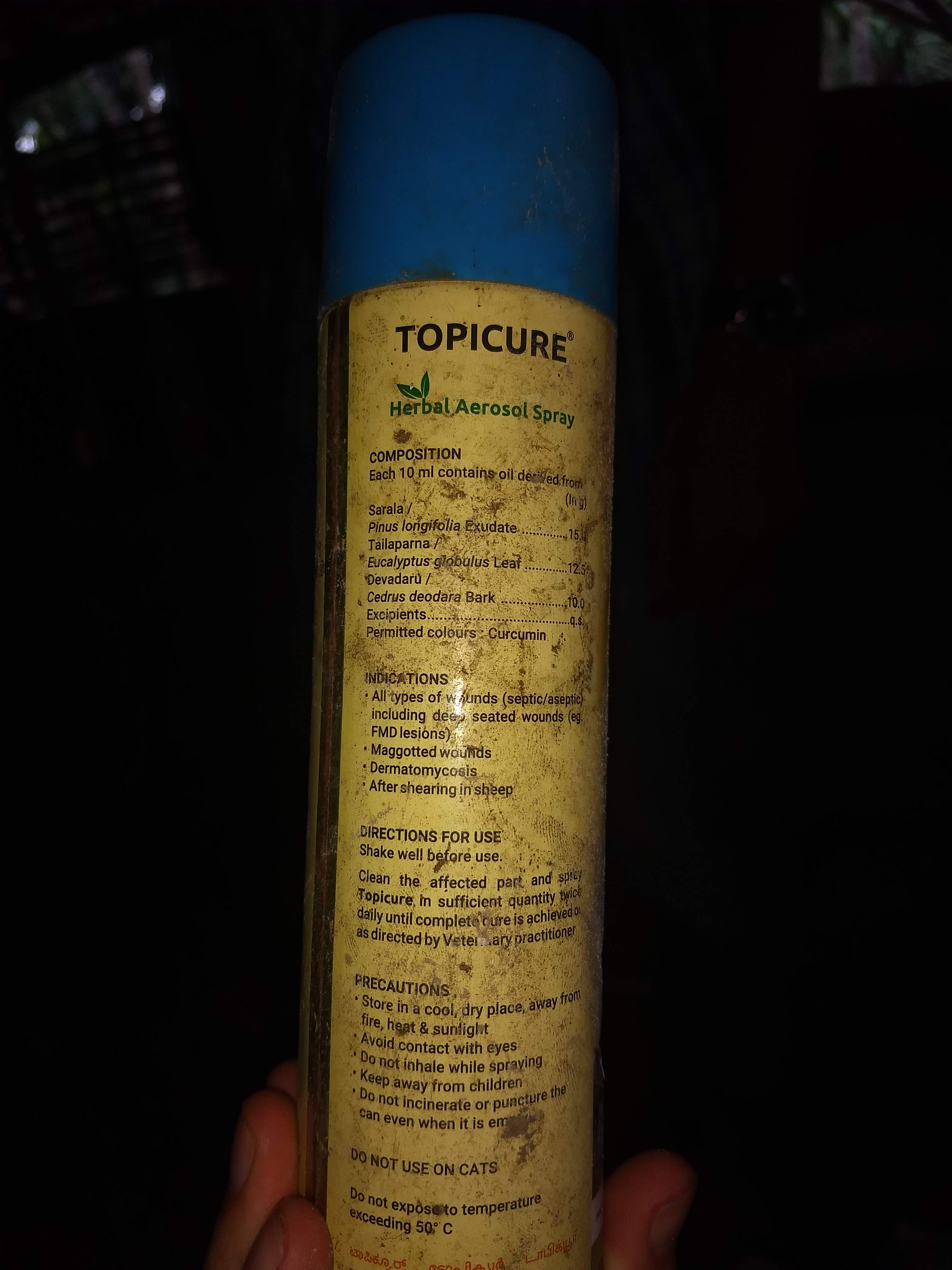

Supplement: Supplementary file 1 [file antibiotics-10-01433-s001.zip › Supplemrnrtary S2_ Site Photographs/Non-antibiotic wound medicine 6-household (site 1).jpg]

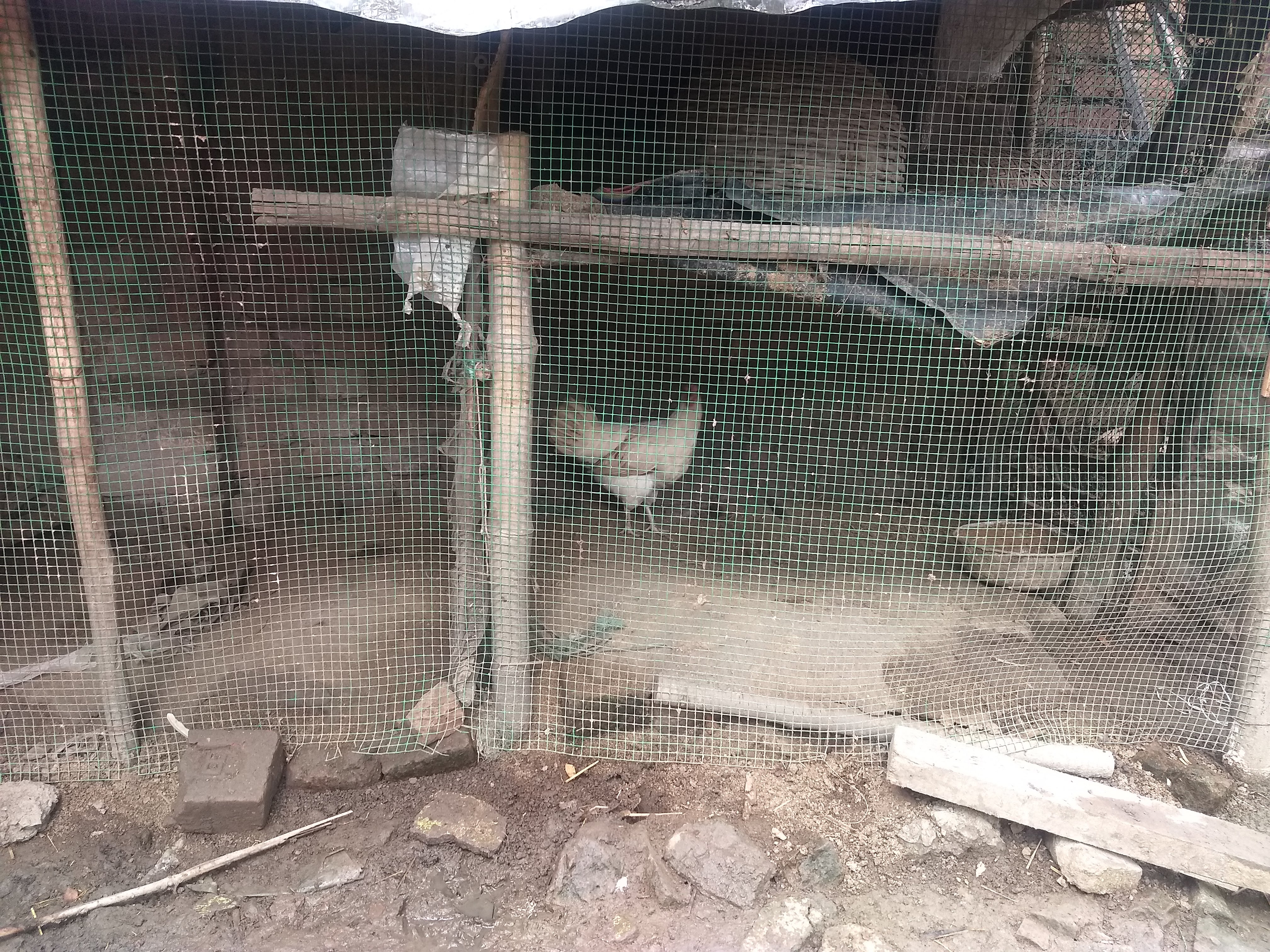

Supplement: Supplementary file 1 [file antibiotics-10-01433-s001.zip › Supplemrnrtary S2_ Site Photographs/Outdoor poultry housing (site 1).jpg]

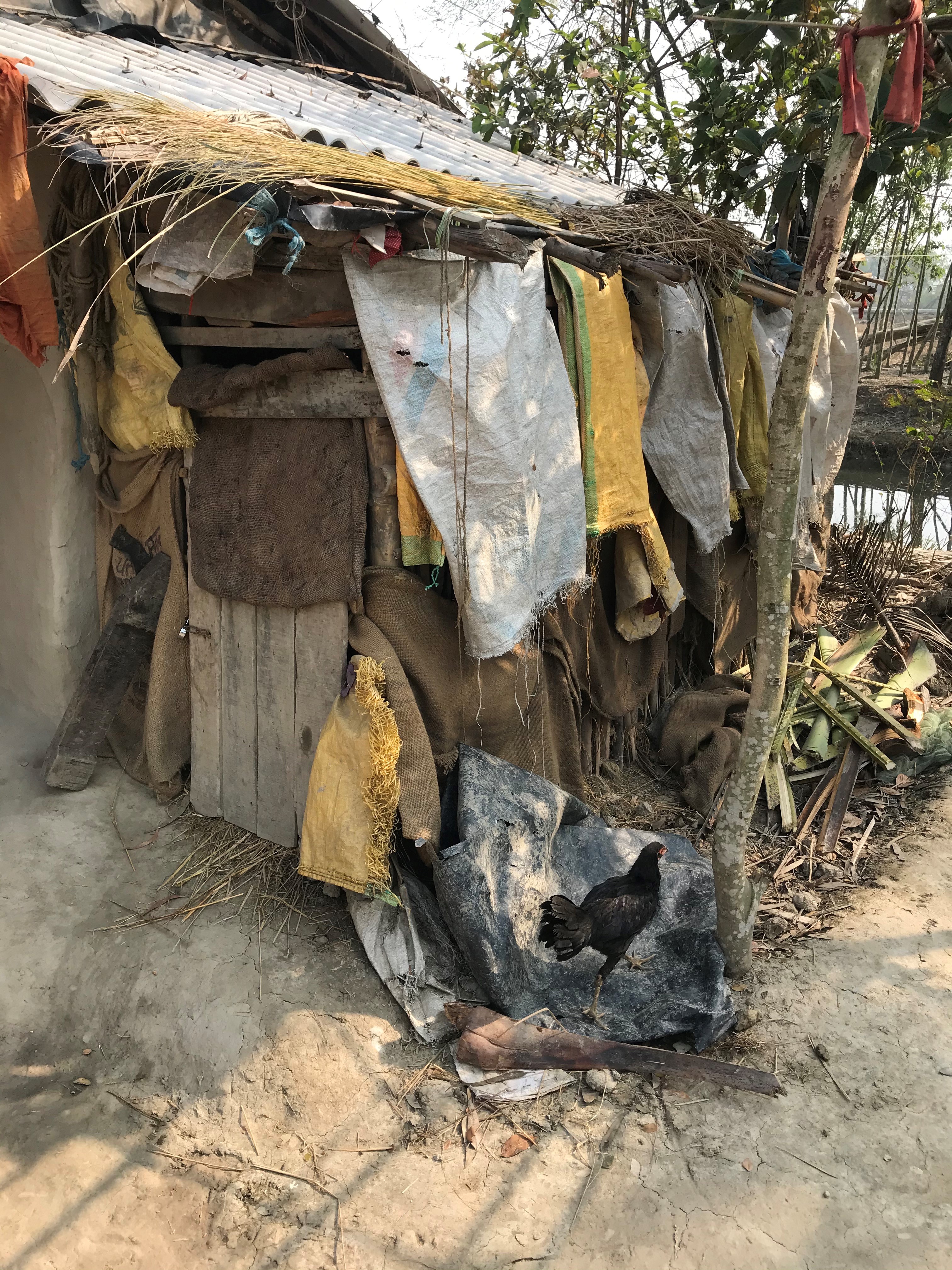

Supplement: Supplementary file 1 [file antibiotics-10-01433-s001.zip › Supplemrnrtary S2_ Site Photographs/Poultry housing (site 2).jpg]

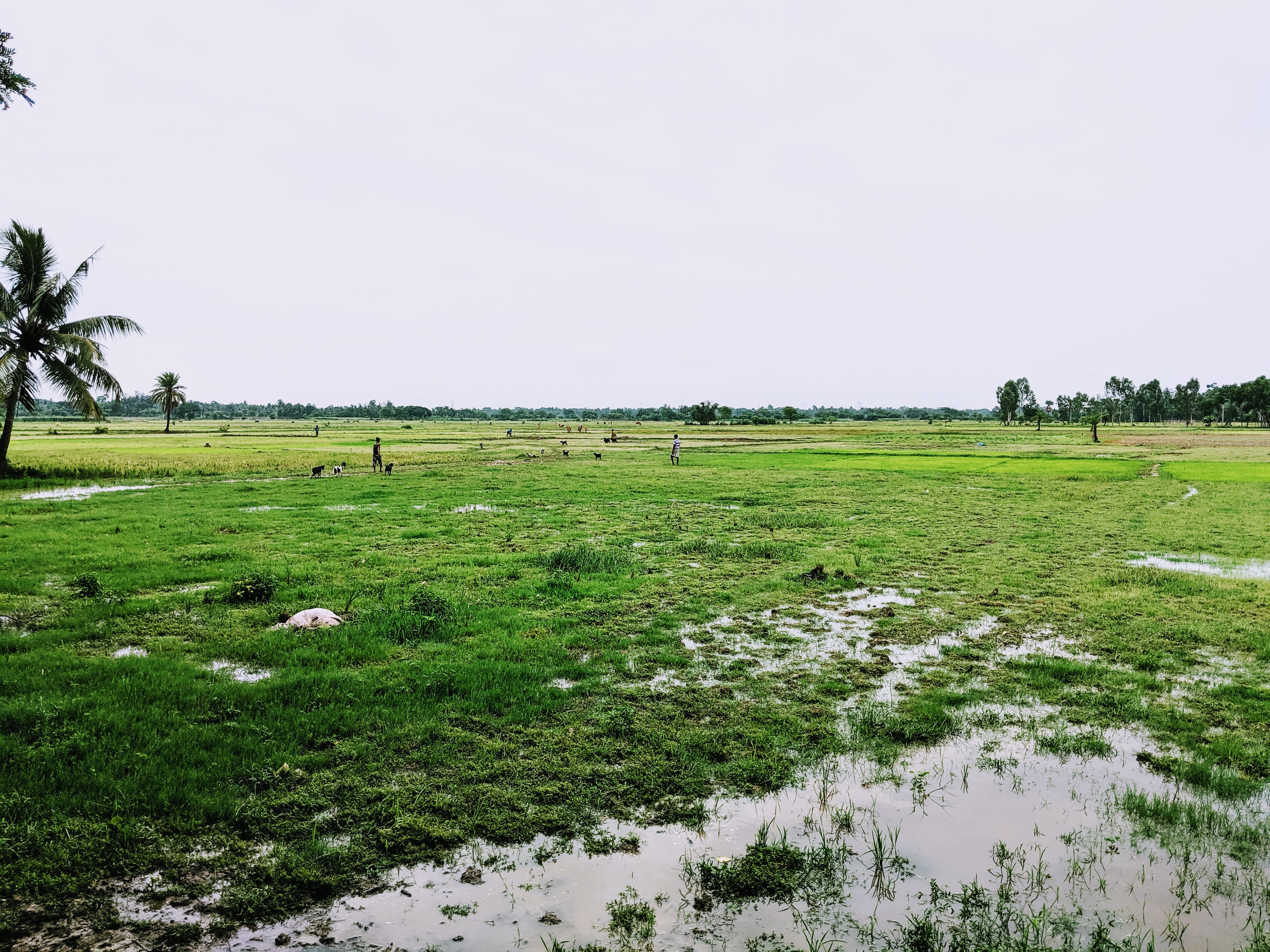

Supplement: Supplementary file 1 [file antibiotics-10-01433-s001.zip › Supplemrnrtary S2_ Site Photographs/Rice paddy farming (site 1).jpg]

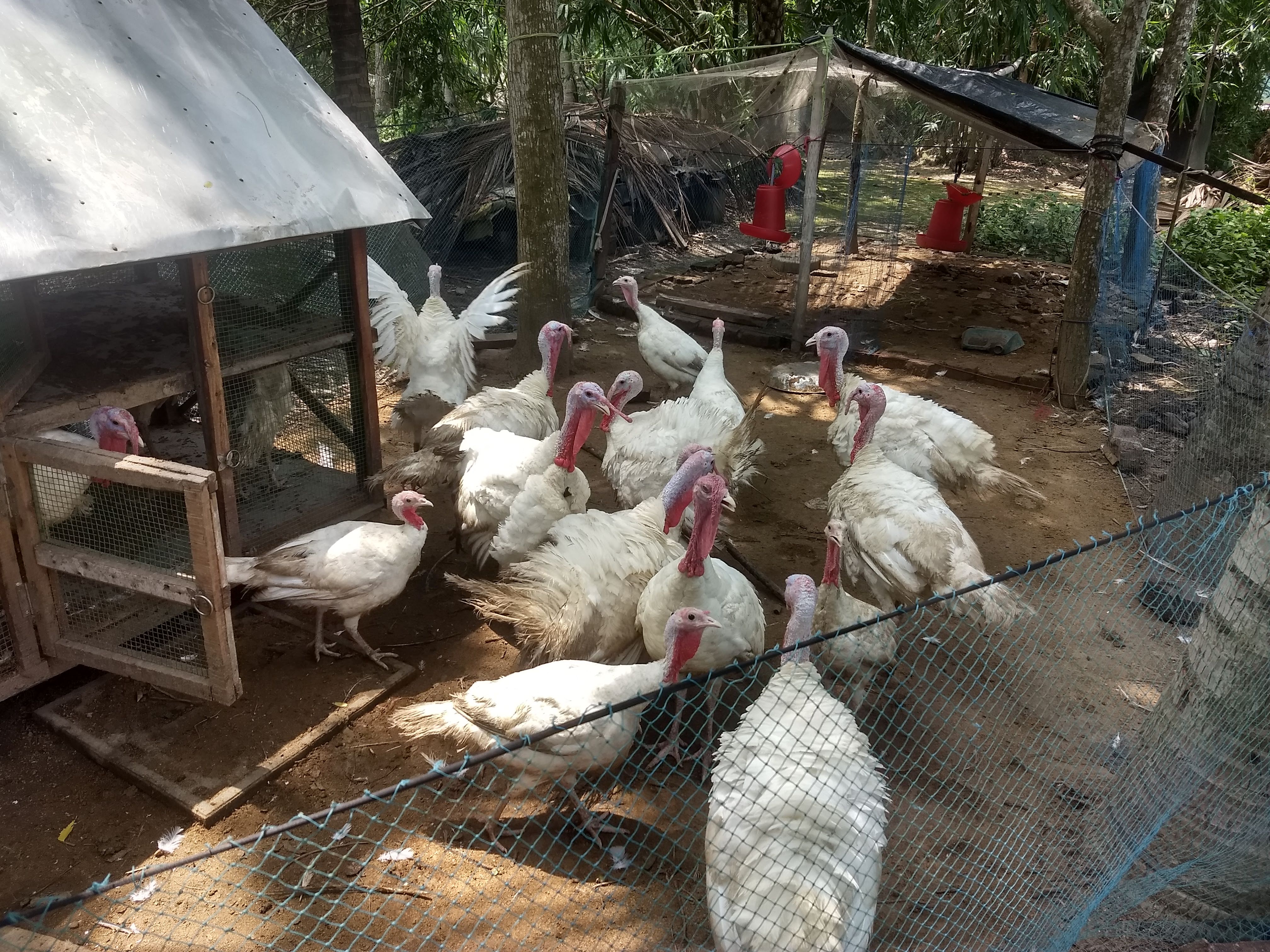

Supplement: Supplementary file 1 [file antibiotics-10-01433-s001.zip › Supplemrnrtary S2_ Site Photographs/Small-scale poultry rearing 1 (site 1).jpg]

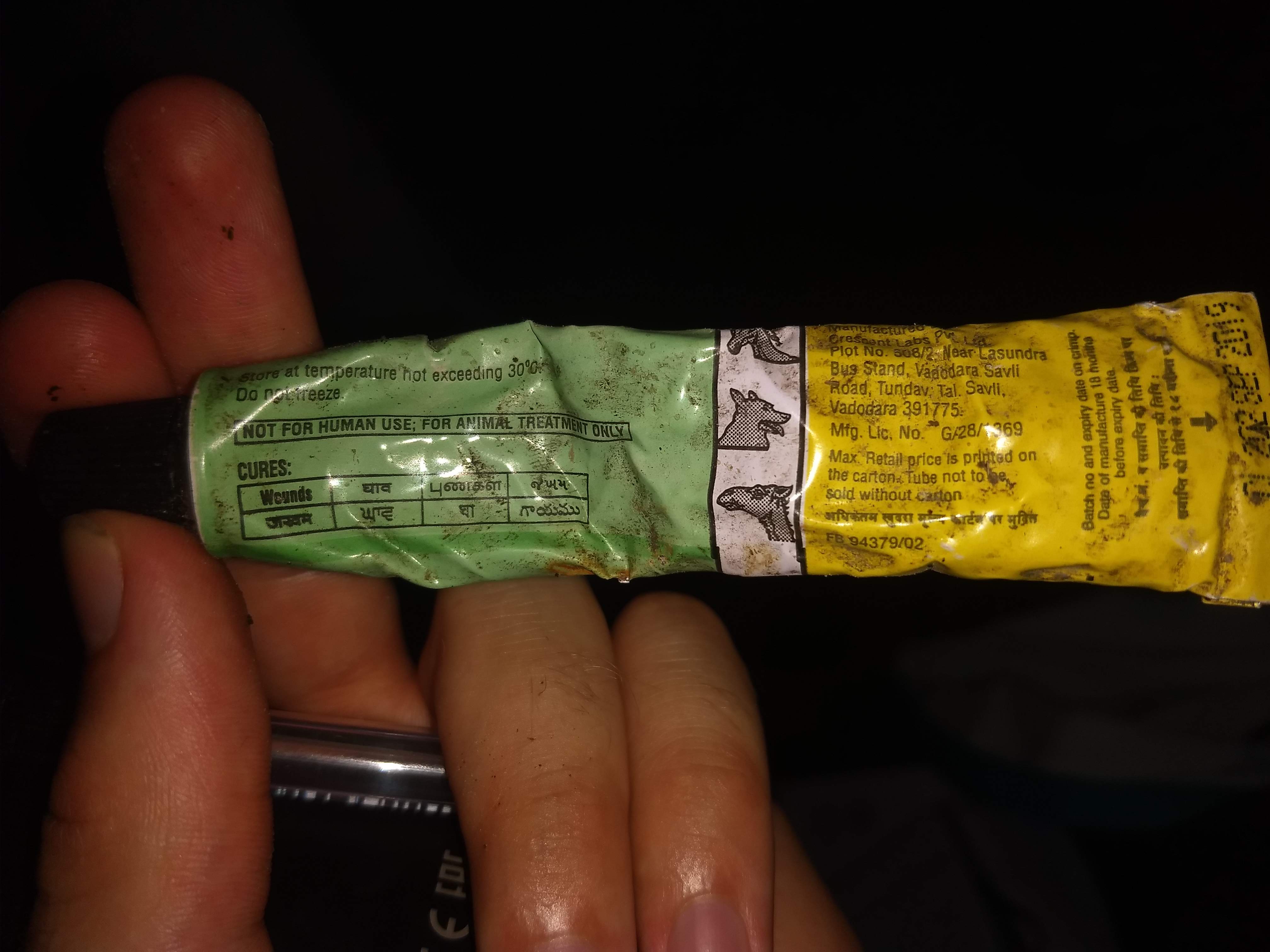

Supplement: Supplementary file 1 [file antibiotics-10-01433-s001.zip › Supplemrnrtary S2_ Site Photographs/vet antibiotic (neomycin-bacitracin) 1-household (site 1).jpg]

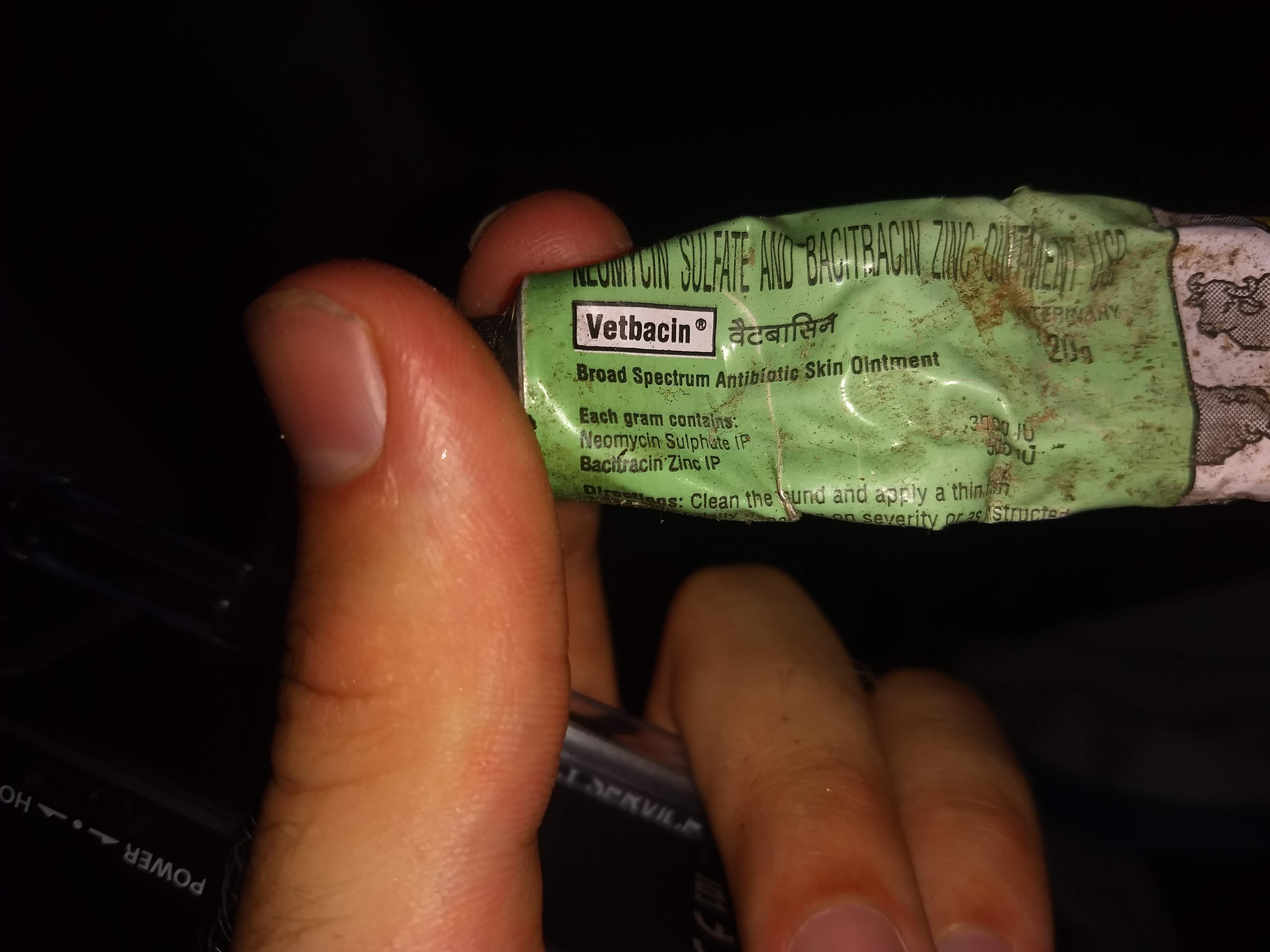

Supplement: Supplementary file 1 [file antibiotics-10-01433-s001.zip › Supplemrnrtary S2_ Site Photographs/vet antibiotic (neomycin-bacitracin) 2-household (site 1).jpg]

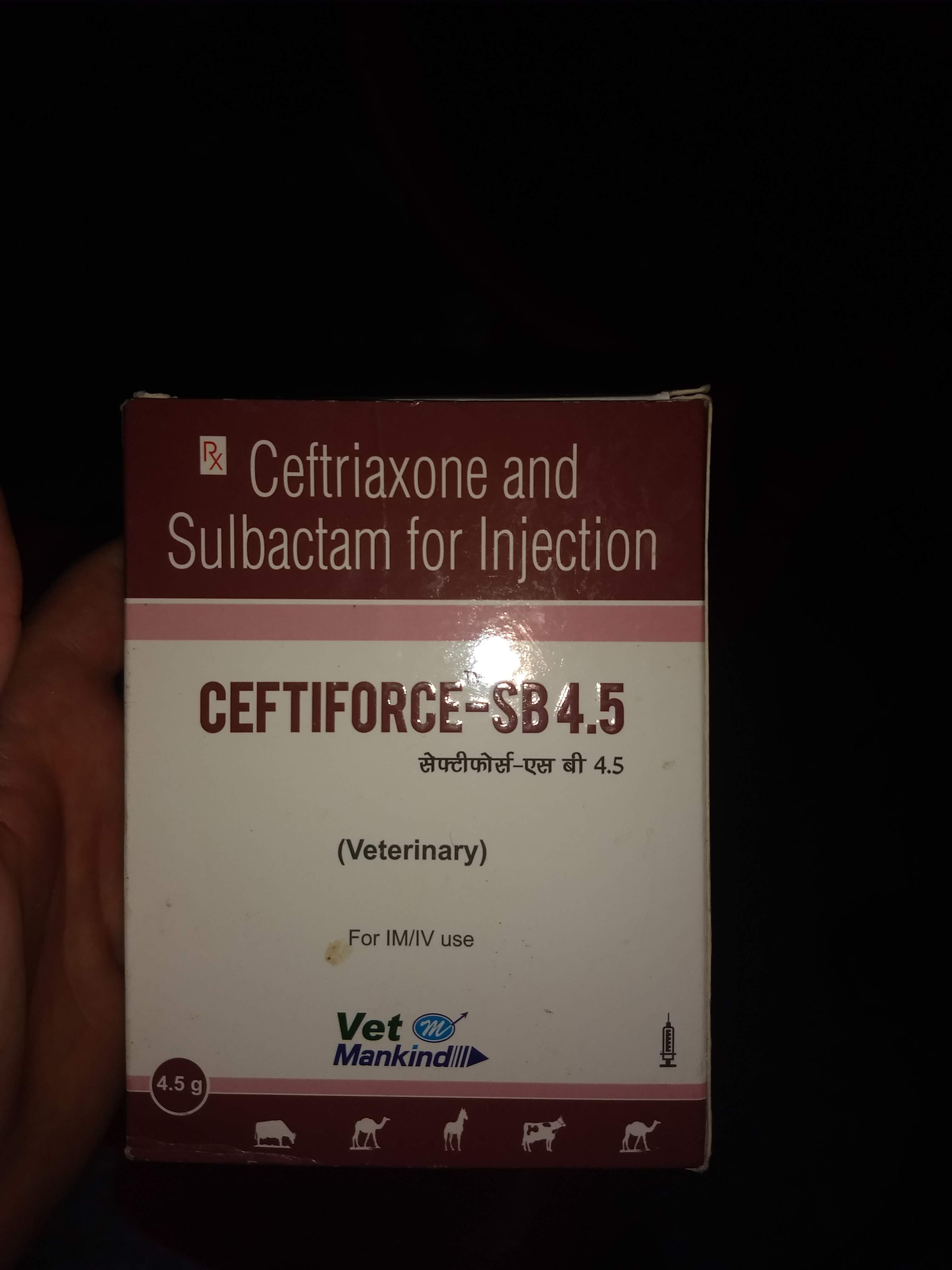

Supplement: Supplementary file 1 [file antibiotics-10-01433-s001.zip › Supplemrnrtary S2_ Site Photographs/Vet antibiotic 3 (ceftriaxone-sulbactam)- household (site 1).jpg]

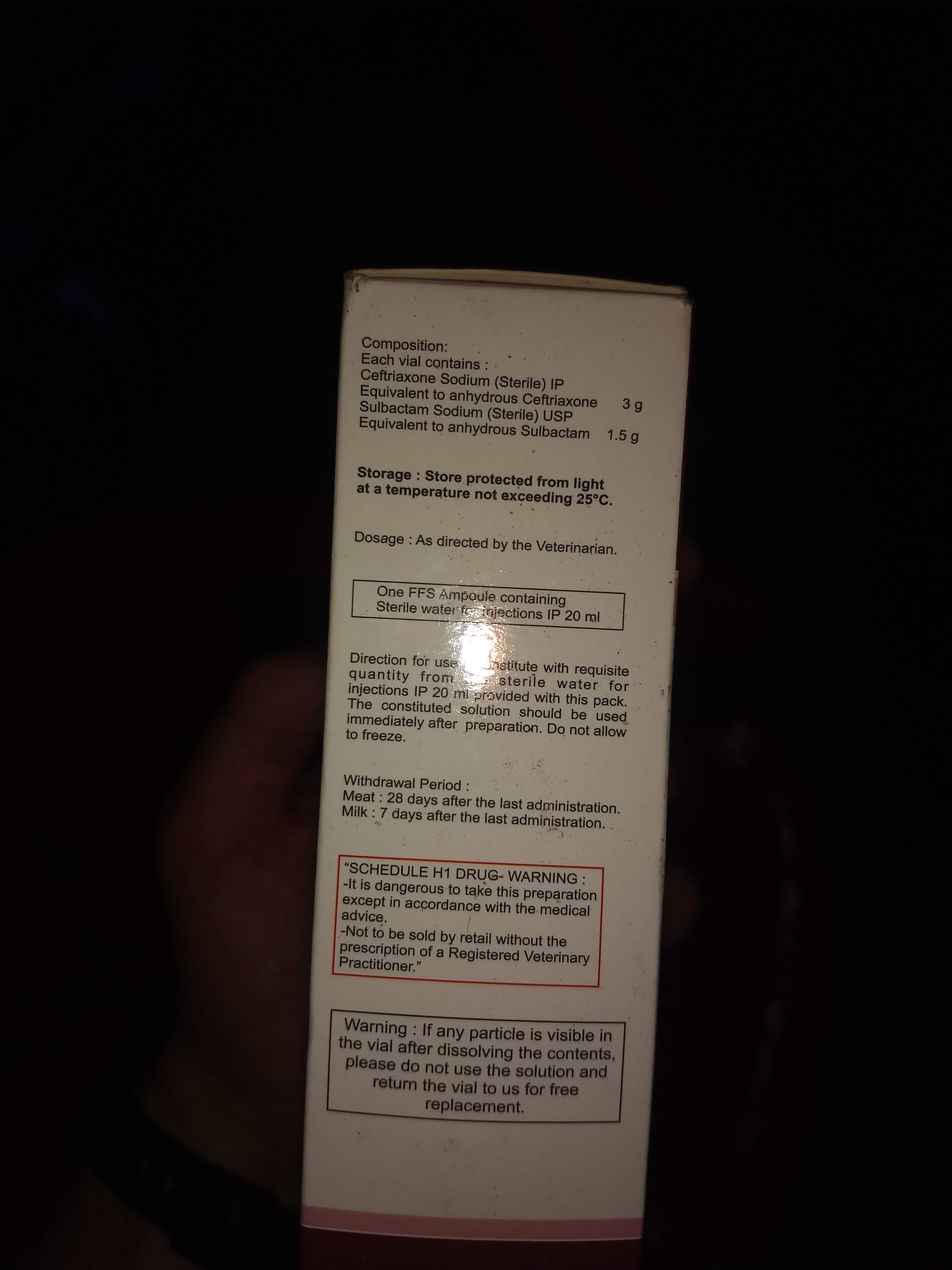

Supplement: Supplementary file 1 [file antibiotics-10-01433-s001.zip › Supplemrnrtary S2_ Site Photographs/Vet antibiotic 4 (ceftriaxone-sulbactam)- household (site 1).jpg]

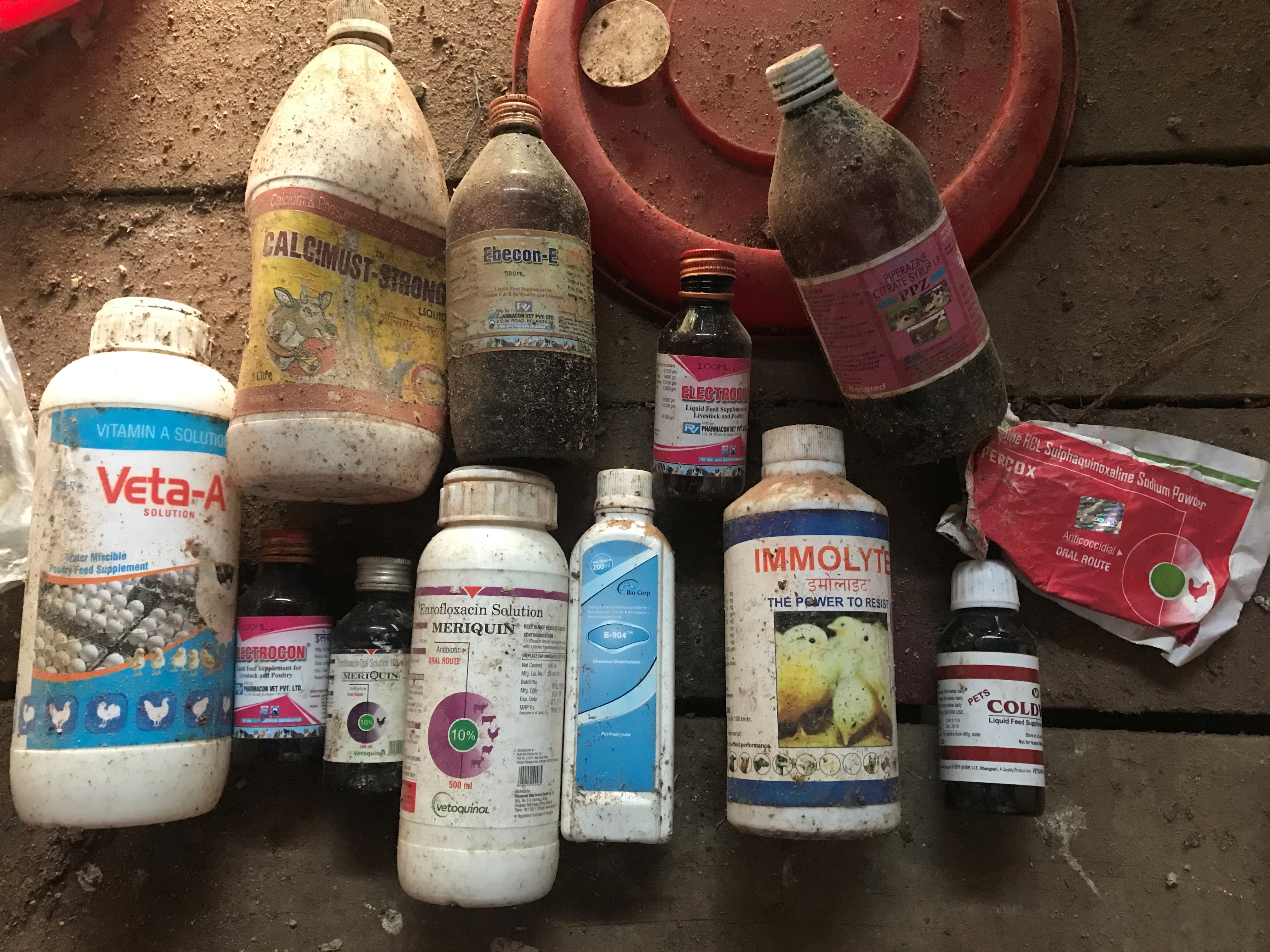

Supplement: Supplementary file 1 [file antibiotics-10-01433-s001.zip › Supplemrnrtary S2_ Site Photographs/Vet antibiotics and supplements-household (site 2).jpg]

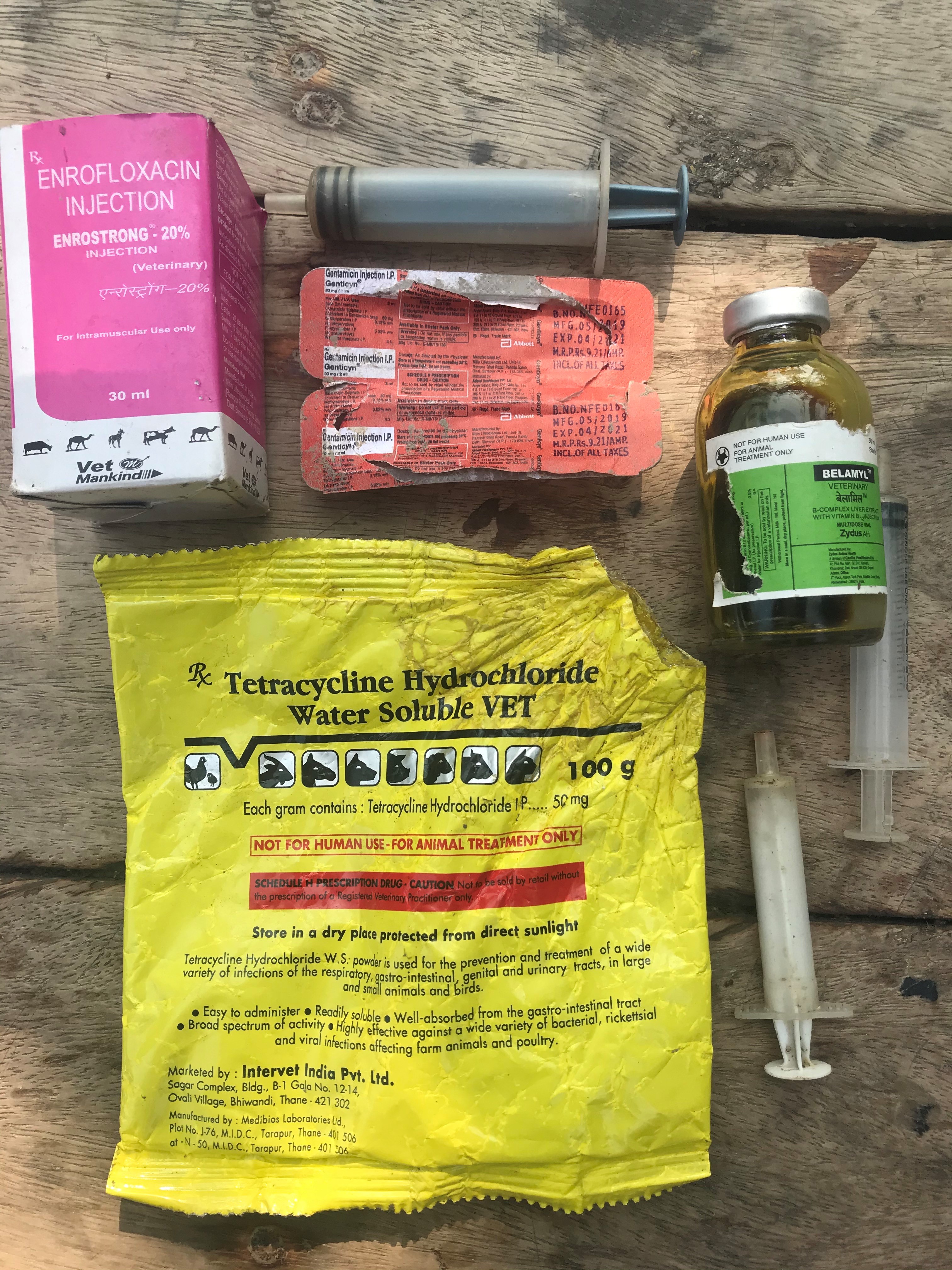

Supplement: Supplementary file 1 [file antibiotics-10-01433-s001.zip › Supplemrnrtary S2_ Site Photographs/vet antibiotics- household (site 2).jpg]

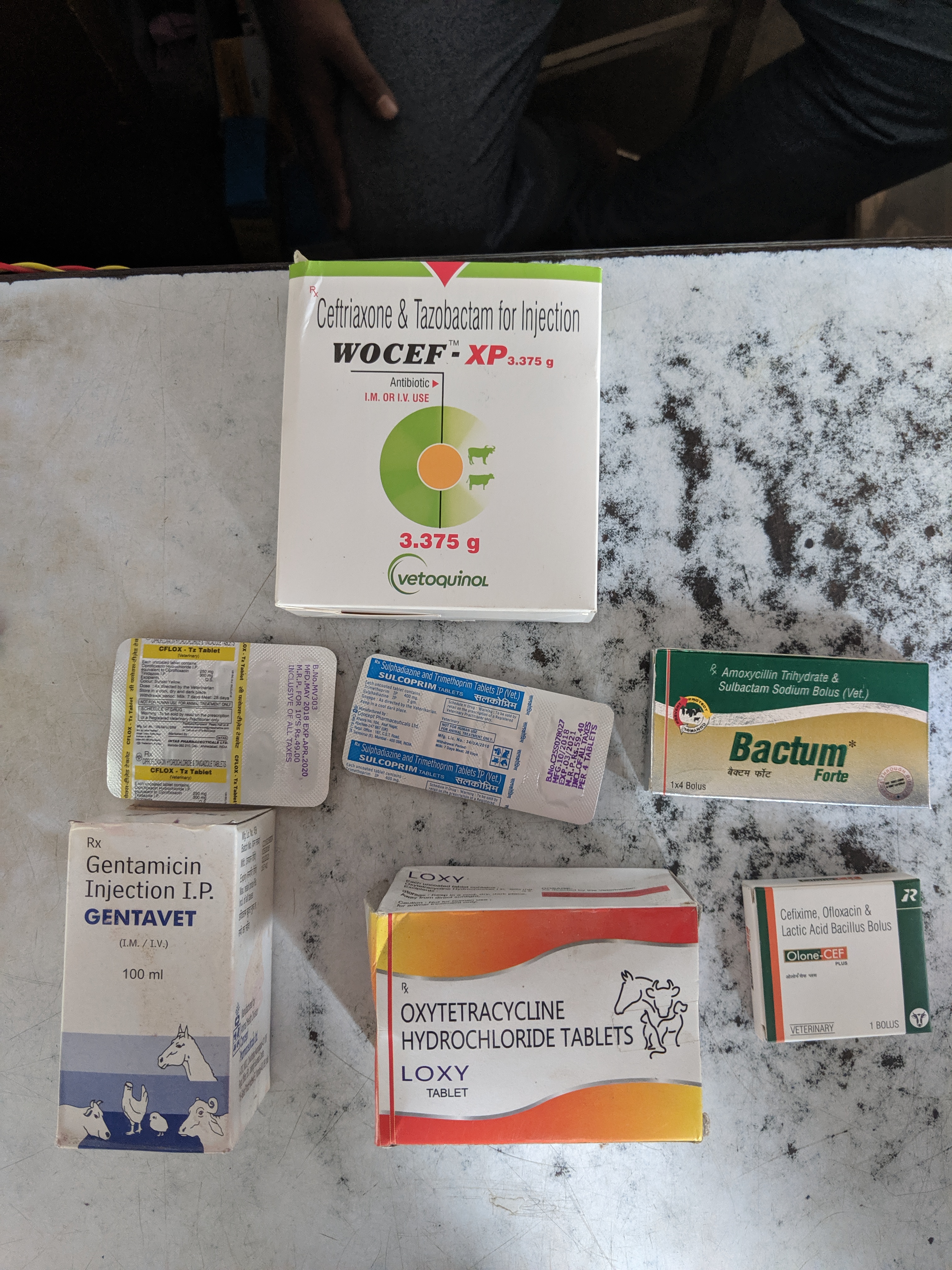

Supplement: Supplementary file 1 [file antibiotics-10-01433-s001.zip › Supplemrnrtary S2_ Site Photographs/Veterinary antibiotics from veterinary drug shop (site 1).jpg]

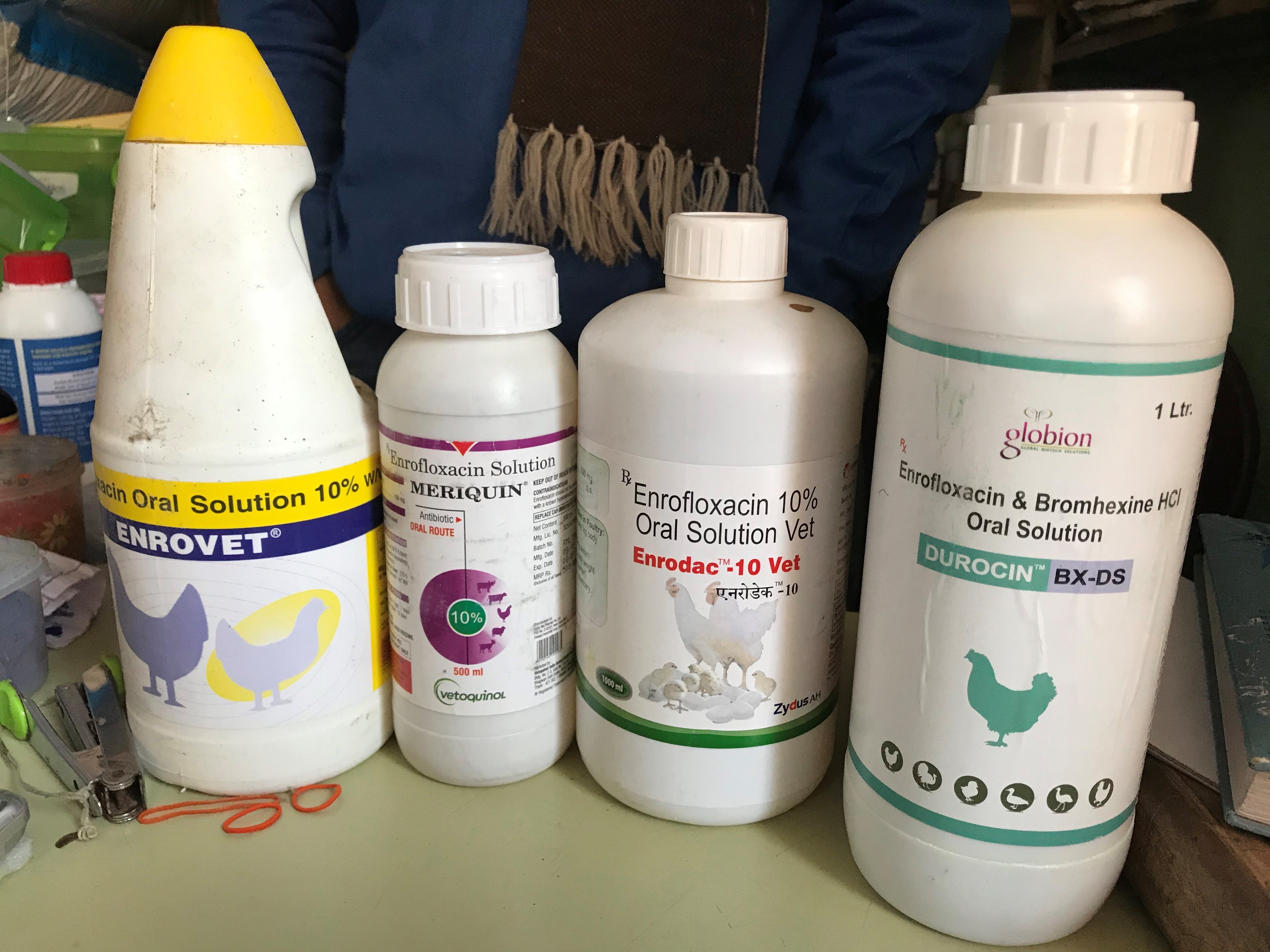

Supplement: Supplementary file 1 [file antibiotics-10-01433-s001.zip › Supplemrnrtary S2_ Site Photographs/Veterinary Enrofloxacin used by public veterinarian (site 2).jpg]

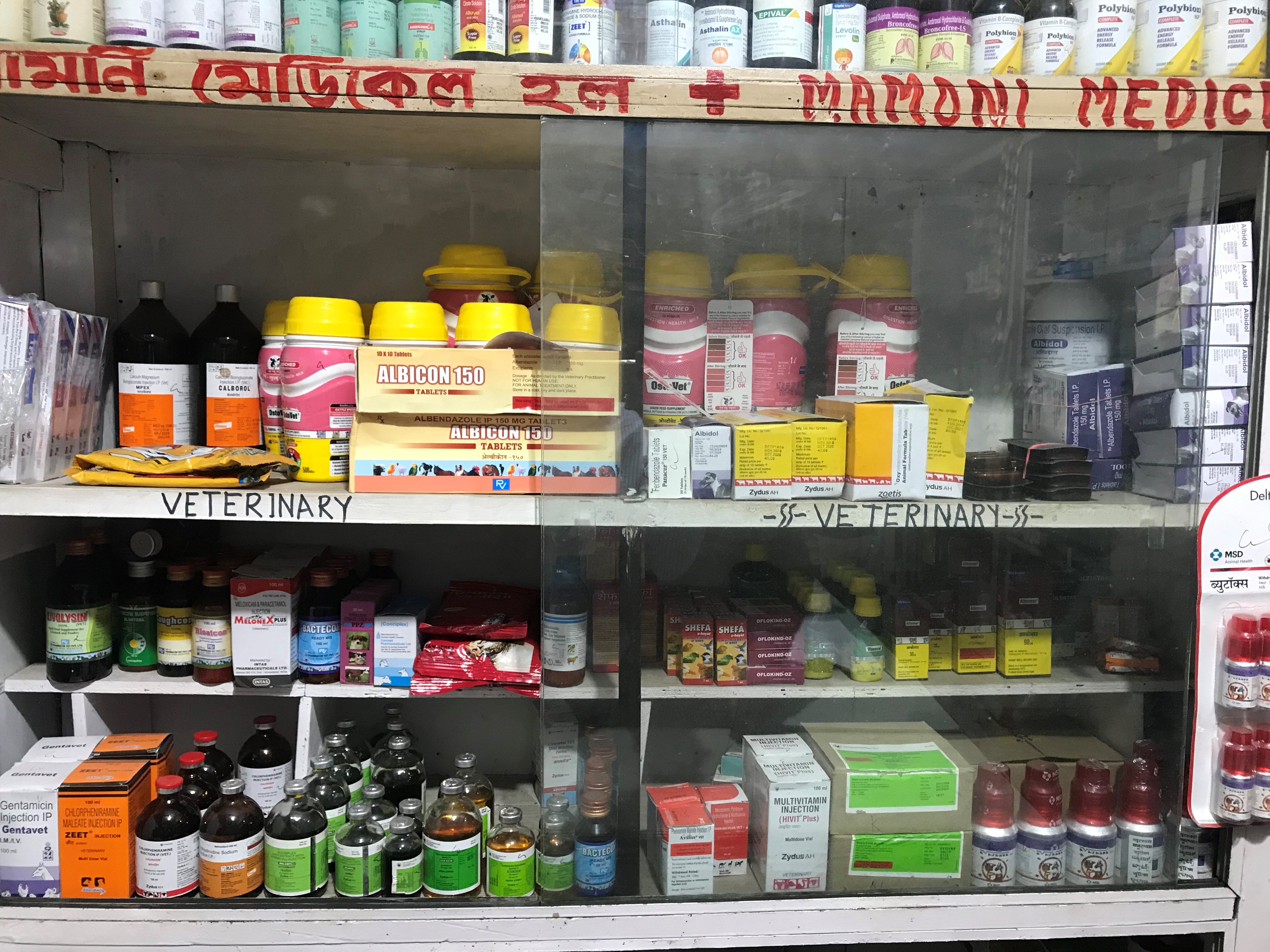

Supplement: Supplementary file 1 [file antibiotics-10-01433-s001.zip › Supplemrnrtary S2_ Site Photographs/Veterinary section of human drugs shop (site 2).jpg]

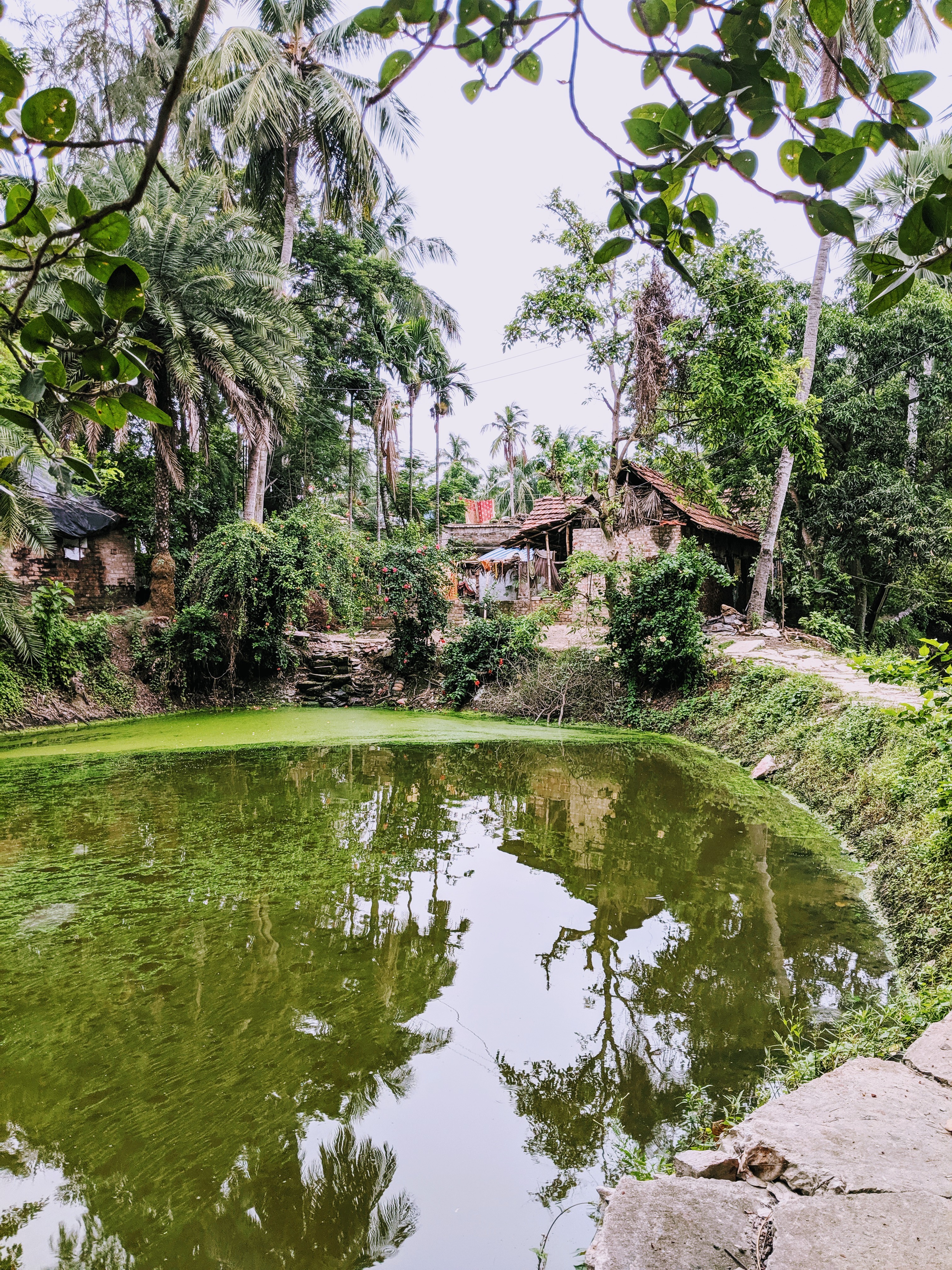

Supplement: Supplementary file 1 [file antibiotics-10-01433-s001.zip › Supplemrnrtary S2_ Site Photographs/Village pond 1(site 1).jpg]

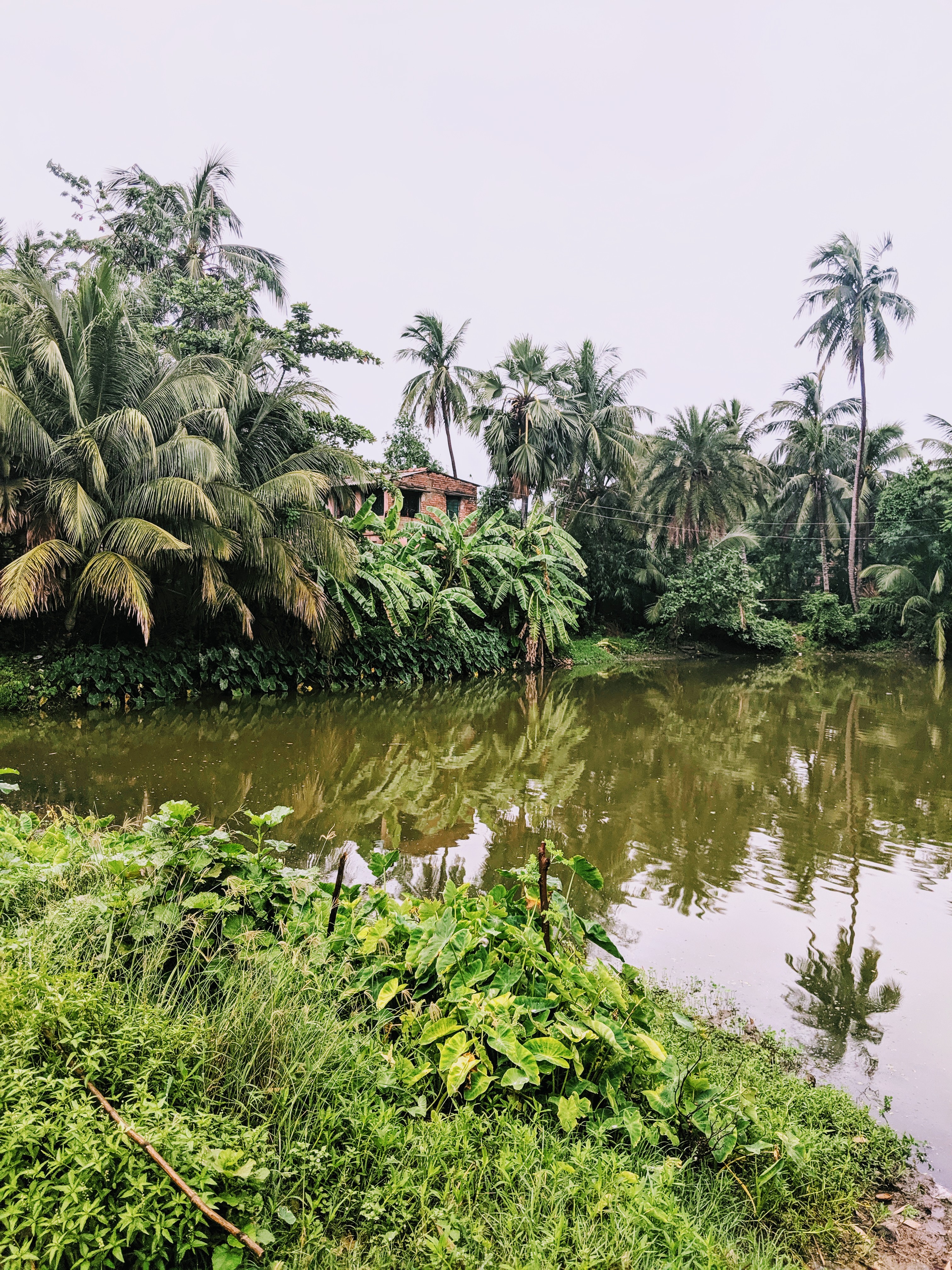

Supplement: Supplementary file 1 [file antibiotics-10-01433-s001.zip › Supplemrnrtary S2_ Site Photographs/village pond 2 (site 1).jpg]

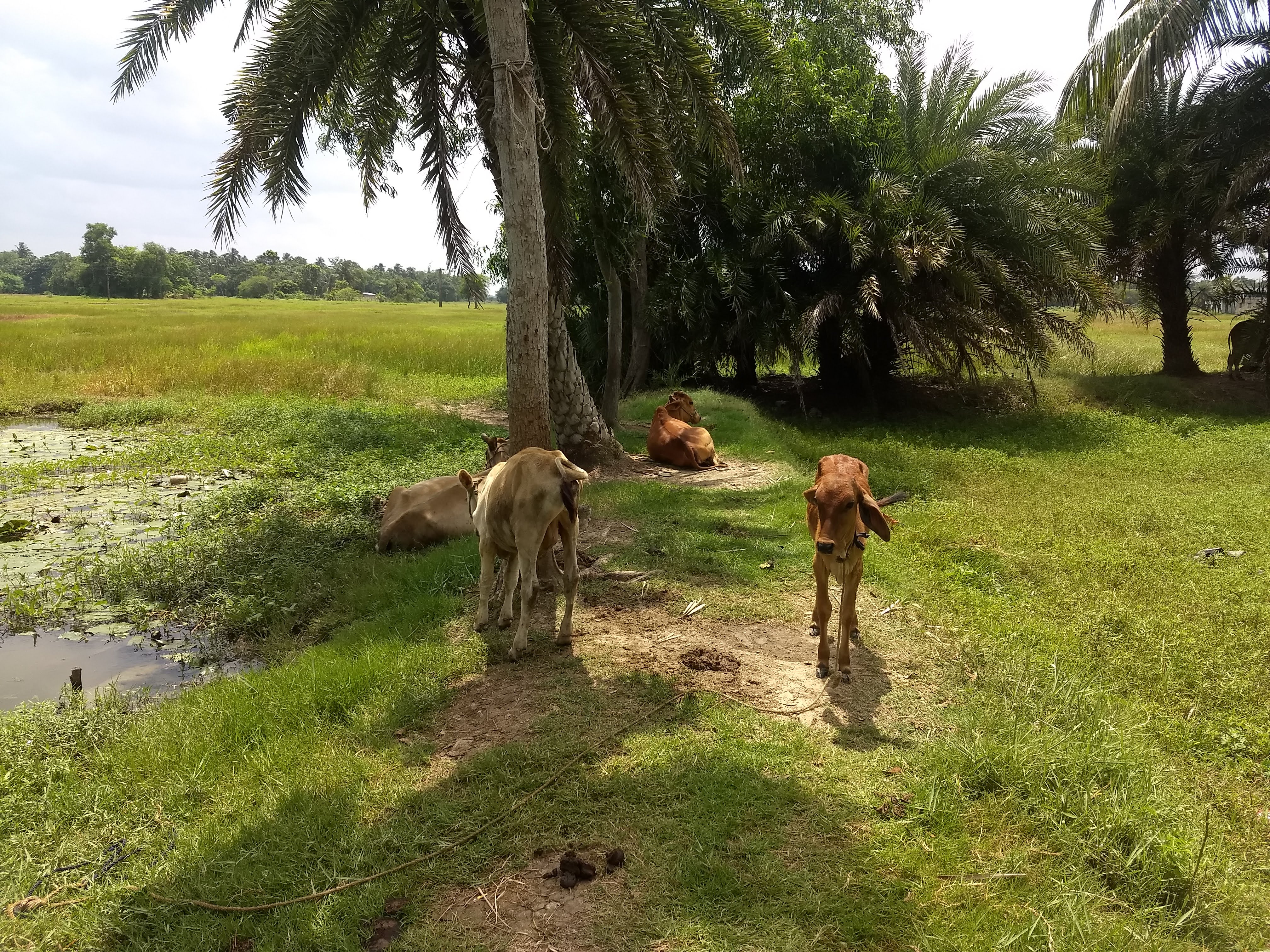

Supplement: Supplementary file 1 [file antibiotics-10-01433-s001.zip › Supplemrnrtary S2_ Site Photographs/Young cattle 1 (site 2).jpg]

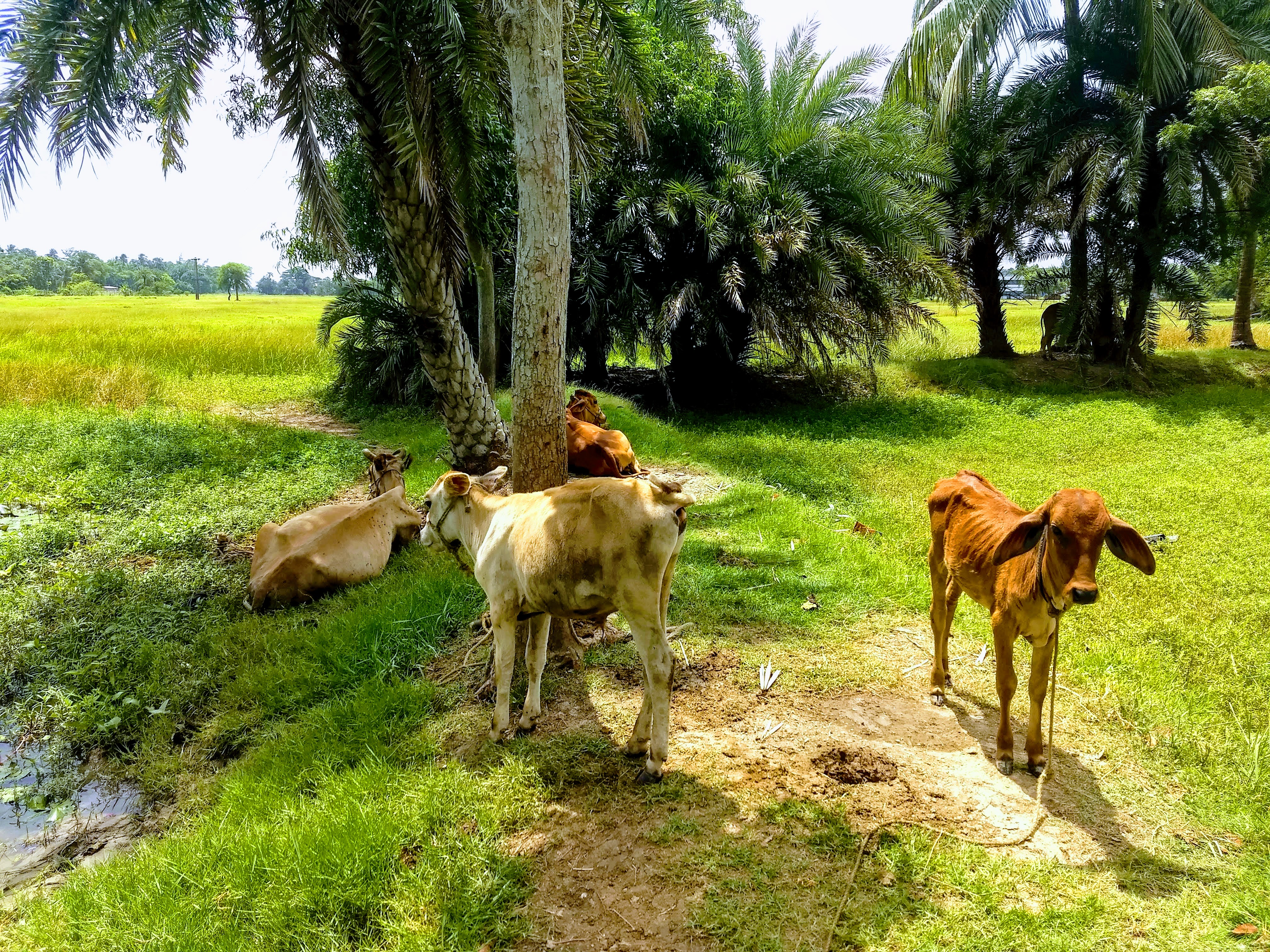

Supplement: Supplementary file 1 [file antibiotics-10-01433-s001.zip › Supplemrnrtary S2_ Site Photographs/Young cattle 2 (site 2).jpg]
